# Synthesis and Evaluation of a Non-Peptide Small-Molecule Drug Conjugate Targeting Integrin α_V_β_3_

**DOI:** 10.3389/fchem.2022.869639

**Published:** 2022-04-11

**Authors:** Jannik Paulus, Norbert Sewald

**Affiliations:** Organic and Bioorganic Chemistry, Department of Chemistry, Bielefeld University, Bielefeld, Germany

**Keywords:** integrins, RGD mimetics, linear conjugates, SAR study, SMDC, DAD mapping, α_V_β_3_

## Abstract

An integrin α_V_β_3_-targeting linear RGD mimetic containing a small-molecule drug conjugate (SMDC) was synthesized by combining the antimitotic agent monomethyl auristatin E (MMAE), an enzymatically cleavable Val-Ala-PABC linker with a linear conjugable RGD mimetic. The structure proposal for the conjugable RGD mimetic was suggested upon the DAD mapping analysis of a previously synthesized small-molecule RGD mimetic array based on a tyrosine scaffold. Therefore, a diversifying strategy was developed as well as a novel method for the partial hydrogenation of pyrimidines in the presence of the hydrogenolytically cleavable Cbz group. The small-molecule RGD mimetics were evaluated in an ELISA-like assay, and the structural relationships were analyzed by DAD mapping revealing activity differences induced by structural changes as visualized in dependence on special structural motifs. This provided a lead structure for generation of an SMDC containing the antimitotic drug MMAE. The resulting SMDC containing a linear RGD mimetic was tested in a cell adhesion and an *in vitro* cell viability assay in comparison to reference SMDCs containing cRGDfK or cRADfK as the homing device. The linear RGD SMDC and the cRGDfK SMDC inhibited adhesion of α_V_β_3_-positive WM115 cells to vitronectin with IC_50_ values in the low µM range, while no effect was observed for the α_V_β_3_-negative M21-L cell line. The cRADfK SMDC used as a negative control was about 30-fold less active in the cell adhesion assay than the cRGDfK SMDC. Conversely, both the linear RGD SMDC and the cRGDfK SMDC are about 55-fold less cytotoxic than MMAE against the α_V_β_3_-positive WM115 cell line with IC50 values in the nM range, while the cRADfK SMDC is 150-fold less cytotoxic than MMAE. Hence, integrin binding also influences the antiproliferative activity giving a targeting index of 2.8.

## 1 Introduction

**FIGURE 1:**
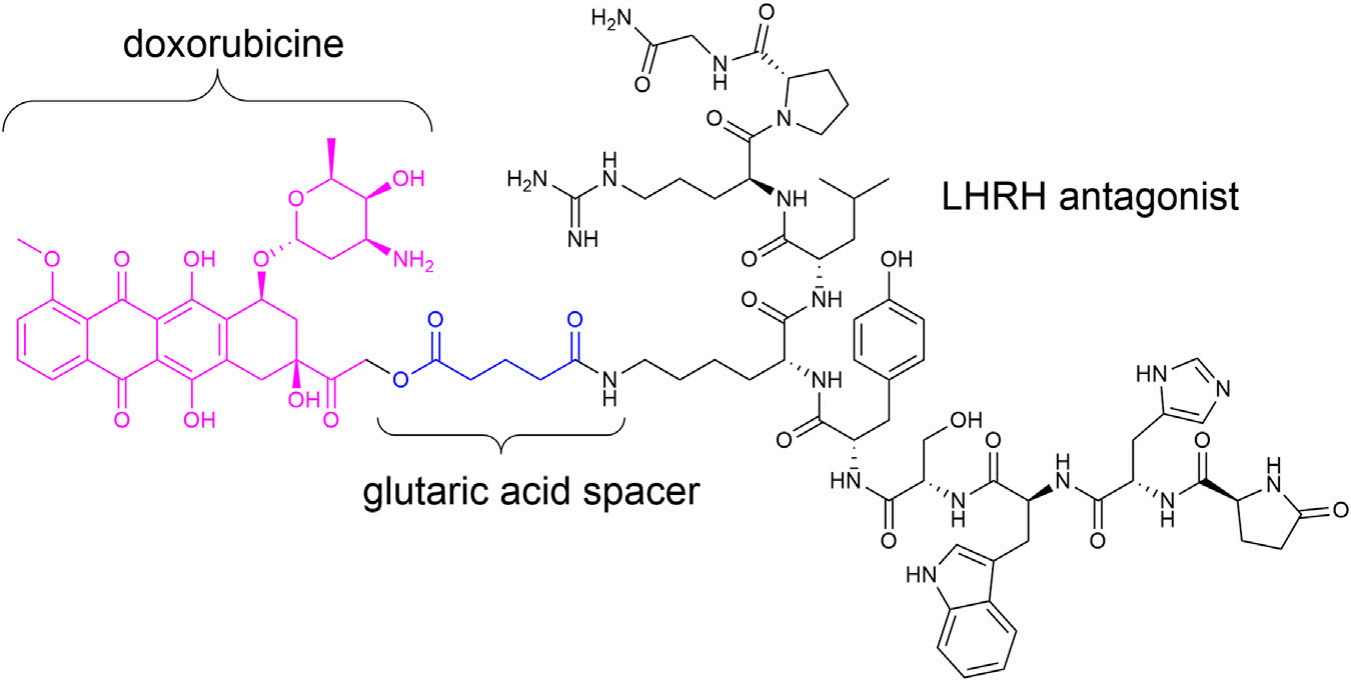
Chemical structure of Zoptrex™ (Nagy et al., 1996).

Targeted therapy devoid of side effects is a promising option in particular for cancer treatment. In this connection, antibody–drug conjugates (ADCs) (Gerber et al., 2009; Chari et al., 2014; Deneka et al., 2019; Hoppenz et al., 2020; Khongorzul et al., 2020) and small-molecule drug conjugates (SMDCs) (Srinivasarao et al., 2015; Deonarain et al., 2018; Hoppenz et al., 2020) were of great interest in the last decades. Such conjugates generally consist of a homing device (ADC: antibody; SMDC: small molecule/peptide), which addresses the desired receptor/cell and a payload (e.g., toxin) connected across a linker (stable or enzymatically/chemically cleavable) (Casi and Neri, 2015; Wei et al., 2018; Bargh et al., 2019; Hoppenz et al., 2020). Zoptarelin doxorubicin (Zoptrex™, Figure 1) is an example for an SMDC that reached clinical phase III for endometrial cancer treatment. It is composed of doxorubicin connected across a glutaric acid spacer to a small peptide agonist of the luteinizing hormone-releasing hormone (LHRH) receptor (Rékási et al., 1993; Nagy et al., 1996; Engel et al., 2012; Hoppenz et al., 2020).

Integrins are important targets for drug conjugates. They consist of an α and a β unit, which are non-covalently associated. Currently, there are 24 unique integrin heterodimers known, formed from 18 α and eight β subunits (Humphries et al., 2006; Barczyk et al., 2010; Cooper and Giancotti, 2019). The integrins are located in the cell membrane and operate as a bidirectional connection between the extracellular matrix (ECM) and the cytoplasmic domain to transmit signals in both directions. The ectodomain acts as an aerial or an anchor to receive signals from other cells or the ECM and link between cells (cell adhesion protein) (Bachmann et al., 2019). Hence, it is not surprising that integrins are involved in many important processes like cell proliferation, migration, and angiogenesis (Eliceiri and Cheresh, 1999; Franceschi et al., 2015), which makes them attractive as a target to modulate cellular control mechanisms. Integrin α_V_β_3_ is one of the most important representatives of the integrin family because of its significant impact in cellular processes (Giancotti and Ruoslahti, 1999; Hynes, 2002). It plays an important role in tumorigenesis because of its high expression level on tumor cells and its pro-angiogenic effect. This overexpression renders it a promising target in targeted cancer treatment. Therefore, integrin α_V_β_3_ is the target in a multitude of SMDCs (Nahrwold et al., 2013; Dal Corso et al., 2016; Borbély et al., 2019a; Borbély et al., 2019b), dye conjugates (Jin et al., 2017; Kemker et al., 2021), or difunctionalized ligands, which consists of an α-Gal epitope and an integrin-addressing moiety for redirecting endogenous and immunogenic antibodies to cancer cells (Owen et al., 2007).

The tripeptide sequence Arg-Gly-Asp (RGD) present in many integrin ligands is recognized by eight of the 24 integrin heterodimers (Barczyk et al., 2010; Nieberler et al., 2017), and it is considered a universal recognition motif for cell–cell and cell–ECM interactions. The selectivity for being recognized by a specific integrin is defined by the orientation, distance, and exposure of the essential residues and functional groups (Frank et al., 2010; Kapp et al., 2016; Kapp et al., 2017). Molecules which represent these properties and mimic the structural key elements are called RGD mimetics.

**FIGURE 2:**
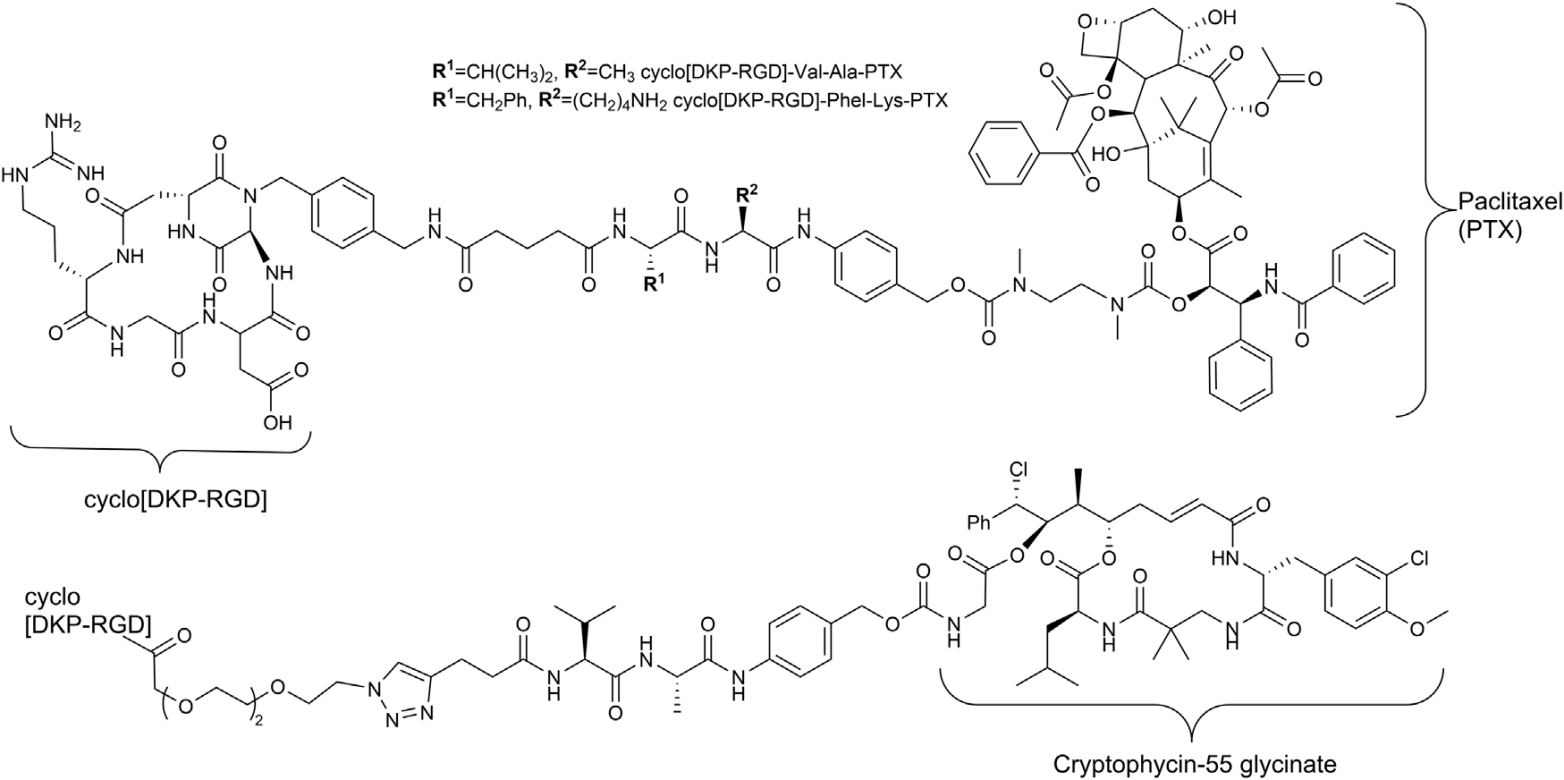
Structures of RGD containing SMDC with enzymatically cleavable linkers and paclitaxel (Pina et al., 2017) as well as cryptophycin-55 (Borbély et al., 2019a) as payloads.

Since the early 1990s, Kessler and his group developed cyclic pentapeptides (Aumailley et al., 1991; Gurrath et al., 1992; Haubner et al., 1997) first with a high affinity for the integrin α_V_β_3_ but a low selectivity against integrin α_5_β_1_, which is also a RGD recognizing integrin (Schaffner et al., 2013). Later, *iso*DGR peptides (Frank et al., 2010; Bochen et al., 2013; Mas-Moruno et al., 2016a) and linear tyrosine-based RGD mimetics were investigated (Heckmann et al., 2007; Heckmann et al., 2009). The group of DeGrado designed and synthesized linear RGD mimetics with high affinity and high selectivity against integrin α_5_β_1_ based on a diamine scaffold (Corbett et al., 1997; Rockwell et al., 1999). Most notably, in the last years, the cyclic cRGDfK peptide and its analogs [e.g., cyclo(*iso*DGR) and cyclo(DKP-RGD)] have been used as integrin α_V_β_3_-addressing homing devices in SMDCs (Figure 2) (Pina et al., 2017; Anselmi et al., 2020; Battistini et al., 2021; Lerchen et al., 2022).

## 2 Materials and Methods

### 2.1 General

Dichloromethane (DCM), petroleum ether, diethyl ether, and ethyl acetate were purchased at technical grade and distilled before usage. All other solvents were used as purchased (analytical grade). For further drying, DMF was stored over a molecular sieve (3 Å), and DCM was freshly distilled over CaH_2_ and THF over sodium. Inert reactions took place under an argon atmosphere and in baked-out equipment.

### 2.2 NMR Spectroscopy

NMR spectra were recorded on a Bruker Avance 600 (600 MHz for ^1^H, 564 MHz for ^19^F, and 150 MHz for ^13^C) and a Bruker Avance III 500HD (500 MHz for ^1^H, 126 MHz for ^13^C, and 471 MHz for ^19^F). The chemical shift δ is reported in ppm relative to the residual proton signal of the solvent: CDCl_3_ 7.26 ppm (^1^H) and 77.2 ppm (^13^C); DMSO-*d*_6_ 2.50 ppm (^1^H) and 39.52 ppm (^13^C); CD_3_OD 3.31 ppm (^1^H) and δ 49.0 ppm (^13^C). Two-dimensional methods (HMBC, HMQC, and COSY) were used to support and confirm the assignment.

### 2.3 LCMS and HRMS

LCMS analysis was performed by using an Agilent 6220 TOF-MS with a dual ESI source; 1200 HPLC system (Agilent) with an autosampler, degasser, binary pump, column oven, and diode array detector; and a Hypersil Gold C18 column (1.9 µm, 50 × 2.1 mm). The gradient started with 100% A (water/ACN/formic acid, 94.9:5:0.1); during 11 min, the percentage of eluent B (ACN/water/formic acid, 94.9:5:0.1) increased from 0 to 98% B and returned to 0% B in 0.5 min. The total run time was 15 min at a flow rate of 0.3 ml/min and a column oven temperature of 40°C. After separation *via* the 1200 HPLC system, ESI mass spectra were recorded in an extended dynamic range mode equipped with a dual-ESI source, operating with a spray voltage of 2.5 kV. The same system was used for high-resolution mass spectrometry.

### 2.4 Purification by Column Chromatography/RP-HPLC

Normal column chromatography was performed with silica gel (particle size: 40–60 µm) from Merck. Automatic column chromatography (MPLC, medium-performance liquid chromatography) was carried out with a Büchi Reveleris X2 system and purchased columns. Polar compounds and final products were purified *via* a preparative reverse-phase HPLC (RP-HPLC, Thermo Separation Products) consisting of a degasser, a pump (P4000), a Hypersil gold column (8 μm, 250 × 21.2 mm cartridge; Thermo Fisher Scientific) and a UV detector (UV1000). The gradients were chosen depending on the compound with eluents A (water/ACN/TFA, 94.9:5:0.1) and B (ACN/water/TFA, 94.9:5:0.1).

### 2.5 Synthesis

#### 2.5.1 General Procedure for Boc-Protection (GP-1)

Boc anhydride (1.2 eq) was dissolved in a mixture of water and dioxane and cooled to 0°C in an ice bath. Subsequently, the corresponding amino alcohol (1 eq.) was added, followed by addition of triethylamine (2 eq.). The reaction progress was monitored by TLC, and after full conversion, the reaction mixture was diluted with water and ethyl acetate. After phase separation, the water layer was extracted with ethyl acetate (3×), and the combined organic layers were washed with sat. NaCl (aq.) and dried over MgSO_4_. After evaporating the solvent, the desired *N*-Boc-protected amino alcohol was obtained as a highly viscous liquid and was used without further purification.

#### 2.5.2 General Procedure for Mitsunobu-Reaction (GP-2)

The corresponding amino alcohol (1.1 eq.), Cbz-Tyr-OMe (1 eq.), and triphenylphosphine (1.2 eq.) were dissolved in dry THF in baked-out equipment under inert conditions. The solution was cooled to 0°C in an ice bath, and diisopropyl azodicarboxylate (DIAD, 1.2 eq.), dissolved in THF (30 ml), was added dropwise during 1.5 h. After removing the cooling bath, the reaction mixture was stirred overnight at room temperature. Subsequently, the solution was diluted with sat. NaHCO_3_ (aq.), and the water layer was extracted with ethyl acetate (3×). The combined organic layers were dried over MgSO_4_, the solvent was removed under reduced pressure, and the crude product was purified by automatic column chromatography (MPLC, gradient of petroleum ether and ethyl acetate) to obtain the desired alkyl aryl ethers as a colorless foam.

#### 2.5.3 General Procedure for Cbz-Cleavage (GP-3)

The Cbz-protected compound (1 eq.) was dissolved in MeOH, and Pd (OH)_2_/C (10 % Pd, 0.1 eq.) was added to give a black suspension. Hydrogen was bubbled through the reaction mixture, and the reaction progress was monitored by LCMS. After full conversion, the suspension was filtered through a thin pad of celite. Afterward, the solvent was removed under reduced pressure, and the product was dried in vacuum to get the desired unprotected compound as a colorless solid.

#### 2.5.4 General Procedure for *N*-Terminal Modification of RGD Mimetic Precursors With Acid Chlorides (GP-4a)

Triethylamine (3 eq.) was added to a solution of the corresponding unprotected compound (1 eq.) in 2 ml DMF/DCM (1:1, v:v), and after stirring for 5 min, benzoyl chloride (1.5 eq.) was added. The reaction progress was monitored *via* LCMS, and after full conversion, the solvent was removed. Afterward, the crude was dried in vacuum, and the resulting solid was dissolved in a small amount of water/ACN (1:1, v:v) and purified *via* preparative RP-HPLC.

#### 2.5.5 General Procedure for *N*-Terminal Modification of RGD Mimetic Precursors With Carboxylic Acids (GP-4b)

HOBt solution (1.3 M in DMF, 1.3 eq.) was added to the corresponding 4- or 3-hydroxy benzoic acid (2.4 M in DMF, 1.2 eq.), followed by an EDC solution [0.5 M in DMF/DCM (1:1, v:v), 1.4 eq.] and DIPEA (2 eq.). The solution was stirred for 2 mins and was then added to the solution of the amino component **3a-c** (1 eq.) in DMF/DCM (1:1, v:v, 1 ml). The reaction mixture was stirred at room temperature, and the reaction progress was monitored by LCMS. After full consumption of the amine, the reaction was stopped by removing the solvent. Afterward, the crude was dried in vacuum, and the resulting solid was dissolved in a small amount of water/ACN (1:1, v:v) and purified *via* preparative RP-HPLC.

#### 2.5.6 General Procedure for the Synthesis of Final RGD Mimetics—DHI Substituted (GP-5a)

HCl in dioxane (4 M, 100 μl, 13.3 eq.) was added to a solution of protected RGD mimetic precursors **5a-c**, **6a-c**, and **7a-c** (0.1 M in DCM, 300 μl, 1 eq.). After stirring for 1.5 h, the solvent was removed under reduced pressure, and a solution of 2-methylthio-2-imidazoline hydroiodide (0.18 M in MeOH/NEt_3_ 1:1, v:v, 416 μl, 2.5 eq.) was added. The mixture was heated to 80°C in a sealed tube till consumption of the free amine (LC-MS), followed by solvent removal. The residue was then dissolved in a LiOH solution [0.285 M in MeOH/water (3:1, v:v), 526 μl, 5 eq.] and stirred at room temperature. Monitoring of the reaction progress was done *via* LCMS. After complete conversion, the crude mixture was concentrated and purified *via* preparative RP-HPLC.

#### 2.5.7 General Procedure for the Synthesis of Final RGD Mimetics—Pyrimidine and THP Substituted (GP-5b-d)

It used **GP-5a** with 2-bromopyrimidine (0.36 M in MeOH/NEt_3_ 1:1, v:v, 416 μl, 5 eq.) instead of 2-methylthio-2-imidazoline hydroiodide. After ester hydrolysis, the reaction batch was split into two equal amounts (1. **GP-5b** and 2. **GP-5c** or **GP-5d**), and the following procedure was applied:

**GP-5b** for pyr-substituted mimetics: Half of the reaction mixture was concentrated and purified *via* preparative RP-HPLC to obtain the pyrimidine-substituted final RGD mimetic.

**GP-5c** for THP in case of benzoyl-substituted mimetics: Half of the reaction mixture was combined with a suspension of Pd/C (10 mg ml^−1^, 234 µl) and 100 µl acetic acid. Afterward, hydrogen was bubbled through the suspension till LC-MS showed full conversion. The reaction mixture was concentrated, centrifuged, and purified by preparative RP-HPLC to obtain the desired reduced RGD mimetic as TFA salt.

**GP-5d** for THP in case of Cbz-protected mimetics: Half of the reaction mixture was combined with Pd/C (10 % Pd, 0.1 eq.), 2-bromo-pyrimidine (10 eq.), HBr in AcOH (10 eq.), AcOH (200 eq.), and water (400 eq.) in MeOH to result in a 10 mM solution based on the half of the starting material. Hydrogen was bubbled through the suspension upon vigorous stirring till LC-MS showed full conversion. The reaction mixture was centrifuged, the solid residue was discarded, and the solution was diluted with water and freeze-dried. Afterward, the residue was purified by preparative RP-HPLC to obtain the desired reduced RGD mimetic as TFA salt.

#### 2.5.8 Solid-Phase Peptide Synthesis (GP-6)

**Resin loading:** The Fmoc/^t^Bu strategy was chosen for the synthesis of linear peptides and peptide-based enzymatically cleavable linkers. Resin loading and subsequent coupling steps were performed in a syringe and on an automatic shaker. Barlos/2-chlorotrityl chloride resin (CTC-resin, 1.5 mmol/g) was swollen in DCM (10 ml/g resin) for 10–15 min at room temperature. Afterward, the solvent was removed, and a solution of the loading amino acid (1 eq. corresponding to resin) and DIPEA (10 eq.) in DCM (10 ml/g resin) was added. After incubation for 3 h at room temperature, MeOH (2 ml/g resin) was added, and the mixture was shaken for further 30 min. Then the resin was washed with DMF (5×) and DCM (3×) and dried in vacuum to determine the resin loading by a UV analysis of the piperidine–dibenzofulvene adduct formed upon cleavage of the Fmoc-protecting group with 20% piperidine in DMF.

**Fmoc cleavage and coupling of amino acids**: After resin loading, the resin was swollen in DMF for 10 min. Fmoc cleavage was performed twice with 20% piperidine in DMF (4 min in ultrasonic bath at 25°C, followed by 5 min on a shaker at r.t., 5 ml/g resin) and washing with DMF (5 × 10 ml/g resin), DCM (2 × 10 ml/g resin), and DMF (2 × 10 ml/g resin). For the coupling step, the corresponding amino acid (4 eq.), DIC (4 eq.), and oxyma (4 eq.) were dissolved in DMF (10 ml/g resin) and added to the reaction syringe containing the resin, followed by sonication for 4 min and further shaking for 5 min. Afterward, the resin was washed again with DMF (5 × 10 ml/g resin), DCM (2 × 10 ml/g resin), and DMF (2 × 10 ml/g resin). The coupling result was checked by the Kaiser test or analysis by LCMS after test cleavage. For the analysis, the resin was washed with DMF (5×) and DCM (3×) and dried in vacuum, and then approximately 10 beads were transferred into an Eppendorf tube and treated either with the reagents for the Kaiser test or with a mixture of TFA/TIS/MPW (95:2.5:2.5; 100 µl) in case of the test cleavage. After incubation for 5 min, the test cleavage was diluted with 500 µl of ACN/MPW (1:1) and analyzed by LCMS.

**Cleavage from resin:** Unless otherwise stated, the resin was swollen in DCM and treated 10 times with 1% TFA in DCM (5 ml). The resulting cleavage cocktail was passed into prepared *iso*-propanol, followed by evaporating the solvent and precipitation in Et_2_O. After centrifugation, the resulting pellet was separated from the liquid residue and dried in vacuum.

#### 2.5.9 Head-To-Tail Cyclization of Linear Peptides (GP-7)

The crude linear peptide was cyclized under pseudo-high-dilution conditions (Malesevic et al., 2004) without prior purification after cleavage. A solution of the peptide (1 eq.) in DMF and another solution with HATU (1.3 eq.) and HOAt (1.3 eq.) in DMF was prepared and added from two separate syringes to a solution of HATU (0.1 eq.), HOAt (0.1 eq.), and DIPEA (3 eq.) in DMF. The total DMF volume was chosen for a final peptide concentration of 10 mM. The peptide solution and the coupling reagent solution were added at a flow rate of 1.25 ml/h simultaneously to the stirred solution. After complete addition, stirring was continued overnight at room temperature. The solvent was evaporated in vacuum, followed by precipitation of the residue in Et_2_O. The resulting pellet was dried and purified by normal-phase column chromatography (DCM/MeOH).

#### 2.5.10 Allyl-Deprotection and Introduction of Linker Units to Cyclic RGD Mimetics (GP-8)

The resin was swollen in DMF (10 ml/g resin) and degassed by bubbling Ar through the suspension for 1 h, followed by addition of Pd (PPh_3_)_4_ (0.1 eq.) and 1,3-dimethylbarbituric acid (DMBA, 4 eq.). After 30-min shaking under inert conditions, the cleavage cocktail was removed, and the cleavage was repeated for further 30 min with fresh reagents. The resin was washed with DMF (5 × 10 ml/g resin), DCM (2 × 10 ml/g resin), and DMF (2 × 10 ml/g resin), followed by coupling of linker **23** (2 eq., Supplementary Figure S9) with oxyma (4 eq.) and DIC (4 eq.) corresponding to **GP-6**.

#### 2.5.11 Synthesis of Final SMDCs (GP-9)

The corresponding conjugable RGD or RAD mimetic (2.2–2.3 eq.) was dissolved in a cleavage cocktail of TFA/MPW/TIS (1,400 μl, 95:2.5:2.5) and stirred overnight at room temperature. Afterward, the solvent was co-evaporated with toluene and dried in vacuum. The resulting residue was combined with linker-MMAE conjugate **13** (1 eq.) and sodium ascorbate (4.6-4.7 eq.) and dissolved in DMF (1,500 µl) and MPW (200 µl). This solution was degassed by several freeze–pump–thraw cycles and frozen in the end. Under inert conditions (Ar-atmosphere), CuSO_4_∙5H_2_O (2.1–2.6 eq.) was added to the frozen degassed reaction mixture, followed by evacuation of the reaction vessel. The reaction mixture was allowed to warm up to room temperature and was stirred overnight, while the reaction progress was monitored by LCMS. When the consumption of linker-MMAE **13** was complete, the solution was frozen again, and Pd(PPh_3_)_4_ (0.4–0.5 eq.) and morpholine (4 eq.) were transferred into the reaction tube. The reaction was melted at room temperature and stirred for 2 h. After complete allyl deprotection, the reaction mixture was centrifuged and immediately purified by preparative RP-HPLC. The desired compound was obtained as a colorless solid.

#### 2.5.12 Synthesis of Methyl 4-(2-(2-(2-azidoethoxy) ethoxy)ethoxy)benzoate (20)

Chloride **19** (Supplementary Figure S8, 0.753 g, 2.5 mol, 1 eq.) and NaN_3_ (0.447 g, 6.9 mmol, 2.8 eq.) were dissolved in water (25 ml) and DMF (20 ml). The reaction mixture was stirred and heated overnight to 80°C and 1 day at room temperature. Afterward, the mixture was diluted with water and extracted with DCM (3×). The combined organic layers were washed with water (1×) and sat. NaCl (aq., 1×) and dried with MgSO_4_, followed by removal of the solvent under reduced pressure. The product was dried in vacuum to obtain **20** (Supplementary Figure S8, 0.765 g, 2.5 mmol, 99%) as a light-yellow viscous liquid.

#### 2.5.13 Synthesis of 5-Hexynoyl-Glu(All)-Val-Ala-PABA-PNPC (12)

The benzyl alcohol **25** (Supplementary Figure S15, 186.7 mg, 335.4 µmol, 1 eq.) and bis(4-nitrophenyl) carbonate (256.7 mg, 843.9 µmol, 2.5 eq.) were dissolved in dry DMF (6 ml), followed by addition of DIPEA (114.1 µl, 670.9 µmol, 2 eq.). The reaction mixture was stirred for 3 h, and the reaction progress was monitored using LCMS. After full conversion of the starting material, the reaction solution was added to a water/ACN/TFA solution (5:1 + 0,5 % TFA) and immediately frozen and freeze-dried. The resulting solid was purified by column chromatography [DCM—> DCM/MeOH/TFA (95/4.9/0.1)] to give the activated linker **12** (194.4 mg, 269.3 µmol, 80%) as a solid.

#### 2.5.14 Synthesis of 5-Hexynoyl-Glu(All)-Val-Ala-PABA-MMAE (13)

A solution of activated linker **12** (59.9 mg, 83.0 µmol, 1.1 eq.) and HOBt (1.3 mg, 8.42 µmol, 0.1 eq.) in dry DMF (400 µl) was added to a solution of MMAE (54.01 mg, 75.23 µmol, 1 eq.) in dry DMF (400 µl), followed by addition of pyridine (200 µl). The reaction mixture was stirred at room temperature till full conversion of MMAE was observed by LCMS. Afterward, the reaction solution was diluted with MPW and freeze-dried. The crude product was then purified by column chromatography [DCM->DCM/MeOH (90:10, v:v)] to give the linker-MMAE product **13** (94.6 mg, 72.7 µmol, 97%) as a colorless solid.

### 2.6 Biological Analysis and Methods

#### 2.6.1 ELISA-Like Assay

An ELISA-like assay using the isolated extracellular domain of integrins α_V_β_3_ and α_5_β_1_ was performed in flat-bottom 96-well immuno plates (Brand) to determine the activities of the synthesized compounds. All wells were coated overnight with the native integrin ligand vitronectin or fibronectin (1) (Table 1) in a carbonate buffer (150 µl/well), followed by washing of each well with the PBS-T buffer (3 × 200 µl/well) (Table 1) and blocking for 1 h with the TS-B buffer (150 µl/well) at RT. A dilution series was prepared using the internal standard (Cilengitide, 1:5 dilution) and the compounds (1:5 or 1:10 dilution) in the TS-B buffer. The protein-coated assay plate was washed with the PBS-T buffer (3 × 200 µl/well), and 50 µl of the dilution series was transferred to the assay plate wells B–G. The TS-B buffer was filled into row A (100 µl/well) as the negative control and row H (50 µl/well) as the positive control. Afterward, the corresponding human integrin (2, 50 µl/well) (Table 1) in the TS-B buffer was added to row B–H and incubated for 1 h at RT. After washing the assay plate with the PBS-T buffer (3 × 200 µl/well), the primary antibody (3, 100 µl/well) (Table 1) was transferred to each well and incubated for 1 h at RT. Then the plate was washed with the PBS-T buffer (3 × 200 µl/well), treated with the secondary antibody (4, 100 µl/well) (Table 1), and incubated for 1 h at RT. The plate was washed with the PBS-T buffer (3 × 200 µl/well), and SeramunBlau^®^ fast2 (Seramun Diagnostics GmbH, 50 µl/well) was added to each well. Staining was stopped with 3 M aq. H_2_SO_4_ (50 µl/well) when the rows of the internal standard (cilengitide) showed a blue color gradient from well A to H (α_V_β_3_: 40 s; α_5_β_1_: 1.5 min). The absorbance was measured with a plate reader at 450 nm and corrected by subtraction of the absorbance at 620 nm. Afterward, the resulting values were plotted and analyzed using OriginPro^®^ 2020b where the inflection point of a DoseResp fit describes the IC_50_ value. All compounds were tested in duplicates or triplicates for both integrins.

**TABLE 1:** Proteins and buffers applied in the ELISA-like assay.

Condition		Composition
α_V_β_3_	(1)	1.0 μg/ml human vitronectin
	(2)	2.0 μg/ml human α_V_β_3_ integrin
	(3)	2.0 μg/ml mouse anti-human CD51/CD61
	(4)	1.0 μg/ml anti-mouse IgG-POD goat
α_5_β_1_	(1)	0.5 μg/ml human fibronectin
	(2)	2.0 μg/ml human α_5_β_1_ integrin
	(3)	1.0 μg/ml mouse anti-human CD51/CD61
	(4)	2.0 μg/ml anti-mouse IgG-POD goat
Buffer	Carbonate	15 mM Na_2_CO_3_, 35 mM NaHCO_3_, pH 9.6
	PBS-T	137 mM NaCl, 2.7 mM KCl, 10 mM Na_2_HPO_4_,2 mM KH_2_PO_4_, 0.01% Tween 20
	TS-B	20 mM Tris–HCl, 150 mM NaCl, 1 mM CaCl_2_, 1 mM MgCl_2_, 1 mM MnCl_2_, pH 7.5, 1% BSA

#### 2.6.2 Flow Cytometry

WM115 and M21-L cells were seeded in 12-well plates or cell culture flasks and incubated at 37°C for one to 2 days. The cells were detached with Accutase solution (Pan Biotech), washed with the medium, and resuspended in the PBS buffer (137 mM NaCl, 2.7 mM KCl, 10 mM Na_2_HPO_4_, 2 mM KH_2_PO_4_, 300 µl). Then the primary antibody (1) (Supplementary Table S2) was added, followed by incubation for 15 min on ice. Subsequently, cells were centrifuged (10 min, 1800 rpm/350 g) and washed with PBS (800 μl, 10 min 1,800 rpm/350 g). After resuspension in PBS (300 µl), the secondary antibody (2) (Supplementary Table S2) was added, and the cells were incubated for 15 min on ice. Finally, the cells were centrifuged, washed, and resuspended as described and measured with an S3e Cell Sorter (BioRad) by excitation at 488 and 568 nm. For each sample, 30,000 events were measured. As controls, pure cells and cells treated only with the secondary antibody (2) (Supplementary Table S2) were measured. Results are shown in Supplementary Figure S6.

#### 2.6.3 Cell Adhesion Assay

WM115 cells were cultivated and used in the MEM Eagle medium (Pan Biotech P04-08500 with 10% fetal bovine serum, 50 μg/ml gentamycin, and 0.5 mM sodium pyruvate) and M21-L cells in the RPMI medium (Pan Biotech P04-16500 with 10% fetal bovine serum, 1% Pen-Strep). A flat-bottom MaxiSorp Nunc 96-well plate was coated with recombinant human vitronectin (100 µl/well, 1 μg/ml, Peprotech) in the PBS buffer (137 mM NaCl, 2.7 mM KCl, 10 mM Na_2_HPO_4_, 2 mM KH_2_PO_4_, pH 7.4) at 4°C and blocked at the following day by adding a solution of fatty acid free milk powder in PBS buffer (5 w/v %, 100 µl/well) at 4°C. The WM115 and M21-L cells were washed with the PBS buffer, detached with Accutase solution (5 ml, Pan Biotech P10-21100) at 37°C for 5 min, and then diluted with the medium (15 ml). After centrifugation (850 rpm, 6 min), the resulting cell pellet was resuspended with fluorescein diacetate in the medium (fluorescein diacetate 1.5 mg/ml, cell density 5·× 10^5^ cells/ml) and incubated for 30 min at 37°C in the dark. The cells were washed twice with the medium and then resuspended in the medium (cell density 5 × 10^5^ cells/ml). Afterward, a solution of CaCl_2_, MnCl_2_, and MgCl_2_ (each 100 mM) in the PBS buffer (90 µl) was transferred to the cells and incubated for 30 min on ice in the dark. In the meantime, a dilution series of the compounds in the medium (1:3 dilution) was prepared, and 240 µl were transferred to Eppendorf tubes. Pure medium was used as a positive control. Cell suspension (240 µl) was added to each tube of the dilution series and the control, followed by incubation at 37°C for 30 min in the dark. The assay plate was discharged and washed with 200 µl/well medium. Then the cell suspension with different compound concentrations was added to the assay plate (100 µl/well) and incubated for 60 min at 37°C in the dark. Afterward, the assay plate was washed with the medium (3 × 100 µl/well), and finally, the medium (100 µl/well) was added and the fluorescence was measured with a TecanReader (Excitation: 480 nm; Emission: 520 nm). The determined values were plotted and analyzed using OriginPro^®^ 2020b where the inflection point of a DoseResp fit described the IC_50_ value.

#### 2.6.4 Cell Viability Assay

WM115 cells were cultivated in the MEM medium (with 10% fetal bovine serum, 50 μg/ml gentamycin, and 0.5 mM sodium pyruvate) at 37°C and 5.3% CO_2_-humidified air in an incubator. The cells were seeded in a sterile flat-bottom cell culture 96-well plate (Sarstedt) in a density of 10,000 cells/well (100 µl/well) and incubated for 1 day as described. A serial dilution (1:3 dilution) of the compounds and the standard (cryptophycin-52) in the medium was prepared and transferred to the assay plate (100 µl/well), followed by incubation for 3 days as described. Afterward, a solution of resazurin (175 μM, 30 µl/well) was added, followed by incubation for 6 h. Subsequently, the fluorescence was measured with a TecanReader (Excitation: 530 nm; Emission: 588 nm) and plotted and analyzed using OriginPro^®^ 2020b where the inflection point of a DoseResp fit described the IC_50_ value.

## 3 Results and Discussion

Among the previously described small-molecule drug conjugates (SMDC) based on or inspired by peptides, there are only a few conjugates known for targeting the integrins (Dal Corso et al., 2016; Baiula et al., 2021; Lerchen et al., 2022; Slack et al., 2022), particularly α_V_β_3,_ with non-peptide homing devices. Such RGD mimetics provide additional possibilities of introducing structural elements and are metabolically more stable than peptides. Moreover, the generation of compound arrays is straightforward. We embarked on the development of SMDC using RGD mimetics as homing devices. For selection of promising structures, DAD (dual activity and difference) mapping (Medina-Franco et al., 2011) was used, a methodology to visualize activity/selectivity changes against two different receptors upon partial structural changes in an array of molecules.

### 3.1 Library Design and RGD Mimetic Synthesis

**FIGURE 3:**
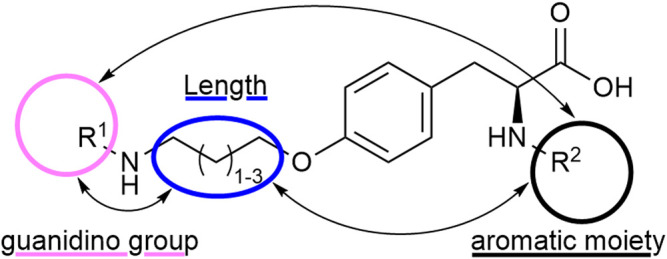
RGD mimetic lead structure with the highlighted variegating moieties.

Tyrosine is a well-established scaffold for non-peptidic RGD mimetics. It lead to a variety of bioactive compounds and RGD mimetics like Tirofiban which is an antiplatelet medication by inhibition of the protein–protein interactions between fibrinogen and integrin α_IIb_β_3_ (Egbertson et al., 1994; Curley et al., 1999) or selective inhibitors for integrin α_V_β_3_/α_5_β_1_ (Heckmann et al., 2007; Heckmann et al., 2008; Heckmann et al., 2009). In contrast to previous approaches, where one or two structural moieties were varied, an approach with variation of three parameters was chosen. Therefore, all possible permutations, depending on the chosen residues, were synthesized. The advantage of this strategy is that every structural change can be observed in all possible structural environments which may lead to a more meaningful SAR study (Figure 3).

A diversifying strategy was employed to generate an array of RGD mimetics using a minimum number of reaction steps by varying the distance (connector unit) between the guanidino-like group (***R*** ^**1**^) and the carboxylic acid, introducing different guanidino analogs (***R*** ^**1**^) and exchanging the *N*-terminal aromatic moiety (***R*** ^**2**^, Figure 3).

**FIGURE 4:**
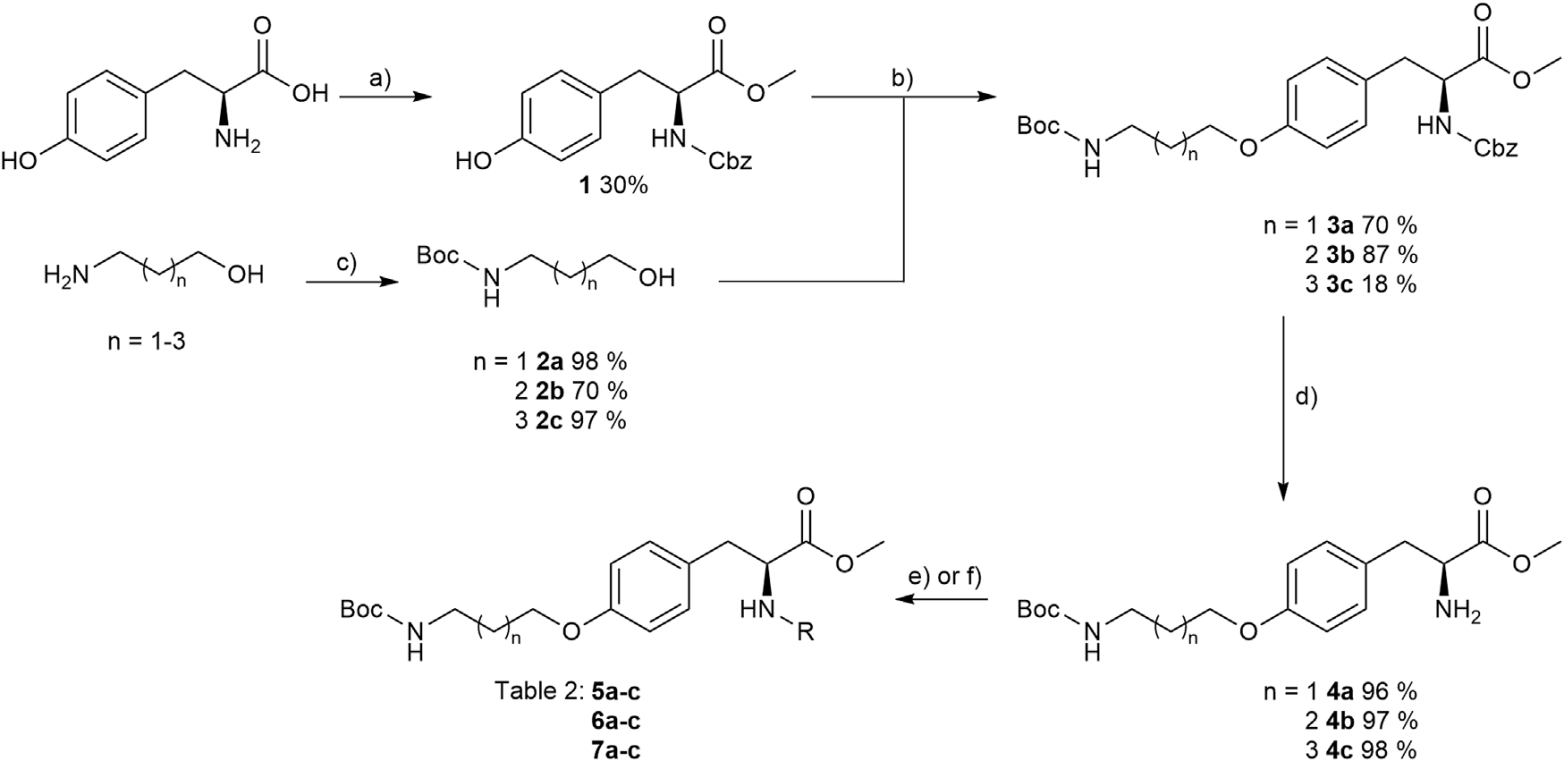
Synthesis of RGD mimetic precursors 5a-c, 6a-c, and 7a-c. Reagents and conditions: a) 1. SOCl_2_, MeOH, and reflux; 2. Cbz-Cl, K_2_CO_3_, acetone, water, 0°C->RT; b) DIAD, PPh_3_, THF, 0°C->RT, o.n.; c) Boc_2_O, NEt_3_, RT, o.n.; d) Pd(OH)_2_/C, H_2_, MeOH/H_2_O 3:1, RT, o.n.; e) benzoic acid, HOBt, EDC, DIPEA, DMF, DCM, RT, o.n.and ; f) benzoyl chloride, DIPEA, DMC, DMF, RT, o.n.

The reaction sequence started with the formation of Cbz-l-tyrosine methyl ester **1**, followed by the first diversification step etherifying the tyrosine phenol by Mitsunobu reaction with three different Boc-protected amino alcohols **2a-c** as connector units. The Cbz-protected amines of the RGD mimetic precursors **3a-c** were deprotected by hydrogenolysis in the presence of Pd (OH)_2_/C. In the next step three different benzoyl substituents were introduced using the corresponding acid chloride or HOBt/EDC mediated amide formation (Figure 4 and Table 2).

**TABLE 2:** Yields and methods for the final reaction step of Figure 4 where e) applies to I and f) to II.

	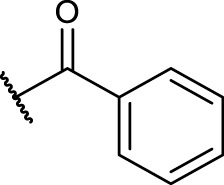			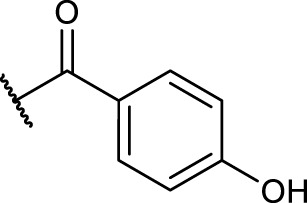			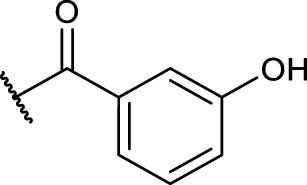		
Length			Yield [%]			Yield [%]			Yield [%]
n = 1	**5a**	II	39	**5b**	II	51	**5c**	I	26
n = 2	**6a**	II	38	**6b**	II	38	**6c**	I	35
n = 3	**7a**	II	26	**7b**	II	24	**7c**	I	33

**FIGURE 5:**
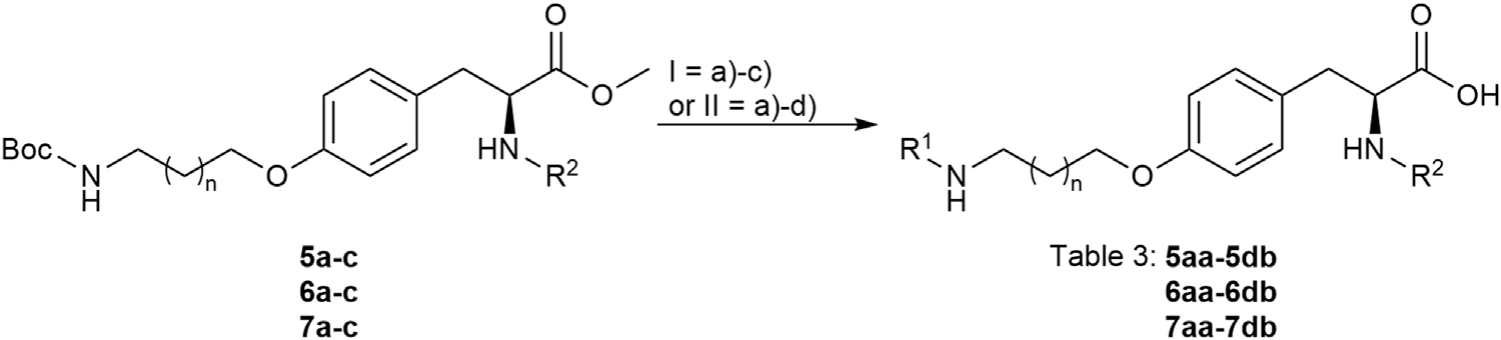
Attachment of the guanidino mimetics giving the final RGD mimetics 5aa-7cd. Reagents and conditions: a) 4 M HCl in dioxane, DCM, RT; b) 2-methylthio-2-imidazoline (DHI) or 2-bromopyrimidine (Pyr/THP), triethylamine, methanol, 80°C, o.n.; c) LiOH, water, methanol, RT; and d) Pd/C, H_2_, AcOH, water, methanol, RT, o.n.

The final reaction sequence comprises three to four steps without purification of intermediate products. After acidolysis of the Boc group the guanidino mimetics were attached. The 2-imidazoline-2-yl moiety (DHI) was introduced using 2-methylthio-2-imidazoline, while the pyrimidin-2-yl residue (Pyr) was attached using 2-bromopyrimidine. The methyl ester was saponified with an excess of LiOH in water/methanol (3:1, *v:v*). The tetrahydropyrimidin-2-yl derivative (THP) as guanidino analog was obtained by catalytic hydrogenation of the pyimidin-2-yl derivatives in the presence of AcOH to avoid the complexation of Pd by the guanidine-like groups (Figure 5 and Table 3).

**TABLE 3:** Yields for the reaction sequence in Figure 5 and Figure 6.

	*R* ^2^	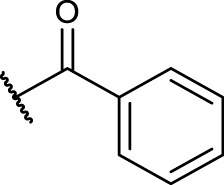			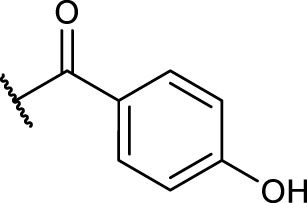			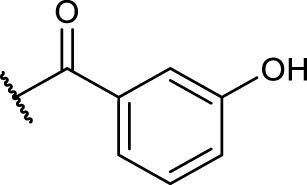			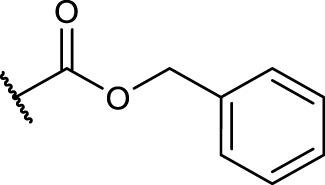		
*R* ^1^	Length			Yield [%]			Yield [%]			Yield [%]			Yield [%]
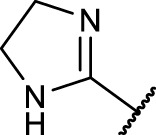	*n* = 1	**5aa**	I	60	**5ba**	I	62	**5ca**	I	43	**5da**	I	68
	*n* = 2	**6aa**	I	21	**6ba**	I	49	**6ca**	I	45	**6da**	I	61
	*n* = 3	**7aa**	I	86	**7ba**	I	73	**7ca**	I	55	**7da**	I	62
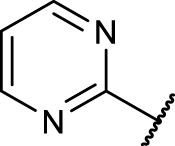	*n* = 1	**5ab**	I	28	**5bb**	I	30	**5cb**	I	42	**5db**	I	90
	*n* = 2	**6ab**	I	33	**6bb**	I	78	**6cb**	I	39	**6db**	I	70
	*n* = 3	**7ab**	I	37	**7bb**	I	41	**7cb**	I	10	**7db**	I	38
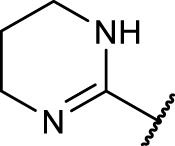	*n* = 1	**5ac**	II	9	**5bc**	II	18	**5cc**	II	61	**5dc**	III	27
	*n* = 2	**6ac**	II	14	**6bc**	II	79	**6cc**	II	47	**6dc**	III	28
	*n* = 3	**7ac**	II	15	**7bc**	II	10	**7cc**	II	43	**7dc**	III	12

**FIGURE 6:**
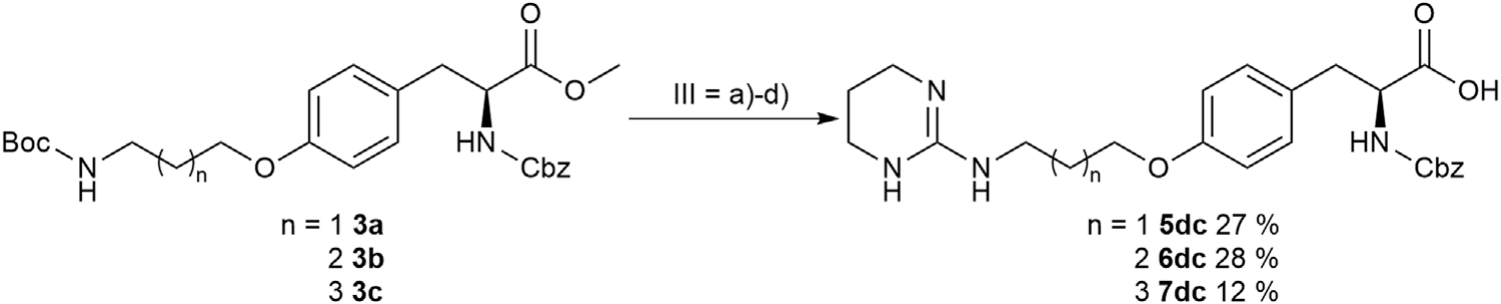
Synthesis of Cbz containing RGD mimetics 5dc, 6dc and 7dc. Reagents and conditions: a) 4 M HCl in dioxane, DCM, RT; b) 2-bromopyrimidine (Pyr/THP), triethylamine, methanol, 80°C, o.n.; c) LiOH, water, methanol, RT; and d) 2-bromopyrimidine, Pd/C, H_2_, AcOH, HBr, water, methanol, RT, o.n.

In order to obtain the THP derivatives **5dc**, **6dc**, and **7dc** containing a Cbz group, a modified procedure for the pyrimidine reduction without cleaving Cbz was required (Figure 6). Interestingly, under reduction conditions II (Figure 5 and Table 3), the expected Cbz cleavage was slow and even in one case the THP derivative could be isolated. Closer investigation of the reaction and improvement of conditions II (Figure 5 and Table 3) resulted in a method for the selective reduction of the pyrimidine ring in the presence of the Cbz group. Noteworthy, 2-bromopyrimidine poisons the Pd catalyst and leads to a selective reduction of the pyrimidine moiety without cleaving the reduction labile Cbz group, while addition of HBr or HCl suppresses side reactions (Figure 6).

### 3.2 Competitive Integrin Binding Assay

The affinities (Table 4) of the RGD mimetics (Table 3) toward the integrins α_V_β_3_ and α_5_β_1_ were determined in a competitive enzyme-linked immunosorbent assay (ELISA) using recombinant human integrins with the native ligands vitronectin and fibronectin as described in the literature (Mas-Moruno et al., 2016b).

**TABLE 4:** Results of the ELISA-like assay with the isolated extracellular domains of integrin α_V_β_3_ and α_5_β_1_. Cilengitide (IC_50_ α_V_β_3_: 0.54 nM; α_5_β_1_: 15.4 nM) was used as reference for all assay plates.

**ID**	***n* =**	***R*** ^**1**^	***R*** ^**2**^	Integrin α_V_β_3_	Integrin α_5_β_1_
				**IC**_**50**_ **[nM]**	**IC**_**50**_ **[nM]**
**5aa**	1	DHI	Benzoic acid	38.5 ± 24.3	25.2 ± 7.35
**5ba**	1	DHI	4-OH benzoic acid	38.8 ± 19.2	43.4 ± 24.4
**5ca**	1	DHI	3-OH benzoic acid	9.29 ± 5.77	30.5 ± 20.7
**5da**	1	DHI	Cbz	1.32 ± 0.11	162 ± 36.0
**5ab**	1	Pyr	Benzoic acid	1,311 ± 4.51	>10,000
**5bb**	1	Pyr	4-OH benzoic acid	6,530 ± 454	>10,000
**5cb**	1	Pyr	3-OH benzoic acid	7,701 ± 416	>10,000
**5db**	1	Pyr	Cbz	237 ± 69.7	>10,000
**5ac**	1	THP	Benzoic acid	18.1 ± 3.68	17.4 ± 3.34
**5bc**	1	THP	4-OH benzoic acid	70.4 ± 0.57	18.5 ± 3.65
**5cc**	1	THP	3-OH benzoic acid	452 ± 120	32.8 ± 11.2
**5dc**	1	THP	Cbz	4.68 ± 4.47	71.6 ± 16.1
**6aa**	2	DHI	Benzoic acid	25.8 ± 6.77	175 ± 73.7
**6ba**	2	DHI	4-OH benzoic acid	1896 ± 175	35.2 ± 18.4
**6ca**	2	DHI	3-OH benzoic acid	1.01 ± 0.56	19.9 ± 8.7
**6da**	2	DHI	Cbz	1.20 ± 0.11	901 ± 223
**6ab**	2	Pyr	Benzoic acid	6,166 ± 1779	>10,000
**6bb**	2	Pyr	4-OH benzoic acid	5,289 ± 1,106	>10,000
**6cb**	2	Pyr	3-OH benzoic acid	1850 ± 241	>10,000
**6db**	2	Pyr	Cbz	8,286 ± 4,393	>10,000
**6ac**	2	THP	Benzoic acid	32.8 ± 17.6	17.5 ± 6.76
**6bc**	2	THP	4-OH benzoic acid	38.7 ± 15.5	511 ± 183
**6cc**	2	THP	3-OH benzoic acid	90.4 ± 31.3	107 ± 32.0
**6dc**	2	THP	Cbz	0.57 ± 0.03	745 ± 117
**7aa**	3	DHI	Benzoic acid	38.5 ± 9.02	1,687 ± 748
**7ba**	3	DHI	4-OH benzoic acid	2.01 ± 0.40	1,652 ± 441
**7ca**	3	DHI	3-OH benzoic acid	98.1 ± 74.8	6,077 ± 3,070
**7da**	3	DHI	Cbz	3.76 ± 1.98	7,746 ± 551
**7ab**	3	Pyr	Benzoic acid	8,766 ± 759	>10,000
**7bb**	3	Pyr	4-OH benzoic acid	8,835 ± 1,268	>10,000
**7cb**	3	Pyr	3-OH benzoic acid	2,887 ± 1,519	>10,000
**7db**	3	Pyr	Cbz	2,625 ± 1,114	8,359 ± 2,598
**7ac**	3	THP	Benzoic acid	58.8 ± 14.4	2,773 ± 899
**7bc**	3	THP	4-OH benzoic acid	36.4 ± 14.3	103 ± 54.4
**7cc**	3	THP	3-OH benzoic acid	42.9 ± 9.47	210 ± 44.8
**7dc**	3	THP	Cbz	2.93 ± 0.12	>10,000

Most of the RGD mimetics investigated display higher affinity to integrin α_V_β_3_, with only a few compounds with a linker length of *n* = 1 or 2, a DHI/THP guanidino mimetic and a benzoyl derivative at the Tyr nitrogen preferring integrin α_5_β_1_. Based on the chosen guanidino analogs it was expected that all mimetics should favor the α_V_-subunit by preventing hydrogen bonds to a glutamic acid side chain (Q221) of the α_5_-subunit (Kapp et al., 2016). Furthermore, the Pyr derivatives generally display less affinity toward both integrins. Several RGD mimetics have high affinity to integrin α_V_β_3_ with good selectivity over integrin α_5_β_1_ whereupon in direct comparison (see Supplementary Figure S1) the longer (*n* = 2–3), Cbz substituted and DHI modified compounds as well as some of the hydroxybenzoyl derivatives showing an outstanding selectivity as well as activity (**6da**, **6dc**, **7ba**, **7da**, and **7dc** in Table 4).

### 3.3 DAD Mapping Analysis

The information gain of direct comparison between molecules/activities among each other is limited and the possible predictions are imprecisely for planning further modifications like the right composition of a conjugable RGD mimetic. Homology modeling as a theoretical approach is a method to explain activity changes by docking ligands into a calculated 3D model of a structural unknown protein (Marinelli et al., 2005; Heckmann et al., 2007; Heckmann et al., 2008).

**FIGURE 7:**
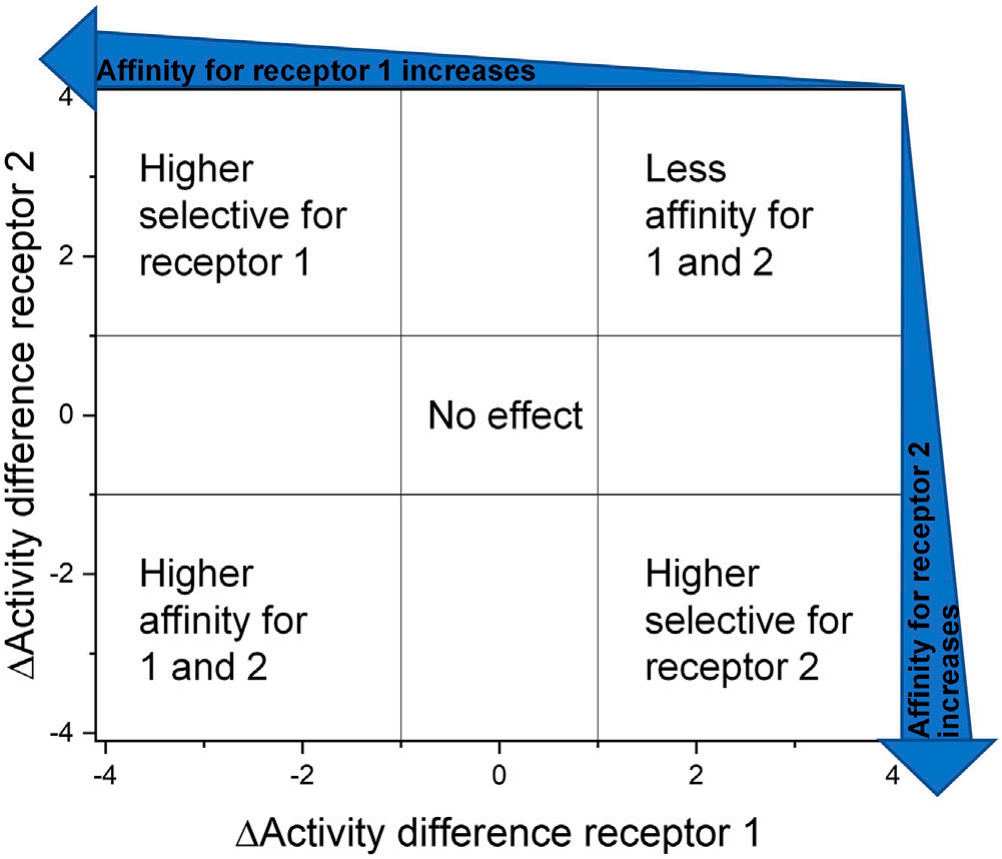
Dual activity and difference map according to Medina-Franco (2012). The areas were selected to assess and present the effect of the structural change they are visible as data points in the corresponding areas.

Another approach to overcome this obstacle is to visualize the impact of structural changes by creating DAD (dual activity and difference) maps. These maps were developed by Jose L. Medina-Franco (Pérez-Villanueva et al., 2011; Medina-Franco, 2012) to point out what consequence a structural change is effecting in dependence of two or more receptors/targets (Medina-Franco et al., 2013). Therefore, the affinity/activity difference, in a logarithmic scale, of two compounds for one specific target is presented on the *X*-axis and for the second target on the *Y*-axis. The amount of deflection from the center describes the magnitude of the affinity and selectivity change that is evoked through this variation as well as the direction of deflection shows the nature of the effect (Figure 7).

**FIGURE 8:**
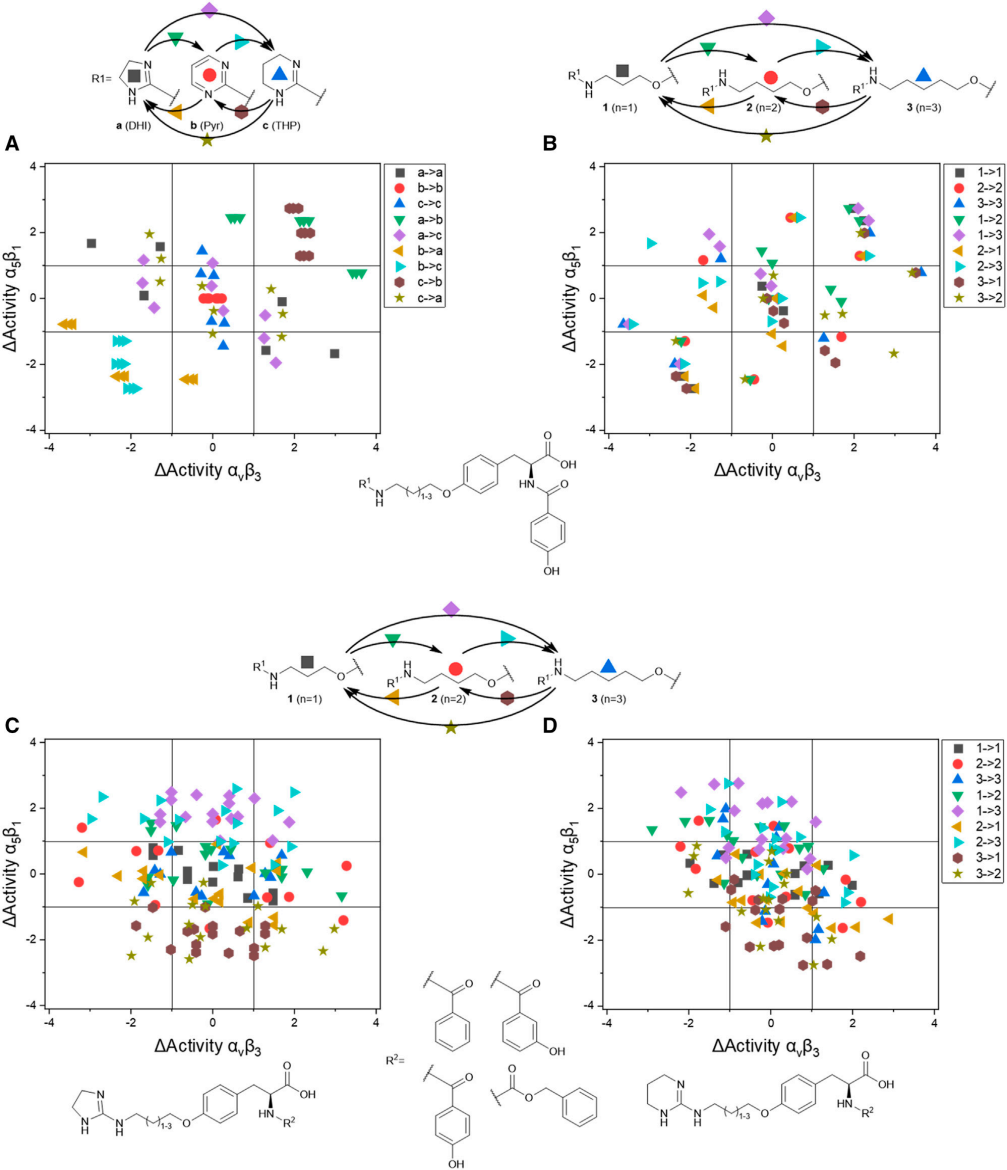
Selected DAD maps of the RGD mimetic array (Table 4), the completing maps are shown in the supplementary material (Supplementary Figures S2–S5). **(A–B)** Influence of variations of the guanidino mimetic **(A)** and linker length **(B)** with the underlying structure shown below the panels. C relates to the guanidino mimetic DHI, while the common element in **(D)** is the guanidino mimetic THP. In both C and D, the influence of the linker length is displayed, and the related lead structure is shown below the panels. The structural change in the aromatic moiety (*R* ^2^) is not highlighted in **(C–D)**. As displayed in Figure 7, signals in the upper-left (high affinity and selectivity for integrin α_V_β_3_) and the lower-right (high affinity and selectivity for integrin α_5_β_1_) corner indicate structural changes which lead to higher affinity and selectivity.

The IC_50_ affinity values of the RGD mimetics toward the integrins α_V_β_3_ and α_5_β_1_ (Table 4) were used to create DAD maps for each parameter (length, guanidino mimetic, and aromatic moiety). One parameter was fixed, and a structural change in the second parameter is marked in color (Figure 8). The changes in the third parameter were disregarded. The common logarithm of its affinity (IC_50_) toward an integrin $\left(\alpha_{X}\beta_{Y}\right)$ was calculated for each compound **Mx** $\left(pIC_{50,\;Mx,\mathrm{int}egrin\;\alpha_{X}\beta_{Y}}\right)$ . Then each value was pairwise compared to all other values using Eq. 1, as follows:

$$\Delta pIC_{50,\;M1\to M2,\;\mathrm{int}egrin\;\alpha_{X}\beta_{Y}}=pIC_{50,\;M1,\;\mathrm{int}egrin\;\alpha_{X}\beta_{Y}}-pIC_{50,M2,\;\mathrm{int}egrin\;\alpha_{X}\beta_{Y}},$$ (1)

where M1→M2 indicates the structural change from molecule M1 to another molecule M2. The resulting $\Delta pIC_{50,\;M1\to M2,\;\mathrm{int}egrin\;\alpha_{X}\beta_{Y}}$ may have positive or negative values depending on the affinity gain or loss upon the structural change. A value of $\Delta pIC_{50,\;M1\to M2,\;\mathrm{int}egrin\;\alpha_{X}\beta_{Y}}=0$ represents no change in affinity based on the structural change for the specific integrin (Medina-Franco et al., 2013).

The DAD maps in Figure 8 present the affinity change for integrin α_V_β_3_ on the *X*-axis and for integrin α_5_β_1_ on the *Y*-axis depending on different selected structural changes. Panel **A** (Figure 8) confirms the assumption that a change to a pyrimidinyl group as ***R*** ^**1**^ in each structural environment leads to generally lower affinities and selectivity.

Figure 8 also indicates that an increasing selectivity is induced by replacing THP by DHI as guanidine mimetic (**A**) together with increasing affinity/selectivity by longer connector units (**B**). However, this elongation effect in case of the 4-hydroxybenzoyl derivatives is also influenced by other parameters because of the broad distribution (Figure 8B). Structural changes in presence of other aromatic residues in comparison to 4-hydroxybenzoyl do not lead to significant improvements in selectivity and affinity by changing connector length or the guanidino group (Supplementary Figures S4,S5). Noteworthy, the distribution of 4-hydroxybenzoyl derivatives upon exchanging the guanidino mimetic from DHI or THP to Pyr is narrower than the distribution upon exchanging the guanidino mimetic in presence of the other aromatic moieties (Supplementary Figure S4). This leads to the hypothesis that the influence of introducing a guanidine analog, with a known effect, can be predicted more accurately in presence of this aromatic moiety. Nevertheless, the influence of the aromatic moiety is limited and effects the broad distribution in panels **C** and **D** (Figure 8). A more pronounced influence is shown by variation of the connector length between both pharmacophoric groups with either DHI or THP as guanidino mimetics, independently from the aromatic acyl group (panels **C** and **D**, Figure 8). This effect is more independent of other structural changes in presence of DHI (**C**) as guanidino group than with THP (**D**) whereupon THP leads to greater activity changes (Figure 8). The direct comparison in length changes between DHI (**C**) and THP (**D**) substituted derivatives reveals the selectivity dependency of the THP group by accumulating the changes on the descending diagonal (from left-upper to right-down corner) at which a broad distribution is generated (**D**, Figure 8). In contrast to this observation the length changes from *n* = 1 or 2 to *n* = 3 in presence of DHI resulting in a general decreased activity for integrin α_5_β_1_ where at the distribution is more focused (Figure 8C). This leads to the assumption that DHI as guanidino group has a stabilizing effect for predicting biological behavior for structure similar molecules with this moiety.

In summary the DAD mapping analysis of the ELISA results predicts some structural motifs which have great influence on affinity and selectivity for integrin α_V_β_3_: A length of *n* = 2-3 whereupon *n* = 3 should be better, 4-hydroxybenzoyl as aromatic moiety and DHI as guanidino group because of its stabilizing effect.

### 3.4 Synthesis and Biological Evaluation of Conjugable RGD Mimetics

For the implementation of a linear RGD mimetic as homing device for SMDCs it is necessary to incorporate a conjugable function in the RGD mimetic without losing affinity and selectivity for the desired integrin. Based on the DAD mapping analysis DHI as guanidino analog and 4-hydroxybenzoyl was chosen as aromatic moiety because of its biological behavior and simple synthetic modifiability by functionalization with a short azide-containing polyethylene glycol spacer. The conjugation at the para-position of a *N*-terminal aromatic moiety had been investigated for linear mimetics selectively binding integrin α_V_β_3_ and α_5_β_1_ (Rechenmacher et al., 2013), for piperazine based RGD mimetics (Owen et al., 2007; Klim et al., 2012), and for a tricyclic aminopyrimidine benzoic acid based RGD mimetic (Alsibai et al., 2014). In these cases the decrease in selectivity and activity was only minor.

**FIGURE 9:**
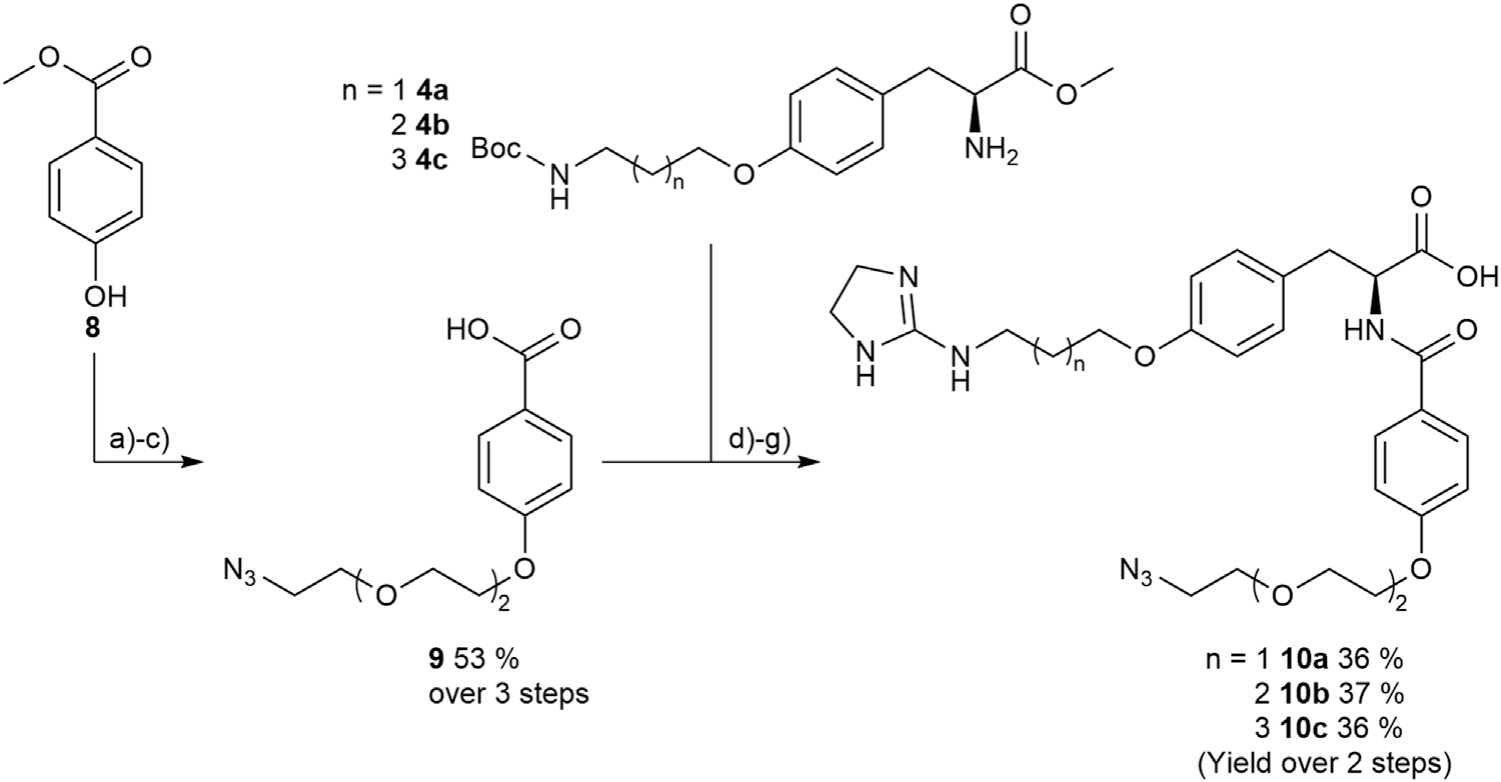
Synthesis of RGD mimetics 10a-c for conjugation. Reagents and conditions: a) 2-(2-(2-chloroethoxy) ethoxy) ethan-1-ol, DIAD, PPh_3_, THF, 0°C->RT, o.n.; b) NaN_3_, water, DMF, 80°C->RT; c) LiOH, water, methanol, THF, o.n. RT; d) HOBt, EDC, DIPEA, DMF, DCM, o.n., RT; e) 4 M HCl in dioxane, DCM, RT; f) 2-methylthio-2-imidazoline, triethylamine, methanol, 80°C, o.n.; and g) LiOH, water, methanol, RT.

Therefore, protected 4-hydroxybenzoic acid **8** was modified in a Mitsunobu reaction with a chlorinated triethylene glycol derivative, followed by azidation using sodium azide. After ester hydrolysis with an excess of LiOH the free acid was coupled with the amines **4a-4c** upon activation with HOBt and EDC to give the three “clickable” RGD mimetics **10a-c** (Figure 9).

The determined IC_50_ values of compounds **10a-c** (Table 5) validate the predicted influence for the used composition. A higher distance between the carboxylic acid and the guanidino group effects a higher affinity toward integrin α_V_β_3_ and a better selectivity over integrin α_5_β_1_. However, the triethylene glycol linker attachment in this position decreases the affinity compared to the unconjugated RGD mimetics and negatively influences the selectivity in comparison to **7ba** (Table 5). This negative effect triggered by the linker introduction was also observed for an integrin α_5_β_1_ selective linear RGD mimetic where the affinity to integrin α_V_β_3_ was increased 13-fold and, consequently, the selectivity was decreased (Rechenmacher et al., 2013).

**TABLE 5:** Results of the ELISA-like assay for the conjugable RGD mimetics 10a-c (Figure 9).

**ID**	***n* =**	Integrin α_V_β_3_	Integrin α_5_β_1_
		**IC50 [nM]**	**IC50 [nM]**
**10a**	1	278 ± 69.2	40.0 ± 6.50
**10b**	2	129 ± 2.35	404 ± 282
**10c**	3	21.0 ± 5.84	136 ± 27.4
**7ba**	3	2.01 ± 0.40	1,652 ± 441

### 3.5 Synthesis of cRGDfK and cRADfK Peptides

In order to evaluate the potency of the conjugable RGD mimetic **10c** as a homing device, the peptides cRGDfK and cRADfK were chosen as positive and negative controls due to their difference in affinity for the α_V_β_3_ integrin. The linear peptides were synthesized by solid-phase peptide synthesis using the 2-chlorotrityl resin according to the ^t^Bu/Fmoc strategy with the coupling reagents oxyma and DIC. Peptide synthesis started with immobilized Fmoc-Gly, as the linear peptide H-Asp (*t*Bu)-d-Phe-Lys (Alloc)-Arg (Pbf)-Gly-OH would not epimerize during macrocyclization with HATU and HOAt. Noteworthy, no epimerization of the C-terminal Ala in H-Asp (*t*Bu)-d-Phe-Lys (Alloc)-Arg (Pbf)-Ala-OH was observed either. After completion of the *N*-terminal Fmoc protected target peptides on resin, the Alloc group at the lysine side chain was cleaved by Pd catalysis with 1,3-dimethylbarbituric acid (DMBA) as scavenger (Tala et al., 2015). An azide-containing triethylene glycol linker **23** (Supplementary Figure S14) was attached to the lysine side chain on resin using the general coupling protocol **GP-6**. The linker **23** (Supplementary Figure S9) was synthesized starting from 2,2'-[ethane-1,2-diylbis (oxy)] bis (ethan-1-ol) following the literature (Gavrilyuk et al., 2009). Afterward the *N*-terminal Fmoc group was cleaved, the peptide was cleaved from the resin using 1% TFA in DCM, and the resulting linear peptides were cyclized under *pseudo*-high dilution (Malesevic et al., 2004) using syringe pumps with separate syringes for the peptide and coupling reagents (see supplementary material). This strategy minimized the number of purification steps to one final normal-phase column chromatography and is more time efficient then the common liquid-phase linker introduction (Gavrilyuk et al., 2009).

### 3.6 Small-Molecule Drug Conjugate Synthesis

The SMDCs were designed to contain an RGD mimetic as the homing device connected to the antimitotic drug MMAE as the toxic payload across a self-immolative linker. The dipeptide sequence Val-Ala, cleavable by cathepsin B, was combined with the self-immolative spacer *para*-aminobenzyl carbamate (PABC) to give a lysosomally cleavable conjugate as shown previously in other cases (Dal Corso et al., 2016; Borbély et al., 2019a).

An additional glutamic acid was incorporated in the linker to increase the plasma stability (Anami et al., 2018; Poreba, 2020) and 5-hexynoic acid was attached to the *N*-terminal for later conjugation *via* copper-catalyzed azide-alkyne cycloaddition (CuAAC). The linker **12** was synthesized on 2-chlorotrityl resin using the All/Fmoc-strategy and oxyma/DIC as coupling reagents.

The resin was loaded with Fmoc-Val-PABA, obtained from Fmoc-Val and PABA (*para*-aminobenzyl alcohol) using EEDQ-mediated coupling according to the literature (Cheng et al., 2020). The loading was done according to the literature (Barthel et al., 2012) with pyridine as base and gave a loading level of 0.90 mmol/g_resin_. After coupling of the subsequent amino acids and *N*-terminal 5-hexynoic acid, the peptide was cleaved from the resin and precipitated in water. The resulting benzyl alcohol-containing linker was then activated with bis(*para*-nitrophenyl) carbonate and the resulting (*para*-nitrophenyl) carbonate **12** was coupled to MMAE. As a result of the methylation the *N*-terminal secondary amine of MMAE is sterically hindered and, therefore, the (*para*-nitrophenyl) carbonate **12** has to be activated by the addition of a catalytic amount of HOBt (0.1 eq.) to reach a yield of 97 % after purification *via* normal-phase column chromatography.

**FIGURE 10:**
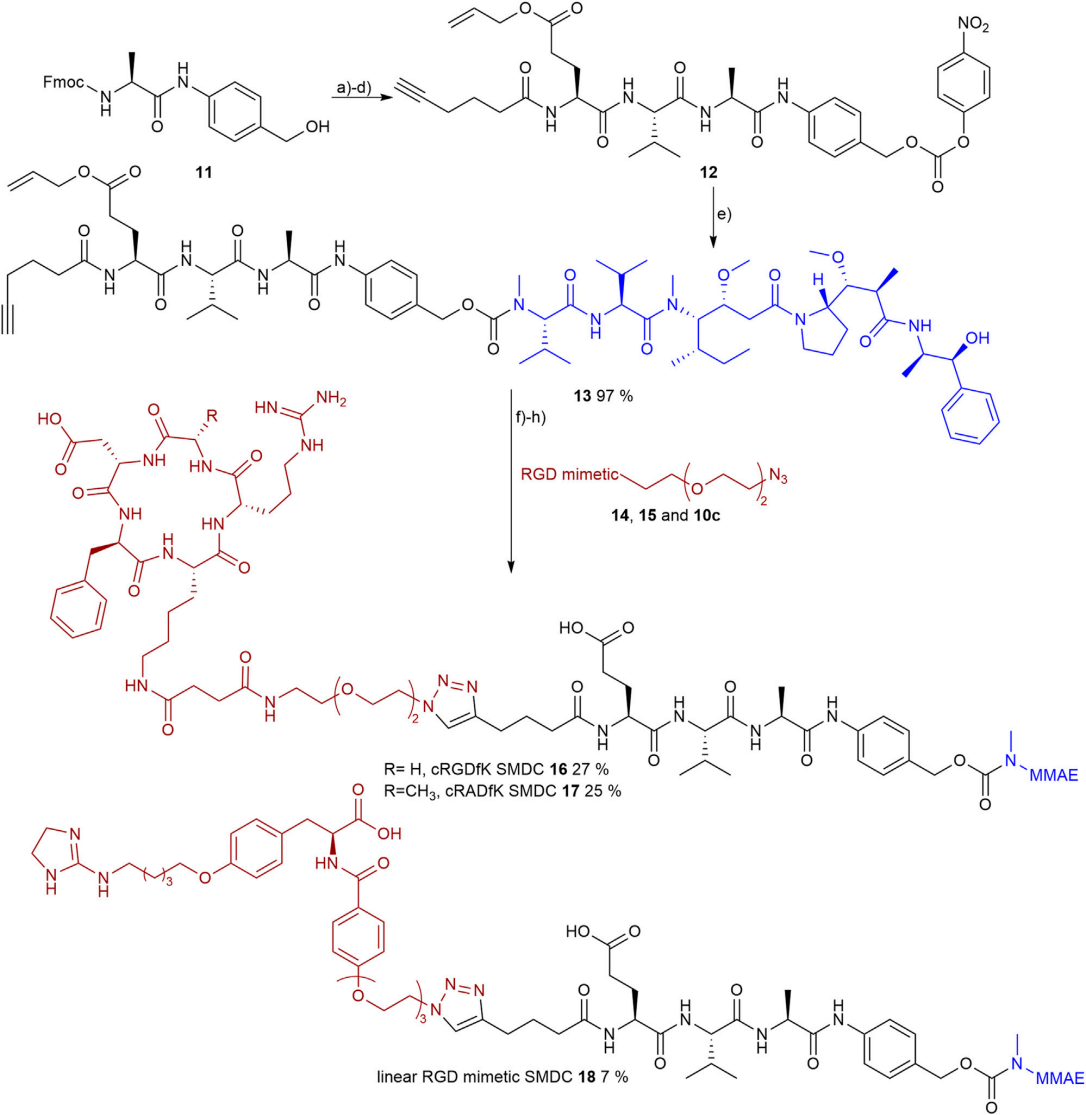
Synthesis of the RGD or RAD containing SMDC 14-15 and 10c as RGD mimetics and MMAE as payloads. Reagents and conditions: a) pyridine, DCM, 2 days, RT; b) SPPS (1. Oxyma, DIC, DMF; 2. 20 % piperidine in DMF); c) TFA, DCM, RT; d) bis(*para*-nitrophenyl) carbonate, DIPEA, DMF, RT; e) MMAE, HOBt, pyridine, DMF, RT; f) TFA, TIPS, MPW, o.n., RT; g) CuSO_4_, Na-ascorbate, DMF, water, o.n., RT; and h) Pd (PPh_3_)_4_, morpholine, DMF, water, RT.

Prior to the final CuAAC the side chain-protecting groups of the reference peptides cRGDfK **14** and cRADfK **15** were cleaved using 95% TFA with scavengers. The azide-containing cyclic peptides **14**, **15** or the RGD mimetic **10c** were attached to the alkyne-modified MMAE-linker construct **13** by CuAAC (Figure 10).

### 3.7 Whole-Cell Evaluation of SMDCs

The RGD mimetic containing SMDC **18** inhibits integrin-dependent cell adhesion, which was shown for WM115 cells presenting the integrin α_V_β_3_. The highly affine α_V_β_3_-selective RGD-cyclopeptide Cilengitide was used as reference (Mas-Moruno et al., 2010). The cRGDfK-containing SMDC **16** served as positive control and the cRADfK-containing SMDC **17** as negative control.

**FIGURE 11:**
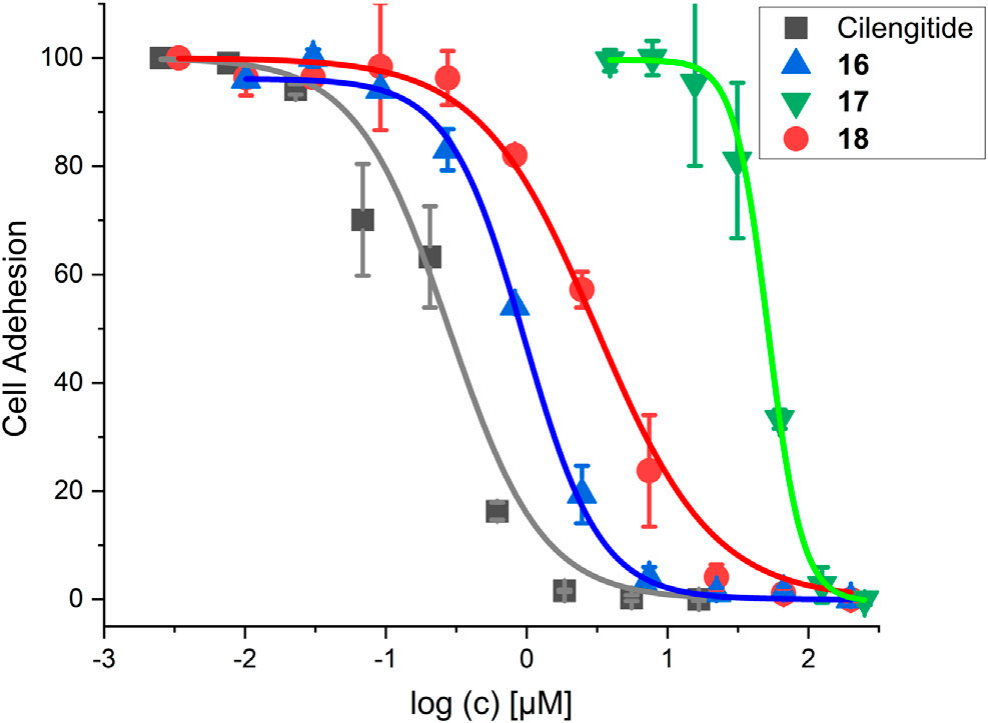
Results for the cell adhesion assay against WM115 cells. For IC_50_ values, see Table 6.

The linear RGD SMDC and the cRGDfK SMDC inhibited adhesion of the α_V_β_3_-positive WM115 cells to vitronectin with IC_50_ values in the low µM range, while no effect was observed for the α_V_β_3_-negative M21-L cell line (Table 6 and Figure 11).

**TABLE 6:** Cell adhesion assay of SMDC 16-18 in comparison to Cilengitide. WM115 cells were used as α_V_β_3_-positive cell line and M21-L as α_V_β_3_-negative cell line.

Compound	WM115 (α_V_β_3_+)	M21-L (α_V_−, α_V_β_3_−)
	IC_50_ [µM]	IC_50_ [µM]
Cilengitide	0.43 ± 0.05	>100 µM
**16**	2.65 ± 0.35	>100 µM
**17**	79.1 ± 1.69	>100 µM
**18**	8.05 ± 0.51	>100 µM

The integrin status of the WM115 cells as well as for the control cell line M21-L was determined by fluorescence-activated cell sorting (FACS) analysis. This proved the occurrence of integrins α_V_β_3_, α_V_β_8_, and α_5_β_1_ on WM115 and the absence on M21-L cells except integrin α_5_β_1_, which is present (Borbély et al., 2019a) (Supplementary Figure S6).

The cRGDfK-SMDC **16** inhibits cell adhesion of the integrin α_V_β_3_-positive WM115 cells to vitronectin nearly as efficiently as Cilengitide, while the cRADfK-SMDC **17** has a significantly lower effect (Figure 11). Noteworthy, the non-peptide RGD mimetic-SMDC **18** has an IC_50_ value comparable to cRGDfK-SMDC **16**. This is in good agreement with ELISA-like assay results for Cilengitide and the unconjugated linear RGD mimetic **10c** (ELISA IC_50_: Cilengitide 0.54 nM, **10c** 21.0 nM, Table 5). This corroborates that the RGD mimetic containing SMDC **18** binds to integrin α_V_β_3_ like the positive control cRGDfK-SMDC **16.**

**FIGURE 12:**
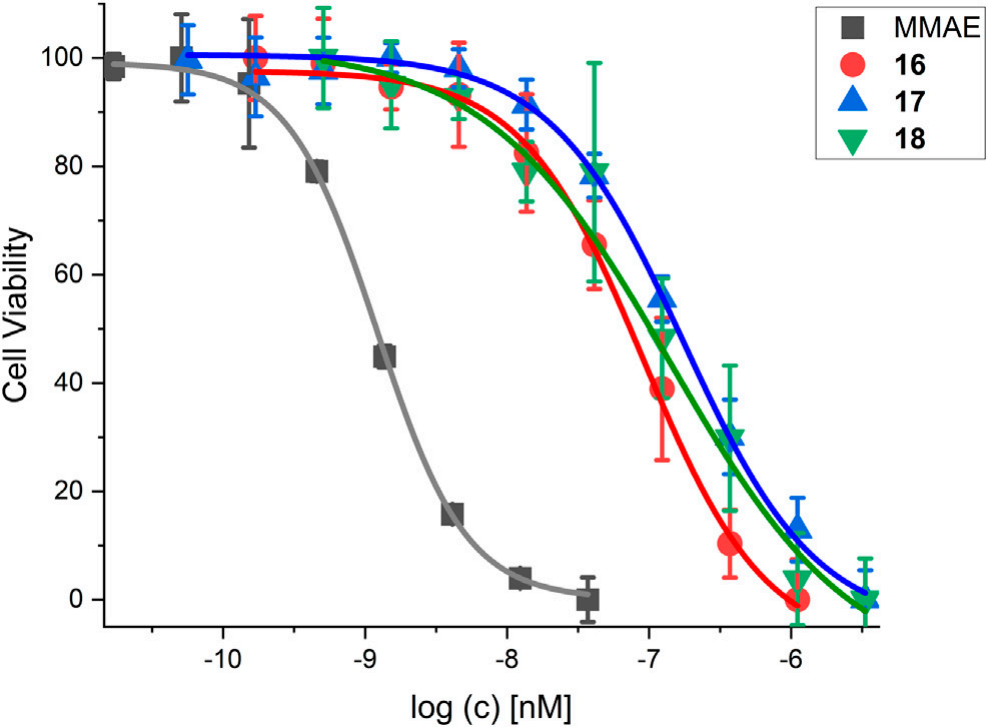
Cell viability assay for SMDC **18** in comparison to control compounds and the free drug MMAE. For IC_50_ values, see Table 7.

The cytotoxicity of SMDC **16-18** was determined in a resazurin based assay with the melanoma cell line WM115 (Table 7 and Figure 12).

**TABLE 7:** Cytotoxicity data with WM115 cells and the calculated targeting index [TI = [IC_50_ (17)]/[IC_50_ (compound)]].

Compound	IC_50_ [nM]	TI (RAD/RGD)
MMAE	1.84 ± 0.26	
**16** (cRGDfK)	91.4 ± 12.3	2.9
**17** (cRADfK)	264 ± 24.6	1.0
**18** (RGD mimetic)	95.0 ± 25.0	2.8

MMAE, a cytotoxic agent with a low nM IC_50_ and used as payload in known ADCs and SMDCs (Bai et al., 1990; Staudacher and Brown, 2017; Akaiwa et al., 2020; Criscitiello et al., 2021; Gao et al., 2021), was used as reference compound in the cell viability assay and as SMDC payload. Both the linear RGD SMDC **18** (IC_50_ = 95.0 ± 25.0 nM) and the cRGDfK SMDC **16** (IC_50_ = 91.4 ± 12.3 nM) are about 50-fold less cytotoxic than free MMAE against the α_V_β_3_-positive WM115 cell line with IC_50_ values in the mid-nM range. In contrast, the cRADfK SMDC **17** is 150-fold less cytotoxic than MMAE. Hence, integrin binding also influences the antiproliferative activity. The ratio IC_50_ (RAD)/IC_50_ (RGD) provides a measure for the selectivity giving a targeting index TI of 2.9 for **16** and 2.8 for **18**. TI values between 1 and 10 have been reported for SMDC (Zanella et al., 2017). Low TI values may also be associated with non-receptor-mediated uptake mechanisms. The size-dependent cellular uptake (Kemker et al., 2019) could be an explanation for this behavior because of the relatively low molecular mass of the conjugates **16-18**. It was also previously reported that a integrin α_V_β_3_-addressing cRGDfK-carboxyfluorescein conjugate was taken up by integrin-positive and integrin-negative cell lines with the assumption of a fluid-phase uptake (Borbély et al., 2019a).

## 4 Conclusion

Starting from an established tyrosine scaffold, an array of 36 small-molecule RGD mimetics was synthesized by varying three parameters (guanidino mimic, linker length, and aromatic acyl moiety). An efficient diversification strategy was used, which also allows further modifications. The affinities of the RGD mimetics toward the integrins α_V_β_3_ and α_5_β_1_ were determined in an ELISA-like assay. The DAD mapping analysis of the IC_50_ values allowed to identify important structural motifs to select a conjugable RGD mimetic (**10c**), consisting of DHI (dihydroimidazole) as guanidino mimetic, a C_5_ connector, and a 4-hydroxybenzoyl-based azide-containing linker for conjugation. The mimetic **10c** was connected by CuAAC to a cathepsin-cleavable linker **13**, where the Val–Ala recognition sequence was linked across a self-immolative PABC (*para*-aminobenzyl carbamate) moiety to MMAE, giving the RGD mimetic-SMDC **18**. Peptide conjugates like the cRGDfK-SMDC **16** as the positive control and the cRADfK-SMDC **17** as the negative control were investigated with respect to integrin binding in cell adhesion assays. The positive control cRGDfK-SMDC **16** and the RGD mimetic-SMDC **18** displayed micromolar IC_50_ values with α_V_β_3_-positive cells, while no influence on cell adhesion was observed for α_V_β_3_-negative cells, which indicates a receptor selectivity for SMDC **16** and **18**. The cell viability assay revealed cytotoxicity in the nanomolar range for SMDC **16** and **18**. Hence, integrin binding also influences the antiproliferative activity giving a targeting index of 2.8. Thus, a bioactive SMDC was obtained based on a linear RGD mimetic retrieved by DAD mapping analysis of a small-molecule array and the resulting structural prediction.

## Data Availability

The original contributions presented in the study are included in the article/Supplementary Material, further inquiries can be directed to the corresponding author.

## References

[B1] Akaiwa M. Dugal-Tessier J. Mendelsohn B. A. (2020). Antibody-Drug Conjugate Payloads; Study of Auristatin Derivatives. Chem. Pharm. Bull. 68, 201–211. 10.1248/cpb.c19-00853 32115527

[B2] Alsibai W. Hahnenkamp A. Eisenblätter M. Riemann B. Schäfers M. Bremer C. (2014). Fluorescent Non-peptidic RGD Mimetics with High Selectivity for αVβ3 vs αIIbβ3 Integrin Receptor: Novel Probes for *In Vivo* Optical Imaging. J. Med. Chem. 57, 9971–9982. 10.1021/jm501197c 25384028

[B67] Anami Y. Yamazaki C. M. Xiong W. Gui X. Zhang N. An Z. (2018). Glutamic Acid-Valine-Citrulline Linkers Ensure Stability And Efficacy Of Antibody-Drug Conjugates In Mice. Nat. Commun. 9, 2512. 10.1038/s41467-018-04982-3 29955061 PMC6023893

[B3] Anselmi M. Borbély A. Figueras E. Michalek C. Kemker I. Gentilucci L. (2020). Linker Hydrophilicity Modulates the Anticancer Activity of RGD-Cryptophycin Conjugates. Chem. Eur. J. 27, 1015–1022. 10.1002/chem.202003471 32955139 PMC7839693

[B4] Aumailley M. Gurrath M. Müller G. Calvete J. Timpl R. Kessler H. (1991). Arg-Gly-Asp Constrained within Cyclic Pentapoptides Strong and Selective Inhibitors of Cell Adhesion to Vitronectin and Laminin Fragment P1. FEBS Lett. 291, 50–54. 10.1016/0014-5793(91)81101-D 1718779

[B5] Bachmann M. Kukkurainen S. Hytönen V. P. Wehrle-Haller B. (2019). Cell Adhesion by Integrins. Physiol. Rev. 99, 1655–1699. 10.1152/physrev.00036.2018 31313981

[B6] Bai R. Petit G. R. Hamel E. (1990). Dolastatin 10, a Powerful Cytostatic Peptide Derived from a marine Animal. Biochem. Pharmacol. 39, 1941–1949. 10.1016/0006-2952(90)90613-P 2353935

[B7] Baiula M. Cirillo M. Martelli G. Giraldi V. Gasparini E. Anelli A. C. (2021). Selective Integrin Ligands Promote Cell Internalization of the Antineoplastic Agent Fluorouracil. ACS Pharmacol. Transl. Sci. 4, 1528–1542. 10.1021/acsptsci.1c00094 34661072 PMC8506610

[B8] Barczyk M. Carracedo S. Gullberg D. (2010). Integrins. Cell Tissue Res 339, 269–280. 10.1007/s00441-009-0834-6 19693543 PMC2784866

[B9] Bargh J. D. Isidro-Llobet A. Parker J. S. Spring D. R. (2019). Cleavable Linkers in Antibody-Drug Conjugates. Chem. Soc. Rev. 48, 4361–4374. 10.1039/C8CS00676H 31294429

[B10] Barthel B. L. Rudnicki D. L. Kirby T. P. Colvin S. M. Burkhart D. J. Koch T. H. (2012). Synthesis and Biological Characterization of Protease-Activated Prodrugs of Doxazolidine. J. Med. Chem. 55, 6595–6607. 10.1021/jm300714p 22742660 PMC3433255

[B11] Battistini L. Bugatti K. Sartori A. Curti C. Zanardi F. (2021). RGD Peptide‐Drug Conjugates as Effective Dual Targeting Platforms: Recent Advances. Eur. J. Org. Chem. 2021, 2506–2528. 10.1002/ejoc.202100240

[B12] Bochen A. Marelli U. K. Otto E. Pallarola D. Mas-Moruno C. Di Leva F. S. (2013). Biselectivity of isoDGR Peptides for Fibronectin Binding Integrin Subtypes α5β1 and αvβ6: Conformational Control through Flanking Amino Acids. J. Med. Chem. 56, 1509–1519. 10.1021/jm301221x 23362923

[B13] Borbély A. Figueras E. Martins A. Bodero L. Raposo Moreira Dias A. López Rivas P. (2019a). Conjugates of Cryptophycin and RGD or Iso DGR Peptidomimetics for Targeted Drug Delivery. ChemistryOpen 8, 737–742. 10.1002/open.201900110 31275795 PMC6587324

[B14] Borbély A. Figueras E. Martins A. Esposito S. Auciello G. Monteagudo E. (2019b). Synthesis and Biological Evaluation of RGD-Cryptophycin Conjugates for Targeted Drug Delivery. Pharmaceutics 11, 151. 10.3390/pharmaceutics11040151 PMC6523311 30939768

[B15] Casi G. Neri D. (2015). Antibody-Drug Conjugates and Small Molecule-Drug Conjugates: Opportunities and Challenges for the Development of Selective Anticancer Cytotoxic Agents. J. Med. Chem. 58, 8751–8761. 10.1021/acs.jmedchem.5b00457 26079148

[B16] Chari R. V. J. Miller M. L. Widdison W. C. (2014). Antibody-drug Conjugates: an Emerging Concept in Cancer Therapy. Angew. Chem. Int. Ed. 53, 3796–3827. 10.1002/anie.201307628 24677743

[B68] Cheng P. Miao Q. Huang J. Li J. Pu K. (2020). Multiplex Optical Urinalysis for Early Detection of Drug-Induced Kidney Injury. Anal. Chem. 92, 6166–6172. 10.1021/acs.analchem.0c00989 32241110

[B17] Cooper J. Giancotti F. G. (2019). Integrin Signaling in Cancer: Mechanotransduction, Stemness, Epithelial Plasticity, and Therapeutic Resistance. Cancer Cell 35, 347–367. 10.1016/j.ccell.2019.01.007 30889378 PMC6684107

[B18] Corbett J. W. Graciani N. R. Mousa S. A. DeGrado W. F. (1997). Solid-phase Synthesis of a Selective αvβ3 Integrin Antagonist Library. Bioorg. Med. Chem. Lett. 7, 1371–1376. 10.1016/S0960-894X(97)00200-X

[B19] Criscitiello C. Morganti S. Curigliano G. (2021). Antibody-drug Conjugates in Solid Tumors: a Look into Novel Targets. J. Hematol. Oncol. 14, 20. 10.1186/s13045-021-01035-z 33509252 PMC7844898

[B69] Curley G. P. Blum H. Humphries M. J. (1999). Integrin Antagonists. Cell Mol. Life Sci. 56, 427–441. 10.1007/s000180050443 11212296 PMC11146902

[B20] Dal Corso A. Pignataro L. Belvisi L. Gennari C. (2016). αvβ3 Integrin-Targeted Peptide/Peptidomimetic-Drug Conjugates: In-Depth Analysis of the Linker Technology. Curr. Top. Med. Chem. 16, 314–329. 10.2174/1568026615666150701114343 26126915

[B21] De Franceschi N. Hamidi H. Alanko J. Sahgal P. Ivaska J. (2015). Integrin Traffic - the Update. J. Cell Sci. 128, 839–852. 10.1242/jcs.161653 25663697 PMC4342575

[B22] Deneka A. Y. Boumber Y. Beck T. Golemis E. A. (2019). Tumor-Targeted Drug Conjugates as an Emerging Novel Therapeutic Approach in Small Cell Lung Cancer (SCLC). Cancers 11, 1297. 10.3390/cancers11091297 PMC6769513 31484422

[B23] Deonarain M. Yahioglu G. Stamati I. Pomowski A. Clarke J. Edwards B. (2018). Small-Format Drug Conjugates: A Viable Alternative to ADCs for Solid Tumours? Antibodies 7, 16. 10.3390/antib7020016 PMC6698822 31544868

[B70] Egbertson M. S. Chang C. T. Duggan M. E. Gould R. J. Halczenko W. Hartman G. D. (1994). Non-Peptide Fibrinogen Receptor Antagonists. 2. Optimization of a Tyrosine Template as a Mimic For Arg-Gly-Asp. J. Med. Chem. 37, 2537–2551. 10.1021/jm00042a007 8057299

[B24] Eliceiri B. P. Cheresh D. A. (1999). The Role of αv Integrins during Angiogenesis: Insights into Potential Mechanisms of Action and Clinical Development. J. Clin. Invest. 103, 1227–1230. 10.1172/JCI6869 10225964 PMC408360

[B25] Engel J. Emons G. Pinski J. Schally A. V. (2012). AEZS-108: a Targeted Cytotoxic Analog of LHRH for the Treatment of Cancers Positive for LHRH Receptors. Expert Opin. Investig. Drugs 21, 891–899. 10.1517/13543784.2012.685128 22577891

[B26] Frank A. O. Otto E. Mas-Moruno C. Schiller H. B. Marinelli L. Cosconati S. (2010). Conformational Control of Integrin-Subtype Selectivity in isoDGR Peptide Motifs: a Biological Switch. Angew. Chem. Int. Edition 49, 9278–9281. 10.1002/anie.201004363 20957712

[B27] Gao G. Wang Y. Hua H. Li D. Tang C. (2021). Marine Antitumor Peptide Dolastatin 10: Biological Activity, Structural Modification and Synthetic Chemistry. Mar. Drugs 19, 363. 10.3390/md19070363 34202685 PMC8303260

[B28] Gavrilyuk J. I. Wuellner U. Barbas C. F. (2009). β-Lactam-based Approach for the Chemical Programming of Aldolase Antibody 38C2. Bioorg. Med. Chem. Lett. 19, 1421–1424. 10.1016/j.bmcl.2009.01.028 19181522 PMC2688461

[B29] Gerber H.-P. Senter P. D. Grewal I. S. (2009). Antibody Drug-Conjugates Targeting the Tumor Vasculature. MAbs 1, 247–253. 10.4161/mabs.1.3.8515 20069754 PMC2726597

[B30] Giancotti F. G. Ruoslahti E. (1999). Integrin Signaling. Science 285, 1028–1033. 10.1126/science.285.5430.1028 10446041

[B31] Gurrath M. Muller G. Kessler H. Aumailley M. Timpl R. (1992). Conformation/activity Studies of Rationally Designed Potent Anti-adhesive RGD Peptides. Eur. J. Biochem. 210, 911–921. 10.1111/j.1432-1033.1992.tb17495.x 1483474

[B32] Haubner R. Finsinger D. Kessler H. (1997). Stereoisomeric Peptide Libraries and Peptidomimetics for Designing Selective Inhibitors of Theαvβ3 Integrin for a New Cancer Therapy. Angew. Chem. Int. Ed. Engl. 36, 1374–1389. 10.1002/anie.199713741

[B33] Heckmann D. Laufer B. Marinelli L. Limongelli V. Novellino E. Zahn G. (2009). Breaking the Dogma of the Metal-Coordinating Carboxylate Group in Integrin Ligands: Introducing Hydroxamic Acids to the MIDAS to Tune Potency and Selectivity. Angew. Chem. Int. Ed. 48, 4436–4440. 10.1002/anie.200900206 19343753

[B34] Heckmann D. Meyer A. Laufer B. Zahn G. Stragies R. Kessler H. (2008). Rational Design of Highly Active and Selective Ligands for the α5β1 Integrin Receptor. Chembiochem 9, 1397–1407. 10.1002/cbic.200800045 18481343

[B35] Heckmann D. Meyer A. Marinelli L. Zahn G. Stragies R. Kessler H. (2007). Probing Integrin Selectivity: Rational Design of Highly Active and Selective Ligands for the α5β1 and αvβ3 Integrin Receptor. Angew. Chem. Int. Ed. 46, 3571–3574. 10.1002/anie.200700008 17394271

[B36] Hoppenz P. Els-Heindl S. Beck-Sickinger A. G. Els-Heindl S. Beck-Sickinger A. G. (2020). Peptide-Drug Conjugates and Their Targets in Advanced Cancer Therapies. Front. Chem. 8, 571. 10.3389/fchem.2020.00571 32733853 PMC7359416

[B37] Humphries J. D. Byron A. Humphries M. J. (2006). Integrin Ligands at a Glance. J. Cell Sci 119, 3901–3903. 10.1242/jcs.03098 16988024 PMC3380273

[B38] Hynes R. O. (2002). Integrins. Cell 110, 673–687. 10.1016/S0092-8674(02)00971-6 12297042

[B39] Jin Z.-H. Furukawa T. Ohya T. Degardin M. Sugyo A. Tsuji A. B. (2017). 67Cu-Radiolabeling of a Multimeric RGD Peptide for αVβ3 Integrin-Targeted Radionuclide Therapy. Nucl. Med. Commun. 38, 347–355. 10.1097/MNM.0000000000000646 28291159

[B40] Kapp T. G. Fottner M. Maltsev O. V. Kessler H. (2016). Small Cause, Great Impact: Modification of the Guanidine Group in the RGD Motif Controls Integrin Subtype Selectivity. Angew. Chem. Int. Ed. 55, 1540–1543. 10.1002/anie.201508713 26663700

[B41] Kapp T. G. Rechenmacher F. Neubauer S. Maltsev O. V. Cavalcanti-Adam E. A. Zarka R. (2017). A Comprehensive Evaluation of the Activity and Selectivity Profile of Ligands for RGD-Binding Integrins. Sci. Rep. 7, 39805. 10.1038/srep39805 28074920 PMC5225454

[B71] Kemker I. Feiner R. C. Müller K. M. Sewald N. (2019). Size-Dependent Cellular Uptake Of RGD Peptides. Chembiochem. 10.1002/cbic.201900512 PMC7064889 31478590

[B42] Kemker I. Schröder D. C. Feiner R. C. Müller K. M. Marion A. Sewald N. (2021). Tuning the Biological Activity of RGD Peptides with Halotryptophans. J. Med. Chem. 64, 586–601. 10.1021/acs.jmedchem.0c01536 33356253

[B43] Khongorzul P. Ling C. J. Khan F. U. Ihsan A. U. Zhang J. (2020). Antibody-Drug Conjugates: A Comprehensive Review. Mol. Cancer Res. 18, 3–19. 10.1158/1541-7786.MCR-19-0582 31659006

[B44] Klim J. R. Fowler A. J. Courtney A. H. Wrighton P. J. Sheridan R. T. C. Wong M. L. (2012). Small-Molecule-Modified Surfaces Engage Cells through the αvβ3 Integrin. ACS Chem. Biol. 7, 518–525. 10.1021/cb2004725 22201290 PMC3306508

[B45] Lerchen H.-G. Stelte-Ludwig B. Kopitz C. Heroult M. Zubov D. Willuda J. (2022). A Small Molecule-Drug Conjugate (SMDC) Consisting of a Modified Camptothecin Payload Linked to an αVß3 Binder for the Treatment of Multiple Cancer Types. Cancers 14, 391. 10.3390/cancers14020391 35053556 PMC8773721

[B46] Malesevic M. Strijowski U. Bächle D. Sewald N. (2004). An Improved Method for the Solution Cyclization of Peptides under Pseudo-high Dilution Conditions. J. Biotechnol. 112, 73–77. 10.1016/j.jbiotec.2004.03.015 15288942

[B72] Marinelli L. Meyer A. Heckmann D. Lavecchia A. Novellino E. Kessler H. (2005). Ligand Binding Analysis For Human alpha5beta1 Integrin: Strategies For Designing New alpha5beta1 Integrin Antagonists. J. Med. Chem. 48, 4204–4207. 10.1021/jm040224i 15974570

[B73] Mas-Moruno C. Rechenmacher F. Kessler H. (2010). Cilengitide: The First Anti-Angiogenic Small Molecule Drug Candidate Design, Synthesis And Clinical Evaluation. Anti-Cancer Agents Med. Chem 10, 753–768. 10.2174/187152010794728639 PMC3267166 21269250

[B47] Mas-Moruno C. Fraioli R. Rechenmacher F. Neubauer S. Kapp T. G. Kessler H. (2016a). αvβ3- oder α5β1-Integrin-selektive Peptidmimetika für die Oberflächenbeschichtung. Angew. Chem. 128, 7162–7183. 10.1002/ange.201509782

[B48] Mas-Moruno C. Fraioli R. Rechenmacher F. Neubauer S. Kapp T. G. Kessler H. (2016b). αvβ3- or α5β1-Integrin-Selective Peptidomimetics for Surface Coating. Angew. Chem. Int. Ed. 55, 7048–7067. 10.1002/anie.201509782 27258759

[B49] Medina-Franco J. L. Edwards B. S. Pinilla C. Appel J. R. Giulianotti M. A. Santos R. G. (2013). Rapid Scanning Structure-Activity Relationships in Combinatorial Data Sets: Identification of Activity Switches. J. Chem. Inf. Model. 53, 1475–1485. 10.1021/ci400192y 23705689 PMC3715655

[B50] Medina-Franco J. L. (2012). Scanning Structure-Activity Relationships with Structure-Activity Similarity and Related Maps: from Consensus Activity Cliffs to Selectivity Switches. J. Chem. Inf. Model. 52, 2485–2493. 10.1021/ci300362x 22989212

[B51] Medina-Franco J. L. Yongye A. B. Pérez-Villanueva J. Houghten R. A. Martínez-Mayorga K. (2011). Multitarget Structure-Activity Relationships Characterized by Activity-Difference Maps and Consensus Similarity Measure. J. Chem. Inf. Model. 51, 2427–2439. 10.1021/ci200281v 21842860

[B52] Nagy A. Schally A. V. Armatis P. Szepeshazi K. Halmos G. Kovacs M. (1996). Cytotoxic Analogs of Luteinizing Hormone-Releasing Hormone Containing Doxorubicin or 2-pyrrolinodoxorubicin, a Derivative 500-1000 Times More Potent. Proc. Natl. Acad. Sci. 93, 7269–7273. 10.1073/pnas.93.14.7269 8692981 PMC38972

[B53] Nahrwold M. Weiß C. Bogner T. Mertink F. Conradi J. Sammet B. (2013). Conjugates of Modified Cryptophycins and RGD-Peptides Enter Target Cells by Endocytosis. J. Med. Chem. 56, 1853–1864. 10.1021/jm301346z 23387527

[B54] Nieberler M. Reuning U. Reichart F. Notni J. Wester H.-J. Schwaiger M. (2017). Exploring the Role of RGD-Recognizing Integrins in Cancer. Cancers 9, 116. 10.3390/cancers9090116 PMC5615331 28869579

[B55] Owen R. M. Carlson C. B. Xu J. Mowery P. Fasella E. Kiessling L. L. (2007). Bifunctional Ligands that Target Cells Displaying the αvβ3 Integrin. Chembiochem 8, 68–82. 10.1002/cbic.200600339 17154219

[B56] Pérez-Villanueva J. Santos R. Hernández-Campos A. Giulianotti M. A. Castillo R. Medina-Franco J. L. (2011). Structure-activity Relationships of Benzimidazole Derivatives as Antiparasitic Agents: Dual Activity-Difference (DAD) Maps. Med. Chem. Commun. 2, 44–49. 10.1039/c0md00159g

[B57] Pina A. Dal Corso A. Caruso M. Belvisi L. Arosio D. Zanella S. (2017). Targeting Integrin αV β3 with Theranostic RGD-Camptothecin Conjugates Bearing a Disulfide Linker: Biological Evaluation Reveals a Complex Scenario. ChemistrySelect 2, 4759–4766. 10.1002/slct.201701052

[B74] Poreba M. (2020). Protease-Activated Prodrugs: Strategies, Challenges, And Future Directions. FEBS J. 287, 1936–1969. 10.1111/febs.15227 31991521

[B58] Rechenmacher F. Neubauer S. Polleux J. Mas-Moruno C. De Simone M. Cavalcanti-Adam E. A. (2013). Functionalizing αvβ3- or α5β1-Selective Integrin Antagonists for Surface Coating: A Method to Discriminate Integrin Subtypes *In Vitro* . Angew. Chem. Int. Ed. 52, 1572–1575. 10.1002/anie.201206370 23345131

[B59] Rékási Z. Szöke B. Nagy A. Groot K. Rékási E. S. Schally A. V. (1993). Effect of Luteinizing Hormone-Releasing Hormone Analogs Containing Cytotoxic Radicals on the Function of Rat Pituitary Cells: Tests in a Long Term Superfusion System. Endocrinology 132, 1991–2000. 10.1210/endo.132.5.8477650 8477650

[B60] Rockwell A. L. Rafalski M. Pitts W. J. Batt D. G. Petraitis J. J. DeGrado W. F. (1999). Rapid Synthesis of RGD Mimetics with Isoxazoline Scaffolds on Solid Phase: Identification of αvβ3 Antagonists lead Compounds. Bioorg. Med. Chem. Lett. 9, 937–942. 10.1016/S0960-894X(99)00114-6 10230615

[B61] Schaffner F. Ray A. Dontenwill M. (2013). Integrin α5β1, the Fibronectin Receptor, as a Pertinent Therapeutic Target in Solid Tumors. Cancers 5, 27–47. 10.3390/cancers5010027 24216697 PMC3730317

[B62] Slack R. J. Macdonald S. J. F. Roper J. A. Jenkins R. G. Hatley R. J. D. (2022). Emerging Therapeutic Opportunities for Integrin Inhibitors. Nat. Rev. Drug Discov. 21, 60–78. 10.1038/s41573-021-00284-4 34535788 PMC8446727

[B63] Srinivasarao M. Galliford C. V. Low P. S. (2015). Principles in the Design of Ligand-Targeted Cancer Therapeutics and Imaging Agents. Nat. Rev. Drug Discov. 14, 203–219. 10.1038/nrd4519 25698644

[B64] Staudacher A. H. Brown M. P. (2017). Antibody Drug Conjugates and Bystander Killing: Is Antigen-dependent Internalisation Required? Br. J. Cancer 117, 1736–1742. 10.1038/bjc.2017.367 29065110 PMC5729478

[B75] Tala S. R. Schnell S. M. Haskell-Luevano C. (2015). Microwave-Assisted Solid-Phase Synthesis of Side-Chain to Side-Chain Lactam-Bridge Cyclic Peptides. Bioorg. Med. Chem. Lett. 25, 5708–5711. 10.1016/j.bmcl.2015.10.095 26555357 PMC4654418

[B65] Wei B. Gunzner-Toste J. Yao H. Wang T. Wang J. Xu Z. (2018). Discovery of Peptidomimetic Antibody-Drug Conjugate Linkers with Enhanced Protease Specificity. J. Med. Chem. 61, 989–1000. 10.1021/acs.jmedchem.7b01430 29227683

[B66] Zanella S. Angerani S. Pina A. López Rivas P. Giannini C. Panzeri S. (2017). Tumor Targeting with an Iso DGR-Drug Conjugate. Chem. Eur. J. 23, 7910–7914. 10.1002/chem.201701844 28449309 PMC5488297

