# Supplementary material for: Synthesis and Evaluation of a Non-Peptide Small-Molecule Drug Conjugate Targeting Integrin αVβ3
Source: Front Chem. 2022 Apr 11;10:869639. doi: 10.3389/fchem.2022.869639 (PMC9035832; doi:10.3389/fchem.2022.869639)

Supplementary Material

Synthesis and Evaluation of a Non-Peptide Small Molecule-Drug Conjugate Targeting Integrin $\alpha_V\beta_3$

Jannik Paulus[1]**, Norbert Sewald**[1*]

[1]Organic and Bioorganic Chemistry, Department of Chemistry Bielefeld University, Universitätsstraße 25, 33615 Bielefeld, Germany

*** Correspondence:** Norbert Sewald, norbert.sewald@uni-bielefeld.de

Table of Contents

1 Figures 2
1.1 Selectivity-Activity plot 2
1.2 DAD maps 3
2 Materials and Methods 7
2.1 General specifications 7
2.2 NMR Spectroscopy 7
2.3 LCMS 7
2.4 Purification *via* column chromatography/RP-HPLC 7
2.5 ELISA-like assay 8
2.6 Flow cytometry 9
2.7 Cell adhesion assay 11
2.8 Cell viability assay 11
3 Synthesis 12
3.1 General procedures 12
3.2 Pre-stages RGD mimetics 17
3.3 Benzoic acid-PEG-linker 22
3.4 Succinidyl-PEG-linker 25
3.5 RGD mimetic precursors 27
3.6 Finale small molecular RGD-mimetics 33
3.7 Precursors for conjugable RGD-mimetics 56
3.8 Conjugable final RGD-mimetics 59
3.9 Conjugable cyclic control RGD mimetics 61
3.10 SMDC synthesis 63
4 Spectroscopic and analytical Data 68

1 Figures

1.1 Selectivity-Activity Plot

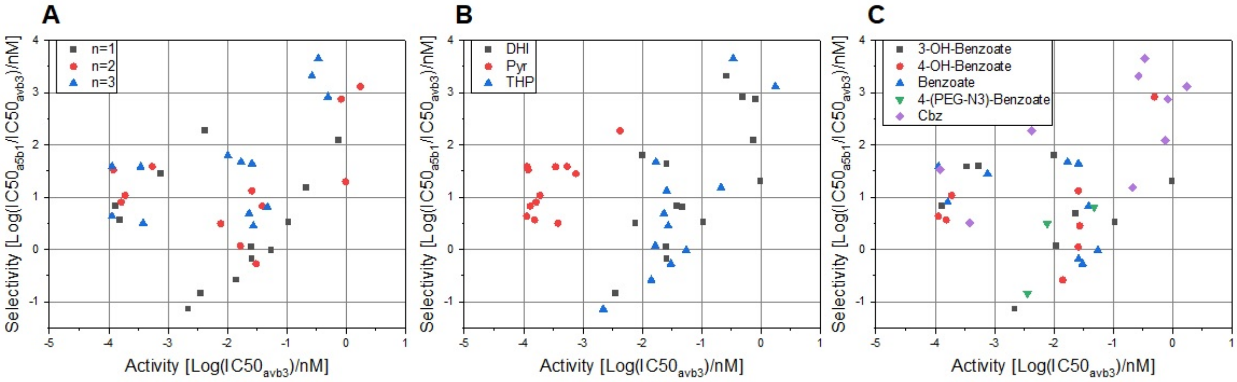

Figure S1: Selectivity-Activity plot for ELISA results (**Table 4**). X-axis shows the activity for the integrin $\alpha_v\beta_3$, and the Y-axis reveals the selectivity for integrin $\alpha_v\beta_3$ against $\alpha_5\beta_1$. Highlighted in colour are the changes in the three different parameters: **A** length, **B** guanidyl analogue group and **C** the *N*-terminal aromatic moiety.

1.2 DAD Maps

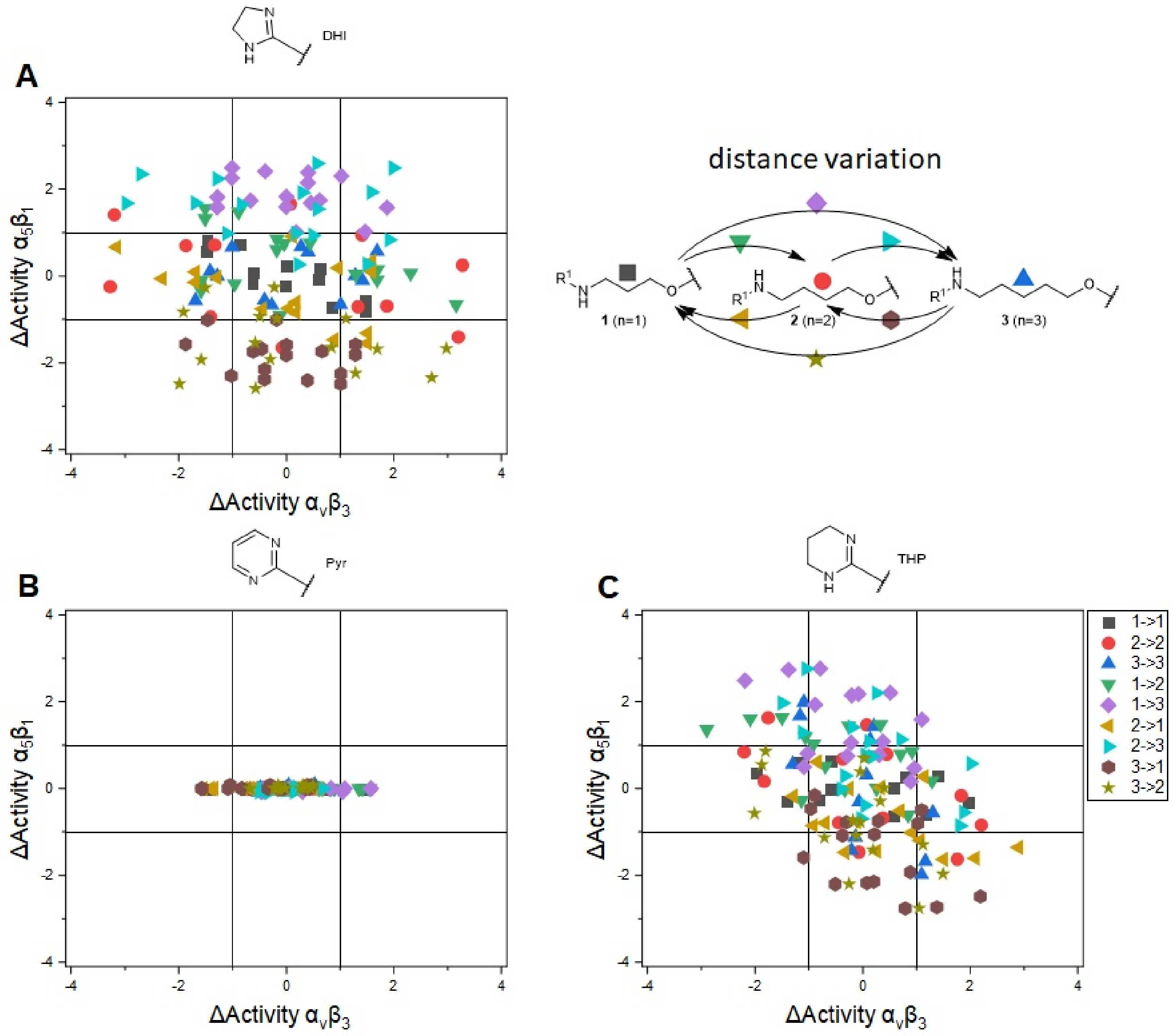

Figure S2: DAD maps based on ELISA data (**Table 4**). The panels show the structural changes between two molecules with **A** DHI, **B** Pyr and **C** THP as constant structure motif within the changes of the length are highlighted in colour. The structure switch in the aromatic moiety is unmarked.

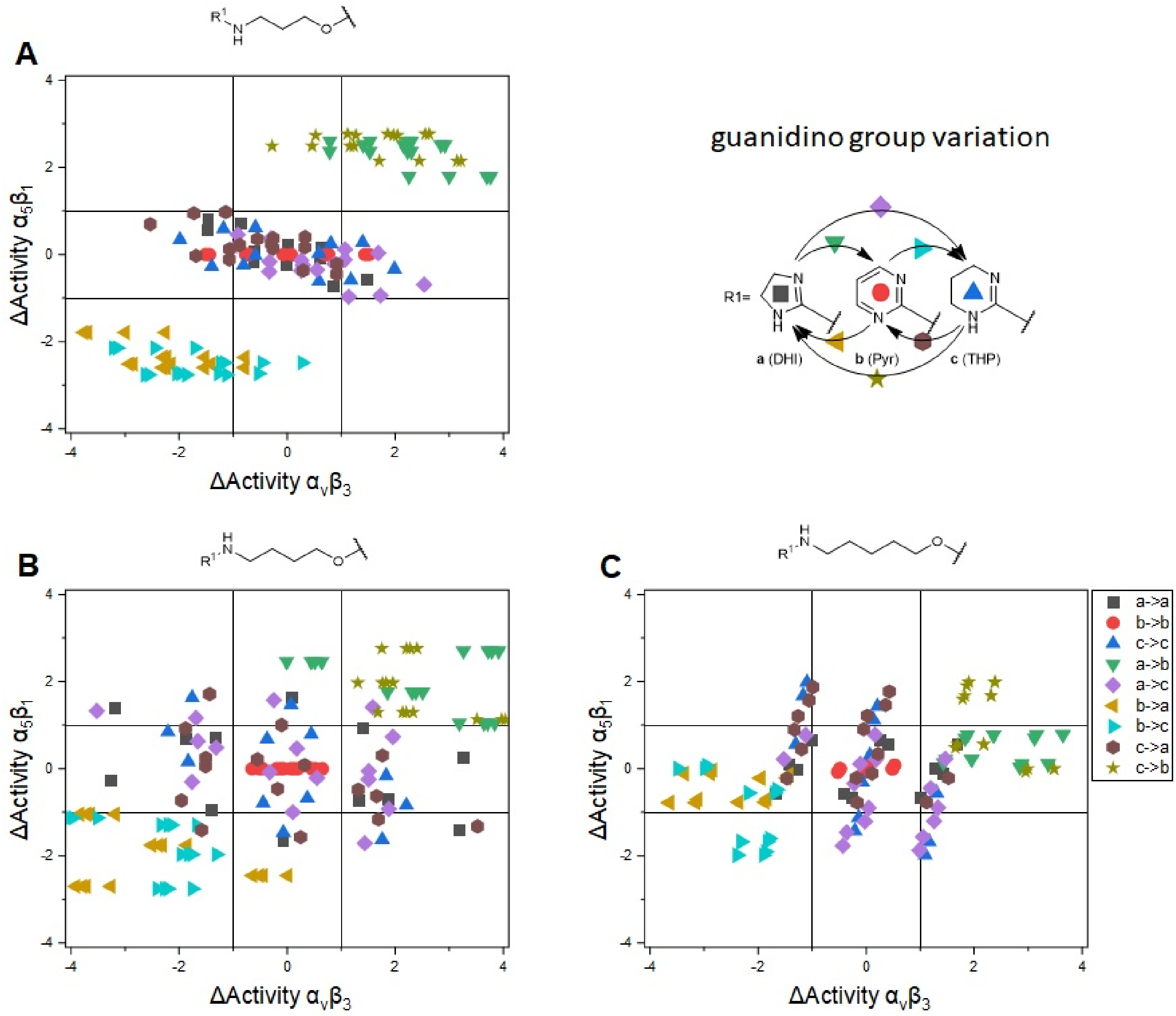

Figure S3: DAD maps based on ELISA data (**Table 4**). The panels show the structural changes between two molecules with **A** n=1, **B** n=2 and **C** n=3 as constant structure motif within the changes of $\mathbf{R^1}$ are highlighted in colour. The structure switch in the aromatic moiety is unmarked.

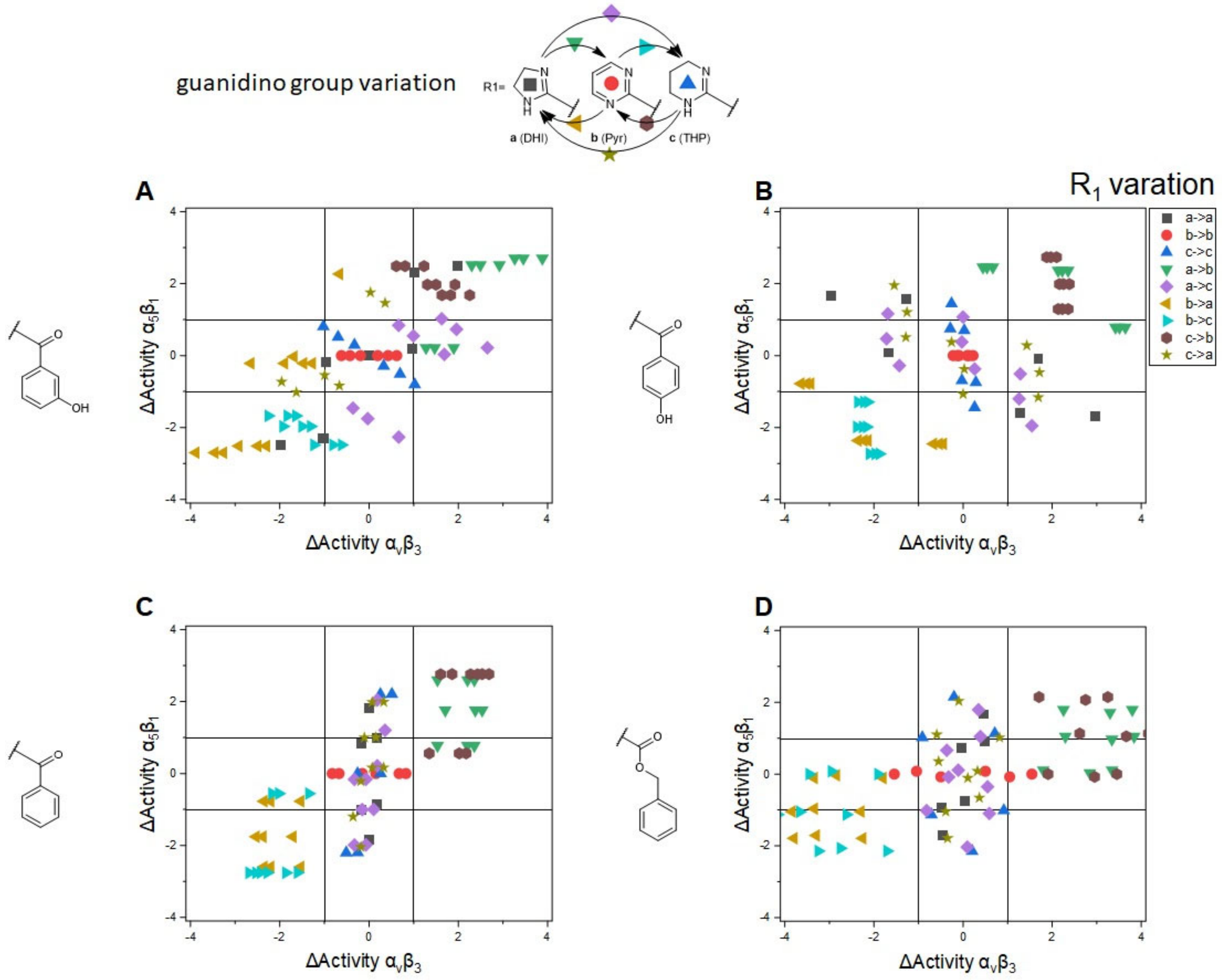

Figure S4: DAD maps based on ELISA results (**Table 4**). The panels show the structural changes between two molecules with **A** 3-OH-benzoate, **B** 4-OH-benzoate, **C** benzoate and **D** Cbz as constant structure motif within the changes of the guanidyl analogue group are highlighted in colour. The structure switch in the length is unmarked.

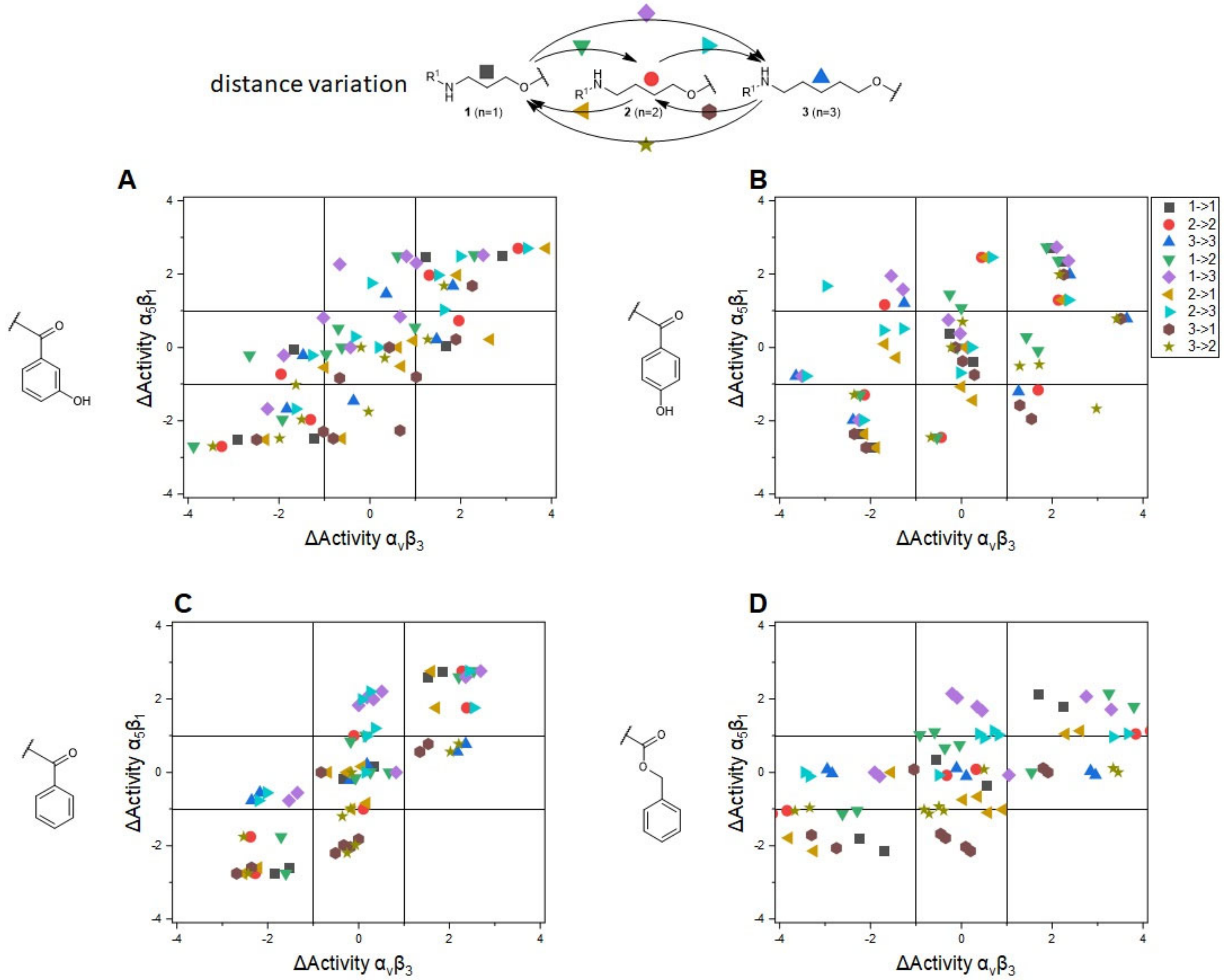

Figure S5: DAD maps were calculated by ELISA results (**Table 4**). The panels show the structural changes between two molecules with **A** 3-OH-benzoate, **B** 4-OH-benzoate, **C** benzoate and **D** Cbz as constant structure motif within the changes of the length is highlighted in colour. The structure switch in the guanidyl analogue group is unmarked.

2 Materials and Methods

2.1 General Specifications

Dichloromethane (DCM), petroleum ether, diethyl ether, and ethyl acetate were purchased as technical grade and distilled before usage. All other solvents were used as purchased (analytical grade). For further drying DMF was stored over mole sieve, DCM was freshly distilled over CaH and THF over sodium. Inert reactions took place under an argon atmosphere and in heated equipment. Distilled water was used in every case and MPW was purified by PURELAB® flex 2.

2.2 NMR Spectroscopy

NMR spectra were recorded on a Bruker Avance 600 (600 MHz for ^{1}H, 564 MHz for ^{19}F, 150 MHz for ^{13}C) and a Bruker Avance III 500HD (500 MHz for ^{1}H, 126 MHz for ^{13}C, 471 MHz for ^{19}F). The chemical shift δ is reported in ppm relative to the residual proton signal of the solvent: $CDCl_3$ 7.26 ppm (^{1}H), 77.2 ppm (^{13}C); DMSO-d_6 2.50 ppm (^{1}H), 39.52 ppm (^{13}C); CD_3OD 3.31 ppm (^{1}H), δ 49.0 ppm (^{13}C). 2D methods (HMBC, HMQC, COSY) were used to support and confirm the assignment.

2.3 LCMS

LCMS analysis was performed with an Agilent 6220 TOF-MS with a Dual ESI-source, 1200 HPLC system (Agilent) with autosampler, degasser, binary pump, column oven, diode array detector and a Hypersil Gold C18 column (1.9 µm, 50 × 2.1 mm). The gradient started with 100 % A (water/ACN/formic acid, 94.9:5:0.1) during 11 min the percentage of eluent B (ACN/water/formic acid, 94.9:5:0.1) increases from 0 % to 98 % B and returned to 0 % B in 0.5 min. Total run time was 15 min at a flow rate of 0.3 mL/min and column oven temperature of 40°C. After separation *via* the 1200 HPLC system ESI mass spectra were recorded in extended dynamic range mode equipped with a Dual-ESI source, operating with a spray voltage of 2.5 kV. The same system was used for high resolution mass spectroscopy.

2.4 Purification by Column Chromatography/RP-HPLC

Normal column chromatography was performed with silica gel (particle size: 40-60 µm) from Merck. Automatically column chromatography (MPLC, medium performance liquid chromatography) was carried out with a Büchi Reveleris X2 system and purchased columns. Polar compounds and final products were purified via preparative reverse phase HPLC (RP-HPLC, Thermo Separation Products) consisting of a degasser, a pump (P4000), a Thermo Scientific Hypersil gold column (8 µm,

250 x 21.2 mm cartridge) and a UV- detector (UV1000). The gradients were chosen depending on the compound with the eluents A (water/ACN/TFA, 94.9:5:0.1) and B (ACN/water/TFA, 94.9:5:0.1).

2.5 ELISA-like Assay

Table S1: Proteins and buffers applied in the ELISA-like assay.

	condition	composition	company
$\alpha_V\beta_3$	(1)	1.0 µg/mL human vitronectin	PeproTech
	(2)	2.0 µg/mL human avb3 integrin	Sino Biological
	(3)	2.0 µg/mL mouse anti-human CD51/CD61	BD Bioscience
	(4)	1.0 µg/mL anti-mouse IgG-POD goat	Sigma-Aldrich
$\alpha_5\beta_1$	(1)	0.5 µg/mL human fibronectin	R&D
	(2)	2.0 µg/mL human avb3 integrin	R&D
	(3)	1.0 µg/mL mouse anti-human CD51/CD61	BD Bioscience
	(4)	2.0 µg/mL anti-mouse IgG-POD goat	Sigma-Aldrich
buffer	carbonate	15 mM Na_2CO_3, 35 mM $NaHCO_3$, pH 9.6	
	PBS-T	137 mM NaCl, 2.7 mM KCl, 10 mM Na_2HPO_4,2 mM KH_2PO_4, 0.01% Tween20, pH 7.4	
	TS-B	20 mM Tris–HCl, 150 mM NaCl, 1 mM $CaCl_2$,1 mM $MgCl_2$, 1 mM $MnCl_2$, pH 7.5, 1% BSA	

An ELISA-like assay using the isolated extracellular domain of integrins $\alpha_V\beta_3$ and $\alpha_5\beta_1$ was performed in flat-bottom 96-well immuno plates (Brand) to determine the activities of the synthesised compounds. All wells were coated overnight with the native integrin ligand vitronectin or fibronectin (**Table S1,** (1)) in carbonate buffer (150 µL/well), followed by washing of each well with PBS-T buffer (3×200 µL/well) (**Table S1**) and blocking for 1 h with TS-B buffer (150 µL/well) at RT. A dilution series was prepared using the internal standard (Cilengitide, 1:5 dilution) and the compounds (1:5 or 1:10 dilution) in TS-B buffer. The protein-coated assay plate was washed with PBS-T buffer (3×200 µL/well) and 50 µL of the dilution series was transferred to the assay plate wells B-G. TS-B buffer was filled into row A (100 µL/well) as negative control and row H (50 µL/well) as positive control. Afterwards, the corresponding human integrin (2, 50 µL/well)) (**Table S1**) in TS-B buffer was added to row B-H and incubated for 1 h at RT. After washing the assay plate with PBS-T buffer (3×200 µL/well) the primary antibody (3, 100 µL/well) (**Table S1**) was transferred to each well and incubated for 1 h at RT. Then the plate was washed with PBS-T buffer (3×200 µL/well), treated with

the secondary antibody (4, 100 µL/well) (**Table S1**) and incubated for 1 h at RT. The plate was washed with PBS-T buffer (3×200 µL/well) and SeramunBlau® fast2 (Seramun Diagnostics GmbH, 50 µL/well) was added to each well. Staining was stopped with 3 M aq. H_2SO_4 (50 µL/well) when the rows of the internal standard (Cilengitide) showed a blue colour gradient from well A to H ($\alpha_V\beta_3$: 40 s; $\alpha_5\beta_1$: 1.5 min). The absorbance was measured with a plate reader at 450 nm and corrected by subtraction of the absorbance at 620 nm. Afterwards, the resulting values were plotted and analysed using OriginPro® 2020b where the inflection point of a DoseResp fit describes the IC_{50} value. All compounds were tested in duplicates or triplicates for both integrins.

2.6 Flow Cytometry

Table S2: Proteins applied in the flow cytometric determination of the integrin status for WM115 and M21-L cells.

integrin	condition	volume [µL]	protein	company
α_V	(1)	0.60	MAB1978 mouse anti-human CD51, 0.5 mg/mL	Sigma-Aldrich
	(2)	1.25	Alexa Fluor® 488 goat anti-mouse IgG (H+L), 2 mg/mL	Invitrogen
$\alpha_V\beta_3$	(1)	0.60	Mouse anti-human CD51/CD61, 0.5 mg/mL	BD Bioscience
	(2)	1.25	Alexa Fluor® 488 goat anti-mouse IgG (H+L), 2 mg/mL	Invitrogen
$\alpha_V\beta_6$ (WM115)	(1)	0.60	Anti-integrin $\alpha_V\beta_6$ rabbit anti-human, 0.3 mg/mL	ZooMAb®
	(2)	1.25	Alexa Fluor® 568 goat anti-rabbit IgG (H+L), 2 mg/mL	Invitrogen
$\alpha_V\beta_6$ (M21-L)	(1)	1.50	PE mouse anti-human integrin $\alpha_V\beta_6$, 0.2 mg/mL	BD Bioscience
	(2)	-	-	-
$\alpha_V\beta_8$	(1)	0.60	Anti-integrin $\alpha_V\beta_8$ rabbit anti-human, 0.3 mg/mL	ZooMAb®
	(2)	1.25	Alexa Fluor® 568 goat anti-rabbit IgG (H+L), 2 mg/mL	Invitrogen
$\alpha_5\beta_1$	(1)	0.60	Mouse anti-human CD49e, 0.5 mg/mL	BD Bioscience
	(2)	1.25	Alexa Fluor® 488 goat anti-mouse IgG (H+L), 2 mg/mL	Invitrogen

WM115 and M21-L cells were seeded in 12-well plates or cell culture flasks and incubated at 37 °C for one to two days. The cells were detached with Accutase solution (Pan Biotech), washed with medium and resuspended in PBS buffer (137 mM NaCl, 2.7 mM KCl, 10 mM Na_2HPO_4,2 mM KH_2PO_4, 300 µL).

Then the primary antibody (1) (**Supplementary Table S2**) was added followed by incubation for 15 min on ice. Subsequently, cells were centrifuged (10 min, 1800 rpm/350 g) and washed with PBS (800 µL, 10 min 1800rpm/350 g). After resuspension in PBS (300 µL) the secondary antibody (2) (**Supplementary Table S2**) was added, and the cells were incubated for 15 min on ice. Finally, the cells were centrifuged, washed and resuspended as described as well as measured with a S3e Cell Sorter (BioRad) by excitation at 488 nm and 568 nm. For each sample 30000 events were measured. As controls pure cells and cells treated only with the secondary antibody (2) (**Supplementary Table S2**) were measured. Results are shown in **Supplementary Figure S6**.

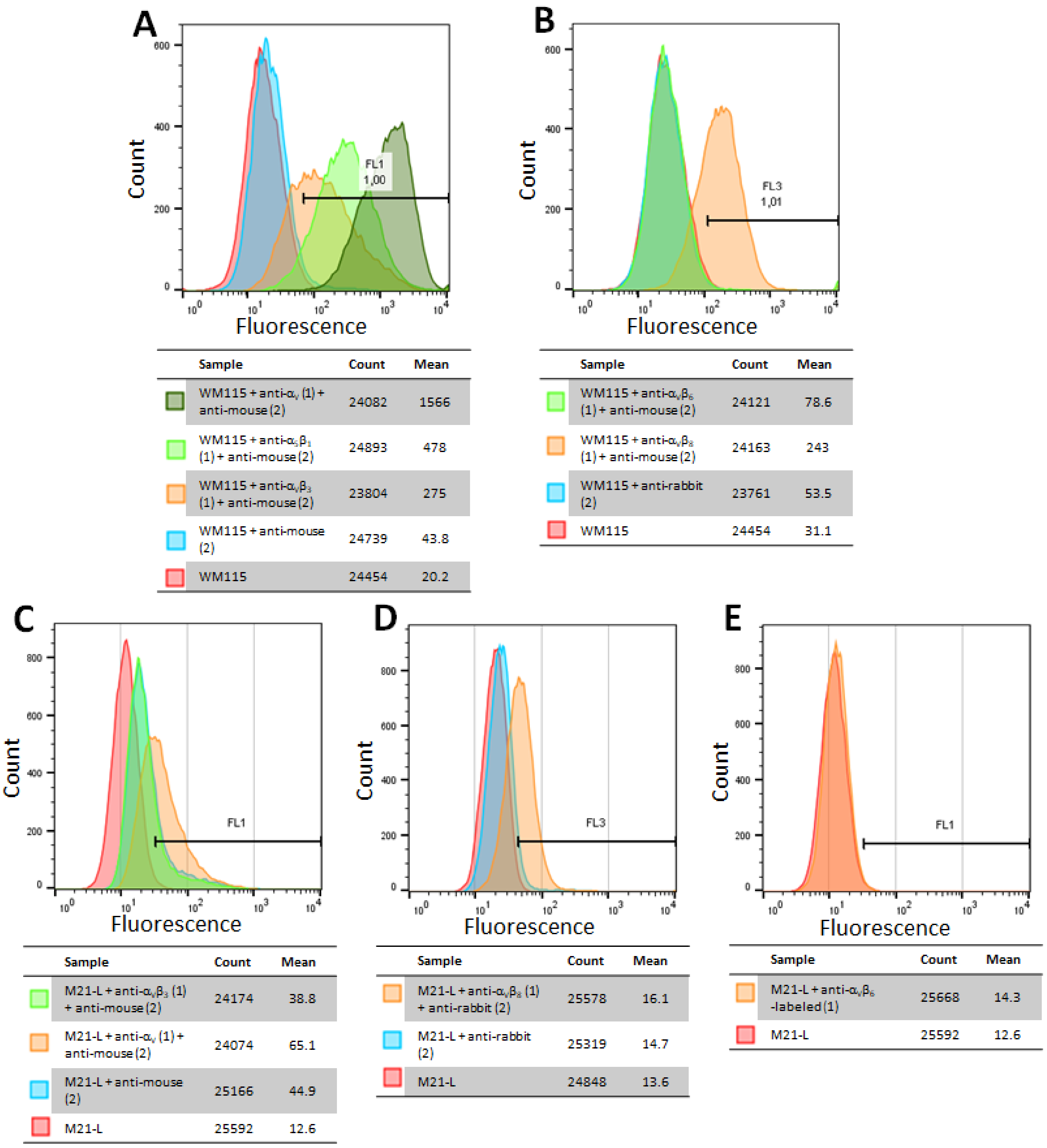

Figure S6: Flow cytometrically determined integrin status of WM115 (**A-B**) and M21-L (**C-E**) cells for integrin $\alpha_V\beta_3$, $\alpha_V\beta_6$, $\alpha_V\beta_8$ and α_V-unit as well as integrin $\alpha_5\beta_1$ for WM115 cells.

2.7 Cell Adhesion Assay

WM115 cells were cultivated and used in MEM Eagle medium (Pan Biotech P04-08500 with 10 % foetal bovine serum, 50 µg/mL gentamycin, 0.5 mM sodium pyruvate) and M21-L cells in RPMI medium (Pan Biotech P04-16500 with 10 % foetal bovine serum, 1 % Pen-Strep). Overnight a flat-bottom MaxiSorp Nunc 96-well plate was coated with recombinant human vitronectin (100 µL/well, 1 µg/mL, Peprotech) in PBS buffer (137 mM NaCl, 2.7 mM KCl, 10 mM Na_2HPO_4,2 mM KH_2PO_4, pH 7.4) at 4 °C and blocked at the following day by adding a solution of fatty acid free milk powder in PBS buffer (5 w/v %, 100 µL/well) at 4 °C. The WM115 and M21-L cells were washed with PBS buffer, detached with Accutase solution (5 mL, Pan Biotech P10-21100) at 37 °C for 5 min and then diluted with medium (15 mL). After centrifugation (850 rpm, 6 min) the resulting cell pellet was resuspended with fluorescein diacetate in medium (fluorescein diacetate 1.5 mg/mL, cell density $5 \cdot 10^5$ cells/mL) and incubated for 30 min at 37 °C in the dark. The cells were washed twice with medium and then resuspended in medium (cell density 5×10^5 cells/mL). Afterwards a solution of $CaCl_2$, $MnCl_2$ and $MgCl_2$ (each 100 mM) in PBS buffer (90 µL) was transferred to the cells and incubated for 30 min on ice in the dark. In the meantime, a dilution series of the compounds in medium (1:3 dilution) was prepared and 240 µL were transferred to Eppendorf tubes. Pure medium was used as positive control. Cell suspension (240 µL) was added to each tube of the dilution series and the control followed by incubation at 37 °C for 30 min in the dark. The assay plate was discharged and washed with 200 µL/well medium. Then the cell suspension with different compound concentrations was added to the assay plate (100 µL/well) and incubated for 60 min at 37 °C in the dark. Afterwards, the assay plate was washed with medium (3×100 µL/well), finally medium (100 µL/well) was added and the fluorescence was measured with a TecanReader (Excitation: 480 nm; Emission: 520 nm). The determined values were plotted and analysed using OriginPro® 2020b where the inflection point of a DoseResp fit describing the IC_{50} value.

2.8 Cell Viability Assay

WM115 cells were cultivated in MEM medium (with 10 % foetal bovine serum, 50 µg/mL gentamycin, 0.5 mM sodium pyruvate) at 37 °C and 5.3 % CO_2-humidified air in an incubator. The cells were seeded in a sterile flat-bottom cell culture 96-well plate (Sarstedt) in a density of 10000 cells/well (100 µL/well) and incubated for 1 day as described. A dilution series (1:3 dilution) of the compounds and the standard (cryptophycin-52) in medium was prepared and transferred to the assay plate (100 µL/well) followed by incubation for 3 d as described. Afterwards a solution of resazurin (175 µM, 30 µL/well) was added followed by incubation for 6 h. Subsequently, the fluorescence was measured with a TecanReader (Excitation: 530 nm; Emission: 588 nm) as well as plotted and analysed using OriginPro® 2020b where the inflection point of a DoseResp fit describes the IC_{50} value.

3 Synthesis

3.1 General Procedures

3.1.1 GP-1: Boc-Protection

Compounds: **2a**, **2b**, **2c**

Boc anhydride (1.2 eq) was dissolved in a mixture of water and dioxane as well as cooled to 0 °C with an ice bath. Subsequently the corresponding amino alcohol (1 eq.) was added following by addition of triethylamine (2 eq.) The reaction progress was monitored by TLC and after full conversion the reaction mixture was diluted with water and ethyl acetate. After phase separation the water layer was extracted with ethyl acetate (3×) and the combined organic layers were washed with sat. NaCl (aq.) and dried over $MgSO_4$. After evaporating the solvent, the desired *N*-Boc protected amino alcohol was obtained as a highly viscous liquid and was used without further purification.

3.1.2 GP-2: Mitsunobu reaction

Compounds: **3a**, **3b**, **3c**, **9**

The corresponding amino alcohol (1.1 eq.), Cbz-Tyr-OMe (1 eq.) and triphenylphosphine (1.2 eq.) were dissolved in dry THF in baked-out equipment under inert conditions. The solution was cooled to 0 °C with an ice bath and diisopropyl azodicarboxylate (DIAD, 1.2 eq.), dissolved in THF (30 mL) was added dropwise during 1.5 h. After removing the cooling bath the reaction mixture was stirred over night at room temperature. Subsequently the solution was diluted with sat. $NaHCO_3$ (aq.) and the water layer was extracted with ethyl acetate (3×). The combined organic layers were dried over $MgSO_4$, the solvent was removed under reduced pressure and the crude product was purified by automatic column chromatography (MPLC, gradient of petroleum ether and ethyl acetate) to obtain the desired alkyl aryl ethers as a colourless foam.

3.1.3 GP-3: Cbz-Cleavage

Compounds: **4a**, **4b**, **4c**

The Cbz-protected compound (1 eq.) was dissolved in MeOH and $Pd(OH)_2/C$ (10 % Pd, 0.1 eq.) was added to give a black suspension. Hydrogen was bubbled through the reaction mixture and the reaction progress was monitored by LCMS. After full conversion the suspension was filtered through a thin pad of celite. Afterwards the solvent was removed under reduced pressure and the product was dried in vacuum to get the desired unprotected compound as a colourless solid.

3.1.4 GP-4a: *N*-Terminal modification of precursor RGD mimetics with acid chlorides

Compounds: **5a**, **6a**, **7a**

Triethylamine (3 eq.) was added to a solution of the corresponding deprotected compound (1 eq.) in 2 mL DMF/DCM (1:1, v:v) and after stirring for 5 min benzoylchloride (1.5 eq.) was added. The reaction progress was monitored via LCMS and after full conversion the solvent was removed. Afterwards the crude was dried in vacuum, the resulting solid was dissolved in a small amount of water/ACN (1:1, v:v) and purified via preparative RP-HPLC.

3.1.5 GP-4b: *N*-Terminal modification of precursor RGD mimetics with carboxylic acids

Compounds: **5b**, **5c**, **6b**, **6c**, **7b**, **7c**, **24a**, **24b**, **24c**

A HOBt solution (1.3 M in DMF, 1.3 eq.) was added to the corresponding 4- or 3-hydroxy benzoic acid (2.4 M in DMF, 1.2 eq.), followed by an EDC solution (0.5 M in DMF/DCM (1:1, v:v), 1.4 eq.) and DIPEA (2 eq.). The solution was stirred for two minutes and was then added to the solution of the amino component **3a-c** (1 eq.) in DMF/DCM (1:1, v:v, 1 mL). The reaction mixture was stirred at room temperature and the reaction progress was monitored by LCMS. After full consumption of the amine the reaction was stopped by removing the solvent. Afterwards the crude was dried in vacuum and the resulting solid was dissolved in a small amount of water/ACN (1:1, v:v) and purified via preparative RP-HPLC.

3.1.6 GP-5a: Synthesis of final DHI substituted RGD-mimetics

Compounds: **5aa**, **5ba**, **5ca**, **6aa**, **6ba**, **6ca**, **7aa**, **7ba**, **7ca**

HCl in dioxane (4 M, 100 µL, 13.3 eq.) was added to a solution of protected RGD mimetic precursors **5a-c**, **6a-c** and **7a-c** (0.1 M in DCM, 300 µL, 1 eq.). After stirring for 1.5 h the solvent was removed under reduced pressure and a solution of 2-methylthio-2-imidazoline hydroiodide (0.18 M in MeOH/NEt_3 (1:1., v:v), 416 µL, 2.5 eq.) was added. The mixture was heated to 80 °C in a sealed tube till consumption of the free amine (LC-MS) followed by solvent removal. The residue was then dissolved in a LiOH solution (0.285 M in MeOH/water (3:1, v:v), 526 µL, 5 eq.) and stirred at room temperature. Monitoring of the reaction progress was done via LCMS. After complete conversion the crude mixture was concentrated and purified via preparative RP-HPLC.

3.1.7 GP-5b-d: Synthesis of final pyrimidine (Pyr) and THP substituted RGD-mimetics

Compounds: **5ab**, **5bb**, **5cb**, **5ac**, **5bc**, **5cc**, **5db**, **5dc**, **6ab**, **6bb**, **6cb**, **6ac**, **6bc**, **6cc**, **6db**, **6dc**, **7ab**, **7bb**, **7cb**, **7ac**, **7bc**, **7cc**, **7db**, **7dc**

It was followed procedure **GP-5a** with 2-bromopyrimidine (0.36 M in MeOH/NEt_3 1:1., v:v, 416 µL, 5 eq.) instead of 2-methylthio-2-imidazoline hydroiodide. After ester hydrolysis the reaction batch was split in two equal amounts (1. **GP-5b**, 2. **GP-5c** or **GP-5d**) and followed by:

GP-5b for Pyr substituted mimetics: A half of the reaction mixture was concentrated and purified via preparative RP-HPLC to give the pyrimidine substituted final RGD mimetic.

GP-5c for THP in case of benzoyl substituted mimetics: A half of the reaction mixture was combined with a suspension of Pd/C (10mg·mL^{-1}, 234 µL) and 100 µL acetic acid. Afterwards hydrogen was bubbled through the suspension till LC-MS show full conversion. The reaction mixture was concentrated, centrifuged, and purified by preparative RP-HPLC to obtain the desired reduced RGD mimetic as TFA salt.

GP-5d for THP in case of Cbz protected mimetics: A half of the reaction mixture was combined with Pd/C (10 % Pd, 0.1 eq.), 2-bromopyrimidine (10 eq.), HBr in AcOH (10 eq.), AcOH (200 eq.) and water (400 eq.) in MeOH to result in a 10 mM solution based on the half of the starting material. Hydrogen was bubbled through the suspension upon vigorous stirring till LC-MS showed full conversion. The reaction mixture was centrifuged, the solid residue was discarded, and the solution was diluted with water and freeze-dried. Afterwards, the residue was purified by preparative RP-HPLC to obtain the desired reduced RGD mimetic as TFA salt.

3.1.8 GP-6: Synthesis of linear peptides

Resin loading: The Fmoc/^{t}Bu-strategy was chosen for the synthesis of linear peptides and peptide based enzymatically cleavable linkers. Resin loading and subsequent coupling steps were performed in a syringe and on an automatic shaker. Barlos/2-Chlorotrityl chloride resin (CTC-resin, 1.5 mmol/g) was swollen in DCM (10 mL/g resin) for 10-15 min at room temperature. Afterwards the solvent was removed and a solution of the loading amino acid (1 eq. corresponding to resin) and DIPEA (10 eq.) in DCM (10 mL/g resin) was added. After incubation for 3 h at room temperature MeOH (2 mL/g resin) was added and the mixture was shaken for further 30 min. Then the resin was washed with DMF (5×) and DCM (3×) and dried in vacuum to determine the resin loading by UV-analysis of the piperidine-dibenzofulvene adduct formed upon cleavage of the Fmoc protecting group with 20 % piperidine in DMF.

Fmoc-cleavage and coupling of amino acids: After resin loading the resin was swollen in DMF for 10 min. Fmoc-cleavage was performed twice with 20 % piperidine in DMF (4 min ultrasonic bath 25 °C

followed by 5 min on shaker at r.t., 5 mL/g resin) followed by washing with DMF (5×10 mL/g resin), DCM (2×10 mL/g resin) and DMF (2×10 mL/g resin). For the coupling step the corresponding amino acid (4 eq.), DIC (4 eq.) and Oxyma (4 eq.) were dissolved in DMF (10 mL/g resin) and added to the reaction syringe containing the resin, followed by sonication for 4 min and further shaking for 5 min. Afterwards the resin was washed again with DMF (5×10 mL/g resin), DCM (2×10 mL/g resin) and DMF (2×10 mL/g resin). The coupling result was checked by Kaiser test or analysis by LCMS after test cleavage. For the analysis the resin was was washed with DMF (5×) and DCM (3×) and dried in vacuum then approx. 10 beads were transferred into an Eppendorf tube and either treated with the reagents for the Kaiser test or with a mixture of TFA/TIS/MPW (95:2.5:2.5; 100 µL) in case of the test cleavage. After incubation for 5 min the test cleavage was diluted with 500 µL of ACN/MPW (1:1) and analysed by LCMS.

Cleavage from resin: Unless otherwise stated the resin was swollen in DCM and treated 10 times with 1% TFA in DCM (5 mL). The resulting cleavage cocktail was passed into prepared *iso*-propanol followed by evaporating the solvent and precipitation in Et_2O. After centrifugation the resulting pellet was separated from the liquid residue and dried in vacuum.

3.1.9 GP-7: Cyclisation of linear peptides

The crud linear peptide was cyclised under pseudo-high dilution conditions (Malesevic et al., 2004) without prior purification after cleavage. A solution of the peptide (1 eq.) in DMF and another solution with HATU (1.3 eq.) and HOAt (1.3 eq.) in DMF was prepared and added from two separate syringes to a solution of HATU (0.1 eq.), HOAt (0.1 eq.) and DIPEA (3 eq.) in DMF. The total DMF volume was chosen for a final peptide concentration of 10 mM. The peptide solution and the coupling reagent solution were added at a flow rate of 1.25 mL/h simultaneously to the stirred solution. After complete addition stirring was continued overnight at room temperature. The solvent was evaporated in vacuum followed by precipitation of the residue in Et_2O. The resulting pellet was dried and purified by normal phase column chromatography (DCM/MeOH).

3.1.10 GP-8: Allyl-deprotection and introduction of linker units to cyclic RGD mimetics

The resin was swollen in DMF (10 mL/g resin) and degassed by bubbling Ar through the suspension for 1 h followed by an addition of $Pd(PPh_3)_4$ (0.1 eq.) and 1,3-dimethylbarbituric acid (DMBA, 4 eq.). After 30 min shaking under inert conditions the cleavage cocktail was removed and the cleavage was repeated for further 30 min with fresh reagents. The resin was washed with DMF (5×10 mL/g resin), DCM (2×10 mL/g resin) and DMF (2×10 mL/g resin) followed by coupling of linker **23** (2 eq.) with oxyma (4 eq.) and DIC (4 eq.) corresponding to **GP-6**.

3.1.11 GP-9: "Click-reaction" between conjugable RGD mimetics and linker-MMAE conjugates to final SMDCs

Compounds: **16**, **17**, **18**

The corresponding conjugable RGD or RAD mimetic (2.2-2.3 eq.) was dissolved in a cleavage cocktail of TFA/MPW/TIS (1400 µL, 95:2.5:2.5) and stirred overnight at room temperature. Afterwards the solvent was co-evaporated with toluene and dried in vacuum. The resulting residue was combined with linker-MMAE conjugate **13** (1 eq.) and sodium ascorbate (4.6-4.7 eq.) as well as dissolved in DMF (1500 µL) and MPW (200 µL). This solution was degassed by several freeze-pump-thraw cycles and frozen in the end. Under inert conditions (Ar-atmosphere) $CuSO_4 \cdot 5\ H_2O$ (2.1-2.6 eq.) was added to the frozen degassed reaction mixture followed by evacuation of the reaction vessel. The reaction mixture was allowed to warm up to room temperature and was stirred overnight, while the reaction progress was monitored by LCMS. When the consumption of linker-MMAE **13** was complete the solution was frozen again and $Pd(PPh_3)_4$ (0.4-0.5 eq.) as well as morpholine (4 eq.) was transferred into the reaction tube. At room temperature the reaction was melted and stirred for 2 h. After complete allyl deprotection the reaction mixture was centrifugated and immediately purified by preparative RP-HPLC. The desired compound was obtained as a colourless solid.

3.2 RGD-Mimetic Precursors

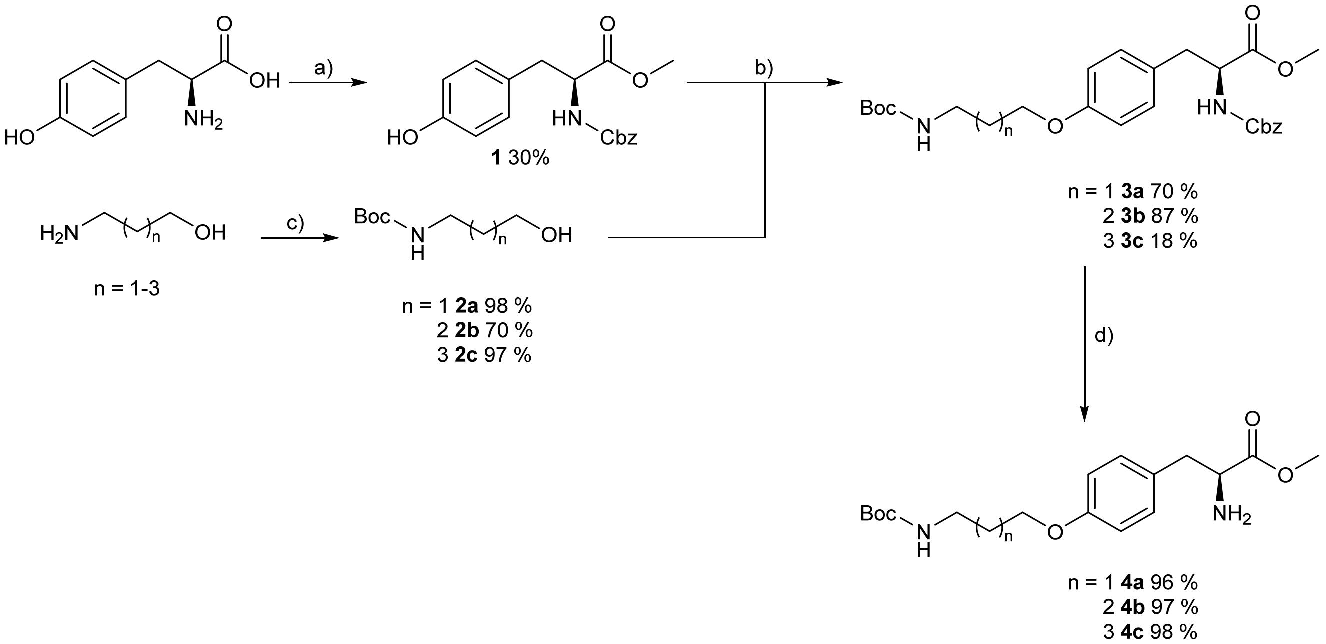

Figure S7: Synthesis of RGD mimetic precursors **4a-c**. Reagents and conditions: a) 1. $SOCl_2$, MeOH, reflux; 2. Cbz-Cl, K_2CO_3, acetone, water, 0 °C->RT; b) DIAD, PPh_3, THF, 0 °C->RT, o.n.; c) Boc_2O, NEt_3, RT, o.n.; d) $Pd(OH)_2/C$, H_2, MeOH/H_2O 3:1, RT, o.n..

3.2.1 Cbz-Tyr-OMe (1)

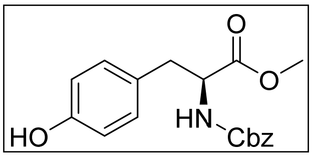

L-Tyrosine (16.545 g, 91.3 mmol, 1 eq.) was suspended in 150 mL MeOH and thionyl chlorid (8.8 mL, 121.3 mmol, 1.3 eq) was added dropwise while stirring vigorously. The clear solution was than refluxed for 2.5 h and stirred at room temperature for further 3 h followed by evaporating the solvent. The resulting solid was dissolved in 100 mL of water and 100 mL acetone. Before adding Cbz-Cl (13.0 mL, 101.3 mmol, 1.1 eq.) under Ar-atmosphere K_2CO_3 (18.953 g, 137.1 mmol, 1.5 eq.) was added and the solution was cooled to 0 °C with an ice bath. After stirring overnight, the solution was cooled again to 0 °C and one more portion of K_2CO_3 (18.5697 g, 134.3 mmol, 1.5 eq.) and Cbz-Cl (7.0 mL, 54.6 mmol, 0.6 eq.) were added. The resulting suspension was stirred at room temperature for 3.5 h and afterwards diluted with 100 mL of water as well as extracted with Ethyl acetate (4×100 mL). The combined organic layers were washed with 1 M HCl (aq., 100 mL) and sat. NaCl (aq., 100 mL). After drying with $MgSO_4$ the solvent was evaporated under reduced pressure and the crude product was purified by automatic column chromatography (MPLC, gradient of petroleum ether and ethyl acetate). Cbz-Tyr-OMe (**1**, 9.077 g, 27.6 mmol, 30 %) was obtained as a white foam.

^{1}H-NMR (600 MHz, Methanol-d_4) δ [ppm] = 7.38 – 7.29 (m, 5H, Cbz-**H**), 6.93 (d, ^3J = 8.1 Hz, 2H, Tyr-2/2′-**H**), 6.70 (d, ^3J = 8.0 Hz, 2H, Tyr-3/3′-**H**), 5.11 (d, ^2J = 12.3 Hz, 1H, Cbz-C**H**$_2$-), 5.08 (d,

^{2}J = 12.4 Hz, 1H, Cbz-C**H**$_2$-), 4.66 – 4.58 (m, 1H, Tyr$^{\alpha}$-**H**), 3.72 (s, 3H, -C**H**$_3$), 3.06 (dd, ^{2}J = 14.0 Hz, ^{3}J = 5.6 Hz, 1H, Tyr$^{\beta}$-**H**/H′), 2.99 (dd, ^{3}J = 14.1 Hz, ^{3}J = 6.2 Hz, 1H, Tyr$^{\beta}$-H/**H**′).

^{13}C{^{1}H}-NMR (151 MHz, Methanol-d_4) δ [ppm] = 172.4 (-**C**OOMe), 155.9 (Cbz-**C**ONH-), 155.2 (Tyr-**C**4), 136.3 (Cbz-**C**1), 130.5 (Tyr-**C**2/2′), 128.7 (Cbz-**C**3/3′), 128.4 (Cbz-**C**4), 128.2 (Cbz-**C**2/2′), 127.4 (Tyr-**C**1), 115.7 (Tyr-**C**3/3′), 67.2 (Cbz-**C**H$_2$-), 55.1 (Tyr-**C**$^{\alpha}$), 52.5 (-**C**H$_3$), 37.6 (Tyr-**C**$^{\beta}$).

R$_f$ (petroleum ether/ethyl acetat; 2:1) = 0.4

3.2.2 *N*-Boc-3-aminopropanol (**2a**)

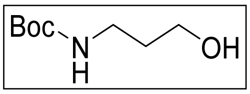

Synthesis according to **GP-1**: Prepared from 3-aminopropanol (5.0 mL, 65.4 mmol, 1 eq.), Boc-anhydride (17.338 g, 79.4 mmol, 1.2 eq.) and triethylamine (18.0 mL, 129.9 mmol, 2 eq.) in water (125 mL) and dioxane (125 mL).

N-Boc-3-aminopropanol (**2a**, 11.222 g, 64.0 mmol, 98 %) was obtained as a high viscous liquid.

^{1}H-NMR (600 MHz, Methanol-d_4) δ [ppm] = 4.75 (s, 1H, -O**H**), 3.66 (t, ^{3}J = 5.7 Hz, 2H, -C**H**$_2$-OH), 3.34 – 3.23 (m, 2H, BocNH-C**H**$_2$-), 1.66 (tt, ^{3}J = 5.9 Hz, ^{3}J = 5.7 Hz, 2H, BocNH-CH$_2$-C**H**$_2$-), 1.45 (s, 9H, Boc-**H**).

R$_f$ (DCM/MeOH; 95:5) = 0.38 ($KMnO_4$-active)

3.2.3 *N*-Boc-4-aminobutanol (**2b**)

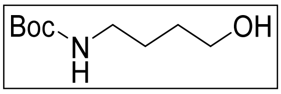

Synthesis according to **GP-1**: Prepared from 4-aminobutanol (1.3 mL, 14.1 mmol, 1 eq.), Boc-anhydride (3.701 g, 17.0 mmol, 1.2 eq.) and triethylamine (3.9 mL, 28.1 mmol, 2 eq.) in water (32 mL) and dioxane (32 mL).

N-Boc-3-aminopropanol (**2b**, 1.871 g, 9.9 mmol, 70 %) was obtained as a high viscous liquid.

^{1}H-NMR (600 MHz, Methanol-d_4) δ [ppm] = 4.64 (s, 1H, -O**H**), 3.66 (t, ^{3}J = 5.9 Hz, 2H, -C**H**$_2$-OH), 3.20 – 3.08 (m, 2H, BocNH-C**H**$_2$-), 1.65 – 1.49 (m, 4H, BocNH-CH$_2$-C**H**$_2$-C**H**$_2$-), 1.43 (s, 9H, Boc-**H**).

R$_f$ (DCM/MeOH; 95:5) = 0.34 ($KMnO_4$-active)

3.2.4 *N*-Boc-3-aminopentanol (**2c**)

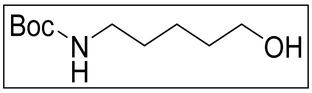

Synthesis according to **GP-1**: Prepared from 5-aminopentanol (2.8 mL, 20.3 mmol, 1 eq.), Boc-anhydride (5.324 g, 24.4 mmol, 1.2 eq.) and triethylamine (5.5 mL, 40.6 mmol, 2 eq.) in water (45 mL) and dioxane (45 mL).

N-Boc-3-aminopropanol (**2c**, 4.005 g, 19.6 mmol, 97 %) was obtained as a high viscous liquid.

^{1}H-NMR (600 MHz, Methanol-d_4) δ [ppm] = 4.58 (s, 1H, -O**H**), 3.63 (t, ^3J = 6.5 Hz, 2H, -C**H**$_2$-OH), 3.16 – 3.03 (m, 2H, BocNH-C**H**$_2$-), 1.62 – 1.52 (m, 2H, -C**H**$_2$-CH$_2$-OH), 1.53 – 1.44 (m, 2H, BocNH-CH$_2$-C**H**$_2$-), 1.46 – 1.34 (m, 11H, Boc-**H**, BocNH-CH$_2$-CH$_2$-C**H**$_2$-).

R$_f$ (DCM/MeOH; 95:5) = 0.42 (KMnO$_4$-active)

3.2.5 Methyl (*S*)-2-(((Benzyloxy)carbonyl)amino)-3-(4-(3-((tert-butoxycarbonyl)amino)-propoxy)phenyl)propanoate (**3a**)

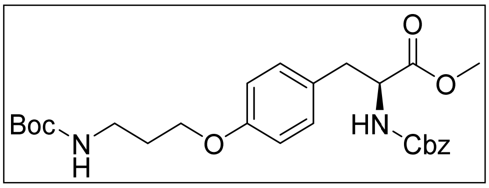

Synthesis according to **GP-2**: Prepared from **1** (6.077 g, 18.5 mmol, 1.1 eq.), **2a** (2.968 g, 16.9 mmol, 1 eq.), TPP (5.349 g, 20.4 mmol, 1.2 eq.) and DIAD (4.00 mL, 20.4 mmol, 1.2 eq.).

The desired compound **3a** (5.778 g, 11.9 mmol, 70 %) was obtained as colourless foam.

^{1}H-NMR (600 MHz, Methanol-d_4) δ [ppm] = 7.37 – 7.23 (m, 5H, Cbz-**H**), 7.10 (d, ^3J = 8.1 Hz, 2H, Tyr-2/2′-**H**), 6.83 (d, ^3J = 8.2 Hz, 2H, Tyr-3/3′-**H**), 5.06 (d, ^2J = 12.7 Hz, 1H, Cbz-C**H**$_2$-), 5.02 (d, ^2J = 12.8 Hz, 1H, Cbz-C**H**$_2$-), 4.40 (dd, ^3J = 9.2 Hz, ^3J = 5.6 Hz, 1H, Tyr^α-**H**), 3.98 (t, ^3J = 6.1 Hz, 2H, -C**H**$_2$-O-), 3.70 (s, 3H, -C**H**$_3$) 3.23 (t, ^3J = 6.9 Hz, 2H, BocNH-C**H**$_2$-), 3.08 (dd, ^2J = 14.0 Hz, ^3J = 5.5 Hz, 1H, Tyr^β-**H**/H′), 2.87 (dd, ^3J = 13.9 Hz, ^3J = 9.1 Hz, 1H, Tyr^β-H/**H**′), 1.93 (tt, ^3J = 6.9 Hz, ^3J = 6.1 Hz, 2H, -CH$_2$-C**H**$_2$-CH$_2$-), 1.43 (s, 9H, Boc-**H**).

3.2.6 Methyl (*S*)-2-Amino-3-(4-(3-((tert-butoxycarbonyl)amino)propoxy)phenyl)propanoate (**4a**)

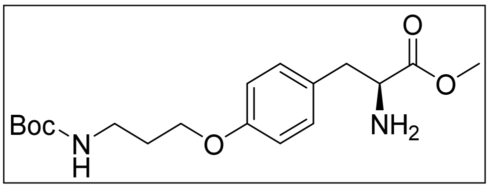

Synthesis according to **GP-3**: Prepared from **3a** (4.505 g, 9.3 mmol, 1 eq.), Pd(OH)$_2$/C (0.129 g, 0.121 mmol, 0.01 eq.) in 50 mL MeOH.

The desired compound **4a** (3.120 g, 8.85 mmol, 96 %) was obtained as a colourless solid.

^{1}H-NMR (600 MHz, Methanol-d_4) δ [ppm] = 7.16 (d, ^3J = 8.5 Hz, 2H, Tyr-2/2′-**H**), 6.93 (d, ^3J = 8.6 Hz, 2H, Tyr-3/3′-**H**), 4.27 (dd, ^3J = 7.4 Hz, 6.0 Hz, 1H, Tyr^α-**H**), 4.01 (t, ^3J = 6.2 Hz, 2H, -C**H**$_2$-O-), 3.82 (s, 3H, -C**H**$_3$), 3.23 (t, ^3J = 6.9 Hz, 2H, BocNH-C**H**$_2$-), 3.19 (dd, ^3J = 14.5 Hz, ^3J = 6.0 Hz, 1H, Tyr^β-**H**/H′), 3.11 (dd, ^2J = 14.5 Hz, ^3J = 7.5 Hz, 1H, Tyr^β-H/**H**′), 1.94 (tt, ^3J = 6.9 Hz, ^3J = 6.2 Hz, 2H, -CH$_2$-C**H**$_2$-CH$_2$-), 1.44 (s, 9H, Boc-**H**).

^{13}C{^{1}H}-NMR (151 MHz, Methanol-d_4) δ [ppm] = 170.5 (-**C**OOMe), 160.2 (^{t}BuO**C**ONH-), 158.6 (Tyr-**C**4), 131.5 (Tyr-**C**2/2′), 127.0 (Tyr-**C**1), 116.2 (Tyr-**C**3/3′), 80.0 (-**C**(CH$_3$)$_3$), 66.7 (-**C**H$_2$-O-), 55.3 (Tyr-**C**^{α}), 53.6 (-**C**H$_3$), 38.4 (BocNH-**C**H$_2$), 36.6 (Tyr-**C**^{β}), 30.8 (-CH$_2$-**C**H$_2$-CH$_2$-), 28.8 (-C(**C**H$_3$)$_3$).

LC-MS (ESI+) m/z: 353.3 [M+H]$^+$, 297.2 [M-^{t}Bu+H]$^+$, 253.2 [M-Boc+H]$^+$; t$_R$ = 5.3min.

3.2.7 Methyl (*S*)-2-(((Benzyloxy)carbonyl)amino)-3-(4-(4-((tert-butoxycarbonyl)amino)-butoxy)phenyl)propanoate (**3b**)

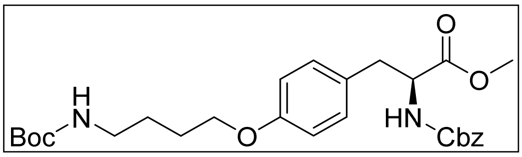

Synthesis according to **GP-2**: Prepared from **1** (6.077 g, 18.5 mmol, 1.1 eq.), **2b** (3.194 g, 16.9 mmol, 1 eq.), TPP (5.317 g, 20,3 mmol, 1.2 eq.) and DIAD (4.00 mL, 20.4 mmol, 1.2 eq.).

The desired compound **3b** (7.340 g, 14.7 mmol, 87 %) was obtained as colourless foam.

^{1}H-NMR (600 MHz, Methanol-d_4) δ [ppm] = 7.39 – 7.24 (m, 5H, Cbz-**H**), 7.10 (d, ^3J = 8.1 Hz, 2H, Tyr-2/2′-**H**), 6.82 (d, ^3J = 8.1 Hz, 2H, Tyr-3/3′-**H**), 5.07 (d, ^2J = 12.7 Hz, 1H, Cbz-C**H**$_2$-), 5.02 (d, ^2J = 12.9 Hz, 1H, Cbz-C**H**$_2$-), 4.40 (dd, ^3J = 9.1 Hz, ^3J = 5.5 Hz, 1H, Tyr^α-**H**), 3.96 (t, ^3J = 6.3 Hz, 2H, -C**H**$_2$-O-), 3.70 (s, 3H, -C**H**$_3$), 3.11 (t, ^3J = 7.1 Hz, 2H, BocNH-C**H**$_2$-), 3.06 (d, ^2J = 14.0 Hz, ^3J = 5.5 Hz, 1H, Tyr^β-**H**/H′), 2.87 (dd, ^2J = 14.0 Hz, ^3J = 9.1 Hz, 1H, Tyr^β-H/**H**′), 1.78 (tt, ^3J = 6.6 Hz, ^3J = 6.3 Hz, 2H, -C**H**$_2$-CH$_2$-O-), 1.65 (tt, ^3J = 7.2 Hz, ^3J = 7.1 Hz, 2H, BocNH-CH$_2$-C**H**$_2$-), 1.44 (s, 9H, Boc-**H**).

LC-MS (ESI+) m/z: 523.2 [M+Na]$^+$, 401.3 [M-Boc+H]$^+$; t$_R$ = 10.3 min.

3.2.8 Methyl (*S*)-2-Amino-3-(4-(4-((tert-butoxycarbonyl)amino)butoxy)phenyl)propanoate (**4b**)

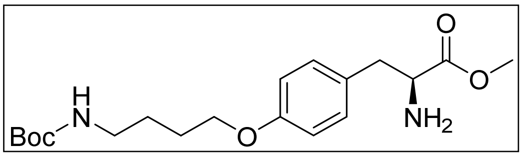

Synthesis according to **GP-3**: Prepared from **1** (6.020 g, 12.03 mmol, 1 eq.), Pd(OH)$_2$/C (0.172 g, 0.161 mmol, 0.01 eq.) in 50 mL MeOH.

The desired compound **4b** (4.276 g, 11.67 mmol, 97 %) was obtained as a colourless solid.

^{1}H-NMR (600 MHz, Methanol-d_4) δ [ppm] = 7.17 (d, ^3J = 8.6 Hz, 2H, Tyr-2/2′-**H**), 6.94 (d, ^3J = 8.6 Hz, 2H, Tyr-3/3′-**H**), 4.28 (dd, ^3J = 7.4 Hz, ^3J = 6.0 Hz, 1H, Tyr^α-**H**), 4.01 (t, ^3J = 6.3 Hz, 2H, -C**H**$_2$-O-), 3.83 (s, 3H, -C**H**$_3$), 3.21 (dd, ^2J = 14.5 Hz, ^3J = 6.0 Hz, 1H, Tyr^β-**H**/H′), 3.12 (t, ^3J = 7.0 Hz, 2H, BocNH-C**H**$_2$-), 3.12 (dd, ^2J = 14.5 Hz, ^3J = 7.4 Hz, 1H, Tyr^β-H/**H**′), 1.86 – 1.76 (m, 2H, -C**H**$_2$-CH$_2$-O-), 1.71 – 1.61 (m, 2H, BocNH-CH$_2$-C**H**$_2$-), 1.46 (s, 9H, Boc-**H**).

$^{13}C\{^{1}H\}$-NMR (151 MHz, Methanol-d_4) δ [ppm] = 170.5 (-**C**OOMe), 160.3 (^{t}BuO**C**ONH-), 158.6 (Tyr-**C**4), 131.5 (Tyr-**C**2/2′), 126.9 (Tyr-**C**1),), 116.2 (Tyr-**C**3/3′), 79.9 (-**C**(CH_3)$_3$), 68.7 (-**C**H_2-O-), 55.3 (Tyr-**C**$^{\alpha}$), 53.6 (-**C**H_3), 41.0 (BocNH-**C**H_2), 36.6 (Tyr-**C**$^{\beta}$), 28.78 (-C(**C**H_3)$_3$), 27.7 (BocNH-CH_2-**C**H_2-), 27.6 (-**C**H_2-CH_2-O-).

LC-MS (ESI+) m/z: 367.2 $[M+H]^+$, 311.2 $[M\text{-}^{t}Bu+H]^+$, 267.2 $[M\text{-}Boc+H]^+$; t_R = 5.5min.

3.2.9 Methyl (*S*)-2-(((Benzyloxy)carbonyl)amino)-3-(4-((5-((tert-butoxycarbonyl)amino)-pentyl)oxy)phenyl)propanoate (3c)

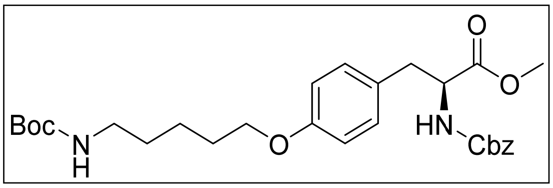

Synthesis according to **GP-2**: Prepared from **1** (6.077 g, 18.5 mmol, 1.1 eq.), **2c** (3.479 g, 17.1 mmol, 1 eq.), TPP (5.459 g, 20.8 mmol, 1.2 eq.) and DIAD (4.00 mL, 20.4 mmol, 1.2 eq.).

The desired compound **3c** (1.605 g, 3.1 mmol, 18 %) was obtained as colourless foam.

^{1}H-NMR (600 MHz, Methanol-d_4) δ [ppm] = 7.37 – 7.28 (m, 5H, Cbz-**H**), 7.11 (d, ^{3}J = 8.3 Hz, 2H, Tyr-2/2′-**H**), 6.83 (d, ^{3}J = 8.6 Hz, 2H, Tyr-3/3′-**H**), 5.08 (d, ^{2}J = 12.6 Hz, 1H, Cbz-C**H**$_2$-), 5.03 (d, ^{2}J = 12.7 Hz, 1H, Cbz-C**H**$_2$-), 4.42 (dd, ^{3}J = 9.0 Hz, ^{3}J = 5.6 Hz, 1H, Tyr$^{\alpha}$-**H**), 3.95 (t, ^{3}J = 6.4 Hz, 2H, -C**H**$_2$-O-), 3.71 (s, 3H, -C**H**$_3$), 3.11 – 3.06 (m, 3H, BocNH-C**H**$_2$-, Tyr$^{\beta}$-**H**/H′), 2.89 (dd, ^{2}J = 13.9 Hz, ^{3}J = 9.1 Hz, 1H, Tyr$^{\beta}$-H/**H**′), 1.89 – 1.69 (m, 2H, BocNH-CH_2-C**H**$_2$-), 1.65 – 1.48 (m, 4H, -C**H**$_2$-C**H**$_2$-CH_2-O-), 1.46 (s, 9H, Boc-**H**).

$^{13}C\{^{1}H\}$-NMR (151 MHz, Methanol-d_4) δ [ppm] = 174.0 (-**C**OOMe), 159.5 (Tyr-**C**4), 158.6 (^{t}BuO**C**ONH-), 158.3 (BnO**C**ONH-), 138.2 (Ar-**C**1), 131.2 (Tyr-**C**2/2′), 130.0 (Tyr-**C**1), 129.4 (Ar-**C**3/3′), 128.9 (Ar-**C**4′), 128.6 (Ar-**C**2/2′), 115.5 (Tyr-**C**3/3′), 79.8 (-**C**(CH_3)$_3$), 68.8 (-**C**H_2-O-), 67.5 (Cbz-**C**H_2-), 57.2 (Tyr-**C**$^{\alpha}$), 52.6 (-**C**H_3), 41.3 (BocNH-**C**H_2), 37.8 (Tyr-**C**$^{\beta}$), 30.7 (-**C**H_2-CH_2-O-), 30.1 (BocNH-CH_2-**C**H_2-), 28.8 (-C(**C**H_3)$_3$), 24.4 (BocNH-CH_2-CH_2-**C**H_2-).

LC-MS (ESI+) m/z: 537.2 $[M+Na]^+$, 415.2 $[M\text{-}Boc+H]^+$; t_R = 10.6 min.

3.2.10 Methyl (*S*)-2-Amino-3-(4-((5-((tert-butoxycarbonyl)amino)pentyl)oxy)phenyl)-propanoate (4c)

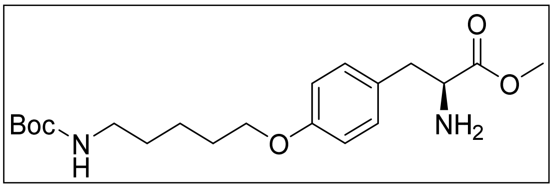

Synthesis according to **GP-3**: Prepared from **3c** (1.605 g, 3.11 mmol, 1 eq.), $Pd(OH)_2/C$ (0.043 g, 0.040 mmol, 0.01 eq.) in 18 mL MeOH.

The desired compound **4c** (0.534 g, 3.06 mmol, 98 %) was obtained as a colourless solid.

^{1}H-NMR (600 MHz, Methanol-d_4) δ [ppm] = 7.15 (d, ^{3}J = 8.5 Hz, 2H, Tyr-2/2′-**H**), 6.92 (d, ^{3}J = 8.6 Hz, 2H, Tyr-3/3′-**H**), 4.26 (dd, ^{3}J = 7.4 Hz, ^{3}J = 6.0 Hz, 1H, Tyr$^{\alpha}$-**H**), 3.98 (t, ^{3}J = 6.4 Hz, 2H, -C**H**$_2$-O-), 3.82 (s, 3H, -C**H**$_3$), 3.20 (dd, ^{2}J = 14.5 Hz, ^{3}J = 5.9 Hz, 1H, Tyr$^{\beta}$-**H**/H′), 3.11 (dd, ^{2}J = 14.5 Hz, ^{3}J = 7.4 Hz, 1H, Tyr$^{\beta}$-H/**H**′), 3.06 (t, ^{3}J = 6.8 Hz, 2H, BocNH-C**H**$_2$-), 1.84 – 1.75 (m, 2H, BocNH-CH$_2$-C**H**$_2$-), 1.57 – 1.47 (m, 4H, -C**H**$_2$-C**H**$_2$-CH$_2$-O-), 1.44 (s, 9H, Boc-**H**).

^{13}C{^{1}H}-NMR (151 MHz, Methanol-d_4) δ [ppm] = 170.5 (-**C**OOMe), 160.3 (^{t}BuO**C**ONH-), 158.6 (Tyr-**C**4), 131.5 (Tyr-**C**2/2′), 126.8 (Tyr-**C**1), 116.1 (Tyr-**C**3/3′), 79.8 (-**C**(CH$_3$)$_3$), 68.9 (-**C**H$_2$-O-), 55.3 (Tyr-**C**$^{\alpha}$), 53.6 (-**C**H$_3$), 41.2 (BocNH-**C**H$_2$), 36.6 (Tyr-**C**$^{\beta}$), 30.7 (-**C**H$_2$-CH$_2$-O-), 30.0 (BocNH-CH$_2$-**C**H$_2$-), 28.8 (-C(**C**H$_3$)$_3$), 24.4 (BocNH-CH$_2$-CH$_2$-**C**H$_2$-).

LC-MS (ESI+) m/z: 381.3 [M+H]$^+$, 325.3 [M-^{t}Bu+H]$^+$, 281.2 [M-Boc+H]$^+$; t_R = 6.2 min.

3.3 Benzoic Acid-PEG-Linker

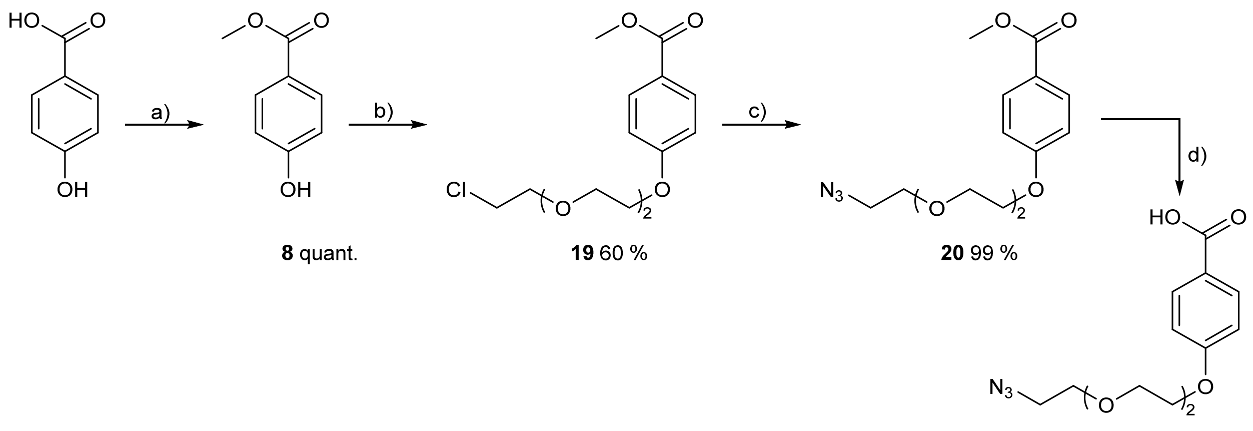

8 quant. **19** 60 % **20** 99 % **9** 90 %

Figure S8: Synthesis of the benzoic acid-based linker **9**. Reagents and conditions: a) $SOCl_2$, MeOH, 0 -> RT -> reflux, o.n; b) PEG-3, DIAD, PPh_3, THF, 0 °C->RT, o.n; c) NaN_3, water, DMF, reflux, o.n; d) LiOH, water, MeOH, DMF, 0 °C -> RT, o.n..

3.3.1 Methyl 4-Hydroxybenzoate (**8**)

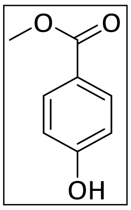

4-Hydroxybenzoic acid (4.151 g, 30.1 mmol, 1 eq.) was dissolved in MeOH (30 mL) and cooled to 0 °C with an ice bath. While cooling and intense stirring thionyl chloride (4.2 mL, 57.7 mmol, 1.9 eq.) was dropwise added in 15 min. After the addition the reaction mixture was stirred overnight while the cooling bath come up to room temperature. Another admission of thionyl chloride (0.5 mL, 6.9 mmol, 0.2 eq.) followed as well as stirring for further 6 h. After complete conversion the solvent was removed under reduced pressure and the product was dried in vacuum. Methyl 4-hydroxybenzoate (**8**, 4.576 g, 30.1 mmol, quant.) was obtained as a light-yellow solid.

^{1}H-NMR (600 MHz, Methanol-d_4) δ [ppm] = 10.33 (s, 1H, Ph-O**H**), 7.81 (d, ^3J = 8.2 Hz, 2H, Ph-2/2´-**H**), 6.85 (d, ^3J = 8.2 Hz, 2H, Ph-3/3´-**H**), 3.78 (s, 3H, -C**H**$_3$).

^{13}C{^{1}H}-NMR (151 MHz, Methanol-d_4) δ [ppm] = 166.0 (Ph-**C**O$_2$-CH$_3$), 161.9 (Ph-**C**-4), 131.4 (Ph-**C**-2/2´), 120.2 (Ph-**C**-1), 115.3 (Ph-**C**-3/3´), 51.6 (-**C**H$_3$).

3.3.2 Methyl 4-(2-(2-(2-Chloroethoxy)ethoxy)ethoxy)benzoate (**19**)

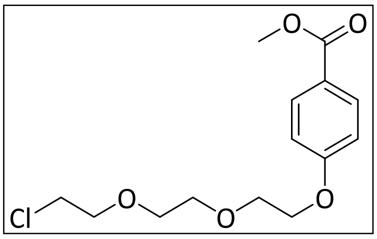

Synthesis according to **GP-2**: Prepared from **8** (1.2635 g, 8.3 mmol, 1 eq.), 2-(2-(2-chloroethoxy)ethoxy)ethan-1-ol (2.2 mL, 15.1 mmol, 1.8 eq.), TPP (2.596 g, 9.9 mmol, 1.2 eq.) and DIAD (1.3 mL, 9.7 mmol, 1.2 eq.). The desired compound **19** (1.500 g, 5.0 mmol, 60 %) was obtained.

^{1}H-NMR (600 MHz, Methanol-d_4) δ [ppm] = 7.98 (d, ^3J = 7.6 Hz, 2H, Ph-2/2´-**H**), 6.93 (d, ^3J = 7.7 Hz, 2H, Ph-3/3´-**H**), 4.19 (t, ^3J = 4.2 Hz, 2H, PEG-C**H**$_2$-O-Ph), 3.92-3.89 (m, 2H, PEG-C**H**$_2$-CH$_2$-O-Ph), 3.88 (s, 3H, -C**H**$_3$), 3.79-3.72 (m, 4H, PEG-**H**), 3.72-3.69 (m, 2H, PEG-**H**), 3.63 (t, ^3J = 5.8 Hz, 2H, Cl-C**H**$_2$-PEG).

^{13}C{^{1}H}-NMR (151 MHz, Methanol-d_4) δ [ppm] = 167.0 (Ph-**C**O$_2$-CH$_3$), 162.7 (Ph-**C**-4), 131.7 (Ph-**C**-2/2´), 122.9 (Ph-**C**-1), 114.3 (Ph-**C**-3/3´), 71.6 (Cl-CH$_2$-C**H**$_2$-PEG), 71.0 (PEG-**C**), 70.9 (PEG-**C**), 69.8 (PEG-**C**H$_2$-CH$_2$-O-Ph), 67.7 (PEG-CH$_2$-**C**H$_2$-O-Ph), 52.0 (-**C**H$_3$), 42.9 (Cl-**C**H$_2$-CH$_2$-PEG).

LC-MS (ESI+) m/z: 303.1 [M+H]$^+$; t_R = 9.1 min.

R$_f$ (PE/EtOAc; 5:1) = 0.35

3.3.3 Methyl 4-(2-(2-(2-Azidoethoxy)ethoxy)ethoxy)benzoate (**20**)

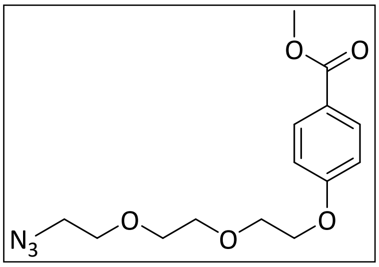

Chloride **19** (0.753 g, 2.5 mol, 1 eq.) and NaN$_3$ (0.447 g, 6.9 mmol, 2.8 eq.) were dissolved in water (25 mL) and DMF (20 mL). The reaction mixture was stirred and heated overnight to 80 °C and 1 d at room temperature. Afterwards the mixture was diluted with water and extracted with DCM (3×). The combined organic layers were washed with water (1×) and sat. NaCl (aq., 1×) as well as dried with MgSO$_4$ followed by removing the solvent under reduced pressure. The product was dried in vacuum to obtain **9** (0.765 g, 2.5 mmol, 99 %) as a light-yellow viscous liquid.

^{1}H-NMR (600 MHz, Methanol-d_4) δ [ppm] = 7.98 (d, ^3J = 8.3 Hz, 2H, Ph-2/2´-**H**), 6.93 (d, ^3J = 8.2 Hz, 2H, Ph-3/3´-**H**), 4.22 – 4.16 (m, 2H, PEG-C**H**$_2$-O-Ph), 3.91 – 3.89 (m, 2H, PEG-C**H**$_2$-CH$_2$-O-Ph), 3.88

(s, 3H, -C**H**$_3$), 3.78 – 3.72 (m, 2H, PEG-**H**), 3.72 – 3.65 (m, 4H, PEG-**H**), 3.42 – 3.35 (m, 2H, N_3-C**H**$_2$-PEG).

^{13}C{^{1}H}-NMR (151 MHz, Methanol-*d*$_4$) δ [ppm] = 167.0 (Ph-**C**O$_2$-CH$_3$), 162.7 (Ph-**C**-4), 131.7 (Ph-**C**-2/2´), 122.9 (Ph-**C**-1), 114.3 (Ph-**C**-3/3´), 71.1 (PEG-**C**), 70.9 (PEG-**C**), 70.3 (N_3-CH$_2$-C**H**$_2$-PEG), 69.8 (PEG-**C**H$_2$-CH$_2$-O-Ph), 67.7 (PEG-CH$_2$-**C**H$_2$-O-Ph), 52.0 (-**C**H$_3$), 50.8 (N_3-C**H**$_2$-CH$_2$-PEG).

LC-MS (ESI+) m/z: 332.1 [M+Na]$^+$; 310.2 [M+H]$^+$; t_R = 9.1 min.

3.3.4 4-(2-(2-(2-Azidoethoxy)ethoxy)ethoxy)benzoic acid (**9**)

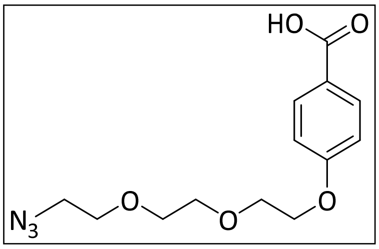

Ester **20** (0.765 g, 2.5 mmol, 1 eq.) was cooled with an ice bath to 0 °C followed by addition of LiOH (1M in THF/MeOH/water (3:1:1), 8 mL, 8.0 mmol, 3.2 eq.). The reaction solution was stirred during cooling for 40 min and then at room temperature overnight. Afterwards the solvent was reduced, and the residue was diluted with water and acidified with 1 M HCl (aq.) followed by extraction with DCM (3x). The combined organic layers were washed with sat. NaCl (aq.) and dried with $MgSO_4$. After removing the solvent under reduced pressure and drying in vacuum **9** (0.654 g, 2.2 mmol, 90 %) was obtained as a colourless solid.

^{1}H-NMR (600 MHz, Methanol-*d*$_4$) δ [ppm] = 8.05 (d, ^3J = 8.1 Hz, 2H, Ph-2/2´-**H**), 6.96 (d, ^3J = 8.2 Hz, 2H, Ph-3/3´-**H**), 4.21 (s, 2H, PEG-C**H**$_2$-O-Ph), 3.93-3.87 (m, 2H, PEG-C**H**$_2$-CH$_2$-O-Ph), 3.75 (s, 2H, PEG-**H**), 3.73-3.66 (m, 4H, PEG-**H**), 3.42-3.36 (m, 2H, N_3-C**H**$_2$-CH$_2$-PEG).

^{13}C{^{1}H}-NMR (151 MHz, Methanol-*d*$_4$) δ [ppm] = 171.1 (Ph-**C**O$_2$H), 163.4 (Ph-**C**-4), 132.5 (Ph-**C**-2/2´), 121.9 (Ph-**C**-1), 114.5 (Ph-**C**-3/3´), 71.1 (PEG-**C**), 70.9 (PEG-**C**), 70.3 (N_3-CH$_2$-C**H**$_2$-PEG), 69.8 (PEG-**C**H$_2$-CH$_2$-O-Ph), 67.8 (PEG-CH$_2$-**C**H$_2$-O-Ph), 50.8 (N_3-C**H**$_2$-CH$_2$-PEG)

LC-MS (ESI+) m/z: 318.1 [M+Na]$^+$, 296.1 [M+H]$^+$; t_R = 7.7 min.

3.4 Succinidyl-PEG-Linker

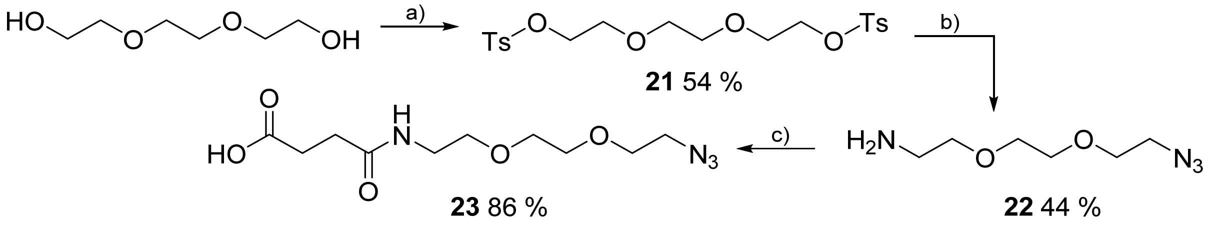

Figure S9: Synthesis of aliphatic linker **23**. Reagents and conditions: a) Ts-Cl, Net_3, 1-methylimidazole, DCM, 0 °C -> RT, o.n.; b) 1. NaN_3, acetone, water, reflux, o.n.; 2. PPh_3, toluene, water, EtOH, RT, o.n.; c) succinic anhydride, acetonitrile, RT, o.n..

3.4.1 (Ethane-1,2-diylbis(oxy))bis(ethane-2,1-diyl) Bis(4-methylbenzenesulfonate) (**21**)

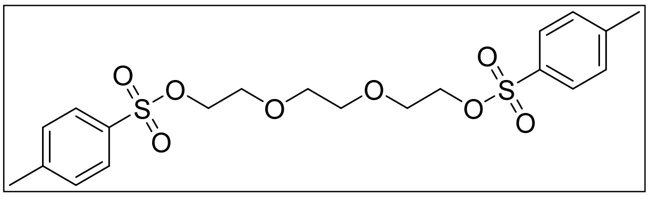

2,2'-(ethane-1,2-diylbis(oxy)) Bis(ethan-1-ol) (16.701 g, 111.2 mmol, 1 eq.) in a flask was cooled to 0 °C with an ice bath followed by addition of NEt_3 (32.5 mL, 233.0 mmol, 2.7 eq.) and 1-methylimidazole (14.0 µL, 176 µmol, 0.002 eq.). 4-Toluenesulfonyl chloride (44.746 g, 234.7 mmol, 2.7 eq.) was suspended in DCM (170 mL) and added portion wise to the cooled PEG solution over 1 h. After complete addition the cooling bath was removed, and the reaction mixture was stirred overnight. The organic layer was washed with 1 M HCl (aq., 200 mL) and sat. $NaHCO_3$ (aq., 200 mL) followed by drying with $MgSO_4$. Afterwards solvent was removed, and the crude product was purified by column chromatography (DCM->DCM/MeOH, 95:5) so that **21** (27.539 g, 60.1 mmol, 54 %) was obtained as a colourless solid.

^{1}H-NMR (600 MHz, Methanol-d_4) δ [ppm] = 7.79 (d, ^3J = 8.3 Hz, 4H, Ar-2/2′-**H**), 7.34 (d, ^3J = 8.0 Hz, 4H, Ar-3/3′-**H**), 4.13 (d, ^3J = 4.8 Hz, 4H, PEG-**H**), 3.65 (t, ^3J = 4.8 Hz, 4H, PEG-**H**), 3.52 (s, 4H, PEG-**H**), 2.44 (s, 6H, Ar-C**H**$_3$).

^{13}C{^{1}H}-NMR (151 MHz, Methanol-d_4) δ [ppm] = 145.0 (Ar-**C**-1), 133.1 (Ar-**C**-4), 130. (Ar-**C**-3/3′), 128.1 (Ar-**C**-2/2′), 70.8 (PEG-**C**), 69.3 (PEG-**C**), 68.9 (PEG-**C**), 21.8 (Ar-**C**H$_3$).

LC-MS (ESI+) m/z: 481.1 $[M+Na]^+$, 459.1 $[M+H]^+$; t_R = 9.8 min.

3.4.2 2-(2-(2-Azidoethoxy)ethoxy)ethan-1-amine (**22**)

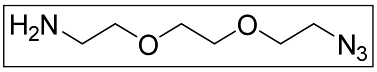

21 (27.539 g, 30.1 mmol, 1 eq.) was dissolved in acetone (200 mL) and water (70 mL) together with sodium azide (39.037 g, 600 mmol, 10 eq.). Afterwards the reaction was refluxed overnight and stirred for 1 d at room temperature. The reaction mixture was diluted with water and extracted with DCM (3×) followed by evaporating the solvent under reduced pressure and dissolving the residue in toluene (94 mL) as well as additionally 5 %

HCl (aq., 144 mL) and EtOH (28.5 mL). To this two-layer system TPP (15.764 g, 60.1 mmol, 1 eq.) in toluene (284 ml) was added dropwise in 1 h. The reaction was stirred overnight at room temperature. Thereafter 1 M HCl (aq., 100 mL) was added, and the layers were separated. The water layer was washed twice with Et_2O (100 mL) and then 10 % KOH (aq.) was added to reach pH 12 followed by an extraction with DCM (3×150 mL). The resulting organic layers were combined and dried with $MgSO_4$. After evaporating the solvent under reduced pressure und drying in vacuum the desired product **22** (4.646 g, 26.7 mmol, 44 %) was obtained.

^{1}H-NMR (600 MHz, Methanol-d_4) δ [ppm] = 3.69 – 3.62 (m, 6H, PEG-**H**), 3.52 (t, ^3J = 5.2 Hz, 2H, PEG-**H**), 3.39 (t, ^3J = 5.1 Hz, 2H, PEG-**H**), 2.87 (t, ^3J = 5.2 Hz, 2H, PEG-**H**), 1.80 – 1.59 (m, 2H, -N**H**$_2$).

3.4.3 4-((2-(2-(2-Azidoethoxy)ethoxy)ethyl)amino)-4-oxobutanoic acid (23)

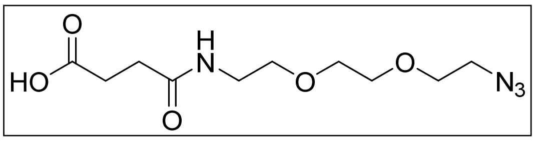

To solution of Amine **22** (3.001 g, 17.2 mmol, 1 eq.) in acetonitrile (175 mL) was added dropwise a suspension of succinic anhydride (2.807 g, 28.0 mmol, 1.6 eq.) in acetonitrile (175 mL) in 1 h. The reaction mixture was stirred overnight at room temperature followed by evaporating the solvent and purification of the crude product *via* automatically column chromatography (MPLC, DCM/MeOH) so that product **23** (4.048 g, 14.8 mmol, 86 %) could be isolated.

^{1}H-NMR (600 MHz, Methanol-d_4) δ [ppm] = 3.73 – 3.61 (m, 6H, PEG-**H**), 3.57 (t, ^3J = 4.8 Hz, 2H, PEG-**H**), 3.50 – 3.44 (m, 2H, -CONH-C**H**$_2$-), 3.41 (t, ^3J = 4.9 Hz, 2H, -C**H**$_2$-N$_3$), 2.69 (t, ^3J = 6.2 Hz, 2H, -C**H**$_2$-CONH-), 2.53 (t, ^3J = 6.2 Hz, 2H, -C**H**$_2$-COOH).

^{13}C{^{1}H}-NMR (151 MHz, Methanol-d_4) δ [ppm] = 175.5 (-**C**OOH), 172.7 (-**C**ONH-), 70.7 (PEG-**C**), 70.3 (PEG-**C**), 70.3 (PEG-**C**), 69.8 (PEG-**C**), 50.8 (-**C**H$_2$-N$_3$), 39.7 (-CONH-**C**H$_2$-), 31.0 (-**C**H$_2$-COOH), 30.2 (-**C**H$_2$-CONH-).

3.5 RGD Mimetic Precursors

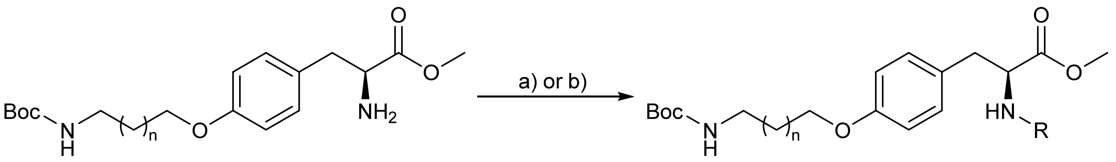

n = 1 **4a**
2 **4b**
3 **4c**

Table 2: **5a-c**
6a-c
7a-c

Figure S10: Synthesis of RGD mimetic precursors **5a-c**, **6a-c** and **7a-c**. Reagents and conditions: a) benzoate, HOBt, EDC, DIPEA, DMF, DCM, RT, o.n.; b) benzoic acid chloride, DIPEA, DMC, DMF, RT, o.n..

3.5.1 Methyl (*S*)-2-Benzamido-3-(4-(3-((tert-butoxycarbonyl)amino)propoxy)phenyl)-propanoate (**5a**)

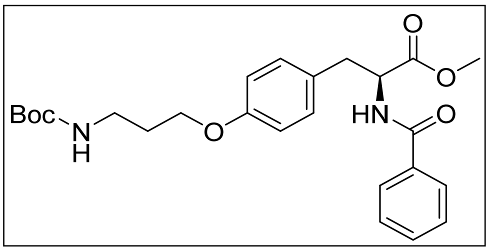

Synthesis according to **GP-4a**: Prepared from (**4a**).

The RGD mimetic precursor (**5a**) (50.6 mg, 110.8 µmol, 39 %) was isolated as a colourless solid.

^{1}H-NMR (600 MHz, Methanol-d_4) δ [ppm] = 7.75 (d, ^3J = 7.0 Hz, 2H, Ar-2/2′-**H**), 7.54 (t, ^3J = 7.4 Hz, 1H, Ar-4-**H**), 7.45 (dd, ^3J = 7.8 Hz, ^3J = 7.8 Hz, 2H, Ar-3/3′-**H**), 7.17 (d, ^3J = 8.4 Hz, 2H, Tyr-2/2′-**H**), 6.85 (d, ^3J = 8.6 Hz, 2H, Tyr-3/3′-**H**), 4.81 (dd, ^3J = 9.3 Hz, ^3J = 5.6 Hz, 1H, Tyr$^\alpha$-**H**), 3.97 (t, ^3J = 6.1 Hz, 2H, -C**H**$_2$-O-), 3.74 (s, 3H, -C**H**$_3$), 3.26 – 3.20 (m, 3H, BocNH-C**H**$_2$-, Tyr$^\beta$-**H**/H′), 3.07 (dd, ^2J = 14.0 Hz, ^3J = 9.3 Hz, 1H, Tyr$^\beta$-H/**H′**), 1.91 (tt, ^3J = 6.5 Hz, ^3J = 6.5 Hz, 2H, -CH$_2$-C**H**$_2$-CH$_2$-), 1.43 (s, 9H, Boc-**H**).

^{13}C{^{1}H}-NMR (151 MHz, Methanol-d_4) δ [ppm] = 173.7 (-**C**OOMe), 170.2 (Ar-**C**ONH-), 159.4 (Tyr-**C**4), 158.5 (^{t}BuO**C**ONH-), 135.2 (Ar-**C**1), 132.9 (Ar-**C**4), 131.2 (Tyr-**C**2/2′), 130.4 (Tyr-**C**1), 129.5 (Ar-**C**3/3′), 128.4 (Ar-**C**2/2′), 115.6 (Tyr-**C**3/3′), 79.9 (-**C**(CH$_3$)$_3$), 66.6 (-**C**H$_2$-O-), 56.1 (Tyr-**C**$^\alpha$), 52.8 (-**C**H$_3$), 38.4 (BocNH-**C**H$_2$), 37.4 (Tyr-**C**$^\beta$), 30.7 (-CH$_2$-**C**H$_2$-CH$_2$-), 28.8 (-C(**C**H$_3$)$_3$).

LC-MS (ESI+) m/z: 457.1 [M+H]$^+$, 401.1 [M-^{t}Bu+H]$^+$, 357.1 [M-Boc+H]$^+$; t_R = 9.4min.

3.5.2 Methyl (*S*)-3-(4-(3-((Tert-butoxycarbonyl)amino)propoxy)phenyl)-2-(4-hydroxy-benzamido)propanoate (**5b**)

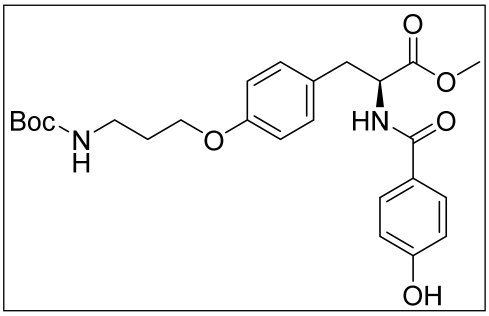

Synthesis according to **GP-4b**: Prepared from (**4a**).

The RGD mimetic precursor (**5b**) (68.4 mg, 144.8 µmol, 51 %) was isolated as a colourless solid.

^{1}H-NMR (600 MHz, Methanol-*d*$_4$) δ [ppm] = 7.65 (d, ^3J = 8.7 Hz, 2H, Ar-2/2´-**H**), 7.15 (d, ^3J = 8.5 Hz, 2H, Tyr-2/2´-**H**), 6.84 (d, ^3J = 8.6 Hz, 2H, Tyr-3/3´-**H**), 6.81 (d, ^3J = 8.7 Hz, 2H, Ar-3/3´-**H**), 4.76 (dd, ^3J = 9.1 Hz, ^3J = 5.6 Hz, 1H, Tyr^α-**H**), 3.96 (t, ^3J = 6.1 Hz, 2H, -C**H**$_2$-O-), 3.72 (s, 3H, -C**H**$_3$), 3.23 – 3.17 (m, 3H, BocNH-C**H**$_2$-, Tyr^β-**H**/H´), 3.05 (dd, ^2J = 13.9 Hz, ^3J = 9.1 Hz, 1H, Tyr^β-H/**H**´), 1.90 (p, ^3J = 6.5 Hz, 2H, -CH$_2$-C**H**$_2$-CH$_2$-), 1.42 (s, 9H, Boc-**H**).

^{13}C{^{1}H}-NMR (151 MHz, Methanol-*d*$_4$) δ [ppm] = 174.0 (-**C**OOMe), 169.9 (Ar-**C**ONH-), 162.3 (Ar-**C**4), 159.4 (Tyr-**C**4), 158.6 (^{t}BuO**C**ONH-), 131.2 (Tyr-**C**2/2´), 130.4 (Tyr-**C**1, Ar-**C**2/2´), 125.9 (Ar-**C**1), 116.0 (Ar-**C**3/3´), 115.5 (Tyr-**C**3/3´), 79.9 (-**C**(CH$_3$)$_3$), 66.6 (-**C**H$_2$-O-), 56.0 (Tyr-**C**^{α}), 52.7 (-**C**H$_3$), 38.5 (BocNH-**C**H$_2$), 37.4 (Tyr-**C**^{β}), 30.7 (-CH$_2$-**C**H$_2$-CH$_2$-), 28.8 (-C(**C**H$_3$)$_3$).

LC-MS (ESI+) m/z: 473.1 [M+H]$^+$, 417.1 [M-^{t}Bu+H]$^+$, 373.1 [M-Boc+H]$^+$; t_R = 8.6min.

3.5.3 Methyl (*S*)-3-(4-(3-((Tert-butoxycarbonyl)amino)propoxy)phenyl)-2-(3-hydroxy-benzamido)propanoate (**5c**)

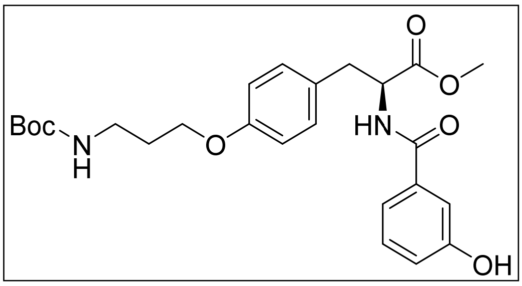

Synthesis according to **GP-4b**: Prepared from **4a**.

The RGD mimetic precursor **5c** (34.9 mg, 74.0 µmol, 26 %) was isolated as a colourless solid.

^{1}H-NMR (600 MHz, Methanol-*d*$_4$) δ [ppm] = 7.26 (dd, ^3J = 7.8 Hz, ^3J = 7.8 Hz, 1H, Ar-5-**H**), 7.20 (dd, ^3J = 7.7 Hz, ^4J = 1.3 Hz, 1H, Ar-6-**H**), 7.18 – 7.15 (m, 3H, Tyr-2/2´-**H**, Ar-1-**H**), 6.96 (ddd, ^3J = 8.1 Hz, ^4J = 2.6 Hz, ^4J = 1.1 Hz, 1H, Ar-4-**H**), 6.86 (d, ^3J = 8.6 Hz, 2H, Tyr-3/3´-**H**), 4.79 (dd, ^3J = 9.2 Hz, ^3J = 5.6 Hz, 1H, Tyr^α-**H**), 3.98 (t, ^3J = 6.2 Hz, 2H, -C**H**$_2$-O-), 3.74 (s, 3H, -C**H**$_3$), 3.27 – 3.17 (m, 3H, BocNH-C**H**$_2$-, Tyr^β-**H**/H´), 3.06 (dd, ^2J = 14.0 Hz, ^3J = 9.1 Hz, 1H, Tyr^β-H/**H**´), 1.92 (tt, ^3J = 6.5 Hz, ^3J = 6.5 Hz, 2H, -CH$_2$-C**H**$_2$-CH$_2$-), 1.43 (s, 9H, Boc-**H**).

^{13}C{^{1}H}-NMR (151 MHz, Methanol-*d*$_4$) δ [ppm] = 173.7 (-**C**OOMe), 170.3 (Ar-**C**ONH-), 159.4 (Tyr-C4), 158.8 (Ar-**C**3), 158.6 (^{t}BuO**C**ONH-), 136.6 (Ar-**C**1), 131.2 (Tyr-**C**2/2´), 130.6 (Ar-**C**5), 130.4 (Tyr-**C**1), 119.8 (Ar-**C**4), 119.2 (Ar-**C**6), 115.6 (Tyr-**C**3/3´), 115.3 (Ar-**C**2), 80.0 (-**C**(CH$_3$)$_3$), 66.6

(-CH$_2$-O-), 56.0 (Tyr-**C**$^{\alpha}$), 52.7 (-**C**H$_3$), 38.5 (BocNH-**C**H$_2$), 37.3 (Tyr-**C**$^{\beta}$), 30.7 (-CH$_2$-**C**H$_2$-CH$_2$-), 28.8 (-C(**C**H$_3$)$_3$).

LC-MS (ESI+) m/z: 473.1 [M+H]$^+$, 417.1 [M-^{t}Bu+H]$^+$, 373.1 [M-Boc+H]$^+$; t_R = 8.7min.

3.5.4 Methyl (*S*)-2-Benzamido-3-(4-(4-((tert-butoxycarbonyl)amino)butoxy)phenyl)-propanoate (**6a**)

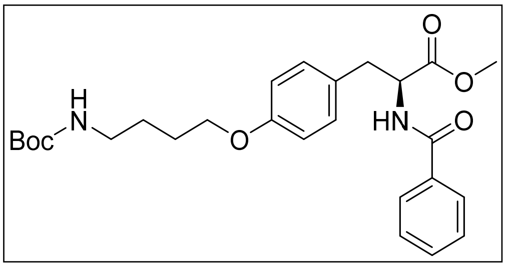

Synthesis according to **GP-4a**: Prepared from **4b**.

The RGD mimetic precursor **6a** (48.9 mg, 104.0 µmol, 38 %) was isolated as a colourless solid.

^{1}H-NMR (600 MHz, Methanol-*d*$_4$) δ [ppm] = 7.75 (d, ^3J = 7.1 Hz, 2H, Ar-2/2′-**H**), 7.54 (t, ^3J = 6.8 Hz, 1H, Ar-5-**H**), 7.45 (t, ^3J = 7.8 Hz, 2H, Ar-3/3′-**H**), 7.17 (d, ^3J = 8.6 Hz, 2H, Tyr-2/2′-**H**), 6.84 (d, ^3J = 8.6 Hz, 2H, Tyr-3/3′-**H**), 4.81 (dd, ^3J = 9.3, ^3J = 5.6 Hz, 1H, Tyr$^{\alpha}$-**H**), 3.95 (t, ^3J = 6.3 Hz, 2H, -C**H**$_2$-O-), 3.74 (s, 3H, -C**H**$_3$), 3.23 (dd, ^2J = 14.0 Hz, ^3J = 5.6 Hz, 1H, Tyr$^{\beta}$-**H**/H′), 3.09 (t, ^3J = 6.9 Hz, 2H, BocNH-C**H**$_2$-), 3.06 (dd, ^2J = 14.0 Hz, ^3J = 9.4 Hz, 1H, Tyr$^{\beta}$-H/**H**′), 1.88 – 1.73 (m, 2H, -C**H**$_2$-CH$_2$-O-), 1.63 (tt, ^3J = 7.2 Hz, ^3J = 7.2 Hz, 2H, BocNH-CH$_2$-C**H**$_2$-), 1.44 (s, 9H, Boc-**H**).

^{13}C{^{1}H}-NMR (151 MHz, Methanol-*d*$_4$) δ [ppm] = 173.7 (-**C**OOMe), 170.2 (Ar-**C**ONH-), 159.5 (Tyr-**C**4), 158.6 (^{t}BuO**C**ONH-), 135.2 (Ar-**C**1), 132.9 (Ar-**C**4), 131.2 (Tyr-**C**2/2′), 130.3 (Tyr-**C**1), 129.5 (Ar-**C**3/3′), 128.4 (Ar-**C**2/2′), 115.6 (Tyr-**C**3/3′), 79.9 (-**C**(CH$_3$)$_3$), 68.6 (-**C**H$_2$-O-), 56.1 (Tyr-**C**$^{\alpha}$), 52.7 (-**C**H$_3$), 41.1 (BocNH-**C**H$_2$), 37.4 (Tyr-**C**$^{\beta}$), 28.8 (-C(**C**H$_3$)$_3$), 27.7 (-**C**H$_2$-CH$_2$-O-), 27.6 (BocNH-CH$_2$-**C**H$_2$-).

LC-MS (ESI+) m/z: 471.2 [M+H]$^+$, 415.1 [M-^{t}Bu+H]$^+$, 371.1 [M-Boc+H]$^+$; t_R = 9.6min.

3.5.5 Methyl (*S*)-3-(4-(4-((Tert-butoxycarbonyl)amino)butoxy)phenyl)-2-(4-hydroxy-benzamido)propanoate (**6b**)

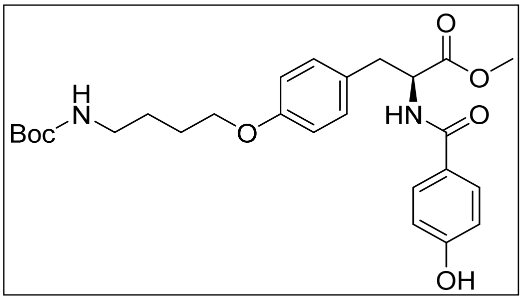

Synthesis according to **GP-4b**: Prepared from **4b**.

The RGD mimetic precursor **6b** (51.1 mg, 105.0 µmol, 38 %) was isolated as a colourless solid.

^{1}H-NMR (600 MHz, Methanol-*d*$_4$) δ [ppm] = 7.65 (d, ^3J = 8.7 Hz, 2H, Ar-2/2′-**H**), 7.15 (d, ^3J = 8.5 Hz, 2H, Tyr-2/2′-**H**), 6.83 (d, ^3J = 8.6 Hz, 2H, Tyr-3/3′-**H**), 6.81 (d, ^3J = 8.7 Hz, 2H, Ar-3/3′-**H**), 4.76 (dd, ^3J = 9.1 Hz, ^3J = 5.7 Hz, 1H, Tyr$^{\alpha}$-**H**), 3.94 (t, ^3J = 6.3 Hz, 2H, -C**H**$_2$-O-), 3.72 (s, 3H, -C**H**$_3$), 3.19 (dd,

^{2}J = 14.0 Hz, ^{3}J = 5.7 Hz, 1H, Tyr^{β}-**H**/H′), 3.09 (t, ^{3}J = 7.0 Hz, 2H, BocNH-C**H**$_2$-), 3.05 (dd, ^{2}J = 14.0 Hz, ^{3}J = 9.1 Hz, 1H, Tyr^{β}-H/**H′**), 1.75 (tt, ^{3}J = 8.4 Hz, ^{3}J = 6.4 Hz, 2H, -C**H**$_2$-CH$_2$-O-), 1.62 (tt, ^{3}J = 7.2 Hz, ^{3}J = 7.2 Hz, 2H, BocNH-CH$_2$-C**H**$_2$-), 1.43 (s, 9H, Boc-**H**).

^{13}C{^{1}H}-NMR (151 MHz, Methanol-d_4) δ [ppm] = 174.0 (-**C**OOMe), 169.9 (Ar-**C**ONH-), 162.3 (Ar-**C**4), 159.4 (Tyr-**C**4), 158.6 (^{t}BuO**C**ONH-), 131.2 (Tyr-**C**2/2′), 130.4 (Ar-**C**2/2′), 130.3 (Tyr-**C**1), 125.9 (Ar-**C**1), 116.1 (Ar-**C**3/3′), 115.5 (Tyr-**C**3/3′), 79.9 (-**C**(CH$_3$)$_3$), 68.6 (-**C**H$_2$-O-), 56.1 (Tyr-**C**^{α}), 52.7 (-**C**H$_3$), 41.1 (BocNH-**C**H$_2$), 37.4 (Tyr-**C**^{β}), 28.8 (-C(**C**H$_3$)$_3$), 27.7 (-**C**H$_2$-CH$_2$-O-), 27.6 (BocNH-CH$_2$-**C**H$_2$-).

LC-MS (ESI+) m/z: 487.1 [M+H]$^+$, 431.1 [M-^{t}Bu+H]$^+$, 387.1 [M-Boc+H]$^+$; t_R = 8.8min.

3.5.6 Methyl (*S*)-3-(4-(4-((Tert-butoxycarbonyl)amino)butoxy)phenyl)-2-(3-hydroxy-benzamido)propanoate (**6c**)

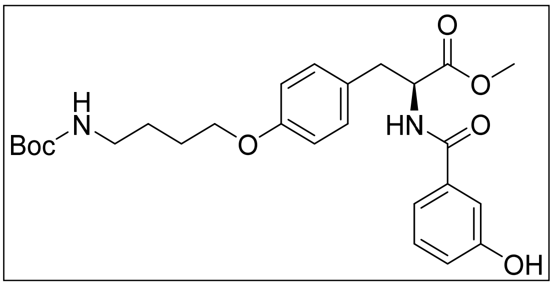

Synthesis according to **GP-4b**: Prepared from **4b**.

The RGD mimetic precursor **6c** (46.6 mg, 96.0 µmol, 35 %) was isolated as a colourless solid.

^{1}H-NMR (600 MHz, Methanol-d_4) δ [ppm] = 7.25 (dd, ^{3}J = 7.9 Hz, ^{3}J = 7.9 Hz, 1H, Ar-5-**H**), 7.19 (dd, ^{3}J = 7.7 Hz, ^{4}J = 1.3 Hz, 1H, Ar-6-**H**), 7.18 – 7.14 (m, 3H, Ar-2-**H**, Tyr-2/2′-**H**), 6.96 (ddd, ^{3}J = 8.0 Hz, ^{4}J = 2.6 Hz, ^{4}J = 1.1 Hz, 1H, Ar-4-**H**), 6.84 (d, ^{3}J = 8.6 Hz, 2H, Tyr-3/3′-**H**), 4.78 (dd, ^{3}J = 9.2 Hz, ^{3}J = 5.6 Hz, 1H, Tyr^{α}-**H**), 3.95 (t, ^{3}J = 6.3 Hz, 2H, -C**H**$_2$-O-), 3.73 (s, 3H, -C**H**$_3$), 3.21 (dd, ^{2}J = 14.0 Hz, ^{3}J = 5.6 Hz, 1H, Tyr^{β}-**H**/H′), 3.10 (t, ^{3}J = 7.0 Hz, 2H, BocNH-C**H**$_2$-), 3.05 (dd, ^{2}J = 14.0 Hz, ^{3}J = 9.2 Hz, 1H, Tyr^{β}-H/**H′**), 1.81 – 1.72 (m, 2H, -C**H**$_2$-CH$_2$-O-), 1.63 (p, ^{3}J = 7.2 Hz, 2H, BocNH-CH$_2$-C**H**$_2$-), 1.44 (s, 9H, Boc-**H**).

^{13}C{^{1}H}-NMR (151 MHz, Methanol-d_4) δ [ppm] = 173.8 (-**C**OOMe), 170.3 (Ar-**C**ONH-), 159.4 (Tyr-**C**4), 158.7 (Ar-**C**3), 158.6 (^{t}BuO**C**ONH-), 136.5 (Ar-**C**1), 131.2 (Tyr-**C**2/2′), 130.6 (Ar-**C**5), 130.2 (Tyr-**C**1), 119.8 (Ar-**C**4), 119.3 (Ar-**C**2), 115.6 (Tyr-**C**3/3′), 115.3 (Ar-**C**6), 79.9 (-**C**(CH$_3$)$_3$), 68.6 (-**C**H$_2$-O-), 56.0 (Tyr-**C**^{α}), 52.8 (-**C**H$_3$), 41.1 (BocNH-**C**H$_2$), 37.3 (Tyr-**C**^{β}), 28.8 (-C(**C**H$_3$)$_3$), 27.7 (-**C**H$_2$-CH$_2$-O-), 27.6 (BocNH-CH$_2$-**C**H$_2$-).

LC-MS (ESI+) m/z: 487.1 [M+H]$^+$, 431.1 [M-^{t}Bu+H]$^+$, 387.1 [M-Boc+H]$^+$; t_R = 8.9 min.

3.5.7 Methyl (*S*)-2-Benzamido-3-(4-((5-((tert-butoxycarbonyl)amino)pentyl)oxy)phenyl)-propanoate (**7a**)

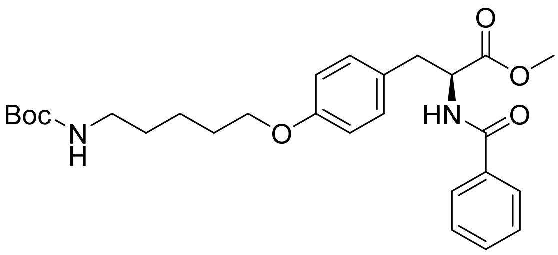

Synthesis according to **GP-4a**: Prepared from **4c**.

The RGD mimetic precursor **7a** (49.7 mg, 102.5 µmol, 26 %) was isolated as a colourless solid.

^{1}H-NMR (600 MHz, Methanol-*d*$_4$) δ [ppm] = 7.75 (d, ^3J = 8.6 Hz, 2H, Ar-2/2´-**H**), 7.54 (t, ^3J = 7.4 Hz, 1H, Ar-4-**H**), 7.45 (dd, ^3J = 8.6 Hz, ^3J = 7.5 Hz, 2H, Ar-3/3´-**H**), 7.16 (d, ^3J = 8.6 Hz, 2H, Tyr-2/2´-**H**), 6.84 (d, ^3J = 8.6 Hz, 2H, Tyr-3/3´-**H**), 4.81 (dd, ^3J = 9.3 Hz, ^3J = 5.6 Hz, 1H, Tyr^α-**H**), 3.94 (t, ^3J = 6.4 Hz, 2H, -C**H**$_2$-O-), 3.74 (s, 3H, -C**H**$_3$), 3.23 (dd, ^3J = 14.0 Hz, ^3J = 5.6 Hz, 1H, Tyr^β-**H**/H´), 3.09 – 3.03 (m, 3H, Tyr^β-H/**H**´, BocNH-C**H**$_2$), 1.81 – 1.71 (m, 2H, BocNH-CH$_2$-C**H**$_2$-), 1.58 – 1.45 (m, 4H, -C**H**$_2$-C**H**$_2$-CH$_2$-O-), 1.44 (s, 9H, Boc-**H**).

^{13}C{^{1}H}-NMR (151 MHz, Methanol-*d*$_4$) δ [ppm] = 173.7 (-**C**OOMe), 170.2 (Ar-**C**ONH-), 159.5 (Tyr-**C**4), 158.6 (^{t}BuO**C**ONH-), 135.2 (Ar-**C**1), 132.9 (Ar-**C**4), 131.2 (Tyr-**C**2/2´), 130.2 (Tyr-**C**1), 129.5 (Ar-**C**3/3´), 128.4 (Ar-**C**2/2´), 115.5 (Tyr-**C**3/3´), 79.8 (-**C**(CH$_3$)$_3$), 68.8 (-**C**H$_2$-O-), 56.1 (Tyr-**C**^{α}), 52.7 (-**C**H$_3$), 41.2 (BocNH-**C**H$_2$), 37.4 (Tyr-**C**^{β}), 30.7 (-**C**H$_2$-CH$_2$-O-), 30.1 (BocNH-CH$_2$-**C**H$_2$-), 28.8 (-C(**C**H$_3$)$_3$), 24.4 (-**C**H$_2$-CH$_2$-CH$_2$-O-).

LC-MS (ESI+) m/z: 485.4 [M+H]$^+$, 429.3 [M-^{t}Bu+H]$^+$, 385.3 [M-Boc+H]$^+$; t_R = 10.0 min.

3.5.8 Methyl (*S*)-3-(4-((5-((Tert-butoxycarbonyl)amino)pentyl)oxy)phenyl)-2-(4-hydroxy-benzamido)propanoate (**7b**)

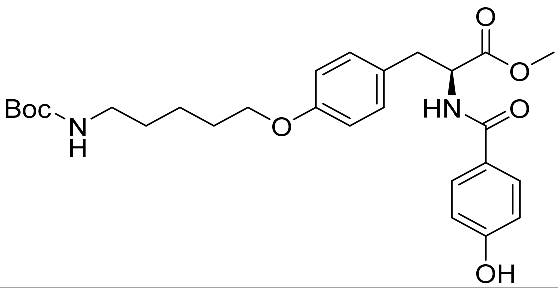

Synthesis according to **GP-4b**: Prepared from **4c**.

The RGD mimetic precursor **7b** (31.5 mg, 63.0 µmol, 24 %) was isolated as a colourless solid.

^{1}H-NMR (600 MHz, Methanol-*d*$_4$) δ [ppm] = 7.65 (d, ^3J = 8.7 Hz, 2H, Ar-2/2´-**H**), 7.14 (d, ^3J = 8.5 Hz, 2H, Tyr-2/2´-**H**), 6.82 (d, ^3J = 8.5 Hz, 2H, Tyr-3/3´-**H**), 6.81 (d, ^3J = 8.7 Hz, 2H, Ar-3/3´-**H**), 4.76 (dd, ^3J = 9.1 Hz, ^3J = 5.5 Hz, 1H, Tyr^α-**H**), 3.92 (t, ^3J = 6.4 Hz, 2H, -C**H**$_2$-O-), 3.71 (s, 3H, -C**H**$_3$), 3.19 (dd, ^2J = 13.9 Hz, ^3J = 5.6 Hz, 1H, Tyr^β-**H**/H´), 3.08 – 3.02 (m, 3H, Tyr^β-H/**H**´, BocNH-C**H**$_2$-), 1.75 (tt, ^3J = 6.6 Hz, ^3J = 6.6 Hz, 2H, BocNH-CH$_2$-C**H**$_2$-), 1.60 – 1.44 (m, 4H, -C**H**$_2$-C**H**$_2$-CH$_2$-O-), 1.43 (s, 9H, Boc-**H**).

^{13}C{^{1}H}-NMR (151 MHz, Methanol-*d*$_4$) δ [ppm] = 174.0 (-**C**OOMe), 169.9 (Ar-**C**ONH-), 162.2 (Ar-**C**4), 159.5 (Tyr-**C**4), 158.6 (^{t}BuO**C**ONH-), 131.2 (Tyr-**C**2/2´), 130.4 (Ar-**C**2/2´), 130.3 (Tyr-**C**1),

125.9 (Ar-**C**1), 116.1 (Ar-**C**3/3′), 115.5 (Tyr-**C**3/3′), 79.8 (-**C**(CH_3)$_3$), 68.8 (-**C**H_2-O-), 56.1 (Tyr-**C**$^\alpha$), 52.7 (-**C**H_3), 41.2 (BocNH-**C**H_2), 37.4 (Tyr-**C**$^\beta$), 30.7 (-**C**H_2-CH_2-O-), 30.1 (BocNH-CH_2-**C**H_2-), 28.8 (-C(**C**H_3)$_3$), 24.4 (-**C**H_2-CH_2-CH_2-O-).

LC-MS (ESI+) m/z: 501.3 $[M+H]^+$, 445.2 $[M-{}^tBu+H]^+$, 401.2 $[M-Boc+H]^+$; t_R = 9.2 min.

3.5.9 Methyl (*S*)-3-(4-((5-((Tert-butoxycarbonyl)amino)pentyl)oxy)phenyl)-2-(3-hydroxy-benzamido)propanoate (**7c**)

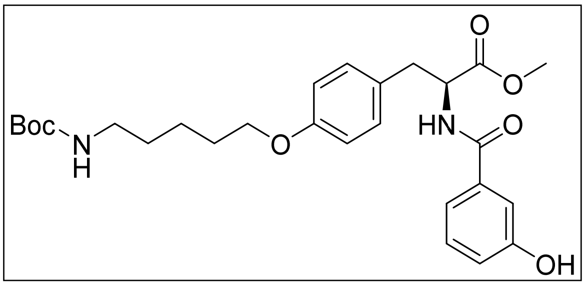

Synthesis according to **GP-4b**: Prepared from **4c**.

The RGD mimetic precursor **7c** (42.8 mg, 85.5 µmol, 33 %) was isolated as a colourless solid.

^{1}H-NMR (600 MHz, Methanol-d_4) δ [ppm] = 7.26 (dd, ^3J = 7.8 Hz, ^3J = 7.8 Hz, 1H, Ar-5-**H**), 7.20 (ddd, ^3J = 7.8 Hz, ^4J = 1.3 Hz, ^4J = 1.3 Hz, 1H, Ar-6-**H**), 7.18 – 7.13 (m, 3H, Tyr-2/2′-**H**, Ar-2-**H**), 6.95 (ddd, ^3J = 8.0 Hz, ^4J = 2.6 Hz, ^4J = 1.2 Hz, 1H, Ar-4-**H**), 6.84 (d, ^3J = 8.6 Hz, 2H, Tyr-3/3′-**H**), 4.79 (dd, ^3J = 9.2 Hz, ^3J = 5.4 Hz, 1H, Tyr$^\alpha$-**H**), 3.94 (t, ^3J = 6.4 Hz, 2H, -C**H**$_2$-O-), 3.74 (s, 3H, -C**H**$_3$), 3.22 (dd, ^2J = 13.9 Hz, ^3J = 5.5 Hz, 1H, Tyr$^\beta$-**H**/H′), 3.10 – 3.02 (m, 3H, Tyr$^\beta$-H/**H′**, BocNH-C**H**$_2$-), 1.85 – 1.69 (m, 2H, BocNH-CH_2-C**H**$_2$-), 1.61 – 1.46 (m, 4H, -C**H**$_2$-CH_2-O-, -CH_2-C**H**$_2$-CH_2-), 1.44 (s, 9H, Boc-**H**).

^{13}C{^{1}H}-NMR (151 MHz, Methanol-d_4) δ [ppm] = 173.7 (-**C**OOMe), 170.3 (Ar-**C**ONH-), 159.5 (Tyr-**C**4), 158.8 (Ar-**C**3), 158.6 (^{t}BuO**C**ONH-), 136.6 (Ar-**C**1), 131.2 (Tyr-**C**2/2′), 130.6 (Ar-**C**5), 130.2 (Tyr-**C**1), 119.8 (Ar-**C**4), 119.2 (Ar-**C**6), 115.6 (Tyr-**C**3/3′), 115.3 (Ar-**C**1), 79.8 (-**C**(CH_3)$_3$), 68.8 (-**C**H_2-O-), 56.1 (Tyr-**C**$^\alpha$), 52.7 (-**C**H_3), 41.3 (BocNH-**C**H_2), 37.4 (Tyr-**C**$^\beta$), 30.7 (-**C**H_2-CH_2-O-), 30.1 (BocNH-CH_2-**C**H_2-), 28.8 (-C(**C**H_3)$_3$), 24.4 (-CH_2-**C**H_2-CH_2-).

LC-MS (ESI+) m/z: 501.3 $[M+H]^+$, 445.2 $[M-{}^tBu+H]^+$, 401.2 $[M-Boc+H]^+$; t_R = 9.3 min

3.6 Final Small Molecule RGD-Mimetics

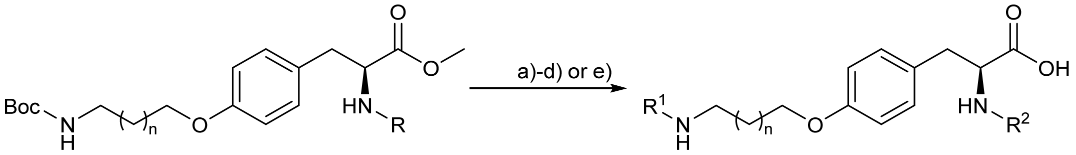

Figure S11: Final reaction steps in the synthesis of the final RGD mimetics **5aa-7cd**. Reagents and conditions: a) 4 M HCl in dioxane, DCM, RT; b) 2-methylthio-2-imidazoline (DHI) or 2-bromopyrimidine (Pyr/THP), triethylamine, methanol, 80 °C, o.n.; c) LiOH, water, methanol, RT; d) Pd/C, H_2, AcOH, water, methanol, RT, o.n. or e) 2-bromopyridine, HBr, Pd/C, H_2, AcOH, water, methanol, RT, o.n..

3.6.1 (*S*)-2-Benzamido-3-(4-(3-((4,5-dihydro-1H-imidazol-2-yl)amino)propoxy)phenyl)-propanoic acid (**5aa**)

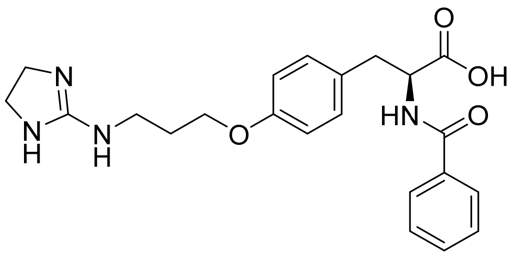

Synthesis according to **GP-5a**: Prepared from **5a**.

The final DHI substituted RGD mimetic **5aa** (9.4 mg, 18.0 µmol, 60 %) was isolated as a colourless TFA salt.

^{1}H-NMR (600 MHz, Methanol-d_4) δ [ppm] = 7.75 (d, ^3J = 7.0 Hz, 2H, Ar-2/2′-**H**), 7.54 (t, ^3J = 7.4 Hz, 1H, Ar-4-**H**), 7.45 (dd, ^3J = 7.4 Hz, ^3J = 7.0 Hz, 2H, Ar-3/3′-**H**), 7.22 (d, ^3J = 8.7 Hz, 2H, Tyr-2/2′-**H**), 6.87 (d, ^3J = 8.6 Hz, 2H, Tyr-3/3′-**H**), 4.83 (dd, ^3J = 9.8 Hz, ^3J = 4.9 Hz, 1H, Tyr$^\alpha$-**H**), 4.02 (t, ^3J = 5.8 Hz, 2H, -C**H**$_2$-O-), 3.59 (s, 4H, DHI-**H**4/5), 3.40 (t, ^3J = 6.7 Hz, 2H, DHI-NH-C**H**$_2$-), 3.29 (d, ^2J = 14.0 Hz, ^3J = 4.9 Hz, 1H, Tyr$^\beta$-**H**/H′), 3.05 (dd, ^2J = 14.0 Hz, ^3J = 9.8 Hz, 1H, Tyr$^\beta$-H/**H**′), 2.03 (tt, ^3J = 5.8 Hz, ^3J = 6.7 Hz, 2H, -CH$_2$-C**H**$_2$-CH$_2$-).

^{13}C{^{1}H}-NMR (151 MHz, Methanol-d_4) δ [ppm] = 174.8 (-**C**OOH), 170.1 (Ar-**C**ONH-), 161.4 (DHI-**C**2), 159.1 (Tyr-**C**4), 135.4 (Ar-**C**1), 132.8 (Ar-**C**4), 131.3 (Tyr-**C**2/2′), 131.2 (Tyr-**C**1), 129.5 (Ar-**C**3/3′), 128.4 (Ar-**C**2/2′), 115.4 (Tyr-**C**3/3′), 65.8 (-**C**H$_2$-O-), 55.8 (Tyr-**C**$^\alpha$), 44.0 (DHI-**C**4/5), 41.0 (DHI-NH-**C**H$_2$-), 37.5 (Tyr-**C**$^\beta$), 29.9 (-CH$_2$-**C**H$_2$-CH$_2$-).

^{19}F{^{1}H}-NMR (470 MHz, Methanol-d_4) δ [ppm] = -77.01 (TFA).

LC-MS (ESI+) m/z: 821.2 $[2M+H]^+$, 411.1 $[M+H]^+$; t_R = 4.8 min.

3.6.2 (*S*)-3-(4-(3-((4,5-Dihydro-1H-imidazol-2-yl)amino)propoxy)phenyl)-2-(4-hydroxy-benzamido)propanoic acid (**5ba**)

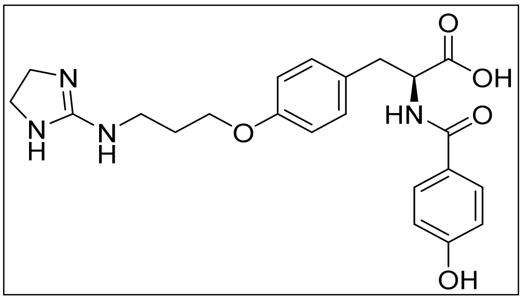

Synthesis according to **GP-5a**: Prepared from **5b**.

The final DHI substituted RGD mimetic **5ba** (10.1 mg, 18.7 µmol, 62 %) was isolated as a colourless TFA salt.

^{1}H-NMR (600 MHz, Methanol-*d*$_4$) δ [ppm] = 7.65 (d, ^3J = 8.7 Hz, 2H, Ar-2/2´-**H**), 7.21 (d, ^3J = 8.6 Hz, 2H, Tyr-2/2´-**H**), 6.86 (d^3J = 8.6 Hz, 2H, Tyr-3/3´-**H**), 6.81 (d, ^3J = 8.7 Hz, 2H, Ar-3/3´-**H**), 4.80 (dd, ^3J = 9.6 Hz, ^3J = 4.9 Hz, 1H, Tyr^α-**H**), 4.02 (t, ^3J = 5.7 Hz, 2H, -C**H**$_2$-O-), 3.58 (s, 4H, DHI-4/5-**H**), 3.40 (t, ^3J = 6.7 Hz, 2H, DHI-NH-C**H**$_2$-), 3.28 (dd, ^2J = 14.0 Hz, ^3J = 4.9 Hz, 1H, Tyr^β-**H**/H´), 3.04 (dd, ^3J = 14.0 Hz, ^3J = 9.6 Hz, 1H, Tyr^β-H/**H**´), 2.08 – 1.95 (m, 2H, -CH$_2$-C**H**$_2$-CH$_2$-).

^{13}C{^{1}H}-NMR (151 MHz, Methanol-*d*$_4$) δ [ppm] = 175.0 (-**C**OOH), 169.8 (Ar-**C**ONH-), 162.2 (Ar-**C**4), 161.4 (DHI-**C**2), 159.0 (Tyr-**C**4), 131.3 (Tyr-**C**2/2´), 131.2 (Tyr-**C**1), 130.4 (Ar-**C**2/2´), 126.0 (Ar-**C**1), 116.0 (Tyr-**C**3/3´), 115.4 (Ar-**C**3/3´), 65.8 (-**C**H$_2$-O-), 55.7 (Tyr-**C**^{α}), 44.0 (DHI-**C**4/5), 41.0 (DHI-NH-**C**H$_2$-), 37.5 (Tyr-**C**^{β}), 29.9 (-CH$_2$-**C**H$_2$-CH$_2$-).

^{19}F{^{1}H}-NMR (470 MHz, Methanol-*d*$_4$) δ [ppm] = -77.05 (TFA).

LC-MS (ESI+) m/z: 853.2[2M+H]$^+$, 427.1 [M+H]$^+$; t$_R$ = 4.4 min.

3.6.3 (*S*)-3-(4-(3-((4,5-Dihydro-1H-imidazol-2-yl)amino)propoxy)phenyl)-2-(3-hydroxy-benzamido)propanoic acid (**5ca**)

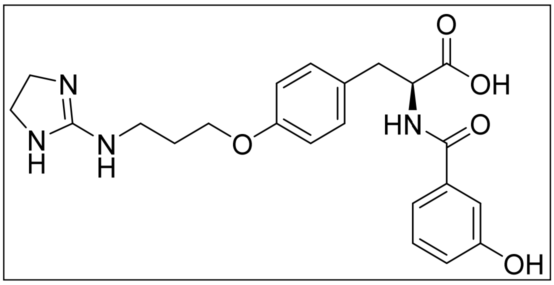

Synthesis according to **GP-5a**: Prepared from **5c**.

The final DHI substituted RGD mimetic **5ca** (7.0 mg, 13.0 µmol, 43 %) was isolated as a colourless TFA salt.

^{1}H-NMR (600 MHz, Methanol-*d*$_4$) δ [ppm] = 7.25 (dd, ^3J = 8.0 Hz, ^3J = 7.7 Hz 1H, Ar-5-**H**), 7.22 (d, ^3J = 8.6 Hz, 2H, Tyr-2/2´-**H**), 7.19 (ddd, ^3J = 7.7 Hz, ^4J = 2.5 Hz, ^4J = 1.1 Hz, 1H, Ar-6-**H**), 7.16 (dd, ^3J = 2.5 Hz, ^4J = 1.1 Hz, 1H, Ar-2-**H**), 6.95 (ddd, ^3J = 8.0 Hz, ^4J = 2.5 Hz, ^4J = 1.1 Hz, 1H, Ar-4-**H**), 6.87 (d, ^3J = 8.6 Hz, 2H, Tyr-3/3´-**H**), 4.81 (dd, ^3J = 9.7 Hz, ^3J = 4.8 Hz, 1H, Tyr^α-**H**), 4.03 (t, ^3J = 5.7 Hz, 2H, -C**H**$_2$-O-), 3.59 (s, 4H, DHI-4/5-**H**), 3.40 (t, ^3J = 6.7 Hz, 2H, DHI-NH-C**H**$_2$-), 3.29 (dd, ^2J = 14.0 Hz, ^3J = 4.8 Hz, 1H, Tyr^β-**H**/H´), 3.04 (dd, ^2J = 14.0 Hz, ^3J = 9.6 Hz, 1H, Tyr^β-H/**H**´), 2.04 (tt, ^3J = 6.7 Hz, ^3J = 5.7 Hz, 2H, -CH$_2$-C**H**$_2$-CH$_2$-).

^{13}C{^{1}H}-NMR (151 MHz, Methanol-*d*$_4$) δ [ppm] = 174.8 (-**C**OOH), 170.1 (Ar-**C**ONH-), 161.4 (DHI-**C**2), 159.1 (Tyr-**C**4), 158.8 (Ar-**C**3), 136.8 (Ar-**C**1), 131.4 (Tyr-**C**2/2´), 131.1 (Tyr-**C**1), 130.6 (Ar-**C**5),

119.7 (Ar-**C**4), 119.2 (Ar-**C**6), 115.4 (Tyr-**C**3/3´), 115.3 (Ar-**C**2), 65.8 (-**C**H$_2$-O-), 55.7 (Tyr-**C**$^{\alpha}$), 44.0 (DHI-**C**4/5), 41.1 (DHI-NH-**C**H$_2$-), 37.5 (Tyr-**C**$^{\beta}$), 29.9 (-CH$_2$-**C**H$_2$-CH$_2$-).

^{19}F{^{1}H}-NMR (470 MHz, Methanol-d_4) δ [ppm] = -77.01 (TFA).

LC-MS (ESI+) m/z: 853.2 [2M+H]$^+$, 427.1 [M+H]$^+$; t_R = 4.4 min.

3.6.4 (*S*)-2-(((Benzyloxy)carbonyl)amino)-3-(4-(3-((4,5-dihydro-1H-imidazol-2-yl)amino)-propoxy)phenyl)propanoic acid (**5da**)

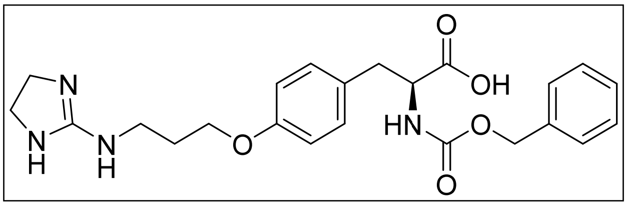

Synthesis according to **GP-5a**: Prepared from **3a**.

The final DHI substituted RGD mimetic **5da** (20.0 mg, 29.9 µmol, 57 %) was isolated as a colourless TFA salt.

^{1}H-NMR (600 MHz, Methanol-d_4) δ [ppm] = 7.35 – 7.24 (m, 5H, Cbz-**H**), 7.13 (d, ^3J = 8.5 Hz, 2H, Tyr-2/2´-**H**), 6.83 (d, ^3J = 8.6 Hz, 2H, Tyr-3/3´-**H**), 5.03 (d, ^2J = 12.6 Hz, 1H, Cbz-C**H**$_2$-), 5.00 (d, ^2J = 12.6 Hz, 1H, Cbz-C**H**$_2$-), 4.35 (dd, ^3J = 9.2 Hz, ^3J = 4.9 Hz, 1H, Tyr$^{\alpha}$-**H**), 4.01 (t, ^3J = 5.8 Hz, 2H, -C**H**$_2$-O-Tyr), 3.61 (s, 4H, DHI-4/5-**H**), 3.39 (t, ^3J = 6.7 Hz, 2H, DHI-NH-C**H**$_2$-), 3.12 (dd, ^2J = 14.0 Hz, ^3J = 4.9 Hz, 1H, Tyr$^{\beta}$-**H**/H´), 2.85 (dd, ^2J = 14.0 Hz, ^3J = 9.2 Hz, 1H, Tyr$^{\beta}$-H/**H**´), 2.02 (tt, ^3J = 6.7 Hz, ^3J = 5.8 Hz, 2H, -CH$_2$-C**H**$_2$-CH$_2$-).

^{13}C{^{1}H}-NMR (151 MHz, Methanol-d_4) δ [ppm] = 175.1 (-**C**OOH), 161.4 (DHI-**C**2), 159.0 (Tyr-**C**4), 158.4 (BnO**C**ONH-), 138.2 (Ar-**C**1), 131.4 (Tyr-**C**2/2´), 130.9 (Tyr-**C**1), 129.4 (Ar-**C**3/3´), 128.9 (Ar-**C**4), 128.6 (Ar-**C**2/2´), 115.4 (Tyr-**C**3/3´), 67.5 (Cbz-**C**H$_2$-), 65.8 (-**C**H$_2$-O-Tyr), 57.0 (Tyr-**C**$^{\alpha}$), 44.0 (DHI-**C**4/5), 41.0 (DHI-NH-**C**H$_2$-), 37.8 (Tyr-**C**$^{\beta}$), 29.9 (-CH$_2$-**C**H$_2$-CH$_2$-).

^{19}F{^{1}H}-NMR (470 MHz, Methanol-d_4) δ [ppm] = -77.01 (TFA).

LC-MS (ESI+) m/z: 1321.7 [3M+H]$^+$, 881.5 [2M+H]$^+$, 441.4 [M+H]$^+$; t_R = 5.3 min.

3.6.5 (*S*)-2-Benzamido-3-(4-(4-((4,5-dihydro-1H-imidazol-2-yl)amino)butoxy)phenyl)-propanoic acid (**6aa**)

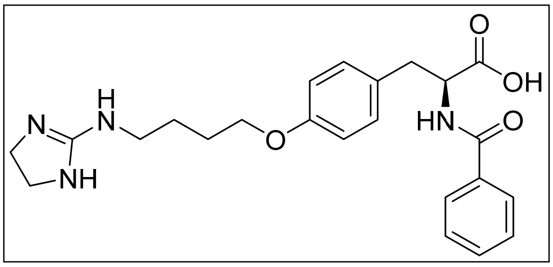

Synthesis according to **GP-5a**: Prepared from **5c**.

The final DHI substituted RGD mimetic **6aa** (5.0 mg, 9.3 µmol, 21 %) was isolated as a colourless TFA salt.

^{1}H-NMR (600 MHz, Methanol-d_4) δ [ppm] = 7.74 (d, ^3J = 8.5 Hz, 1H, Ar-2/2´-**H**), 7.53 (t, ^3J = 7.4 Hz, 1H, Ar-4-**H**), 7.44 (dd, ^3J = 8.5 Hz, ^3J = 7.4 Hz, 2H, Ar-3/3´-**H**), 7.20 (d, ^3J = 8.6 Hz, 2H, Tyr-2/2´-**H**), 6.84 (d, ^3J = 8.6 Hz, 2H, Tyr-3/3´-**H**), 4.82 (dd, ^3J = 9.6 Hz, ^3J = 4.9 Hz, 1H, Tyr^α-**H**), 3.98 (t, ^3J = 5.9 Hz, 2H, -C**H**$_2$-O-), 3.69 (s, 4H, DHI-4/5-**H**), 3.29 (dd, ^2J = 14.1 Hz, ^3J = 4.9 Hz, 1H, Tyr^β-**H**/H´), 3.25 (t, ^3J = 6.9 Hz, 2H, DHI-NH-C**H**$_2$-), 3.05 (dd, ^3J = 14.1 Hz, ^3J = 9.6 Hz, 1H, Tyr^β-H/**H´**), 1.86 – 1.71 (m, 4H, -CH$_2$-C**H**$_2$-C**H**$_2$-CH$_2$-).

^{13}C{^{1}H}-NMR (151 MHz, Methanol-d_4) δ [ppm] = 174.9 (-**C**OOH), 170.2 (Ar-**C**ONH-), 161.3 (DHI-**C**2), 159.3 (Tyr-**C**4), 135.4 (Ar-**C**1), 132.8 (Ar-**C**4), 131.3 (Tyr-**C**2/2´), 130.9 (Tyr-**C**1), 129.5 (Ar-**C**3/3´), 128.4 (Ar-**C**2/2´), 115.5 (Tyr-**C**3/3´), 68.3 (-**C**H$_2$-O-), 55.9 (Tyr-**C**^{α}), 44.0 (DHI-**C**4/5), 43.6 (DHI-NH-**C**H$_2$-), 37.5 (Tyr-**C**^{β}), 27.4 (-CH$_2$-CH$_2$-**C**H$_2$-CH$_2$-), 27.0 (-CH$_2$-**C**H$_2$-CH$_2$-CH$_2$-).

^{19}F{^{1}H}-NMR (470 MHz, Methanol-d_4) δ [ppm] = -77.01 (TFA).

LC-MS (ESI+) m/z: 849.6 [2M+H]$^+$, 425.4 [M+H]$^+$; t_R = 5.1 min.

3.6.6 (*S*)-3-(4-(4-((4,5-Dihydro-1H-imidazol-2-yl)amino)butoxy)phenyl)-2-(4-hydroxybenzamido)propanoic acid (**6ba**)

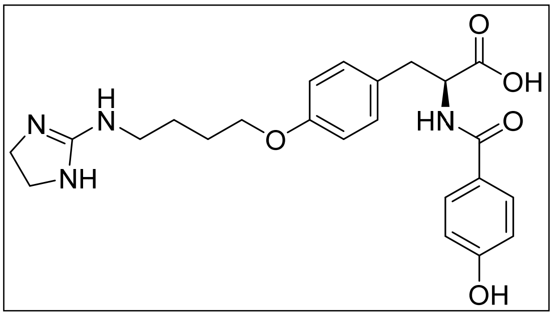

Synthesis according to **GP-5a**: Prepared from **6b**.

The final DHI substituted RGD mimetic **6ba** (9.9 mg, 17.8 µmol, 59 %) was isolated as a colourless TFA salt.

^{1}H-NMR (600 MHz, Methanol-d_4) δ [ppm] = 7.65 (d, ^3J = 8.7 Hz, 2H, Ar-2/2´-**H**), 7.20 (d, ^3J = 8.6 Hz, 2H, Tyr-2/2´-**H**), 6.84 (d^3J = 8.7 Hz, 2H, Tyr-3/3´-**H**), 6.82 (d, ^3J = 8.7 Hz, 2H, Ar-3/3´-**H**), 4.79 (dd, ^3J = 9.4 Hz, ^3J = 5.0 Hz, 1H, Tyr^α-**H**), 3.99 (t, ^3J = 5.8 Hz, 2H, -C**H**$_2$-O-), 3.70 (s, 4H, DHI-4/5-**H**), 3.27 (dd, ^2J = 14.0 Hz, ^3J = 4.9 Hz, 1H, Tyr^β-**H**/H´), 3.26 (t, ^3J = 6.8 Hz, 2H, DHI-NH-C**H**$_2$-), 3.05 (dd, ^2J = 14.0 Hz, ^3J = 9.4 Hz, 1H, Tyr^β-H/**H´**), 1.86 – 1.72 (m, 4H, -CH$_2$-C**H**$_2$-C**H**$_2$-CH$_2$-).

^{13}C{^{1}H}-NMR (151 MHz, Methanol-d_4) δ [ppm] = 175.1 (-**C**OOH), 169.9 (Ar-**C**ONH-), 162.2 (Ar-**C**4), 161.3 (DHI-**C**2), 159.2 (Tyr-**C**4), 131.3 (Tyr-**C**2/2´), 130.9 (Tyr-**C**1), 130.4 (Ar-**C**2/2´), 126.1 (Ar-**C**1), 116.0 (Ar-**C**3/3´), 115.5 (Tyr-**C**3/3´), 68.3 (-**C**H$_2$-O-), 55.8 (Tyr-**C**^{α}), 44.0 (DHI-**C**4/5), 43.6 (DHI-NH-**C**H$_2$-), 37.5 (Tyr-**C**^{β}), 27.4 (-CH$_2$-CH$_2$-**C**H$_2$-CH$_2$-), 27.0 (-CH$_2$-**C**H$_2$-CH$_2$-CH$_2$-).

^{19}F{^{1}H}-NMR (470 MHz, Methanol-d_4) δ [ppm] = -77.06 (TFA).

LC-MS (ESI+) m/z: 881.3 [2M+H]$^+$, 441.1 [M+H]$^+$; t_R = 4.7 min.

3.6.7 (*S*)-3-(4-(4-((4,5-Dihydro-1H-imidazol-2-yl)amino)butoxy)phenyl)-2-(3-hydroxybenzamido)propanoic acid (**6ca**)

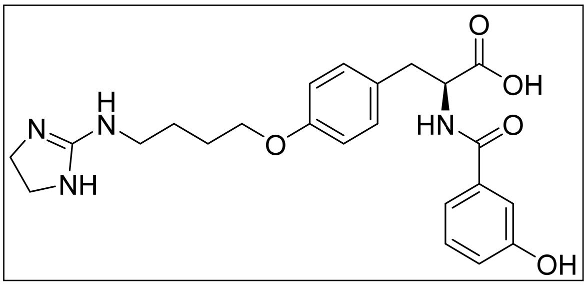

Synthesis according to **GP-5a**: Prepared from **6c**.

The final DHI substituted RGD mimetic **6ca** (7.5 mg, 13.4 µmol, 45 %) was isolated as a colourless TFA salt.

^{1}H-NMR (600 MHz, Methanol-d_4) δ [ppm] = 7.25 (dd, ^3J = 7.8 Hz, ^3J = 8.1 Hz, 1H, Ar-5-**H**), 7.22 – 7.17 (m, 3H, Tyr-2/2′-**H**, Ar-6-**H**), 7.15 (dd, ^4J = 2.1 Hz, ^4J = 1.0 Hz, 1H, Ar-2-**H**), 6.95 (ddd, ^3J = 8.1 Hz, ^4J = 2.6 Hz, ^4J = 1.0 Hz, 1H, Ar-4-**H**), 6.85 (d, ^3J = 8.6 Hz, 2H, Tyr-3/3′-**H**), 4.80 (dd, ^3J = 9.4 Hz, ^3J = 4.9 Hz, 1H, Tyr$^\alpha$-**H**), 3.99 (t, ^3J = 5.8 Hz, 2H, -C**H**$_2$-O-), 3.70 (s, 4H, DHI-4/5-**H**), 3.28 (dd, ^3J = 14.0 Hz, ^3J = 4.9 Hz, 1H, Tyr$^\beta$-**H**/H′), 3.26 (t, ^3J = 6.9 Hz, 2H, DHI-NH-C**H**$_2$-), 3.05 (dd, ^3J = 14.0 Hz, ^3J = 9.4 Hz, 1H, Tyr$^\beta$-H/**H′**), 1.88 – 1.67 (m, 4H, -CH$_2$-C**H**$_2$-C**H**$_2$-CH$_2$-).

^{13}C{^{1}H}-NMR (151 MHz, Methanol-d_4) δ [ppm] = 174.8 (-**C**OOH), 170.2 (Ar-**C**ONH-), 161.3 (DHI-**C**2), 159.3 (Tyr-**C**4), 158.8 (Ar-**C**3), 136.8 (Ar-**C**1), 131.3 (Tyr-**C**2/2′), 130.8 (Tyr-**C**1), 130.6 (Ar-**C**5), 119.7 (Ar-**C**4), 119.2 (Ar-**C**6), 115.5 (Tyr-**C**3/3′), 115.3 (Ar-**C**2), 68.3 (-**C**H$_2$-O-), 55.8 (Tyr-**C**$^\alpha$), 44.0 (DHI-**C**4/5), 43.6 (DHI-NH-**C**H$_2$-), 37.4 (Tyr-**C**$^\beta$), 27.4 (-CH$_2$-CH$_2$-**C**H$_2$-CH$_2$-), 27.0 (-CH$_2$-**C**H$_2$-CH$_2$-CH$_2$-).

^{19}F{^{1}H}-NMR (470 MHz, Methanol-d_4) δ [ppm] = -76.99 (TFA).

LC-MS (ESI+) m/z: 881.3 [2M+H]$^+$, 441.1 [M+H]$^+$; t_R = 4.8 min.

3.6.8 (*S*)-2-(((Benzyloxy)carbonyl)amino)-3-(4-(4-((4,5-dihydro-1H-imidazol-2-yl)amino)-butoxy)phenyl)propanoic acid (**6da**)

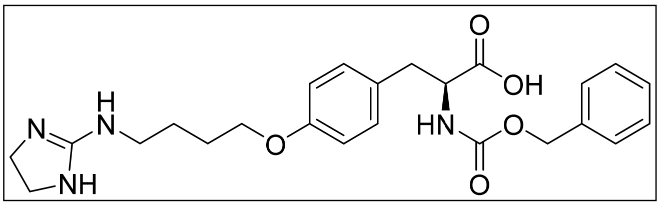

Synthesis according to **GP-5a**: Prepared from **3c**.

The final DHI substituted RGD mimetic **6da** (31.5 mg, 55.0 µmol, 61 %) was isolated as a colourless TFA salt.

^{1}H-NMR (600 MHz, Methanol-d_4) δ [ppm] = 7.34 – 7.23 (m, 5H, Cbz-**H**), 7.11 (d, ^3J = 8.0 Hz, 2H, Tyr-2/2′-**H**), 6.80 (d, ^3J = 8.1 Hz, 2H, Tyr-3/3′-**H**), 5.04 (d, ^2J = 12.7 Hz, 1H, Cbz-C**H**$_2$-), 4.99 (d, ^2J = 12.4 Hz, 1H, Cbz-C**H**$_2$-), 4.36 (dd, ^3J = 9.4 Hz, ^3J = 4.9 Hz, 1H, Tyr$^\alpha$-**H**), 3.97 (t, ^3J = 5.9 Hz, 2H, -C**H**$_2$-O-Tyr), 3.67 (s, 4H, DHI-4/5-**H**), 3.25 (t, ^3J = 6.7 Hz, 2H, DHI-NH-C**H**$_2$-), 3.12 (dd,

^{2}J = 14.1 Hz, ^{3}J = 4.9 Hz, 1H, Tyr$^{\beta}$-**H**/H′), 2.85 (dd, ^{2}J = 14.1 Hz, ^{3}J = 9.3 Hz, 1H, Tyr$^{\beta}$-H/**H**′), 1.92 – 1.69 (m, 4H, -CH$_2$-C**H**$_2$-C**H**$_2$-CH$_2$-).

^{13}C{^{1}H}-NMR (151 MHz, Methanol-*d*$_4$) δ [ppm] = 175.2 (-**C**OOH), 161.3 (DHI-**C**2), 159.2 (Tyr-**C**4), 158.4 (BnO**C**ONH-), 138.2 (Ar-**C**1), 131.3 (Tyr-**C**2/2′), 130.6 (Tyr-**C**1), 129.4 (Ar-**C**3/3′), 128.9 (Ar-**C**4), 128.6 (Ar-**C**2/2′), 115.4 (Tyr-**C**3/3′), 68.3 (-**C**H$_2$-O-Tyr), 67.4 (Cbz-**C**H$_2$-), 57.0 (Tyr-**C**$^{\alpha}$), 44.0 (DHI-**C**4/5), 43.6 (DHI-NH-**C**H$_2$-), 37.8 (Tyr-**C**$^{\beta}$), 27.4 (-**C**H$_2$-CH$_2$-O-Tyr), 27.0 (DHI-NH-CH$_2$-**C**H$_2$-).

^{19}F{^{1}H}-NMR (470 MHz, Methanol-*d*$_4$) δ [ppm] = -77.00 (TFA).

LC-MS (ESI+) m/z: 1363.7 [3M+H]$^+$, 909.5 [2M+H]$^+$, 455.4 [M+H]$^+$; t_R = 5.6 min.

3.6.9 (*S*)-2-Benzamido-3-(4-((5-((4,5-dihydro-1H-imidazol-2-yl)amino)pentyl)oxy)phenyl)-propanoic acid (**7aa**)

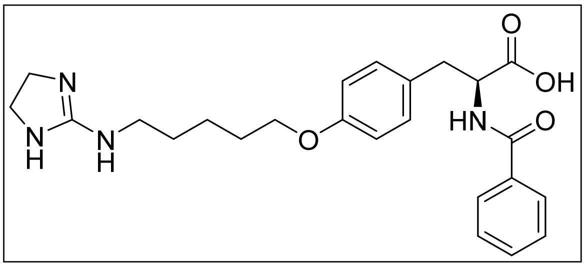

Synthesis according to **GP-5a**: Prepared from **7a**.

The final DHI substituted RGD mimetic **7aa** (9.6 mg, 17.3 µmol, 86 %) was isolated as a colourless TFA salt.

^{1}H-NMR (600 MHz, Methanol-*d*$_4$) δ [ppm] = 7.73 (d, ^{3}J = 8.5 Hz, 2H, Ar-2/2′-**H**), 7.53 (t, ^{3}J = 7.4 Hz, 1H, Ar-4-**H**), 7.44 (dd, ^{3}J = 8.5 Hz, ^{3}J = 7.4 Hz, 2H, Ar-3/3′-**H**), 7.19 (d, ^{3}J = 8.6 Hz, 2H, Tyr-2/2′-**H**), 6.82 (d, ^{3}J = 8.6 Hz, 2H, Tyr-3/3′-**H**), 4.81 (dd, ^{3}J = 9.6 Hz, ^{3}J = 4.9 Hz, 1H, Tyr$^{\alpha}$-**H**), 3.95 (t, ^{3}J = 6.2 Hz, 2H, -C**H**$_2$-O-), 3.68 (s, 4H, DHI-4/5-**H**), 3.28 (dd, ^{3}J = 14.1 Hz, ^{3}J = 4.9 Hz, 1H, Tyr$^{\beta}$-**H**/H′), 3.19 (t, ^{3}J = 7.0 Hz, 2H, DHI-NH-C**H**$_2$-), 3.04 (dd, ^{3}J = 14.1 Hz, ^{3}J = 9.6 Hz, 1H, Tyr$^{\beta}$-H/**H**′), 1.82 – 1.73 (m, 2H, DHI-NH-CH$_2$-C**H**$_2$-), 1.69 – 1.60 (m, 2H, -C**H**$_2$-CH$_2$-O-), 1.53 (tt, ^{3}J = 9.9 Hz, ^{3}J = 6.0 Hz, 2H, -C**H**$_2$-CH$_2$-CH$_2$-O-).

^{13}C{^{1}H}-NMR (151 MHz, Methanol-*d*$_4$) δ [ppm] = 174.9 (-**C**OOH), 170.2 (Ar-**C**ONH-), 161.3 (DHI-**C**2), 159.4 (Tyr-**C**4), 135.4 (Ar-**C**1), 132.8 (Ar-**C**4), 131.3 (Tyr-**C**2/2′), 130.7 (Tyr-**C**1), 129.5 (Ar-**C**3/3′), 128.4 (Ar-**C**2/2′), 115.5 (Tyr-**C**3/3′), 68.6 (-**C**H$_2$-O-), 55.9 (Tyr-**C**$^{\alpha}$), 44.0 (DHI-**C**4/5), 43.8 (DHI-NH-**C**H$_2$-), 37.5 (Tyr-**C**$^{\beta}$), 29.9 (DHI-NH-CH$_2$-**C**H$_2$-), 29.8 (-**C**H$_2$-CH$_2$-O-), 24.3 (-**C**H$_2$-CH$_2$-CH$_2$-O-).

^{19}F{^{1}H}-NMR (470 MHz, Methanol-*d*$_4$) δ [ppm] = -76.98 (TFA).

LC-MS (ESI+) m/z: 877.6 [2M+H]$^+$, 439.4 [M+H]$^+$; t_R = 5.3 min.

3.6.10 (*S*)-3-(4-((5-((4,5-Dihydro-1H-imidazol-2-yl)amino)pentyl)oxy)phenyl)-2-(4-hydroxybenzamido)propanoic acid (**7ba**)

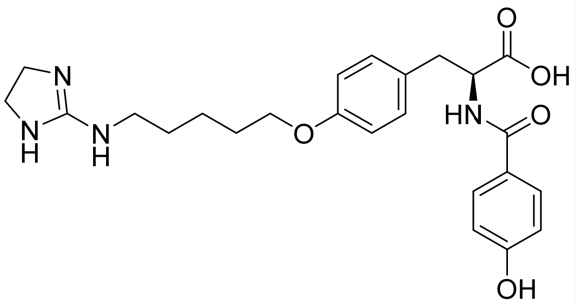

Synthesis according to **GP-5a**: Prepared from **7b**.

The final DHI substituted RGD mimetic **7ba** (8.3 mg, 14.6 µmol, 73 %) was isolated as a colourless TFA salt.

^{1}H-NMR (600 MHz, Methanol-d_4) δ [ppm] = 7.65 (d, ^{3}J = 8.7 Hz, 2H, Ar-2/2′-**H**), 7.19 (d, ^{3}J = 8.6 Hz, 2H, Tyr-2/2′-**H**), 6.83 (d, ^{3}J = 8.5 Hz, 2H, Tyr-3/3′-**H**), 6.81 (d, ^{3}J = 8.6 Hz, 2H, Ar-3/3′-**H**), 4.79 (dd, ^{3}J = 9.4 Hz, ^{3}J = 4.9 Hz, 1H, Tyr$^{\alpha}$-**H**), 3.96 (t, ^{3}J = 6.1 Hz, 2H, -C**H**$_2$-O-), 3.68 (s, 4H, DHI-4/5-**H**), 3.26 (dd, ^{2}J = 14.0 Hz, ^{3}J = 4.9 Hz, 1H, Tyr$^{\beta}$-**H**/H′), 3.20 (t, ^{3}J = 7.0 Hz, 2H, DHI-NH-C**H**$_2$-), 3.04 (dd, ^{3}J = 14.0 Hz, ^{3}J = 9.4 Hz, 1H, Tyr$^{\beta}$-H/**H′**), 1.84 – 1.73 (m, 2H, DHI-NH-CH$_2$-C**H**$_2$-), 1.69 – 1.61 (m, 2H, -C**H**$_2$-CH$_2$-O-), 1.59 – 1.47 (m, 2H, -C**H**$_2$-CH$_2$-CH$_2$-O-).

^{13}C{^{1}H}-NMR (151 MHz, Methanol-d_4) δ [ppm] = 175.1 (-**C**OOH), 169.9 (Ar-**C**ONH-), 162.2 (Ar-**C**4), 161.3 (DHI-**C**2), 159.3 (Tyr-**C**4), 131.3 (Tyr-**C**2/2′), 130.8 (Tyr-**C**1), 130.4 (Ar-**C**2/2′), 126.1 (Ar-**C**1), 116.0 (Ar-**C**3/3′), 115.5 (Tyr-**C**3/3′), 68.6 (-**C**H$_2$-O-), 55.8 (Tyr-**C**$^{\alpha}$), 44.0 (DHI-**C**4/5), 43.8 (DHI-NH-**C**H$_2$-), 37.5 (Tyr-**C**$^{\beta}$), 29.9 (DHI-NH-CH$_2$-**C**H$_2$-), 29.8 (-**C**H$_2$-CH$_2$-O-), 24.3 (-**C**H$_2$-CH$_2$-CH$_2$-O-).

^{19}F{^{1}H}-NMR (470 MHz, Methanol-d_4) δ [ppm] = -77.11 (TFA).

LC-MS (ESI+) m/z: 909.5 [2M+H]$^+$, 455.3 [M+H]$^+$; t_R = 4.8 min.

3.6.11 (*S*)-3-(4-((5-((4,5-Dihydro-1H-imidazol-2-yl)amino)pentyl)oxy)phenyl)-2-(3-hydroxybenzamido)propanoic acid (**7ca**)

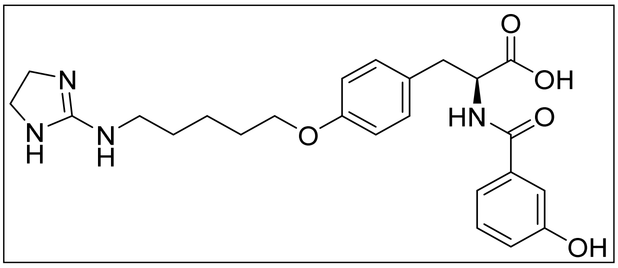

Synthesis according to **GP-5a**: Prepared from **7c**.

The final DHI substituted RGD mimetic **7ca** (6.3 mg, 11.1 µmol, 55 %) was isolated as a colourless TFA salt.

^{1}H-NMR (600 MHz, Methanol-d_4) δ [ppm] = 7.26 (dd, ^{3}J = 7.9 Hz, 1H, Ar-**C**5), 7.21 – 7.17 (m, 3H, Tyr-2/2′-**H**, Ar-**C**6), 7.16 (dd, ^{3}J = 2.5 Hz, ^{3}J = 1.0 Hz, 1H, Ar-**C**2), 6.95 (ddd, ^{3}J = 8.1 Hz, ^{4}J = 2.5 Hz, ^{4}J = 1.0 Hz, 1H, Ar-**C**4), 6.84 (d, ^{3}J = 8.6 Hz, 2H, Tyr-3/3′-**H**), 4.80 (dd, ^{3}J = 9.4 Hz, ^{3}J = 4.9 Hz, 1H, Tyr$^{\alpha}$-**H**), 3.97 (t, ^{3}J = 6.1 Hz, 2H, -C**H**$_2$-O-), 3.69 (s, 4H, DHI-4/5-**H**), 3.28 (dd, ^{2}J = 14.1 Hz, ^{3}J = 5.0 Hz, 1H, Tyr$^{\beta}$-**H**/H′), 3.21 (t, ^{3}J = 7.0 Hz, 2H,

DHI-NH-C**H**$_2$-), 3.05 (dd, ^2J = 14.0 Hz, ^3J = 9.4 Hz, 1H, Tyr^β-H/**H**′), 1.85 – 1.76 (m, 2H, DHI-NH-CH$_2$-C**H**$_2$-), 1.70 – 1.61 (m, 2H, -C**H**$_2$-CH$_2$-O-), 1.59 – 1.48 (m, 2H, -C**H**$_2$-CH$_2$-CH$_2$-O-).

^{13}C{^{1}H}-NMR (151 MHz, Methanol-*d*$_4$) δ [ppm] = 174.9 (-**C**OOH), 170.2 (Ar-**C**ONH-), 161.3 (DHI-**C**2), 159.4 (Tyr-**C**4), 158.8 (Ar-**C**3), 136.8 (Ar-**C**1), 131.3 (Tyr-**C**2/2′), 130.7 (Tyr-**C**1), 130.6 (Ar-**C**5), 119.7 (Ar-**C**4), 119.2 (Ar-**C**6), 115.5 (Tyr-**C**3/3′), 115.3 (Ar-**C**2), 68.6 (-**C**H$_2$-O-), 55.8 (Tyr-**C**^{α}), 44.0 (DHI-**C**4/5), 43.8 (DHI-NH-**C**H$_2$-), 37.4 (Tyr-**C**^{β}), 29.9 (DHI-NH-CH$_2$-**C**H$_2$-), 29.8 (-**C**H$_2$-CH$_2$-O-), 24.3 (-**C**H$_2$-CH$_2$-CH$_2$-O-).

^{19}F{^{1}H}-NMR (470 MHz, Methanol-*d*$_4$) δ [ppm] = -77.08 (TFA).

LC-MS (ESI+) m/z: 909.5 [2M+H]$^+$, 455.3 [M+H]$^+$; t$_R$ = 4.9 min.

3.6.12 (*S*)-2-(((Benzyloxy)carbonyl)amino)-3-(4-((5-((4,5-dihydro-1H-imidazol-2-yl)amino)-pentyl)oxy)phenyl)propanoic acid (**7da**)

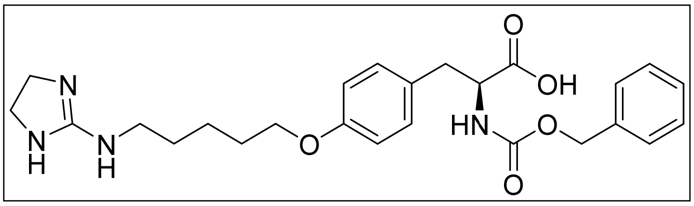

Synthesis according to **GP-5a**: Prepared from **3c**.

The final DHI substituted RGD mimetic **7da** (31.6 mg, 37.0 µmol, 62 %) was isolated as a colourless TFA salt.

^{1}H-NMR (600 MHz, Methanol-*d*$_4$) δ [ppm] = 7.33 – 7.24 (m, 5H, Cbz-**H**), 7.11 (d, ^3J = 8.1 Hz, 2H, Tyr-2/2′-**H**), 6.79 (d, ^3J = 8.1 Hz, 2H, Tyr-3/3′-**H**), 5.04 (d, ^2J = 12.7 Hz, 1H, Cbz-C**H**$_2$-), 4.99 (d, ^2J = 12.4 Hz, 1H, Cbz-C**H**$_2$-), 4.36 (dd, ^3J = 9.4 Hz, ^3J = 4.8 Hz, 1H, Tyr^α-**H**), 3.95 (t, ^3J = 6.2 Hz, 2H, -C**H**$_2$-O-Tyr), 3.66 (s, 4H, DHI-4/5-**H**), 3.20 (t, ^3J = 7.0 Hz, 2H, DHI-NH-C**H**$_2$-), 3.12 (dd, ^2J = 14.0 Hz, ^3J = 4.9 Hz, 1H, Tyr^β-**H**/H′), 2.85 (dd, ^2J = 14.2 Hz, ^3J = 9.3 Hz, 1H, Tyr^β-H/**H**′), 1.84 – 1.74 (m, 2H, DHI-NH-CH$_2$-C**H**$_2$-), 1.68 – 1.60 (m, 2H, -C**H**$_2$-CH$_2$-O-), 1.60 – 1.47 (m, 2H, DHI-NH-CH$_2$-CH$_2$-C**H**$_2$-).

^{13}C{^{1}H}-NMR (151 MHz, Methanol-*d*$_4$) δ [ppm] = 175.2 (-**C**OOH), 161.3 (DHI-**C**2), 159.3 (Tyr-**C**4), 158.4 (BnO**C**ONH-), 138.3 (Ar-**C**1), 131.3 (Tyr-**C**2/2′), 130.5 (Tyr-**C**1), 129.4 (Ar-**C**3/3′), 128.9 (Ar-**C**4), 128.6 (Ar-**C**2/2′), 115.4 (Tyr-**C**3/3′), 68.6 (-**C**H$_2$-O-Tyr), 67.4 (Cbz-**C**H$_2$-), 57.0 (Tyr-**C**^{α}), 44.0 (DHI-**C**4/5), 43.8 (DHI-NH-**C**H$_2$-), 37.8 (Tyr-**C**^{β}), 29.9 (-**C**H$_2$-CH$_2$-O-Tyr), 29.9 (DHI-NH-CH$_2$-**C**H$_2$-), 24.3 (DHI-NH-CH$_2$-CH$_2$-CH$_2$-**C**H$_2$-).

^{19}F{^{1}H}-NMR (470 MHz, Methanol-*d*$_4$) δ [ppm] = -76.96 (TFA).

LC-MS (ESI+) m/z: 937.5 [2M+H]$^+$, 469.4 [M+H]$^+$; t$_R$ = 5.6 min.

3.6.13 (*S*)-2-Benzamido-3-(4-(3-(pyrimidin-2-ylamino)propoxy)phenyl)propanoic acid (**5ab**)

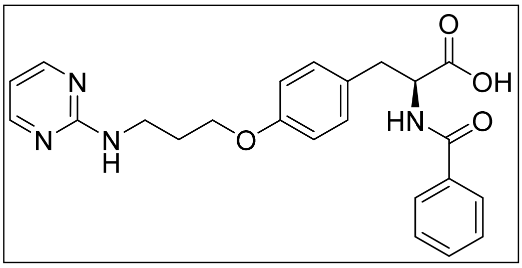

Synthesis according to **GP-5b**: Prepared from **5a**.

The final Pyr substituted RGD mimetic **5ab** (2.7 mg, 6.4 µmol, 43 %) was isolated as colourless solid.

^{1}H-NMR (600 MHz, Methanol-d_4) δ [ppm] = 8.39 (d, ^3J = 5.1 Hz, 2H, Pyr-4/6-**H**), 7.75 (d, ^3J = 8.6 Hz, 1H, Ar-2/2′-**H**), 7.54 (t, ^3J = 7.5 Hz, 1H, Ar-4-**H**), 7.45 (dd, ^3J = 8.5 Hz, ^3J = 7.5 Hz, 2H, Ar-3/3′-**H**), 7.20 (d, ^3J = 8.6 Hz, 2H, Tyr-2/2′-**H**), 6.85 (d, ^3J = 8.7 Hz, 2H, Tyr-3/3′-**H**), 6.77 (t, ^3J = 5.1 Hz, 1H, Pyr-5-**H**), 4.82 (dd, ^3J = 9.6 Hz, ^3J = 4.9 Hz, 1H, Tyr^α-**H**), 4.07 (t, ^3J = 5.9 Hz, 2H, -C**H**$_2$-O-), 3.65 (t, ^3J = 6.7 Hz, 2H, Pyr-NH-C**H**$_2$-), 3.29 (dd, ^2J = 14.0 Hz, ^3J = 4.9 Hz, 1H, Tyr^β-**H**/H′), 3.06 (dd, ^2J = 14.0 Hz, ^3J = 9.5 Hz, 1H, Tyr^β-H/**H′**), 2.11 (tt, ^3J = 6.7 Hz, ^3J = 5.9 Hz, 2H, -CH$_2$-C**H**$_2$-CH$_2$-).

^{13}C{^{1}H}-NMR (151 MHz, Methanol-d_4) δ [ppm] = 174.8 (-**C**OOH), 170.2 (Ar-**C**ONH-), 159.2 (Pyr-**C**2), 159.2 (Tyr-**C**4), 135.4 (Ar-**C**1), 132.8 (Ar-**C**4), 131.3 (Tyr-**C**2/2′), 130.9 (Tyr-**C**1), 129.5 (Ar-**C**3/3′), 128.4 (Ar-**C**2/2′), 115.5 (Tyr-**C**3/3′), 110.9 (Pyr-**C**5), 66.5 (-**C**H$_2$-O-), 55.8 (Tyr-**C**^{α}), 39.9 (Pyr-NH-**C**H$_2$-), 37.5 (Tyr-**C**^{β}), 29.8 (-CH$_2$-**C**H$_2$-CH$_2$-).

LC-MS (ESI+) m/z: 863.3 [2M+Na]$^+$, 840.5 [2M+H]$^+$, 421.2 [M+H]$^+$; t_R = 6.5 min.

3.6.14 (*S*)-2-(4-Hydroxybenzamido)-3-(4-(3-(pyrimidin-2-ylamino)propoxy)phenyl)-propanoic acid (**5bb**)

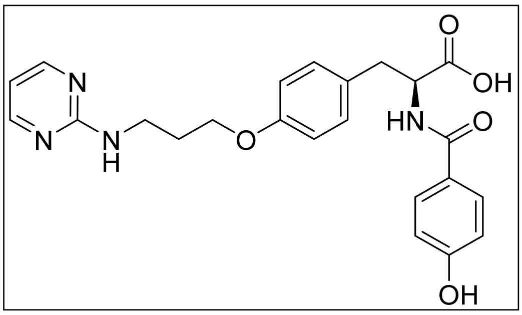

Synthesis according to **GP-5b**: Prepared from **5b**.

The final Pyr substituted RGD mimetic **5bb** (2.5 mg, 5.6 µmol, 38 %) was isolated as colourless solid.

^{1}H-NMR (600 MHz, Methanol-d_4) δ [ppm] = 8.38 (d, ^3J = 5.2 Hz, 2H, Pyr-4/6-**H**), 7.65 (d, ^3J = 8.7 Hz, 2H, Ar-2/2′-**H**), 7.19 (d, ^3J = 8.7 Hz, 2H, Tyr-2/2′-**H**), 6.84 (d, ^3J = 8.7 Hz, 2H, Tyr-3/3′-**H**), 6.82 (d, ^3J = 8.7 Hz, 2H, Ar-3/3′-**H**), 6.75 (t, ^3J = 5.1 Hz, 1H, Pyr-5-**H**), 4.79 (dd, ^3J = 9.4 Hz, ^3J = 4.9 Hz, 1H, Tyr^α-**H**), 4.07 (t, ^3J = 5.9 Hz, 2H, -C**H**$_2$-O-), 3.64 (t, ^3J = 6.7 Hz, 2H, Pyr-NH-C**H**$_2$-), 3.27 (dd, ^2J = 14.0 Hz, ^3J = 5.0 Hz, 1H, Tyr^β-**H**/H′), 3.05 (dd, ^2J = 14.0 Hz, ^3J = 9.3 Hz, 1H, Tyr^β-H/**H′**), 2.11 (tt, ^3J = 6.7 Hz, ^3J = 5.9 Hz, 2H, -CH$_2$-C**H**$_2$-CH$_2$-).

^{13}C{^{1}H}-NMR (151 MHz, Methanol-d_4) δ [ppm] = 175.1 (-**C**OOH), 169.8 (Ar-**C**ONH-), 162.2 (Ar-**C**4), 159.3 (Pyr-**C**2), 159.2 (Tyr-**C**4), 131.3 (Tyr-**C**2/2′), 130.9 (Tyr-**C**1), 130.4 (Ar-**C**2/2′), 126. (Ar-**C**1), 116.1 (Ar-**C**3/3′), 115.5 (Tyr-**C**3/3′), 111.0 (Pyr-**C**5), 66.6 (-**C**H$_2$-O-), 55.8 (Tyr-**C**^{α}), 39.8 (Pyr-NH-**C**H$_2$-), 37.5 (Tyr-**C**^{β}), 29.8 (-CH$_2$-**C**H$_2$-CH$_2$-).

LC-MS (ESI+) m/z: 437.2 $[M+H]^+$; t_R = 5.8 min.

3.6.15 (*S*)-2-Benzamido-3-(4-(4-(pyrimidin-2-ylamino)butoxy)phenyl)propanoic acid (**5cb**)

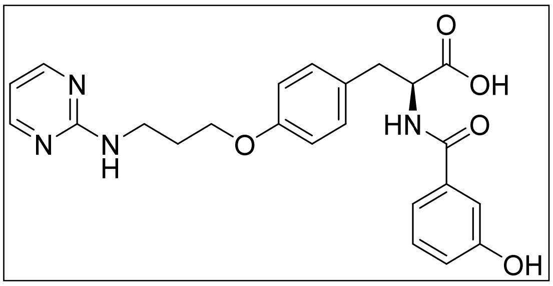

Synthesis according to **GP-5b**: Prepared from **5c**.

The final Pyr substituted RGD mimetic **5cb** (2.8 mg, 6.3 μmol, 38 %) was isolated as a colourless solid.

^{1}H-NMR (600 MHz, Methanol-*d*$_4$) δ [ppm] = 8.39 (d, ^{3}J = 5.1 Hz, 2H, Pyr-4/6-**H**), 7.26 (dd, ^{3}J = 8.0 Hz, ^{3}J = 7.9 Hz, 1H, Ar-5-**H**), 7.22 – 7.17 (m, 3H, Tyr-2/2´-**H**, Ar-6-**H**), 7.16 (dd, ^{4}J = 2.5 Hz, ^{4}J = 1.1 Hz, 1H, Ar-2-**H**), 6.95 (ddd, ^{3}J = 8.0 Hz, ^{4}J = 2.5 Hz, ^{4}J = 1.1 Hz, 1H, Ar-4-**H**), 6.85 (d, ^{3}J = 8.6 Hz, 2H, Tyr-3/3´-**H**), 6.77 (t, ^{3}J = 5.2 Hz, 1H, Pyr-5-**H**), 4.80 (dd, ^{3}J = 9.4 Hz, ^{3}J = 4.9 Hz, 1H, Tyr$^{\alpha}$-**H**), 4.07 (t, ^{3}J = 5.9 Hz, 2H, -C**H**$_2$-O-), 3.66 (t, ^{3}J = 6.7 Hz, 2H, Pyr-NH-C**H**$_2$-), 3.28 (dd, ^{2}J = 14.0 Hz, ^{3}J = 4.9 Hz, 1H, Tyr$^{\beta}$-**H**/H´), 3.05 (dd, ^{3}J = 14.0 Hz, ^{3}J = 9.4 Hz, 1H, Tyr$^{\beta}$-H/**H´**), 2.11 (tt, ^{3}J = 6.7 Hz, ^{3}J = 5.9 Hz, 2H, -CH$_2$-C**H**$_2$-CH$_2$-).

^{13}C{^{1}H}-NMR (151 MHz, Methanol-*d*$_4$) δ [ppm] = 174.8 (-**C**OOH), 170.2 (Ar-**C**ONH-), 159.2 (Pyr-**C**2), 159.2 (Tyr-**C**4), 158.8 (Ar-**C**3), 136.8 (Ar-**C**1), 131.3 (Tyr-**C**2/2´), 130.9 (Tyr-**C**1), 130.6 (Ar-**C**5), 119.7 (Ar-**C**4), 119.2 (Ar-**C**6), 115.5 (Tyr-**C**3/3´), 115.3 (Ar-**C**2), 110.9 (Pyr-**C**5), 66.5 (-**C**H$_2$-O-), 55.8 (Tyr-**C**$^{\alpha}$), 39.9 (Pyr-NH-**C**H$_2$-), 37.4 (Tyr-**C**$^{\beta}$), 29.8 (-CH$_2$-**C**H$_2$-CH$_2$-).

LC-MS (ESI+) m/z: 437.2 $[M+H]^+$; t_R = 5.9 min.

3.6.16 (*S*)-2-(((benzyloxy)carbonyl)amino)-3-(4-(3-(pyrimidin-2-ylamino)propoxy)phenyl)-propanoic acid (**5db**)

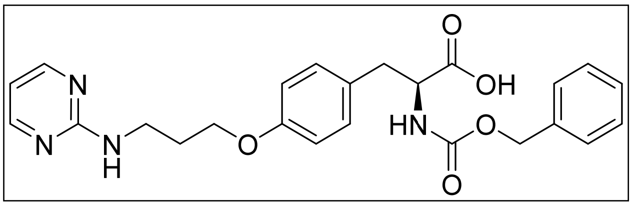

Synthesis according to **GP-5b**: Prepared from **3a**.

The final DHI substituted RGD mimetic **5db** (20.0 mg, 29.9 μmol, 57 %) was isolated as colourless solid.

^{1}H-NMR (600 MHz, Methanol-*d*$_4$) δ [ppm] = 8.41 (d, ^{3}J = 5.2 Hz, 2H Pyr-4/6-**H**), 7.33 – 7.24 (m, 5H, Cbz-**H**), 7.11 (d, ^{3}J = 8.5 Hz, 2H, Tyr-2/2´-**H**), 6.83 – 6.77 (m, 3H, Tyr-3/3´-**H**, Pyr-5-**H**), 5.05 (d, ^{2}J = 12.6 Hz, 1H, Cbz-C**H**$_2$-), 5.01 (d, ^{2}J = 12.5 Hz, 1H, Cbz-C**H**$_2$-), 4.36 (dd, ^{3}J = 9.2 Hz, ^{3}J = 4.9 Hz, 1H, Tyr$^{\alpha}$-**H**), 4.06 (t, ^{3}J = 5.9 Hz, 2H, -C**H**$_2$-O-Tyr), 3.67 (t, ^{3}J = 6.8 Hz, 2H, Pyr-NH-C**H**$_2$-), 3.12 (dd, ^{2}J = 14.0 Hz, ^{3}J = 4.9 Hz, 1H, Tyr$^{\beta}$-**H**/H´), 2.85 (dd, ^{2}J = 14.0 Hz, ^{3}J = 9.2 Hz, 1H, Tyr$^{\beta}$-H/**H´**), 2.11 (t, ^{3}J = 6.8 Hz, ^{3}J = 5.9 Hz, 2H, -CH$_2$-C**H**$_2$-CH$_2$-).

^{13}C{^{1}H}-NMR (151 MHz, Methanol-d_4) δ [ppm] = 175.1 (-**C**OOH), 159.1 (Tyr-**C**4), 158.7 (Pyr-**C**2), 158.4 (BnO**C**ONH), 138.3 (Ar-**C**1), 131.3 (Tyr-**C**2/2´), 130.7 (Tyr-**C**1), 129.4 (Ar-**C**3/3´), 128.9 (Ar-**C**4), 128.6 (Ar-**C**2/2´), 115.4 (Tyr-**C**3/3´), 110.9 (Pyr-**C**5), 67.5 (Cbz-**C**H$_2$-), 66.5 (-**C**H$_2$-O-Tyr), 57.0 (Tyr-**C**$^{\alpha}$), 39.9 (Pyr-NH-**C**H$_2$-), 37.8 (Tyr-**C**$^{\beta}$), 29.7 (-CH$_2$-**C**H$_2$-CH$_2$-).

LC-MS (ESI+) m/z: 451.3 [M+H]$^+$; t_R = 7.5 min.

3.6.17 (*S*)-2-Benzamido-3-(4-(4-(pyrimidin-2-ylamino)butoxy)phenyl)propanoic acid (**6ab**)

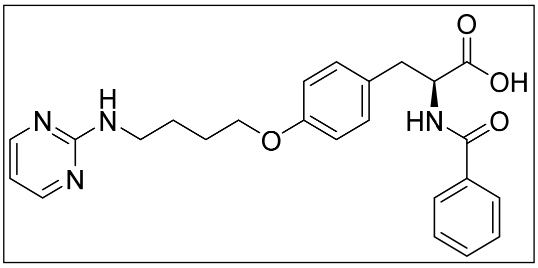

Synthesis according to **GP-5b**: Prepared from **6a**.

The final Pyr substituted RGD mimetic **6ab** (2.7 mg, 6.3 µmol, 42 %) was isolated as a colourless solid.

^{1}H-NMR (600 MHz, Methanol-d_4) δ [ppm] = 8.43 (d, ^3J = 5.2 Hz, 2H, Pyr-4/6-**H**), 7.74 (d, ^3J = 8.5 Hz, 2H, Ar-2/2´-**H**), 7.53 (t, ^3J = 7.4 Hz, 1H, Ar-4-**H**), 7.45 (dd, ^3J = 8.5 Hz, ^3J = 7.4 Hz, 2H, Ar-3/3´-**H**), 7.19 (d, ^3J = 8.5 Hz, 2H, Tyr-2/2´-**H**), 6.84 (d, ^3J = 8.6 Hz, 2H, Tyr-3/3´-**H**), 6.81 (t, ^3J = 5.2 Hz, 1H, Pyr-5-**H**), 4.82 (dd, ^3J = 9.5 Hz, ^3J = 5.0 Hz, 1H, Tyr$^{\alpha}$-**H**), 4.00 (t, ^3J = 5.7 Hz, 2H, -C**H**$_2$-O-), 3.51 (t, ^3J = 6.6 Hz, 2H, Pyr-NH-C**H**$_2$-), 3.31 – 3.23 (m, 1H, Tyr$^{\beta}$-**H**/H´), 3.06 (dd, ^2J = 14.1 Hz, ^3J = 9.5 Hz, 1H, Tyr$^{\beta}$-H/**H**´), 1.91 – 1.76 (m, 4H, -CH$_2$-C**H**$_2$-C**H**$_2$-CH$_2$-).

^{13}C{^{1}H}-NMR (151 MHz, Methanol-d_4) δ [ppm] = 174.9 (-**C**OOH), 170.2 (Ar-**C**ONH-), 159.3 (Tyr-**C**4), 159.0 (Pyr-**C**2), 135.4 (Ar-**C**1), 132.8 (Ar-**C**4), 131.3 (Tyr-**C**2/2´), 130.7 (Tyr-**C**1), 129.5 (Ar-**C**3/3´), 128.4 (Ar-**C**2/2´), 115.5 (Tyr-**C**3/3´), 110.9 (Pyr-**C**5), 68.5 (-**C**H$_2$-O-), 55.8 (Tyr-**C**$^{\alpha}$), 42.3 (Pyr-NH-**C**H$_2$-), 37.4 (Tyr-**C**$^{\beta}$), 27.7 (-CH$_2$-**C**H$_2$-CH$_2$-CH$_2$-), 26.7 (-CH$_2$-CH$_2$-**C**H$_2$-CH$_2$-).

LC-MS (ESI+) m/z: 435.1 [M+H]$^+$; t_R = 6.8 min.

3.6.18 (*S*)-2-(4-Hydroxybenzamido)-3-(4-(4-(pyrimidin-2-ylamino)butoxy)phenyl)propanoic acid (**6bb**)

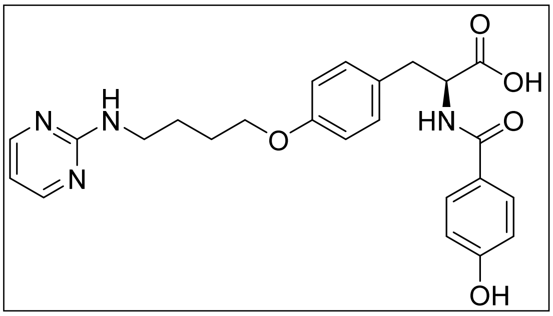

Synthesis according to **GP-5b**: Prepared from **6b**.

The final Pyr substituted RGD mimetic **6bb** (3.5 mg, 7.8 µmol, 78 %) was isolated as a colourless solid.

^{1}H-NMR (600 MHz, Methanol-d_4) δ [ppm] = 8.43 (d, ^3J = 5.2 Hz, 2H, Pyr-4/6-**H**), 7.64 (d, ^3J = 8.7 Hz, 2H, Ar-2/2´-**H**), 7.18 (d, ^3J = 8.6 Hz, 1H, Tyr-2/2´-**H**), 6.83

(d, ^3J = 8.7 Hz, 1H, Tyr-3/3′-**H**), 6.82 – 6.80 (m, 3H, Ar-3/3′-**H**, Pyr-5-**H**), 4.78 (dd, ^3J = 9.3 Hz, ^3J = 5.0 Hz, 1H, Tyr^α-**H**), 4.00 (t, ^3J = 5.7 Hz, 2H, -C**H**$_2$-O-), 3.51 (t, ^3J = 6.6 Hz, 2H, Pyr-NH-C**H**$_2$-), 3.26 (dd, ^2J = 14.0 Hz, ^3J = 5.0 Hz, 1H, Tyr^β-**H**/H′), 3.05 (dd, ^2J = 14.0 Hz, ^3J = 9.3 Hz, 1H, Tyr^β-H/**H′**), 1.92 – 1.77 (m, 4H, -CH$_2$-C**H**$_2$-C**H**$_2$-CH$_2$-).

^{13}C{^{1}H}-NMR (151 MHz, Methanol-*d*$_4$) δ [ppm] = 175.1 (-**C**OOH), 169.9 (Ar-**C**ONH-), 162.2 (Ar-**C**4), 159.3 (Tyr-**C**4), 158.9 (Pyr-**C**2), 131.3 (Tyr-**C**2/2′), 130.8 (Tyr-**C**1), 130.4 (Ar-**C**2/2′), 126.1 (Ar-**C**1), 116.0 (Ar-**C**3/3′), 115.5 (Tyr-**C**3/3′), 110.8 (Pyr-**C**5), 68.5 (-**C**H$_2$-O-), 55.8 (Tyr-**C**^{α}), 42.3 (Pyr-NH-**C**H$_2$-), 37.5 (Tyr-**C**^{β}), 27.6 (-CH$_2$-**C**H$_2$-CH$_2$-CH$_2$-), 26.7 (-CH$_2$-CH$_2$-**C**H$_2$-CH$_2$-).

LC-MS (ESI+) m/z: 451.1 [M+H]$^+$; t$_R$ = 6.1 min.

3.6.19 (*S*)-2-(3-Hydroxybenzamido)-3-(4-(4-(pyrimidin-2-ylamino)butoxy)phenyl)propanoic acid (**6cb**)

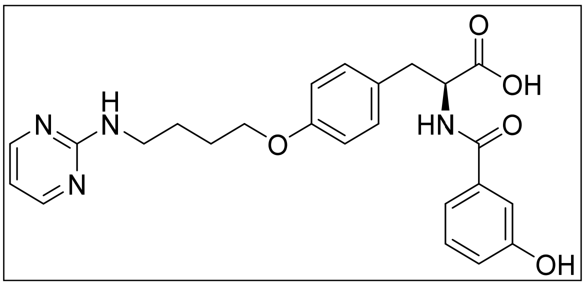

Synthesis according to **GP-5b**: Prepared from **6c**.

The final Pyr substituted RGD mimetic **6cb** (2.7 mg, 5.9 µmol, 39 %) was isolated as a colourless solid.

^{1}H-NMR (600 MHz, Methanol-*d*$_4$) δ [ppm] = 8.43 (d, ^3J = 5.2 Hz, 2H, Pyr-4/6-**H**), 7.25 (dd, ^3J = 8.1 Hz, ^3J = 7.9 Hz, 1H, Ar-5-**H**), 7.21 – 7.16 (m, 3H, Tyr-2/2′-**H**, Ar-6-**H**), 7.15 (dd, ^4J = 2.6 Hz, ^4J = 1.0 Hz, 1H, Ar-2-**H**), 6.94 (ddd, ^3J = 8.1 Hz, ^2J = 2.6 Hz, ^2J = 1.0 Hz, 1H, Ar-4-**H**), 6.84 (d, ^3J = 8.6 Hz, 2H, Tyr-3/3′-**H**), 6.81 (t, ^3J = 5.2 Hz, 1H, Pyr-5-**H**), 4.79 (dd, ^3J = 9.3 Hz, ^3J = 4.9 Hz, 1H, Tyr^α-**H**), 4.01 (t, ^3J = 5.7 Hz, 2H, -C**H**$_2$-O-), 3.51 (t, ^3J = 6.6 Hz, 2H, Pyr-NH-C**H**$_2$-), 3.27 (dd, ^2J = 14.1 Hz, ^3J = 5.0 Hz, 1H, Tyr^β-**H**/H′), 3.05 (dd, ^3J = 14.0 Hz, ^3J = 9.3 Hz, 1H, Tyr^β-H/**H′**), 2.00 – 1.76 (m, 4H, -CH$_2$-C**H**$_2$-C**H**$_2$-CH$_2$-).

^{13}C{^{1}H}-NMR (151 MHz, Methanol-*d*$_4$) δ [ppm] = 174.8 (-**C**OOH), 170.2 (Ar-**C**ONH-), 159.3 (Tyr-**C**4), 158.9 (Pyr-**C**2), 158.8 (Ar-**C**3), 136.8 (Ar-**C**1), 131.3 (Tyr-**C**2/2′), 130.7 (Tyr-**C**1), 130.6 (Ar-**C**5), 119.7 (Ar-**C**4), 119.2 (Ar-**C**6), 115.5 (Tyr-**C**3/3′), 115.3 (Ar-**C**2), 110.8 (Pyr-**C**5), 68.5 (-**C**H$_2$-O-), 55.8 (Tyr-**C**^{α}), 42.3 (Pyr-NH-**C**H$_2$-), 37.4 (Tyr-**C**^{β}), 27.6 (-CH$_2$-**C**H$_2$-CH$_2$-CH$_2$-), 26.7 (-CH$_2$-CH$_2$-**C**H$_2$-CH$_2$-).

LC-MS (ESI+) m/z: 451.1 [M+H]$^+$; t$_R$ = 6.2 min.

3.6.20 (*S*)-2-(((Benzyloxy)carbonyl)amino)-3-(4-(3-(pyrimidin-2-ylamino)butoxy)phenyl)-propanoic acid (**6db**)

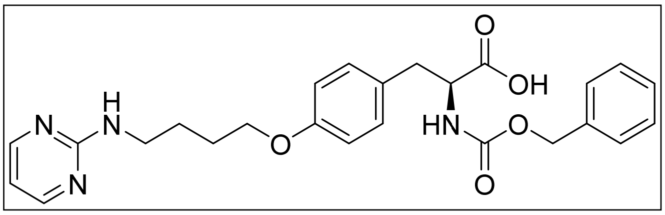

Synthesis according to **GP-5b**: Prepared from **3b**.

The final DHI substituted RGD mimetic **6db** (89.9 mg, 193.5 µmol, 70 %) was isolated as colourless solid.

^{1}H-NMR (600 MHz, Methanol-d_4) δ [ppm] = 8.44 (d, ^3J = 5.1 Hz, 2H, Pyr-4/6-**H**), 7.36 – 7.27 (m, 5H, Cbz-**H**), 7.14 (d, ^3J = 8.2 Hz, 2H, Tyr-2/2′-**H**), 6.87 – 6.76 (m, 3H, Tyr-3/3′-**H**, Pyr-5-**H**), 5.08 (d, ^2J = 12.7 Hz, 1H, Cbz-C**H**$_2$-), 5.03 (d, ^2J = 12.4 Hz, 1H, Cbz-C**H**$_2$-), 4.40 (dd, ^3J = 9.3 Hz, ^3J = 4.9 Hz, 1H, Tyr$^\alpha$-**H**), 4.02 (t, ^3J = 5.6 Hz, 2H, -C**H**$_2$-O-Tyr), 3.54 (t, ^3J = 6.4 Hz, 2H, Pyr-NH-C**H**$_2$-), 3.15 (dd, ^2J = 14.1 Hz, ^3J = 4.9 Hz, 1H, Tyr$^\beta$-**H**/H′), 2.88 (dd, ^2J = 14.0 Hz, ^3J = 9.1 Hz, 1H, Tyr$^\beta$-H/**H**′), 1.93 – 1.83 (m, 4H, -CH$_2$-C**H**$_2$-C**H**$_2$-CH$_2$-).

^{13}C{^{1}H}-NMR (151 MHz, Methanol-d_4) δ [ppm] = 175.2 (-**C**OOH), 159.3 (Tyr-**C**4), 159.3 (Pyr-**C**2), 158.4 (BnO**C**ONH), 138.3 (Ar-**C**1), 131.3 (Tyr-**C**2/2′), 130.5 (Tyr-**C**1), 129.4 (Ar-**C**3/3′), 128.9 (Ar-**C**4), 128.6 (Ar-**C**2/2′), 115.5 (Tyr-**C**3/3′), 110.9 (Pyr-**C**5), 68.5 (-**C**H$_2$-O-Tyr), 67.5 (Cbz-**C**H$_2$-), 57.0 (Tyr-**C**$^\alpha$), 42.3 (Pyr-NH-**C**H$_2$-), 37.9 (Tyr-**C**$^\beta$), 27.7 (Pyr-NH-CH$_2$-**C**H$_2$-), 26.8 (-**C**H$_2$-CH$_2$-O-Tyr).

LC-MS (ESI+) m/z: 465.3 [M+H]$^+$; t_R = 7.8 min.

3.6.21 (*S*)-2-Benzamido-3-(4-((5-(pyrimidin-2-ylamino)pentyl)oxy)phenyl)propanoic acid (**7ab**)

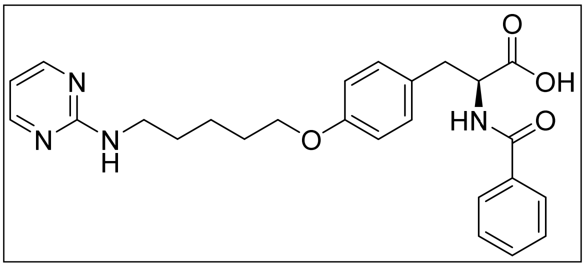

Synthesis according to **GP-5b**: Prepared from **7a**.

The final Pyr substituted RGD mimetic **7ab** (37.5 mg, 83.5 µmol, 37 %) was isolated as a colourless solid.

^{1}H-NMR (600 MHz, Methanol-d_4) δ [ppm] = 8.39 (d, ^3J = 5.2 Hz, 2H, Pyr-4/6-**H**), 7.73 (d, ^3J = 8.6 Hz, 1H, Ar-2/2′-**H**), 7.52 (t, ^3J = 7.4 Hz, 1H, Ar-4-**H**), 7.43 (dd, ^3J = 8.6 Hz, ^3J = 7.4 Hz, 2H, Ar-3/3′-**H**), 7.18 (d, ^3J = 8.6 Hz, 2H, Tyr-**C**2/2′), 6.81 (d, ^3J = 8.6 Hz, 2H, Tyr-**C**3/3′), 6.76 (t, ^3J = 5.1 Hz, 1H, Pyr-5-**H**), 4.81 (dd, ^3J = 9.5 Hz, ^3J = 5.0 Hz, 1H, Tyr$^\alpha$-**H**), 3.95 (t, ^3J = 6.3 Hz, 2H, -C**H**$_2$-O-), 3.44 (t, ^3J = 7.1 Hz, 2H, Pyr-NH-C**H**$_2$-), 3.27 (dd, ^3J = 14.1 Hz, ^3J = 5.0 Hz, 1H, Tyr$^\beta$-**H**/H′), 3.05 (dd, ^3J = 14.0 Hz, ^3J = 9.5 Hz, 1H, Tyr$^\beta$-H/**H**′), 1.85 – 1.75 (m, 2H, -C**H**$_2$-CH$_2$-O-), 1.77 – 1.66 (m, 2H, Pyr-NH-CH$_2$-C**H**$_2$-), 1.61 – 1.52 (m, 2H, -C**H**$_2$-CH$_2$-CH$_2$-O-).

^{13}C{^{1}H}-NMR (151 MHz, Methanol-d_4) δ [ppm] = 174.9 (-**C**OOH), 170.2 (Ar-**C**ONH-), 159.5 (Tyr-**C**4), 159.4 (Pyr-**C**2), 135.4 (Ar-**C**1), 132.8 (Ar-**C**4), 131.2 (Tyr-**C**2/2′), 130.6 (Tyr-**C**1), 129.5 (Ar-**C**3/3′), 128.4 (Ar-**C**2/2′), 115.5 (Tyr-**C**3/3′), 110.8 (Pyr-**C**5), 68.7 (-**C**H$_2$-O-), 55.8 (Tyr-**C**$^{\alpha}$), 42.4 (Pyr-NH-**C**H$_2$-), 37.4 (Tyr-**C**$^{\beta}$), 30.0 (-**C**H$_2$-CH$_2$-O-), 29.7 (Pyr-NH-CH$_2$-**C**H$_2$-), 24.4 (-**C**H$_2$-CH$_2$-CH$_2$-O-).

LC-MS (ESI+) m/z: 449.3 [M+H]$^+$; t$_R$ = 7.2 min.

3.6.22 (*S*)-2-(4-Hydroxybenzamido)-3-(4-((5-(pyrimidin-2-ylamino)pentyl)oxy)phenyl)-propanoic acid (**7bb**)

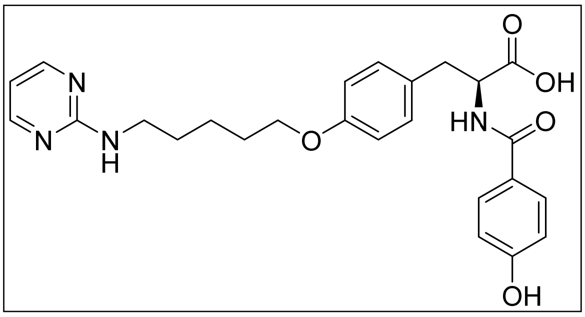

Synthesis according to **GP-5b**: Prepared from **7b**.

The final Pyr substituted RGD mimetic **7bb** (4.1 mg, 8.7 µmol, 51 %) was isolated as a colourless solid.

^{1}H-NMR (600 MHz, Methanol-d_4) δ [ppm] = 8.44 (d, ^3J = 5.2 Hz, 2H, Pyr-4/6-**H**), 7.64 (d, ^3J = 8.7 Hz, 2H, Ar-2/2′-**H**), 7.18 (d, ^3J = 8.6 Hz, 2H, Tyr-2/2′-**H**), 6.85 – 6.78 (m, 5H, Ar-3/3′-**H**, Tyr-3/3′-**H**, Pyr-5-**H**), 4.79 (dd, ^3J = 9.2 Hz, ^3J = 5.0 Hz, 1H, Tyr$^{\alpha}$-**H**), 3.96 (t, ^3J = 6.2 Hz, 2H, -C**H**$_2$-O-), 3.47 (t, ^3J = 7.1 Hz, 2H, Pyr-NH-C**H**$_2$-), 3.26 (dd, ^3J = 14.0 Hz, ^3J = 5.0 Hz, 1H, Tyr$^{\beta}$-**H**/H′), 3.05 (dd, ^3J = 14.0 Hz, ^3J = 9.3 Hz, 1H, Tyr$^{\beta}$-H/**H′**), 1.86 – 1.77 (m, 2H, -C**H**$_2$-CH$_2$-O-), 1.76 – 1.68 (m, 2H, DHI-NH-CH$_2$-C**H**$_2$-), 1.62 – 1.52 (m, 2H, -C**H**$_2$-CH$_2$-CH$_2$-O-).

^{13}C{^{1}H}-NMR (151 MHz, Methanol-d_4) δ [ppm] = 175.1 (-**C**OOH), 169.9 (Ar-**C**ONH-), 162.2 (Ar-**C**4), 159.4 (Tyr-**C**4), 158.5 (Pyr-**C**2), 131.3 (Tyr-**C**2/2′), 130.7 (Tyr-**C**1), 130.4 (Ar-**C**2/2′), 126.1 (Ar-**C**1), 116.0 (Ar-**C**3/3′), 115.5 (Tyr-**C**3/3′), 110.8 (Pyr-**C**5), 68.7 (-**C**H$_2$-O-), 55.8 (Tyr-**C**$^{\alpha}$), 42.5 (Pyr-NH-**C**H$_2$-), 37.5 (Tyr-**C**$^{\beta}$), 30.0 (-**C**H$_2$-CH$_2$-O-), 29.5 (Pyr-NH-CH$_2$-**C**H$_2$-), 24.4 (-**C**H$_2$-CH$_2$-CH$_2$-O-).

LC-MS (ESI+) m/z: 465.3 [M+H]$^+$; t$_R$ = 6.3 min.

3.6.23 (*S*)-2-(3-Hydroxybenzamido)-3-(4-((5-(pyrimidin-2-ylamino)pentyl)oxy)phenyl)-propanoic acid (**7cb**)

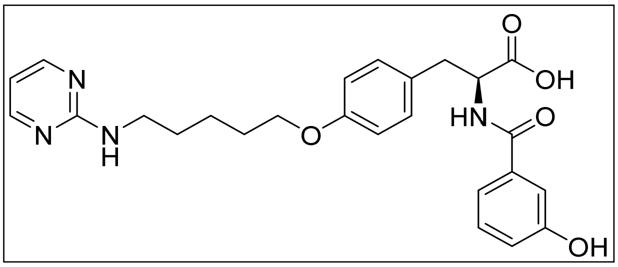

Synthesis according to **GP-5b**: Prepared from **7c**.

The final Pyr substituted RGD mimetic **7cb** (1.1 mg, 2.4 µmol, 12 %) was isolated as a colourless solid.

^{1}H-NMR (600 MHz, Methanol-d_4) δ [ppm] = 8.43 (d, ^3J = 5.2 Hz, 2H, Pyr-4/6-**H**), 7.25 (dd, ^3J = 7.9 Hz, 1H, Ar-**C**5), 7.20 - 7.17 (m, 3H, Tyr-2/2′-**H**, Ar-6-**H**), 7.15 (dd, ^4J = 2.5 Hz, ^4J = 1.0 Hz, 1H, Ar-2-**H**), 6.94 (ddd, ^3J = 8.1 Hz, ^3J = 2.5 Hz, ^3J = 1.0 Hz, 1H, Ar-4-**H**), 6.83 (d, ^3J = 8.8 Hz, 2H, Tyr-3/3′-**H**), 6.81 (t, ^3J = 5.3 Hz, 1H, Pyr-5-**H**), 4.80 (dd, ^3J = 9.3 Hz, ^3J = 4.9 Hz, 1H, Tyr$^{\alpha}$-**H**), 3.97 (t, ^3J = 6.3 Hz, 2H, -C**H**$_2$-O-), 3.46 (t, ^3J = 7.1 Hz, 2H, Pyr-NH-C**H**$_2$-), 3.27 (dd, ^3J = 14.0 Hz, ^3J = 5.0 Hz, 1H, Tyr$^{\beta}$-**H**/H′), 3.05 (dd, ^3J = 14.0 Hz, ^3J = 9.4 Hz, 1H, Tyr$^{\beta}$-H/**H**′), 1.86 – 1.79 (m, 2H, -C**H**$_2$-CH$_2$-O-), 1.73 (tt, ^3J = 7.3 Hz, ^3J = 7.1 Hz, 2H, Pyr-NH-CH$_2$-C**H**$_2$-), 1.63 – 1.54 (m, 2H, -C**H**$_2$-CH$_2$-CH$_2$-O-).

^{13}C{^{1}H}-NMR (151 MHz, Methanol-d_4) δ [ppm] = 174.9 (-**C**OOH), 170.2 (Ar-**C**ONH-), 159.4 (Tyr-**C**4), 158.8 (Pyr-**C**2), 158.8 (Ar-**C**3), 136.8 (Ar-**C**1), 131.3 (Tyr-**C**2/2′), 130.6 (Ar-**C**5), 130.4 (Tyr-**C**1), 119.7 (Ar-**C**4), 119.2 (Ar-**C**6), 115.5 (Tyr-**C**3/3′), 115.3 (Ar-**C**2), 110.8 (Pyr-**C**5), 68.7 (-**C**H$_2$-O-), 55.8 (Tyr-**C**$^{\alpha}$), 42.5 (Pyr-NH-**C**H$_2$-), 37.4 (Tyr-**C**$^{\beta}$), 30.0 (-**C**H$_2$-CH$_2$-O-), 29.3 (Pyr-NH-CH$_2$-**C**H$_2$-), 24.5 (-**C**H$_2$-CH$_2$-CH$_2$-O-).

LC-MS (ESI+) m/z: 465.3 [M+H]$^+$; t_R = 6.4 min.

3.6.24 (*S*)-2-(((Benzyloxy)carbonyl)amino)-3-(4-((5-(pyrimidin-2-ylamino)pentyl)oxy)-phenyl)propanoic acid (**7db**)

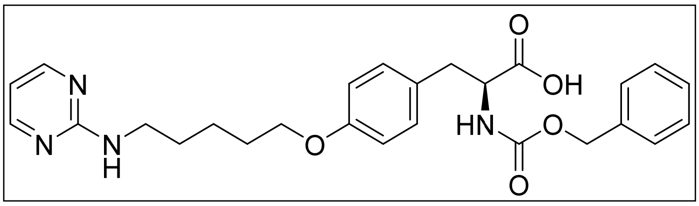

Synthesis according to **GP-5b**: Prepared from **3c**.

The final DHI substituted RGD mimetic **7db** (52.5 mg, 109.7 µmol, 38 %) was isolated as colourless solid.

^{1}H-NMR (600 MHz, Methanol-d_4) δ [ppm] = 8.39 (d, ^3J = 4.5 Hz, 5H, Pyr-4/6-**H**), 7.36 – 7.25 (m, 5H, Cbz-**H**), 7.12 (d, ^3J = 8.5 Hz, 2H, Tyr-2/2′-**H**), 6.80 (d, ^3J = 8.6 Hz, 2H, Tyr-3/3′-**H**), 6.75 (t, ^3J = 5.1 Hz, 1H, Pyr-5-**H**), 5.07 (d, ^2J = 12.7 Hz, 1H, Cbz-C**H**$_2$-), 5.01 (d, ^2J = 12.5 Hz, 1H, Cbz-C**H**$_2$-), 4.38 (dd, ^3J = 9.1 Hz, ^3J = 4.9 Hz, 1H, Tyr$^{\alpha}$-**H**), 3.97 (t, ^3J = 6.3 Hz, 2H, -C**H**$_2$-O-Tyr), 3.45 (t, ^3J = 7.1 Hz, 2H, Pyr-NH-C**H**$_2$-), 3.13 (dd, ^2J = 14.1 Hz, ^3J = 5.1 Hz, 1H, Tyr$^{\beta}$-**H**/H′), 2.86 (dd, ^2J = 14.0 Hz, ^3J = 9.3 Hz,

1H, Tyr$^{\beta}$-H/**H**′), 1.90 - 1.78 (m, 2H, -C**H**$_2$-CH$_2$-O-), 1.77 - 1.68 (m, 2H, Pyr-NH-CH$_2$-C**H**$_2$-), 1.64 - 1.54 (m, 2H, Pyr-NH-CH$_2$-CH$_2$-C**H**$_2$-).

^{13}C{^{1}H}-NMR (151 MHz, Methanol-*d*$_4$) δ [ppm] = 175.2 (-**C**OOH), 160.0 (Pyr-**C**2), 159.4 (Tyr-**C**4), 158.4 (BnO**C**ONH), 138.3 (Ar-**C**1), 131.3 (Tyr-**C**2/2′), 130.4 (Tyr-**C**1), 129.4 (Ar-**C**3/3′), 128.9 (Ar-**C**4), 128.6 (Ar-**C**2/2′), 115.4 (Tyr-**C**3/3′), 110.9 (Pyr-**C**5), 68.7 (-**C**H$_2$-O-Tyr), 67.4 (Cbz-**C**H$_2$-), 57.0 (Tyr-**C**$^{\alpha}$), 42.4 (Pyr-NH-**C**H$_2$-), 37.8 (Tyr-**C**$^{\beta}$), 30.1 (-**C**H$_2$-CH$_2$-O-Tyr), 29.8 (Pyr-NH-CH$_2$-**C**H$_2$-), 24.5 (Pyr-NH-CH$_2$-CH$_2$-**C**H$_2$-).

LC-MS (ESI+) m/z: 479.3 [M+H]$^+$; t$_R$ = 8.1 min.

3.6.25 (*S*)-2-Benzamido-3-(4-(3-((1,4,5,6-tetrahydropyrimidin-2-yl)amino)propoxy)phenyl)-propanoic acid (**5ac**)

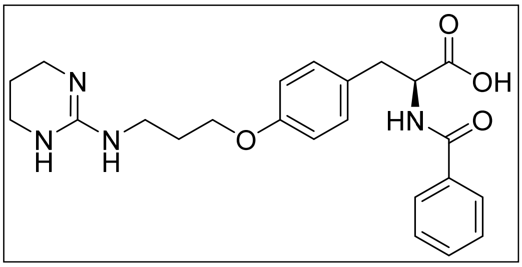

Synthesis according to **GP-5c**: Prepared from **5a**.

The final THP substituted RGD mimetic **5ac** (6.6 mg, 12.2 µmol, 9 %) was isolated as a colourless TFA salt.

^{1}H-NMR (600 MHz, Methanol-*d*$_4$) δ [ppm] = 7.74 (d, ^3J = 8.8 Hz, 2H, Ar-2/2′-**H**), 7.53 (t, ^3J = 7.4 Hz, 1H, Ar-4-**H**), 7.44 (dd, ^3J = 8.8 Hz, ^3J = 7.4 Hz, 2H, Ar-3/3′-**H**), 7.22 (d, ^3J = 8.6 Hz, 2H, Tyr-2/2′-**H**), 6.86 (d, ^3J = 8.6 Hz, 2H, Tyr-3/3′-**H**), 4.82 (dd, ^3J = 9.8 Hz, ^3J = 4.9 Hz, 1H, Tyr$^{\alpha}$-**H**), 4.01 (t, ^3J = 5.7 Hz, 2H, -C**H**$_2$-O-), 3.35 – 3.27 (m, 3H, THP-NH$_2$-C**H**$_2$-, Tyr$^{\beta}$-**H**/H′), 3.27 – 3.22 (m, 4H, THP-4/6-**H**), 3.05 (dd, ^3J = 14.0 Hz, ^3J = 9.7 Hz, 1H, Tyr$^{\beta}$-H/**H**′), 2.00 (tt, ^3J = 6.3 Hz, ^3J = 5.7 Hz, 2H, -CH$_2$-C**H**$_2$-CH$_2$-), 1.83 (p, ^3J = 5.8 Hz, 2H, THP-5-**H**).

^{13}C{^{1}H}-NMR (151 MHz, Methanol-*d*$_4$) δ [ppm] = 175.0 (-**C**OOH), 170.0 (Ar-**C**ONH-), 159.0 (Tyr-**C**4), 154.6 (THP-**C**2), 135.4 (Ar-**C**1), 132.8 (Ar-**C**4), 131.4 (Tyr-**C**2/2′), 131.2 (Tyr-**C**1), 129.5 (Ar-**C**3/3′), 128.3 (Ar-**C**2/2′), 115.4 (Tyr-**C**3/3′), 65.8 (-**C**H$_2$-O-), 55.9 (Tyr-**C**$^{\alpha}$), 39.7 (THP-**C**4/6), 39.0 (THP-NH-**C**H$_2$-), 37.5 (Tyr-**C**$^{\beta}$), 29.8 (-CH$_2$-**C**H$_2$-CH$_2$-), 21.1 (THP-**C**5).

^{19}F{^{1}H}-NMR (470 MHz, Methanol-*d*$_4$) δ [ppm] = -76.93 (TFA).

LC-MS (ESI+) m/z: 425.3 [M+H]$^+$; t$_R$ = 5.1 min.

3.6.26 (*S*)-2-(4-Hydroxybenzamido)-3-(4-(3-((1,4,5,6-tetrahydropyrimidin-2-yl)amino)-propoxy)phenyl)propanoic acid (**5bc**)

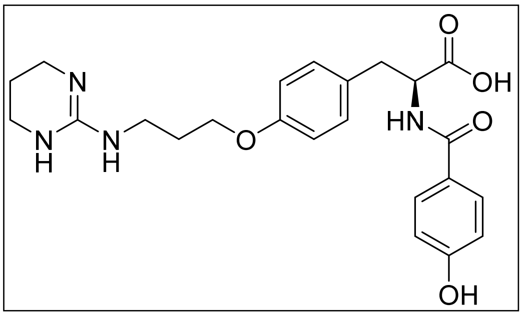

Synthesis according to **GP-5c**: Prepared from **5b**.

The final THP substituted RGD mimetic **5bc** (5.0 mg, 8.9 µmol, 18 %) was isolated as a colourless TFA salt.

^{1}H-NMR (600 MHz, Methanol-d_4) δ [ppm] = 7.65 (d, ^3J = 8.7 Hz, 2H, Ar-2/2′-**H**), 7.21 (d, ^3J = 8.6 Hz, 2H, Tyr-2/2′-**H**), 6.86 (d, ^3J = 8.6 Hz, 2H, Tyr-3/3′-**H**), 6.81 (d, ^3J = 8.7 Hz, 2H, Ar-3/3′-**H**), 4.79 (dd, ^3J = 9.6 Hz, ^3J = 4.9 Hz, 1H, Tyr^α-**H**), 4.01 (t, ^3J = 5.7 Hz, 2H, -C**H**$_2$-O-), 3.36 – 3.31 (m, 2H, THP-NH$_2$-C**H**$_2$-), 3.28 (dd, ^2J = 14.1 Hz, ^3J = 4.9 Hz, 1H, Tyr^β-**H**/H′), 3.24 (t, ^3J = 5.8 Hz, 4H, THP-4/6-**H**), 3.04 (dd, ^3J = 14.0 Hz, ^3J = 9.6 Hz, 1H, Tyr^β-**H**/H′), 2.00 (tt, ^3J = 6.2 Hz, ^3J = 5.7 Hz, 2H, -CH$_2$-**C**H$_2$-CH$_2$-), 1.83 (p, ^3J = 5.8 Hz, 2H, THP-**C**5).

^{13}C{^{1}H}-NMR (151 MHz, Methanol-d_4) δ [ppm] = 175.1 (-**C**OOH), 169.8 (Ar-**C**ONH-), 162.2 (Ar-**C**4), 159.0 (Tyr-**C**4), 154.5 (THP-**C**2), 131.4 (Tyr-**C**2/2′), 131.2 (Tyr-**C**1), 130.4 (Ar-**C**2/2′), 126.0 (Ar-**C**1), 116.0 (Ar-**C**3/3′), 115.4 (Tyr-**C**3/3′), 65.8 (-**C**H$_2$-O-), 55.8 (Tyr-**C**^{α}), 39.7 (THP-**C**4/6), 38.9 (THP-NH-**C**H$_2$-), 37.5 (Tyr-**C**^{β}), 29.8 (-CH$_2$-**C**H$_2$-CH$_2$-), 21.1 (THP-**C**5).

^{19}F{^{1}H}-NMR (470 MHz, Methanol-d_4) δ [ppm] = -76.93 (TFA).

LC-MS (ESI+) m/z: 441.3 [M+H]$^+$; t_R = 4.5 min.

3.6.27 (*S*)-2-(3-Hydroxybenzamido)-3-(4-(3-((1,4,5,6-tetrahydropyrimidin-2-yl)amino)-propoxy)phenyl)propanoic acid (**5cc**)

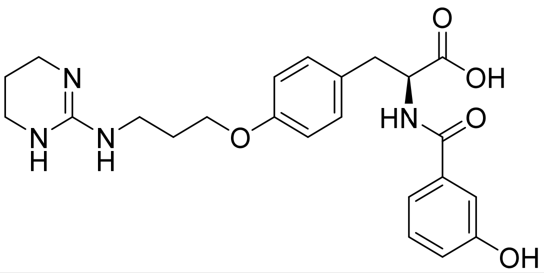

Synthesis according to **GP-5c**: Prepared from **5c**.

The final THP substituted RGD mimetic **5cc** (5.1 mg, 9.1 µmol, 61 %) was isolated as a colourless TFA salt.

^{1}H-NMR (600 MHz, Methanol-d_4) δ [ppm] = 7.24 (dd, ^3J = 8.1 Hz, ^3J = 7.9 Hz, 1H, Ar-5-**H**), 7.19 – 7.17 (m, 1H, Ar-6-**H**), 7.16 (d, ^3J = 8.7 Hz, 2H, Tyr-2/2′-**H**), 7.14 (dd, ^2J = 2.5 Hz, ^2J = 1.0 Hz, 1H, Ar-2-**H**), 6.92 (ddd, ^3J = 8.1 Hz, ^2J = 2.5 Hz, ^2J = 1.0 Hz, 1H, Ar-4-**H**), 6.81 (d, ^3J = 8.5 Hz, 2H, Tyr-3/3′-**H**), 4.65 (dd, ^3J = 7.0 Hz, ^3J = 5.0 Hz, 1H, Tyr^α-**H**), 4.00 (t, ^3J = 5.7 Hz, 2H, -C**H**$_2$-O-), 3.33 – 3.30 (m, 2H, THP-NH-C**H**$_2$-), 3.27 (dd, ^2J = 13.9 Hz, ^3J = 5.0 Hz, 1H, Tyr^β-**H**/H′), 3.24 (t, ^3J = 5.8 Hz, 4H, THP-4/6-**H**), 3.06 (dd, ^3J = 13.8 Hz, ^3J = 7.1 Hz, 1H, Tyr^β-H/**H**′), 1.99 (tt, ^3J = 6.2 Hz, ^3J = 5.7 Hz, 2H, -CH$_2$-C**H**$_2$-CH$_2$-), 1.83 (p, ^3J = 5.9 Hz, 2H, THP-5-**H**).

^{13}C{^{1}H}-NMR (151 MHz, Methanol-d_4) δ [ppm] = 177.3 (-**C**OOH), 169.1 (Ar-**C**ONH-), 158.9 (Ar-**C**3), 158.8 (Tyr-**C**4), 137.3 (Ar-**C**1), 131.7 (Tyr-**C**2/2′), 131.7 (Tyr-**C**1), 130.6 (Ar-**C**5), 119.5 (Ar-**C**4), 119.0 (Ar-**C**6), 115.2 (Tyr-**C**3/3′), 115.0 (Ar-**C**2), 65.8 (-**C**H$_2$-O-), 57.5 (Tyr-**C**$^\alpha$), 39.7 (THP-**C**4/6), 39.0 (THP-NH-**C**H$_2$-), 38.1 (Tyr-**C**$^\beta$), 29.8 (-CH$_2$-**C**H$_2$-CH$_2$-), 21.1 (THP-**C**5).

^{19}F{^{1}H}-NMR (470 MHz, Methanol-d_4) δ [ppm] = -76.96 (TFA).

LC-MS (ESI+) m/z: 441.2 [M+H]$^+$; t_R = 4.8 min.

3.6.28 (*S*)-2-(((Benzyloxy)carbonyl)amino)-3-(4-(3-((1,4,5,6-tetrahydropyrimidin-2-yl)-amino)propoxy)phenyl)propanoic acid (**5dc**)

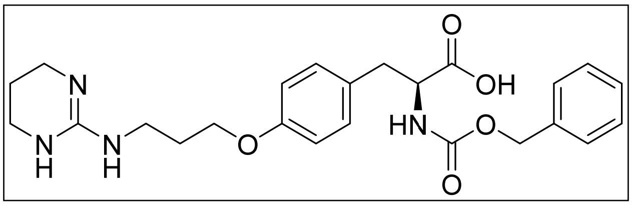

Synthesis according to **GP-5d**: Prepared from **3c**. The final THP substituted RGD mimetic **5dc** (34.9 mg, 61.4 µmol, 27 %) was isolated as a colourless TFA salt.

^{1}H-NMR (600 MHz, Methanol-d_4) δ [ppm] = 7.34 – 7.27 (m, 5H, Cbz-**H**), 7.15 (d, ^3J = 8.2 Hz, 2H, Tyr-2/2′-**H**), 6.85 (d, ^3J = 8.5 Hz, 2H, Tyr-3/3′-**H**), 5.05 (d, ^2J = 12.6 Hz, 1H, Cbz-C**H**$_2$-), 5.02 (d, ^2J = 12.5 Hz, 1H, Cbz-C**H**$_2$-), 4.36 (dd, ^3J = 9.2 Hz, ^3J = 4.9 Hz, 1H, Tyr$^\alpha$-**H**), 4.03 (t, ^3J = 5.7 Hz, 2H, -C**H**$_2$-O-Tyr), 3.34 (t, ^3J = 6.7 Hz, 2H, THP-NH-C**H**$_2$-), 3.28 (t, ^3J = 5.8 Hz, 4H, THP-4/6-**H**), 3.14 (dd, ^2J = 14.0 Hz, ^3J = 4.9 Hz, 1H, Tyr$^\beta$-**H**/H′), 2.87 (dd, ^2J = 14.0 Hz, ^3J = 9.3 Hz, 1H, Tyr$^\beta$-H/**H**′), 2.02 (t, ^3J = 6.7 Hz, ^3J = 5.7 Hz, 2H, -CH$_2$-C**H**$_2$-CH$_2$-), 1.86 (p, ^3J = 5.9 Hz, 2H, THP-5-**H**).

^{13}C{^{1}H}-NMR (151 MHz, Methanol-d_4) δ [ppm] = 175.1 (-**C**OOH), 159.0 (Tyr-**C**4), 158.4 (BnO**C**ONH-), 154.6 (THP-**C**2), 138.2 (Ar-**C**1), 131.4 (Tyr-**C**2/2′), 131.0 (Tyr-**C**1), 129.4 (Ar-**C**3/3′), 128.9 (Ar-**C**4), 128.7 (Ar-**C**2/2′), 115.4 (Tyr-**C**3/3′), 67.5 (Cbz-**C**H$_2$-), 65.8 (-**C**H$_2$-O-Tyr), 57.0 (Tyr-**C**$^\alpha$), 39.7 (THP-**C**4/6), 39.0 (THP-NH-**C**H$_2$-), 37.8 (Tyr-**C**$^\beta$), 29.8 (-CH$_2$-**C**H$_2$-CH$_2$-), 21.1 (THP-**C**5).

^{19}F{^{1}H}-NMR (470 MHz, Methanol-d_4) δ [ppm] = -76.99 (TFA).

LC-MS (ESI+) m/z: 909.5 [2M+H]$^+$, 455.4 [M+H]$^+$; t_R = 6.0 min.

3.6.29 (*S*)-2-Benzamido-3-(4-(4-((1,4,5,6-tetrahydropyrimidin-2-yl)amino)butoxy)phenyl)-propanoic acid (**6ac**)

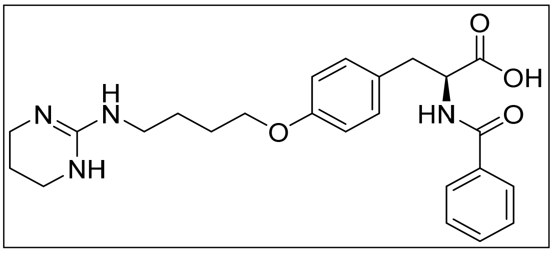

Synthesis according to **GP-5c**: Prepared from **6a**.

The final THP substituted RGD mimetic **6ac** (1.2 mg, 2.1 µmol, 14 %) was isolated as a colourless TFA salt.

^{1}H-NMR (600 MHz, Methanol-*d*$_4$) δ [ppm] = 7.75 (d, ^3J = 8.5 Hz, 2H, Ar-2/2´-**H**), 7.54 (t, ^3J = 7.4 Hz, 1H, Ar-4-**H**), 7.45 (dd, ^3J = 8.5 Hz, ^3J = 7.4 Hz, 2H, Ar-3/3´-**H**), 7.21 (d, ^3J = 8.6 Hz, 2H, Tyr-2/2´-**H**), 6.85 (d, ^3J = 8.6 Hz, 2H, Tyr-3/3´-**H**), 4.83 (dd, ^3J = 9.6 Hz, ^3J = 4.9 Hz, 1H, Tyr$^\alpha$-**H**), 3.99 (t, ^3J = 6.0 Hz, 2H, -C**H**$_2$-O-), 3.38 – 3.32 (m, 4H, THP-4/6-**H**), 3.32 – 3.28 (m, 1H, Tyr$^\beta$-**H**/H´), 3.18 (t, ^3J = 7.0 Hz, 2H, THP-NH-C**H**$_2$-), 3.05 (dd, ^2J = 14.1 Hz, ^3J = 9.6 Hz, 1H, Tyr$^\beta$-H/**H**´), 1.95 (p, ^3J = 5.8 Hz, 2H, THP-5-**H**), 1.85 – 1.79 (m, 2H, -CH$_2$-CH$_2$-C**H**$_2$-CH$_2$-), 1.78 – 1.72 (m, 2H, -CH$_2$-C**H**$_2$-CH$_2$-CH$_2$-).

^{13}C{^{1}H}-NMR (151 MHz, Methanol-*d*$_4$) δ [ppm] = 174.9 (-**C**OOH), 170.2 (Ar-**C**ONH-), 159.3 (Tyr-**C**4), 154.5 (THP-**C**2), 135.4 (Ar-**C**1), 132.8 (Ar-**C**4), 131.3 (Tyr-**C**2/2´), 130.9 (Tyr-**C**1), 129.5 (Ar-**C**3/3´), 128.4 (Ar-**C**2/2´), 115.5 (Tyr-**C**3/3´), 68.3 (-**C**H$_2$-O-), 55.9 (Tyr-**C**$^\alpha$), 41.7 (THP-NH-**C**H$_2$-), 39.7 (THP-**C**4/6), 37.5 (Tyr-**C**$^\beta$), 27.5 (-CH$_2$-**C**H$_2$-CH$_2$-CH$_2$-), 26.8 (-CH$_2$-CH$_2$-**C**H$_2$-CH$_2$-), 21.2 (THP-**C**5).

^{19}F{^{1}H}-NMR (470 MHz, Methanol-*d*$_4$) δ [ppm] = -76.99 (TFA).

LC-MS (ESI+) m/z: 439.3 [M+H]$^+$; t_R = 5.4 min.

3.6.30 (S)-2-(4-hydroxybenzamido)-3-(4-(4-((1,4,5,6-tetrahydropyrimidin-2-yl)amino)-butoxy)phenyl)propanoic acid (**6bc**)

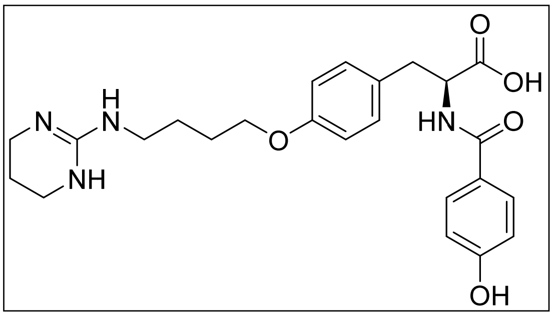

Synthesis according to **GP-5c**: Prepared from **6b**.

The final THP substituted RGD mimetic **6bc** (9.0 mg, 15.7 µmol, 79 %) was isolated as a colourless TFA salt.

^{1}H-NMR (600 MHz, Methanol-*d*$_4$) δ [ppm] = 7.61 (d, ^3J = 8.7 Hz, 2H, Ar-2/2´-**H**), 7.12 (d, ^3J = 8.6 Hz, 2H, Tyr-2/2´-**H**), 6.78 (d, ^3J = 8.7 Hz, 2H, Ar-3/3´-**H**), 6.76 (d, ^3J = 8.6 Hz, 2H, Tyr-3/3´-**H**), 4.64 (dd, ^3J = 6.9 Hz, ^3J = 5.1 Hz, 1H, Tyr$^\alpha$-**H**), 3.94 (t, ^3J = 6.0 Hz, 2H, -C**H**$_2$-O-), 3.33 – 3.31 (m, 4H, THP-4/6-**H**), 3.22 (dd, ^2J = 13.8 Hz, ^3J = 5.1 Hz, 1H, Tyr$^\beta$-**H**/H´), 3.15 (t, ^3J = 6.9 Hz, 2H, THP-NH-C**H**$_2$-), 3.04 (dd, ^2J = 13.8 Hz, ^3J = 7.0 Hz, 1H, Tyr$^\beta$-H/**H**´), 1.91 (p, ^3J = 5.9 Hz, 2H, THP-5-**H**), 1.81 – 1.75 (m, 2H, -CH$_2$-CH$_2$-C**H**$_2$-CH$_2$-), 1.74 – 1.67 (m, 2H, -CH$_2$-C**H**$_2$-CH$_2$-CH$_2$-).

^{13}C{^{1}H}-NMR (151 MHz, Methanol-*d*$_4$) δ [ppm] = 177.6 (-**C**OOH), 169.0 (Ar-**C**ONH-), 162.0 (Ar-**C**4), 159.0 (Tyr-**C**4), 154.5 (THP-**C**2), 131.6 (Tyr-**C**2/2′), 131.5 (Tyr-**C**1), 130.1 (Ar-**C**2/2′), 126.6 (Ar-**C**1), 116.1 (Ar-**C**3/3′), 115.3 (Tyr-**C**3/3′), 68.3 (-**C**H$_2$-O-), 57.5 (Tyr-**C**$^{\alpha}$), 41.6 (THP-NH-**C**H$_2$-), 39.7 (THP-**C**4/6), 38.3 (Tyr-**C**$^{\beta}$), 27.4 (-CH$_2$-**C**H$_2$-CH$_2$-CH$_2$-), 26.8 (-CH$_2$-CH$_2$-**C**H$_2$-CH$_2$-), 21.2 (THP-**C**5).

^{19}F{^{1}H}-NMR (470 MHz, Methanol-*d*$_4$) δ [ppm] = -76.96 (TFA).

LC-MS (ESI+) m/z: 455.3 [M+H]$^+$; t_R = 4.9 min.

3.6.31 (*S*)-2-(3-Hydroxybenzamido)-3-(4-(4-((1,4,5,6-tetrahydropyrimidin-2-yl)amino)-butoxy)phenyl)propanoic acid (**6cc**)

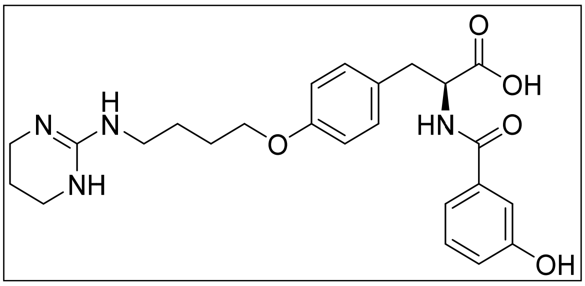

Synthesis according to **GP-5c**: Prepared from **6c**.

The final THP substituted RGD mimetic **6cc** (4.1 mg, 7.1 µmol, 47 %) was isolated as a colourless TFA salt.

^{1}H-NMR (600 MHz, Methanol-*d*$_4$) δ [ppm] = 7.23 (dd, ^3J = 7.9 Hz, ^3J = 7.7 Hz, 1H, Ar-5-**H**), 7.16 (ddd, ^3J = 7.7 Hz, ^4J = 2.6 Hz, ^4J = 1.0 Hz, 1H, Ar-6-**H**), 7.15 – 7.11 (m, 3H, Tyr-2/2′-**H**, Ar-2-**H**), 6.91 (ddd, ^3J = 8.1 Hz, ^4J = 2.6 Hz, ^4J = 1.0 Hz, 1H, Ar-4-**H**), 6.77 (d, *J* = 8.6 Hz, 2H, Tyr-3/3′-**H**), 4.63 (dd, ^3J = 6.8 Hz, ^3J = 5.0 Hz, 1H, Tyr$^{\alpha}$-**H**), 3.96 (t, ^3J = 6.0 Hz, 2H, -C**H**$_2$-O-), 3.34 – 3.31 (m, 4H, Pyr-4/6-**H**), 3.24 (dd, ^2J = 13.8 H, ^3J = 5.0 Hz, 1H, Tyr$^{\beta}$-**H**/H′), 3.15 (t, ^3J = 6.9 Hz, 2H, THP-NH-C**H**$_2$-), 3.05 (dd, ^2J = 13.7 Hz, ^3J = 6.8 Hz, 1H, Tyr$^{\beta}$-H/**H′**), 1.92 (t, ^3J = 5.8 Hz, 2H, THP-5-**H**), 1.83 – 1.75 (m, 2H, -CH$_2$-CH$_2$-C**H**$_2$-CH$_2$-), 1.75 – 1.66 (m, 2H, -CH$_2$-C**H**$_2$-CH$_2$-CH$_2$-).

^{13}C{^{1}H}-NMR (151 MHz, Methanol-*d*$_4$) δ [ppm] = 177.6 (-**C**OOH), 169.2 (Ar-**C**ONH-), 159.0 (Tyr-**C**4), 158.9 (Ar-**C**3), 154.5 (THP-**C**2), 137.3 (Ar-**C**1), 131.6 (Tyr-**C**2/2′), 131.4 (Tyr-**C**1), 130.6 (Ar-**C**5), 119.6 (Ar-**C**4), 119.0 (Ar-**C**6), 115.3 (Tyr-**C**3/3′), 115.0 (Ar-**C**2), 68.3 (-**C**H$_2$-O-), 57.5 (Tyr-**C**$^{\alpha}$), 41.6 (THP-NH-**C**H$_2$-), 39.7 (Pyr-**C**4/6), 38.2 (Tyr-**C**$^{\beta}$), 27.4 (-CH$_2$-**C**H$_2$-CH$_2$-CH$_2$-), 26.8 (-CH$_2$-CH$_2$-**C**H$_2$-CH$_2$-), 21.2 (THP-**C**5).

^{19}F{^{1}H}-NMR (470 MHz, Methanol-*d*$_4$) δ [ppm] = -76.96 (TFA).

LC-MS (ESI+) m/z: 455.2 [M+H]$^+$; t_R = 5.0 min.

3.6.32 (*S*)-2-(((Benzyloxy)carbonyl)amino)-3-(4-(4-((1,4,5,6-tetrahydropyrimidin-2-yl)-amino)butoxy)phenyl)propanoic acid (**6dc**)

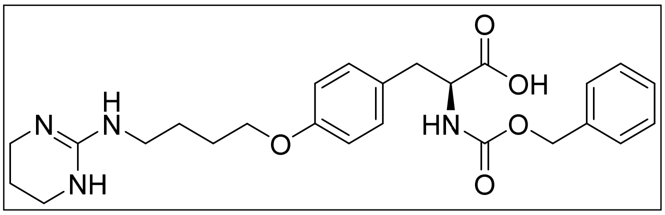

Synthesis according to **GP-5d**: Prepared from **3b**.

The final THP substituted RGD mimetic **6dc** (44.8 mg, 76.8 µmol, 28 %) was isolated as a colourless TFA salt.

^{1}H-NMR (600 MHz, Methanol-d_4) δ [ppm] = 7.35 – 7.25 (m, 5H, Cbz-**H**), 7.13 (d, ^{3}J = 8.1 Hz, 2H, Tyr-2/2′-**H**), 6.81 (d, ^{3}J = 8.1 Hz, 2H, Tyr-3/3′-**H**), 5.05 (d, ^{2}J = 12.6 Hz, 1H, Cbz-C**H**$_2$-), 5.01 (d, ^{2}J = 12.5 Hz, 1H, Cbz-C**H**$_2$-), 4.37 (dd, ^{3}J = 9.2 Hz, ^{3}J = 4.9 Hz, 1H, Tyr$^{\alpha}$-**H**), 3.98 (t, ^{3}J = 5.9 Hz, 2H, -C**H**$_2$-O-Tyr), 3.33 (t, ^{3}J = 5.8 Hz, 4H, THP-4/6-**H**), 3.18 (t, ^{3}J = 6.9 Hz, 2H, THP-NH-C**H**$_2$-), 3.13 (dd, ^{2}J = 14.0 Hz, ^{3}J = 4.8 Hz, 1H, Tyr$^{\beta}$-**H**/H′), 2.86 (dd, ^{3}J = 14.0 Hz, ^{3}J = 9.3 Hz, 1H, Tyr$^{\beta}$-H/**H′**), 1.92 (p, ^{3}J = 6.0 Hz, 2H, THP-5-**H**), 1.86 – 1.70 (m, 4H, -CH$_2$-C**H**$_2$-C**H**$_2$-CH$_2$-).

^{13}C{^{1}H}-NMR (151 MHz, Methanol-d_4) δ [ppm] = 175.1 (-**C**OOH), 159.2 (Tyr-**C**4), 158.3 (BnO**C**ONH-), 154.5 (THP-**C**2), 138.2 (Ar-**C**1), 131.3 (Tyr-**C**2/2′), 130.6 (Tyr-**C**1), 129.4 (Ar-**C**3/3′), 128.9 (Ar-**C**4), 128.6 (Ar-**C**2/2′), 115.5 (Tyr-**C**3/3′), 68.4 (-**C**H$_2$-O-Tyr), 67.4 (Cbz-**C**H$_2$-), 57.0 (Tyr-**C**$^{\alpha}$), 41.7 (THP-NH-**C**H$_2$-), 39.7 (THP-**C**4/6), 37.8 (Tyr-**C**$^{\beta}$), 27.5 (-**C**H$_2$-CH$_2$-O-Tyr), 26.8 (THP-NH-CH$_2$-**C**H$_2$-), 21.2 (THP-**C**5).

^{19}F{^{1}H}-NMR (470 MHz, Methanol-d_4) δ [ppm] = -76.95 (TFA).

LC-MS (ESI+) m/z: 937.5 [2M+H]$^+$, 469.3 [M+H]$^+$; t_R = 6.1 min.

3.6.33 (*S*)-2-Benzamido-3-(4-((5-((1,4,5,6-tetrahydropyrimidin-2-yl)amino)pentyl)oxy)-phenyl)propanoic acid (**7ac**)

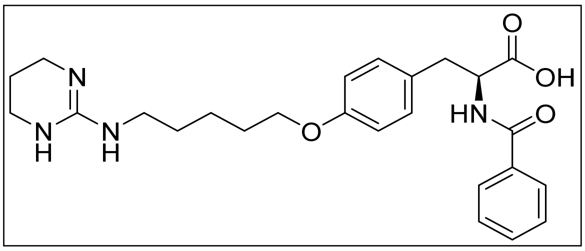

Synthesis according to **GP-5c**: Prepared from **7a**.

The final THP substituted RGD mimetic **7ac** (19.1 mg, 33.8 µmol, 15 %) was isolated as a colourless TFA salt.

^{1}H-NMR (600 MHz, Methanol-d_4) δ [ppm] = 7.75 (d, ^{3}J = 8.5 Hz, 2H, Ar-2/2′-**H**), 7.54 (t, ^{3}J = 7.4 Hz, 1H, Ar-4-**H**), 7.45 (dd, ^{3}J = 8.5 Hz, ^{3}J = 7.4 Hz, 2H, Ar-3/3′-**H**), 7.20 (d, ^{3}J = 8.6 Hz, 2H, Tyr-2/2′), 6.84 (d, ^{3}J = 8.6 Hz, 2H, Tyr-3/3′-**H**), 4.82 (dd, ^{3}J = 9.6 Hz, ^{3}J = 4.9 Hz, 1H, Tyr$^{\alpha}$-**H**), 3.97 (t, ^{3}J = 6.2 Hz, 2H, -C**H**$_2$-O-), 3.36 – 3.32 (m, 4H, THP-4/6-**H**), 3.30 (dd, ^{3}J = 14.1 Hz, ^{3}J = 5.0 Hz, 1H, Tyr$^{\beta}$-**H**/H′), 3.13 (t, ^{3}J = 7.0 Hz, 2H, THP-NH-C**H**$_2$-), 3.05 (dd, ^{3}J = 14.1 Hz, ^{3}J = 9.6 Hz, 1H, Tyr$^{\beta}$-H/**H′**), 1.94 (p,

^3J = 5.8 Hz, 2H, THP-5-**H**), 1.84 - 1.76 (m, 2H, -**C**H_2-CH_2-O-), 1.67 - 1.59 (m, 2H, THP-NH-CH_2-**C**H_2-), 1.58 – 1.51 (m, 2H, -C**H**$_2$-CH_2-CH_2-O-).

^{13}C{^{1}H}-NMR (151 MHz, Methanol-d_4) δ [ppm] = 174.9 (-**C**OOH), 170.2 (Ar-**C**ONH-), 159.4 (Tyr-**C**4), 158.7 (THP-**C**2), 135.4 (Ar-**C**1), 132.8 (Ar-**C**4), 131.3 (Tyr-**C**2/2′), 130.8 (Tyr-**C**1), 129.5 (Ar-**C**3/3′), 128.4 (Ar-**C**2/2′), 115.5 (Tyr-**C**3/3′), 68.6 (-CH_2-O-), 55.7 (Tyr-**C**$^\alpha$), 41.8 (THP-NH-**C**H_2-), 39.7 (THP-**C**4/6), 37.5 (Tyr-**C**$^\beta$), 29.9 (-**C**H_2-CH_2-O-), 29.7 (THP-NH-CH_2-**C**H_2-), 24.4 (-**C**H_2-CH_2-CH_2-O-), 21.2 (THP-**C**5).

^{19}F{^{1}H}-NMR (470 MHz, Methanol-d_4) δ [ppm] = -76.96 (TFA).

LC-MS (ESI+) m/z: 905.5 [2M+H]$^+$, 453.3 [M+H]$^+$; t_R = 5.9 min.

3.6.34 (*S*)-2-(4-Hydroxybenzamido)-3-(4-((5-((1,4,5,6-tetrahydropyrimidin-2-yl)amino)-pentyl)oxy)phenyl)propanoic acid (**7bc**)

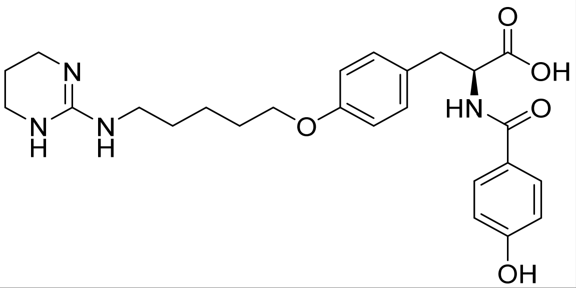

Synthesis according to **GP-5c**: Prepared from **7b**.

The final THP substituted RGD mimetic **7bc** (1.4 mg, 2.4 µmol, 10 %) was isolated as a colourless TFA salt.

^{1}H-NMR (600 MHz, Methanol-d_4) δ [ppm] = 7.64 (d, ^3J = 8.7 Hz, 2H, Ar-2/2′-**H**), 7.18 (d, ^3J = 8.6 Hz, 2H, Tyr-2/2′-**H**), 6.85 – 6.79 (m, 4H, Tyr-3/3′-**H**, Ar-3/3′-**H**), 4.78 (dd, ^3J = 9.4 Hz, ^3J = 4.9 Hz, 1H, Tyr$^\alpha$-**H**), 3.96 (t, ^3J = 6.2 Hz, 2H, -C**H**$_2$-O-), 3.36 – 3.29 (m, 4H, THP-4/6-**H**), 3.26 (dd, ^2J = 14.1 Hz, ^3J = 5.1 Hz, 1H, Tyr$^\beta$-**H**/H′), 3.12 (t, ^3J = 7.0 Hz, 2H, THP-NH-C**H**$_2$-), 3.04 (dd, ^3J = 14.0 Hz, ^3J = 9.4 Hz, 1H, Tyr$^\beta$-H/**H′**), 1.93 (p, ^3J = 5.7 Hz, 2H, THP-5-**H**), 1.83 – 1.75 (m, 2H, -C**H**$_2$-CH_2-O-), 1.66 – 1.60 (m, 2H, THP-NH-CH_2-C**H**$_2$-), 1.58 – 1.49 (m, 2H, -C**H**$_2$-CH_2-CH_2-O-).

^{13}C{^{1}H}-NMR (151 MHz, Methanol-d_4) δ [ppm] = 175.1 (-**C**OOH), 169. (Ar-**C**ONH-), 162.2 (Ar-**C**4), 159.3 (Tyr-**C**4), 154.5 (THP-**C**2), 131.3 (Tyr-**C**2/2′), 130.8 (Tyr-**C**1), 130.4 (Ar-**C**2/2′), 126.1 (Ar-**C**1), 116.0 (Ar-**C**3/3′), 115.4 (Tyr-**C**3/3′), 68.6 (-**C**H_2-O-), 55.8 (Tyr-**C**$^\alpha$), 41.8 (THP-NH-**C**H_2-), 39.7 (THP-**C**4/6), 37.5 (Tyr-**C**$^\beta$), 29.9 (-**C**H_2-CH_2-O-), 29.7 (THP-NH-CH_2-**C**H_2-), 24.4 (-**C**H_2-CH_2-CH_2-O-), 21.2 (THP-**C**5).

^{19}F{^{1}H}-NMR (470 MHz, Methanol-d_4) δ [ppm] = -76.96 (TFA).

LC-MS (ESI+) m/z: 937.6 [2M+H]$^+$, 469.4 [M+H]$^+$; t_R = 5.9 min.

3.6.35 (*S*)-2-(3-Hydroxybenzamido)-3-(4-((5-((1,4,5,6-tetrahydropyrimidin-2-yl)amino)-pentyl)oxy)phenyl)propanoic acid (**7cc**)

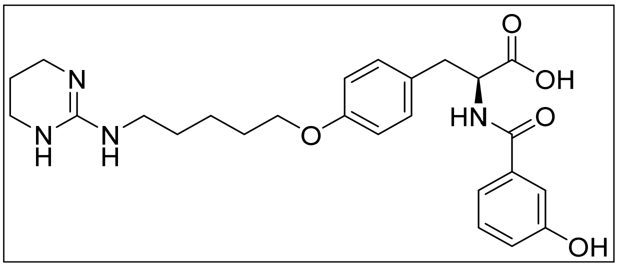

Synthesis according to **GP-5c**: Prepared from **7c**.

The final THP substituted RGD mimetic **7cc** (6.2 mg, 10.6 µmol, 43 %) was isolated as a colourless TFA salt.

^{1}H-NMR (600 MHz, Methanol-*d*$_4$) δ [ppm] = 7.25 (t, ^3J = 7.9 Hz, 1H, Ar-5-**H**), 7.20 – 7.17 (m, 3H, Tyr-2/2′-**H**, Ar-6-**H**), 7.15 (dd, ^4J = 2.6 Hz, ^4J = 1.0 Hz, 1H, Ar-2-**H**), 6.94 (ddd, ^3J = 8.1 Hz, ^3J = 2.6 Hz, ^3J = 1.0 Hz, 1H, Ar-4-**H**), 6.83 (d, ^3J = 8.7 Hz, 2H, Tyr-3/3′-**H**), 4.79 (dd, ^3J = 9.4 Hz, ^3J = 4.9 Hz, 1H, Tyr^α-**H**), 3.96 (t, ^3J = 6.2 Hz, 2H, -C**H**$_2$-O-), 3.36 – 3.30 (m, 4H, THP-4/6-**H**), 3.27 (dd, ^3J = 14.0 Hz, ^3J = 4.9 Hz, 1H, Tyr^β-**H**/H′), 3.12 (t, ^3J = 7.0 Hz, 2H, THP-NH-C**H**$_2$-), 3.04 (dd, ^3J = 14.1 Hz, ^3J = 9.4 Hz, 1H, Tyr^β-H/**H**′), 1.93 (p, ^3J = 5.9 Hz, 2H, THP-5-**H**), 1.84 – 1.74 (m, 2H, -C**H**$_2$-CH$_2$-O-), 1.68 – 1.59 (m, 2H, THP-NH-CH$_2$-C**H**$_2$-), 1.58 – 1.49 (m, 2H, -C**H**$_2$-CH$_2$-CH$_2$-O-).

^{13}C{^{1}H}-NMR (151 MHz, Methanol-*d*$_4$) δ [ppm] = 174.9 (-**C**OOH), 170.2 (Ar-**C**ONH-), 159.4 (Tyr-**C**4), 158.8 (Ar-**C**3), 154.5 (THP-**C**2), 136.8 (Ar-**C**1), 131.3 (Tyr-**C**2/2′), 130.7 (Tyr-**C**1), 130.6 (Ar-**C**5), 119.7 (Ar-**C**4), 119.2 (Ar-**C**6), 115.5 (Tyr-**C**3/3′), 115.3 (Ar-**C**2), 68.6 (-**C**H$_2$-O-), 55.8 (Tyr-**C**^{α}), 41.8 (THP-NH-**C**H$_2$-), 39.7 (THP-**C**4/6), 37.4 (Tyr-**C**^{β}), 29.9 (-**C**H$_2$-CH$_2$-O-), 29.7 (THP-NH-CH$_2$-**C**H$_2$-), 24.4 (-**C**H$_2$-CH$_2$-CH$_2$-O-), 21.2 (THP-**C**5).

^{19}F{^{1}H}-NMR (470 MHz, Methanol-*d*$_4$) δ [ppm] = -76.94 (TFA).

LC-MS (ESI+) m/z: 937.5 [2M+H]$^+$, 469.3 [M+H]$^+$; t_R = 5.5 min.

3.6.36 (*S*)-2-(((Benzyloxy)carbonyl)amino)-3-(4-((5-((1,4,5,6-tetrahydropyrimidin-2-yl)-amino)pentyl)oxy)phenyl)propanoic acid (**7dc**)

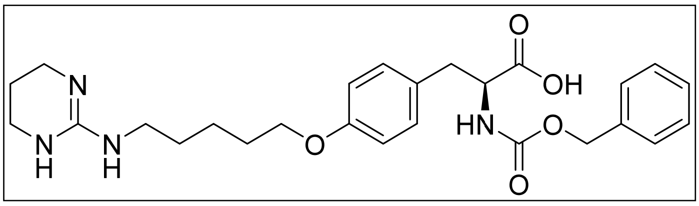

Synthesis according to **GP-5d**: Prepared from **3c**.

The final THP substituted RGD mimetic **7dc** (21.1 mg, 35.3 µmol, 12 %) was isolated as a colourless TFA salt.

^{1}H-NMR (600 MHz, Methanol-*d*$_4$) δ [ppm] = 7.38 – 7.29 (m, 5H, Cbz-**H**), 7.16 (d, ^3J = 8.1 Hz, 2H, Tyr-2/2′-**H**), 6.84 (d, ^3J = 8.1 Hz, 2H, Tyr-3/3′-**H**), 5.09 (d, ^2J = 12.6 Hz, 1H, Cbz-C**H**$_2$-), 5.04 (d, ^2J = 12.4 Hz, 1H, Cbz-C**H**$_2$-), 4.40 (dd, ^3J = 9.2 Hz, ^3J = 4.9 Hz, 1H, Tyr^α-**H**), 4.00 (t, ^3J = 6.2 Hz, 2H, -C**H**$_2$-O-Tyr), 3.36 (d, ^3J = 5.9 Hz, 2H, THP-4/6-**H**), 3.23 – 3.08 (m, 3H, THP-NH-C**H**$_2$-, Tyr^β-

H/**H**′), 2.89 (dd, ^{2}J = 13.9 Hz, ^{3}J = 9.2 Hz, 1H, Tyr$^{\beta}$-H/**H**′), 1.96 (p, ^{3}J = 5.9 Hz, 2H, THP-5-**H**), 1.88 – 1.79 (m, 2H, -C**H**$_2$-CH$_2$-O-), 1.73 - 1.63 (m, 2H, THP-NH-CH$_2$-C**H**$_2$-), 1.62 – 1.54 (m, 2H, -C**H**$_2$-CH$_2$-CH$_2$-O-).

^{13}C{^{1}H}-NMR (151 MHz, Methanol-*d*$_4$) δ [ppm] = 175.1 (-**C**OOH), 159.4 (Tyr-**C**4), 158.4 (BnO**C**ONH-), 154.5 (THP-**C**2), 138.3 (Ar-**C**1), 131.3 (Tyr-**C**2/2′), 130.5 (Tyr-**C**1), 129.4 (Ar-**C**3/3′), 128.9 (Ar-**C**4), 128.6 (Ar-**C**2/2′), 115.5 (Tyr-**C**3/3′), 68.7 (-**C**H$_2$-O-Tyr), 67.5 (Cbz-**C**H$_2$-), 57.0 (Tyr-**C**$^{\alpha}$), 41.9 (THP-NH-**C**H$_2$-), 39.7 (THP-**C**4/6), 37.9 (Tyr-**C**$^{\beta}$), 30.0 (-**C**H$_2$-CH$_2$-O-Tyr), 29.7 (THP-NH-CH$_2$-**C**H$_2$-), 24.4 (-**C**H$_2$-CH$_2$-CH$_2$-O-), 21.2 (THP-**C**5).

^{19}F{^{1}H}-NMR (470 MHz, Methanol-*d*$_4$) δ [ppm] = -77.04 (TFA).

LC-MS (ESI+) m/z: 965.5 [2M+H]$^+$, 483.4 [M+H]$^+$; t$_R$ = 6.6 min.

3.7 Precursors for Conjugable RGD-Mimetics

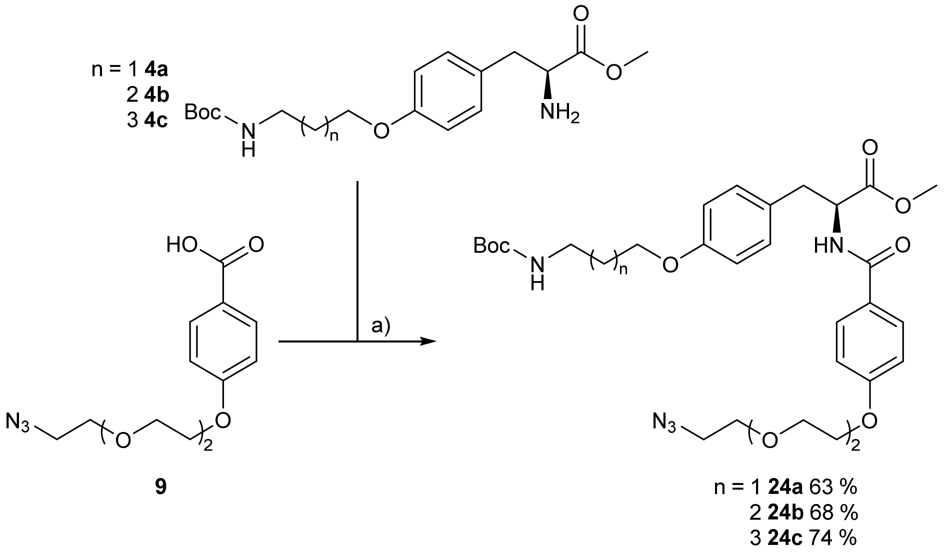

Figure S12: Synthesis of RGD mimetics precursors **24a-c** for conjugation. Reagents and conditions: a) HOBt, EDC, DIPEA, DMF, DCM, o.n., RT.

3.7.1 Methyl (*S*)-2-(4-(2-(2-(2-Azidoethoxy)ethoxy)ethoxy)benzamido)-3-(4-(3-((tert-butoxycarbonyl)amino)propoxy)phenyl)propanoate (**24a**)

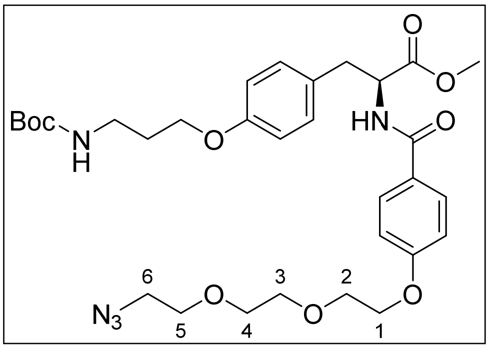

Synthesis according to **GP-5a**: Prepared from **4a** and **9** (1.1 eq.).

The conjugable RGD mimetic precursor **24a** (268.6 mg, 427.0 µmol, 63 %) was isolated as a colourless solid.

^{1}H-NMR (600 MHz, Methanol-d_4) δ [ppm] = 7.77 (d, ^3J = 8.8 Hz, 2H, Ar-2/2′-**H**), 7.19 (d, ^3J = 8.3 Hz, 2H, Tyr-2/2′-**H**), 7.00 (d, ^3J = 8.8 Hz, 2H, Ar-3/3′-**H**), 6.86 (d, ^3J = 8.6 Hz, 2H, Tyr-3/3′-**H**), 4.82 (dd, ^3J = 9.2 Hz, ^3J = 5.6 Hz, 1H, Tyr^α-**H**), 4.22 – 4.15 (m, 2H, PEG-1-**H**), 3.97 (t, ^3J = 6.1 Hz, 2H, -C**H**$_2$-O-), 3.91 – 3.83 (m, 2H, PEG-2-**H**), 3.75 (s, 3H, -C**H**$_3$), 3.74 – 3.72 (m, 2H, PEG-3-**H**), 3.70 – 3.66 (m, 4H, PEG-4-**H**, PEG-5-**H**), 3.38 (t, ^3J = 4.9 Hz, 2H, PEG-6-**H**), 3.28 – 3.21 (m, 3H, BocNH-C**H**$_2$-, Tyr^β-**H**/H′), 3.09 (dd, ^3J = 14.0 Hz, ^3J = 9.2 Hz, 1H, Tyr^β-H/**H′**), 1.93 (p, ^3J = 6.5 Hz, 2H, -CH$_2$-C**H**$_2$-CH$_2$-), 1.45 (s, 9H, Boc-**H**).

^{13}C{^{1}H}-NMR (151 MHz, Methanol-d_4) δ [ppm] = 173.8 (-**C**OOMe), 169.5 (Ar-**C**ONH-), 163.2 (Ar-**C**4), 159.3 (Tyr-**C**4), 158.4 (^{t}BuO**C**ONH-), 131.2 (Tyr-**C**2/2′), 130.4 (Tyr-**C**1), 130.3 (Ar-**C**2/2′), 127.3 (Ar-**C**1), 115.5 (Tyr-**C**3/3′), 115.3 (Ar-**C**3/3′), 79.9 (-**C**(CH$_3$)$_3$), 71.8 (PEG-**C**3), 71.5 (PEG-**C**4), 71.1 (PEG-**C**5), 70.7 (PEG-**C**2), 68.7 (PEG-**C**1), 66.5 (-**C**H$_2$-O-), 56.0 (Tyr-**C**^{α}), 52.7 (-**C**H$_3$), 51.7 (PEG-**C**6), 38.4 (BocNH-**C**H$_2$-), 37.4 (Tyr-**C**^{β}), 30.7 (-CH$_2$-**C**H$_2$-CH$_2$-), 28.8 (-C(**C**H$_3$)$_3$).

LC-MS (ESI+) m/z: 630.4 [M+H]$^+$, 574.4 [M-^{t}Bu+H]$^+$, 530.4 [M-Boc+H]$^+$; t_R = 9.8 min.

3.7.2 Methyl (*S*)-2-(4-(2-(2-(2-Azidoethoxy)ethoxy)ethoxy)benzamido)-3-(4-(4-((tert-butoxycarbonyl)amino)butoxy)phenyl)propanoate (**24b**)

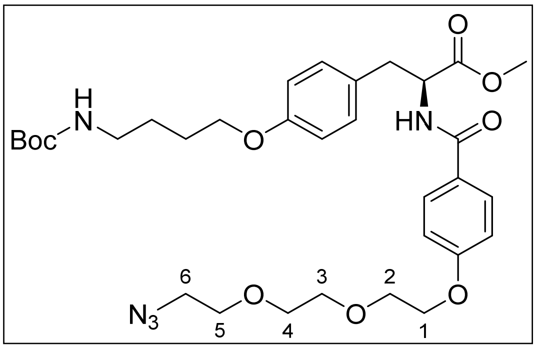

Synthesis according to **GP-5a**: Prepared from **4b** and **9** (1.1 eq.).

The conjugable RGD mimetic precursor **24b** (305.5 mg, 475.0 µmol, 68 %) was isolated as a colourless solid.

^{1}H-NMR (600 MHz, Methanol-d_4) δ [ppm] = 7.76 (d, ^3J = 8.8 Hz, 2H, Ar-2/2′-**H**), 7.17 (d, ^3J = 8.6 Hz, 2H, Tyr-2/2′-**H**), 7.00 (d, ^3J = 8.8 Hz, 2H, Ar-3/3′-**H**), 6.85 (d, ^3J = 8.6 Hz, 2H, Tyr-3/3′-**H**), 4.81 (dd, ^3J = 9.2 Hz, ^3J = 5.6 Hz, 1H, Tyr^α-**H**), 4.23 – 4.13 (m, 2H, PEG-1-**H**), 3.94 (t, ^3J = 6.3 Hz, 2H, -C**H**$_2$-O-), 3.91 – 3.85 (m, 2H, PEG-2-**H**), 3.74 (s, 3H, -C**H**$_3$), 3.73 – 3.71 (m, 2H, PEG-3-**H**), 3.70 – 3.66 (m, 4H, PEG-4-**H**, PEG-5-**H**), 3.37 (t, ^3J = 5.0 Hz, 2H, PEG-6-**H**), 3.23 (dd, ^2J = 13.9 Hz, ^3J = 5.7 Hz, 1H,

Tyr$^{\beta}$-**H**/H′), 3.14 – 3.05 (m, 3H, BocNH-C**H**$_2$-, Tyr$^{\beta}$-H/**H**′), 1.83 – 1.73 (m, 2H, -C**H**$_2$-CH$_2$-O-), 1.69 – 1.57 (m, 2H, BocNH-CH$_2$-C**H**$_2$-), 1.46 (s, 9H, Boc-**H**).

^{13}C{^{1}H}-NMR (151 MHz, Methanol-*d*$_4$) δ [ppm] = 173.8 (-**C**OOMe), 169.5 (Ar-**C**ONH-), 163.2 (Ar-**C**4), 159.4 (Tyr-**C**4), 158.5 (^{t}BuO**C**ONH-), 131.2 (Tyr-**C**2/2′), 130.3 (Tyr-**C**1), 130.3 (Ar-**C**2/2′), 127.3 (Ar-**C**1), 115.5 (Tyr-**C**3/3′), 115.3 (Ar-**C**3/3′), 79.8 (-**C**(CH$_3$)$_3$), 71.8 (PEG-**C**3), 71.5 (PEG-**C**4), 71.1 (PEG-**C**5), 70.7 (PEG-**C**2), 68.7 (PEG-**C**1), 68.6 (-**C**H$_2$-O-), 56.0 (Tyr-**C**$^{\alpha}$), 52.7 (-**C**H$_3$), 51.7 (PEG-**C**6), 41.1 (BocNH-**C**H$_2$-), 37.4 (Tyr-**C**$^{\beta}$), 28.8 (-C(**C**H$_3$)$_3$), 27.7 (BocNH-CH$_2$-**C**H$_2$-), 27.6 (-**C**H$_2$-CH$_2$-O-).

LC-MS (ESI+) m/z: 644.5 [M+H]$^+$, 588.4 [M-^{t}Bu+H]$^+$, 544.4 [M-Boc+H]$^+$; t$_R$ = 10.0 min.

3.7.3 Methyl (*S*)-2-(4-(2-(2-(2-Azidoethoxy)ethoxy)ethoxy)benzamido)-3-(4-((5-((tert-butoxycarbonyl)amino)pentyl)oxy)phenyl)propanoate (**24c**)

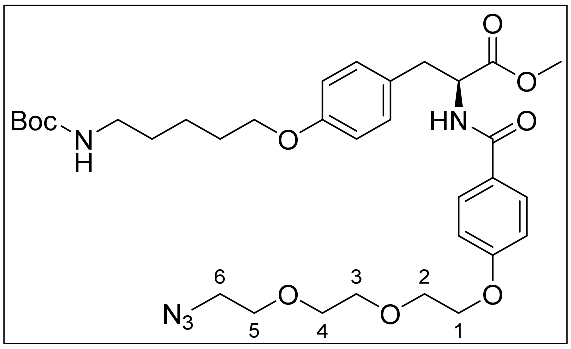

Synthesis according to **GP-5a**: Prepared from **4c** and **9** (1.1 eq.).

The conjugable RGD mimetic precursor **24c** (129.9 mg, 197.5 µmol, 74 %) was isolated as a colourless solid.

^{1}H-NMR (600 MHz, Methanol-*d*$_4$) δ [ppm] = 7.74 (d, ^{3}J = 8.8 Hz, 2H, Ar-2/2′-**H**), 7.16 (d, ^{3}J = 8.6 Hz, 2H, Tyr-2/2′-**H**), 7.00 (d, ^{3}J = 8.8 Hz, 2H, Ar-3/3′-**H**), 6.84 (d, ^{3}J = 8.6 Hz, 2H, Tyr-3/3′-**H**), 4.79 (dd, ^{3}J = 9.2 Hz, ^{3}J = 5.6 Hz, 1H, Tyr$^{\alpha}$-**H**), 4.22 – 4.18 (m, 2H, PEG-1-**H**), 3.94 (t, ^{3}J = 6.4 Hz, 2H, -C**H**$_2$-O-), 3.90 – 3.86 (m, 2H, PEG-2-**H**), 3.73 (s, 3H, -C**H**$_3$), 3.73 – 3.72 (m, 2H, PEG-3-**H**), 3.70 – 3.66 (m, 4H, PEG-4-**H**, PEG-5-**H**), 3.37 (t, ^{3}J = 4.9 Hz, 2H, PEG-6-**H**), 3.21 (dd, ^{2}J = 14.0 Hz, ^{3}J = 5.6 Hz, 1H, Tyr$^{\beta}$-**H**/H′), 3.09 – 3.03 (m, 3H, BocNH-C**H**$_2$-, Tyr$^{\beta}$-H/**H**′), 1.80 – 1.73 (m, 2H, BocNH-CH$_2$-C**H**$_2$-), 1.56 – 1.46 (m, 4H, -C**H**$_2$-C**H**$_2$-CH$_2$-O-), 1.44 (s, 9H, Boc-**H**).

^{13}C{^{1}H}-NMR (151 MHz, Methanol-*d*$_4$) δ [ppm] = 173.9 (-**C**OOMe), 169.7 (Ar-**C**ONH-), 163.3 (Ar-**C**4), 159.5 (Tyr-**C**4), 158.6 (^{t}BuO**C**ONH-), 131.2 (Tyr-**C**2/2′), 130.3 (Tyr-**C**1), 130.3 (Ar-**C**2/2′), 127.3 (Ar-**C**1), 115.5 (Tyr-**C**3/3′), 115.3 (Ar-**C**3/3′), 79.8 (-**C**(CH$_3$)$_3$), 71.9 (PEG-**C**3), 71.6 (PEG-**C**4), 71.2 (PEG-**C**5), 70.8 (PEG-**C**2), 68.8 (PEG-**C**1), 68.8 (-**C**H$_2$-O-), 56.1 (Tyr-**C**$^{\alpha}$), 52.7 (-**C**H$_3$), 51.8 (PEG-**C**6), 41.2 (BocNH-**C**H$_2$-), 37.4 (Tyr-**C**$^{\beta}$), 30.7 (-**C**H$_2$-CH$_2$-O-), 30.1 (BocNH-CH$_2$-**C**H$_2$-), 28.8 (-C(**C**H$_3$)$_3$), 24.4 (-**C**H$_2$-CH$_2$-CH$_2$-O-).

LC-MS (ESI+) m/z: 658.5 [M+H]$^+$, 597.5 [M-^{t}Bu+H]$^+$, 558.4 [M-Boc+H]$^+$; t$_R$ = 10.2 min.

3.8 Conjugable Final RGD-Mimetics

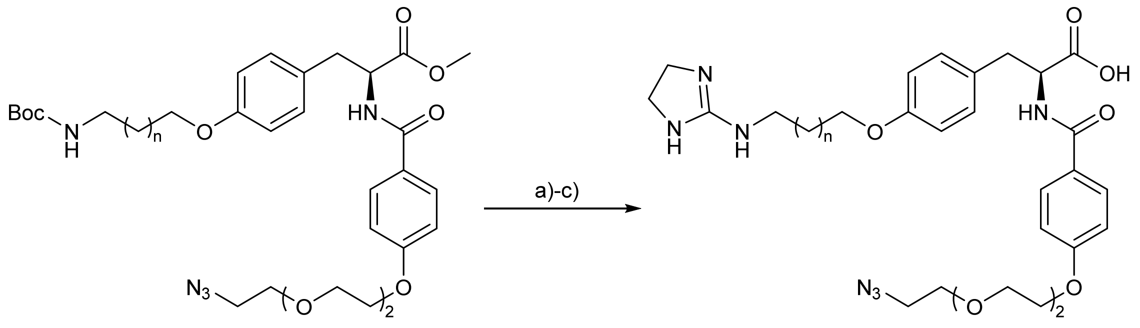

n = 1 **24a**   n = 1 **10a** 57 %
2 **24b**   2 **10b** 55 %
3 **24c**   3 **10c** 49 %

Figure S13: Synthesis of final RGD mimetics **10a-c** for conjugation. Reagents and conditions: a) 4 M HCl in dioxane, DCM, RT; b) 2-methylthio-2-imidazoline, triethylamine, methanol, 80 °C, o.n.; c) LiOH, water, methanol, RT.

3.8.1 (*S*)-2-(4-(2-(2-(2-Azidoethoxy)ethoxy)ethoxy)benzamido)-3-(4-(3-((4,5-dihydro-1H-imidazol-2-yl)amino)propoxy)phenyl)propanoic acid (**10a**)

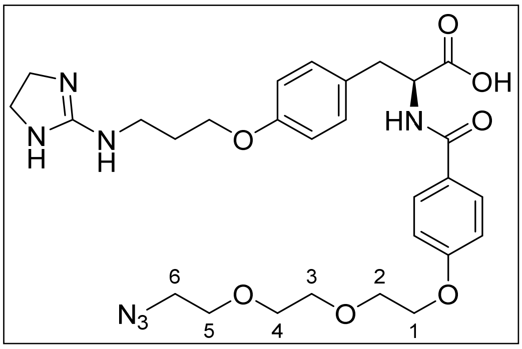

Synthesis according to **GP-5a**: Prepared from **24a.**

The final conjugable RGD mimetic **10a** (20.0 mg, 28.6 µmol, 57 %) was isolated as a colourless TFA salt.

^{1}H-NMR (600 MHz, Methanol-d_4) δ [ppm] = 7.73 (d, ^3J = 8.5 Hz, 2H, Ar-2/2′-**H**), 7.20 (d, ^3J = 8.2 Hz, 2H, Tyr-2/2′-**H**), 6.98 (d, ^3J = 8.8 Hz, 2H, Ar-3/3′-**H**), 6.85 (d, ^3J = 8.2 Hz, 2H, Tyr-3/3′-**H**), 4.80 (dd, ^3J = 9.5 Hz, ^3J = 4.8 Hz, 1H, Tyr$^{\alpha}$-**H**), 4.18 (t, ^3J = 4.6 Hz, 2H, PEG-1-**H**), 4.01 (t, ^3J = 5.8 Hz, 2H, -C**H**$_2$-O-Tyr), 3.87 (t, ^3J = 4.6 Hz, 2H, PEG-2-**H**), 3.74 – 3.71 (m, 2H, PEG-3-**H**), 3.69 – 3.64 (m, 4H, PEG-4-**H**, PEG-5-**H**), 3.58 (s, 4H, DHI-4/5-**H**), 3.41 – 3.34 (m, 4H, DHI-NH-C**H**$_2$-, PEG-6-**H**), 3.28 (dd, ^2J = 14.1 Hz, ^3J = 5.0 Hz, 1H, Tyr$^{\beta}$-**H**/H′), 3.03 (dd, ^2J = 14.0 Hz, ^3J = 9.6 Hz, 1H, Tyr$^{\beta}$-H/**H**′), 2.01 (tt, ^3J = 6.3 Hz, ^3J = 5.8 Hz, 2H, -CH$_2$-C**H**$_2$-CH$_2$-).

^{13}C{^{1}H}-NMR (151 MHz, Methanol-d_4) δ [ppm] = 175.1 (-**C**OOH), 169.5 (Ar-**C**ONH-), 163.2 (Ar-**C**4), 161.4 (DHI-**C**2), 159.0 (Tyr-**C**4), 131.3 (Tyr-**C**2/2′), 131.2 (Tyr-**C**1), 130.3 (Ar-**C**2/2′), 127.5 (Ar-**C**1), 115.4 (Tyr-**C**3/3′), 115.3 (Ar-**C**3/3), 71.8 (PEG-**C**3), 71.6 (PEG-**C**4), 71.2 (PEG-**C**5), 70.7 (PEG-**C**2), 68.8 (PEG-**C**1), 65.8 (-**C**H$_2$-O-), 55.9 (Tyr-**C**$^{\alpha}$), 51.8 (PEG-**C**6), 44.0 (DHI-**C**4/5), 41.0 (DHI-NH-**C**H$_2$-), 37.5 (Tyr-**C**$^{\beta}$), 29.9 (-CH$_2$-**C**H$_2$-CH$_2$-).

^{19}F{^{1}H}-NMR (470 MHz, Methanol-d_4) δ [ppm] = -76.89 (TFA).

LC-MS (ESI+) m/z: 584.5 [M+H]$^+$; t_R = 5.6 min.

3.8.2 (*S*)-2-(4-(2-(2-(2-Azidoethoxy)ethoxy)ethoxy)benzamido)-3-(4-(4-((4,5-dihydro-1H-imidazol-2-yl)amino)butoxy)phenyl)propanoic acid (**10b**)

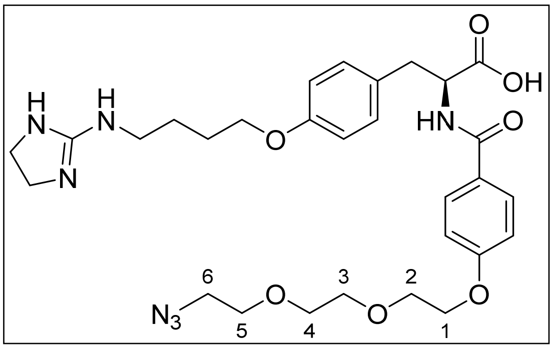

Synthesis according to **GP-5a**: Prepared from **24b**.

The final conjugable RGD mimetic **10b** (19.4 mg, 27.3 µmol, 55 %) was isolated as a colourless TFA salt.

^{1}H-NMR (600 MHz, Methanol-d_4) δ [ppm] = 7.72 (d, ^3J = 8.8 Hz, 2H, Ar-2/2′-**H**), 7.19 (d, ^3J = 8.6 Hz, 2H, Tyr-2/2′-**H**), 6.99 (d, ^3J = 8.8 Hz, 2H, Ar-3/3′-**H**), 6.83 (d, ^3J = 8.6 Hz, 2H, Tyr-3/3′-**H**), 4.79 (dd, ^3J = 9.5 Hz, ^3J = 4.9 Hz, 1H, Tyr$^\alpha$-**H**), 4.22 – 4.17 (m, 2H, PEG-1-**H**), 3.97 (t, ^3J = 5.9 Hz, 2H, -C**H**$_2$-O-Tyr), 3.91 – 3.84 (m, 2H, PEG-2-**H**), 3.74 – 3.70 (m, 2H, PEG-3-**H**), 3.70 – 3.64 (m, 8H, DHI-4/5-**H**, PEG-4-**H**, PEG-5-**H**), 3.36 (t, ^3J = 4.9 Hz, 2H, PEG-6-**H**), 3.29 – 3.22 (m, 3H, DHI-NH-C**H**$_2$-, Tyr$^\beta$-**H**/H′), 3.04 (dd, ^2J = 14.0 Hz, ^3J = 9.5 jHz, 1H, Tyr$^\beta$-H/**H′**), 1.84 – 1.71 (m, 4H, -CH$_2$- -**C**H$_2$-CH$_2$-CH$_2$-).

^{13}C{^{1}H}-NMR (151 MHz, Methanol-d_4) δ [ppm] = 175.0 (-**C**OOH), 169.6 (Ar-**C**ONH-), 163.2 (Ar-**C**4), 161.3 (DHI-**C**2), 159.2 (Tyr-**C**4), 131.3 (Tyr-**C**2/2′), 130.9 (Tyr-**C**1), 130.3 (Ar-**C**2/2′), 127.5 (Ar-**C**1), 115.5 (Tyr-**C**3/3′), 115.3 (Ar-**C**3/3′), 71.9 (PEG-**C**3), 71.6 (PEG-**C**4), 71.2 (PEG-**C**5), 70.8 (PEG-**C**2), 68.8 (PEG-**C**1), 68.3 (-**C**H$_2$-O-), 55.8 (Tyr-**C**$^\alpha$), 51.8 (PEG-**C**6), 44.0 (DHI-**C**4/5), 43.6 (DHI-NH-**C**H$_2$-), 37.5 (Tyr-**C**$^\beta$), 27.4 (-**C**H$_2$-CH$_2$-O-Tyr), 27.0 (DHI-NH-CH$_2$-**C**H$_2$-).

LC-MS (ESI+) m/z: 598.5 [M+H]$^+$; t_R = 5.8 min.

3.8.3 (*S*)-2-(4-(2-(2-(2-Azidoethoxy)ethoxy)ethoxy)benzamido)-3-(4-((5-((4,5-dihydro-1H-imidazol-2-yl)amino)pentyl)oxy)phenyl)propanoic acid (**10c**)

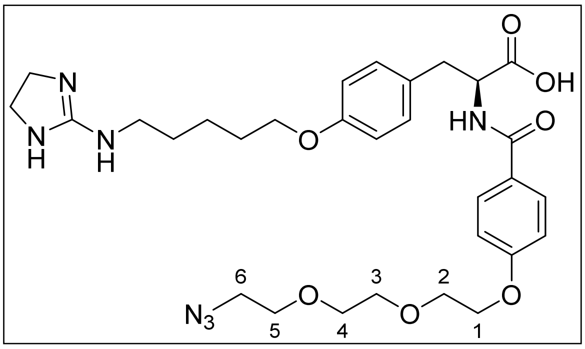

Synthesis according to **GP-5a**: Prepared from **24c**

The final conjugable RGD mimetic **10c** (17.8 mg, 24.5 µmol, 49 %) was isolated as a colourless TFA salt.

LC-MS (ESI+) m/z: 1257.4 [2M+Na]$^+$; 612.5 [M+H]$^+$; t_R = 6.1 min.

3.9 Conjugable Cyclic RGD Peptides

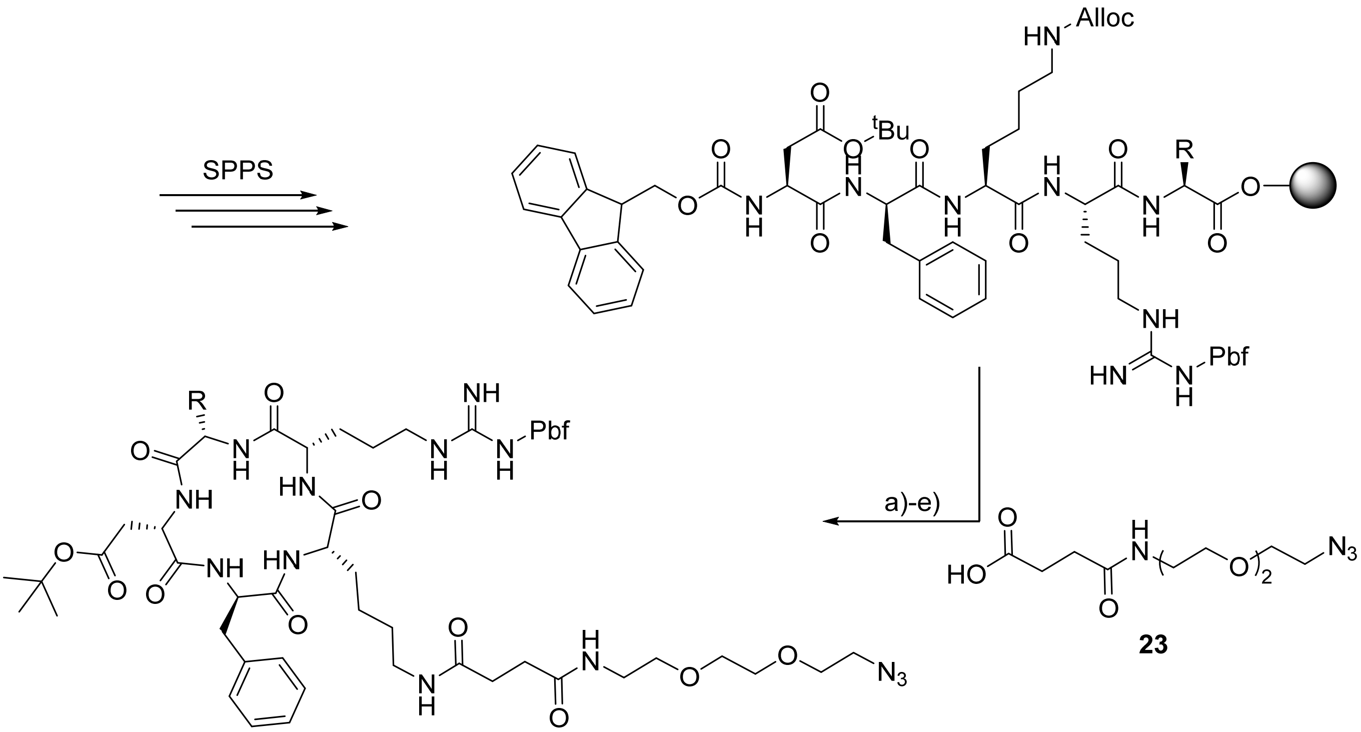

R = H **14** 21 %
CH_3 **15** 57 %

Figure S14: Synthesis of cRGDfK-PEG-N_3 **14** and cRADfK-PEG-N_3 **15**. Reagents and conditions: SPPS **GP-6-8**; a) $Pd(PPh_3)_4$, DMBU, DMF, RT; b) **23**, oxyma, DIC, DMF, RT; c) 20 % piperidine in DMF, RT; d) 1 % TFA, DCM, RT; e) HATU, HOAt, DIPEA, DMF, o.n., RT.

3.9.1 c(Arg(Pbf)-Gly-Asp(tBu)-D-Phe-Lys(PEG-N_3)) (**14**)

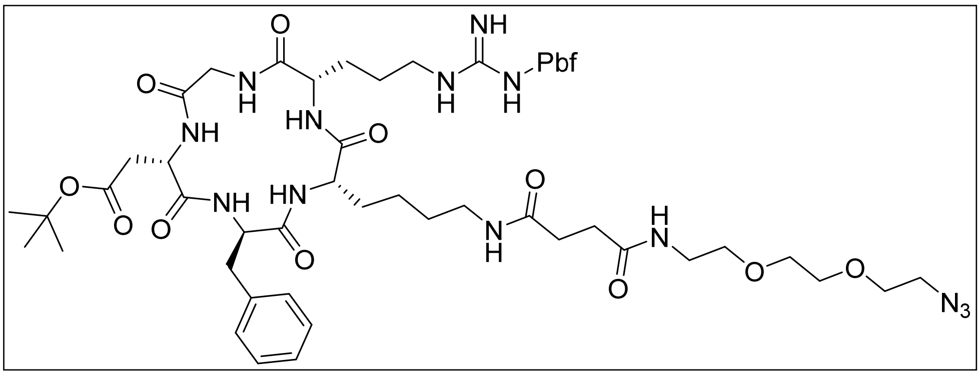

The linear peptide RGDfK was synthesized *via* solid phase peptide synthesis (SPPS) using 2-chlorotrityl chloride resin (1.057 g). Fmoc-Gly-OH was attached (**GP-6**) to the resin as well as the resin loading was determined by following **GP-6** (0.964 mmol/g resin). The further amino acids were coupled in correspondence to **GP-6** by checking the success of every coupling step *via* test cleavage and LC-MS analysis. The resulting linear peptide was treated with $Pd(PPh_3)_4$ and DMBA following **GP-8** to cleave the Alloc protective group. After Fmoc-cleavage (**GP-6**) the resin was washed with DMF (5x10 mL/g resin) and DCM (2x10 mL/g resin) followed by cleaving the peptide (**GP-6**) from the resin. The crude peptide was

cyclised and purified following **GP-7**. The protected conjugable cRGDfK peptide **14** (248.0 mg, 212.5 µmol, 21 %) was isolated as solid.

LC-MS (ESI+) m/z: 1168.6 $[M+H]^+$, 1112.5 $[M-^tBu+H]^+$, 584.8 $[M+2H]^{2+}$, 557.0 $[M+2H]^2$; t_R = 8.8 min.

HRMS (ESI-MS) m/z: $[M+H]^+$ calc. for $[C_{54}H_{82}N_{13}O_{14}S]^+$: 1168.58001, found: 1168.58194; $[M+2H]^{2+}$ calc. for $[C_{54}H_{83}N_{13}O_{14}S]^{2+}$: 584.79335, found: 584.79461.

3.9.2 c(Arg(Pbf)-Ala-Asp(^{t}Bu)-D-Phe-Lys(PEG-N_3)) (**15**)

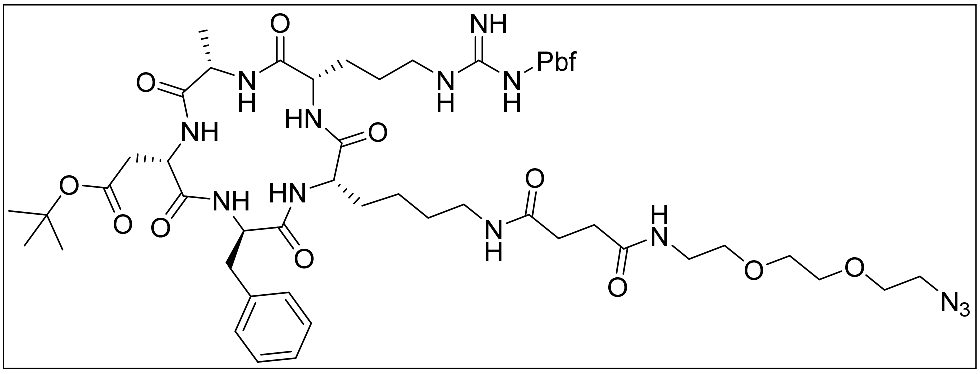

The conjugable cRADfK peptid **15** was synthesised following the same procedure as described for **14**: 2-Chlorotrityl chloride resin (1.039 g, 1.041 mmol/g resin).

The protected conjugable cRADfK peptide **15** (732.8 mg, 619.8 µmol, 57 %) was isolated as solid.

LC-MS (ESI+) m/z: 1182.6z $[M+H]^+$, 1126.5$[M-^tBu+H]^+$, 592.0 $[M+2H]^{2+}$, 564.0 $[M+2H]^2$; t_R = 8.8 min.

HRMS (ESI-MS) m/z: $[M+H]^+$ calc. for $[C_{55}H_{84}N_{13}O_{14}S]^+$: 1182.59685, found: 1182.59759; $[M+2H]^{2+}$ calc. for $[C_{55}H_{85}N_{13}O_{14}S]^{2+}$: 591.80365, found: 591.80243.

3.10 SMDC Synthesis

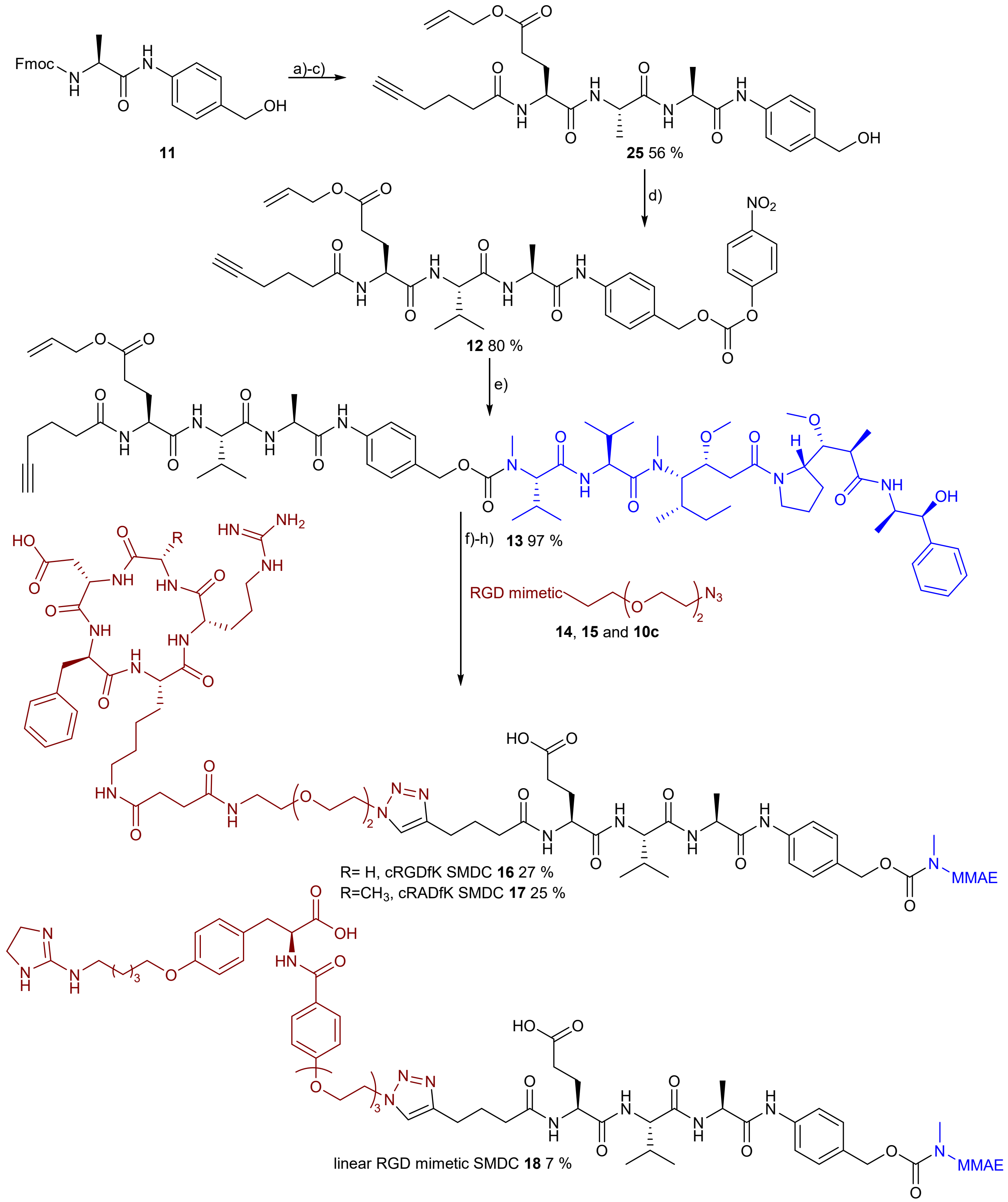

Figure S15: Synthesis of the RGD or RAD containing SMDC using **14**, **15** and **10c** as RGD mimetics and MMAE as payload. Reagents and conditions: a) pyridine, DCM, 2 d, RT; b) SPPS (1. Oxyma, DIC, DMF; 2. 20 % piperidine in DMF); c) TFA, DCM, RT; d) Bis-(*para*-nitrophenyl) carbonate, DIPEA, DMF, RT; e) MMAE, HOBt, pyridine, DMF, RT; f) TFA, TIPS, MPW, o.n., RT; g) $CuSO_4$, Na-ascorbate, DMF, water, o.n., RT; h) $Pd(PPh_3)_4$, morpholine, DMF, water, RT.

3.10.1 Fmoc-Ala-PABA (**11**)

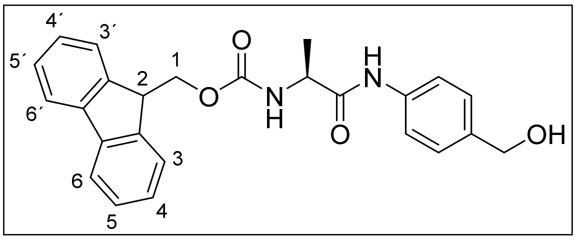

To a solution of Fmoc-Ala-OH (0.314 g, 1.0 mmol, 1 eq.) in DCM (10 mL) and MeOH (5 mL) was added 4-aminobenzyl alcohol (0.136 g, 1.1 mmol, 1.1 eq.) and EEDQ (0.545 g, 2.2 mmol, 2.2 eq.). After stirring at room temperature for 5 min the reaction solution was heated to 40 °C in a sealed tube overnight. The solvent was removed under reduced pressure and the resulting residue was treated with Et_2O to give a slurry. This was subsequently sonicated for 5 min and cooled to 4 °C for 30 min. Afterwards the slurry was filtered and the solid was washed with a small amount of cooled Et_2O. The product was dried in vacuum to result in Fmoc-Ala-PABA **11** (0.386 g, 0.93 mmol, 92 %) as a light grey solid.

^{1}H-NMR (500 MHz, DMCO-d_6) δ [ppm] = 9.94 (s, 1H, Ala-N**H**-PABA), 7.89 (d, ^3J = 7.6 Hz, 2H, Fmoc-6/6′-**H**), 7.74 (t, ^3J = 8.3 Hz, 2H, Fmoc-3/3′-**H**), 7.66 (d, ^3J = 7.4 Hz, 1H, Fmoc-N**H**-Ala), 7.55 (d, ^3J = 8.1 Hz, 2H, PABA-3/3′-**H**), 7.42 (t, ^3J = 7.6 Hz, 2H, Fmoc-5/5′-**H**), 7.33 (dd, ^3J = 6.8 Hz, 2H, Fmoc-4/4′-**H**), 7.24 (d, ^3J = 8.1 Hz, 2H, PABA-2/2′-**H**), 5.09 (t, ^3J = 5.7 Hz, 1H, PABA-CH_2O**H**), 4.43 (d, ^3J = 5.7 Hz, 2H, PABA-C**H**$_2$OH), 4.28 (d, ^3J = 7.1 Hz, 2H, Fmoc-1-**H**), 4.25 – 4.17 (m, 2H, Fmoc-2-**H**, Ala-**H**^{α}), 1.30 (d, ^3J = 7.1 Hz, 3H, Ala-**H**^{β}).

3.10.2 5-Hexynoyl-Glu(All)-Val-Ala-PABA (**25**)

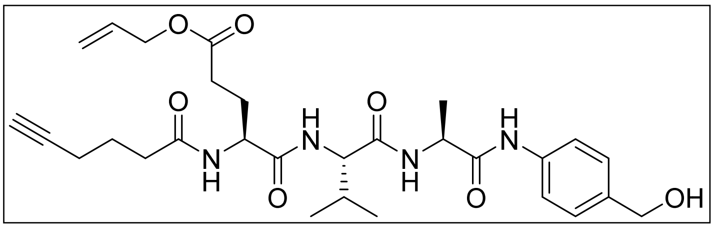

2-Chlorotrityl chloride resin (1.023 g) was swelled in dry DCM (10 mL) for 10 min and then the solvent was replaced by Fmoc-Ala-PABA **11** (477.8 mg, 1.2 mmol) dissolved in a mixture of DCM and pyridine (1:1, v:v, 5 mL) followed by shaking at RT for 2 d. The resin loading (0.585 mmol/g resin) was determined and the further amino acids as well as hexenoic acid (2 eq.) were coupled following **GP-6**. The linear peptide was cleaved with TFA in DCM (1 %, 7x3 mL for 2min) whereupon the cleavage cocktail was transferred into a solution of pyridine in methanol (10 %, 20 mL) after each step. The solvent was reduced in vacuum and the residue was precipitated in water. After centrifugation the solid was dissolved in acetonitrile/water (1:1, v:v) and freeze-dried to obtain the "clickable" linker (186.7 mg, 335.4 mmol, 56 %) as a fluffy colourless solid.

LC-MS (ESI+) m/z: 557.3 $[M+H]^+$, 539.3 $[M-H_2O]^+$; t_R = 7.1 min.

HRMS (ESI-MS) m/z: $[M+H]^+$ calc. for $[C_{29}H_{41}N_4O_7]^+$: 557.29698, found: 557.29750; $[M-H_2O]^+$ calc. for $[C_{29}H_{39}N_4O_6]^+$: 539.28742, found: 539.28641.

3.10.3 5-Hexynoyl-Glu(All)-Val-Ala-PABA-PNPC (12)

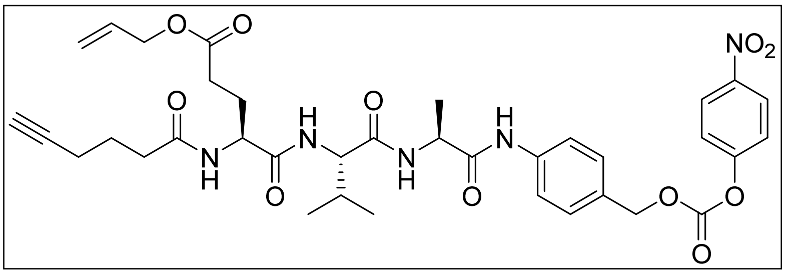

The benzyl alcohol **25** (186.7 mg, 335.4 µmol, 1 eq.) and bis(4-nitrophenyl)carbonate (256.7 mg, 843.9 µmol, 2.5 eq.) were dissolved in dry DMF (6 mL), followed by addition of DIPEA (114.1 µL, 670.9 µmol, 2 eq.). The reaction mixture was stirred for 3 h and the reaction progress was monitored using LCMS. After full conversion of the starting material the reaction solution was added to a water/ACN/TFA solution (5:1+0,5 % TFA) and immediately frozen and freeze-dried. The resulting solid was purified by column chromatography (DCM -> DCM/MeOH/TFA (95/4.9/0.1)) to give the activated linker **12** (194.4 mg, 269.3 µmol, 80 %) as a solid.

LC-MS (ESI+) m/z: 744.3 $[M+Na]^+$, 722.3 $[M+H]^+$, 539.3 $[M-C_7H_4NO_5]^+$; t_R = 9.6 min.

HRMS (ESI-MS) m/z: $[M+Na]^+$ calc. for $[C_{36}H_{44}N_5NaO_{11}]^+$: 744.28573, found: 744.28513; $[M+H]^+$ calc. for $[C_{36}H_{44}N_5O_{11}]^+$: 722.30431, found: 722.30319.

3.10.4 5-Hexynoyl-Glu(All)-Val-Ala-PABA-MMAE (13)

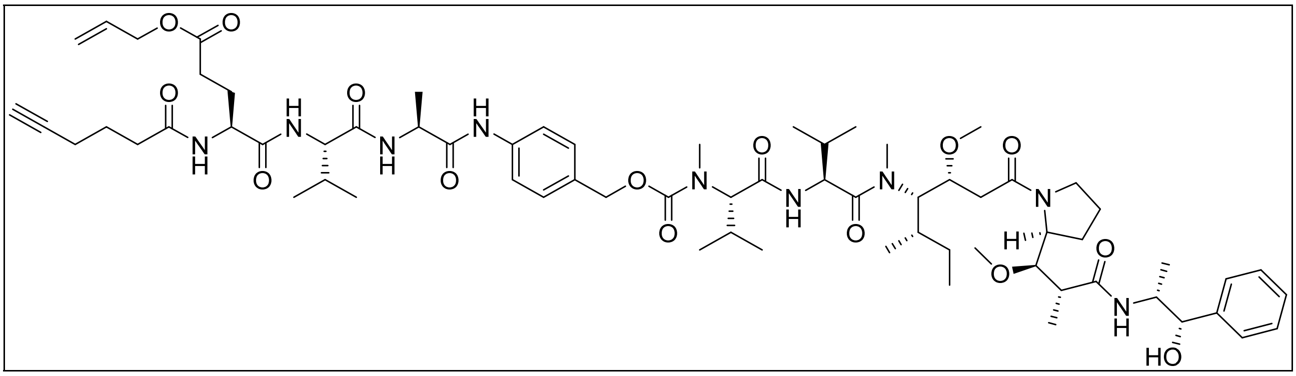

A solution of activated linker **12** (59.9 mg, 83.0 µmol, 1.1 eq.) and HOBt (1.3 mg, 8.42 µmol, 0.1 eq.) in dry DMF (400 µL) was added to a solution of MMAE (54.01 mg, 75.23 µmol, 1 eq.) in dry DMF (400 µL), followed by addition of pyridine (200 µL). The reaction mixture was stirred at room temperature till full conversion of MMAE was observed by LCMS. Afterwards the reaction solution was diluted with MPW and freeze-dried. The crude product was then purified by column chromatography (DCM->DCM/MeOH (90:10, v:v)) to give the linker-MMAE product **13** (94.6 mg, 72.7 µmol, 97 %) as a colourless solid.

LC-MS (ESI+) m/z: 1322.8 $[M+Na]^+$, 1300.8 $[M+H]^+$, 762.5 $[MMAE+carbonate+H]^+$ ($C_{40}H_{68}N_5O_9$), 718.5 $[MMAE+H]^+$, 662.1 $[M+Na+H]^{2+}$, 650.1 $[M+2H]^{2+}$, 539.3 $[linker-carbonat+H]^+$ ($C_{29}H_{39}N_4O_6$), 434.3 $[M+3H]^{3+}$; t_R = 9.8 min.

HRMS (ESI-MS) m/z: $[M+H]^+$ calc. for $[C_{69}H_{106}N_9O_{15}]^+$: 1300.78009, found: 1300.78029; $[M+Na+H]^{2+}$ calc. for $[C_{69}H_{106}N_9NaO_{15}]^{2+}$: 661.88347, found: 661.88476; $[M+2H]^{2+}$ calc. for $[C_{69}H_{107}N_9O_{15}]^{2+}$: 650.89212, found: 650.89378.

3.10.5 c(RGDfK)-PEG-Glu-Val-Ala-PABA-MMAE (**16**)

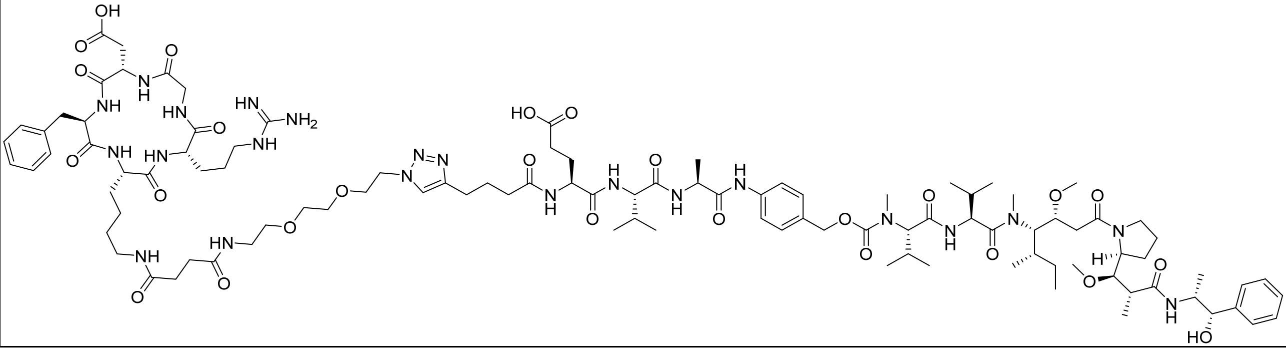

Synthesis according to **GP-9**: Prepared from **14** (17.5 mg, 15.0 µmol, 2.3 eq.) and **13** (8.5 mg, 6.5 µmol, 1 eq.).

The desired compound **16** (4.3 mg, 2.0 µmol, 27 %) was obtained as a colourless fluffy TFA salt.

LC-MS (ESI+) m/z: 2122.2 $[M+H]^+$, 1061.8 $[M+2H]^{2+}$, 708.0 $[M+3H]^{3+}$; t_R = 7.4 min.

HRMS (ESI-MS) m/z: $[M+H]^+$ calc. for $[C_{103}H_{159}N_{22}O_{26}]^+$: 2120.17306, found: 2120.17904; $[M+2H]^{2+}$ calc. for $[C_{103}H_{160}N_{22}O_{26}]^{2+}$: 1060.59772, found: 1060.59316; $[M+3H]^{3+}$ calc. for $[C_{103}H_{161}N_{22}O_{26}]^{3+}$: 707.40035, found: 707.39786.

3.10.6 c(RADfK)-PEG-Glu-Val-Ala-PABA-MMAE (**17**)

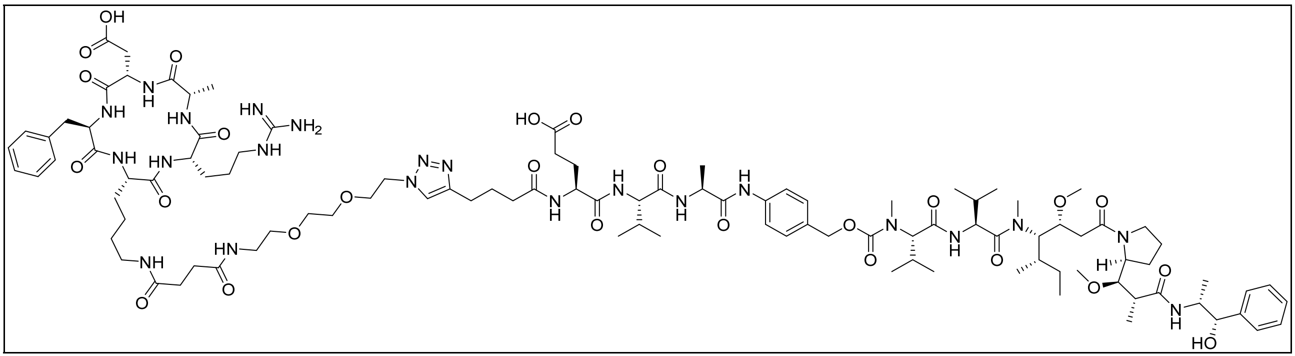

Synthesis according to **GP-9**: Prepared from **15** (17.5 mg, 14.8 µmol, 2.2 eq.) and **13** (8.6 mg, 6.6 µmol, 1 eq.).

The desired compound **17** (4.0 mg, 1.9 µmol, 25 %) was obtained as a colourless fluffy TFA salt.

LC-MS (ESI+) m/z: 2136.2 $[M+H]^+$, 1068.6 $[M+2H]^{2+}$, 712.7 $[M+3H]^{3+}$; t_R = 7.5 min.

HRMS (ESI-MS) m/z: [M+H]$^{+}$ calc. for [$C_{104}H_{161}N_{22}O_{26}$]$^{+}$: 2134.19585, found: 2134.19469; [M+2H]$^{2+}$ calc. for [$C_{104}H_{162}N_{22}O_{26}$]$^{2+}$: 1067.60269, found: 1067.60098; [M+3H]$^{3+}$ calc. for [$C_{104}H_{163}N_{22}O_{26}$]$^{3+}$: 712.06702, found: 712.06975.

3.10.7 Linear RGD-PEG-Glu-Val-Ala-PABA-MMAE (**18**)

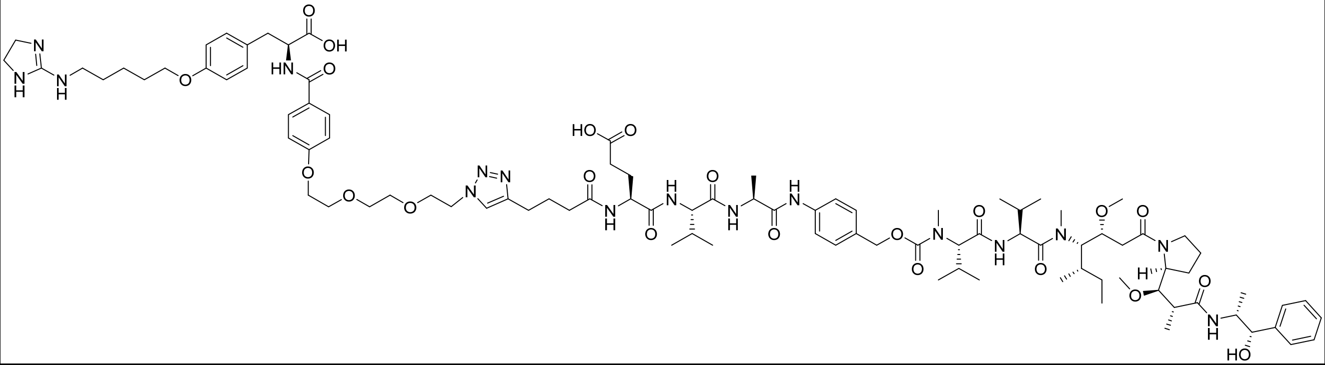

Synthesis according to **GP-9**: Prepared from **10c** (7.3 mg, 10.0 µmol, 1.3 eq.) and **13** (8.6 mg, 6.6 µmol, 1 eq.) without prior Pbf/^{t}Bu-cleavage.

The desired compound **18** (1.1 mg, 0.5 µmol, 7 %) was obtained as a colourless fluffy TFA salt.

LC-MS (ESI+) m/z: 936,8 [M+2H]$^{2+}$, 625.0 [M+3H]$^{3+}$; t_R = 8.1 min.

HRMS (ESI-MS) m/z: [M+2H]$^{2+}$ calc. for [$C_{96}H_{144}N_{16}O_{22}$]$^{2+}$: 936.53015, found: 936.53151; [M+3H]$^{3+}$ calc. for [$C_{96}H_{145}N_{16}O_{22}$]$^{3+}$: 624.68889, found: 624.69010.

4 Spectroscopic and Analytical Data

5a

1H-NMR

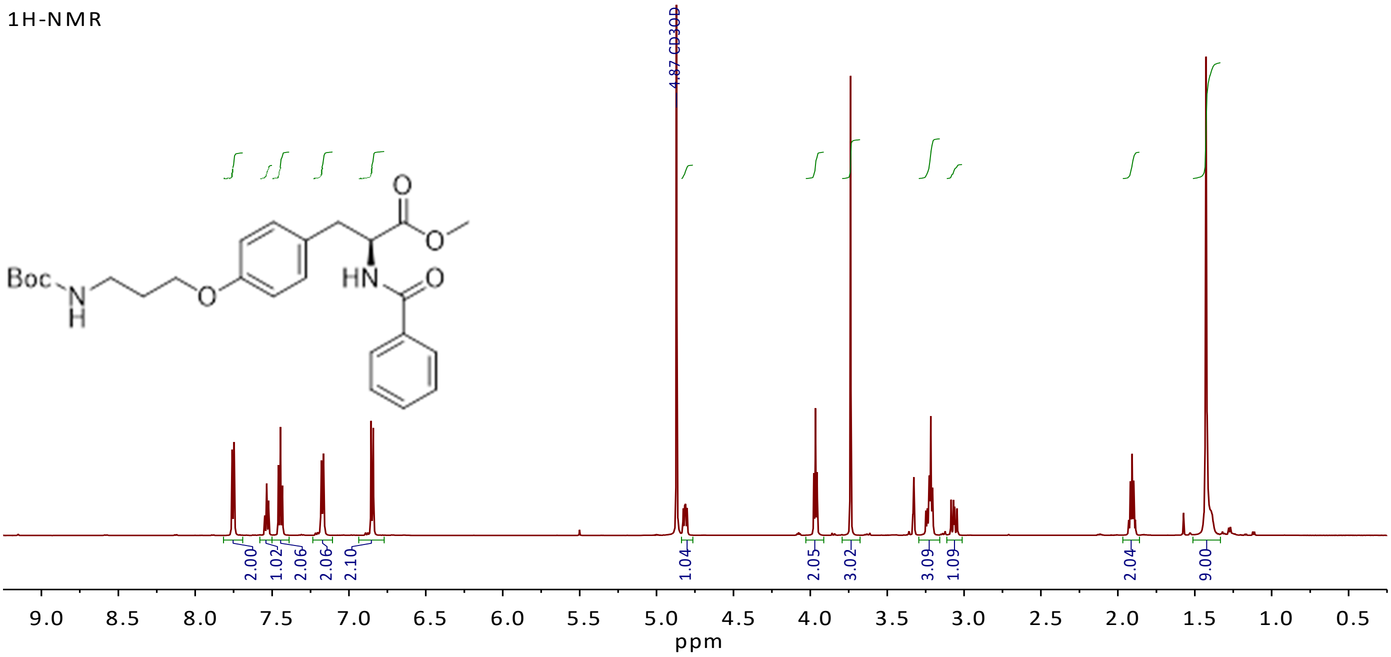

13C-NMR

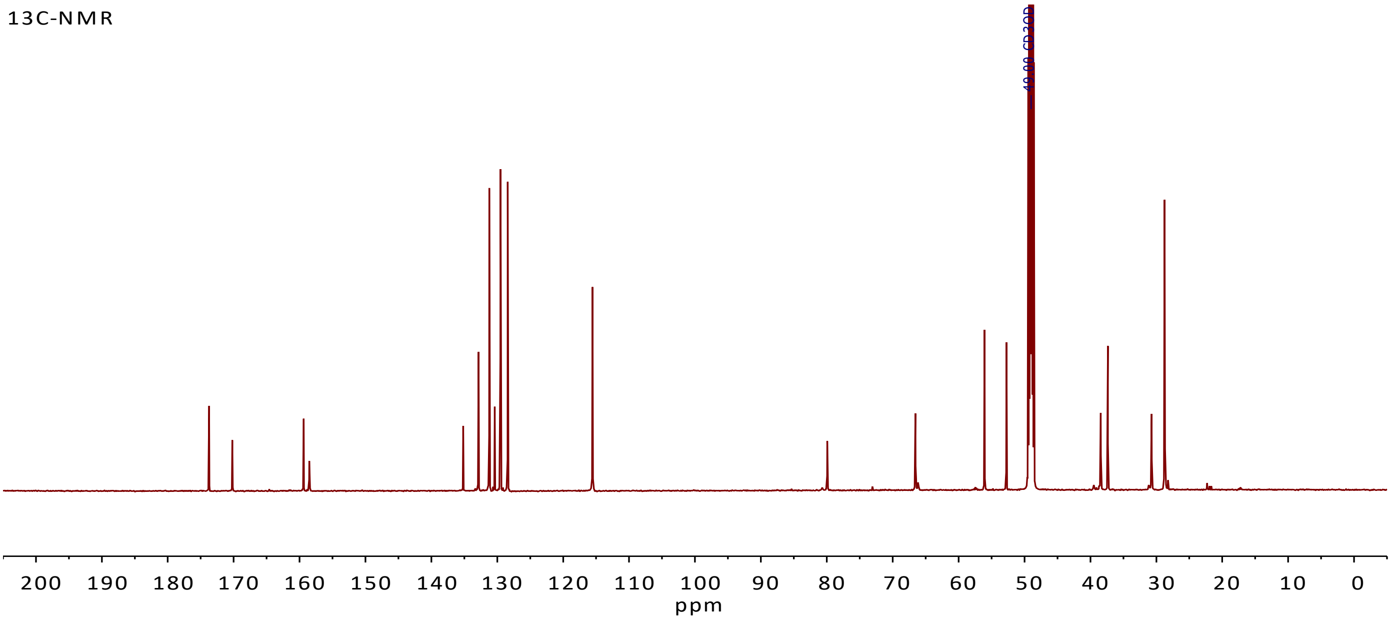

254 nm (LC-MS)

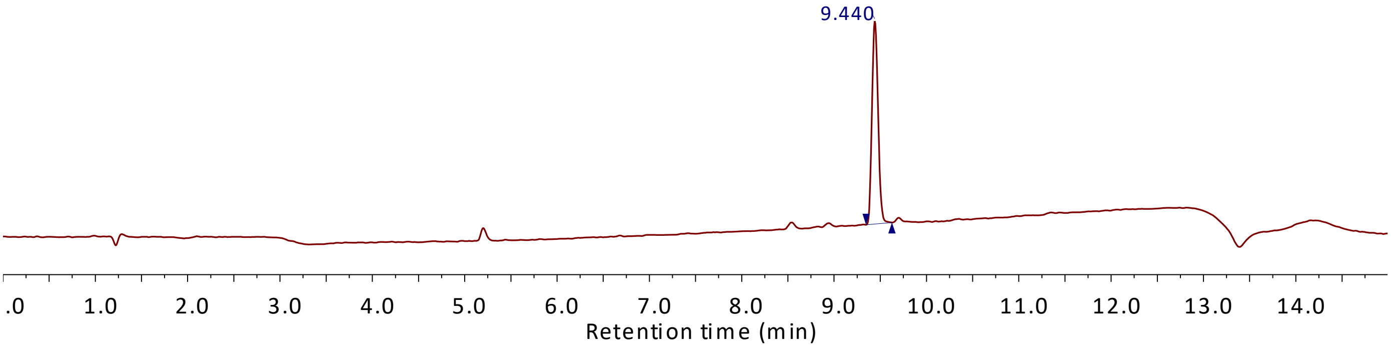

5b

1H-NMR

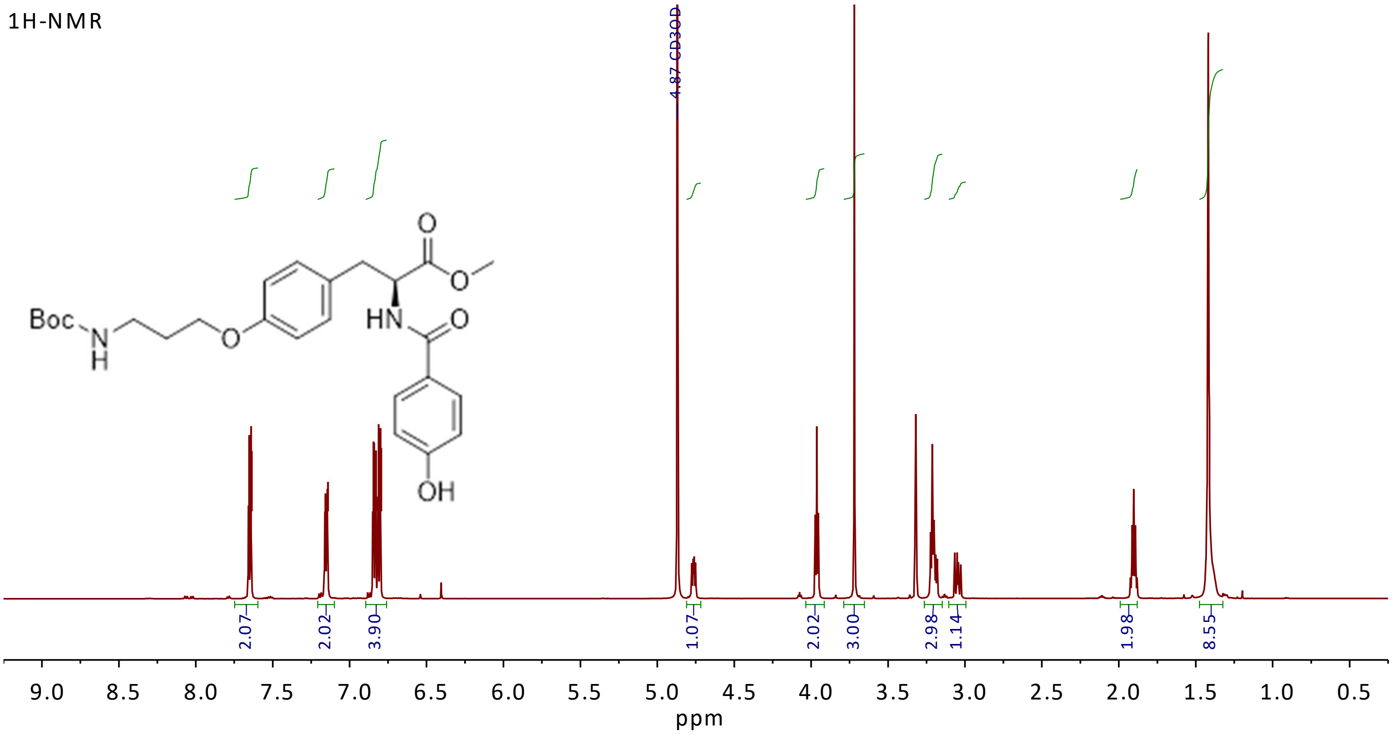

13C-NMR

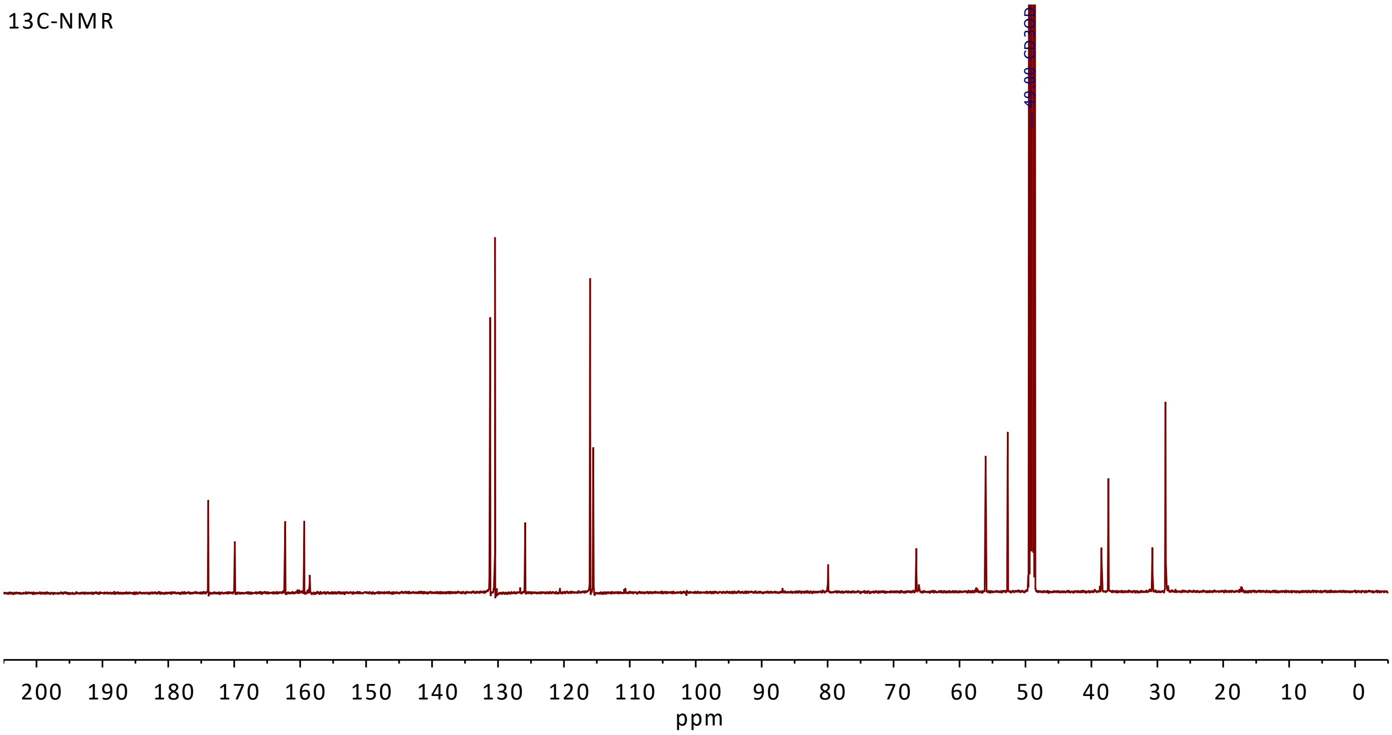

254 nm (LC-MS)

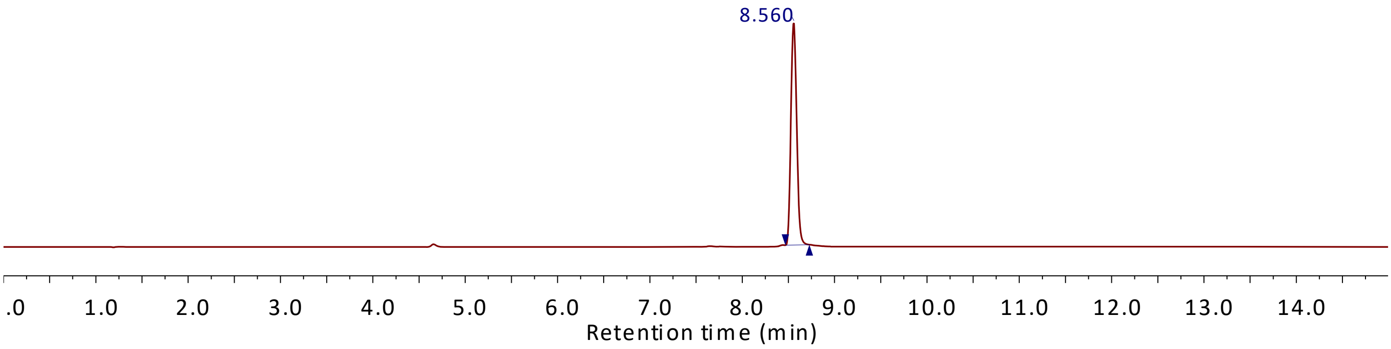

5c

1H-NMR

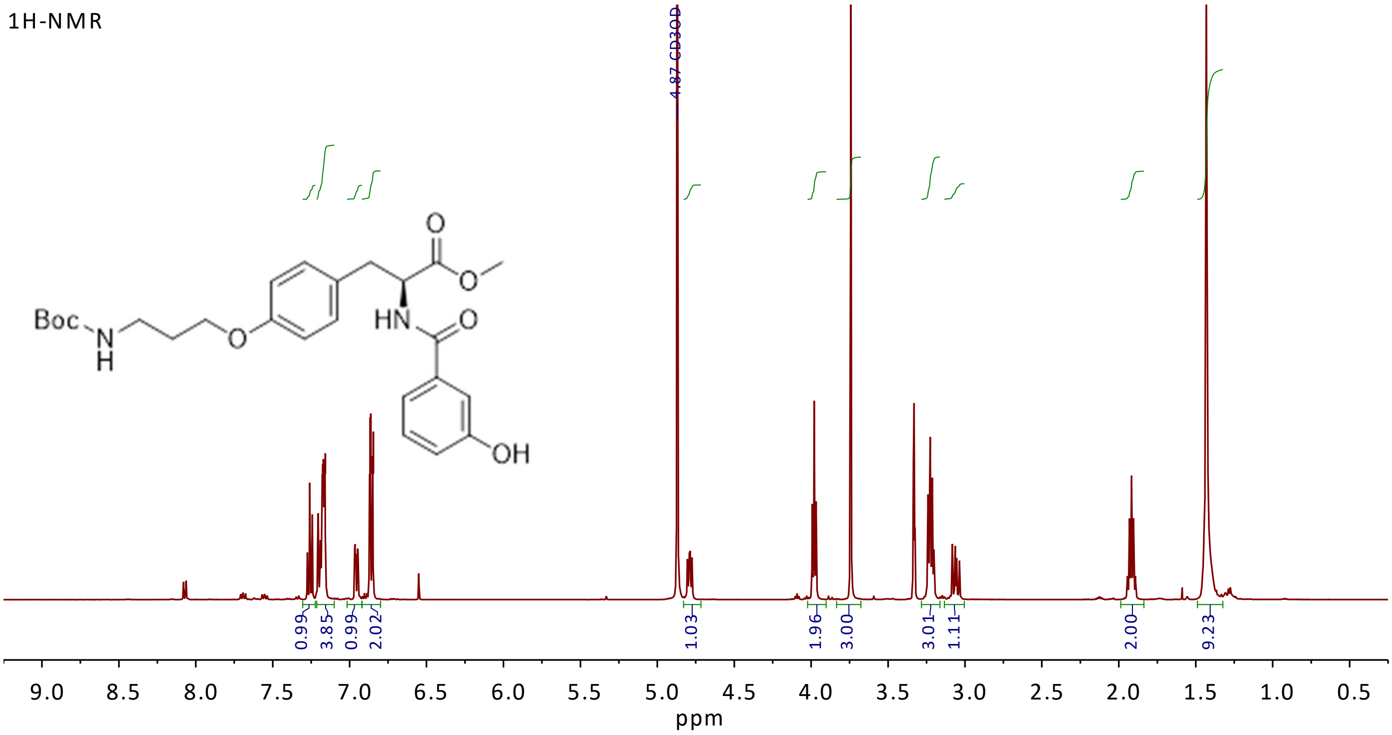

13C-NMR

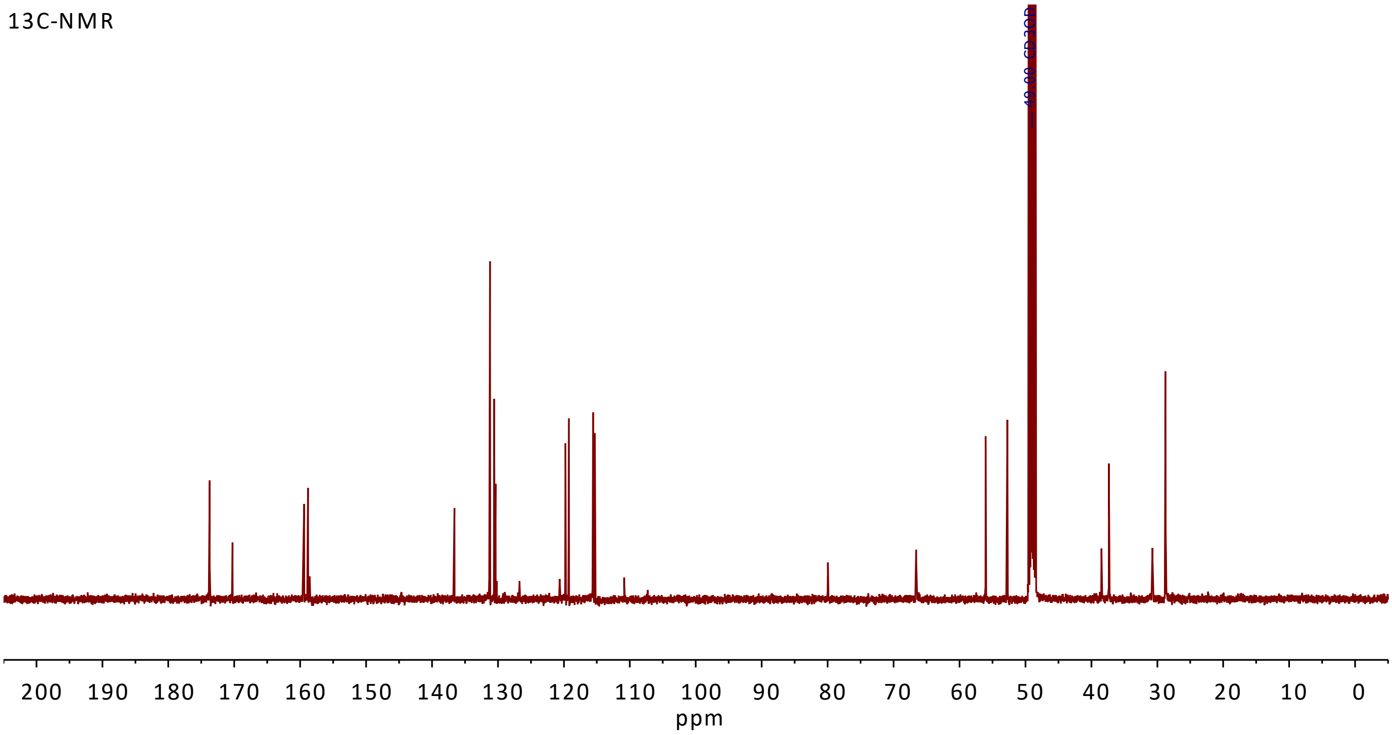

220 nm (LC-MS)

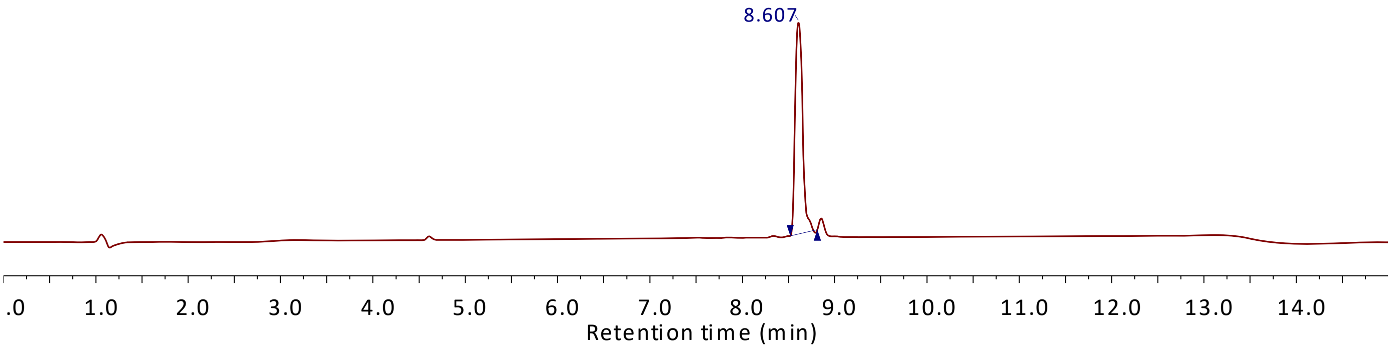

3a

1H-NMR

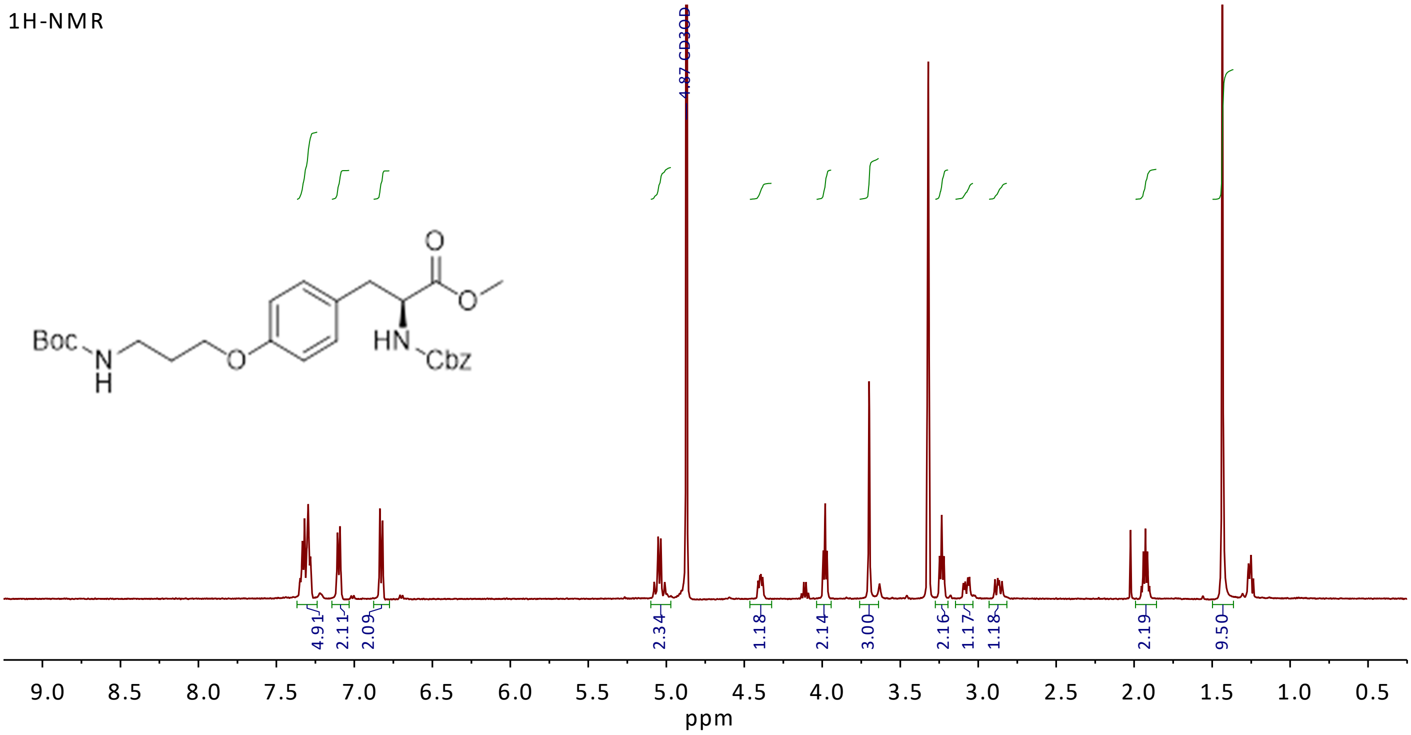
4.87 CD3OD
Boc
N
H
O
O
O
HN
Cbz
4.91
2.11
2.09
2.34
1.18
2.14
3.00
2.16
1.17
1.18
2.19
9.50
9.0
8.5
8.0
7.5
7.0
6.5
6.0
5.5
5.0
4.5
4.0
3.5
3.0
2.5
2.0
1.5
1.0
0.5
ppm

6a

1H-NMR

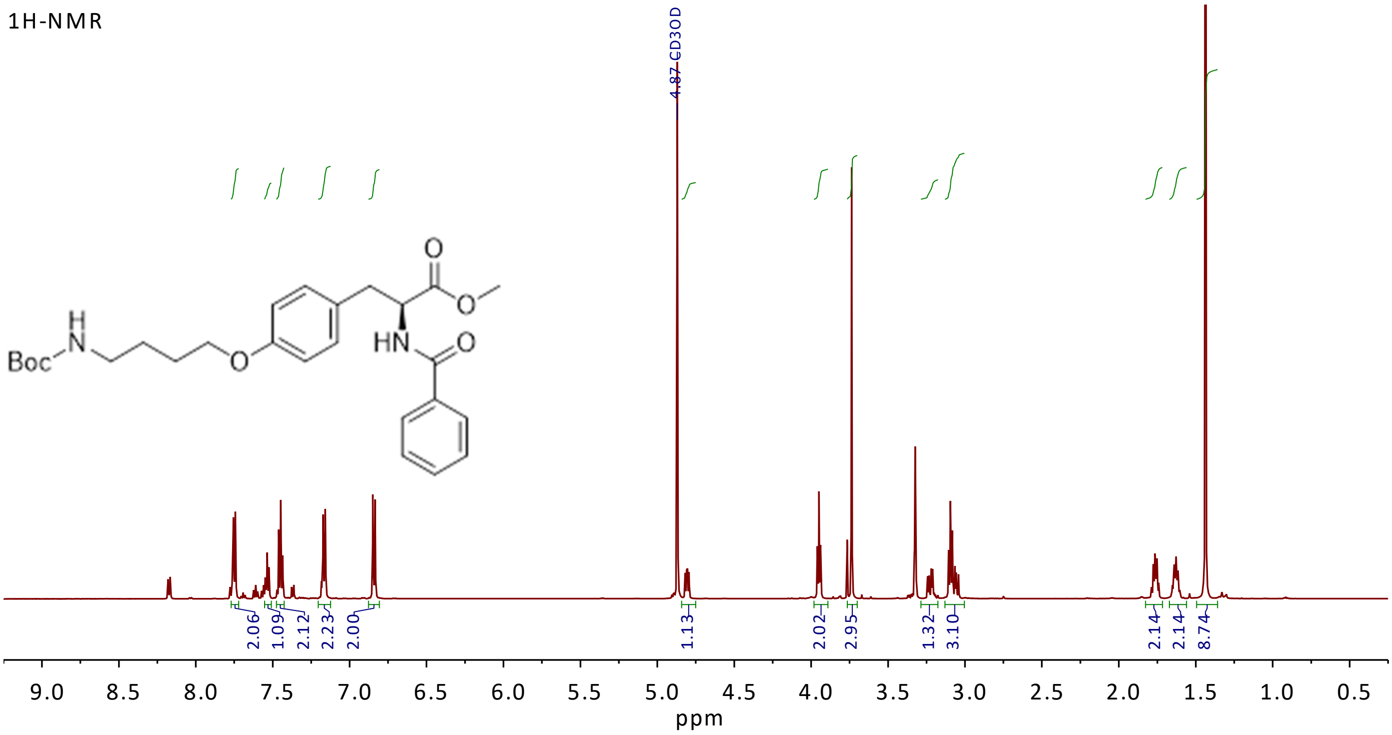

13C-NMR

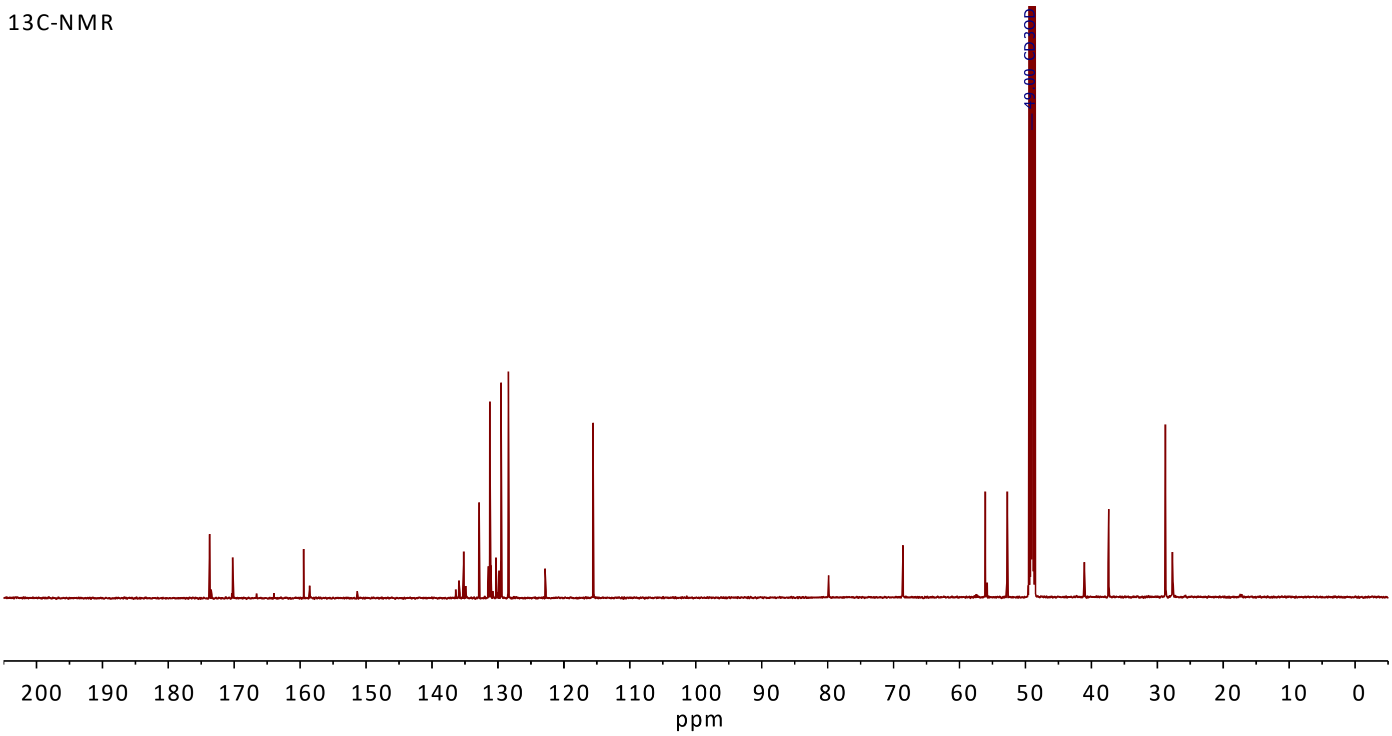

220 nm (LC-MS)

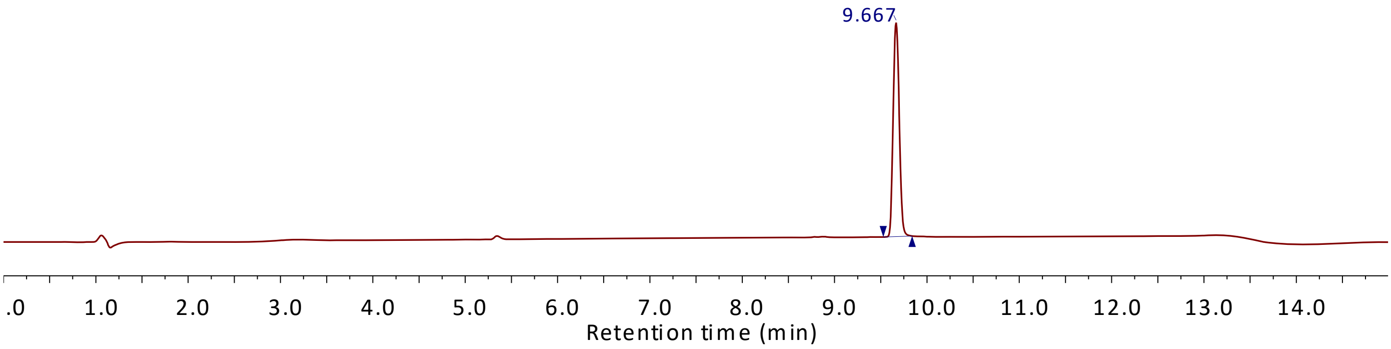

6b

1H-NMR

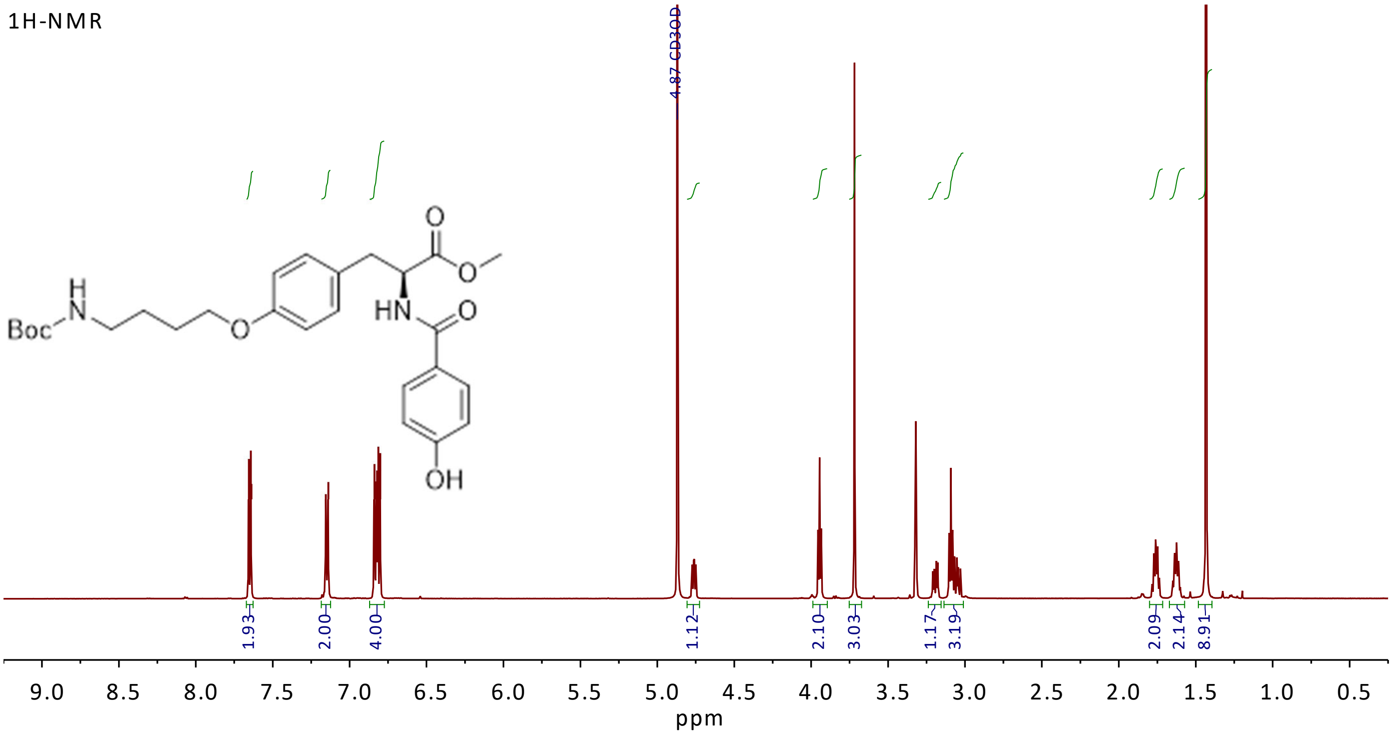

13C-NMR

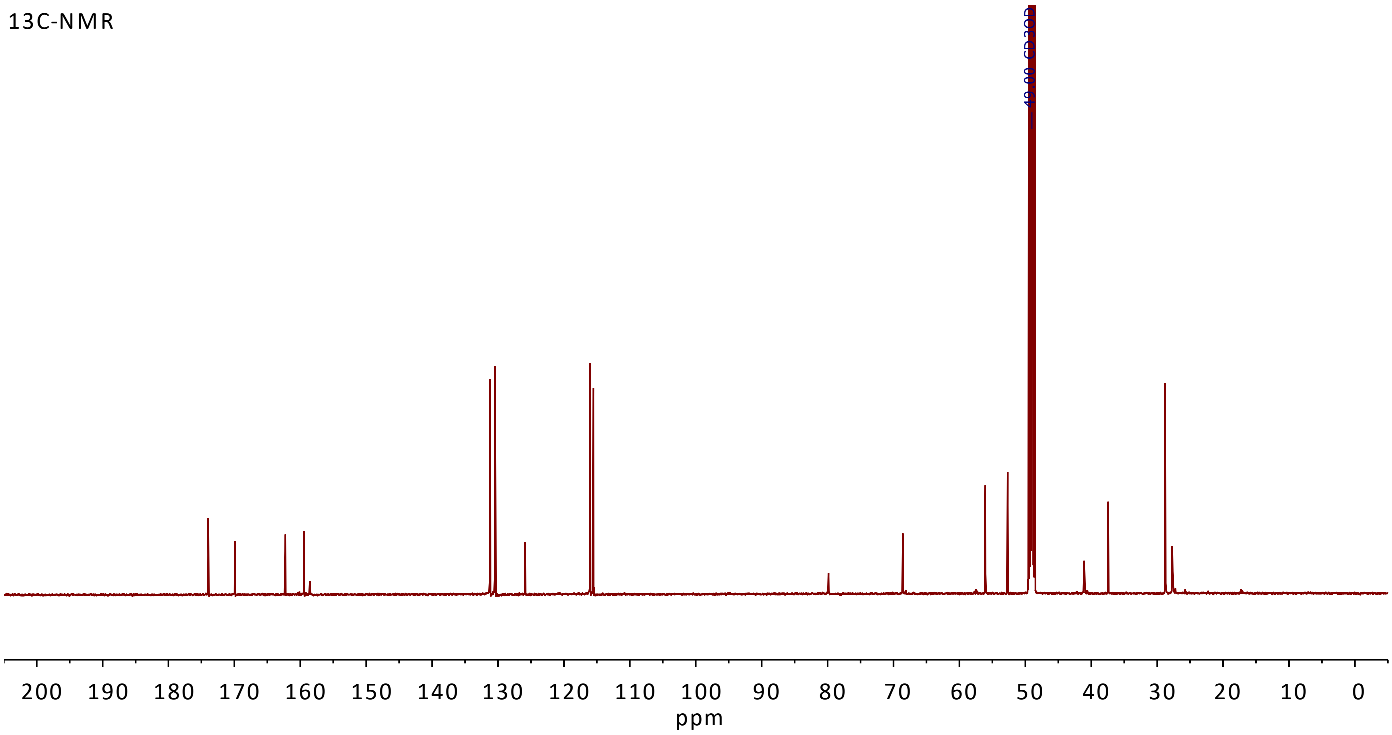

254 nm (LC-MS)

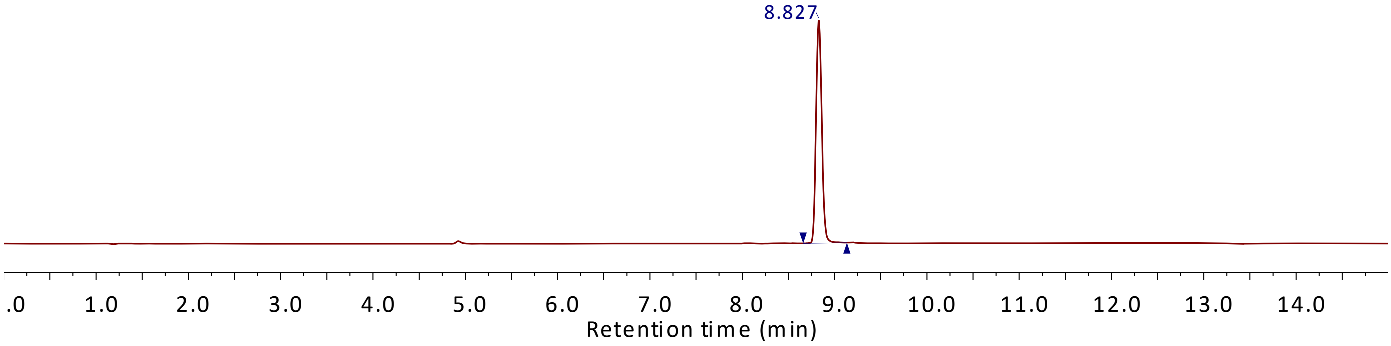

6c

1H-NMR

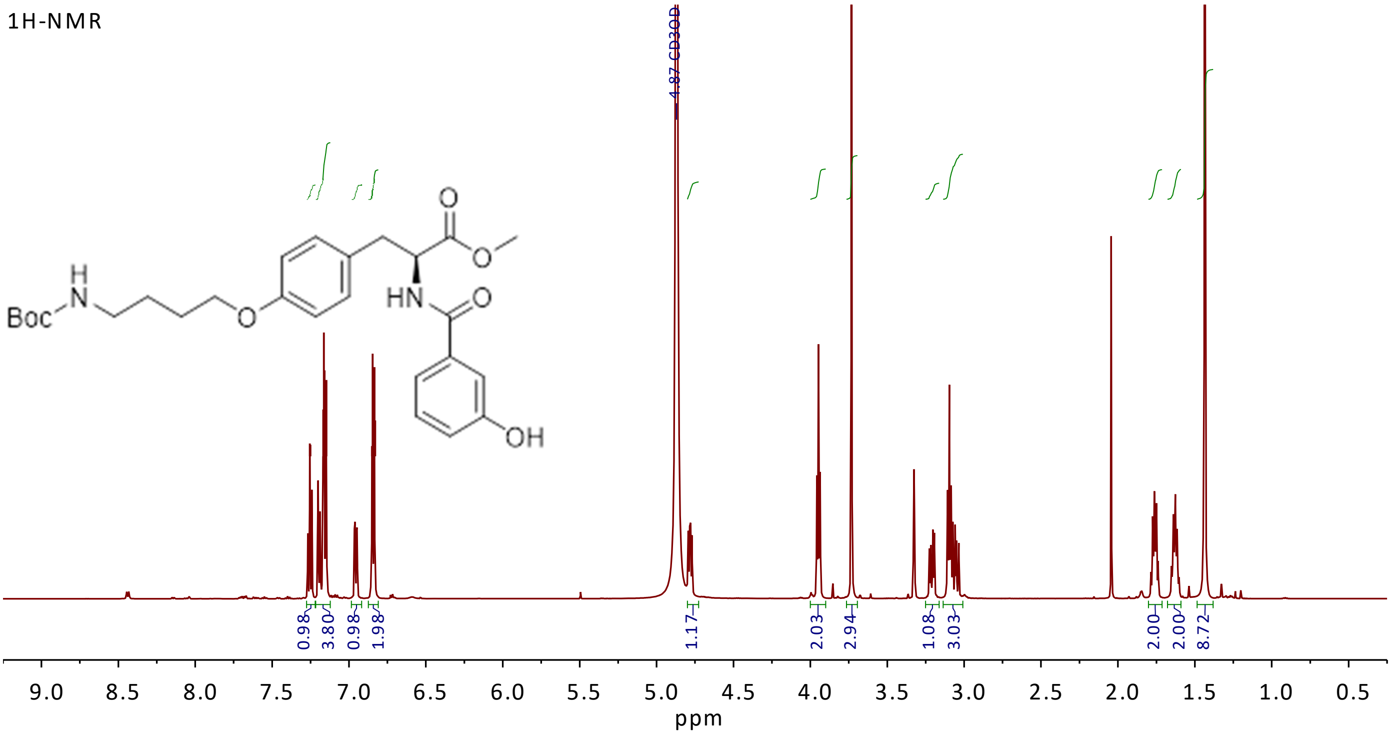

13C-NMR

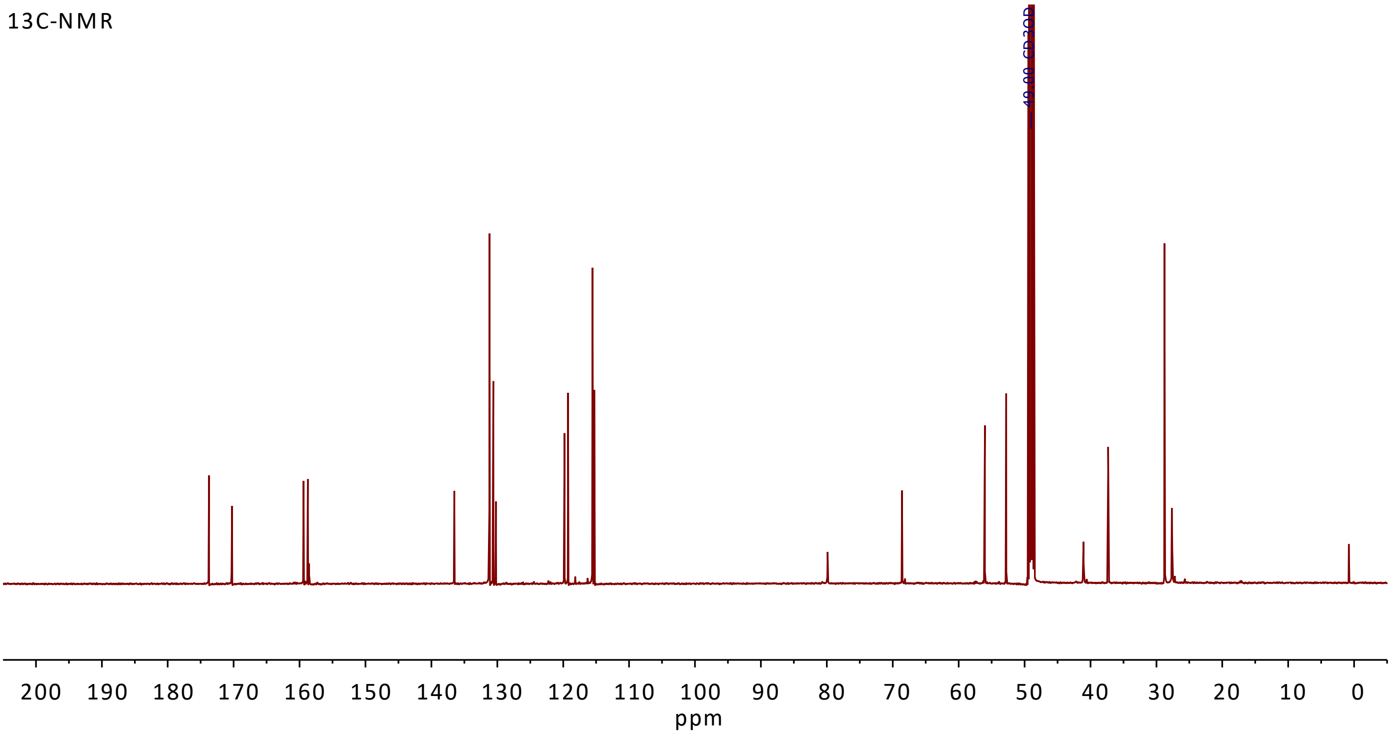

220 nm (LC-MS)

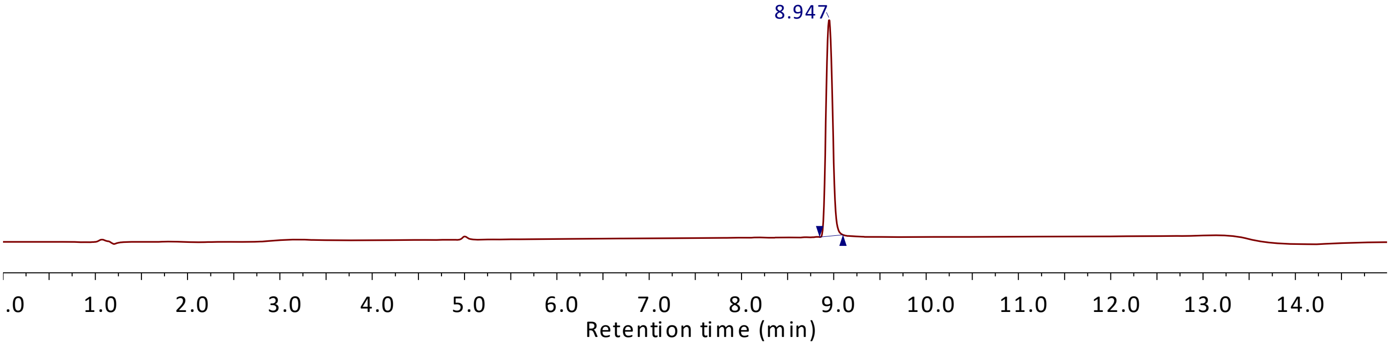

3b

1H-NMR

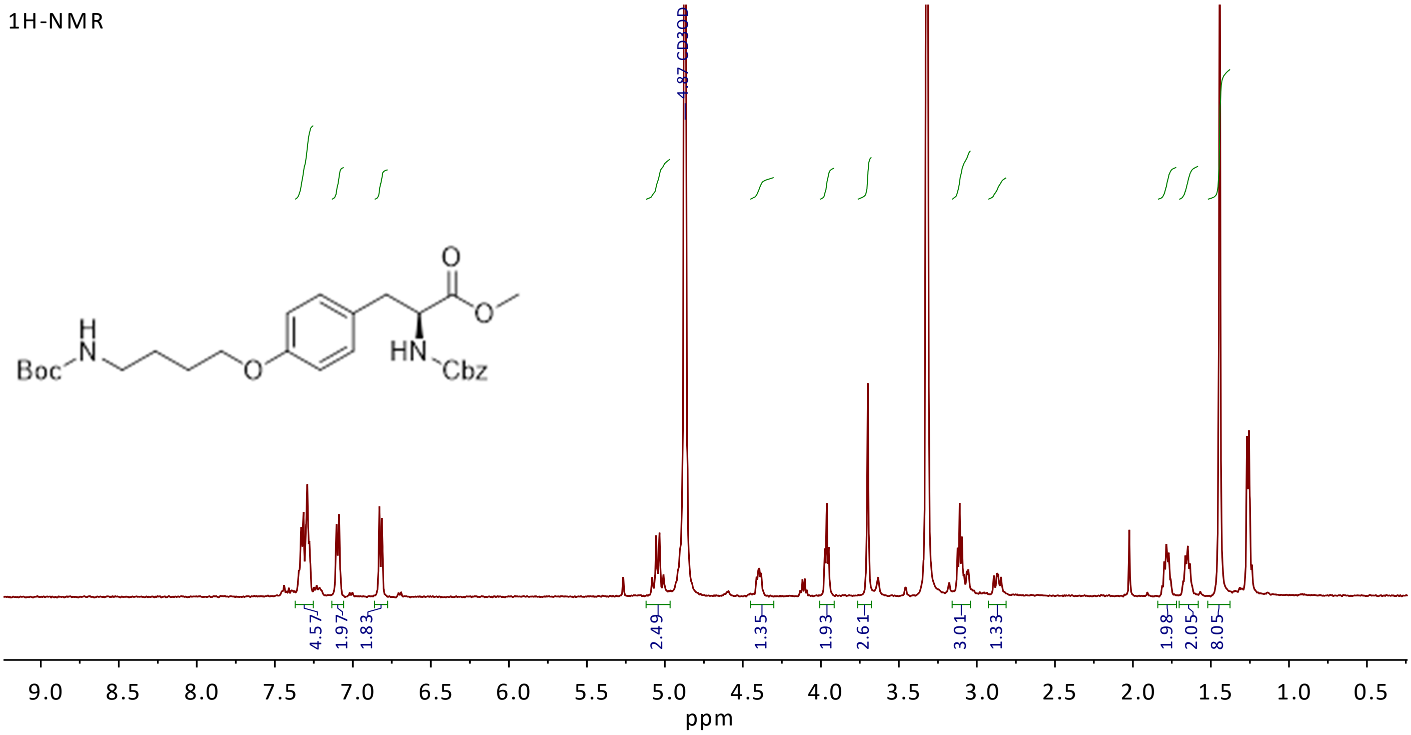

220 nm (LC-MS)

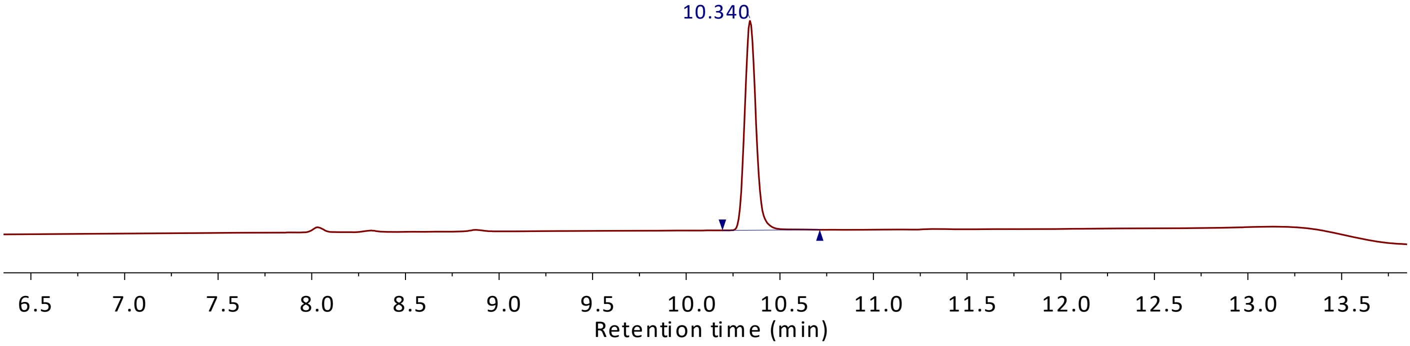

7a

1H-NMR

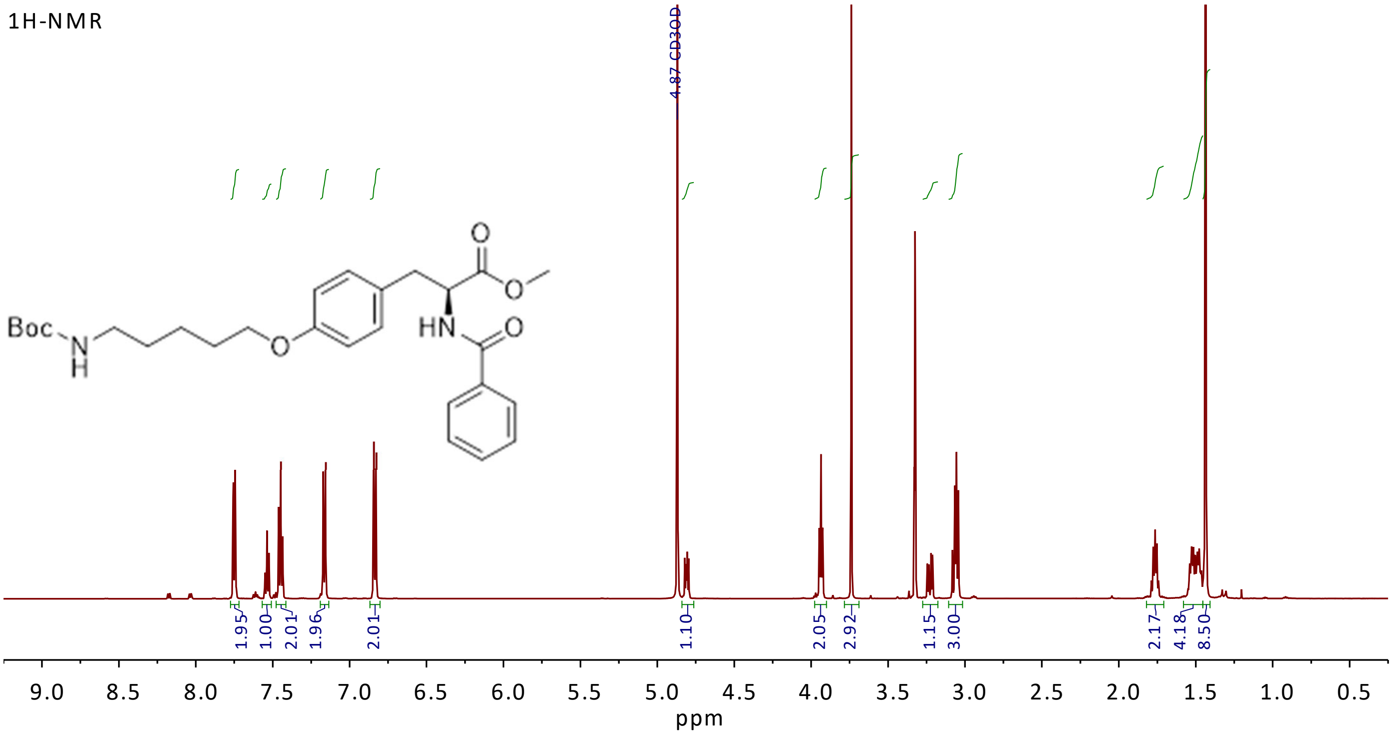

13C-NMR

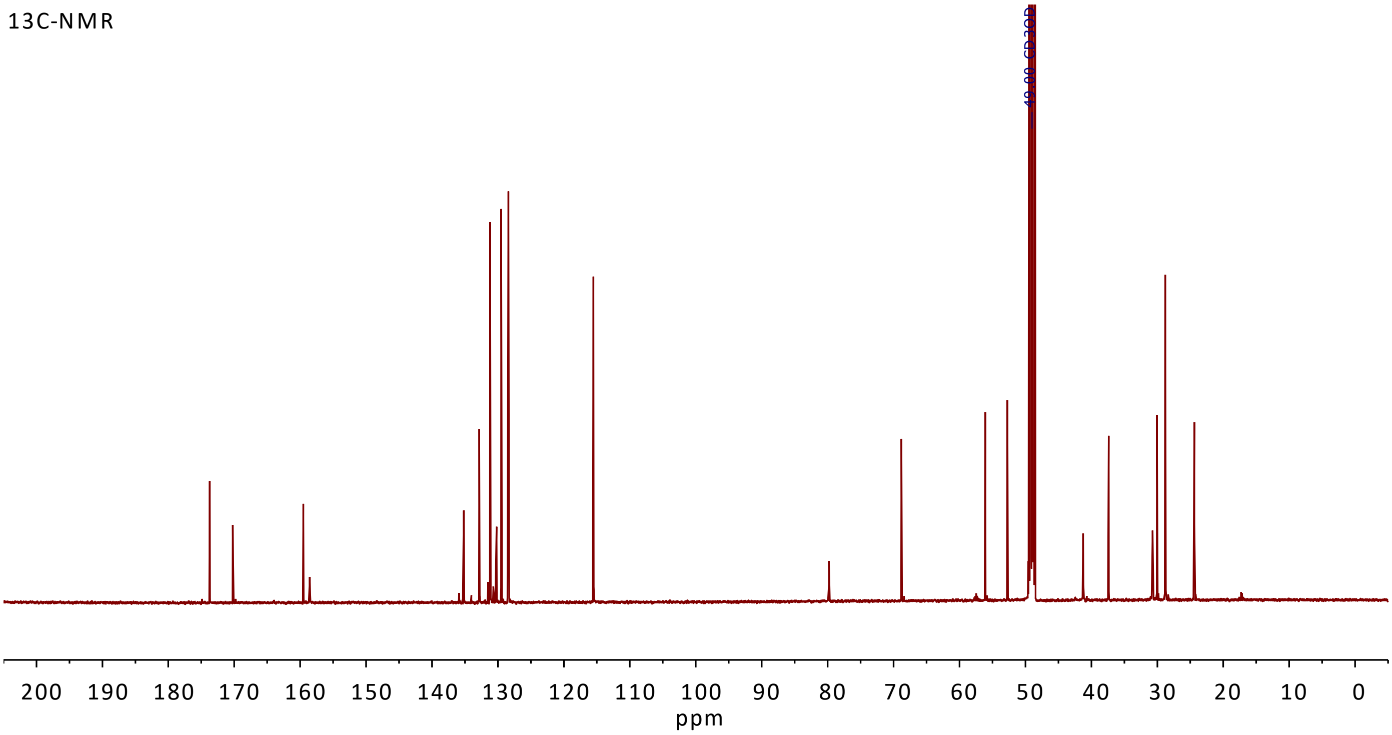

220 nm (LC-MS)

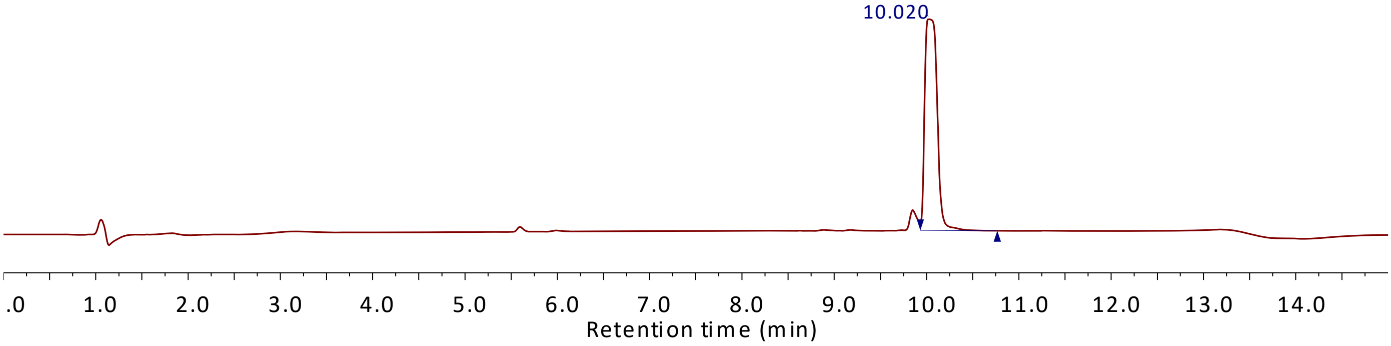

7b

1H-NMR

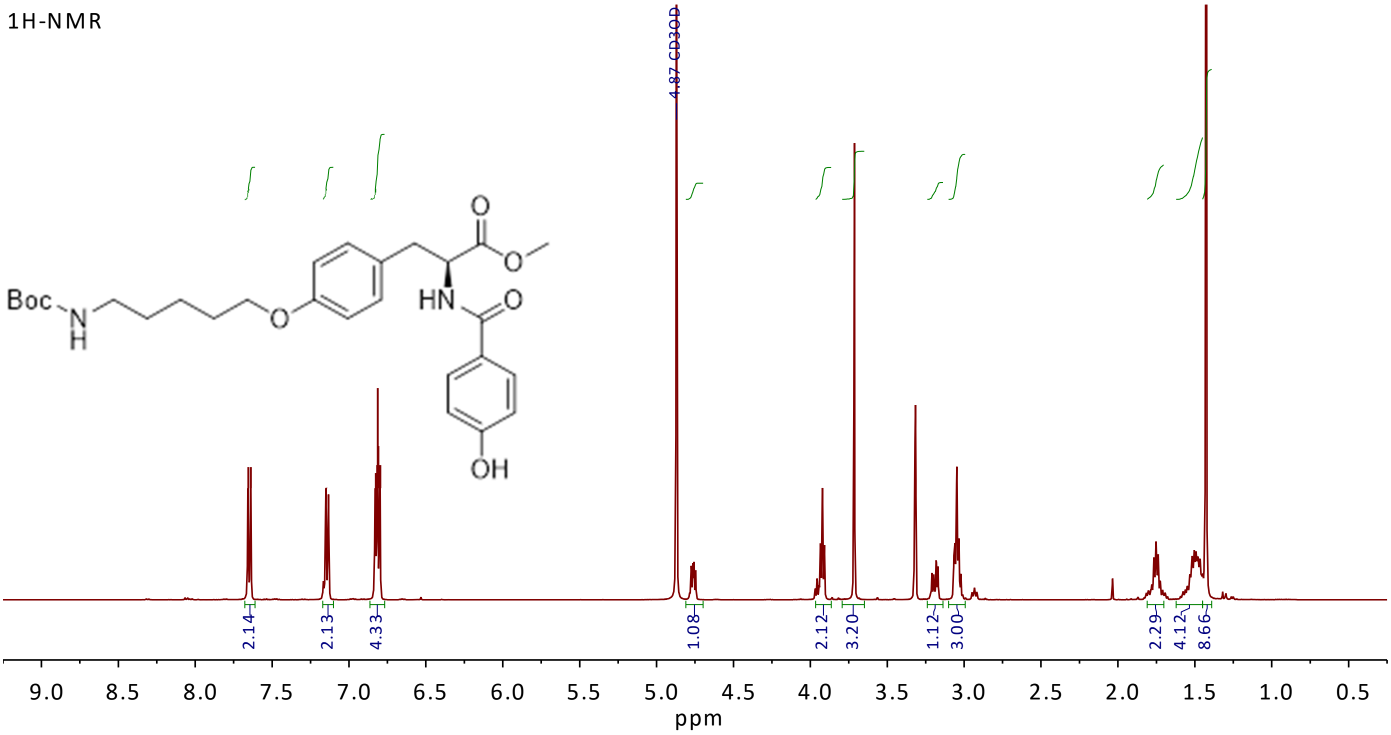

13C-NMR

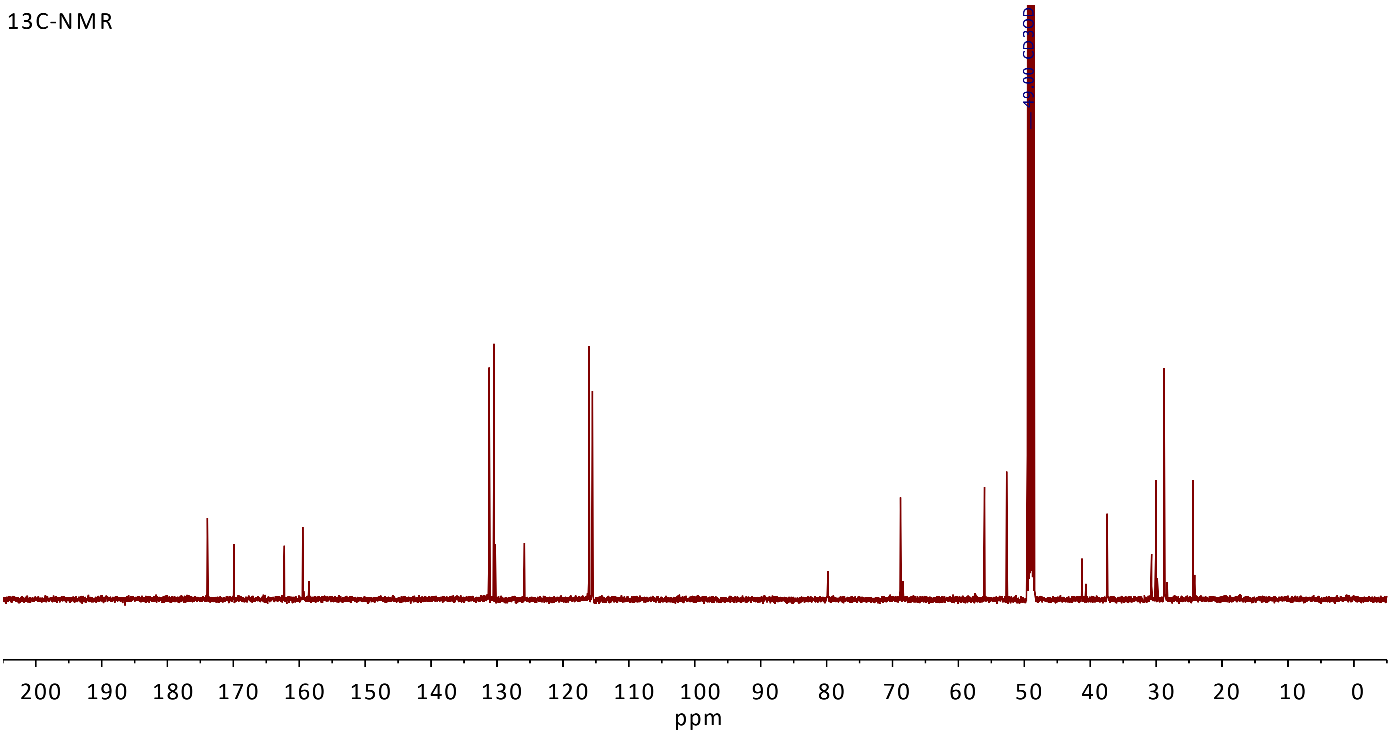

254 nm (LC-MS)

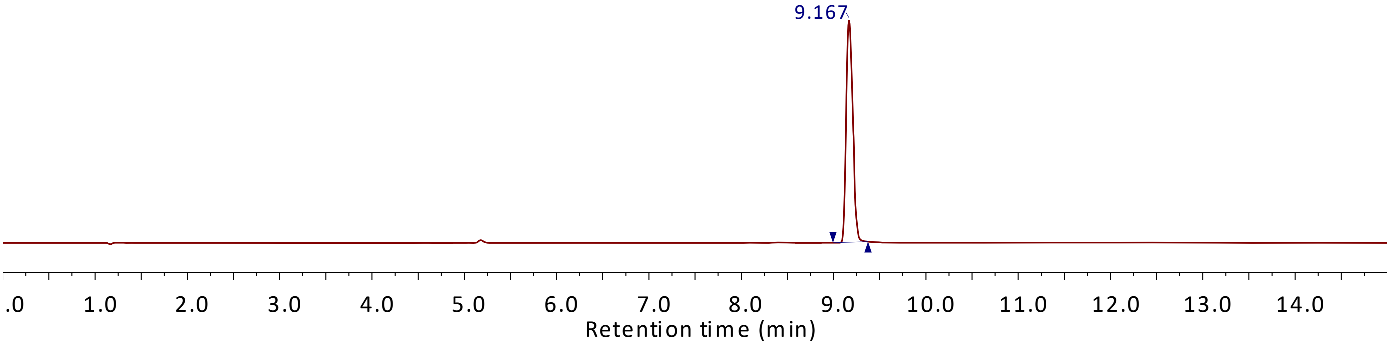

7c

1H-NMR

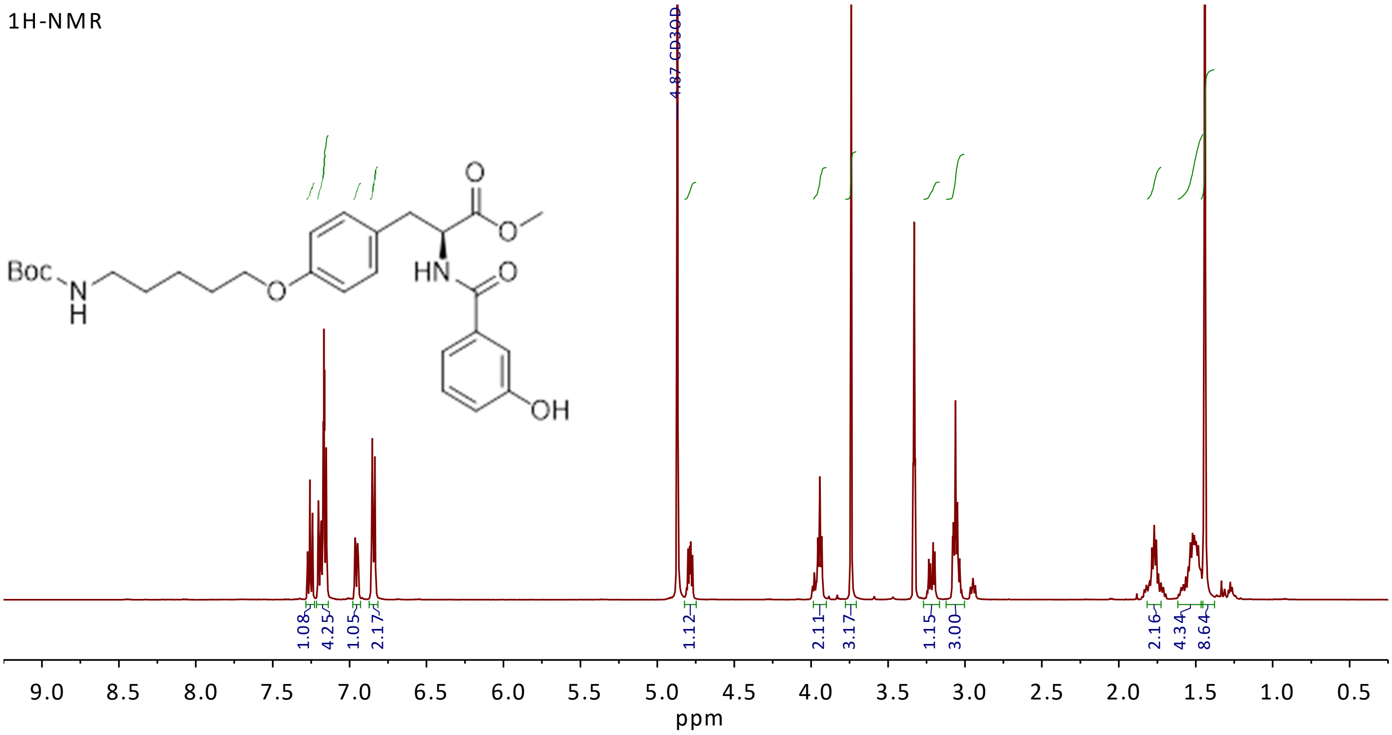

13C-NMR

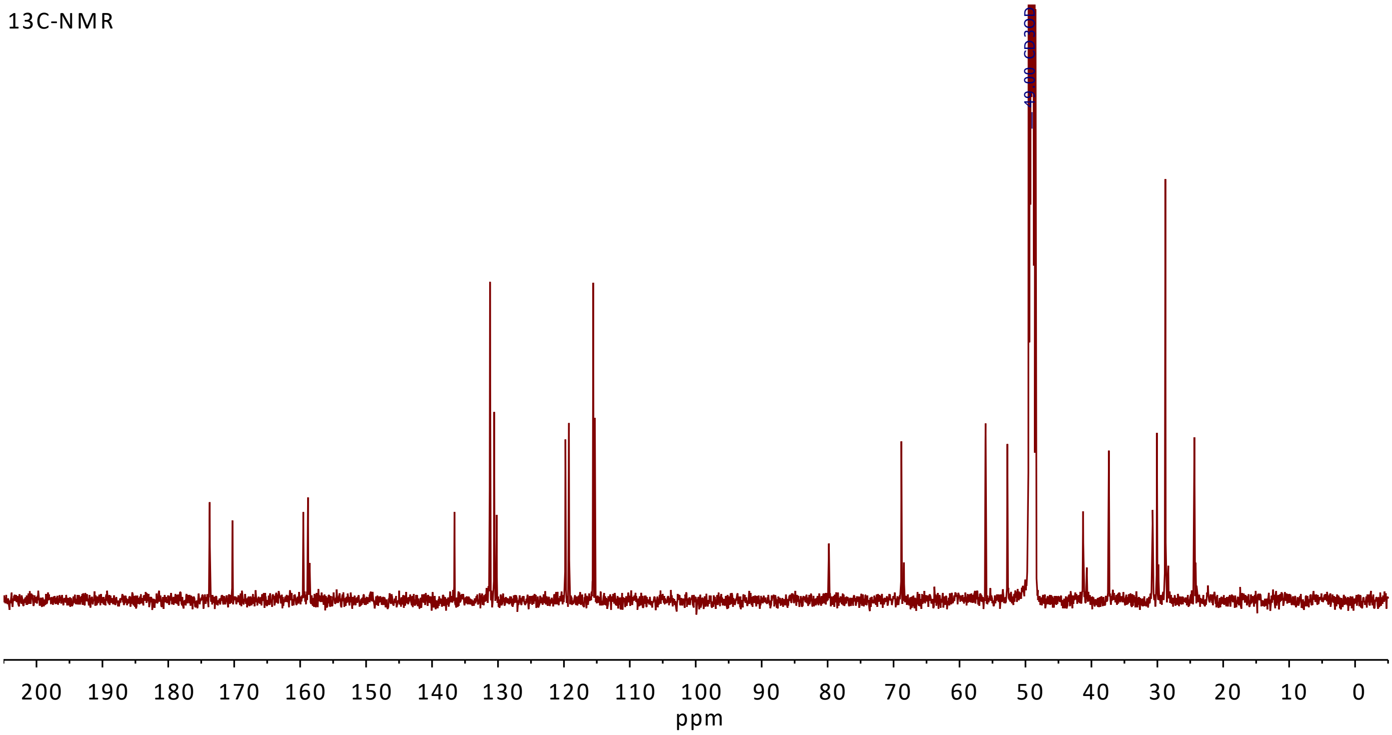

254 nm (LC-MS)

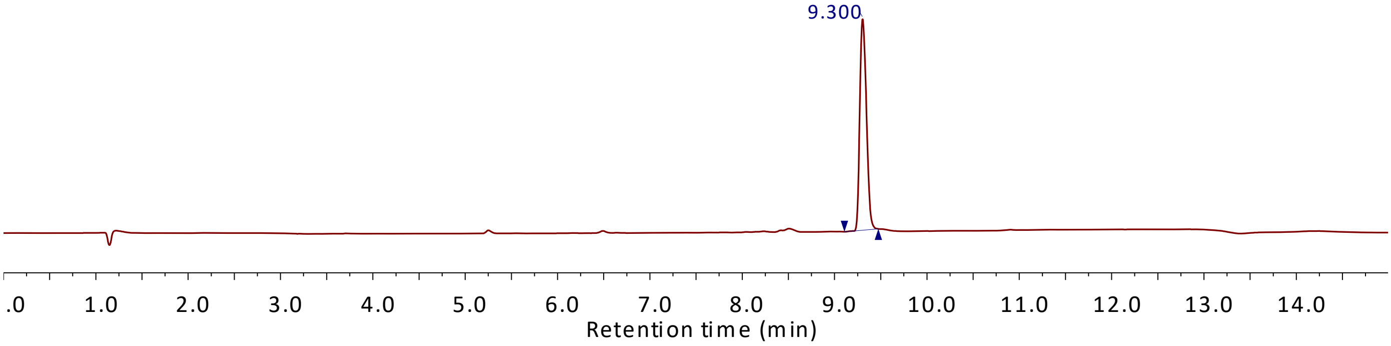

3c

1H-NMR

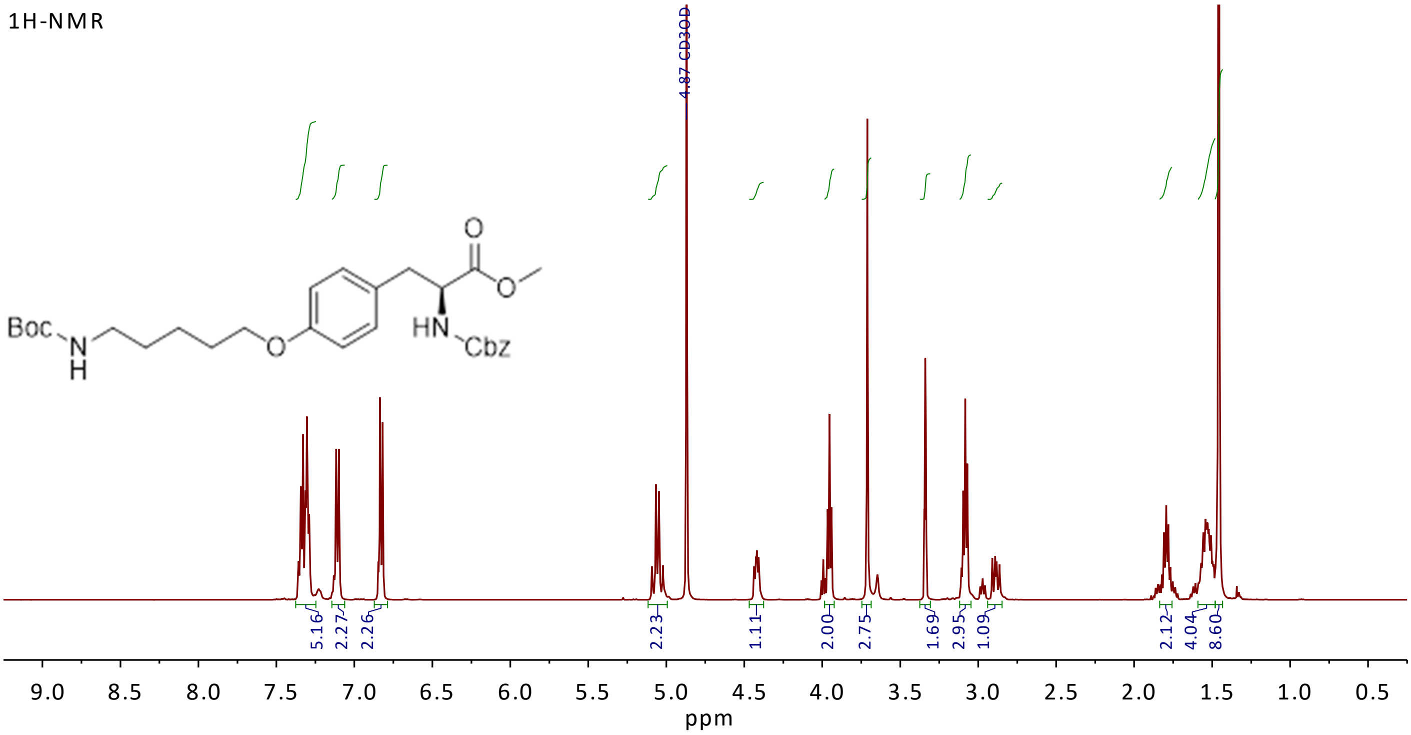

13C-NMR

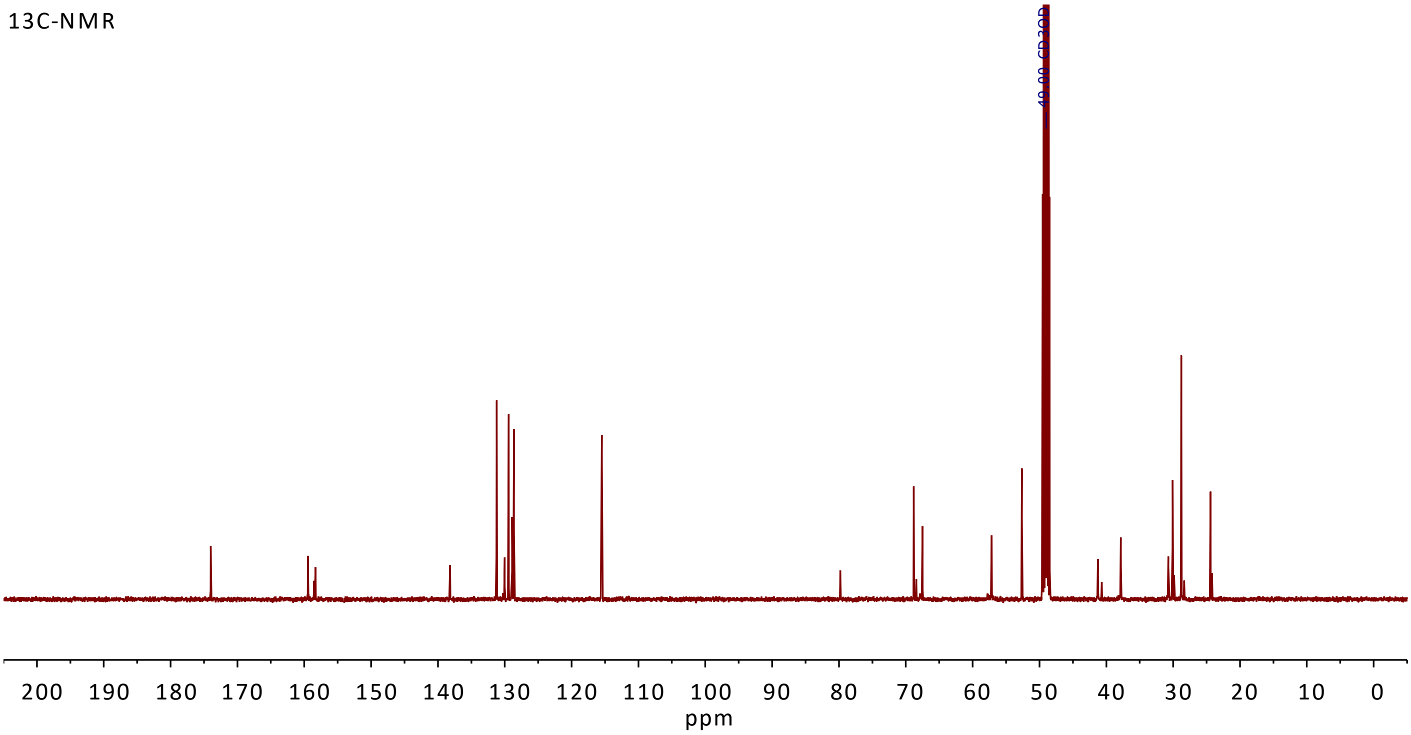

220 nm (LC-MS)

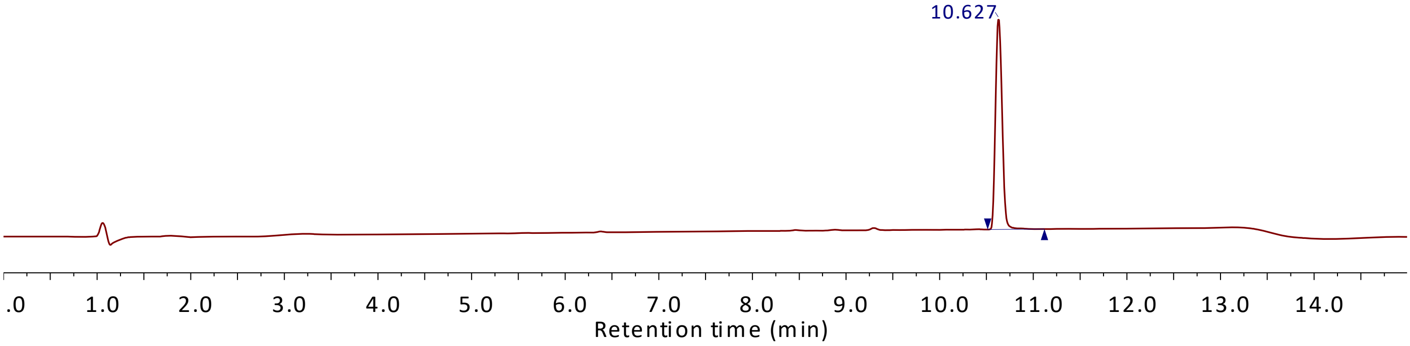

5aa

1H-NMR

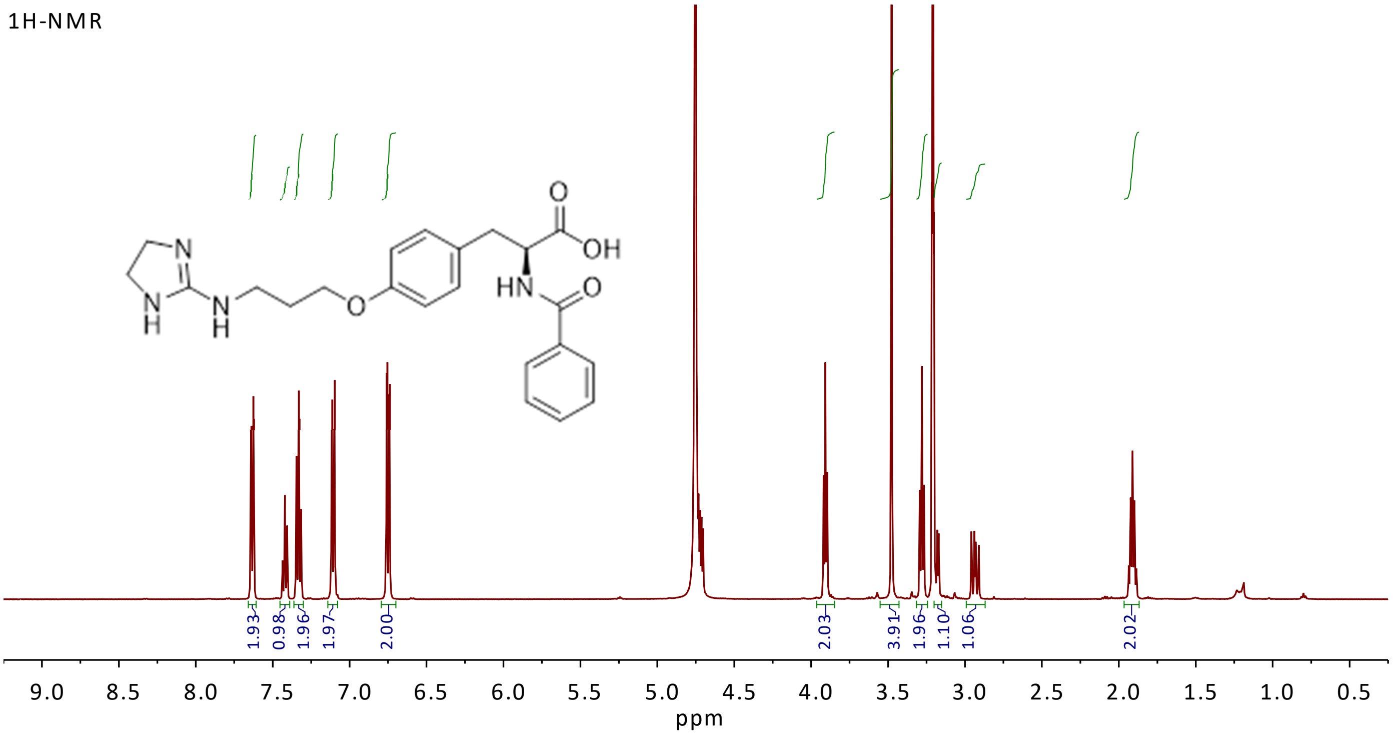

13C-NMR

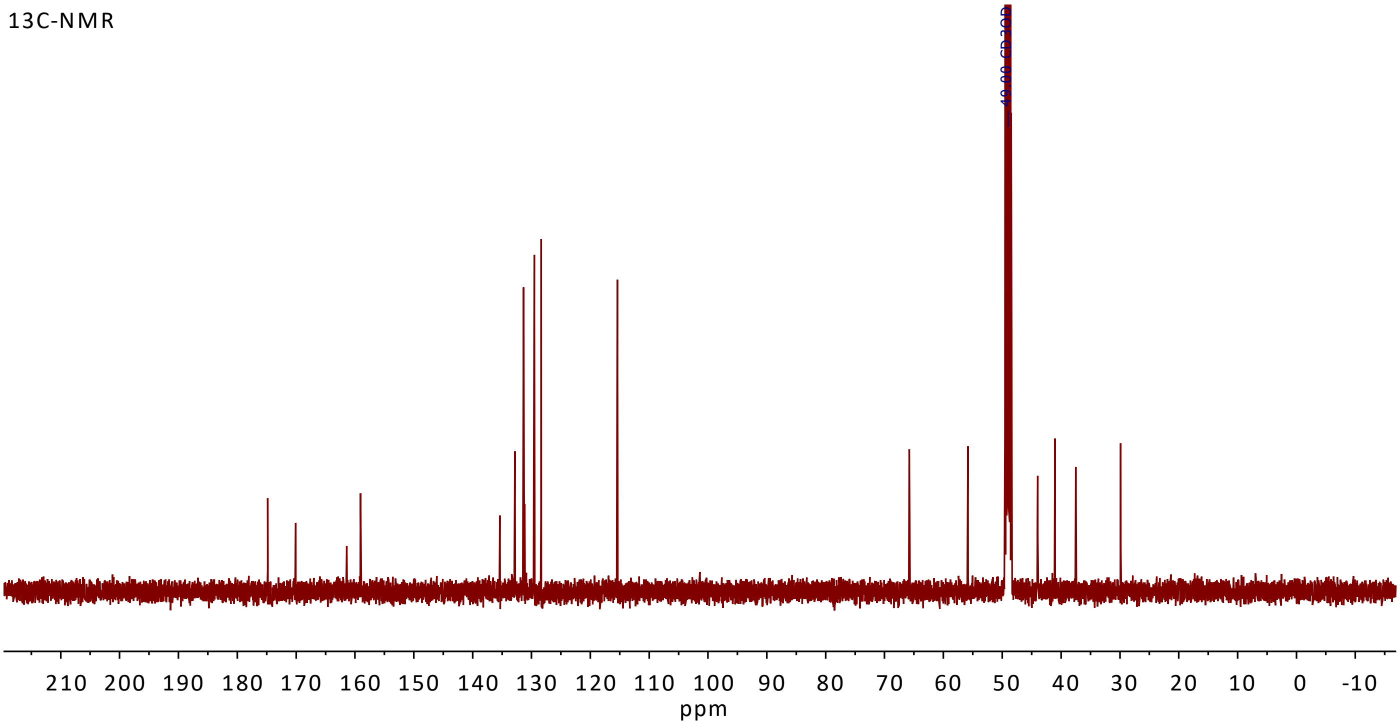

254 nm (LC-MS)

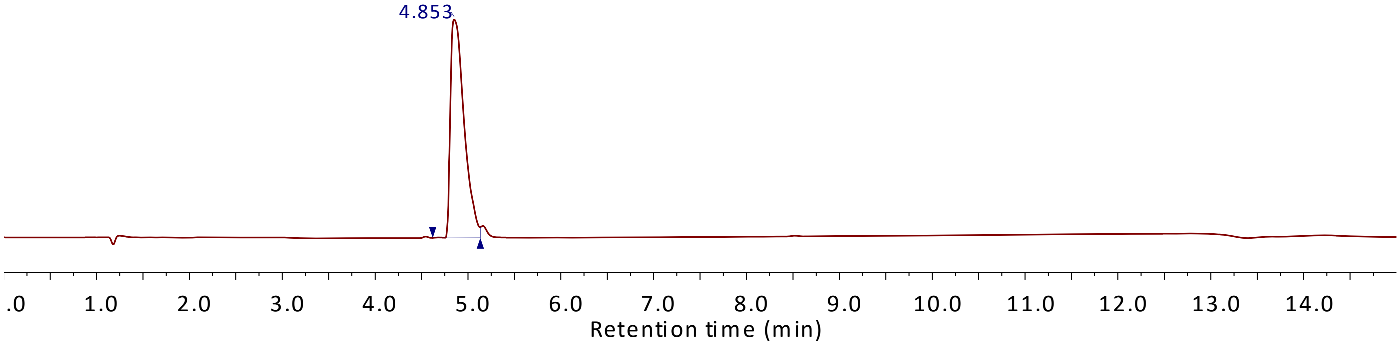

5ba

1H-NMR

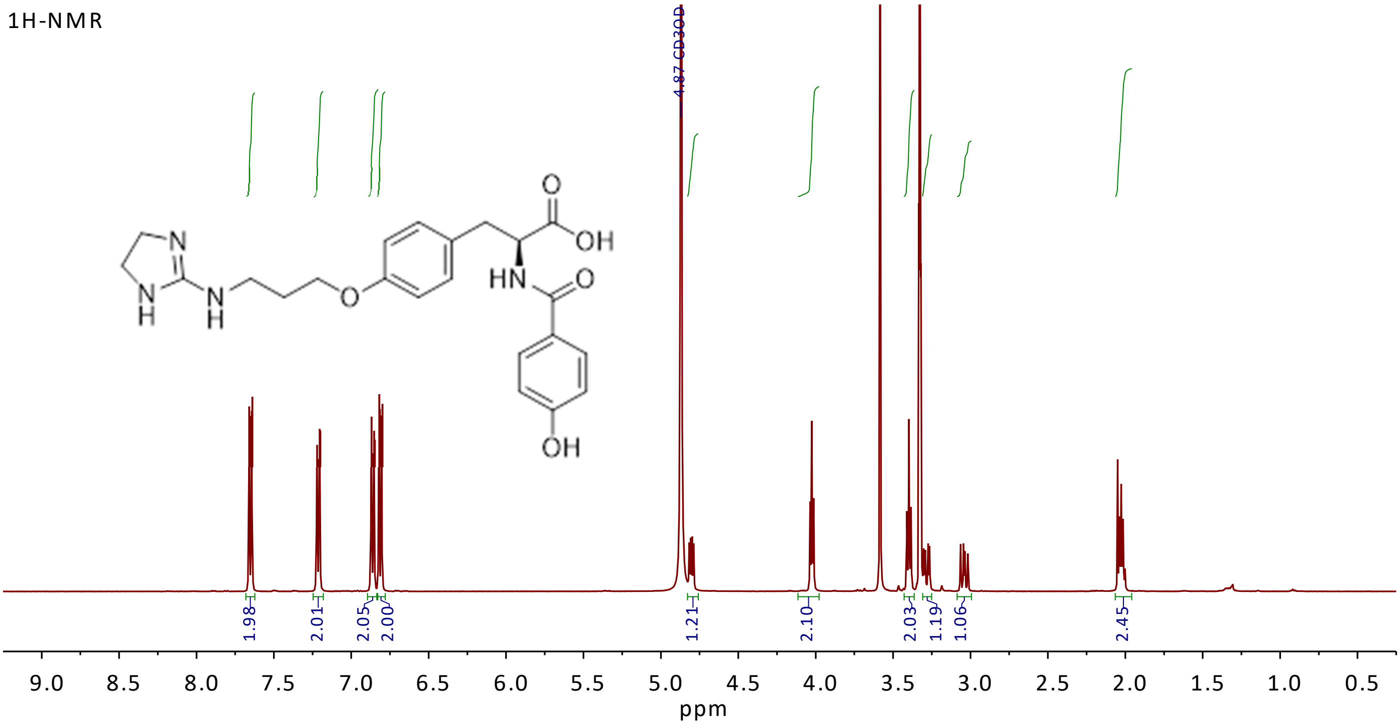

13C-NMR

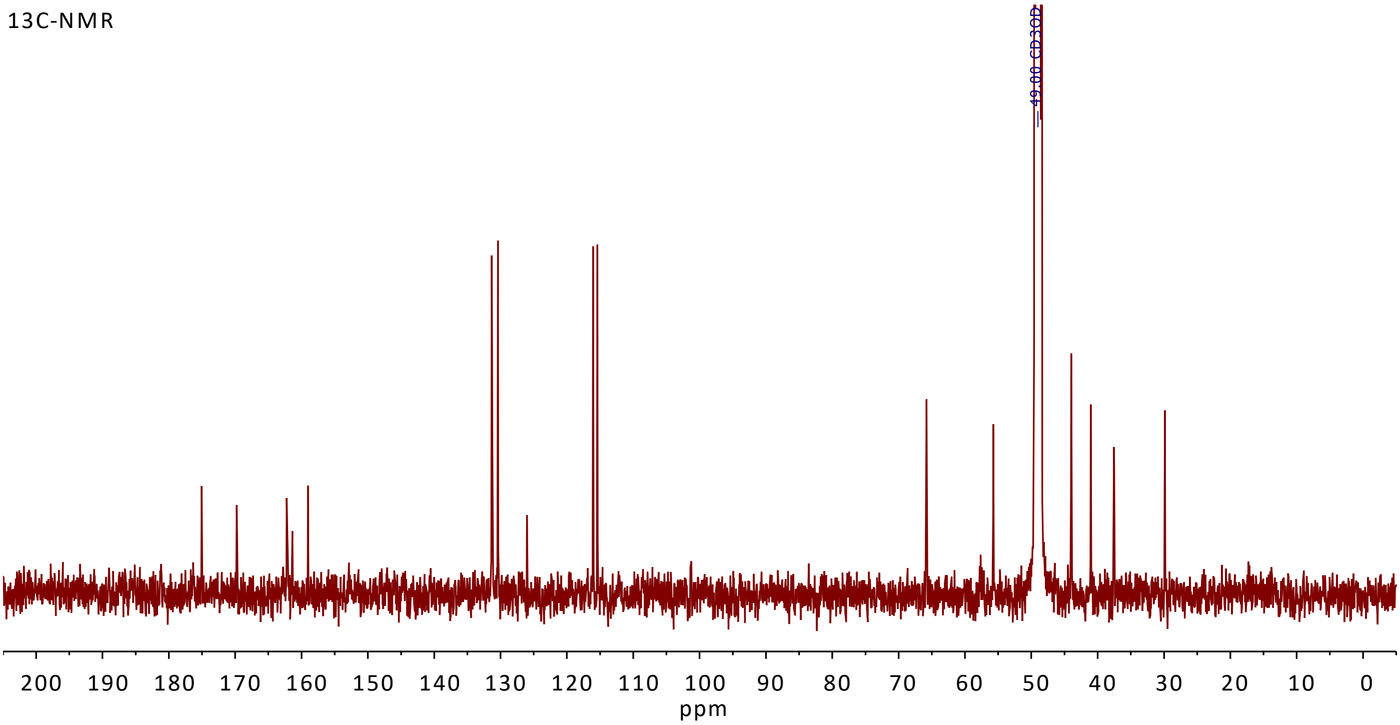

254 nm (LC-MS)

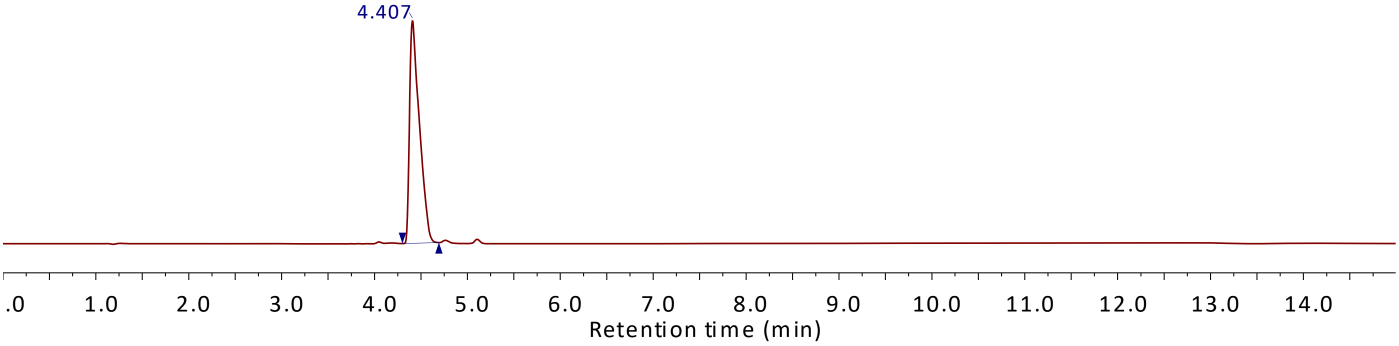

5ca

1H-NMR

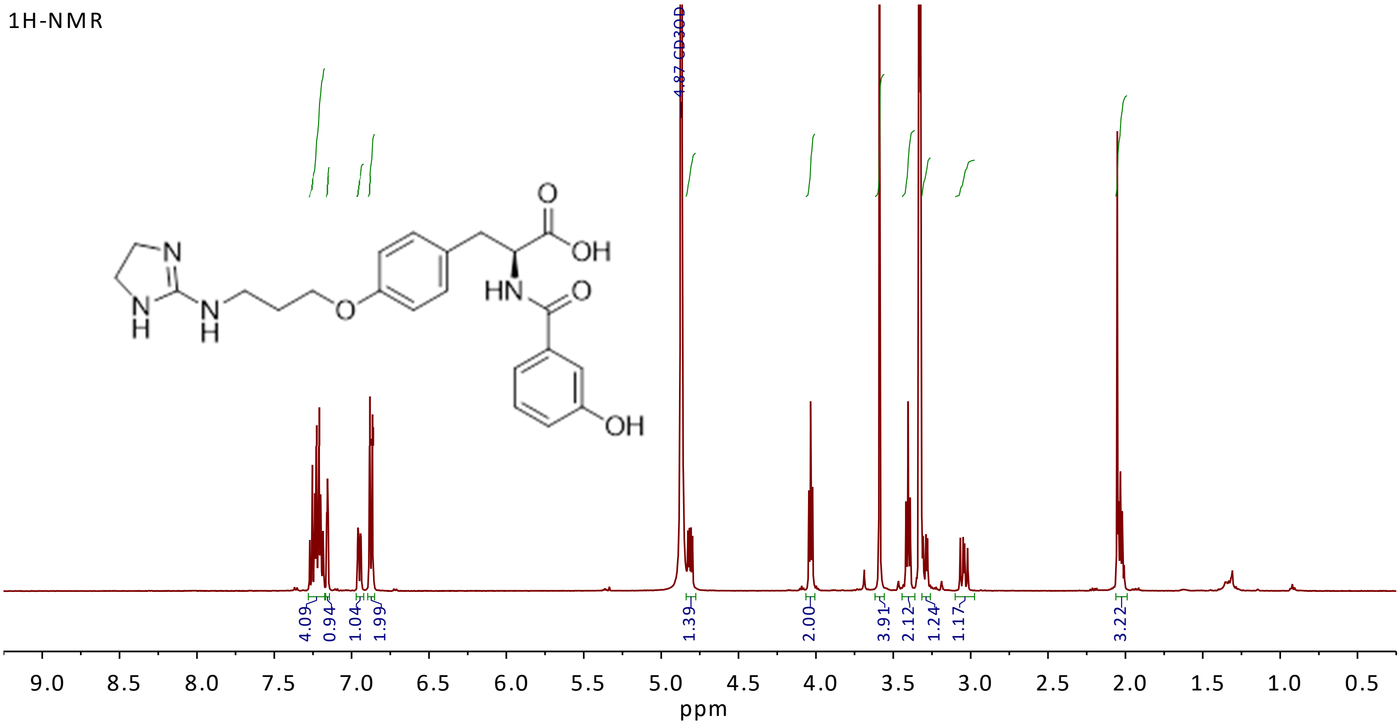

13C-NMR

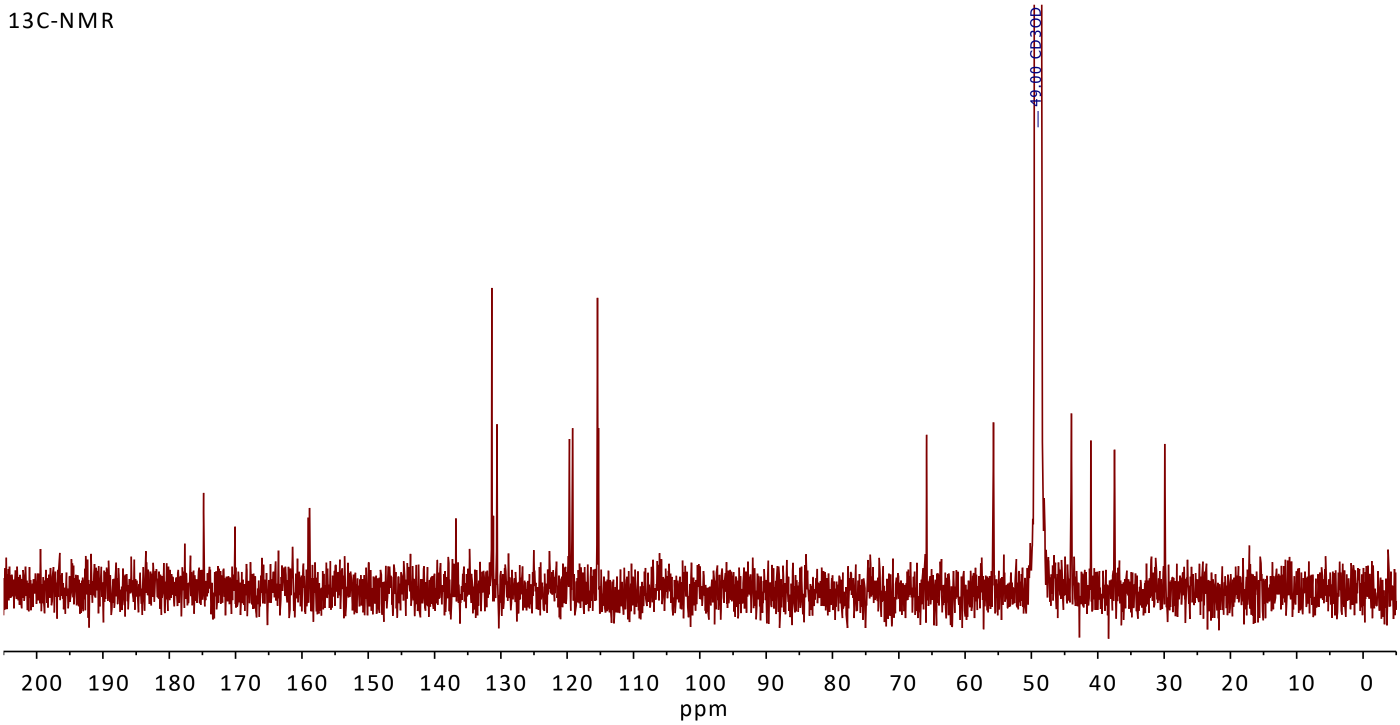

254 nm (LC-MS)

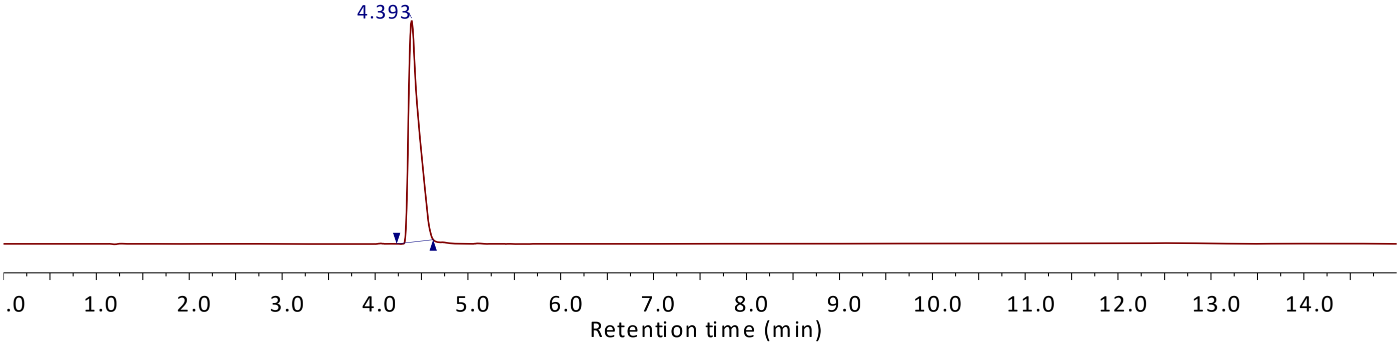

5da

1H-NMR

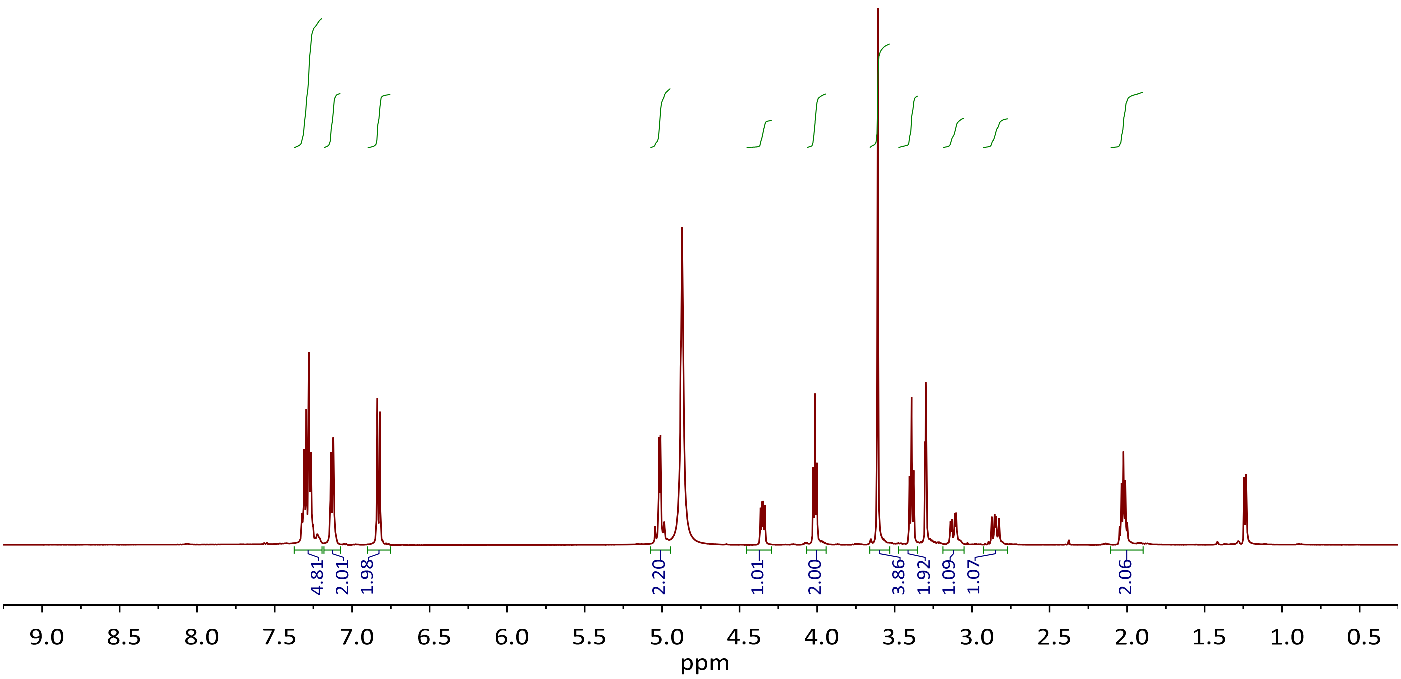

13C-NMR

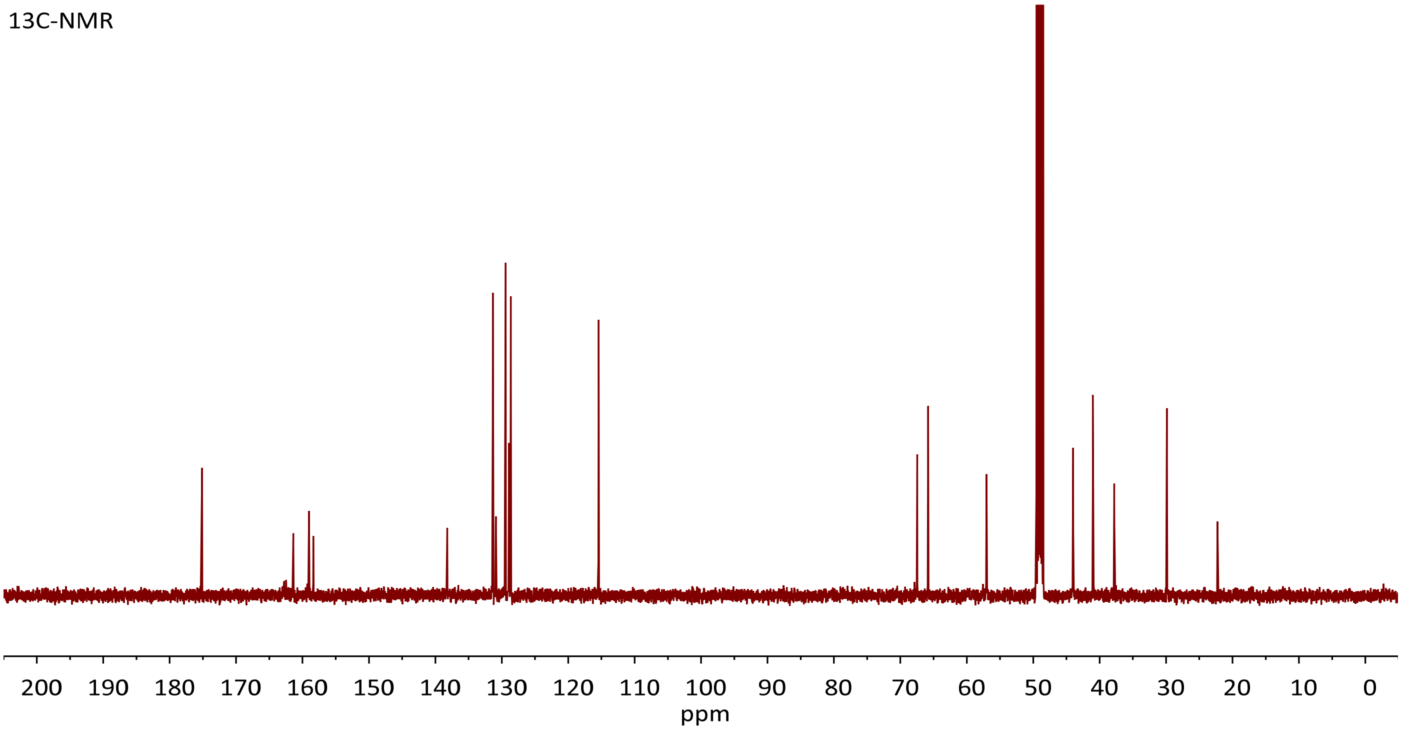

254 nm (LC-MS)

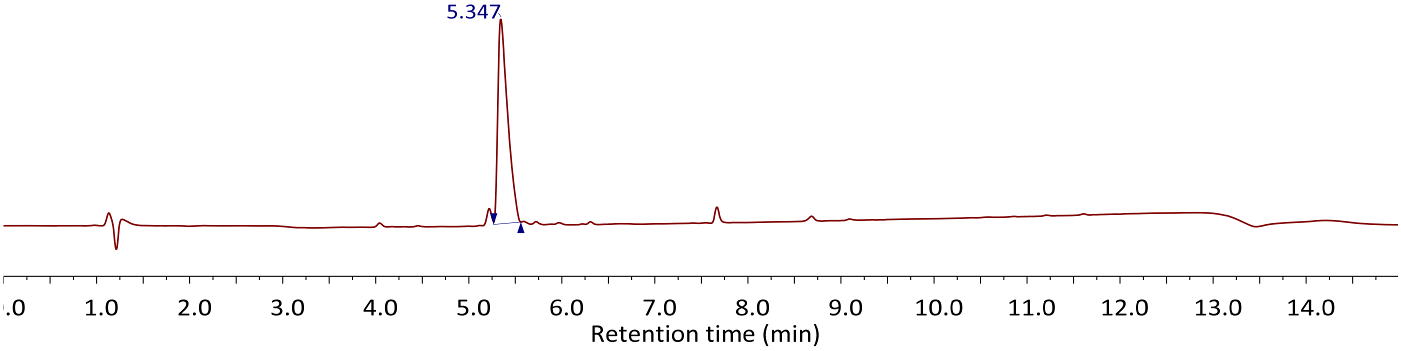

6aa

1H-NMR

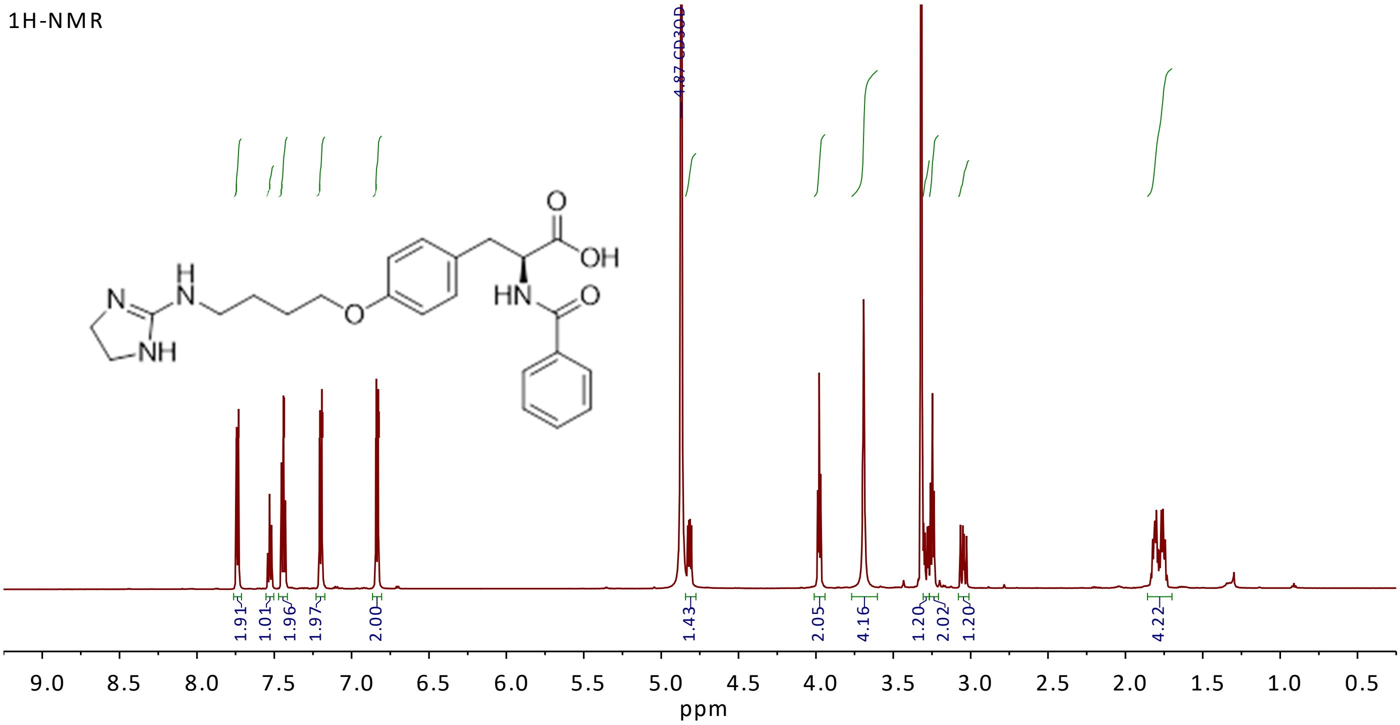

13C-NMR

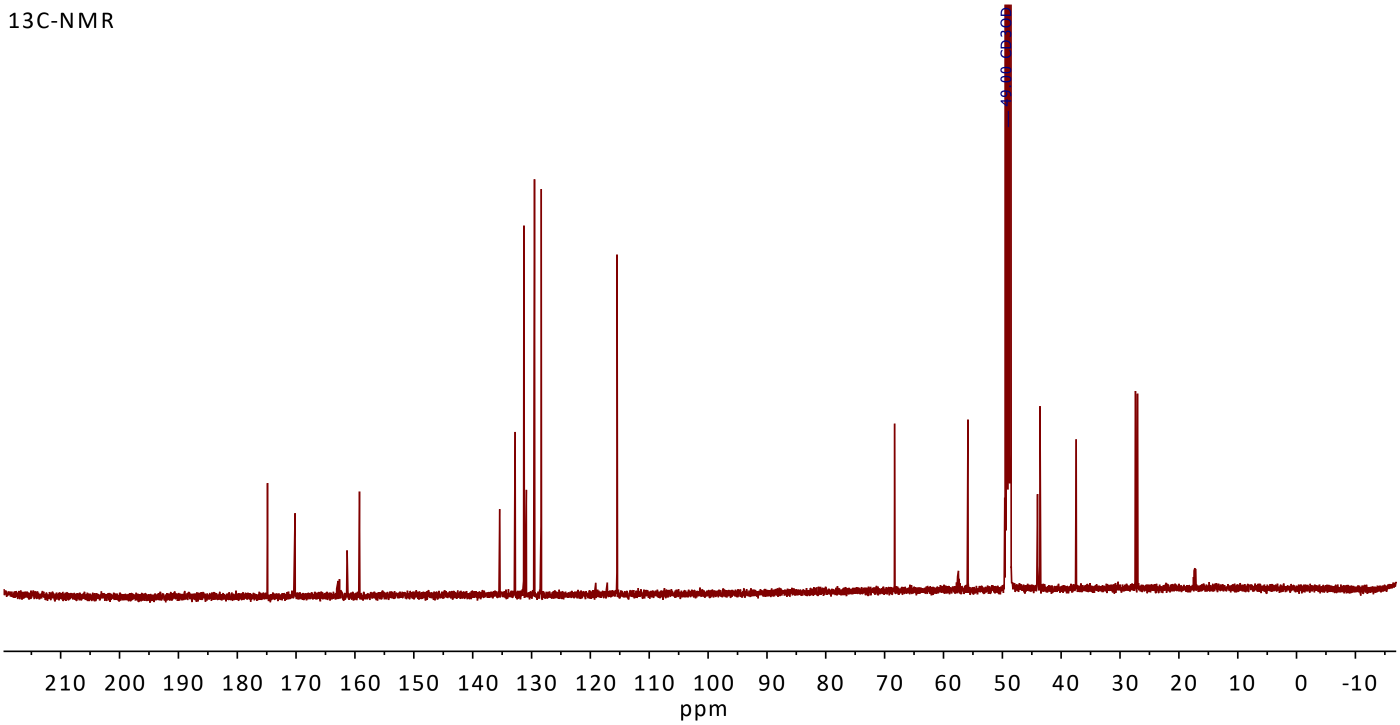

6ba

1H-NMR

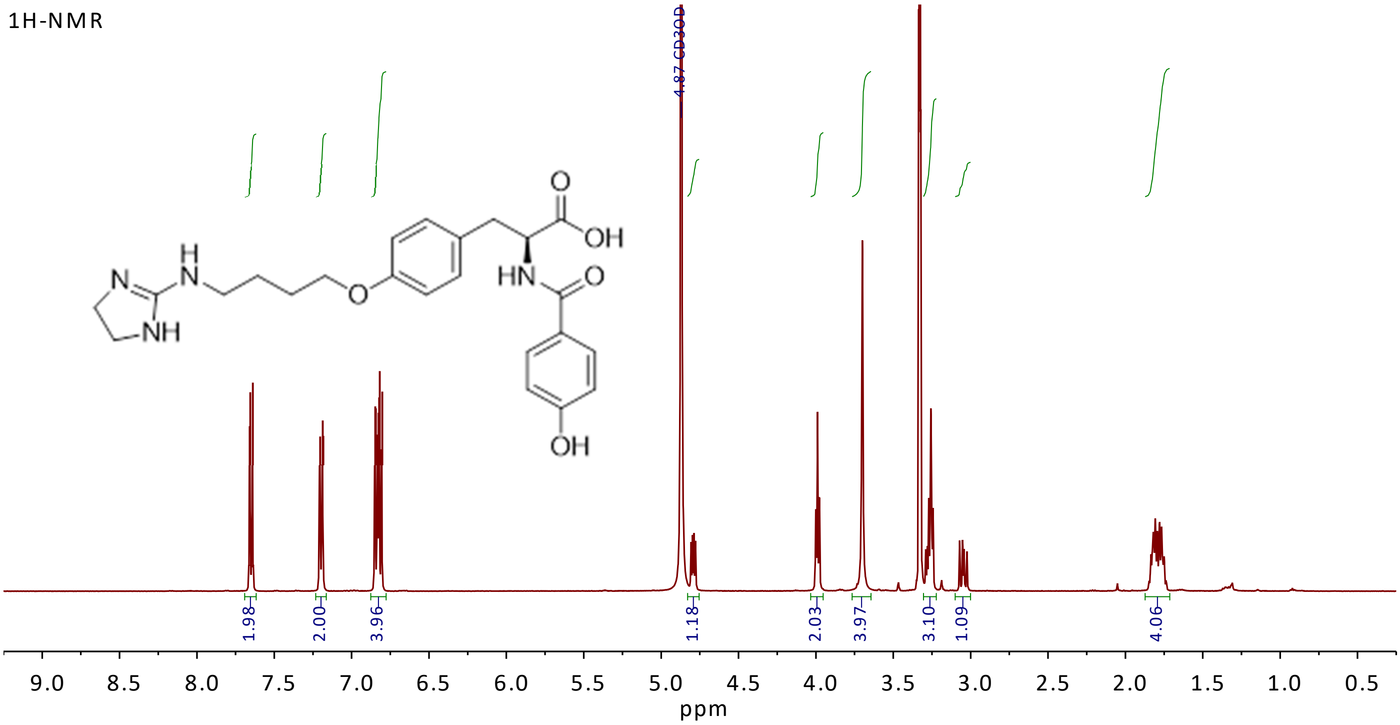

13C-NMR

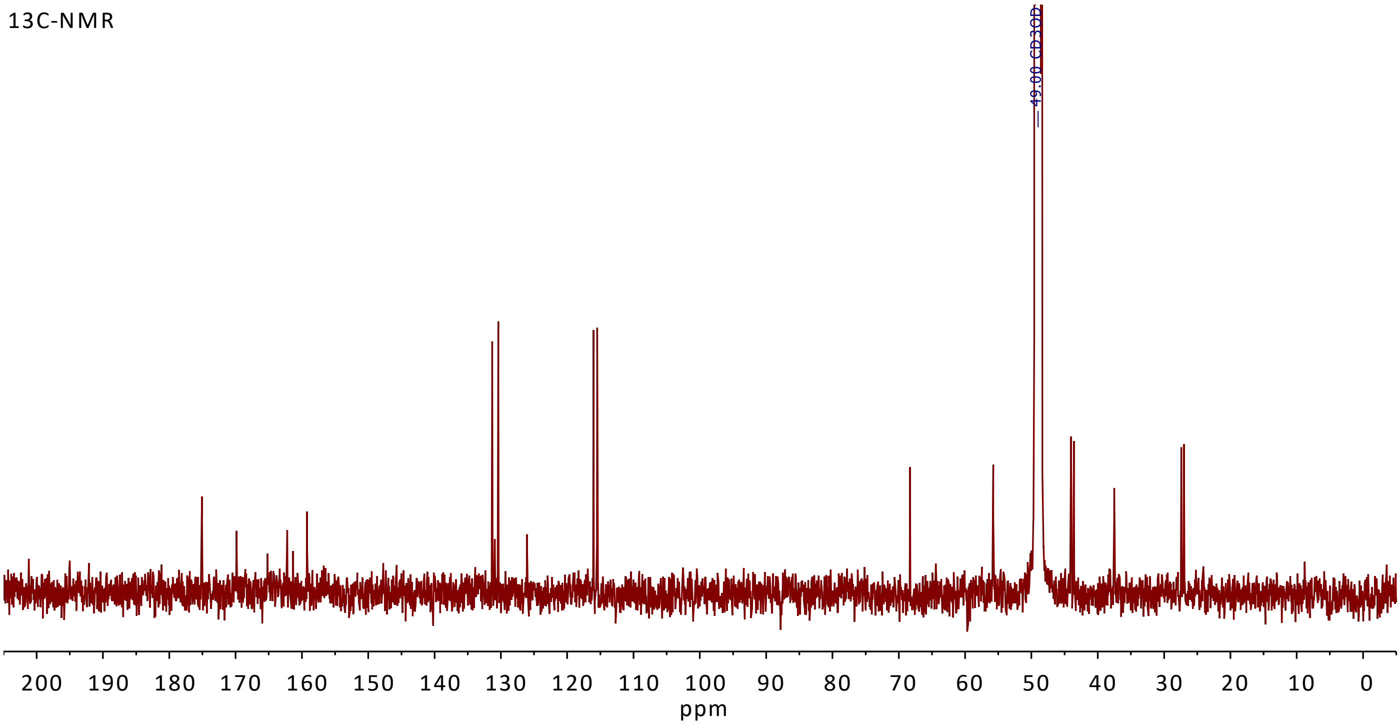

254 nm (LC-MS)

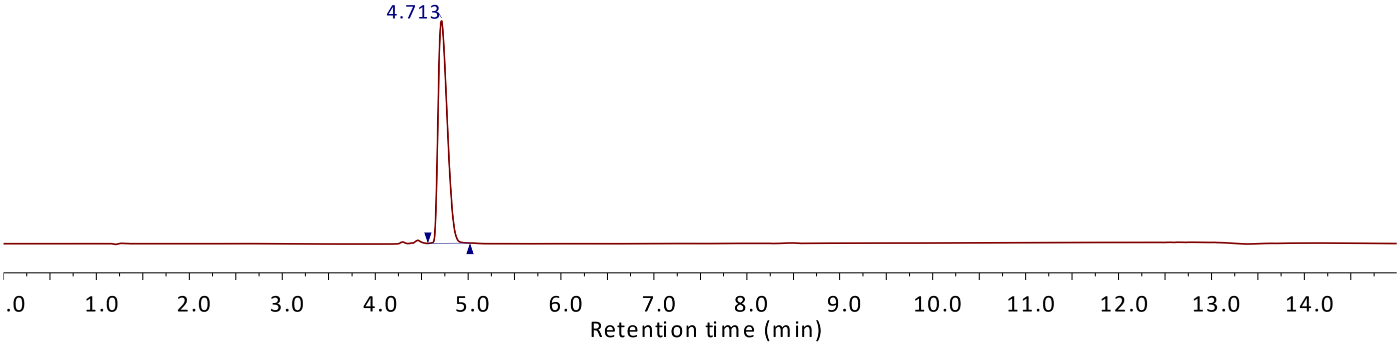

6ca

1H-NMR

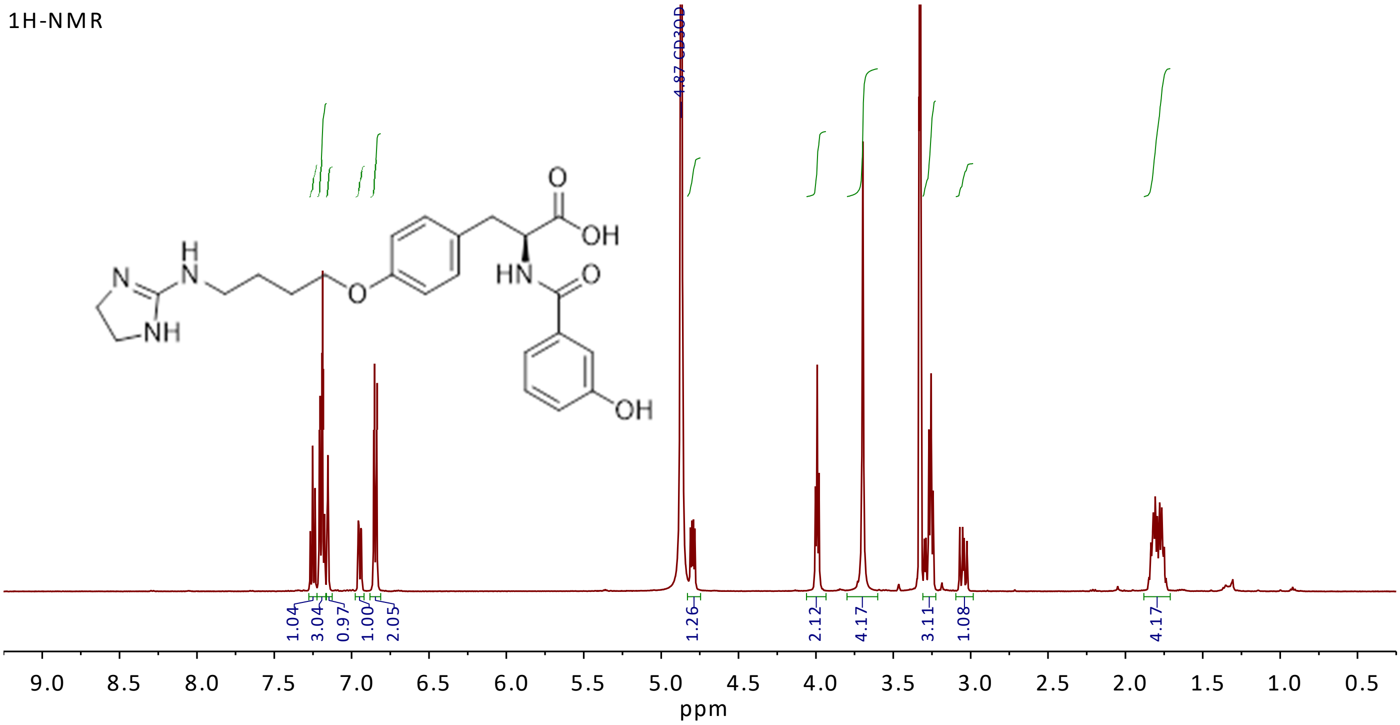

13C-NMR

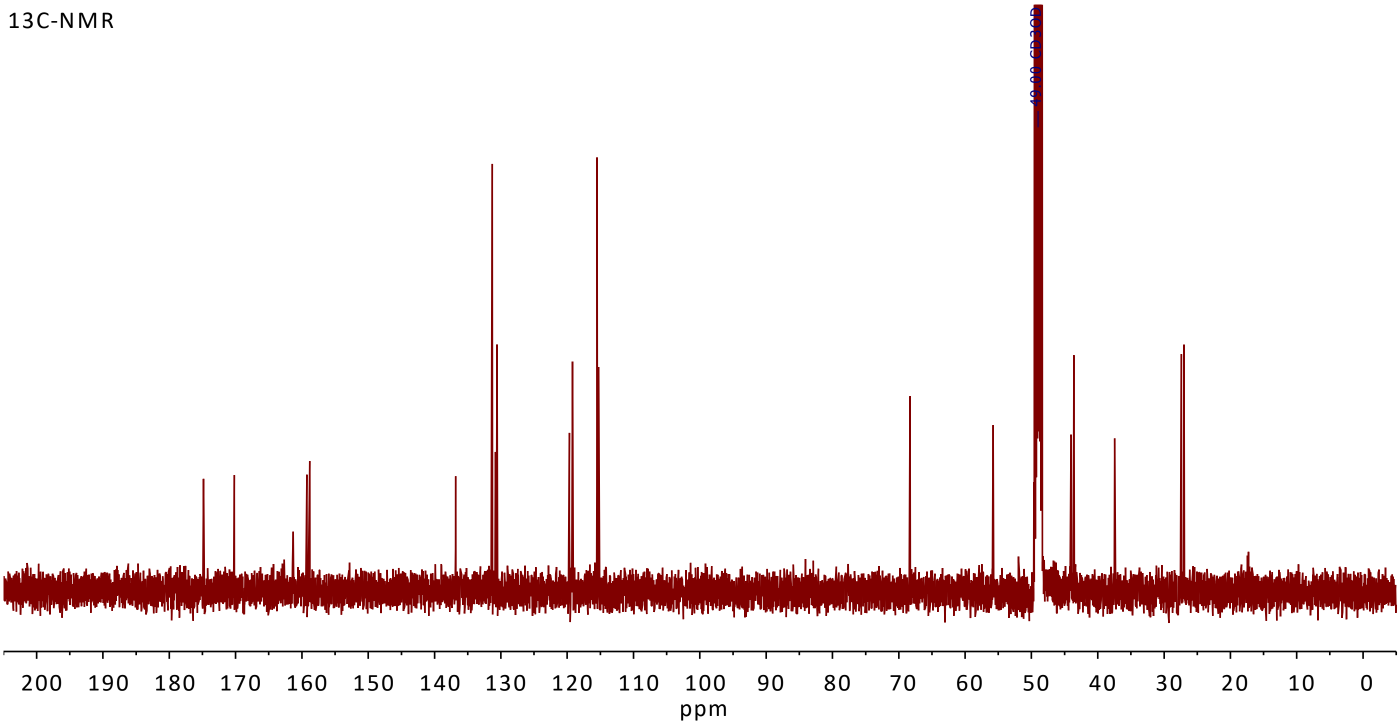

254 nm (LC-MS)

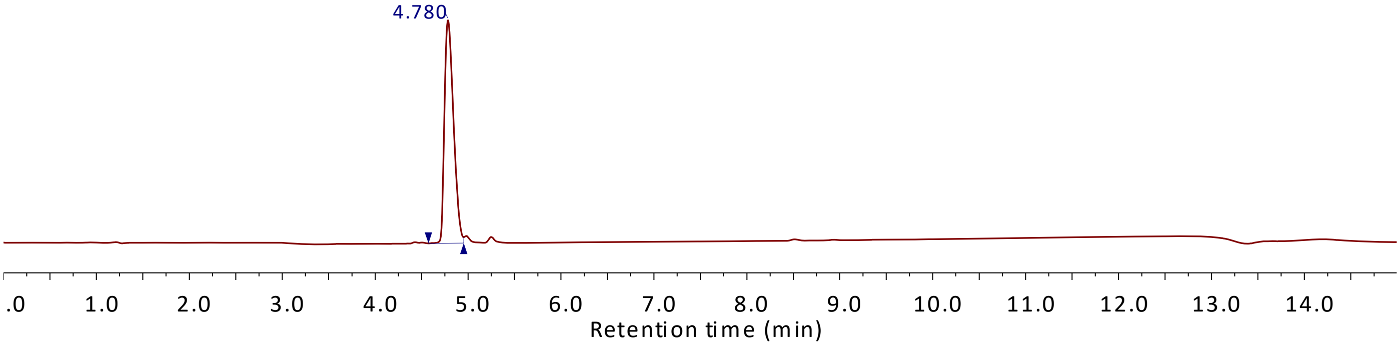

6da

1H-NMR

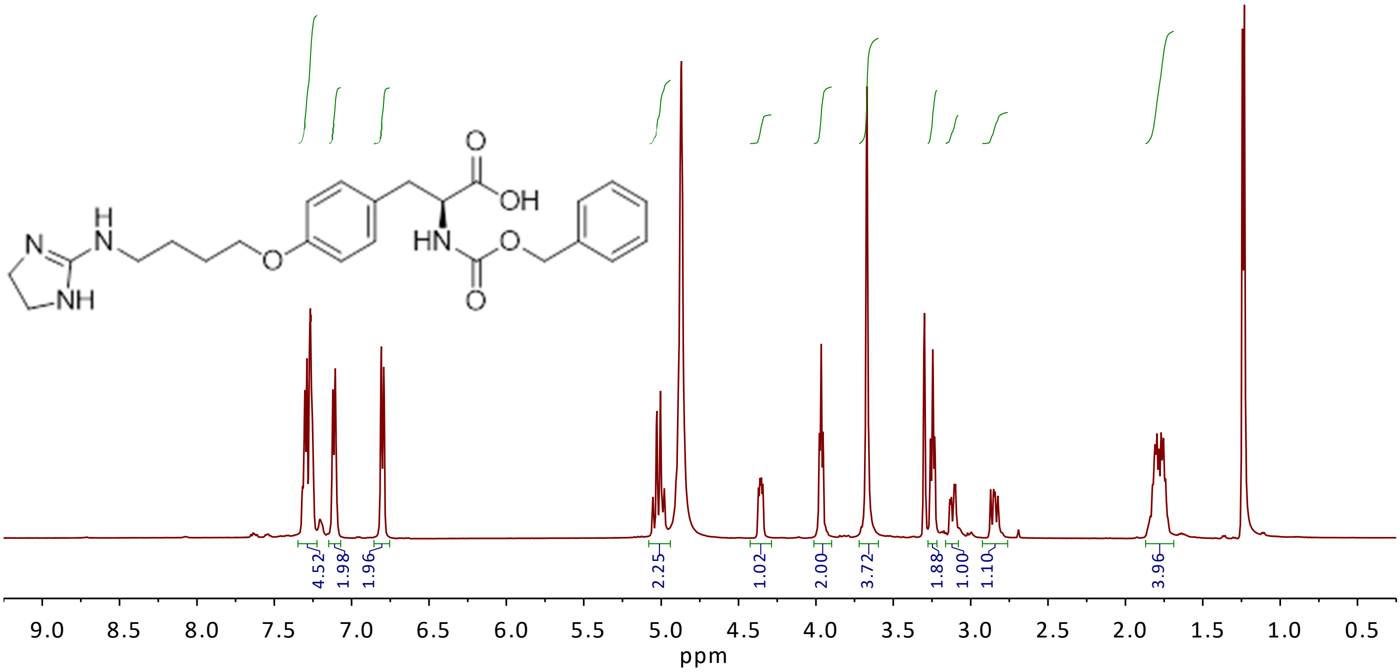

13C-NMR

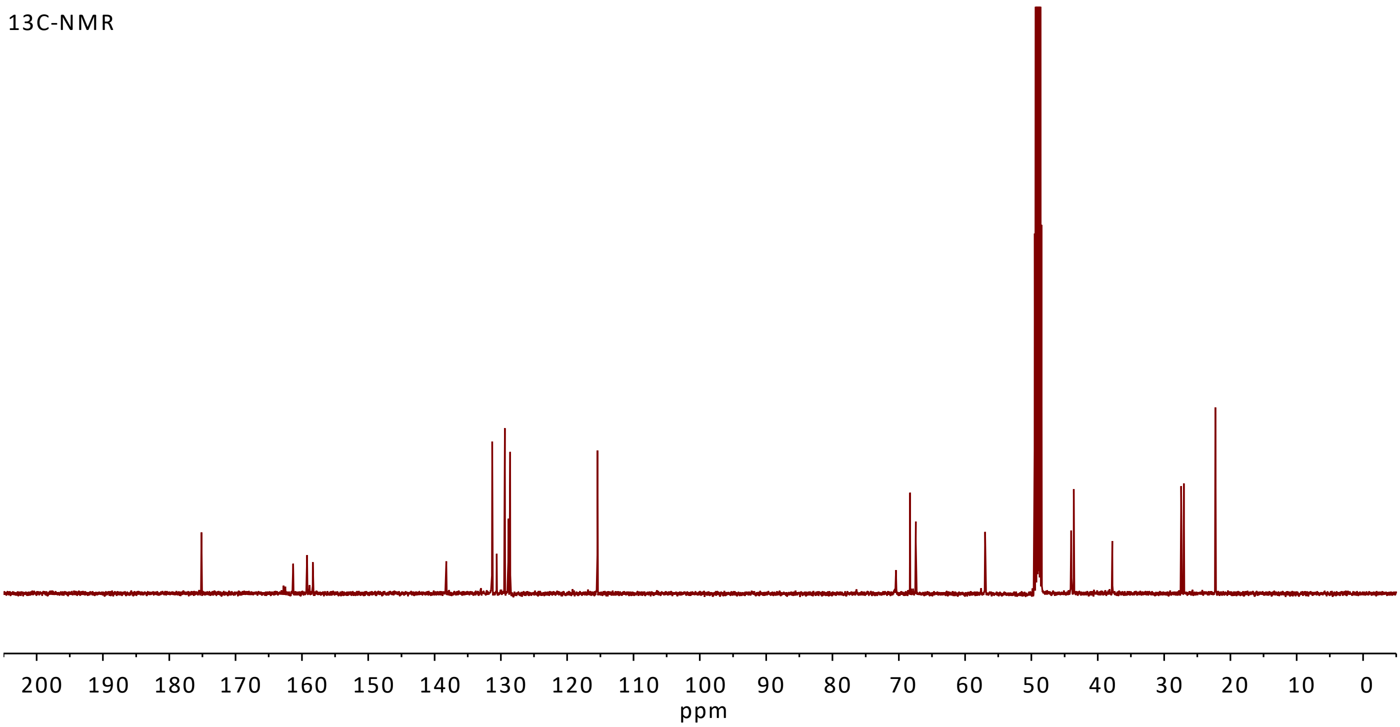

220 nm (LC-MS)

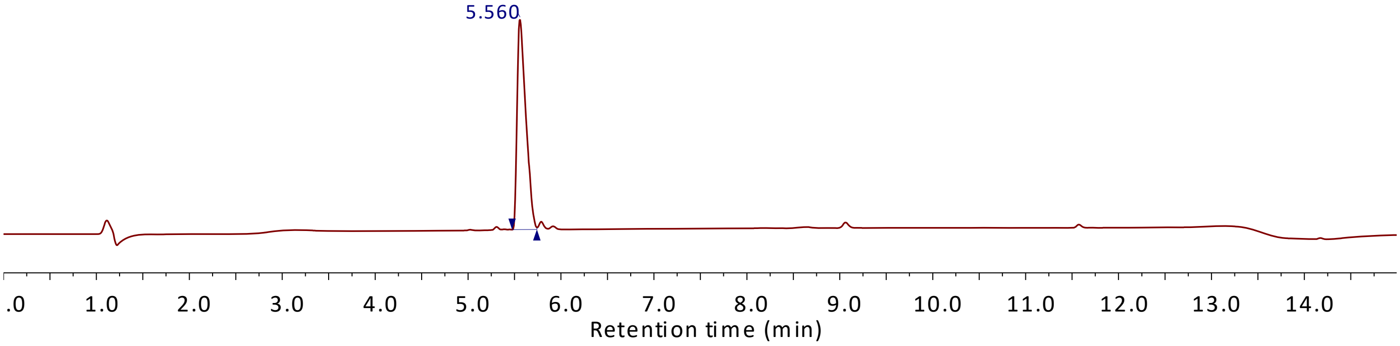

7aa

1H-NMR

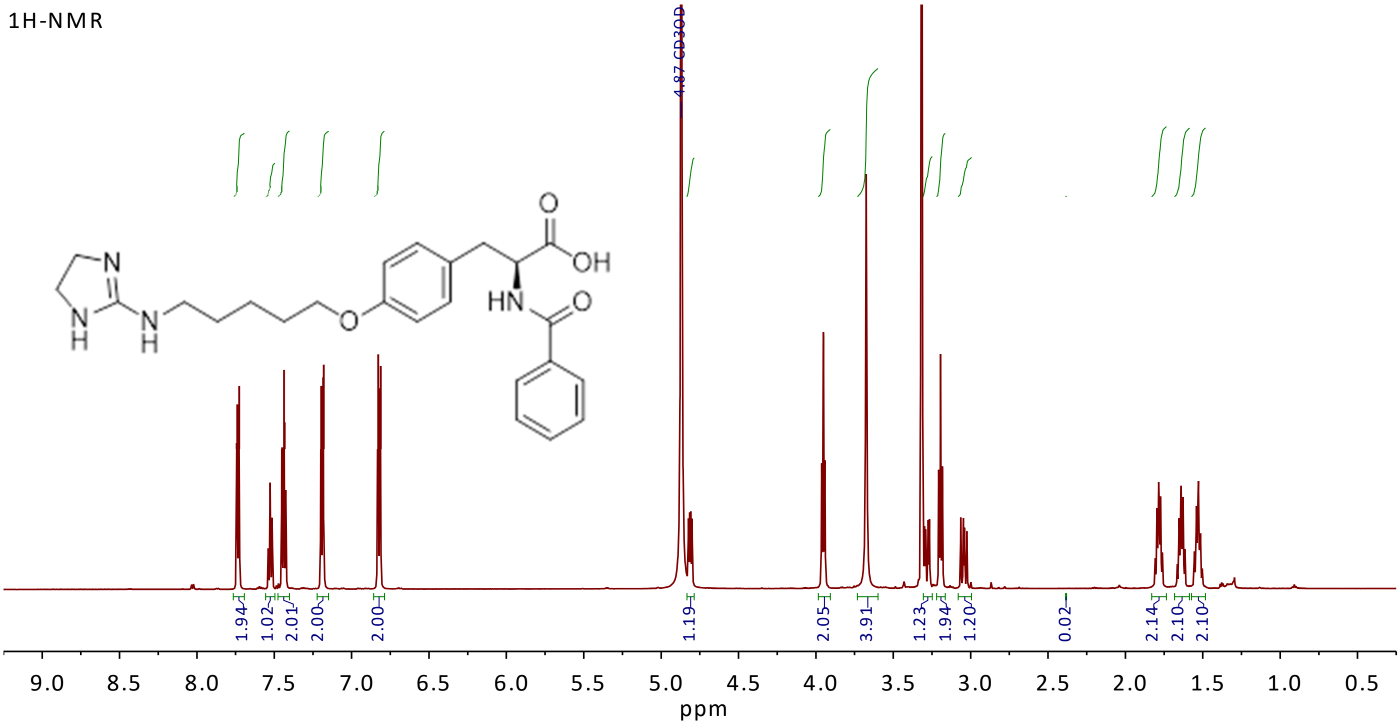

13C-NMR

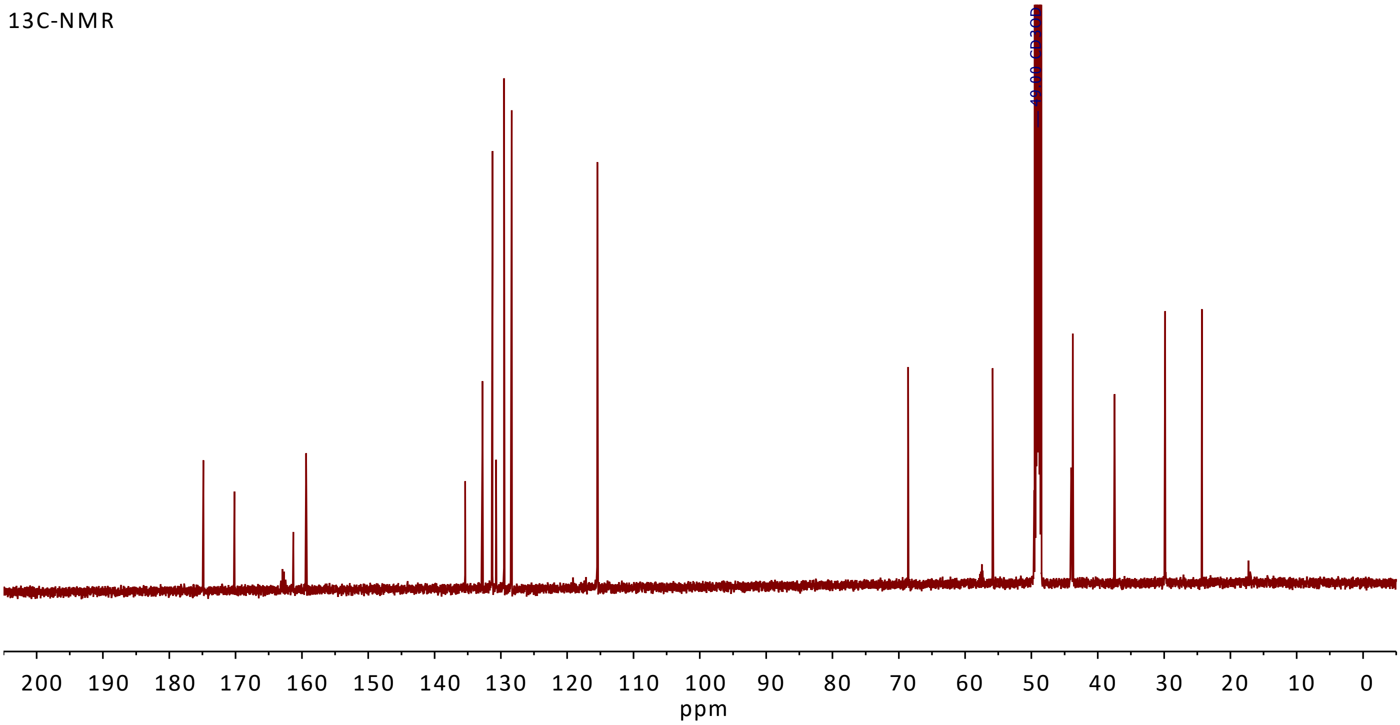

254 nm (LC-MS)

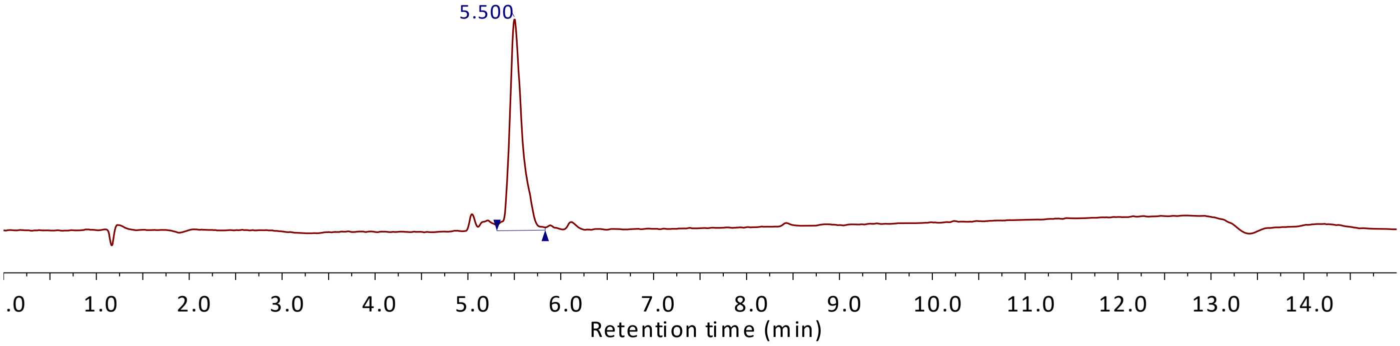

7ba

1H-NMR

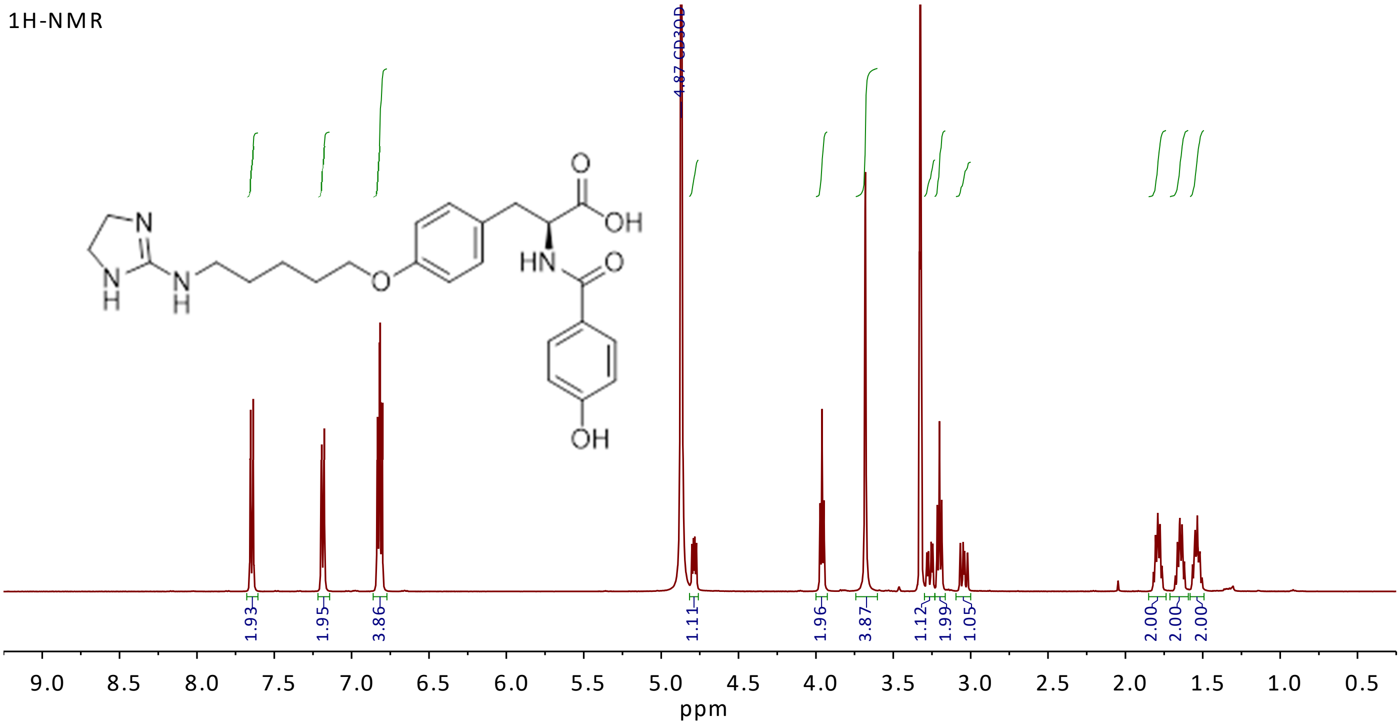

13C-NMR

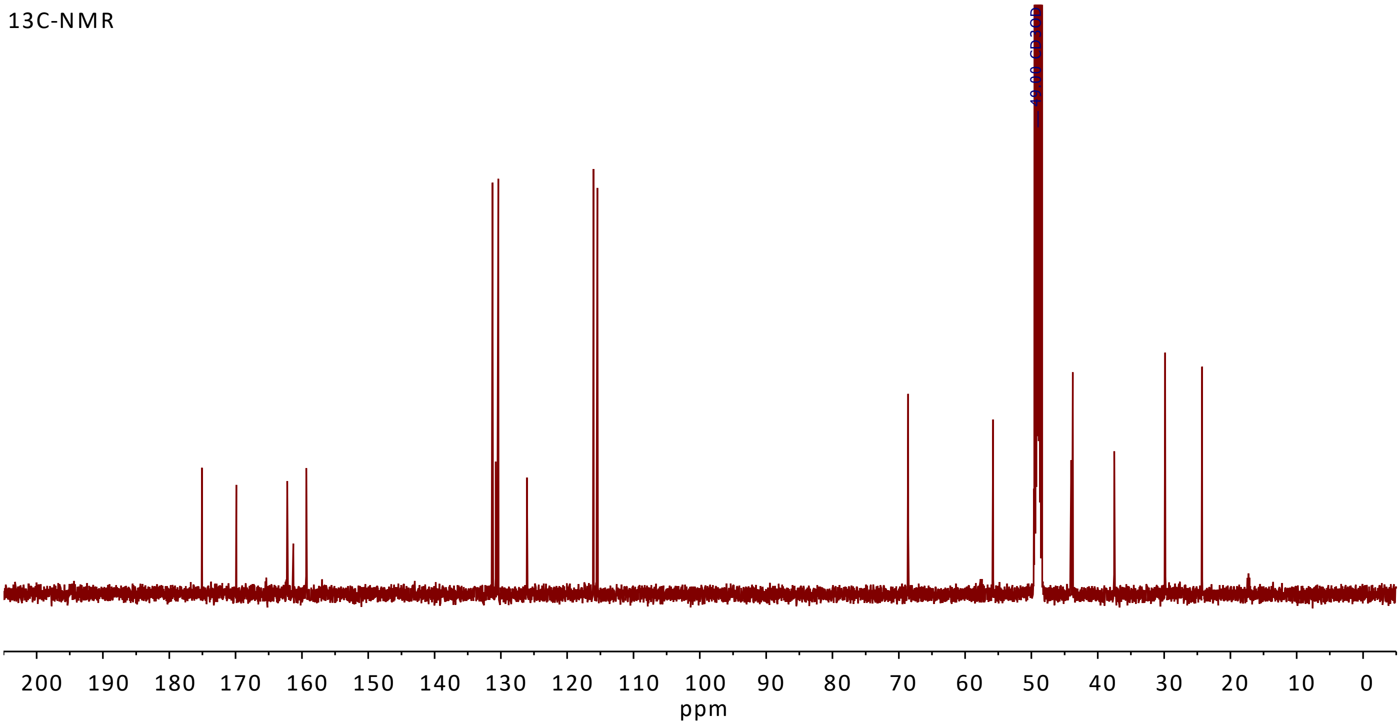

254 nm (LC-MS)

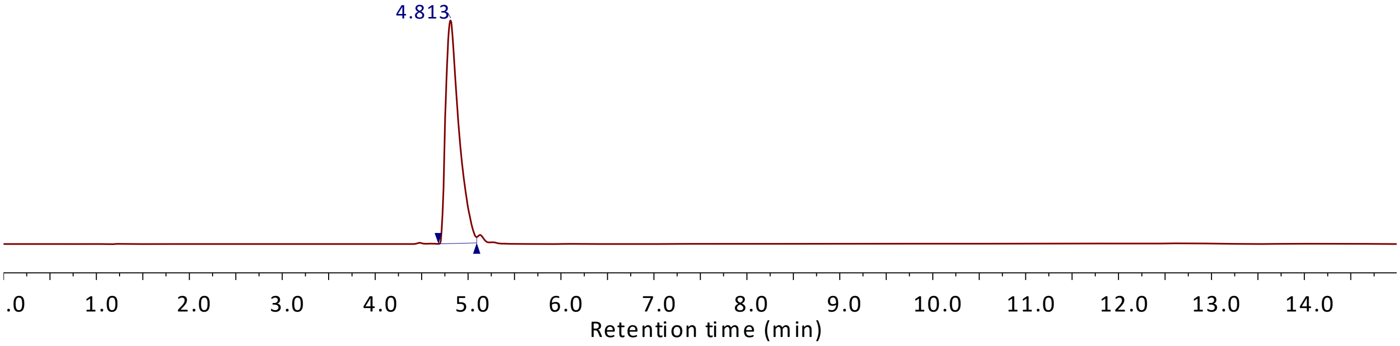

7ca

1H-NMR

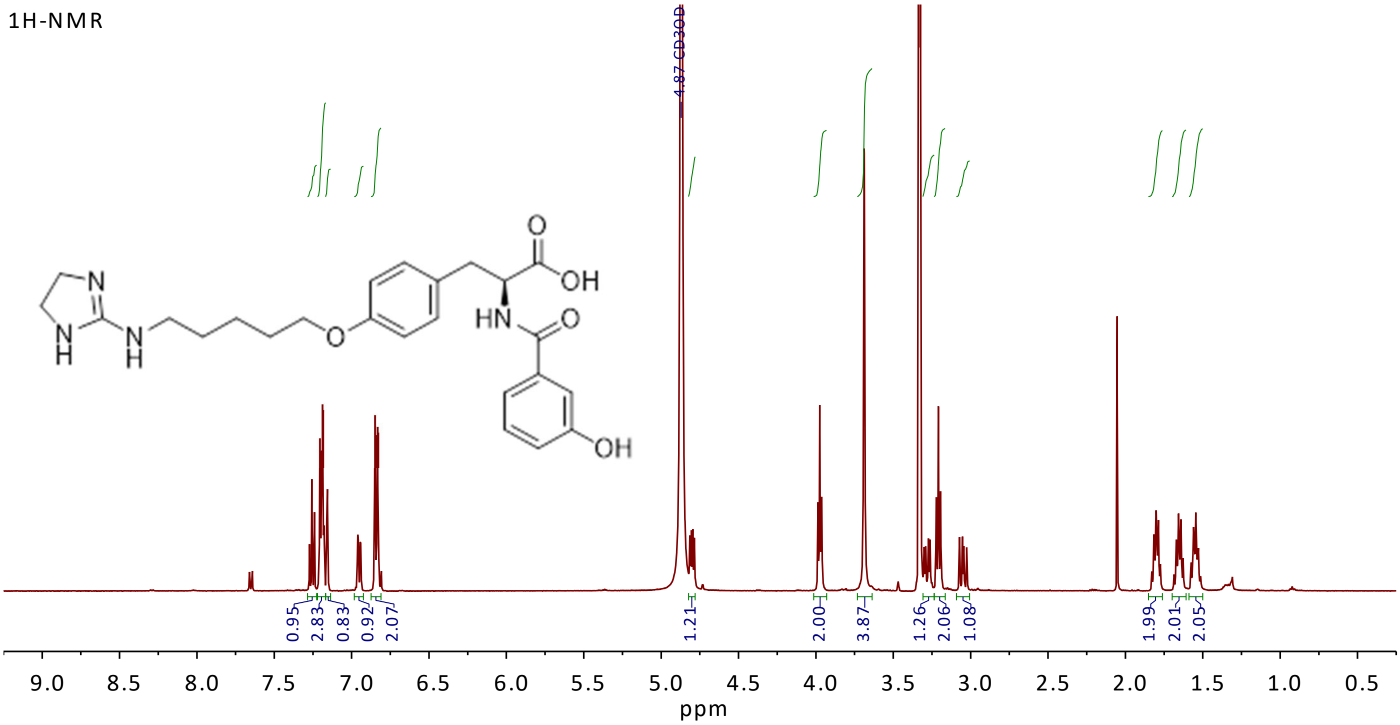

13C-NMR

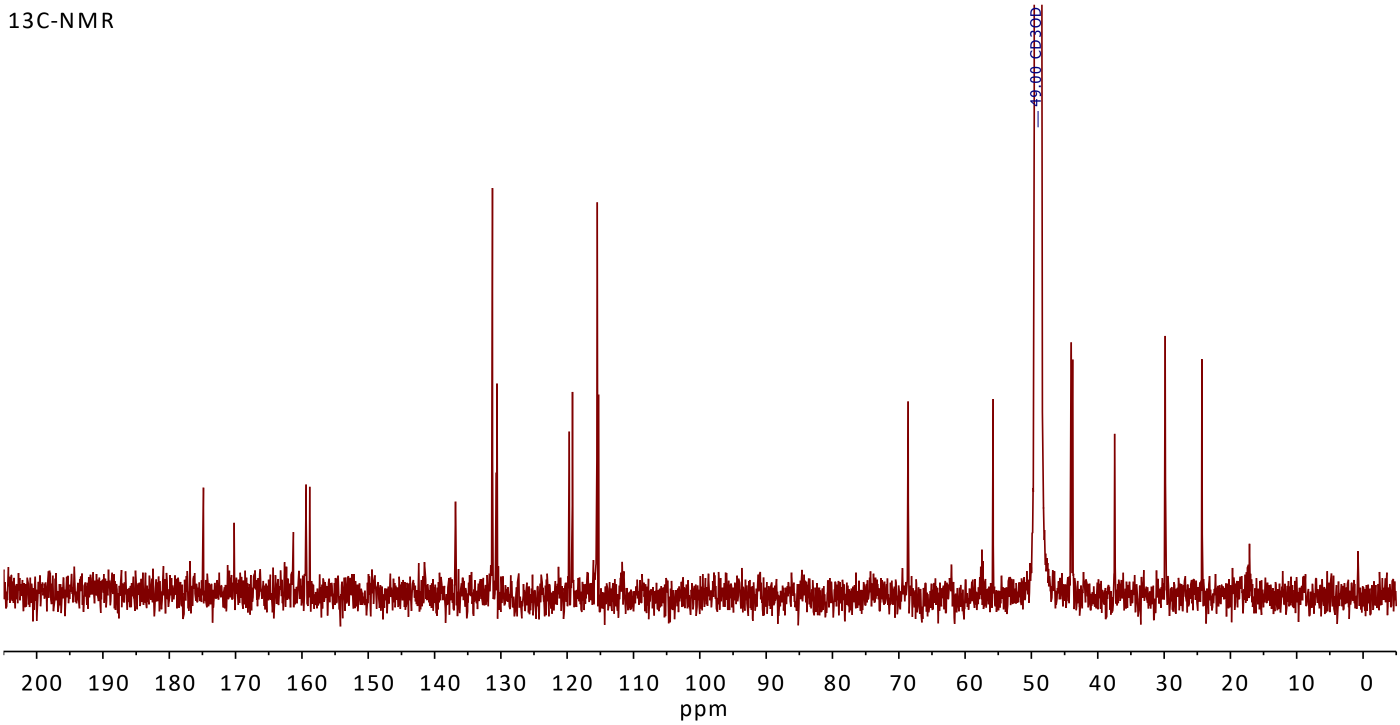

254 nm (LC-MS)

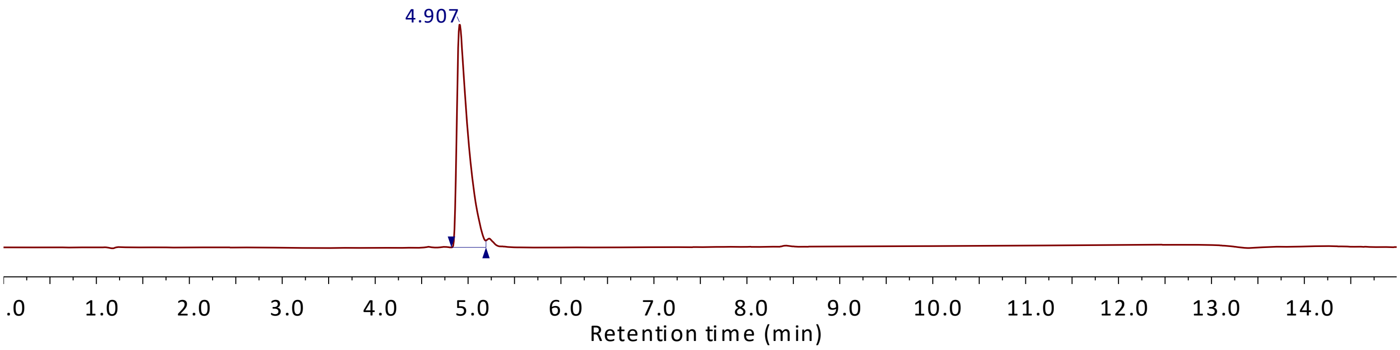

7da

1H-NMR

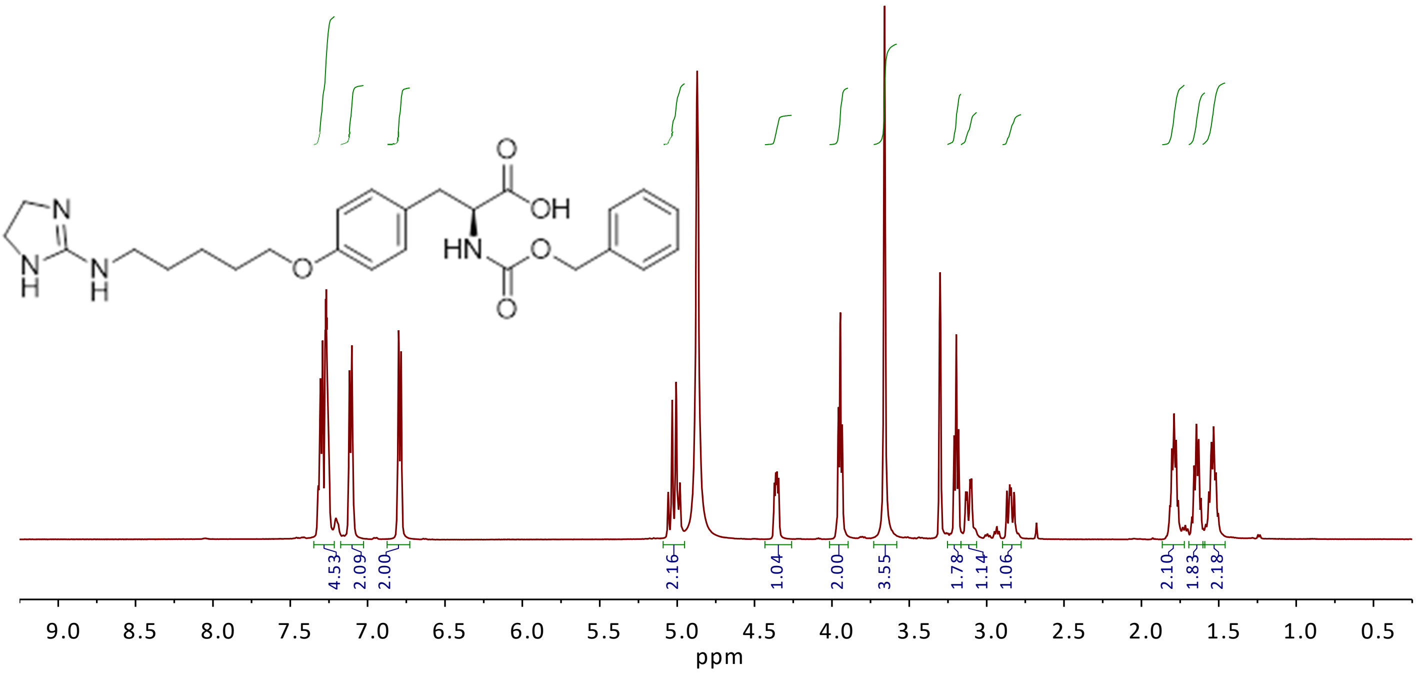

13C-NMR

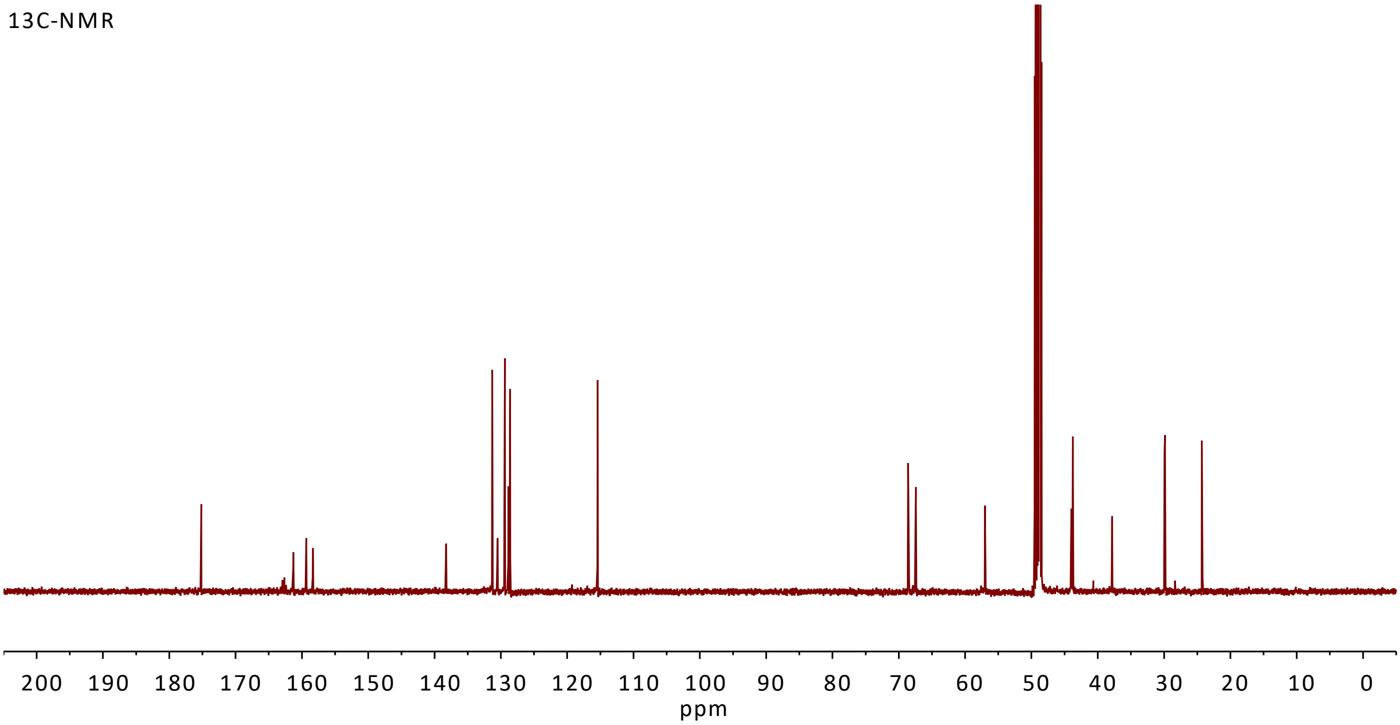

220 nm (LC-MS)

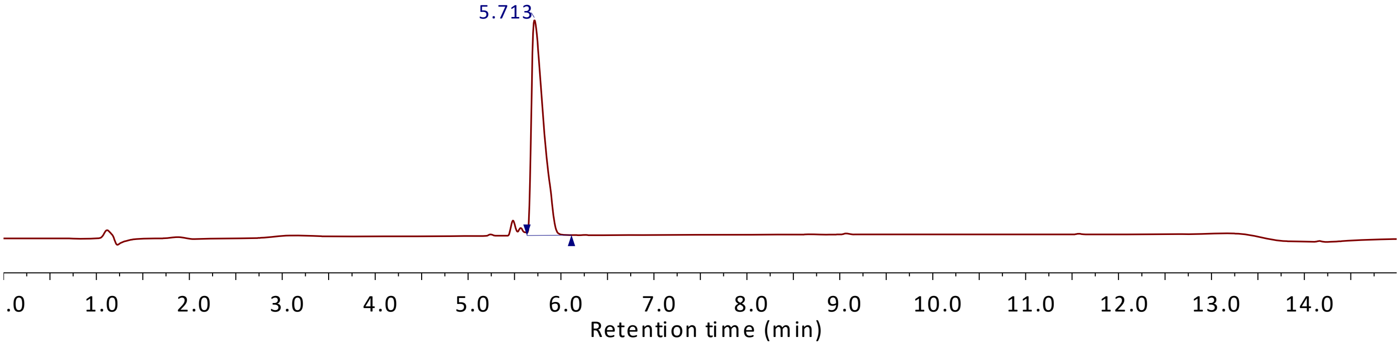

5ab

1H-NMR

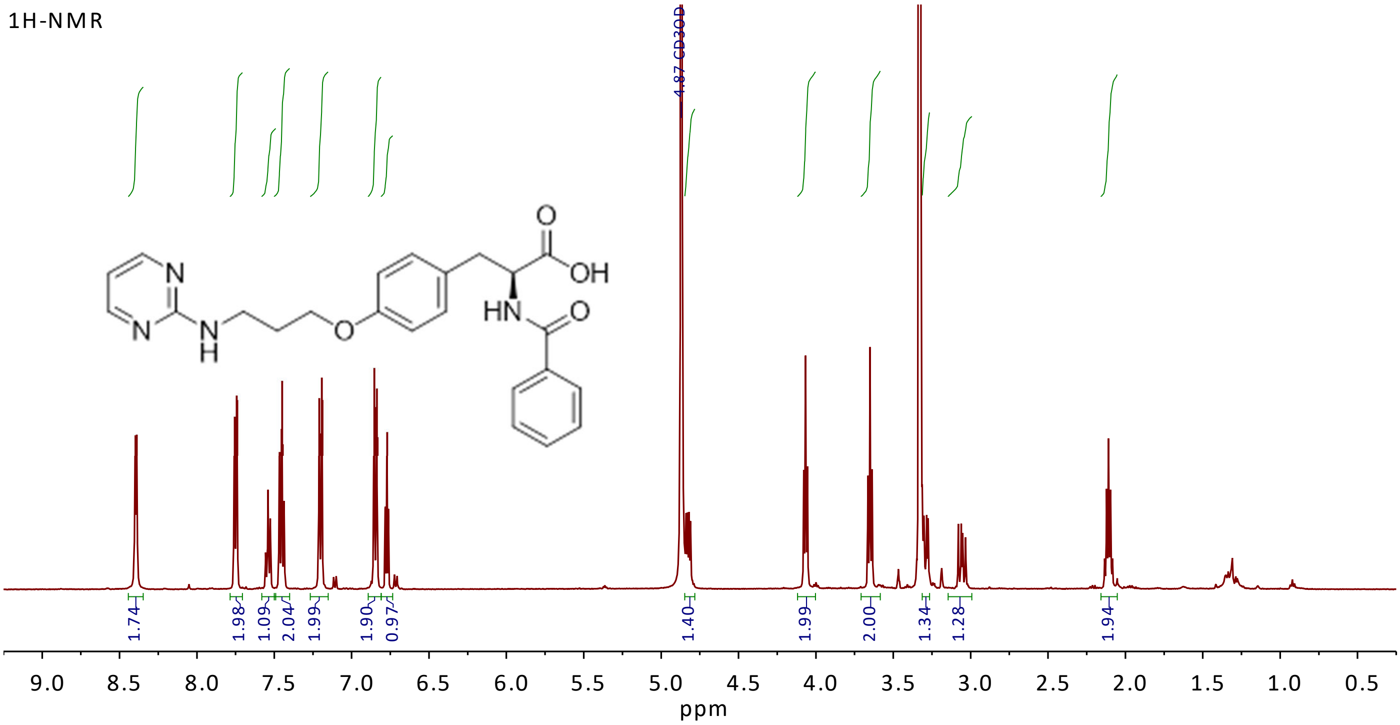

13C-NMR

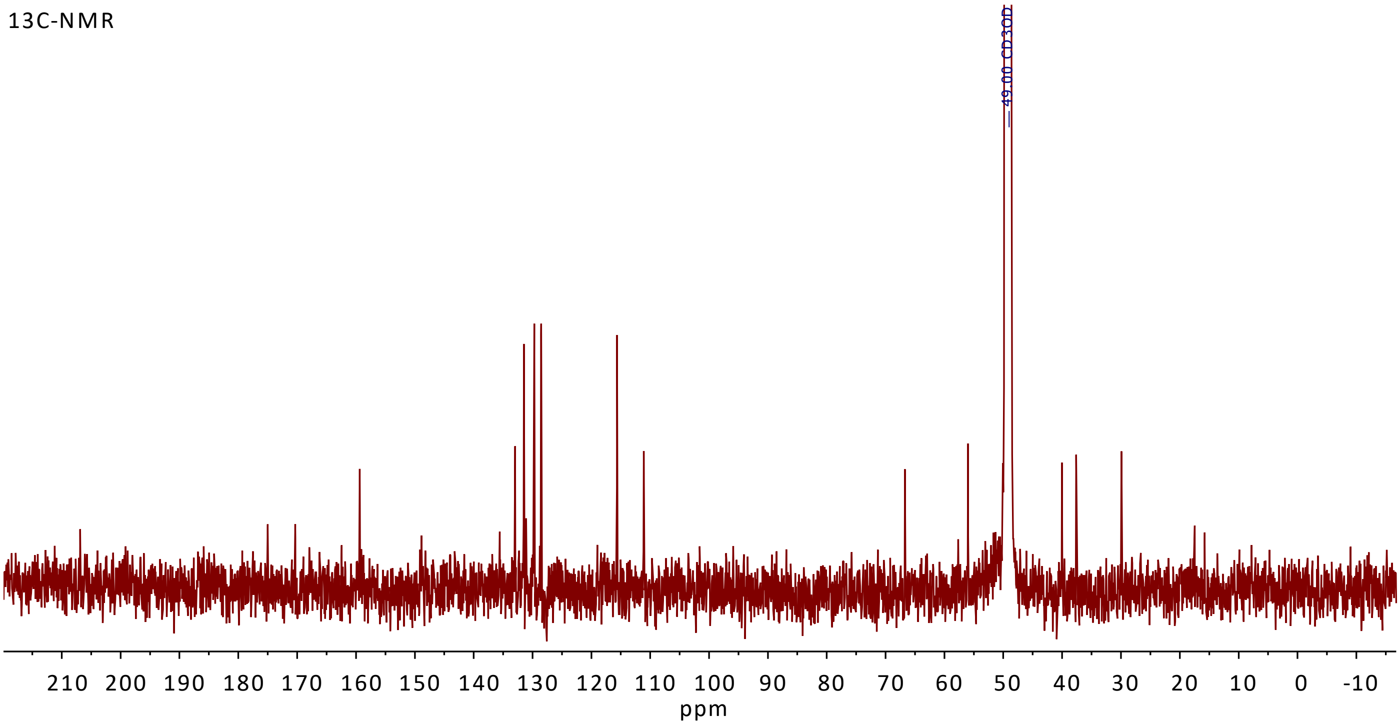

254 nm (LC-MS)

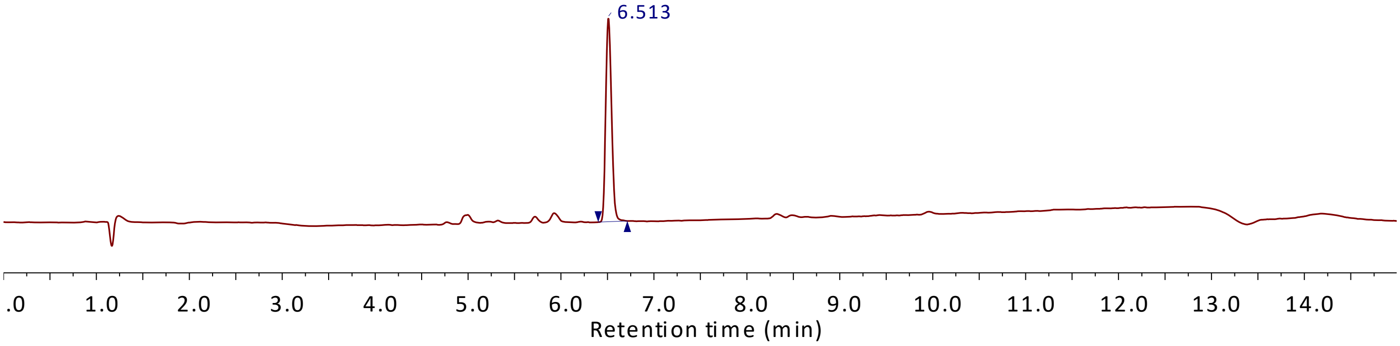

5bb

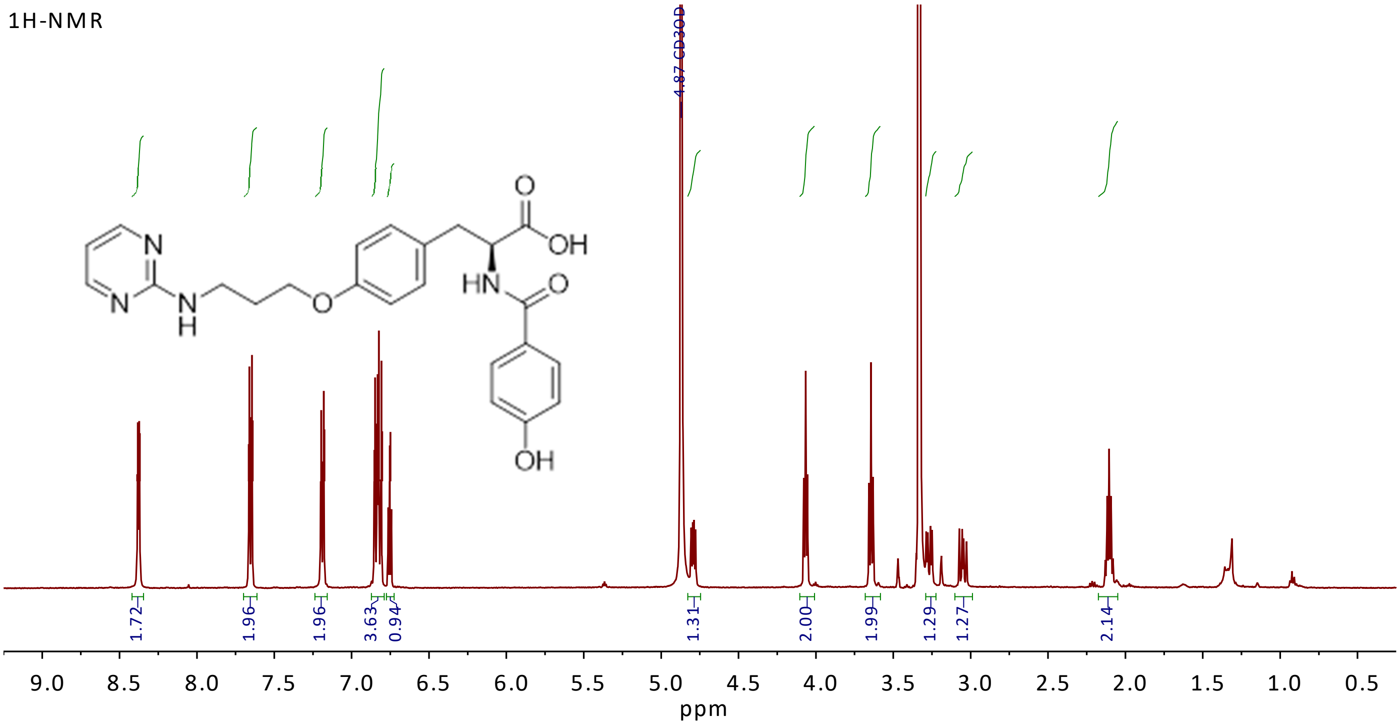
1H-NMR
4.87 CD3OD
1.72
1.96
1.96
3.63
0.94
1.31
2.00
1.99
1.29
1.27
2.14
9.0
8.5
8.0
7.5
7.0
6.5
6.0
5.5
5.0
4.5
4.0
3.5
3.0
2.5
2.0
1.5
1.0
0.5
ppm
N
N
N
H
O
OH
O
HN
O
OH

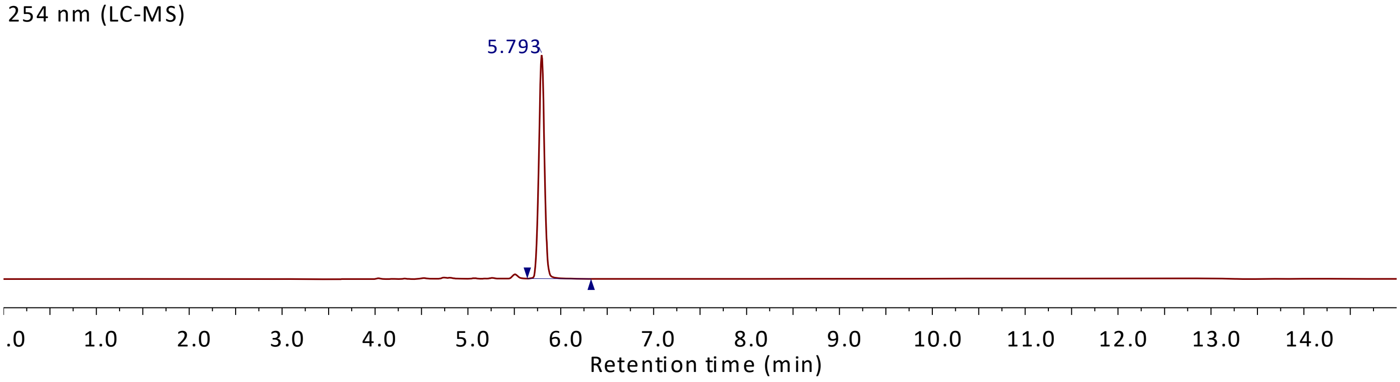
254 nm (LC-MS)
5.793
.0
1.0
2.0
3.0
4.0
5.0
6.0
7.0
8.0
9.0
10.0
11.0
12.0
13.0
14.0
Retention time (min)

5cb

1H-NMR

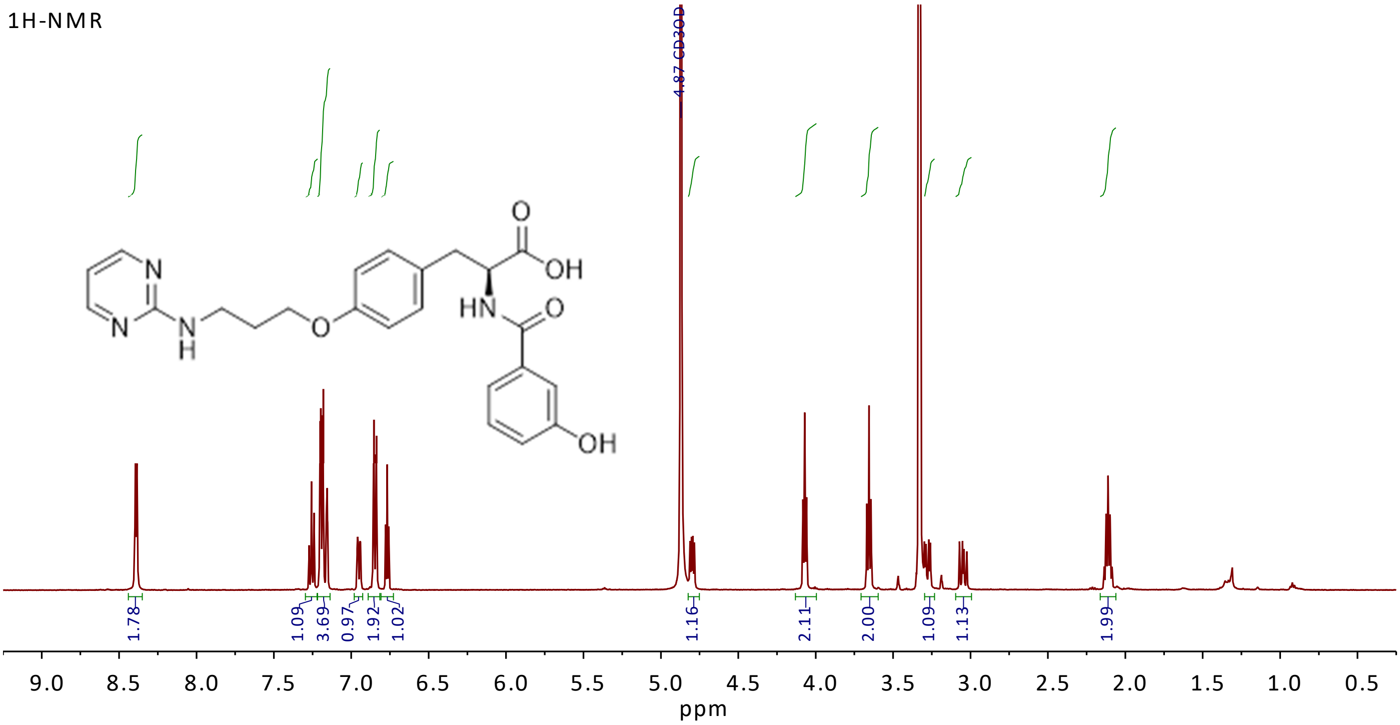

13C-NMR

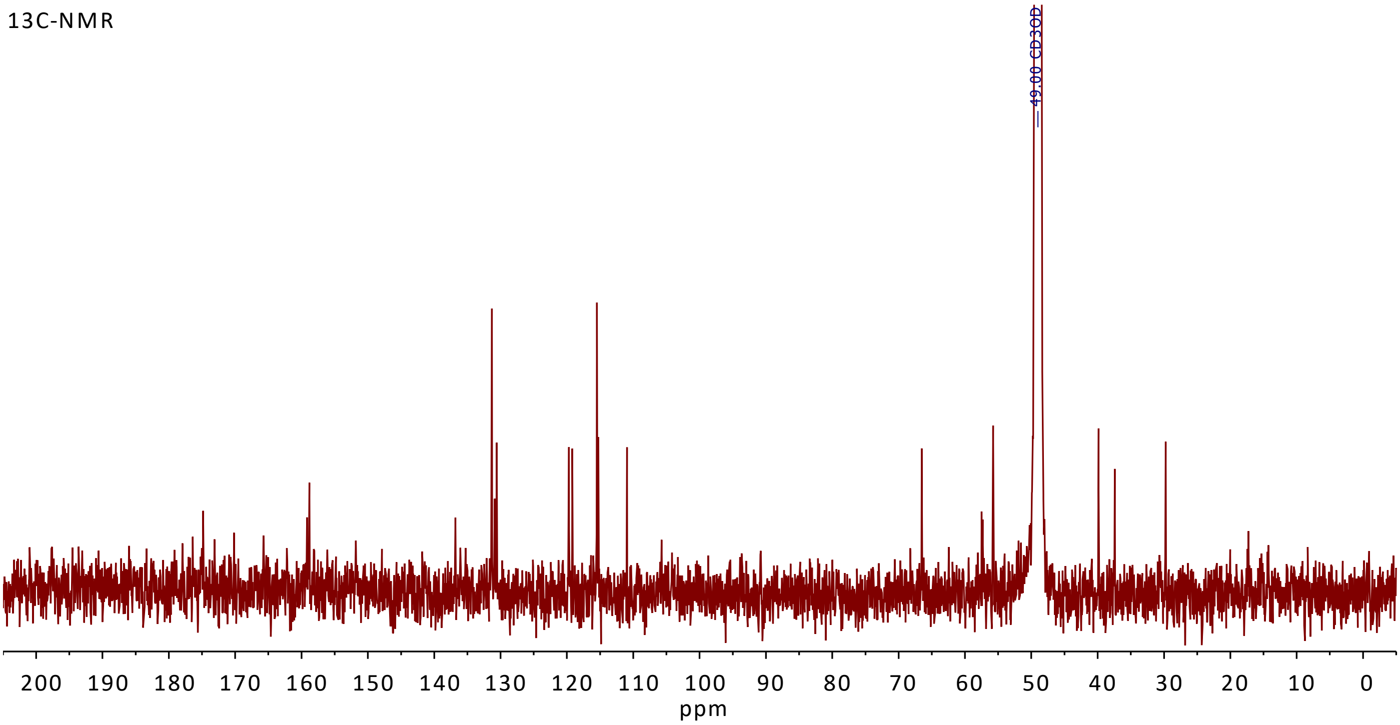

254 nm (LC-MS)

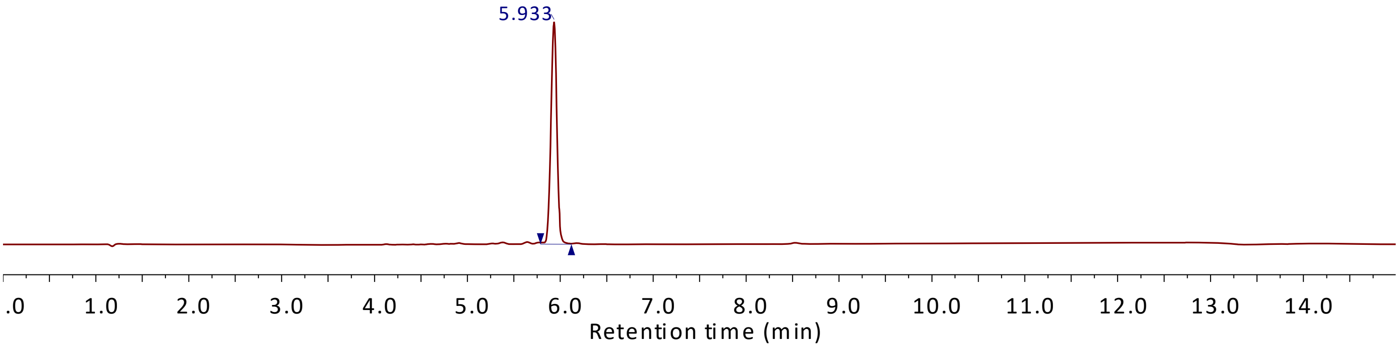

5db

1H-NMR

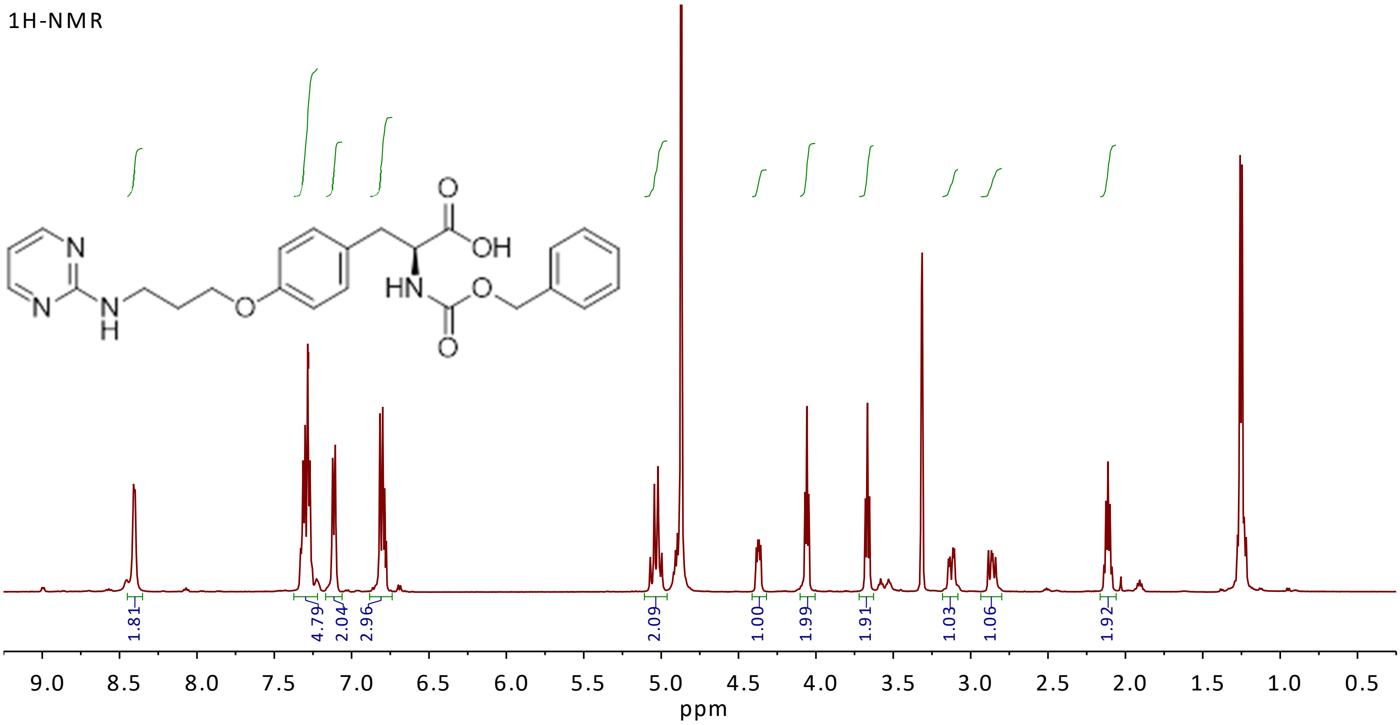

13C-NMR

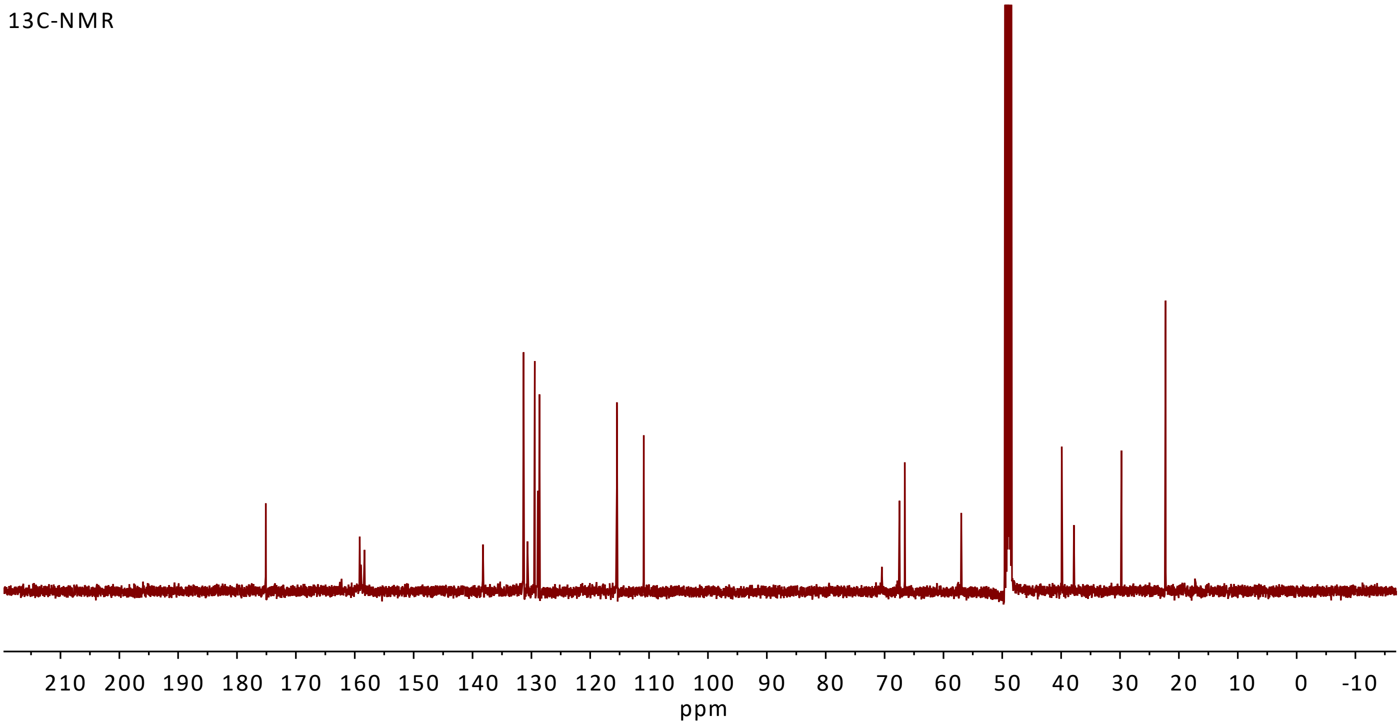

220 nm (LC-MS)

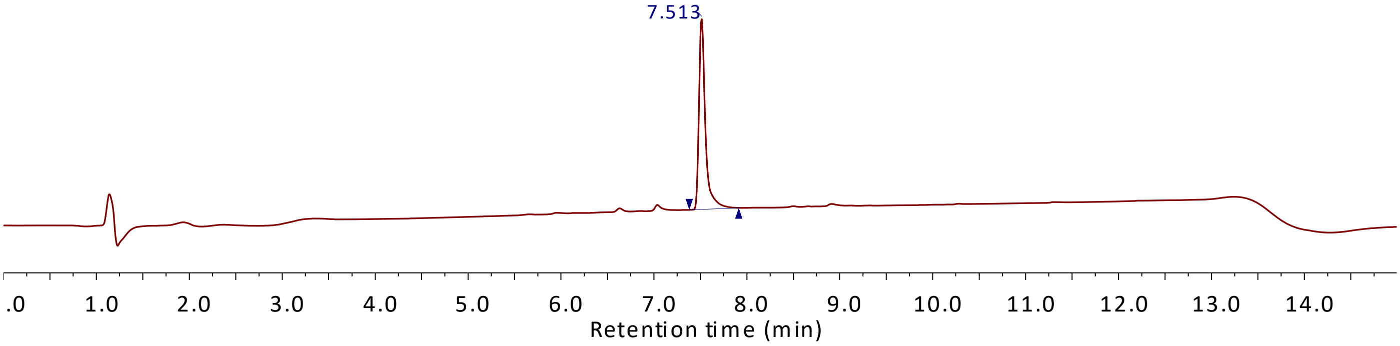

6ab

1H-NMR

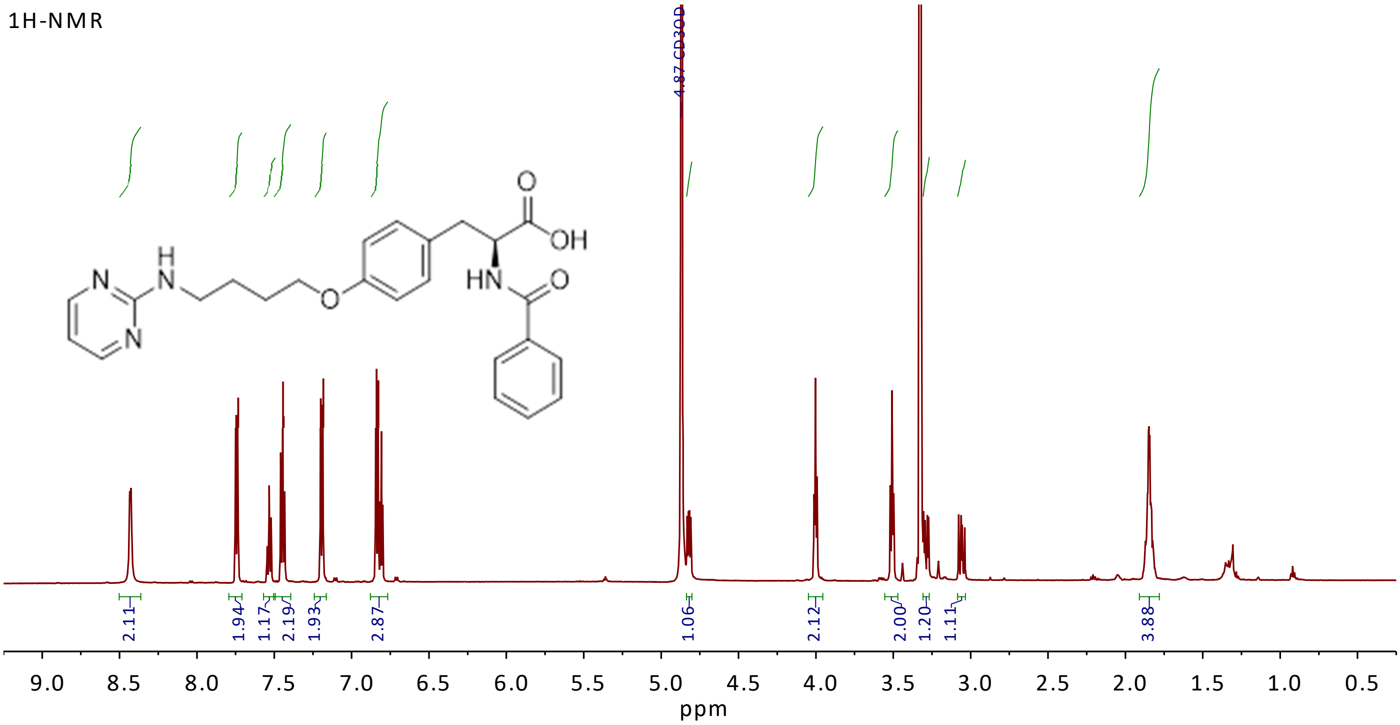

13C-NMR

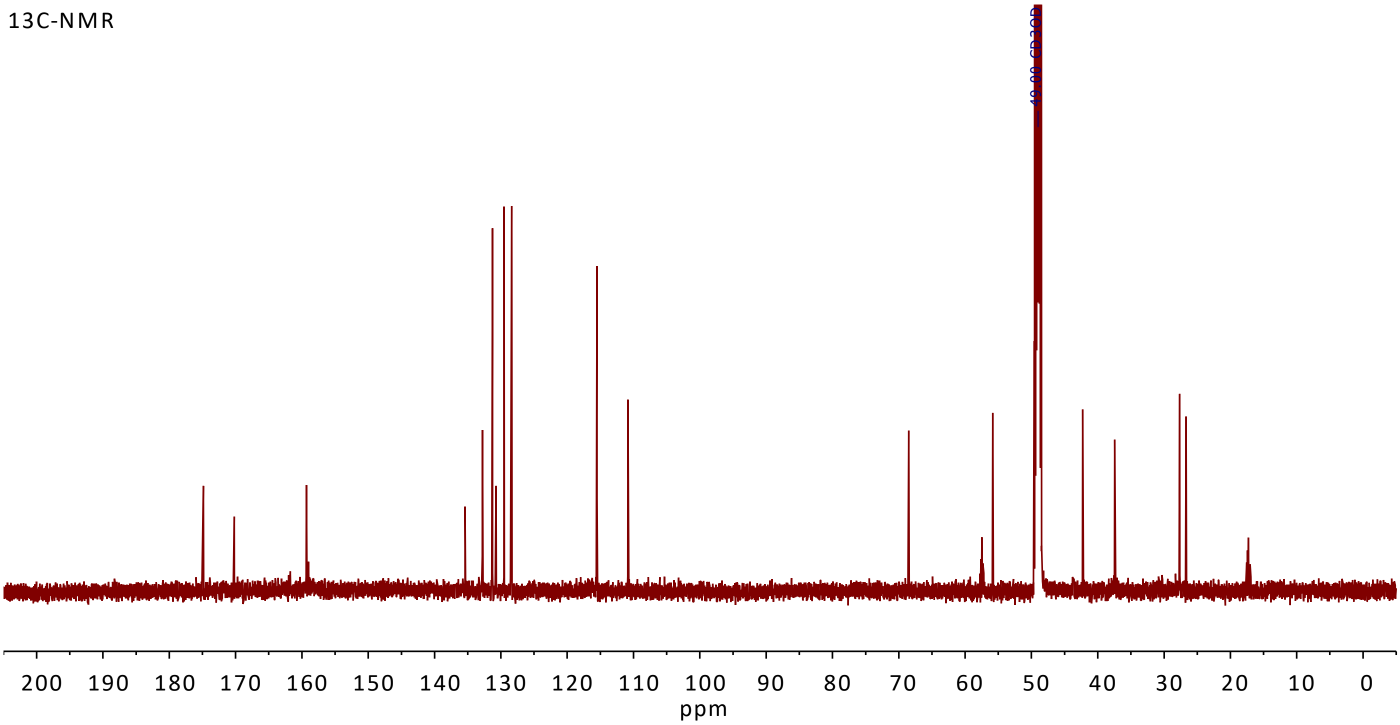

254 nm (LC-MS)

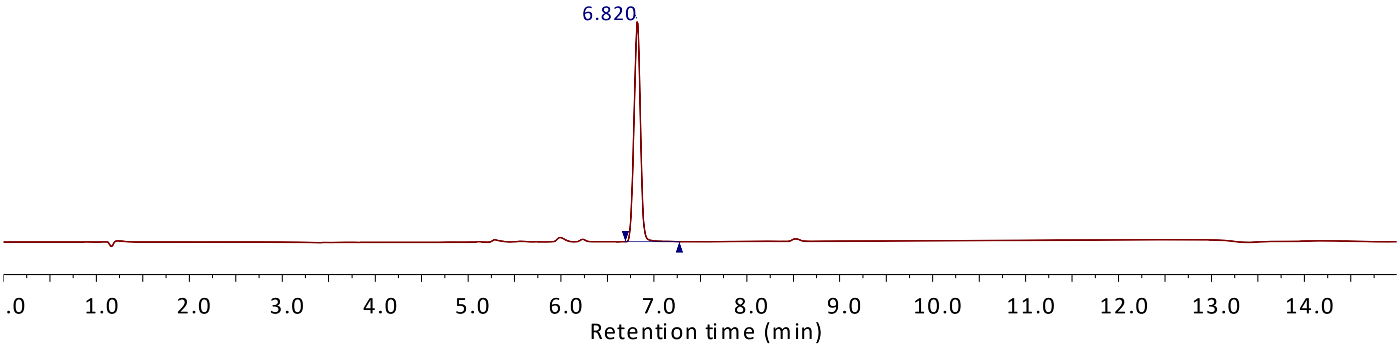

6bb

1H-NMR

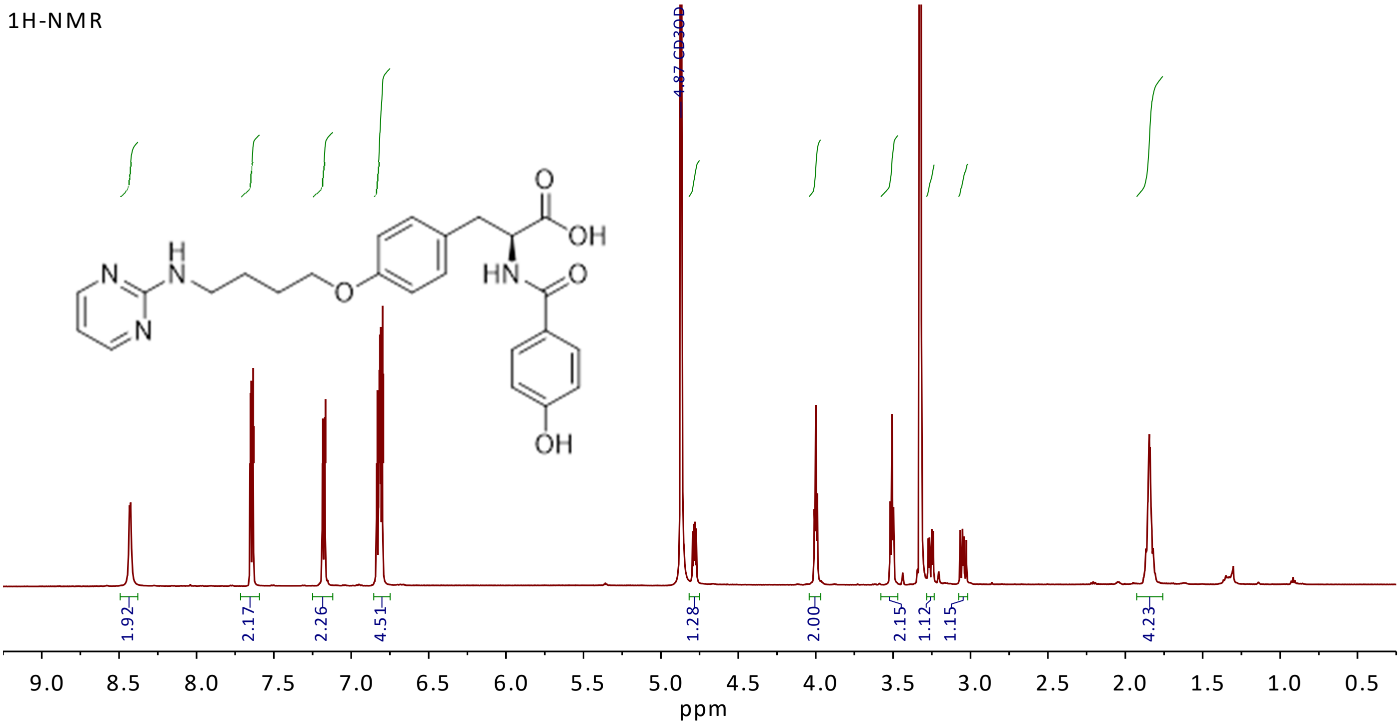

13C-NMR

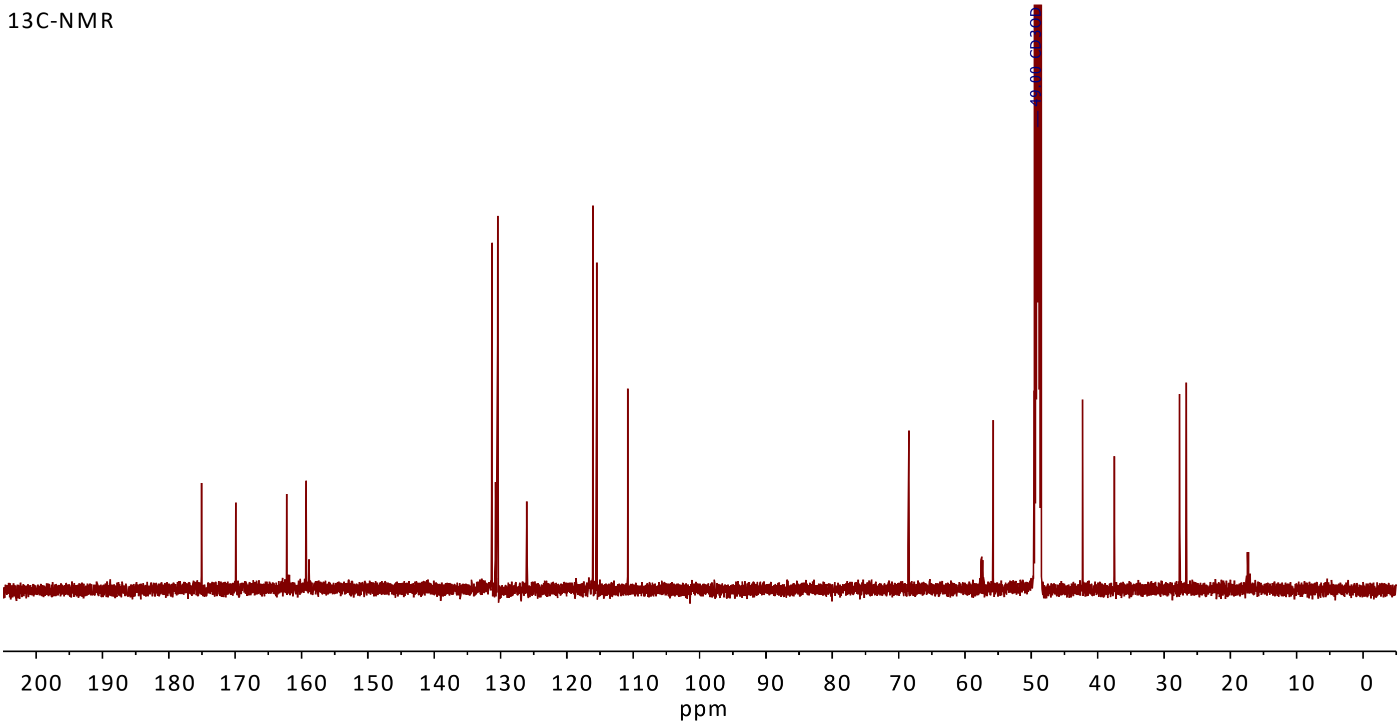

254 nm (LC-MS)

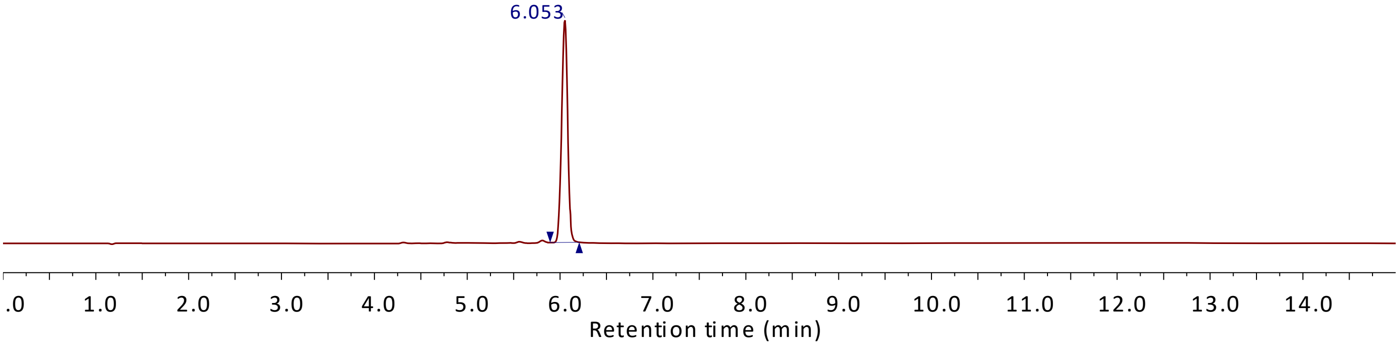

6cb

1H-NMR

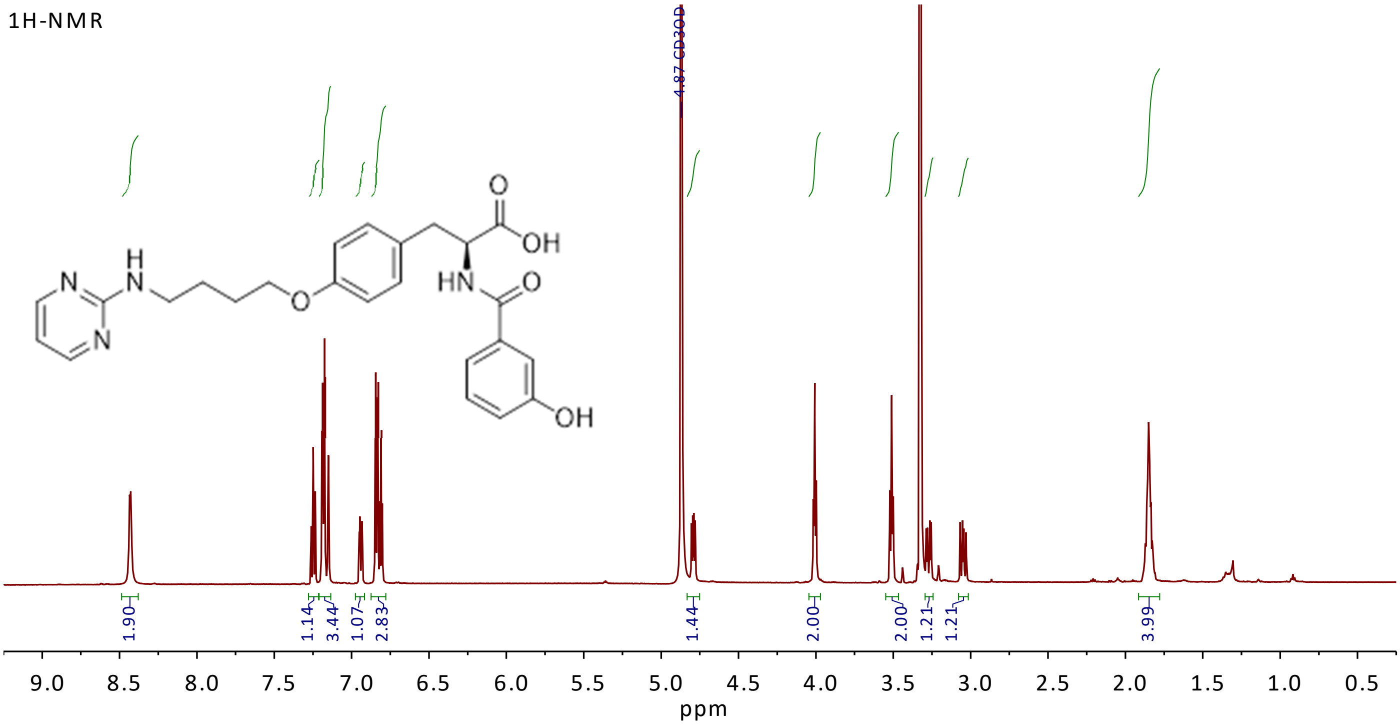

13C-NMR

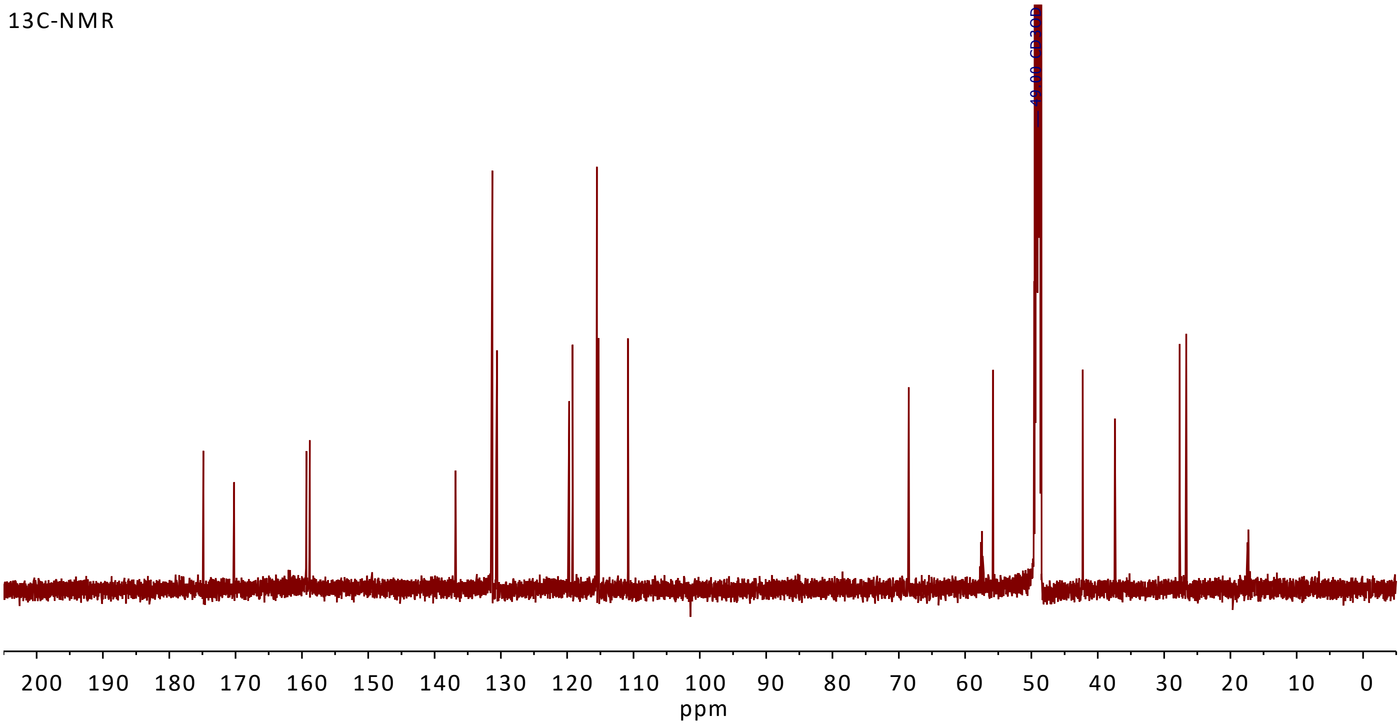

254 nm (LC-MS)

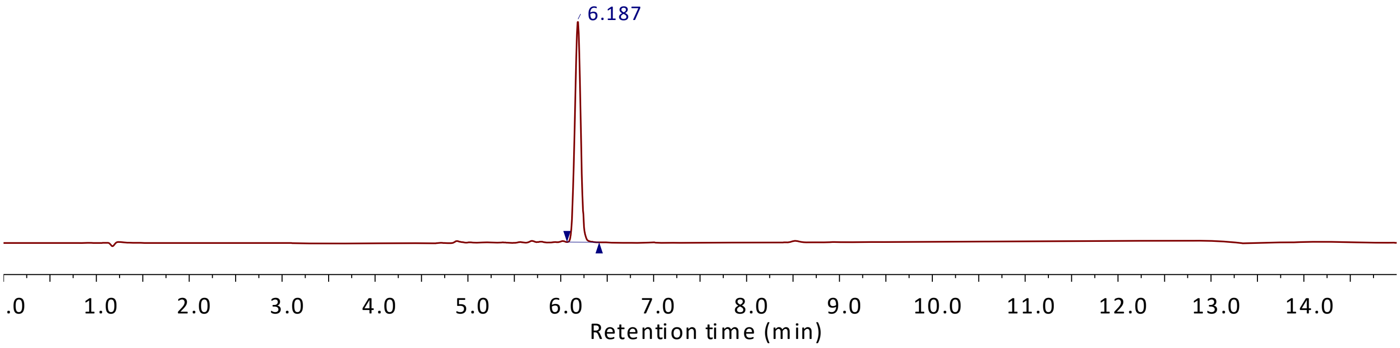

6db

1H-NMR

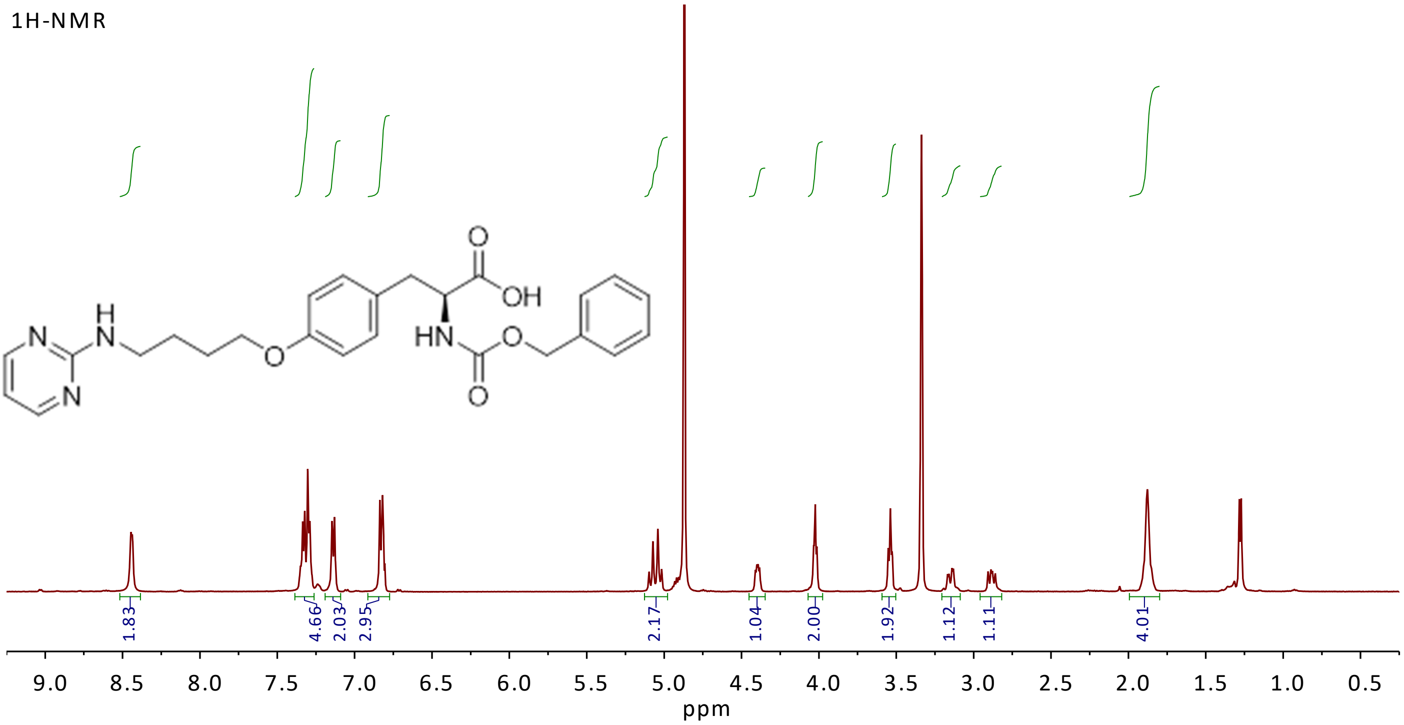

13C-NMR

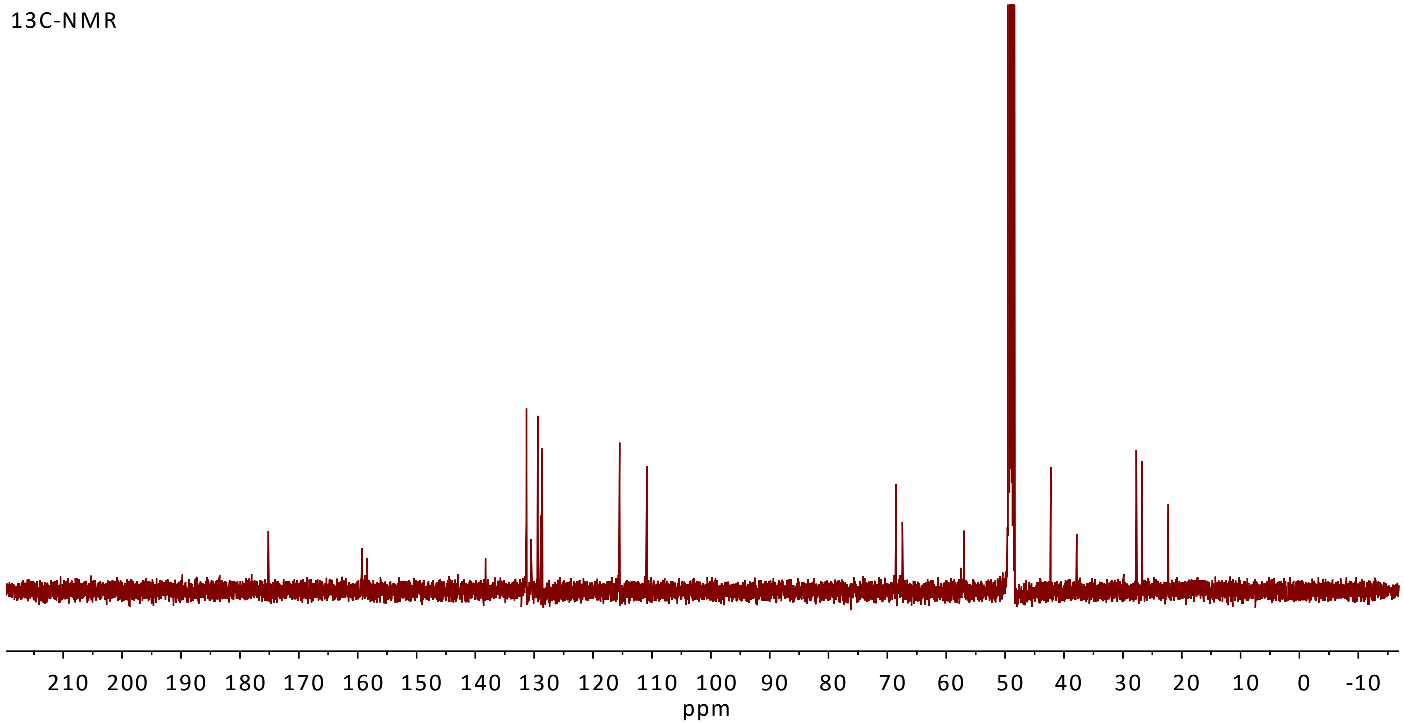

254 nm (LC-MS)

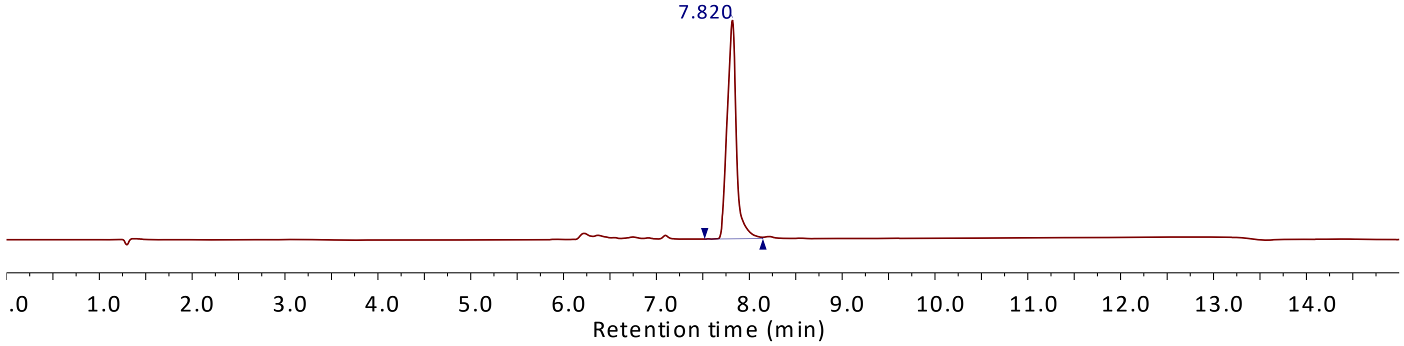

7ab

1H-NMR

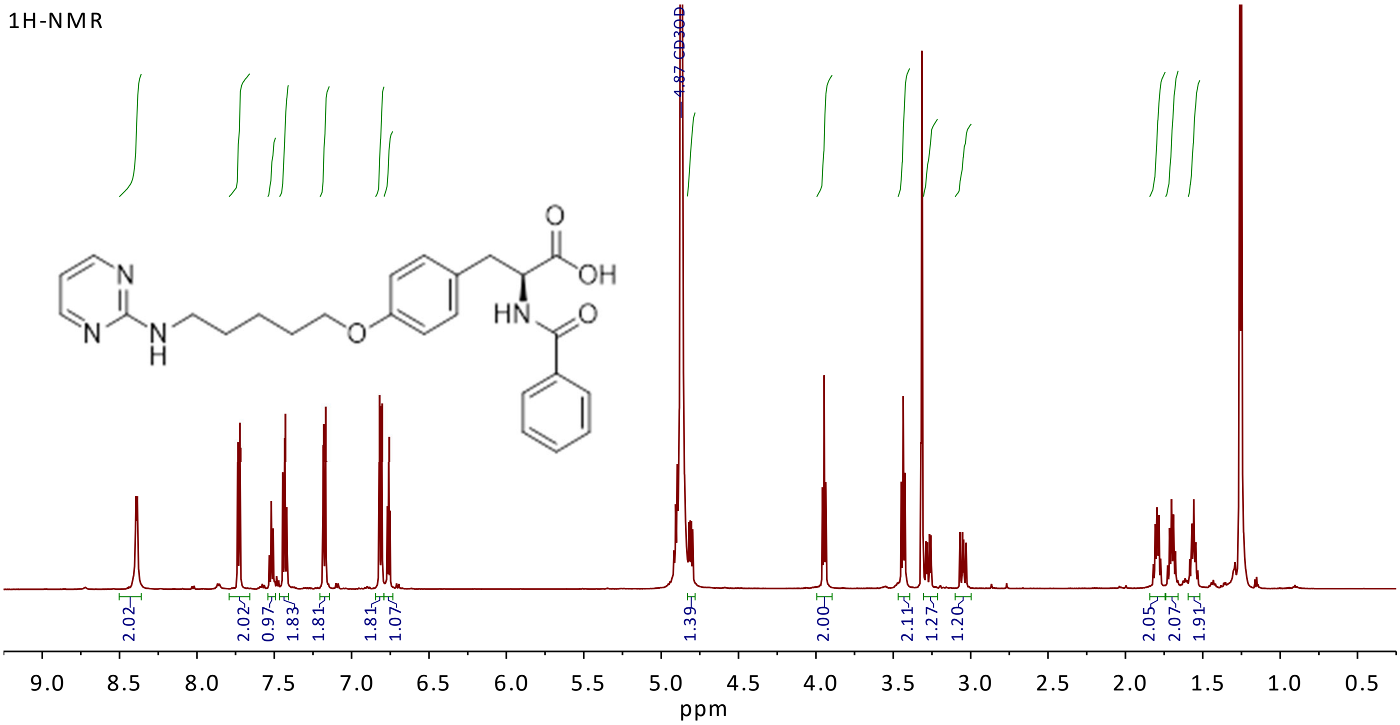

13C-NMR

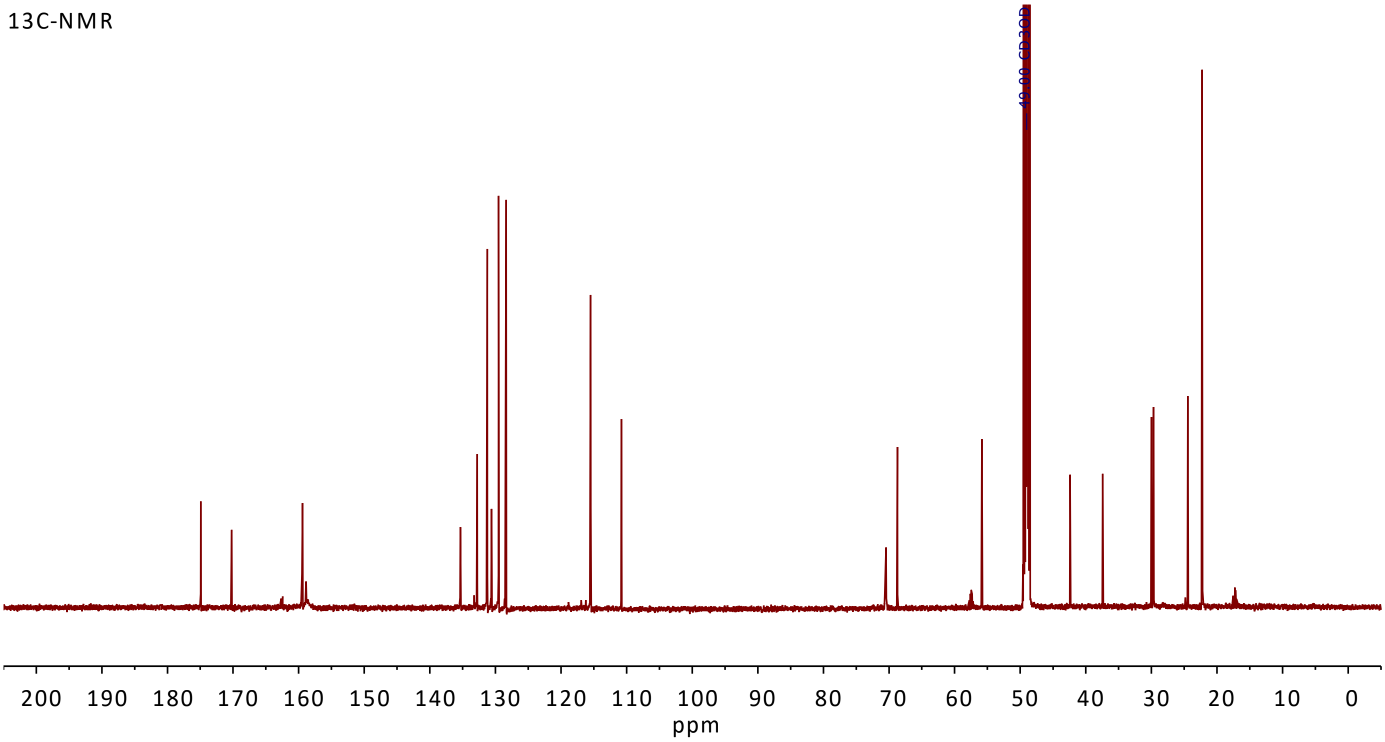

254 nm (LC-MS)

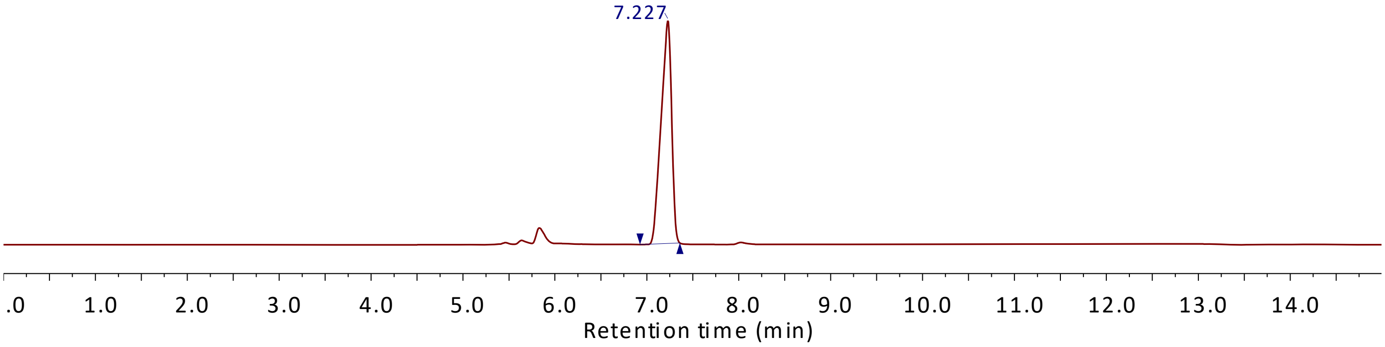

7bb

1H-NMR

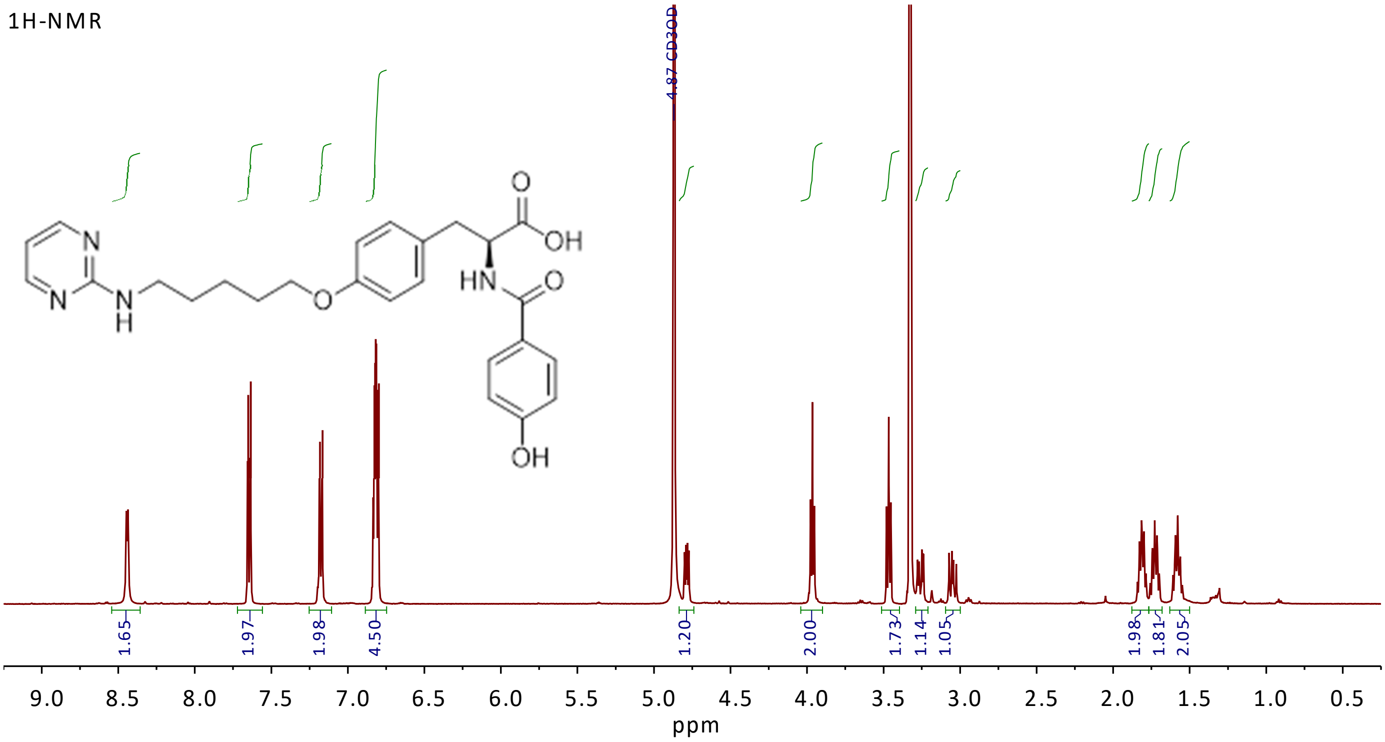

13C-NMR

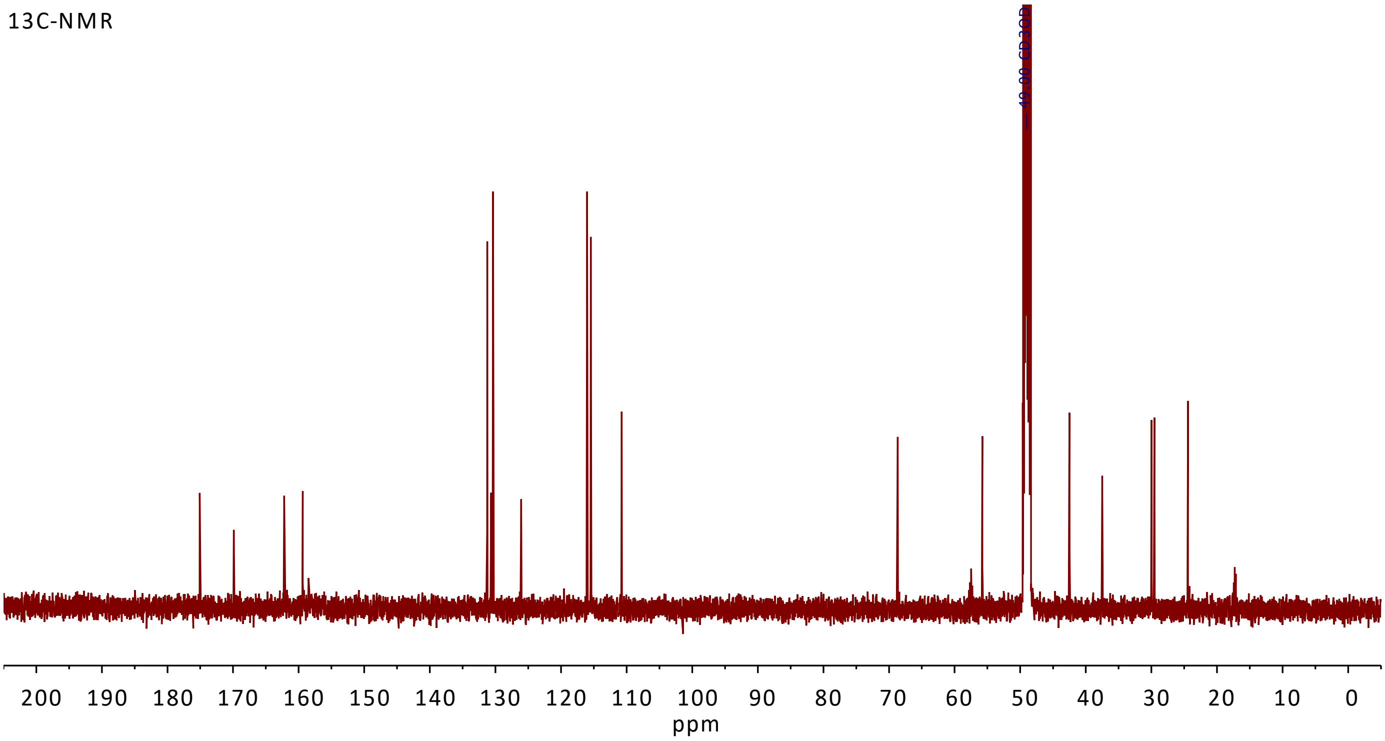

254 nm (LC-MS)

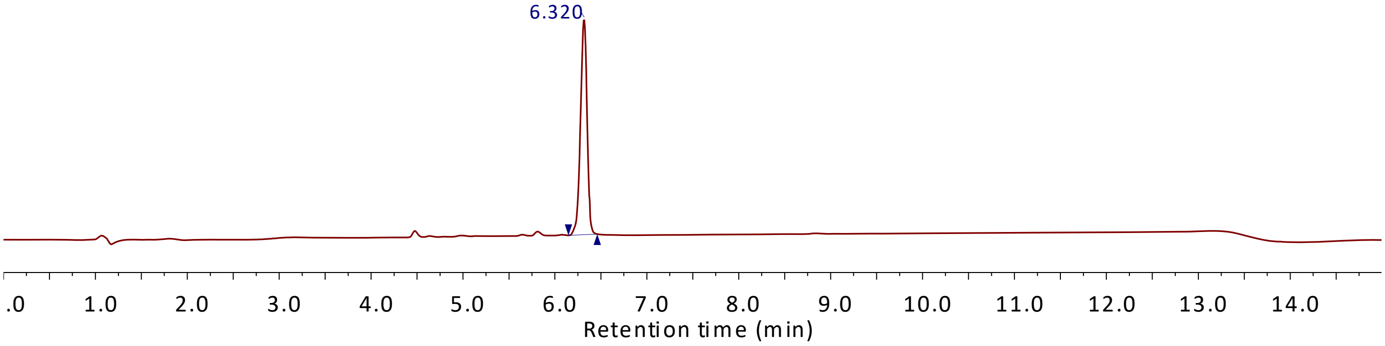

7cb

1H-NMR

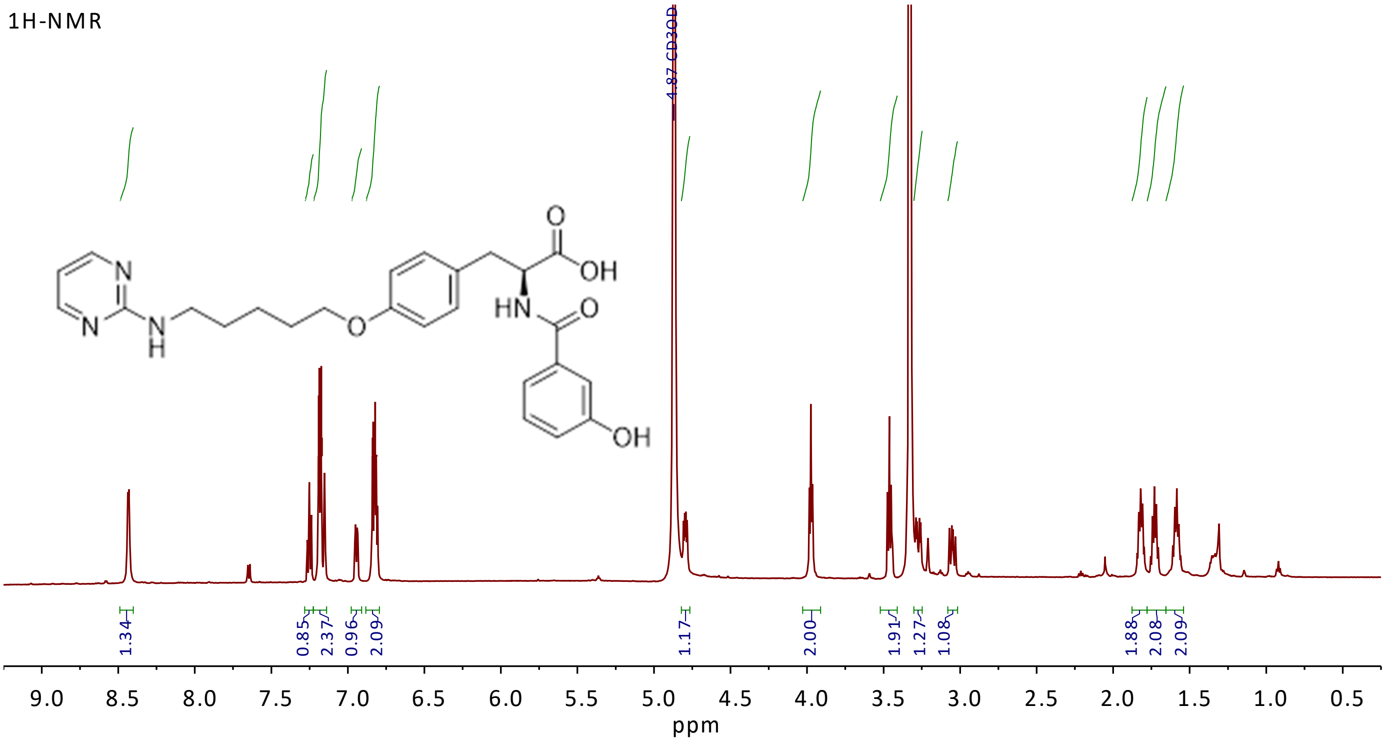

13C-NMR

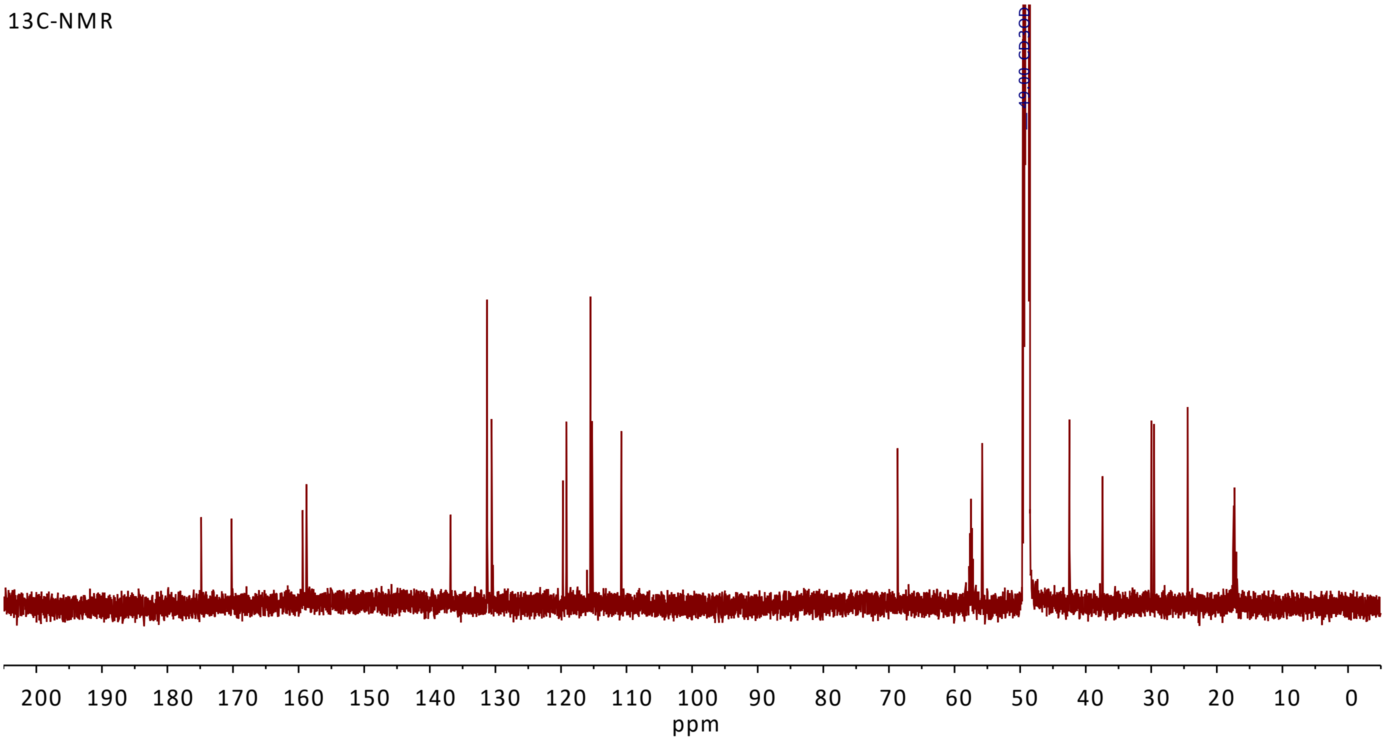

220 nm (LC-MS)

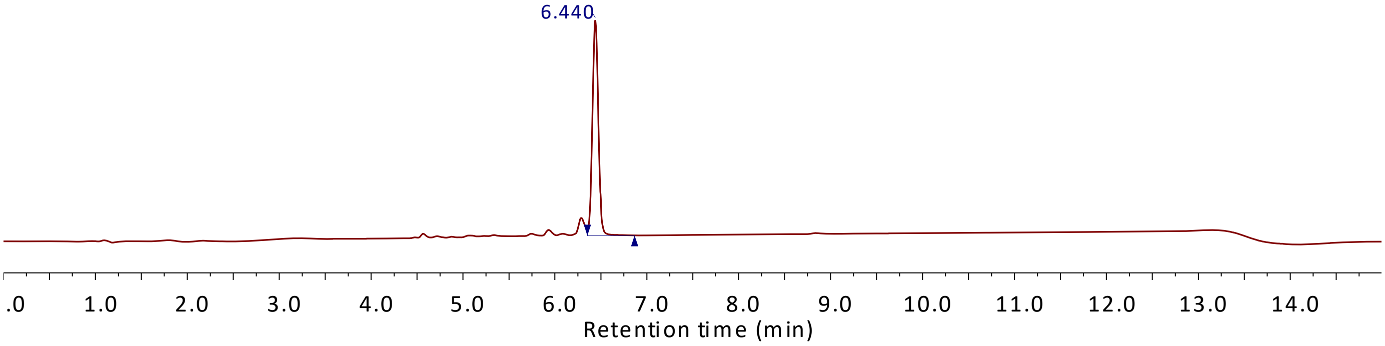

7db

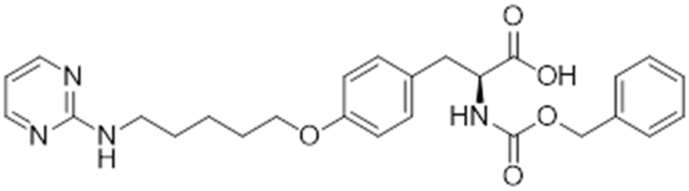

254 nm (LC-MS)

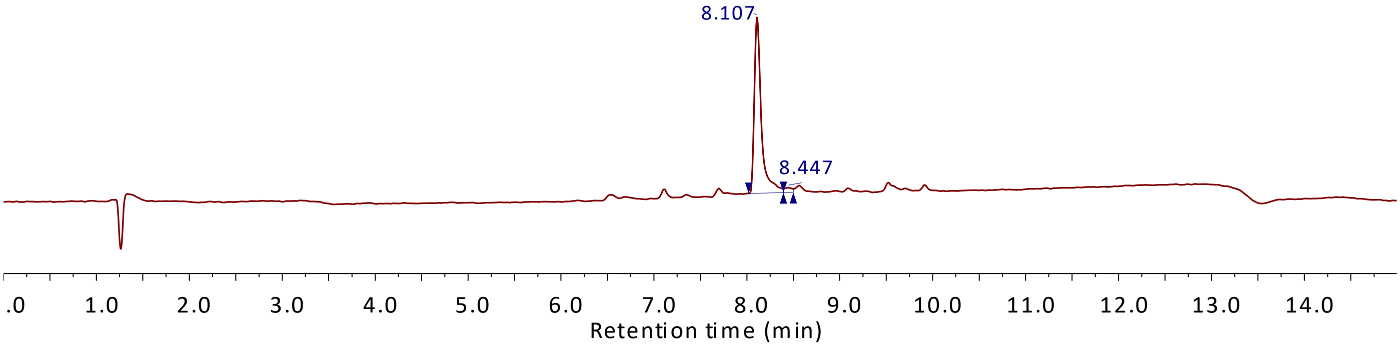

5ac

1H-NMR

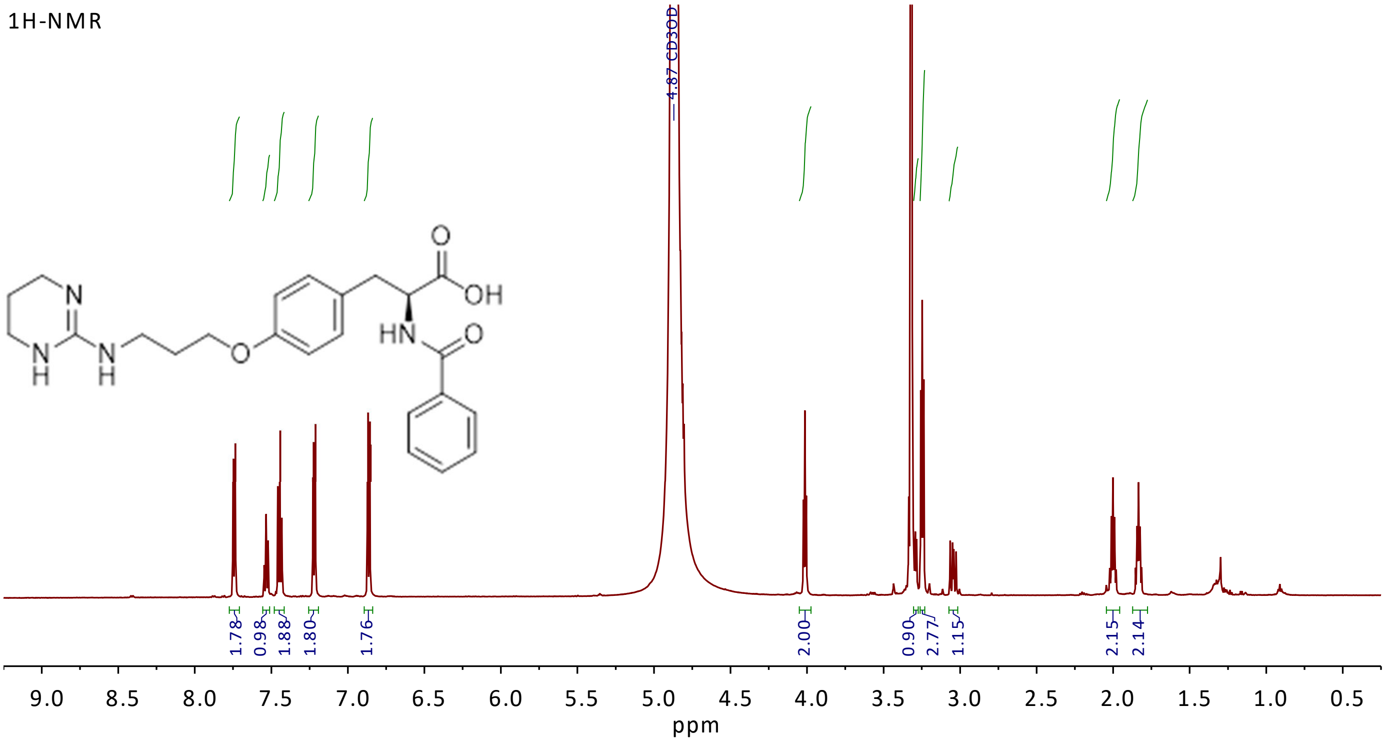

13C-NMR

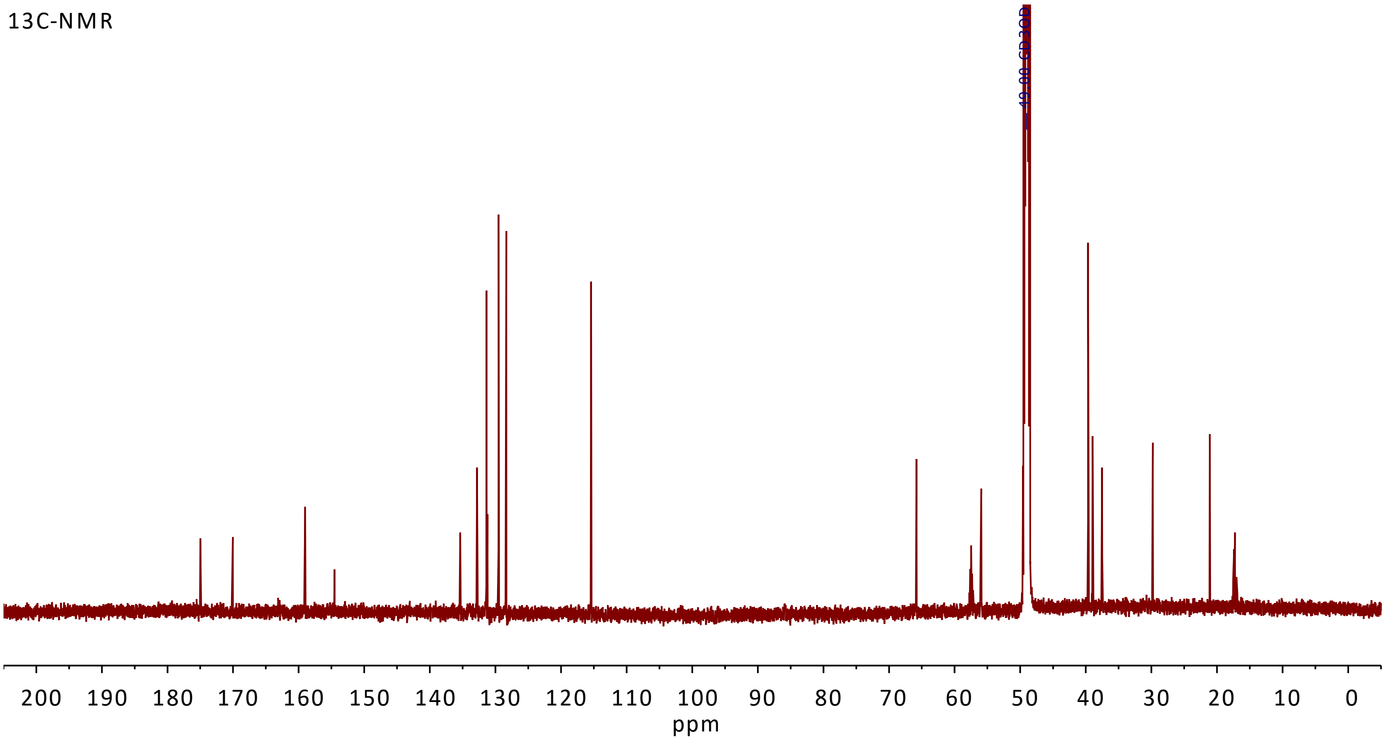

254 nm (LC-MS)

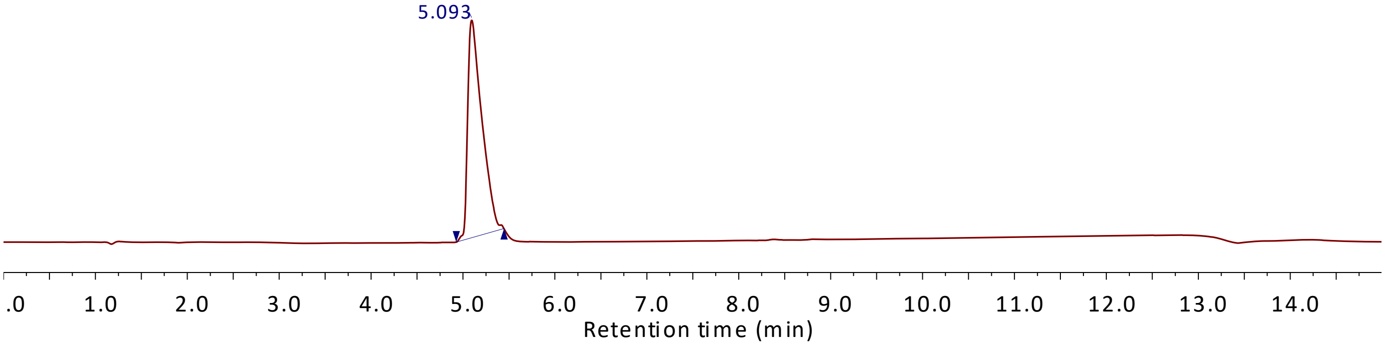

5bc

1H-NMR

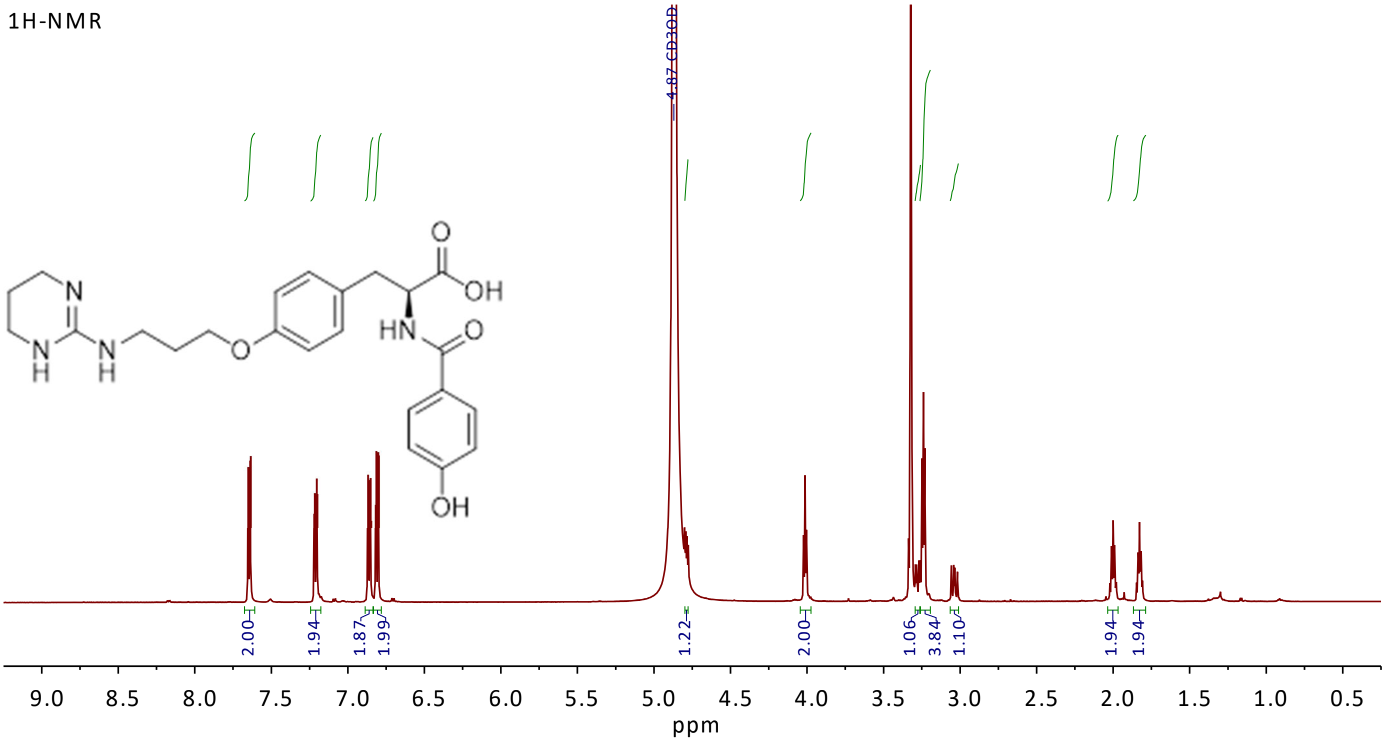

13C-NMR

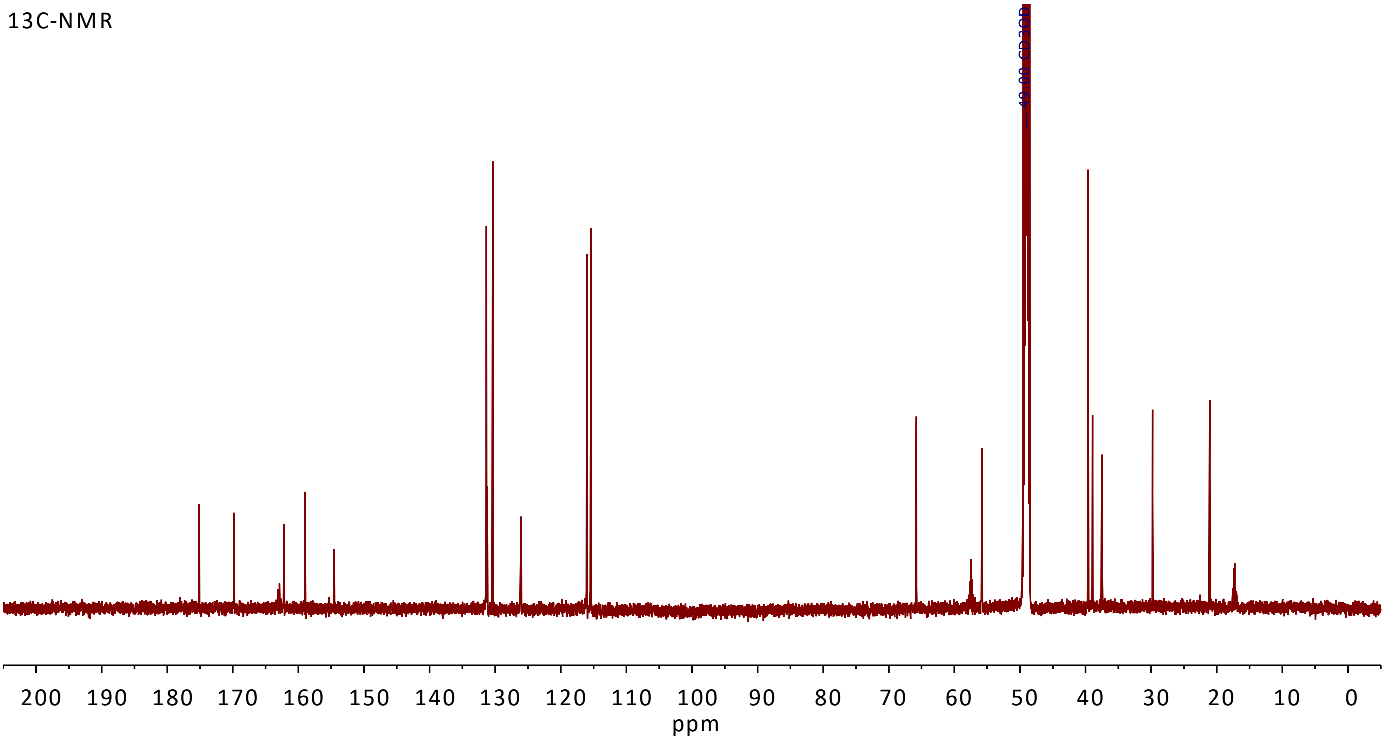

254 nm (LC-MS)

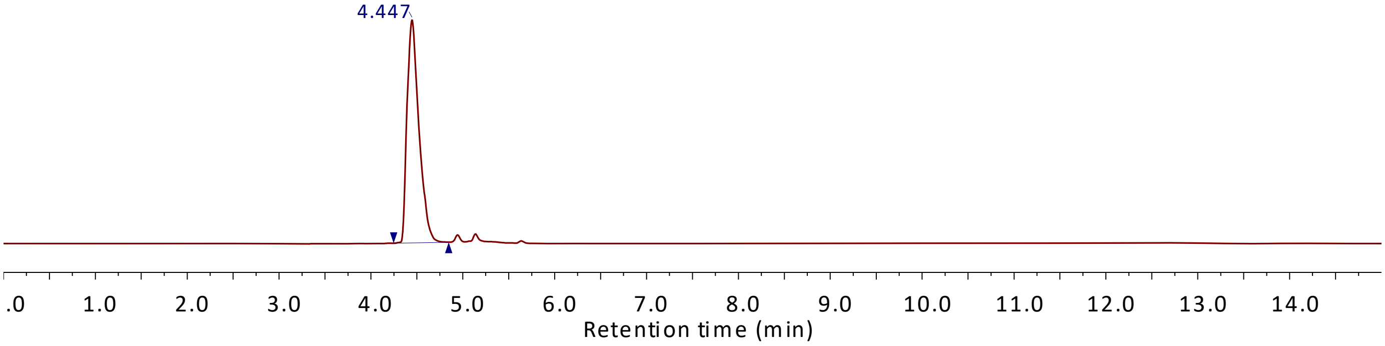

5cc

1H-NMR

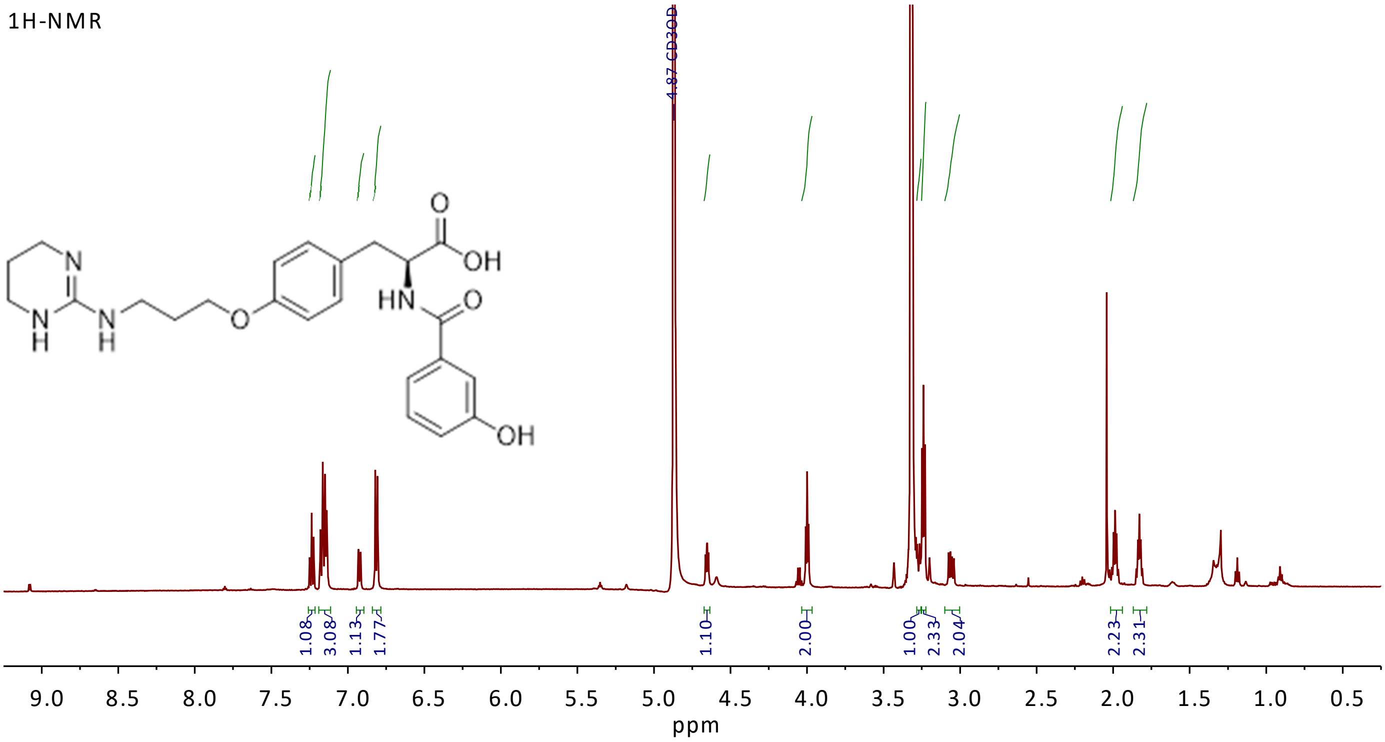

13C-NMR

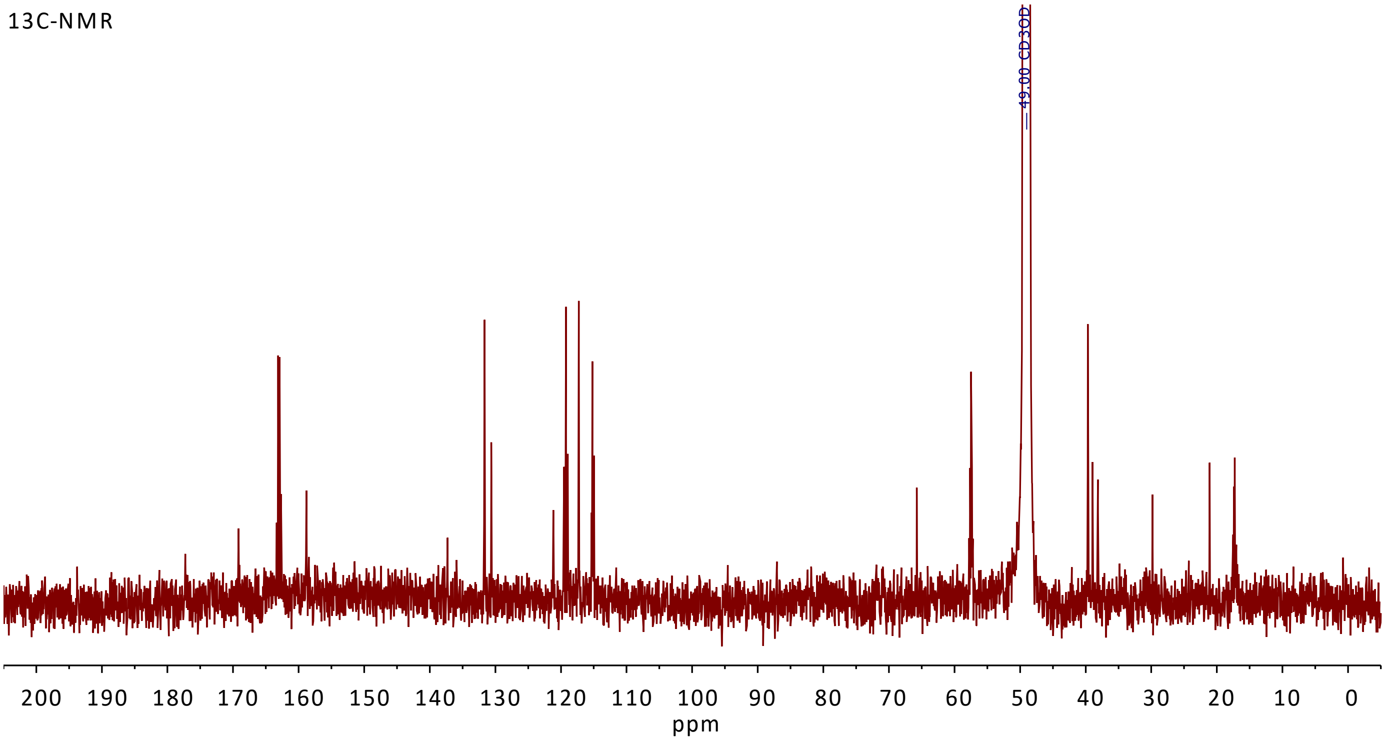

254 nm (LC-MS)

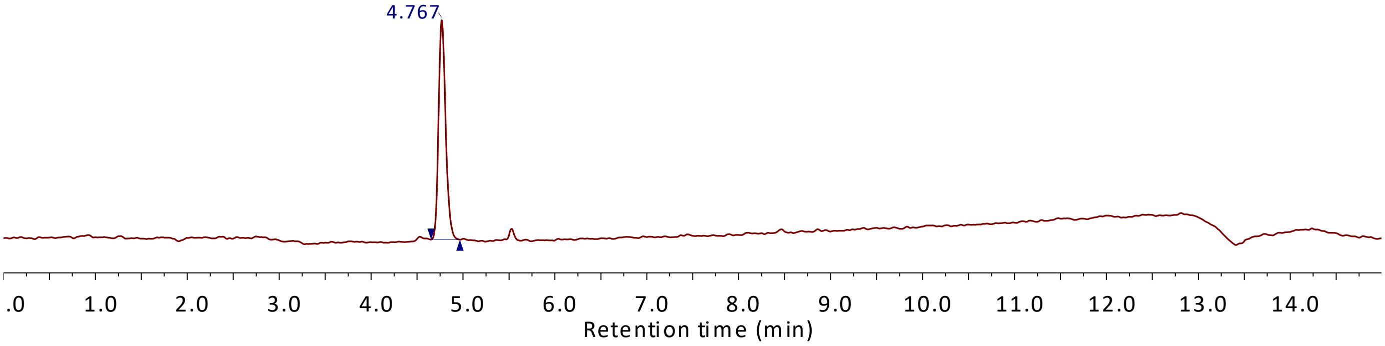

5dc

1H-NMR

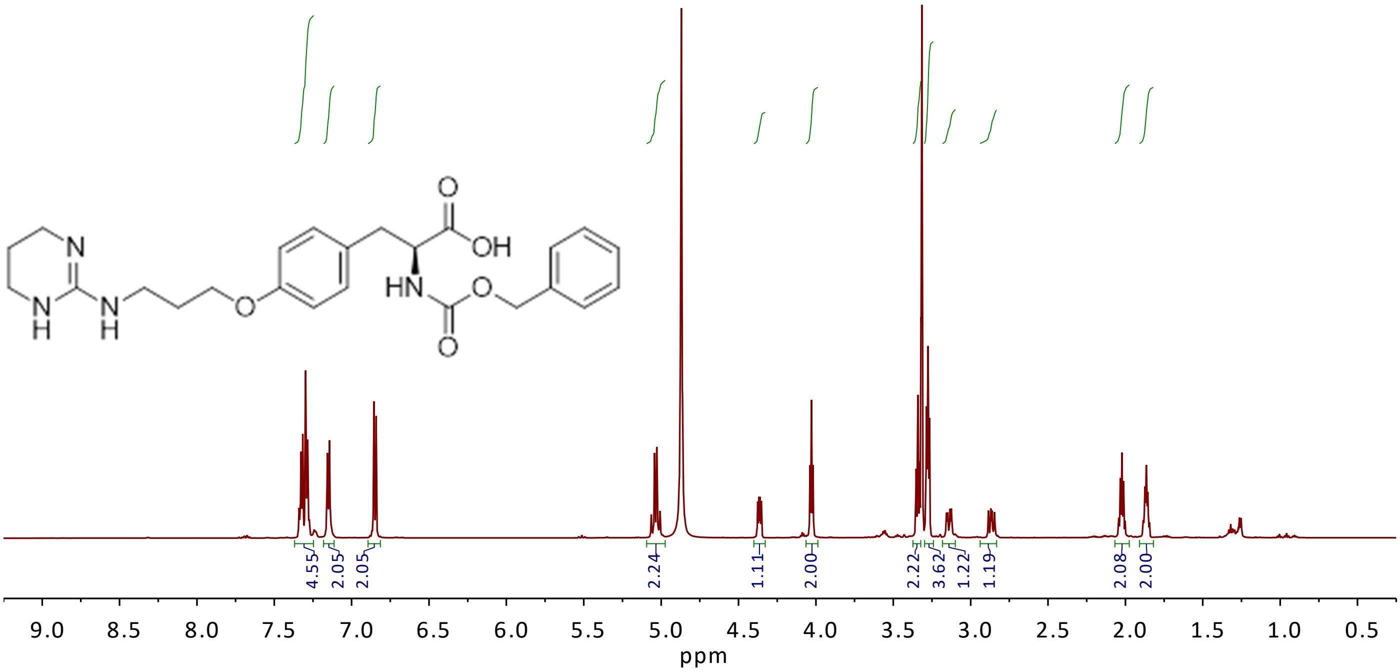

13C-NMR

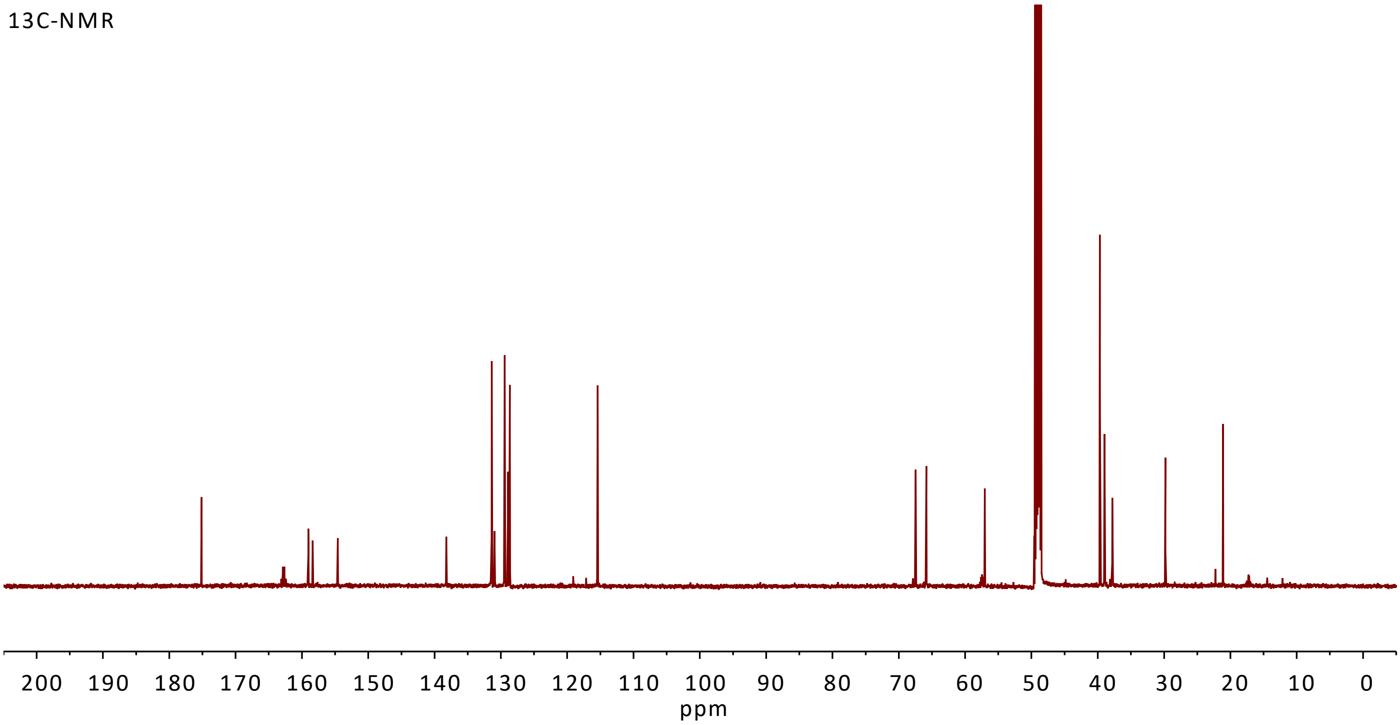

220 nm (LC-MS)

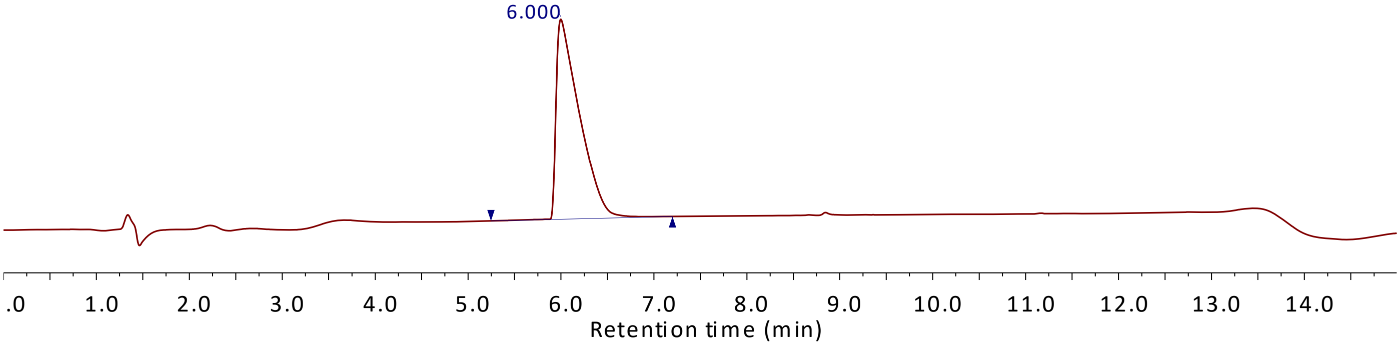

6ac

1H-NMR

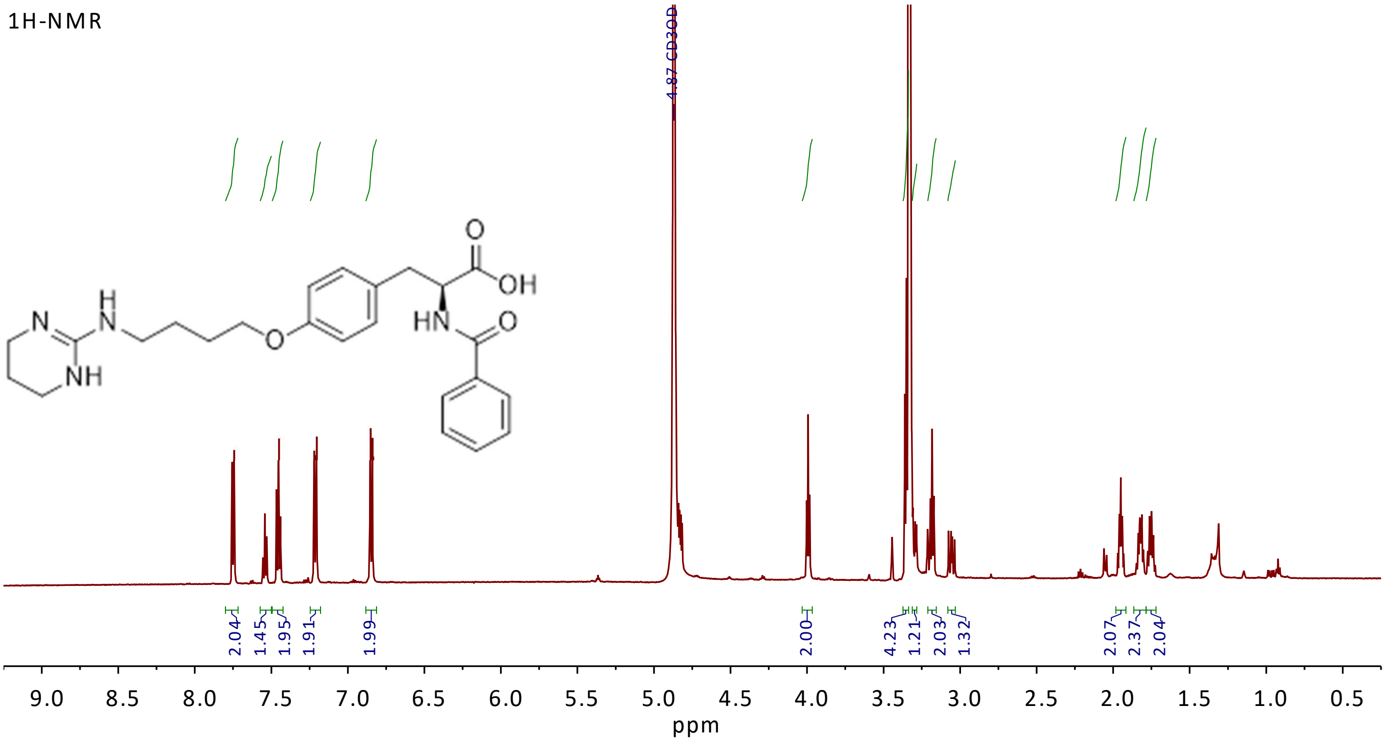

13C-NMR

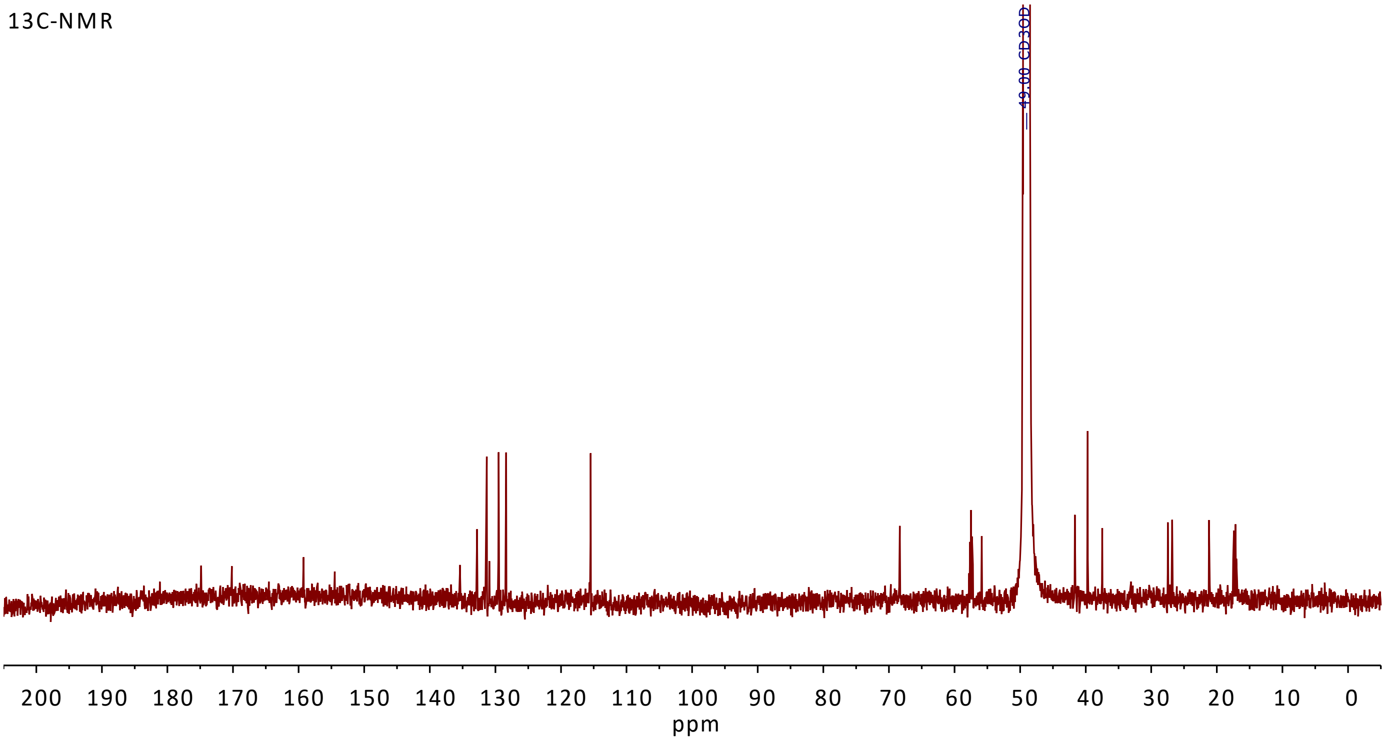

254 nm (LC-MS)

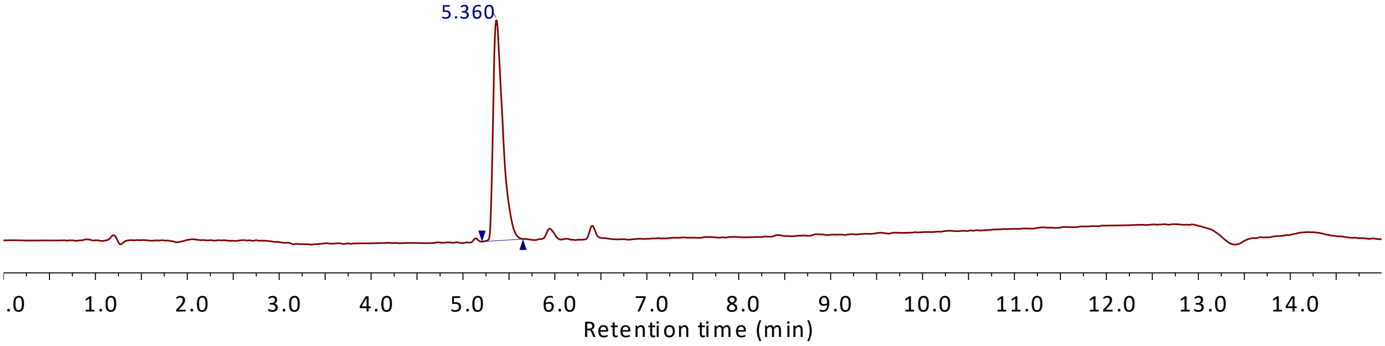

6bc

1H-NMR

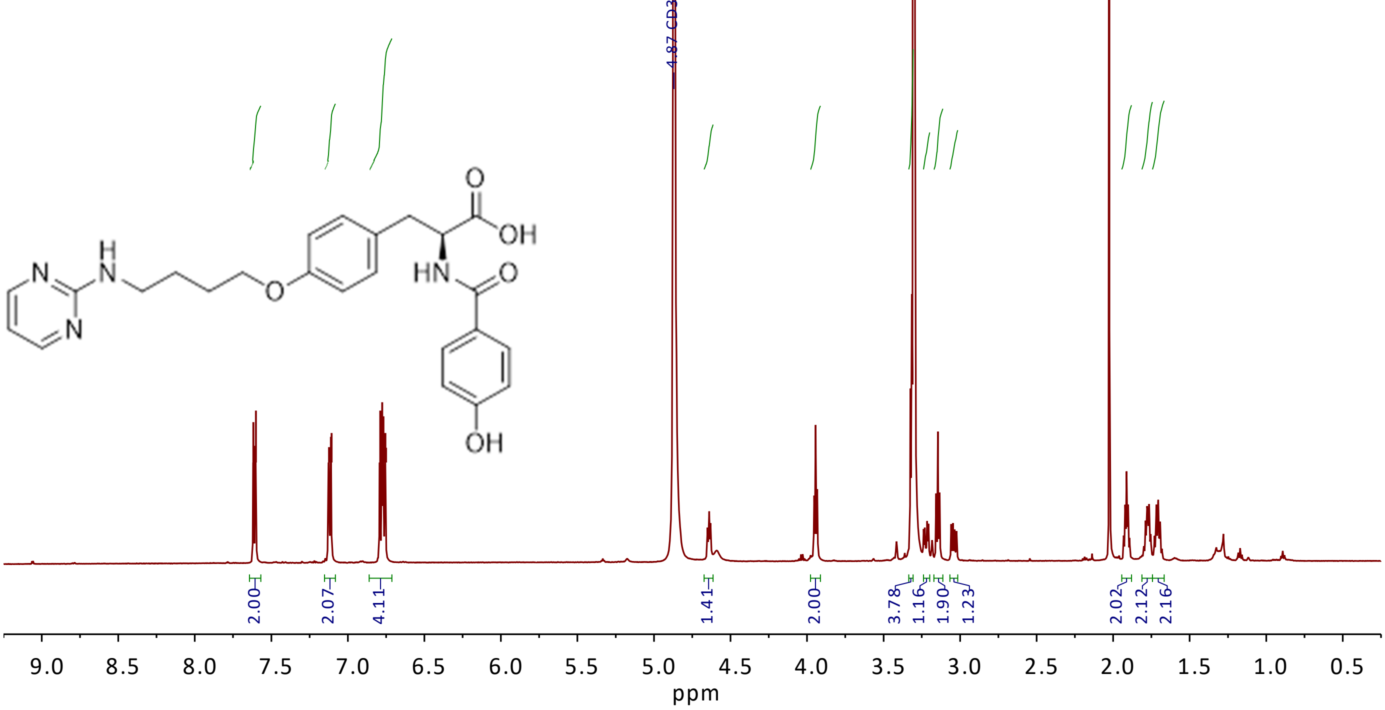

13C-NMR

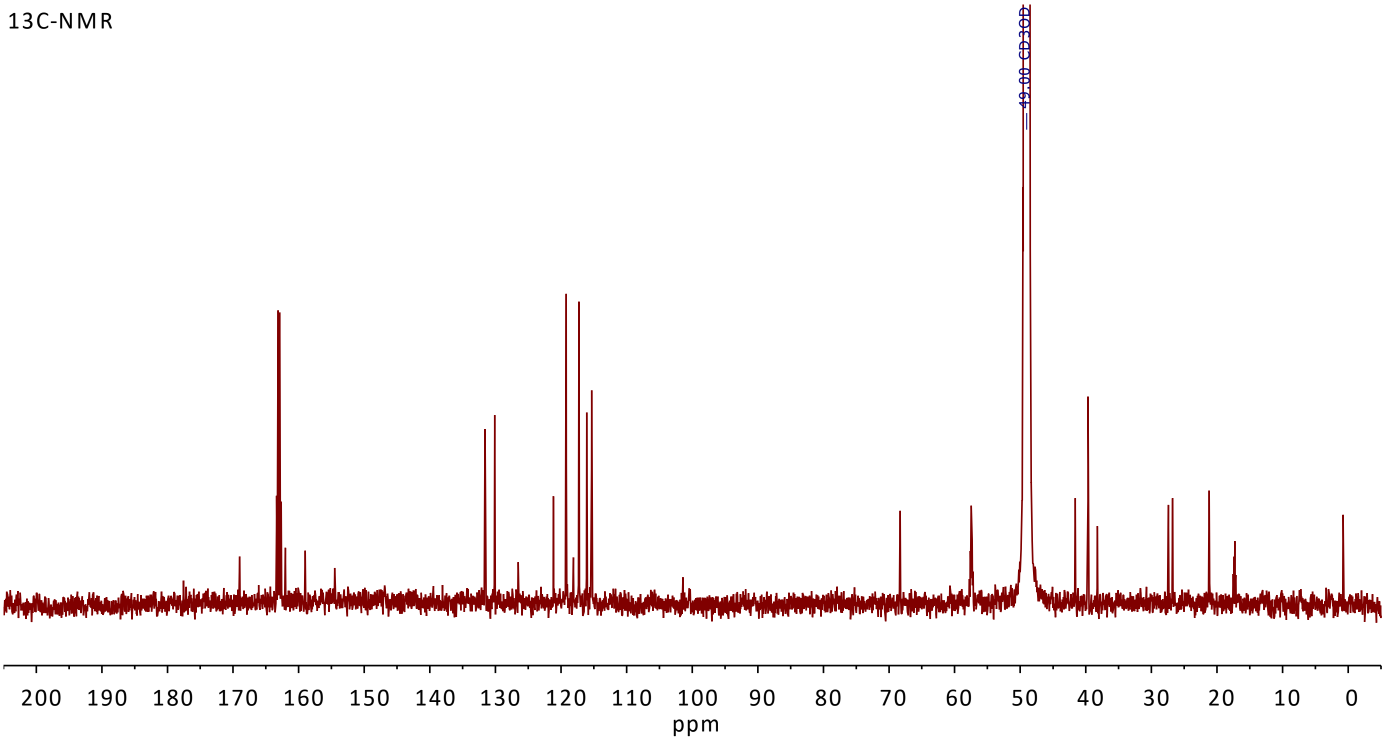

254 nm (LC-MS)

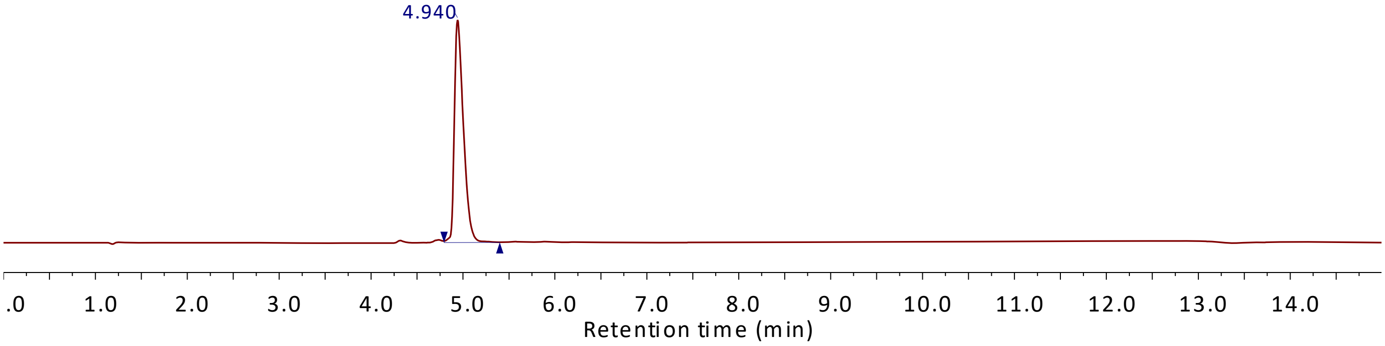

6cc

1H-NMR

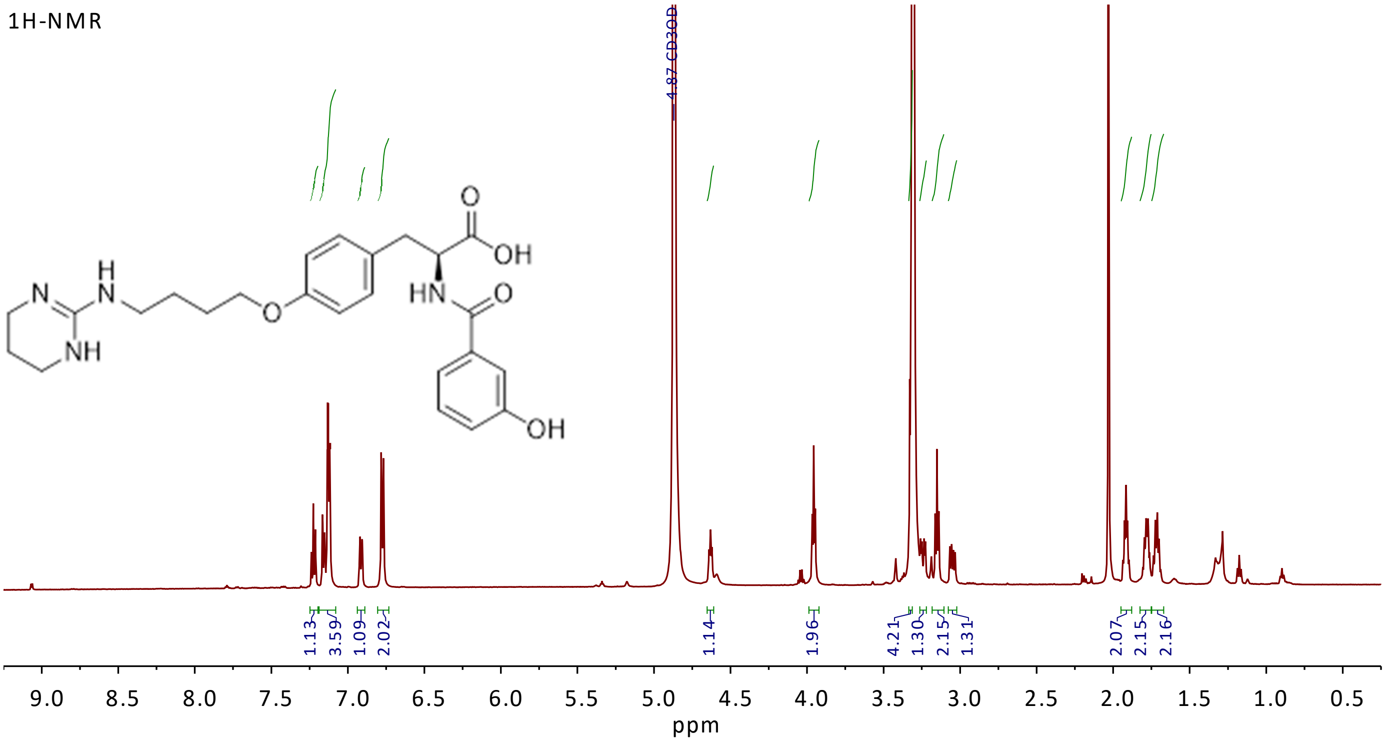

13C-NMR

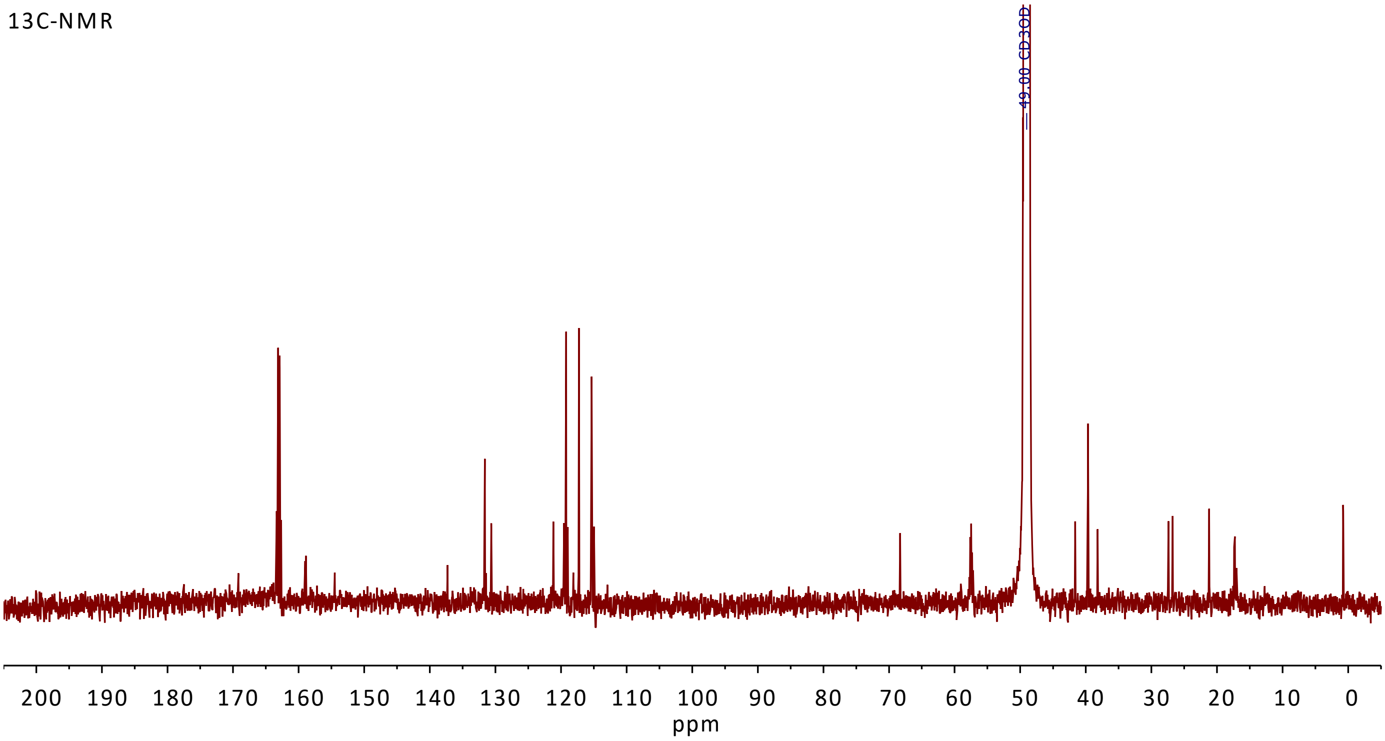

254 nm (LC-MS)

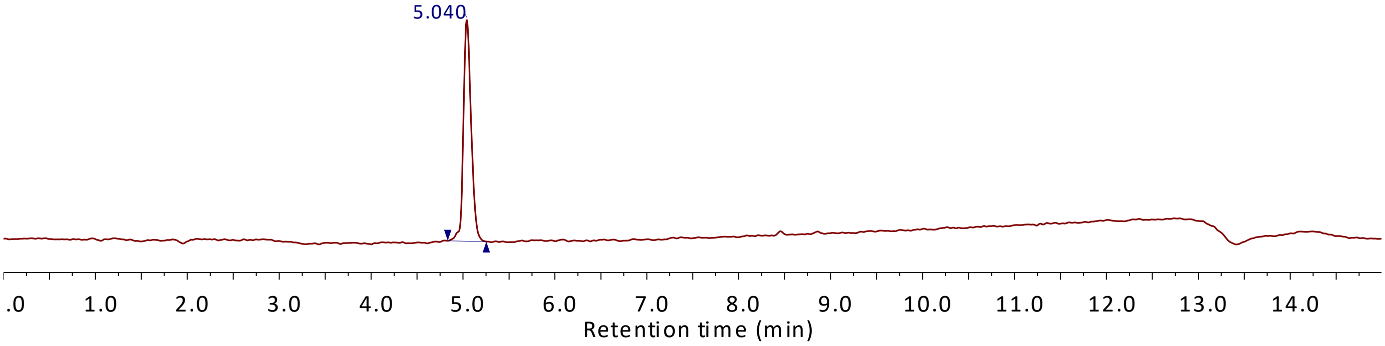

6dc

1H-NMR

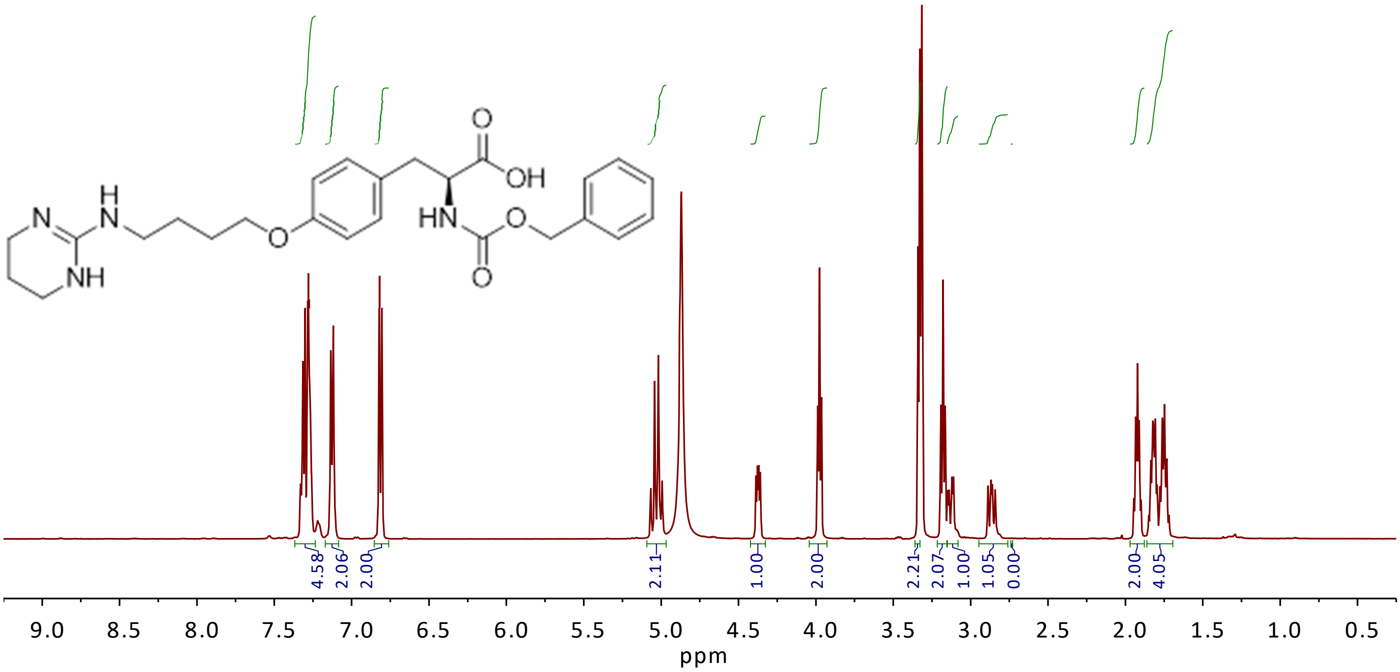

13C-NMR

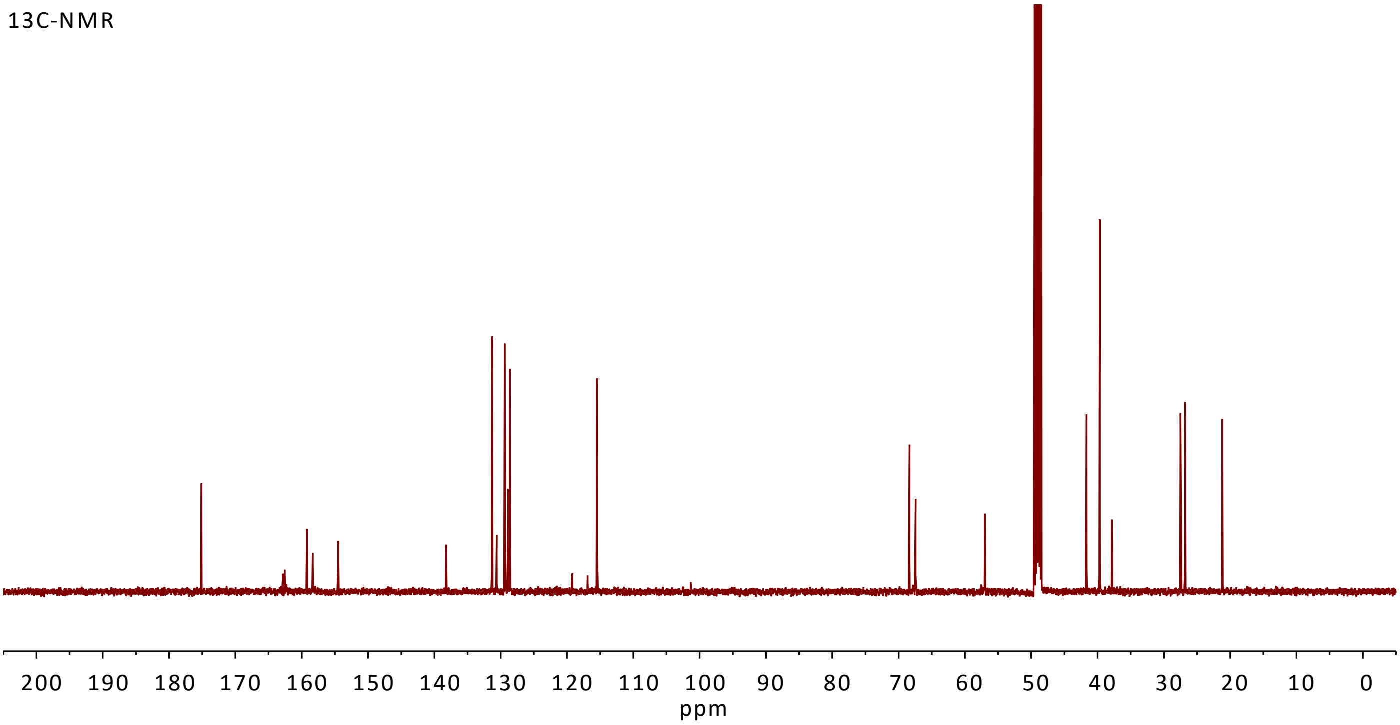

220 nm (LC-MS)

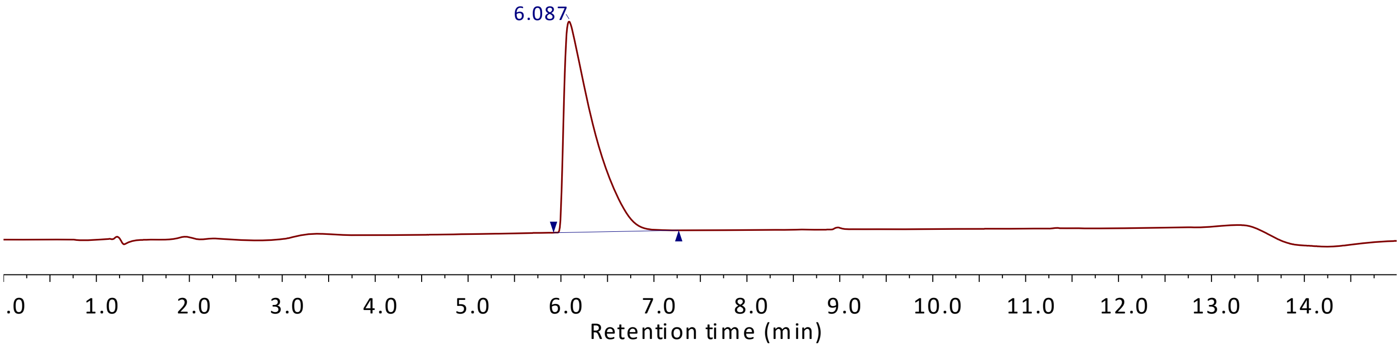

7ac

1H-NMR

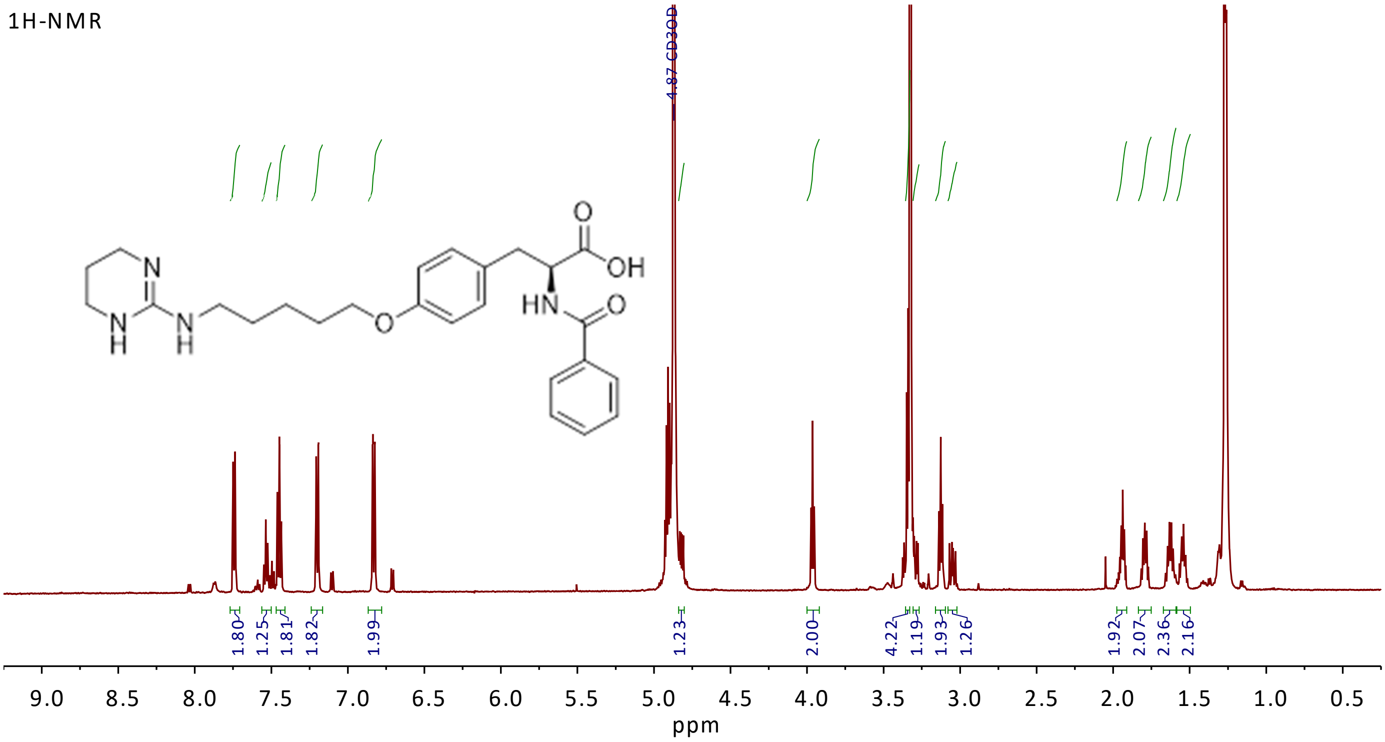

13C-NMR

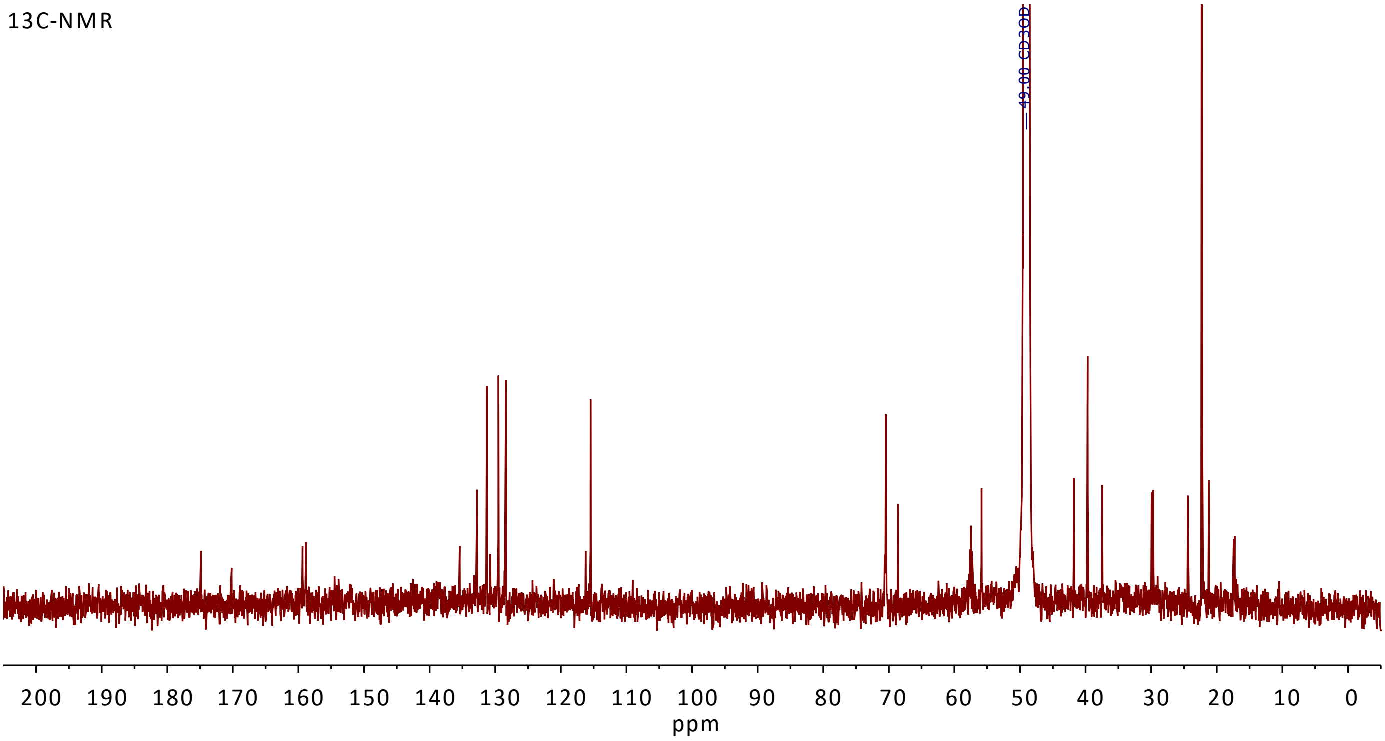

254 nm (LC-MS)

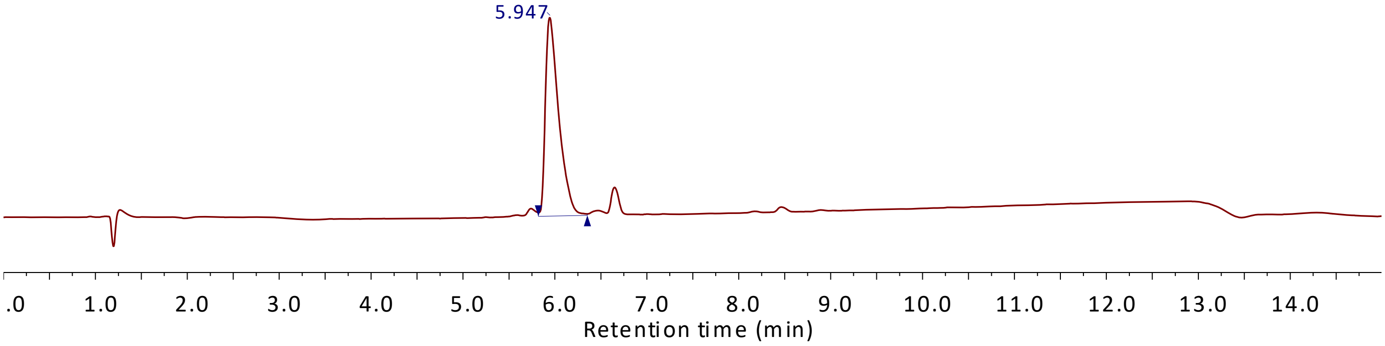

7bc

1H-NMR

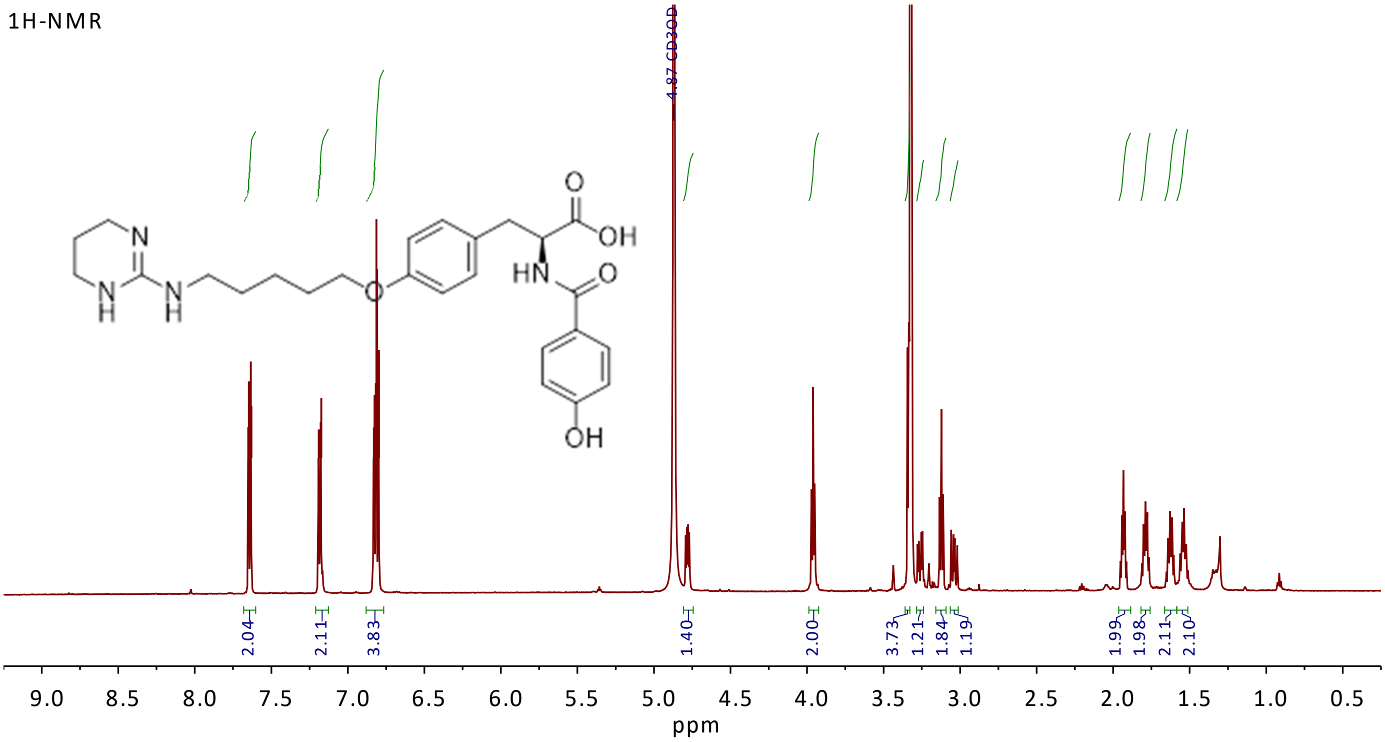

13C-NMR

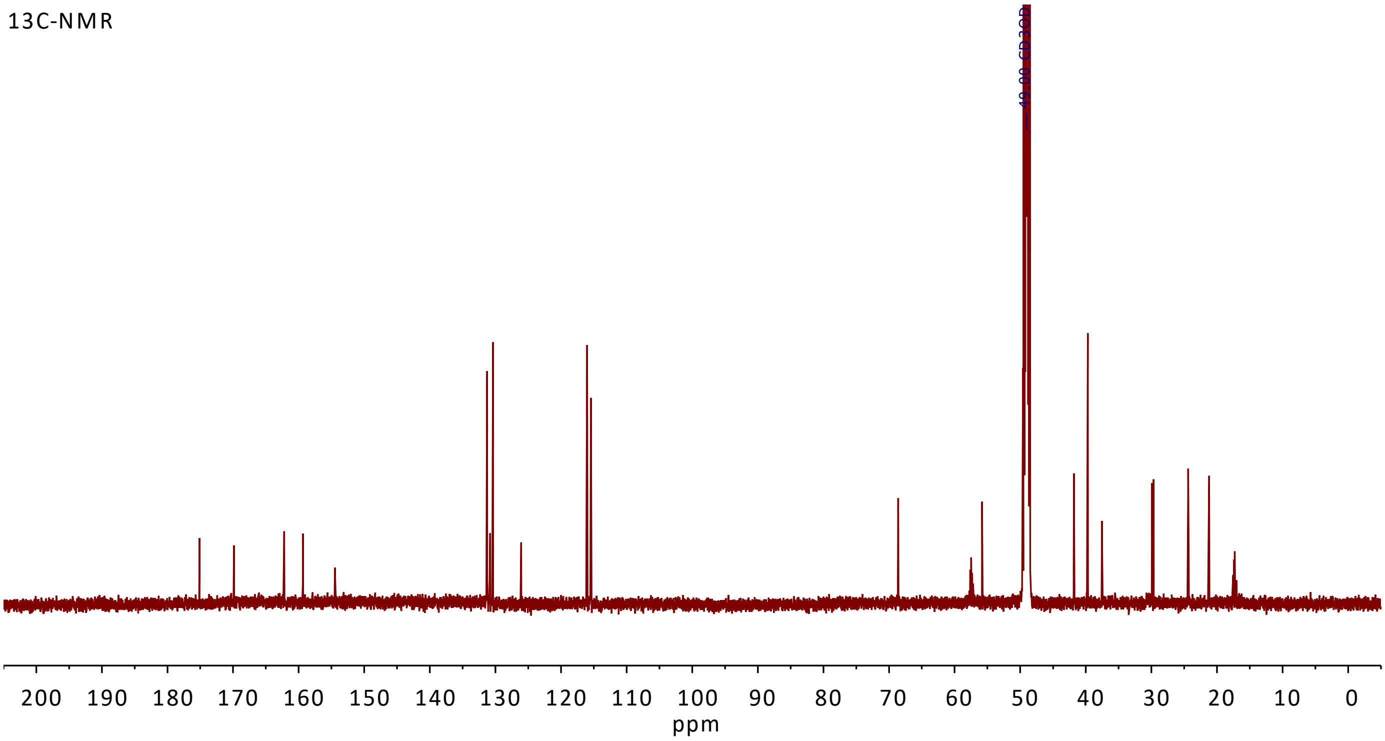

254 nm (LC-MS)

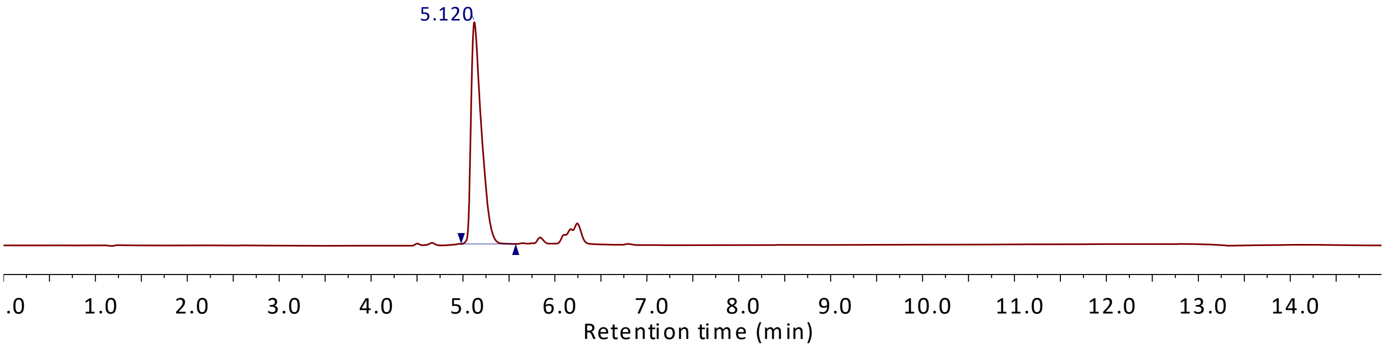

7cc

1H-NMR

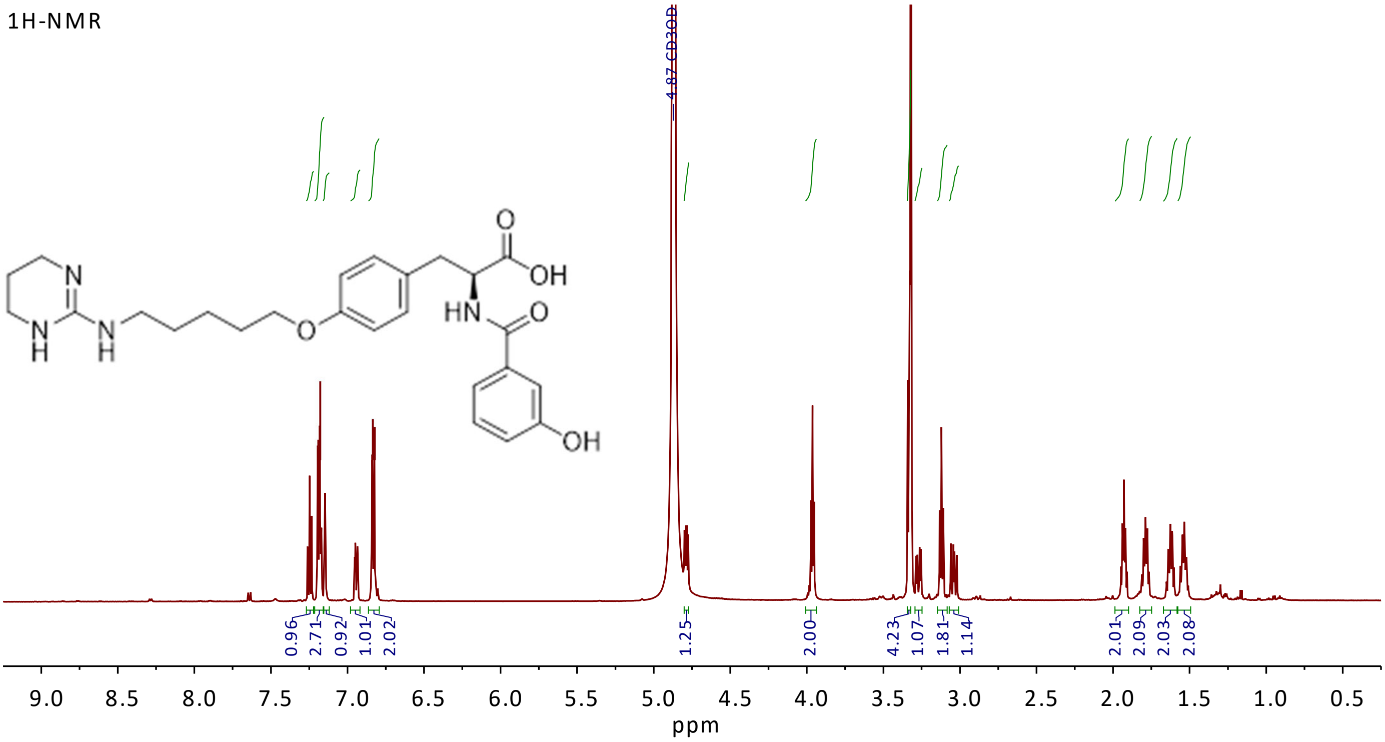

13C-NMR

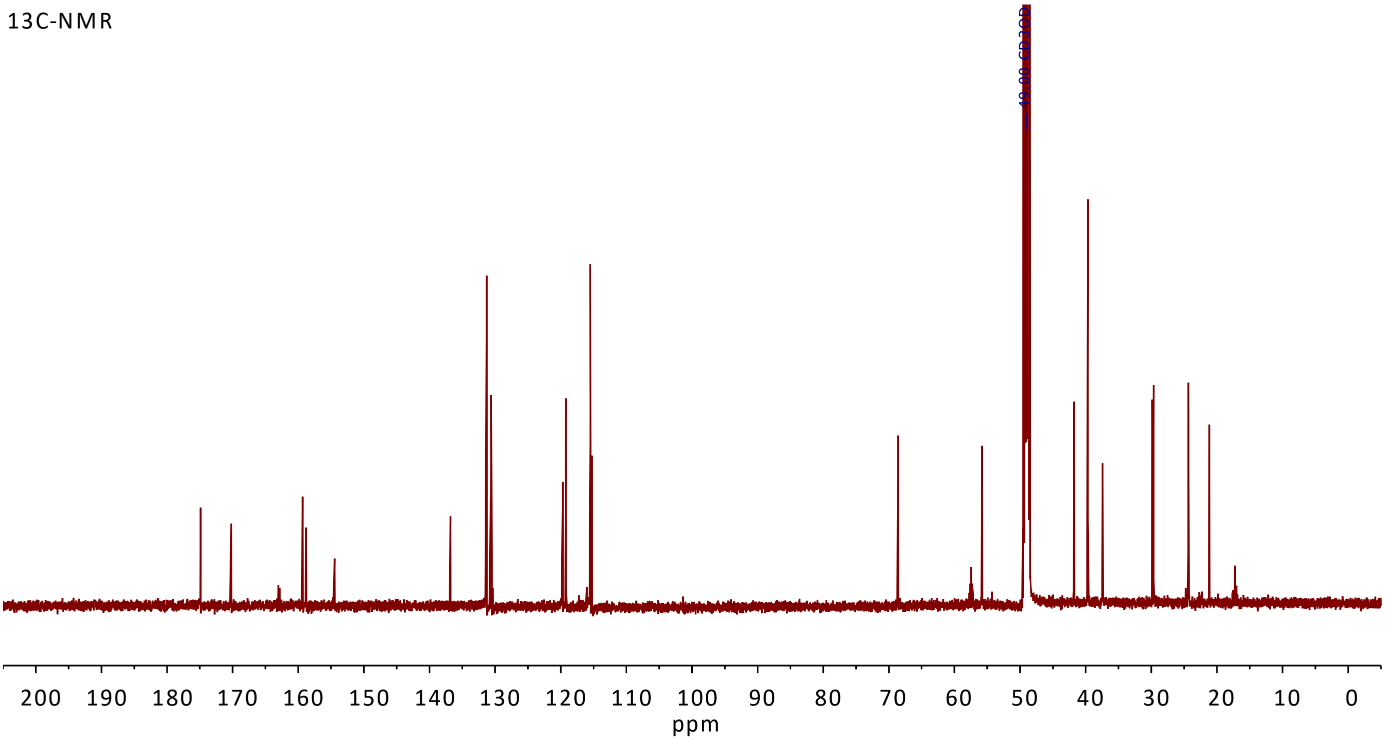

254 nm (LC-MS)

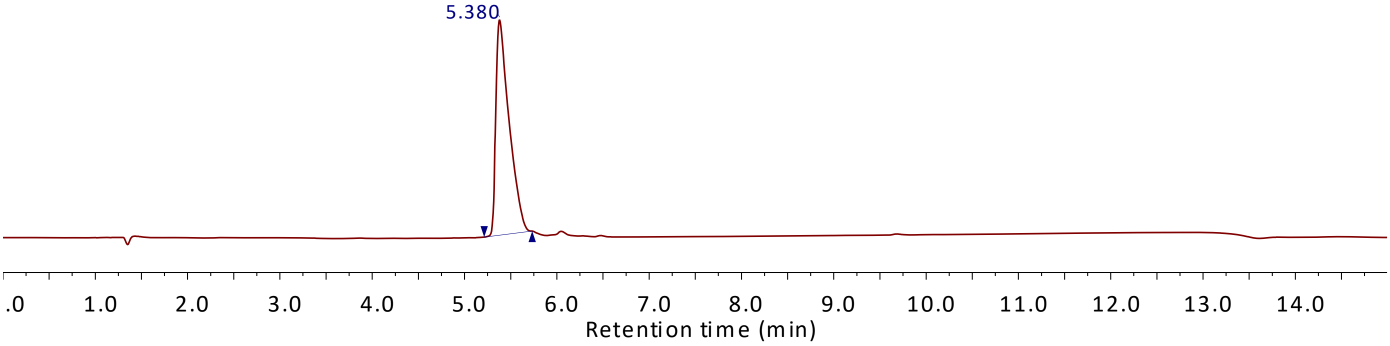

7dc

1H-NMR

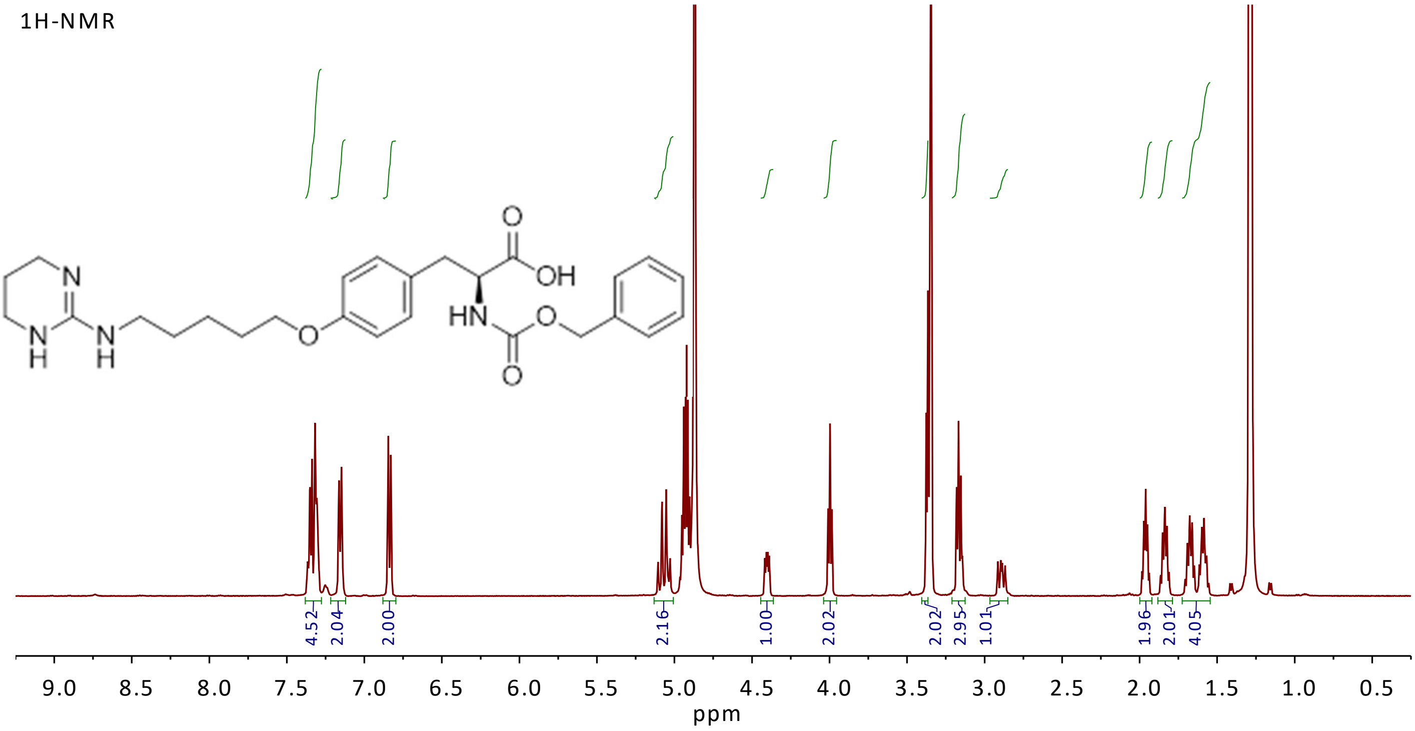

13C-NMR

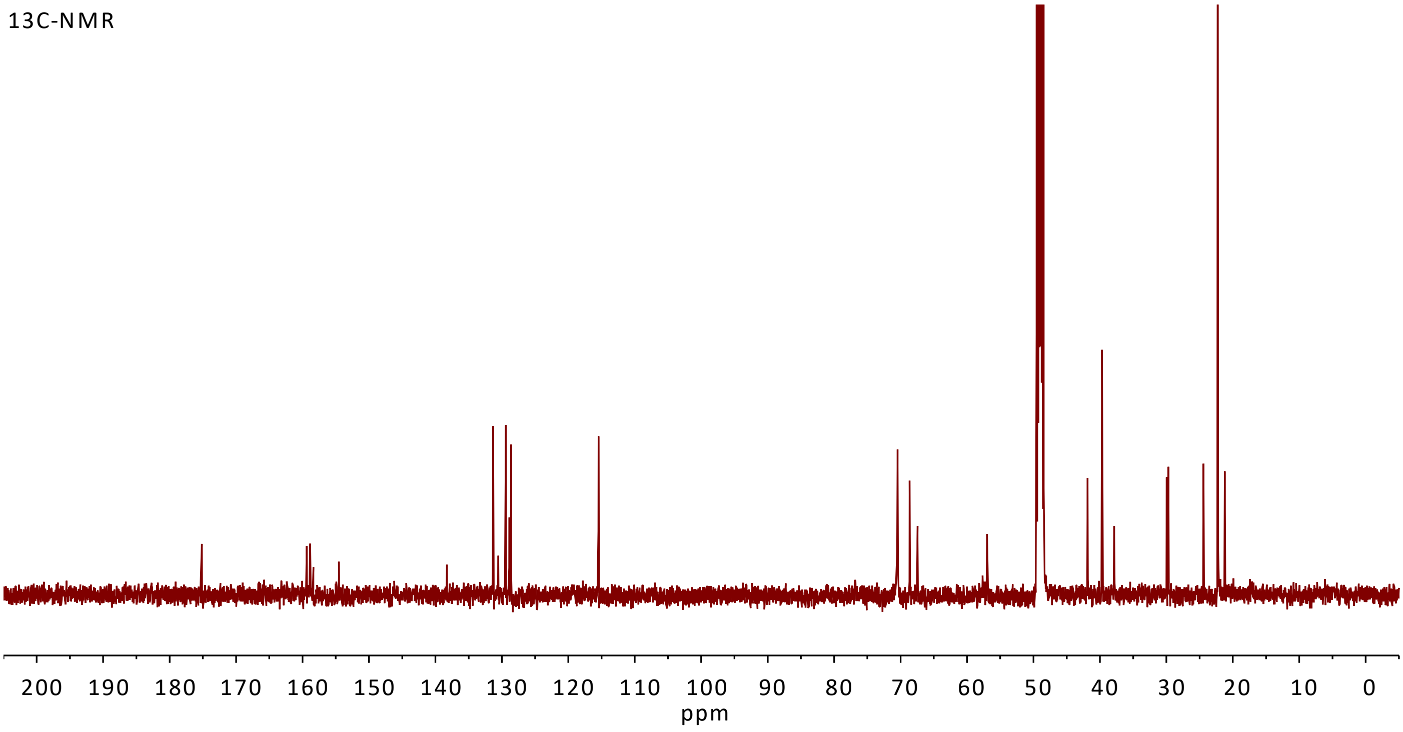

220 nm (LC-MS)

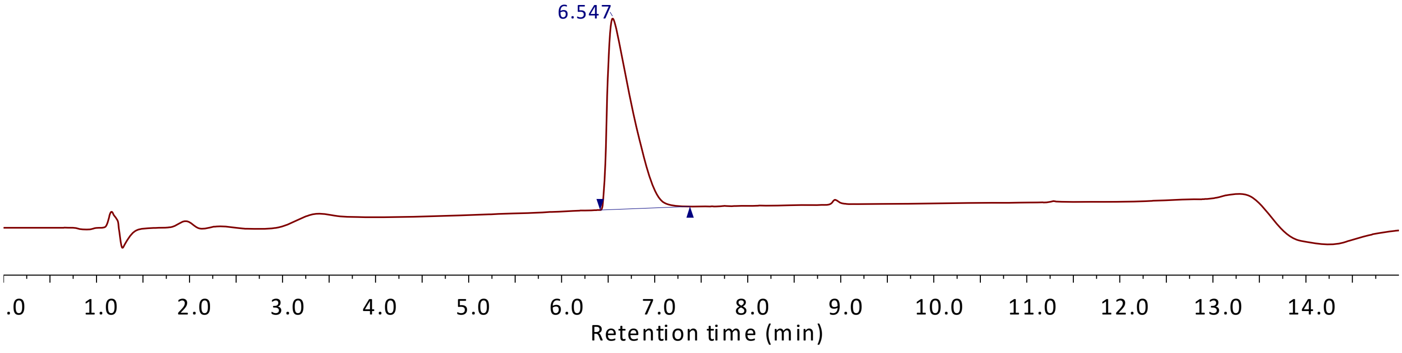

24a

1H-NMR

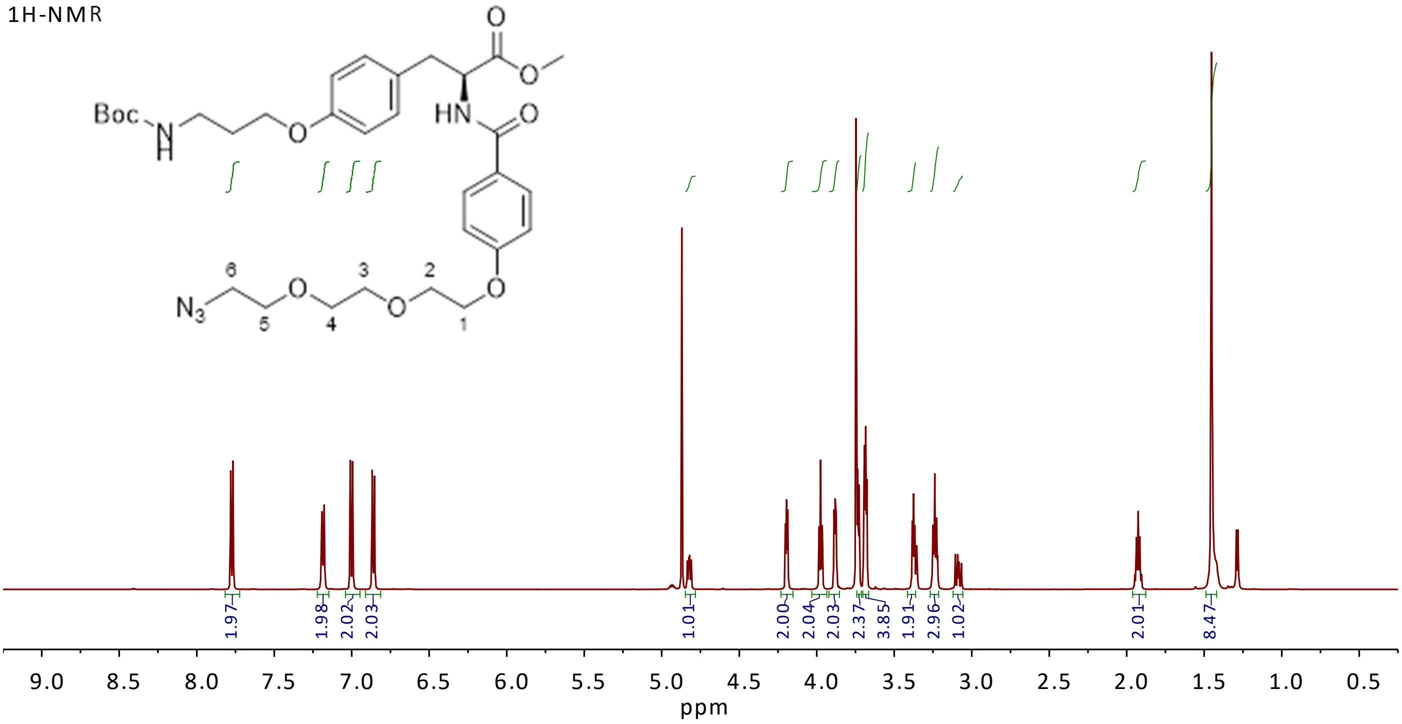

13C-NMR

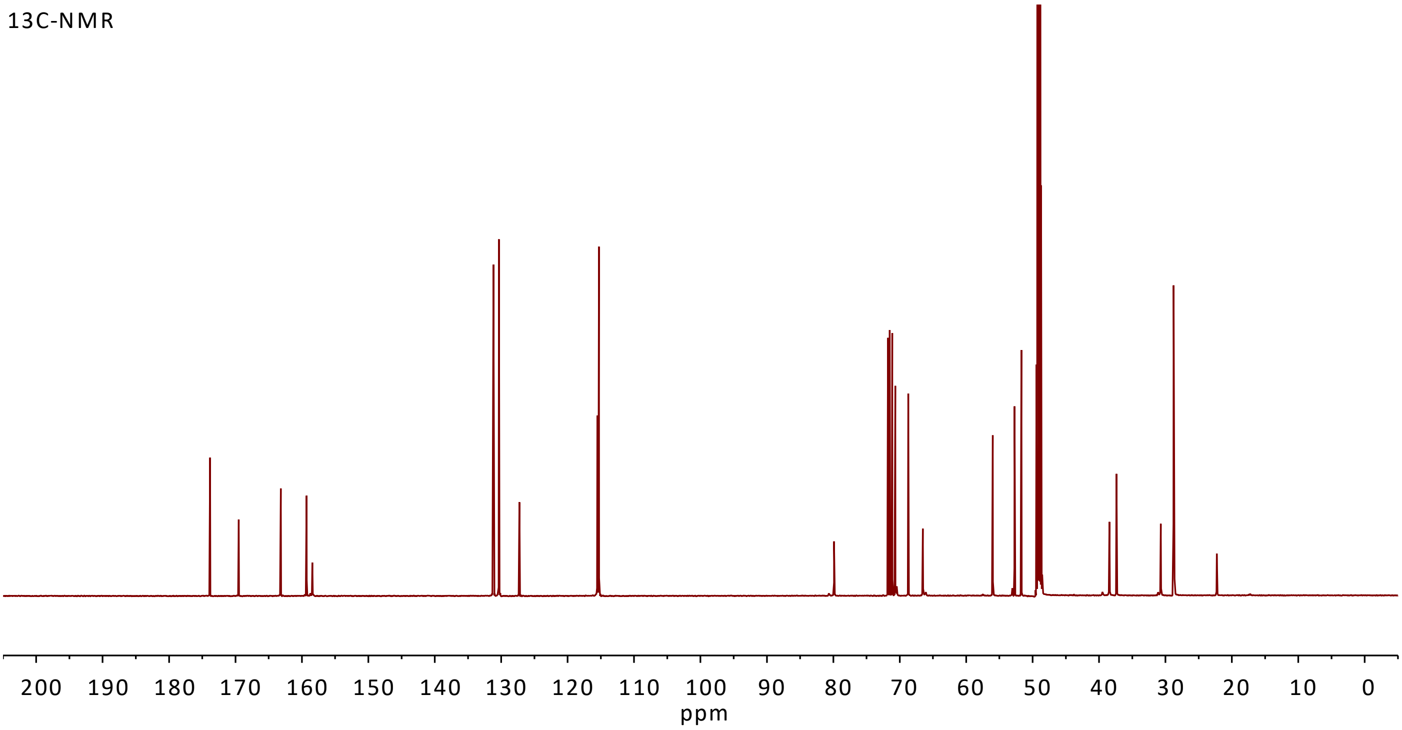

254 nm (LC-MS)

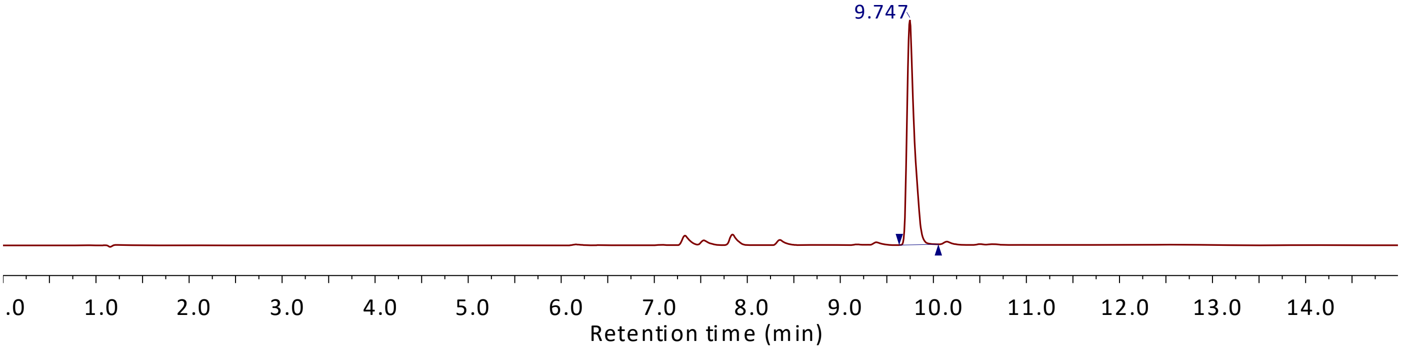

24b

1H-NMR

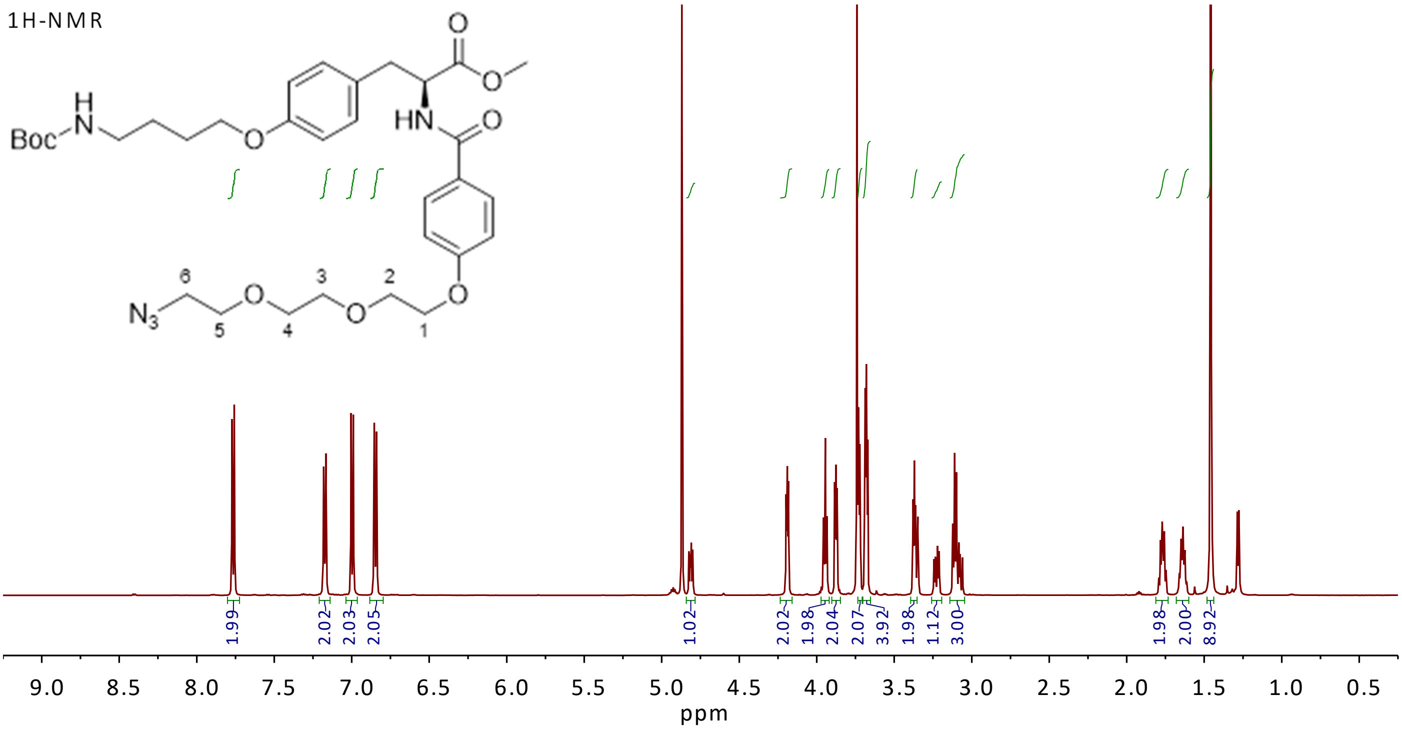

13C-NMR

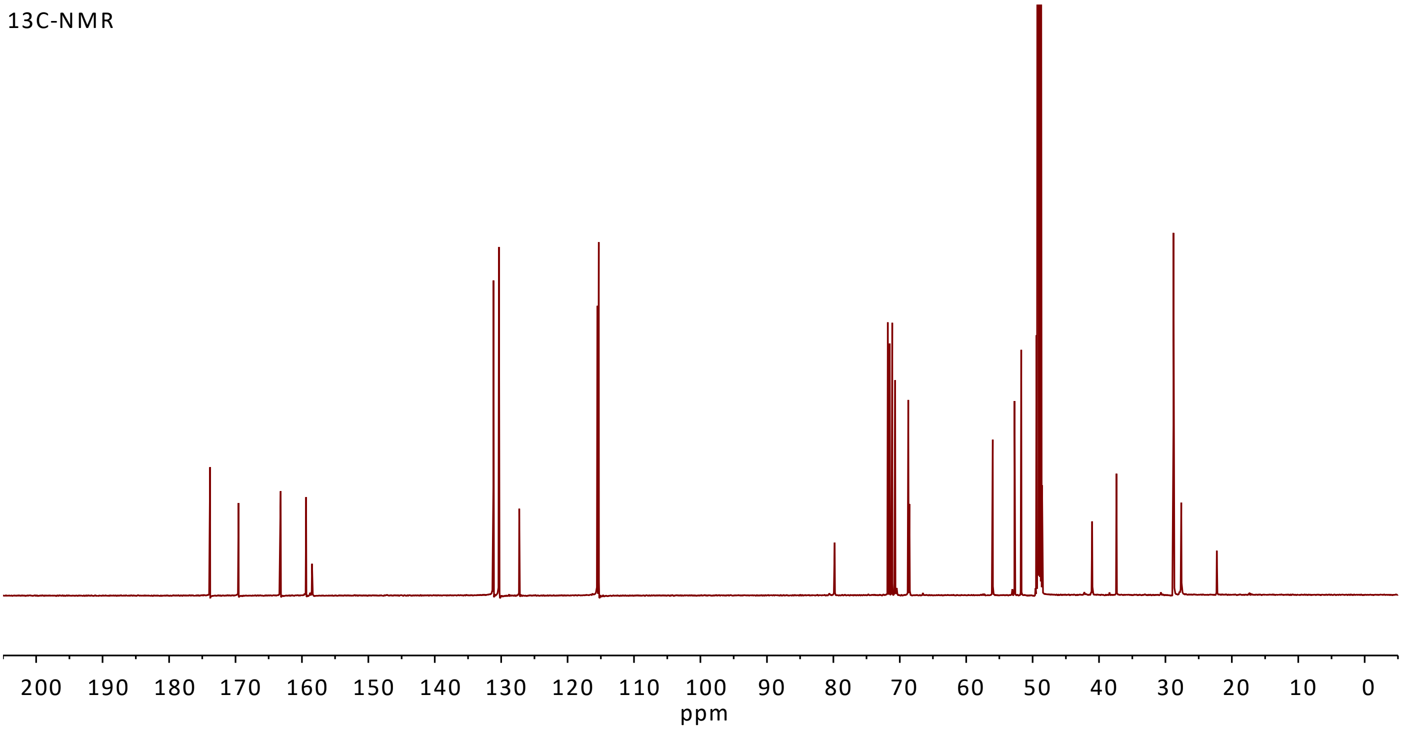

254 nm (LC-MS)

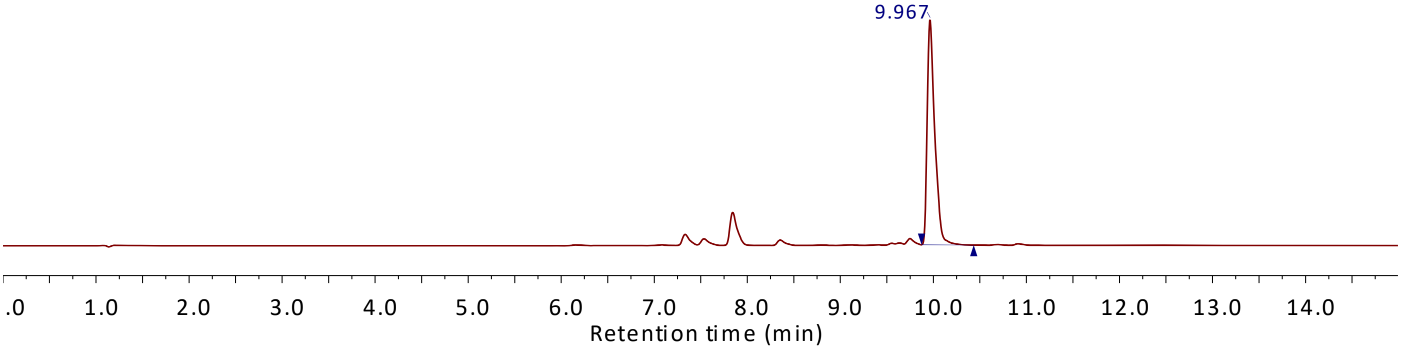

24c

1H-NMR

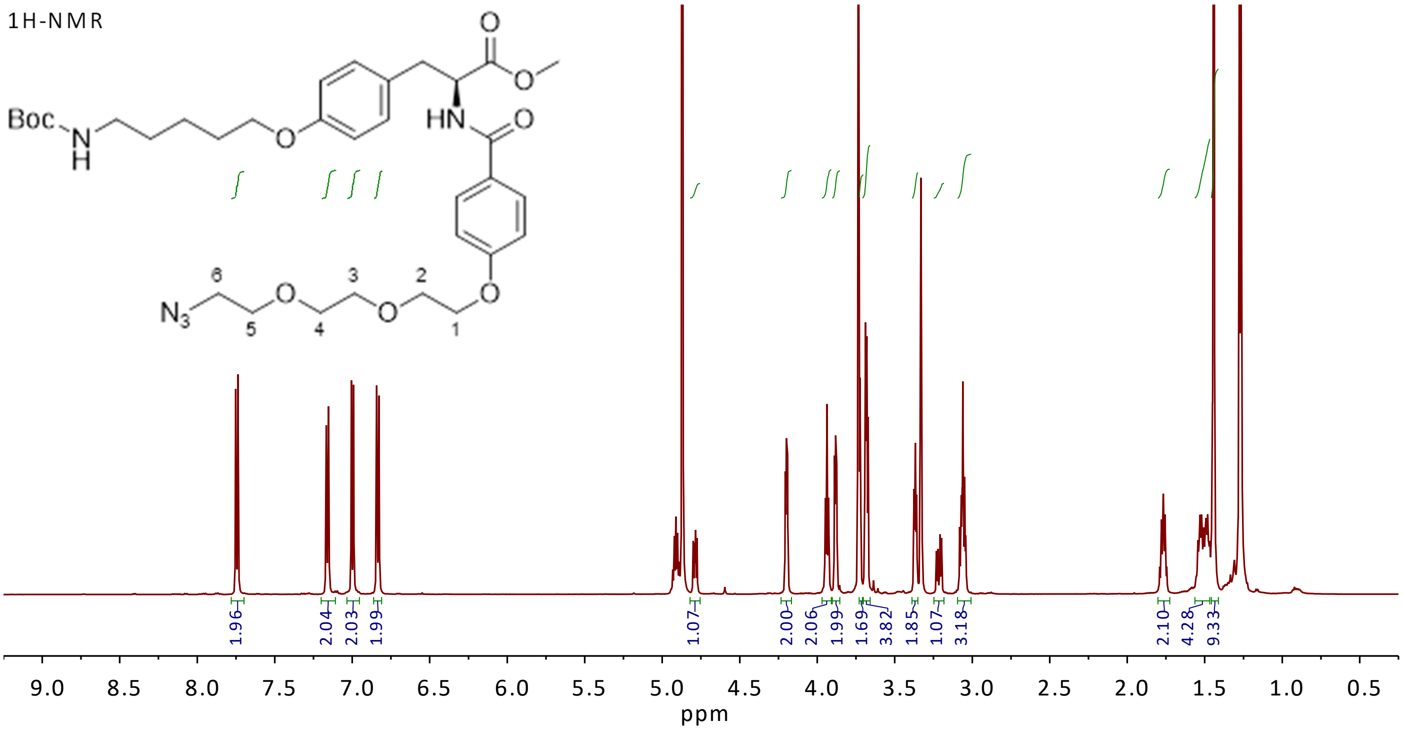

13C-NMR

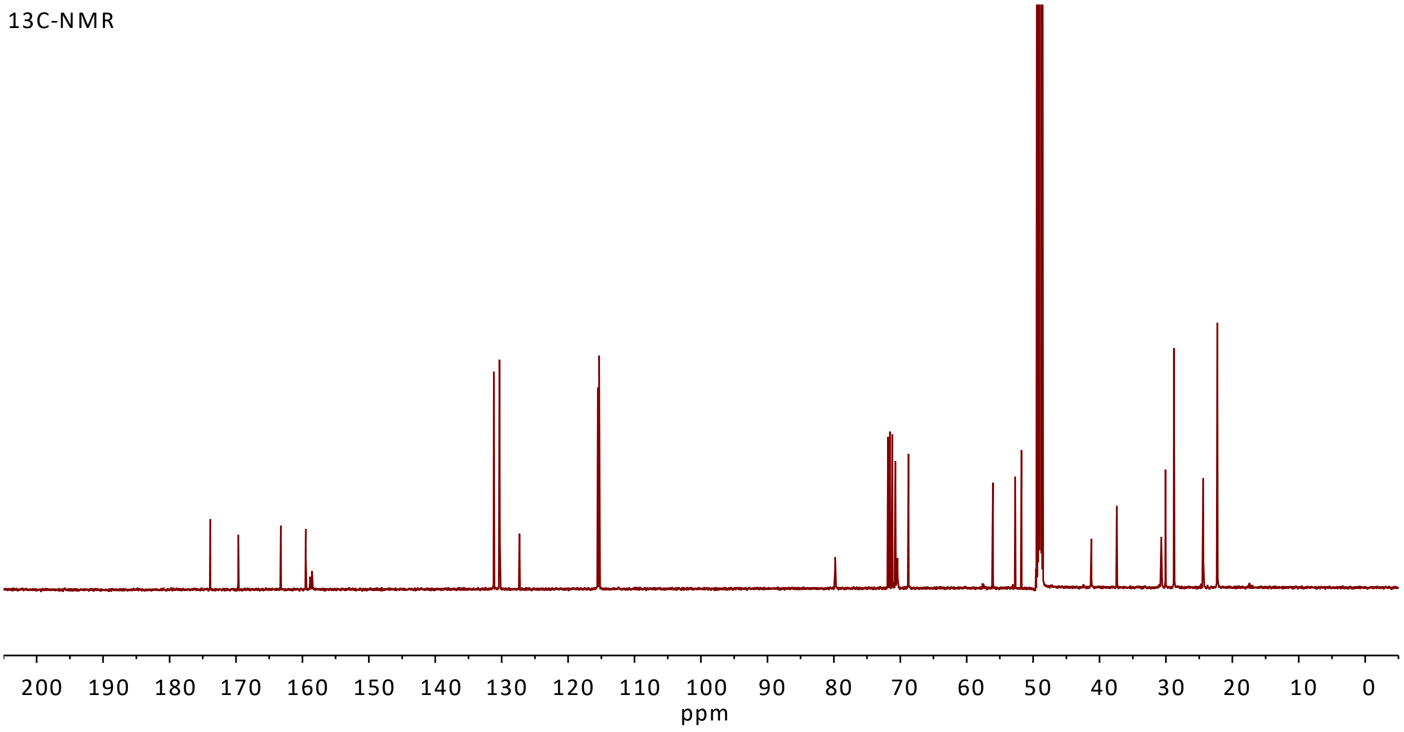

254 nm (LC-MS)

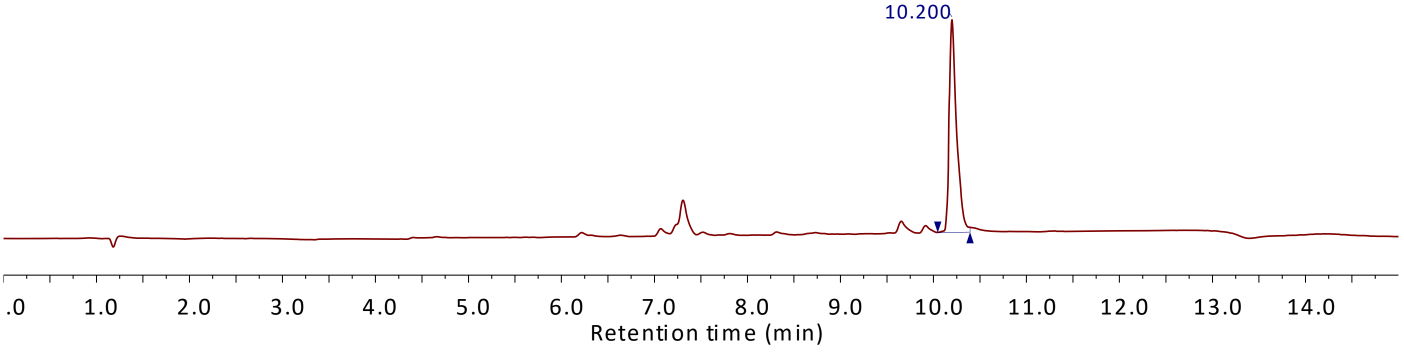

10a

1H-NMR

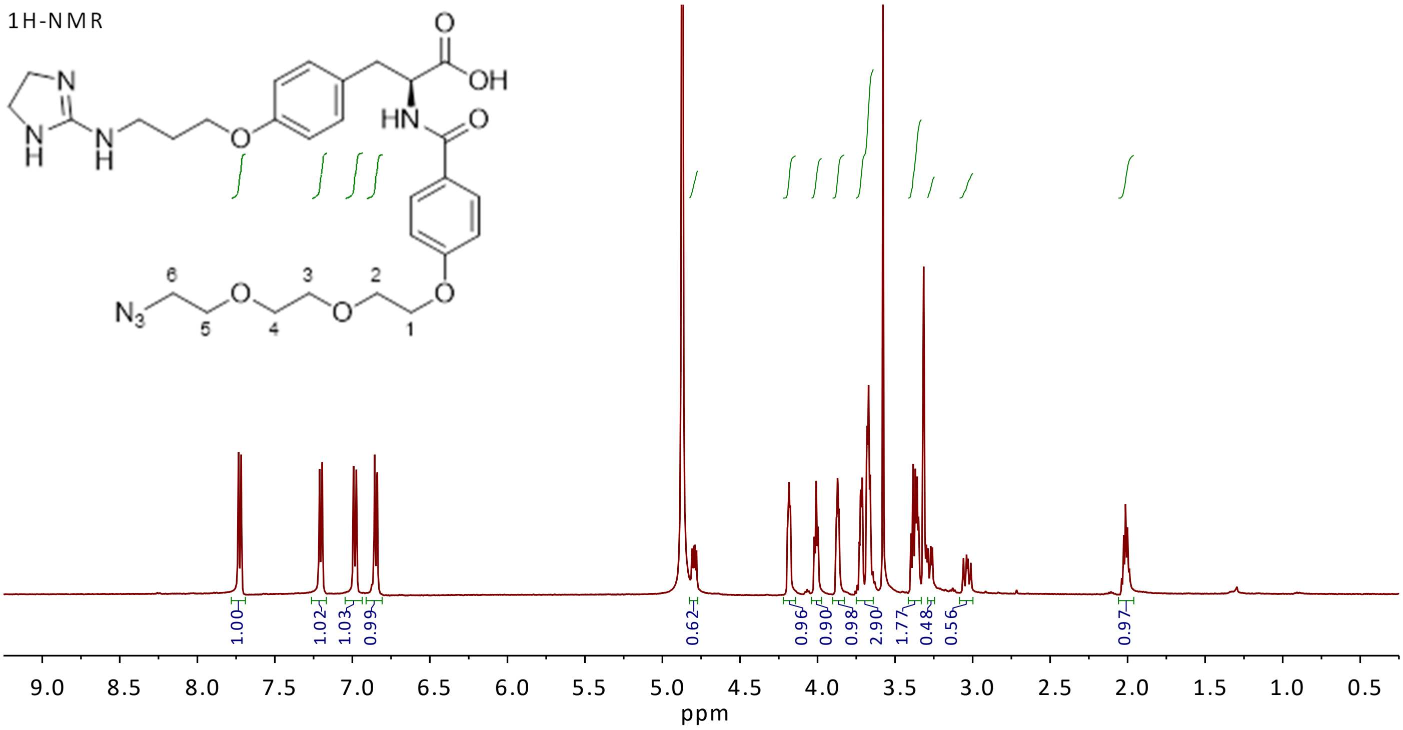

13C-NMR

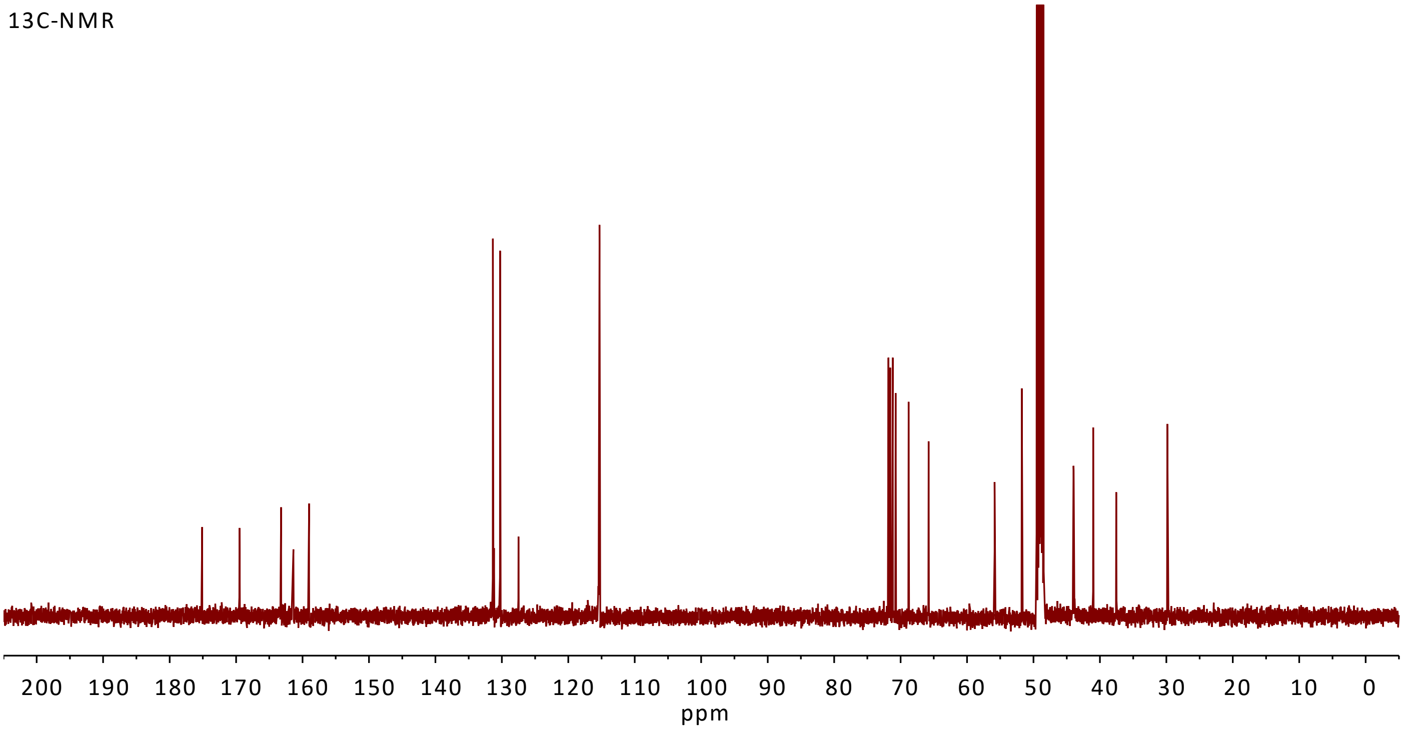

220 nm (LC-MS)

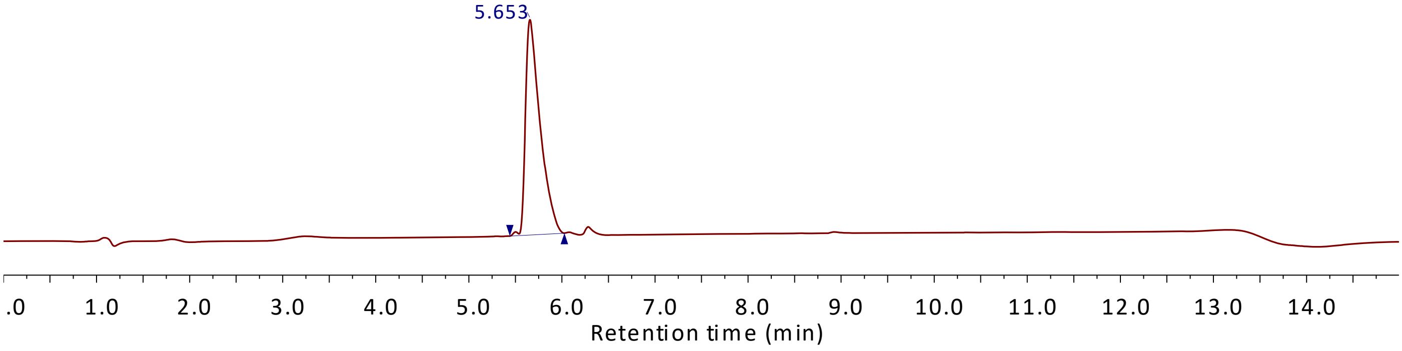

10b

1H-NMR

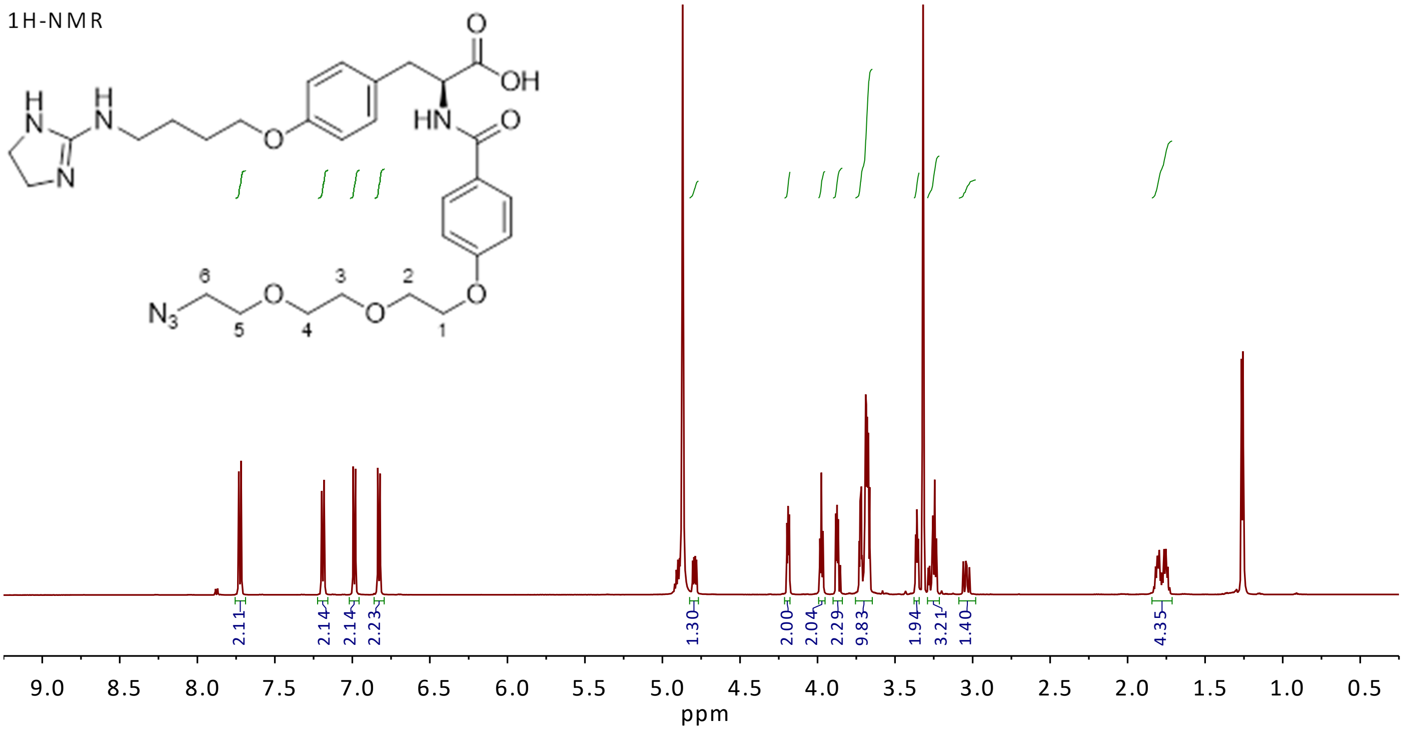

13C-NMR

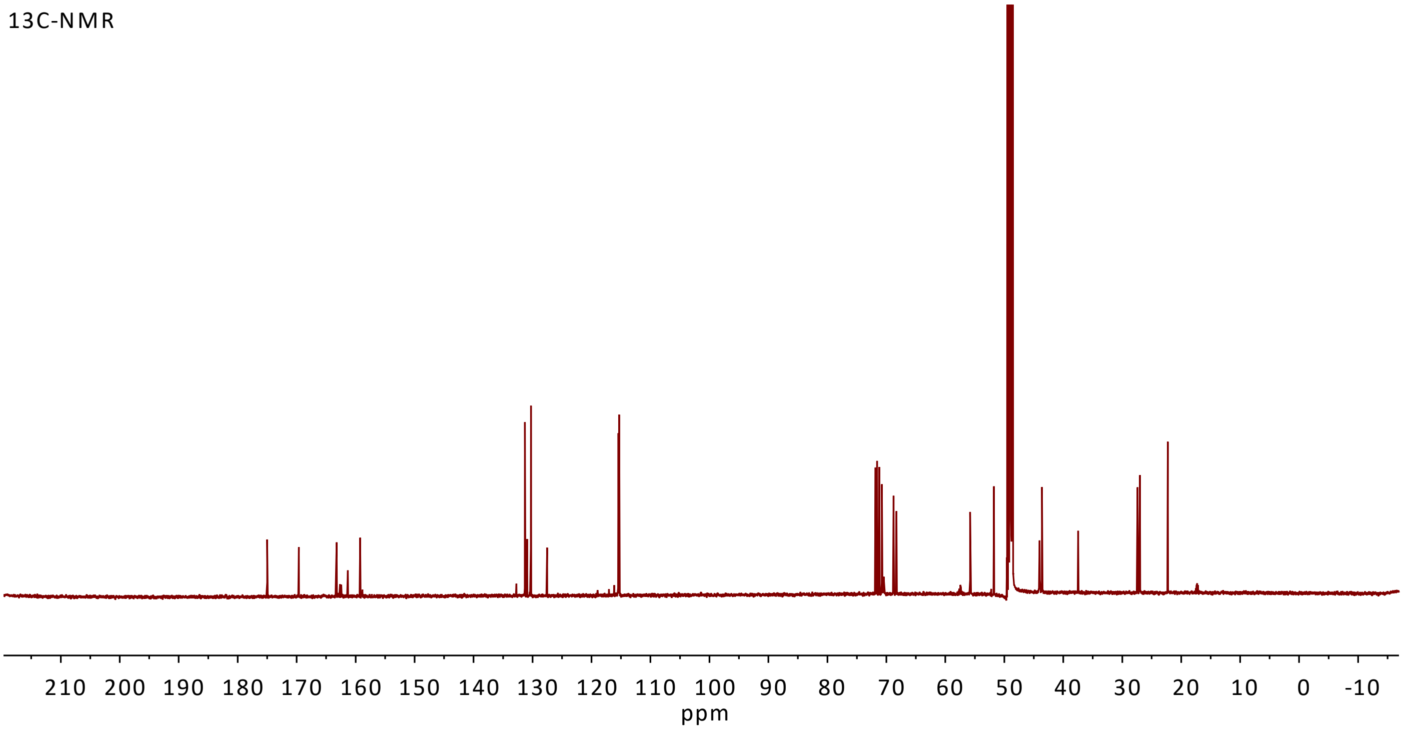

220 nm (LC-MS)

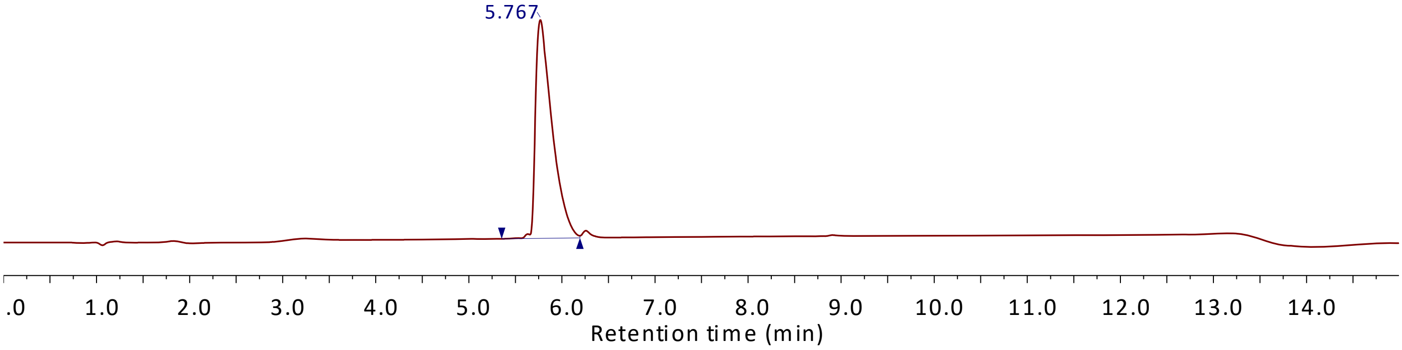

10c

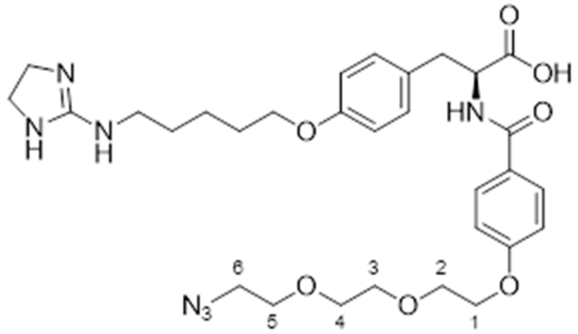

220 nm (LC-MS)

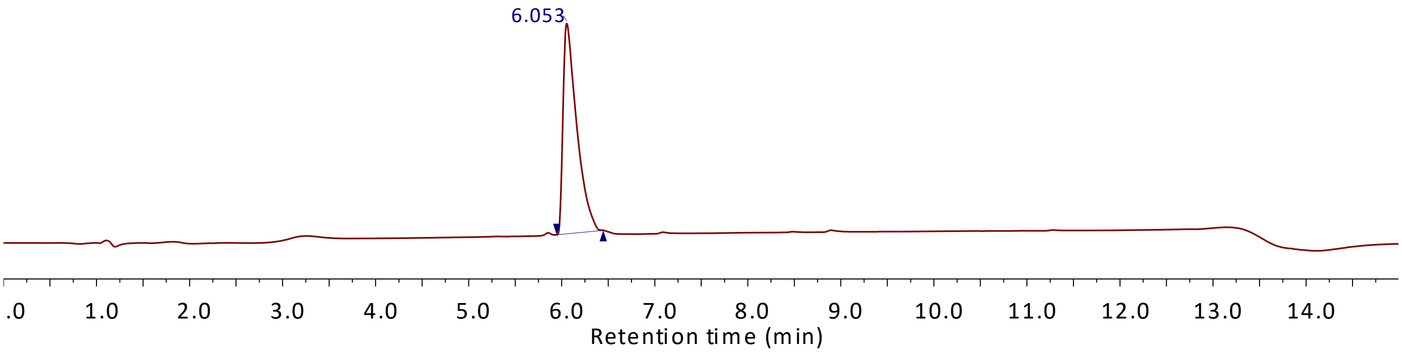

14

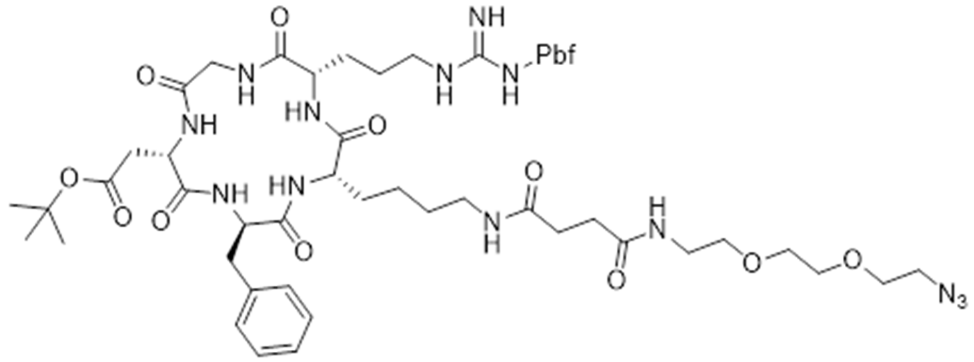

220 nm (LC-MS)

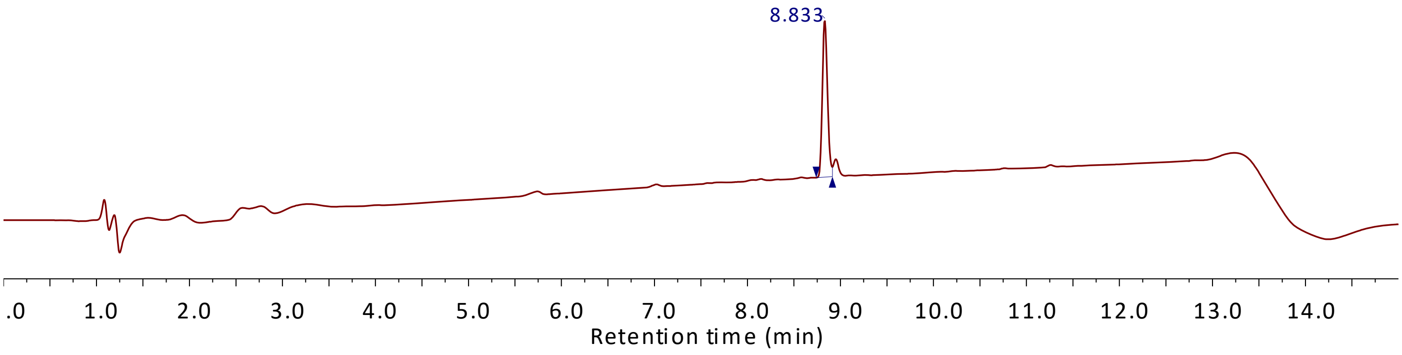

15

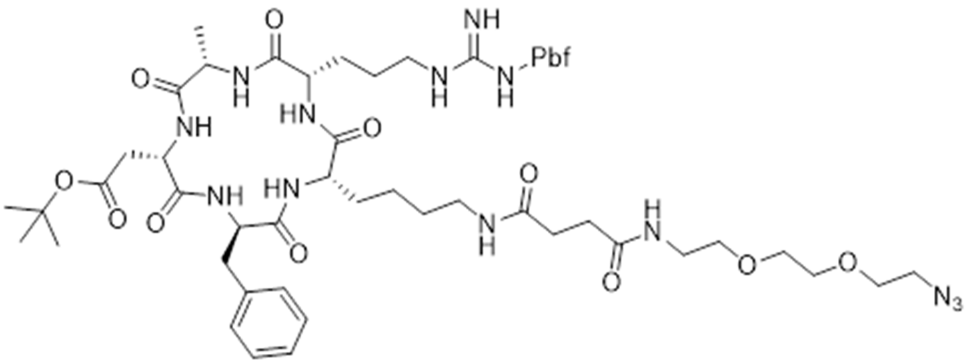

220 nm (LC-MS)

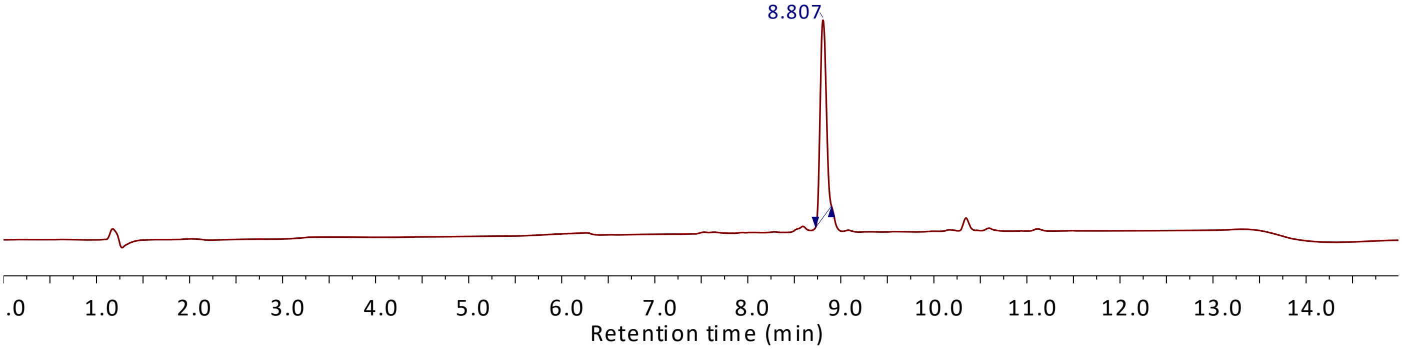

12

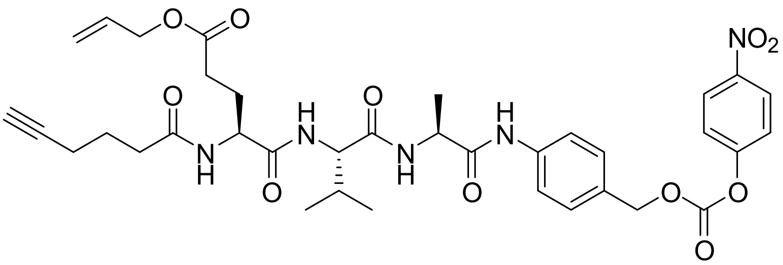

254 nm (LC-MS)

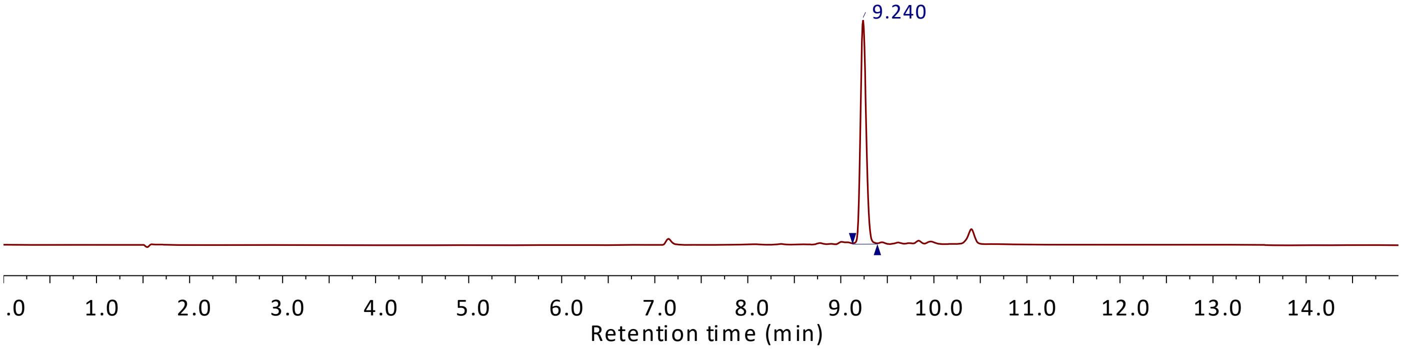

13

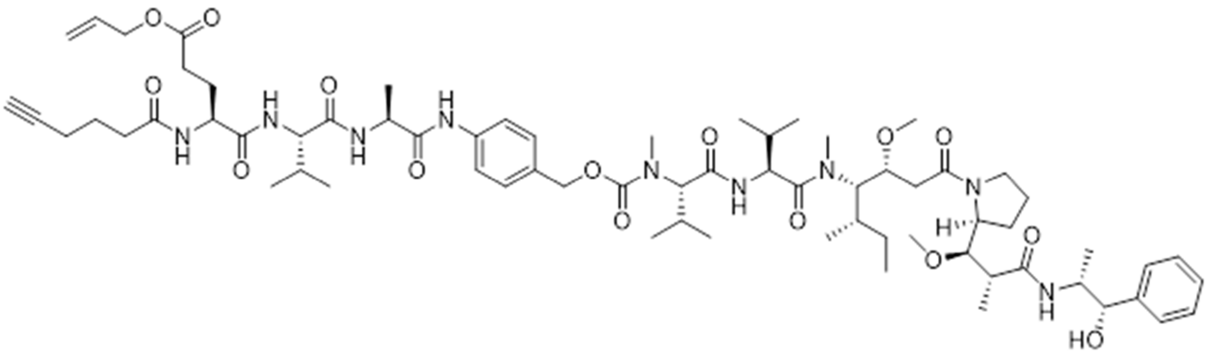

254 nm (LC-MS)

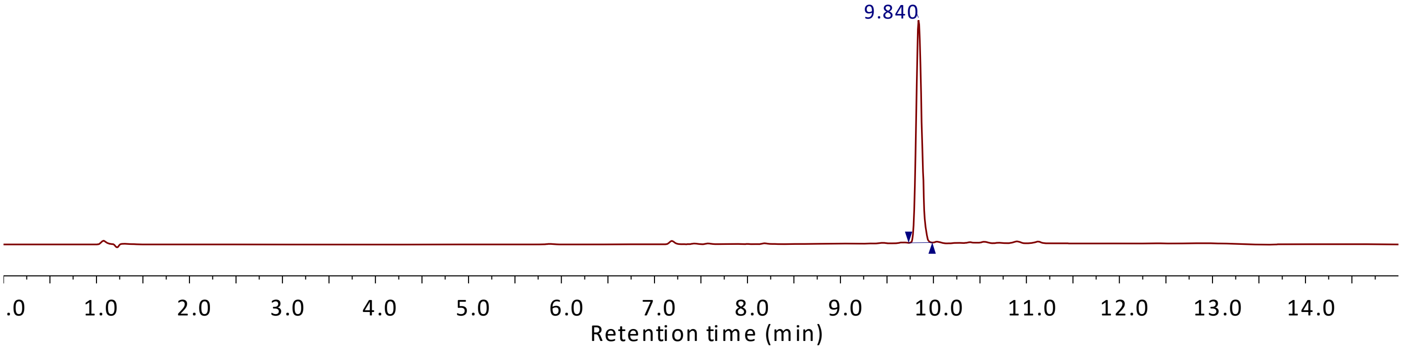

16

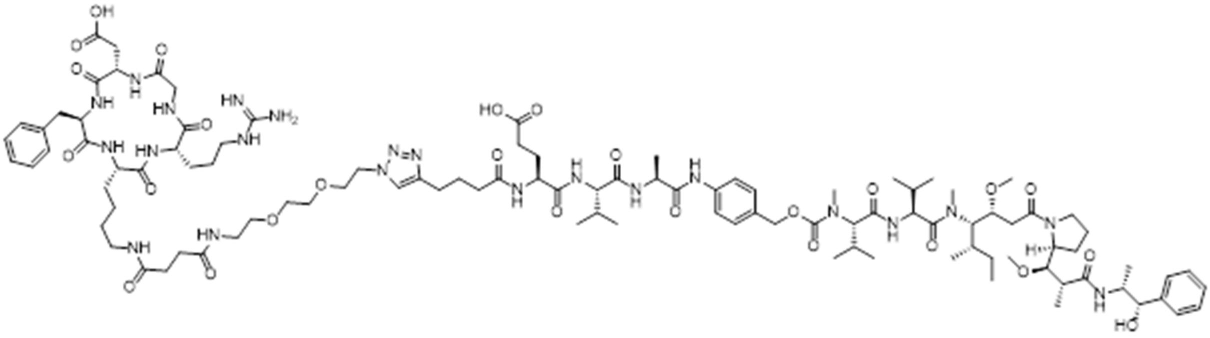

254 nm (LC-MS)

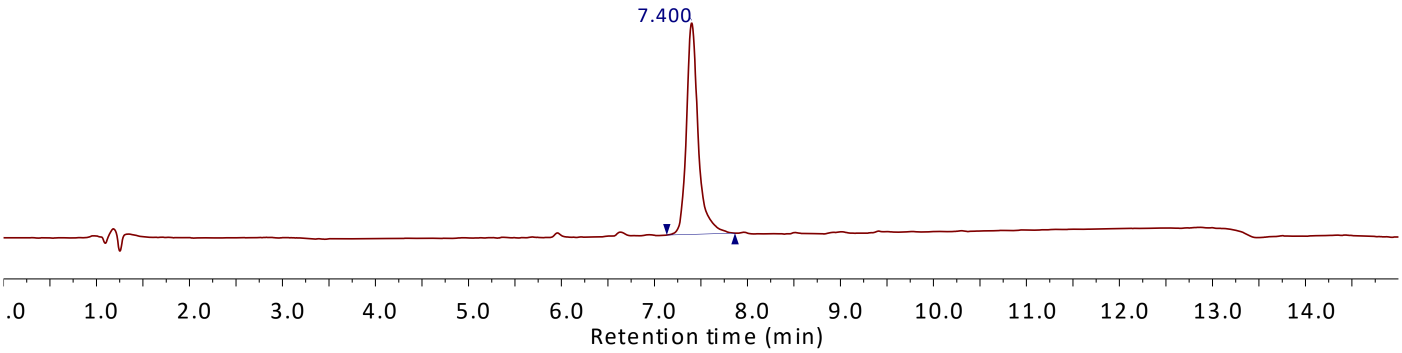

17

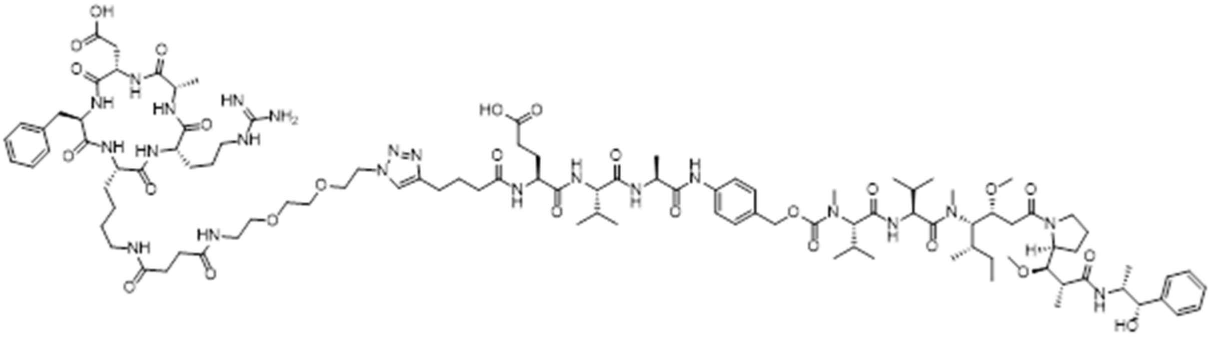

254 nm (LC-MS)

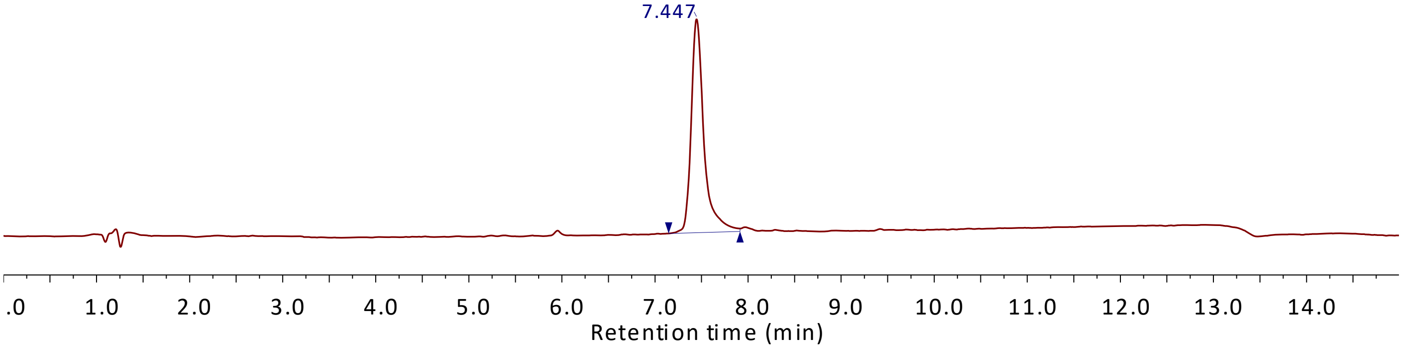

18

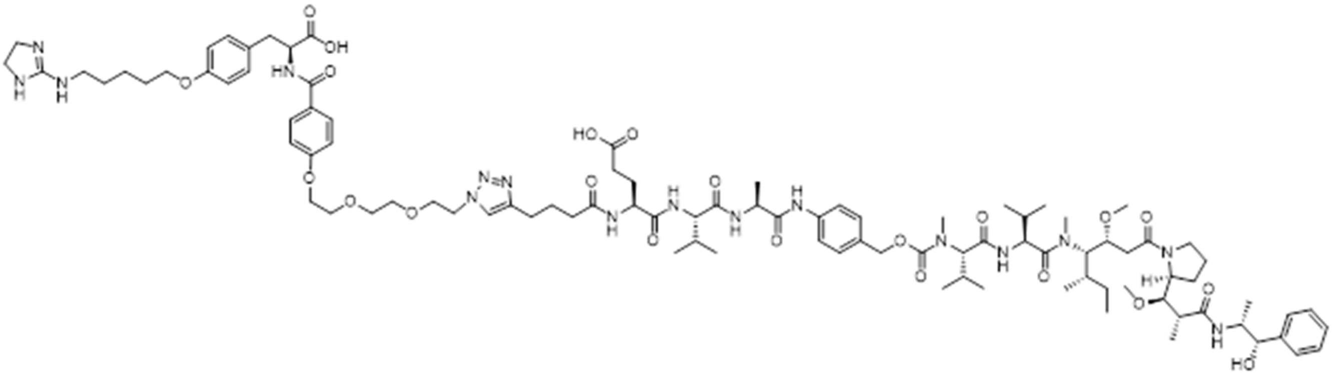

254 nm (LC-MS)

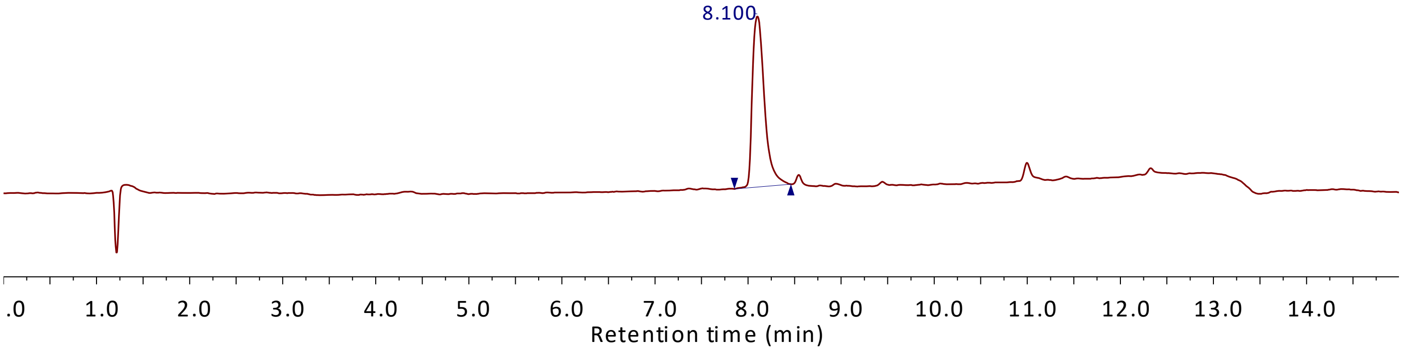

Supplement: Supplementary file 1 [file DataSheet1.pdf]
